# A comprehensive and comparative phenotypic analysis of the collaborative founder strains identifies new and known phenotypes

**DOI:** 10.1007/s00335-020-09827-3

**Published:** 2020-02-14

**Authors:** Heike Kollmus, Helmut Fuchs, Christoph Lengger, Hamed Haselimashhadi, Molly A. Bogue, Manuela A. Östereicher, Marion Horsch, Thure Adler, Juan Antonio Aguilar-Pimentel, Oana Veronica Amarie, Lore Becker, Johannes Beckers, Julia Calzada-Wack, Lillian Garrett, Wolfgang Hans, Sabine M. Hölter, Tanja Klein-Rodewald, Holger Maier, Philipp Mayer-Kuckuk, Gregor Miller, Kristin Moreth, Frauke Neff, Birgit Rathkolb, Ildikó Rácz, Jan Rozman, Nadine Spielmann, Irina Treise, Dirk Busch, Jochen Graw, Thomas Klopstock, Eckhard Wolf, Wolfgang Wurst, Ali Önder Yildirim, Jeremy Mason, Arturo Torres, Mouse Phenome Database Team, Rudi Balling, Terry Mehaan, Valerie Gailus-Durner, Klaus Schughart, Martin Hrabě de Angelis

**Affiliations:** 1 Department of Infection Genetics, Helmholtz Centre for Infection Research, Inhoffenstr.7, 38124 Braunschweig, Germany; 2 German Mouse Clinic, Institute of Experimental Genetics, Helmholtz Zentrum München, German Research Center for Environmental Health, Ingolstädter Landstrasse 1, 85764 Neuherberg, Germany; 3 European Molecular Biology Laboratory, European Bioinformatics Institute, Wellcome Trust Genome Campus, Hinxton, Cambridge, CB10 1SD UK; 4 The Jackson Laboratory, Bar Harbor, ME 04609 USA; 5 Institute of Developmental Genetics, Helmholtz Zentrum München, German Research Center for Environmental Health, Ingolstädter Landstrasse 1, 85764 Neuherberg, Germany; 6 Chair of Experimental Genetics, School of Life Science Weihenstephan, Technische Universität München, Alte Akademie 8, 85354 Freising, Germany; 7 German Center for Diabetes Research (DZD), Ingolstädter Landstr. 1, 85764 Neuherberg, Germany; 8 Institute of Molecular Animal Breeding and Biotechnology, Gene Center, Ludwig-Maximilians-University München, Feodor-Lynen Str. 25, 81377 Munich, Germany; 9 Institute for Medical Microbiology, Immunology and Hygiene, Technische Universität München, Trogerstrasse 30, 81675 Munich, Germany; 10 Department of Neurology, Friedrich-Baur-Institute, Klinikum Der Ludwig-Maximilians-Universität München, Ziemssenstr. 1a, 80336 Munich, Germany; 11 Deutsches Zentrum für Neurodegenerative Erkrankungen (DZNE) Site Munich, Feodor-Lynen-Str. 17, 81377 Munich, Germany; 12 Munich Cluster for Systems Neurology (SyNergy), Adolf-Butenandt-Institut, Ludwig-Maximilians-Universität München, Feodor-Lynen-Str. 17, 81377 Munich, Germany; 13 Chair of Developmental Genetics, Technische Universität München-Weihenstephan, C/O Helmholtz Zentrum München, Ingolstädter Landstr. 1, 85764 Neuherberg, Germany; 14 Institute of Lung Biology and Disease, Helmholtz Zentrum München, German Research Center for Environmental Health, Ingolstädter Landstrasse 1, 85764 Neuherberg, Germany; 15 German Center for Lung Research, Marburg, Germany; 16 Luxembourg Centre for Systems Biomedicine (LCSB), University of Luxembourg, Luxembourg, Luxembourg; 17 University of Veterinary Medicine Hannover, Hanover, Germany; 18 University of Tennessee Health Science Center, Memphis, TN USA; 19 Present Address: Clinic of Neurodegenerative Diseases and Gerontopsychiatry, University of Bonn Medical Center, Bonn, Germany

## Abstract

The collaborative cross (CC) is a large panel of mouse-inbred lines derived from eight founder strains (NOD/ShiLtJ, NZO/HILtJ, A/J, C57BL/6J, 129S1/SvImJ, CAST/EiJ, PWK/PhJ, and WSB/EiJ). Here, we performed a comprehensive and comparative phenotyping screening to identify phenotypic differences and similarities between the eight founder strains. In total, more than 300 parameters including allergy, behavior, cardiovascular, clinical blood chemistry, dysmorphology, bone and cartilage, energy metabolism, eye and vision, immunology, lung function, neurology, nociception, and pathology were analyzed; in most traits from sixteen females and sixteen males. We identified over 270 parameters that were significantly different between strains. This study highlights the value of the founder and CC strains for phenotype-genotype associations of many genetic traits that are highly relevant to human diseases. All data described here are publicly available from the mouse phenome database for analyses and downloads.

**Electronic supplementary material:**

The online version of this article (10.1007/s00335-020-09827-3) contains supplementary material, which is available to authorized users.

## Introduction

The mouse is the most extensively used mammalian model for biomedical research. Mouse genetic reference populations (GRPs) have become an important experimental system to model the heterogeneity in the human population (Saul et al. 2019). Recently a new GRP, the Collaborative Cross (CC) was established (The Collaborative Cross Consortium 2012). In contrast to classical recombinant inbred strains that use two strains as progenitors, eight inbred strains were used as parental strains. The eight founder strains represent the three major *Mus musculus* subspecies: *M. m. domesticus*, *M. m. musculus*, and *M. m. castaneus*. Five of the founder strains (A/J, C57BL/6J, 129S1/SvImJ, NOD/ShiLtJ, NZO/HILtJ) are common laboratory strains, and three are wild-derived inbred strains (CAST/EiJ, PWK/PhJ, and WSB/EiJ), each of which have different phenotypic characteristics. By combining eight founder strains, the genetic and phenotypic diversity is similar to that of the human population. As the genomic sequences of the eight founder strains are available (Keane et al. 2011; Lilue et al. 2018), the CC represents an unprecedented and unique resource for genetic mapping and correlation studies (Roberts et al. 2007). Detailed information about characteristics of the single founder strains can be found in the Jackson laboratory (JAX) mice database (https://www.jax.org/jax-mice-and-services).

In addition, there are numerous phenotyping data available for several founder strains that have been deposited in the Mouse Phenome Database (MPD; https://phenome.jax.org; RRID:SCR_003212, (Bogue et al. 2018; Bogue et al. 2019; Grubb et al. 2014). For example, a high-throughput phenotyping protocol was used to measure the body composition and blood components of 43 inbred mouse strains including the CC founder strains after a high fat diet (Svenson et al. 2007). Furthermore, phenotype data are available for several focus areas e.g., immunology (Phillippi et al. 2014), morphology (Percival et al. 2016), behavior (Logan et al. 2013) and intestinal microbiota (Campbell et al. 2012). Also, founder strains were subjected to multiple challenges such as susceptibility to quantum dot (Scoville et al. 2015), response to microbiological (Smith et al. 2016) and viral (Ferris et al. 2013; Leist et al. 2016) infections. In this context, investigations of the pre-CC strains were often carried out as well (Ferris et al. 2013; Gralinski et al. 2015; Kelada 2016; Kelada et al. 2012; Phillippi et al. 2014; Rutledge et al. 2014). However, a comprehensive and comparative phenotyping analysis that comprises many parameters in a single study has not yet been performed. Extensive phenotyping data from the parental strains will allow assessing which parental alleles may contribute to which part of the phenotypic spectrum and thus help to better interpret QTL studies (e.g., Ferris et al. 2013; Graham et al. 2015; Gralinski et al. 2013; Phillippi et al. 2014; Vered et al. 2014; Zhang et al. 2018).

Therefore, we carried out a large-scale phenotyping study at the German Mouse Clinic (GMC, www.mouseclinic.de) (Fuchs et al. 2011, 2009, 2012; Gailus-Durner et al. 2005). The GMC is one of 19 worldwide research institutions in the International Mouse Phenotyping Consortium [IMPC, (Dickinson et al. 2016)] to produce and phenotype mouse strains with the aim of characterizing a knockout mouse line for every protein-coding gene.

Here, the founder strains were examined in a phenotyping pipeline that comprises standardized procedures in the areas of behavior, bone and cartilage development, neurology, clinical chemistry, hematology, eye development, immunology, allergy, energy metabolism, lung function, vision and pain perception, cardiology and pathology. Sixteen animals per sex and strain were examined in several batches to generate a statistically robust data set with the founder strains showing a high range of phenotypic differences. Our results confirmed already known strain characteristics and identified new phenotypes. Primary data and first line visualization and analyses are publicly available in MPD and can be downloaded for further analyses.

## Results

### Outline of phenotyping strategy

**Fig. 1:**
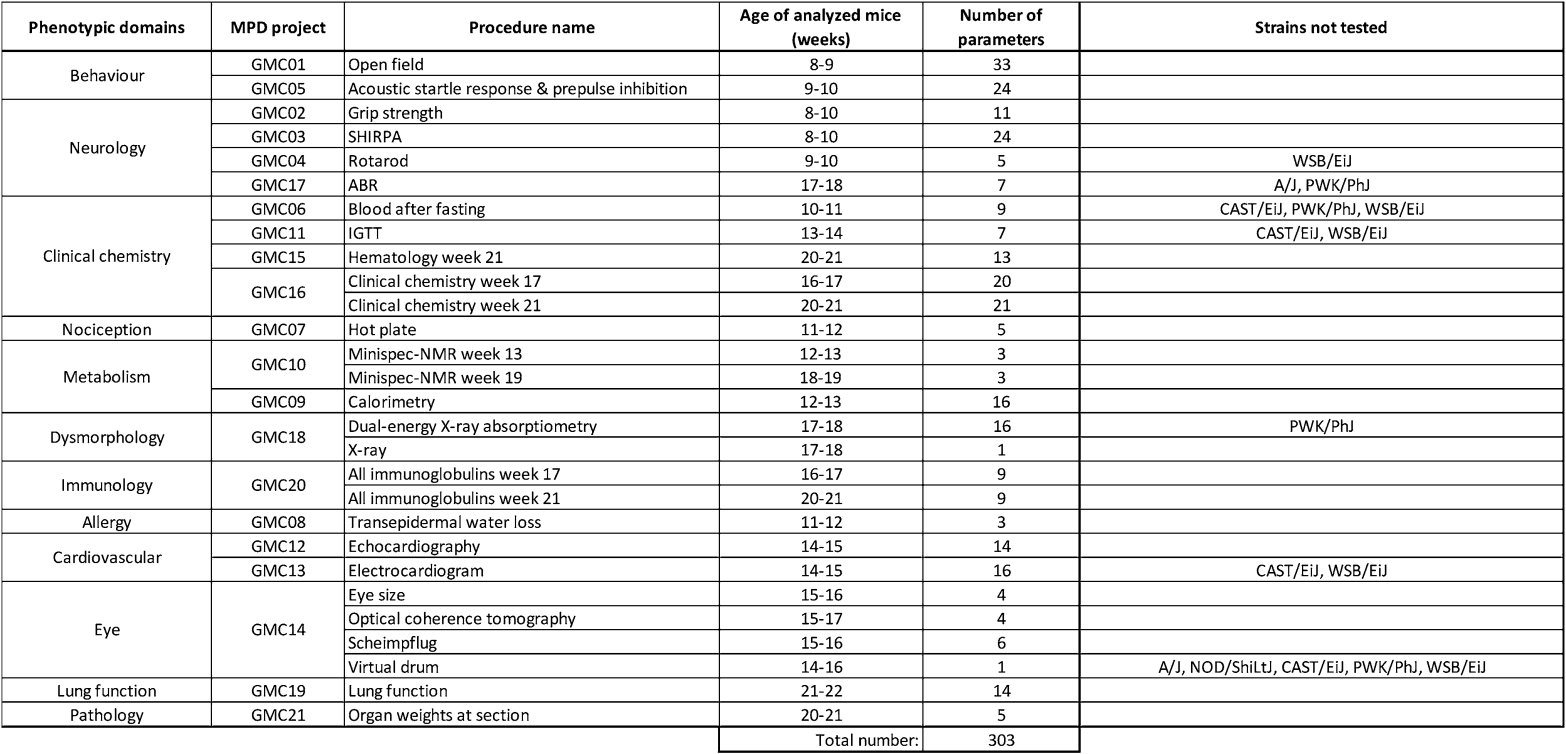
Overview of MPD projects, phenotypic domains and parameters measured

We performed a comprehensive standardized phenotyping pipeline at the German Mouse Clinic (GMC) for all eight CC founder strains (A/J, C57BL/6J, 129S1/SvImJ, NOD/ShiLtJ, NZO/HILtJ, CAST/EiJ, PWK/PhJ, and WSB/EiJ) covering all clinically relevant physiological systems (Figs. 1, S1). Mice were bred by synchronized mating at the Helmholtz Centre for Infection Research with sixteen or more animals per sex and strain analyzed in a timeframe of one year (January 2013 to April 2014).

Furthermore, we used five cohorts in total, supplied every three months, and distributed mice from a given strain and sex over these cohorts to ensure the phenotypic characterization covered all seasons. Cohorts of age-matched (7 weeks old, plus/minus 7 days) mice from each strain were shipped to the animal facility of the Helmholtz Zentrum München. After 2 weeks of acclimatization, mice were subjected to a phenotyping pipeline that encompassed 303 parameters in many phenotypic domains: allergy, behavior, cardiovascular analysis, clinical chemistry, hematology, dysmorphology including bone and cartilage, energy metabolism, eye analysis and vision, immunology, lung function, neurology, nociception, and pathology. In general, the same mice were tested for different phenotypes. In cases where mice did not survive the standardized workflow of 13 weeks, additional mice were used to achieve the targeted total number of 16 female and male mice per strain. Figure 1 summarizes the order and age of analysis for each procedure in the phenotyping pipeline. Fig. S1 details the order and the weeks of each measurement. The order of tests within the GMC phenotyping pipeline is based on experience from large-scale phenotyping efforts including the IMPC and EUMODIC programs. The order of tests reflects the input of experts from global research institutes about optimal age for each test and to minimize carry-over effects between tests including having sufficient recovery time between tests, e.g., having glucose challenge be the only test in a week period (Karp et al. 2015).

Phenotypic analyses were carried out in the German Mouse Clinic (GMC) at the Helmholtz Zentrum München by using standardized examination protocols (Fuchs et al. 2011; Gailus-Durner et al. 2009, 2005) (https://www.mouseclinic.de). The entire data set as well as the experimental procedures is available at MPD (https://phenome.jax.org/). All derived data analyses described here (*e.g.* global analyses, regression analyses) were performed with a data download from MPD dated 28.8.2018. The downloaded dataset (Data_dwnld_F1_20180828) has been deposited at the public repository RADAR (see chapter Data availability) for reproducibility of the results described here. Please note that the current and future datasets at MPD might slightly diverge from a RADAR submission if errors have been corrected after that date or if data formats changed.

### Deviations from the standard GMC phenotyping pipeline

In some cases, the GMC standard pipeline had to be modified to adapt to the special characteristics of some founder strains: For example, only a final bleeding was performed at week 21. Originally, blood sampling was also planned at week 17, but the wild-derived strains, WSB/EiJ, CAST/EiJ and PWK/PhJ, were too small to collect blood at this time. In addition, wild-derived strains were highly sensitive to the ketamine/xylazine injection anesthesia. Therefore, data for eye screen, bone density analysis and ABR were not obtained for all strains. Since the wild-derived strains were small and light, it was not possible to perform the Glucose Tolerance Test (GTT) because fasting over night was not possible for these strains. NZO/HILtJ mice were too heavy to measure body mass and body composition by qNMR due to size limitation of the machine. Awake electrocardiogram and visual acuity measurements had to be omitted for WSB/EiJ and CAST/EiJ since those strains were too active. The visual acuity measurement was not possible for NOD/ShiLtJ and A/J since these strains have an albino background. There are no rotarod data for WSB/EiJ since these mice were too active to stay on the apparatus. These limitations resulted in a reduction of the total number of mice per sex and strain for some parameters (Fig. 3 lists the total number of assays per GMC project per strain: significant parameters/total number of parameters measured per strain). However, most parameters were still measured with 16 animals per group. The exact number of mice for each parameter measurement can be found at MPD.

Besides these limitations, our phenotyping data represent the largest most comprehensive data set for the CC founder mice that will be highly valuable for the scientific community providing a baseline for studies with CC strains and the Diversity Outbred (DO) resource as well as facilitating the identification of new models for human diseases.

### Detailed phenotype data and first line analysis are publicly available in MPD

The phenotype data were deposited at MPD after extensive data quality control. In this step, extreme outliers caused by failures of machines or human error were excluded. In total, 303 parameters were uploaded to MPD. The data set is organized in 21 projects, each with a unique ID (GMC01 to GMC21) based on phenotypic domains. A procedure name describes a specific set of parameters, or measurements, in a given protocol (Fig. 1). The project protocol is attached in MPD and provides information about workflow, sampling, equipment, supplies, reagents, solutions and the type of data collected for each procedure. Each project has one accessioned data set, which is available for download (https://phenome.jax.org/). The downloadable tables contain the day of birth (DOB), date of test (DOT) and the values for each measured parameter including measurement units for each individual mouse and covariates like sex, body weight, etc.

For each measurement in MPD, a plot and overall summary table are provided as well as a table of strain means (unadjusted and Least Squares Means), standard deviation (SD), standard error of mean (SEM), number of mice, coefficient of variation, and Z-scores. The means tables are searchable and sortable so that strains with special characteristics may be quickly identified for each measurement. Individual animal data are available for online viewing or downloading. Analysis of variance (ANOVA) results are also made available such that sex, strain, and sex:strain interaction are analyzed. There is also a Q–Q normality assessment plot where theoretical quantile values are plotted against observed quantile values. Finally, for each eligible measurement, there is a possibility to run a genome-wide association analysis. Repeated measurements are plotted together so that trends in the data may be visualized. Each individual measurement of the repeated measurement series has the functionality just described. Some examples from the MPD data sets are shown and discussed below.

### Global analysis of phenotype data reveals many significant differences between strains and sex

We performed two types of global analyses to determine statistically significant differences at the level of parameters. First, in MPD, an ANOVA was performed as part of the data upload and subsequent presentation in MPD (as detailed above) that describes statistically significant differences between groups, including covariates. Second, we performed a pairwise comparison, based on the IMPC statistical pipeline contrasting parameter measurements for each strain with C57BL/6J as reference (Kurbatova et al. 2015). Both approaches are highly complementary. ANOVA provides a first level of statistical information for group differences and relates to the data graphs shown in MPD. On the other hand, the IMPC statistical pipeline was especially developed to analyze large-scale data from mouse phenotyping pipelines and allows adjusting for several confounding factors. It reports the results from pairwise comparisons to the reference strain C57BL/6J.

**Fig. 2:**
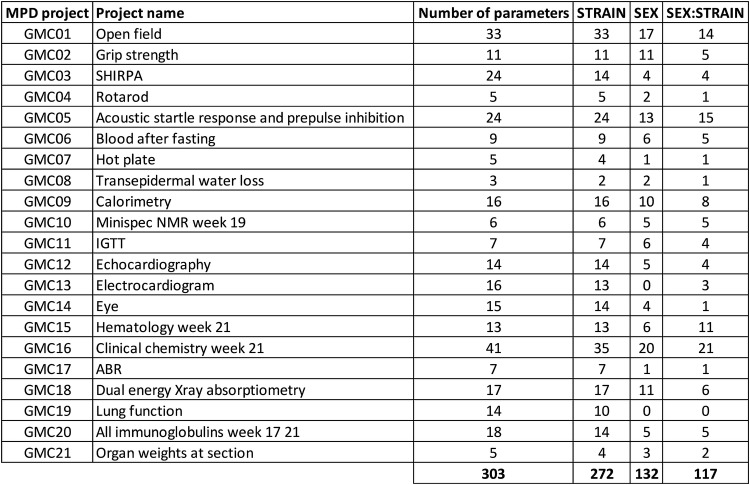
Number of significant parameters per project by strain and sex after ANOVA. ANOVA results for all parameters were extracted from MPD (freeze from 28th August 2018) and summarized. *p* values for individual parameters were adjusted for multiple testing using BH correction separately for the fixed variables strain, sex and sex:strain interaction. The figure illustrates the number of significant (*p* < 0.05) parameter measurements for each MPD project for the indicated explanatory variables

For the ANOVA MPD analysis, 272 parameters were significantly different by strain and 132 parameters by sex; ANOVA of sex by strain interaction revealed 117 significantly different parameters (Fig. 2, detailed results are presented in Tables S1, S2 and S3). Most different parameters between strains were found in the phenotypic domains ‘clinical chemistry’, ‘open field’, ‘acoustic startle response, and ‘prepulse inhibition’. A simple reason for this observation may be that these domains contained the largest number of parameters measured (compare to Fig. 2).

**Fig. 3:**
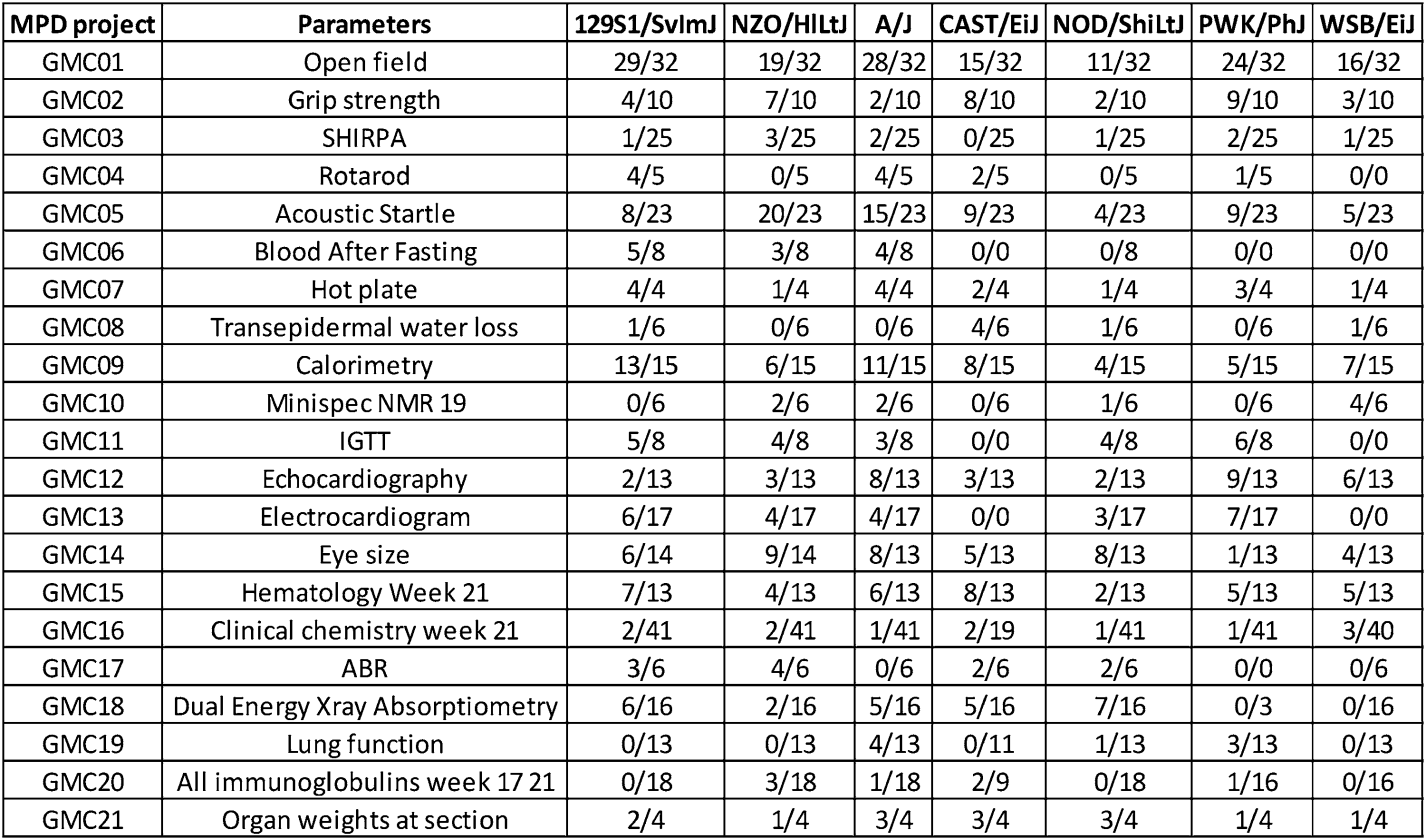
Number of significant parameters after pairwise comparisons of individual strains to C57BL/6J. Overview of the pairwise comparison of each founder strain to C57BL/6J as the reference. *p* values obtained from each individual comparison of parameters were adjusted for multiple testing using BH correction. The Figure summarizes the number of significant (*p* < 0.05) parameter measurements per MPD project from each pairwise comparison of the indicated strain to C57BL/6J per total number of assays performed

For the pairwise comparison based on the IMPC statistical pipeline, a large number of strain-specific significant differences to the C57BL/6J reference can be found (Fig. 3) with A/J showing the largest number of significantly different parameters and WSB/EiJ the least. However, it should be noted that these numbers are somewhat skewed by missing measurements in the wild-derived strains (Fig. 3; significant parameters/total number of parameters measured per strain).

**Fig. 4:**
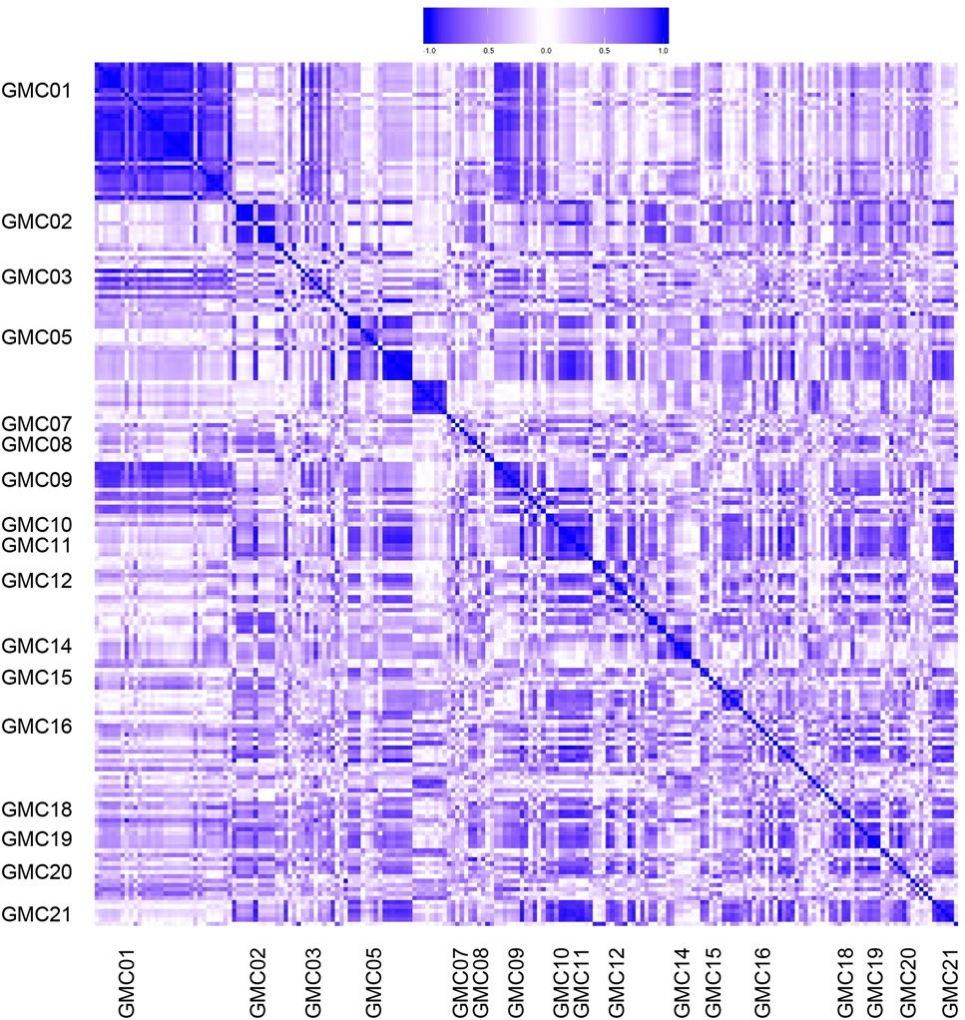
Correlation map of MPD projects. Pearson correlations between numerical values from all procedure measurements were calculated and then represented as heat map. A high resolution heat map with labels for all procedures can be found in the supplemental material (Fig. S3)

A correlation heat map (Fig. 4, detailed results are shown in Table S4 and a high resolution figure is shown in Fig S3) for all parameter measurements showed that the largest number of procedures were measured for GMC01 (behavior, open field) and that all results from this trait were highly correlated. In addition, several correlations existed between various MPD projects, e.g., GMC01 (behavior, open field) and GMC09 (metabolism, calorimetry) suggesting that activity in mice was correlated with energy metabolism. Also, GMC21 (organ weights) was correlated with several other GMC projects, notably GMC10 (metabolism, NMR), GMC11 (clinical chemistry, IGTT) indicating correlations between metabolic and clinical chemistry traits.

**Fig. 5:**
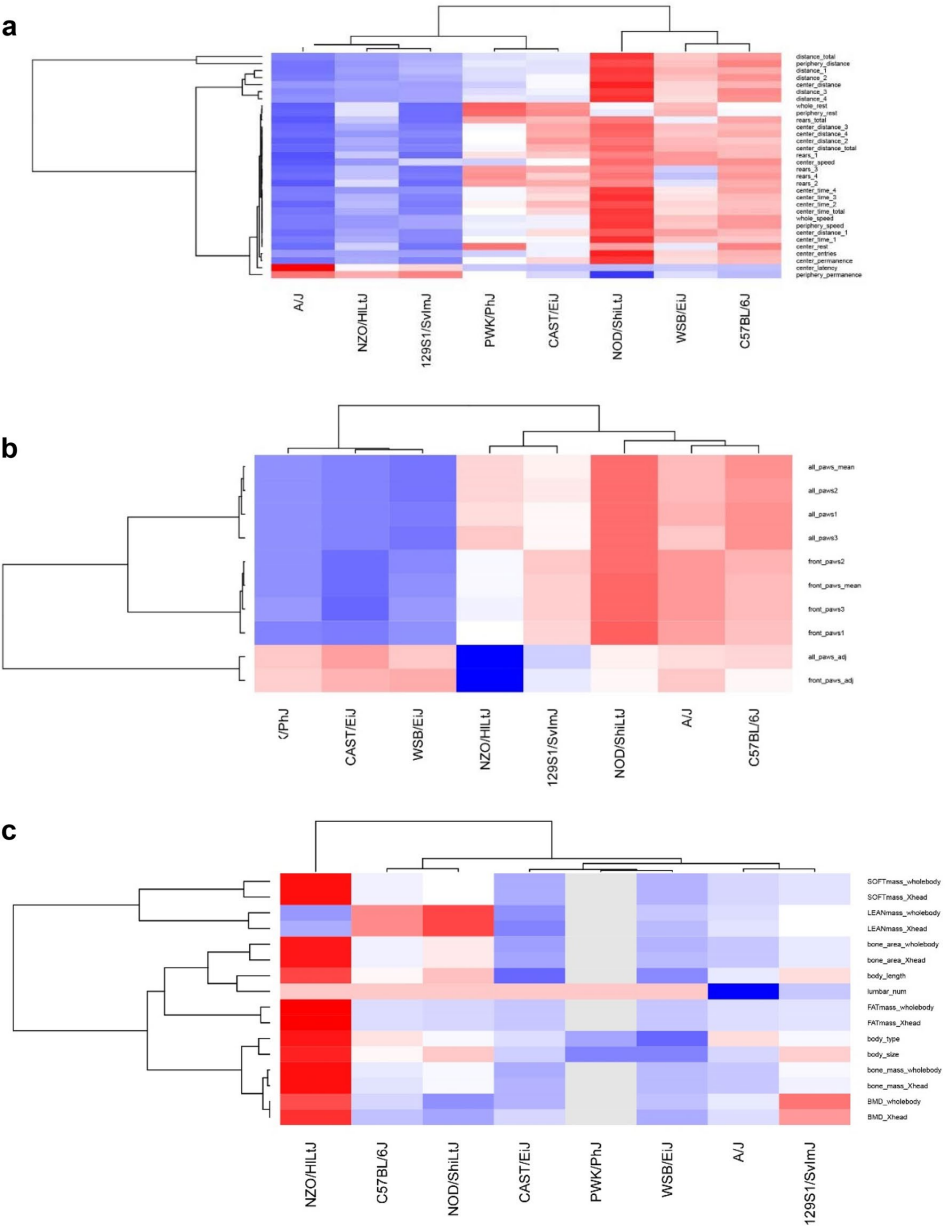
Heat maps of measurements for individual GMC projects. Heat maps of the means per strain for a given GMC project are shown. **a** GMC01, **b** GMC02, **c** GMC18. Values were scaled by rows (parameter measurements). Blue: low values, red: high values

The CC parental strains consist of three laboratory strains (A/J, C57BL/6J, 129S1/SvImJ), two disease models (NOD/ShiLtJ, NZO/HILtJ) and three wild-derived (CAST/EiJ, PWK/PhJ, and WSB/EiJ) strains. We performed an ANOVA to identify significant differences between these groups (Table S5). 144 parameter measurements were significantly different (*p* < 0.05) between the laboratory and wild-derived strains (top three most abundant procedures: Open_field: 32; Acoustic_Startle: 18; Dual_Energy_Xray_Absorptiometry: 13) and 163 parameters were significantly different (*p* < 0.05) between the laboratory strains and the disease models (top three most abundant procedures: Open_field: 25; SHIRPA: 13; Eye_size: 11). To illustrate further the differences between strains for any given parameter, we generated heat maps for each project using the means for each measurement per strain (examples are shown in Fig. 5 and all results are presented in Fig. S2). In general, there was no consistent pattern over all strains, e.g., that wild-derived strains would always differ from the other strains. Groupings were specific for each project. For example, for GMC01 (Open field; Fig. 5a), the difference in behavior (high resting time in center) between the diabetic strain NOD/ShiLtJ, the obese strain NZO/HILtJ (low resting time in center) and all others was quite obvious. Low grip strength values (all_paws_adj) for NOD/ShiLtJ in GMC02 was one of the strongest differences to all other strains (Fig. 5b) whereas it was high for all wild-derived trains. All wild-derived strains were low for GMC18 (Xray absorptiometry, Fig. 5c) whereas NZO/HILtJ was high, indicating a difference in body composition between wild-derived and laboratory strains and an outsider position for NZO/HILtJ.

Mice were shipped in five cohorts (batches). We thus performed an ANOVA for all measured parameters and batch as explanatory variable (model: parameter ~ batch) to evaluate possible batch effects. The results are listed in Table S6; 80 measurements showed significant batch effects (*p* < 0.01). Thus, batch was included in the model for the pairwise comparisons, and we corrected for this effect for the above analyses.

Ethics committees frequently request a power analysis to determine group sizes for approval of animal experimental protocols. However, this is often impossible because no data exist or are not available as raw data from publications. Our extensive data set on the phenotypes from the CC founder will allow performing power calculations for many phenotypic traits because the raw data are readily accessible at MPD, group sizes in our settings are large enough to perform a power analysis, the appropriate comparison and delta of means can be selected. As an example, we performed a power analyses for three significant phenotype measurements based on pairwise comparisons from projects GMC01 and GMC16. Group sizes for power were calculated using the following settings: power = 0.8, significance level = 0.05, standard deviation (sd) = mean of all sd values for the selected parameter over all strains, delta = difference in means between strains. The results are listed in Table S7. It becomes evident that group sizes are highly variable depending on which strains to compare and the selected difference in means. Thus, our data should provide a valuable resource to decide for the appropriate comparisons and group sizes.

### Examples of phenotypes from the pipeline analyses

Below, we describe some selected projects/phenotypes in more detail to give the reader some insights into the type of results that we obtained, as well as differences that can be observed between groups, and the discovery of novel phenotypes and the data visualizations and analysis tools that are provided in MPD.

### Spontaneous locomotor activity

**Fig. 6:**
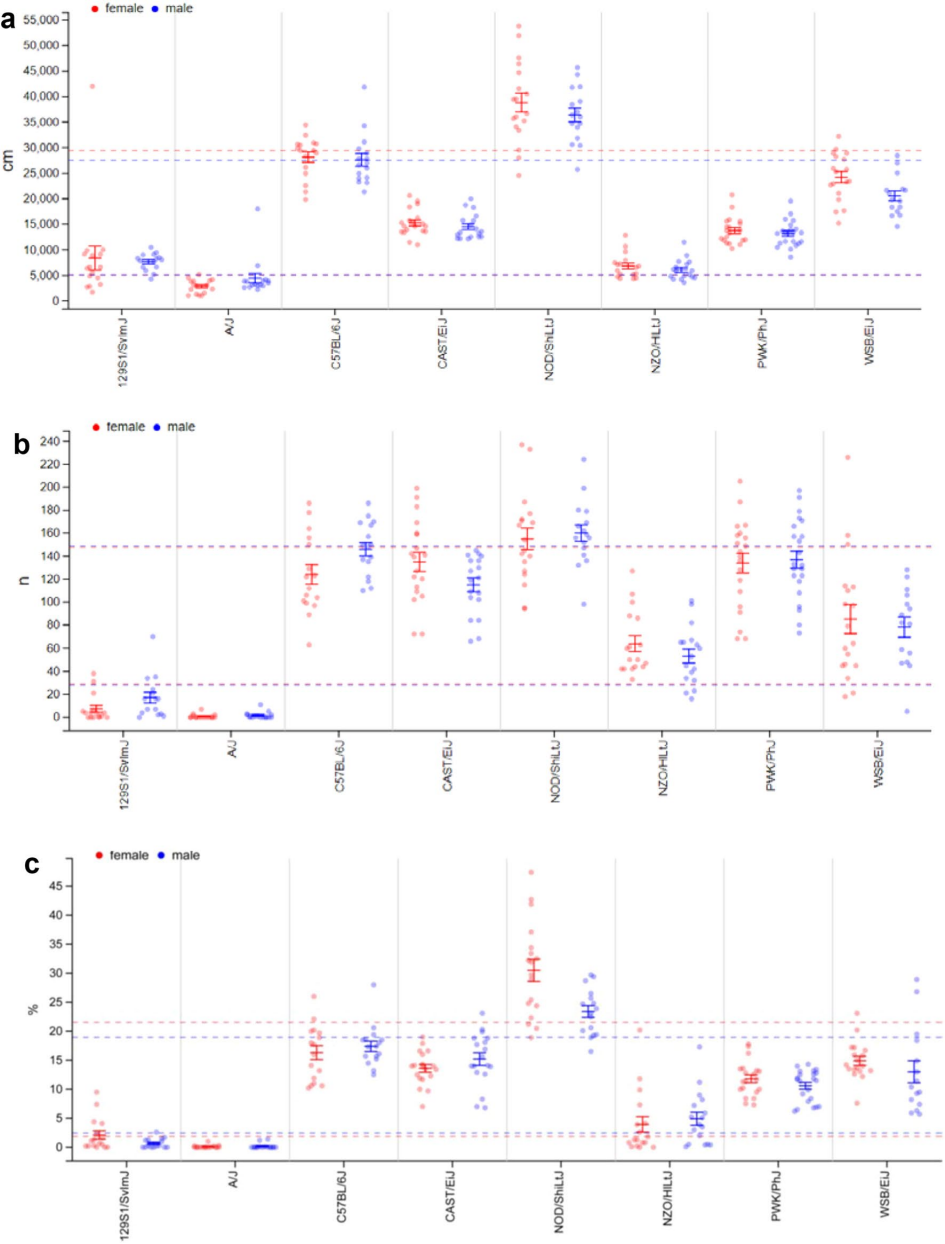
MPD view of project GMC01. **a** GMC01—distance traveled total, 20 min test; **b** GMC01—number of rears total, 20 min test; **c** GMC01—resting time in center of arena, 20 min test. Each dot represents the value for a single mouse, males in blue, females in red. Solid vertical bars show the means and standard error of mean (SEM) for each strain (red for females, blue for males). Stippled lines indicate the overall mean per sex and standard deviation (SD). x-axis: strain names; y-axis **a** distance traveled in cm (cm); y-axis **b** total number of rears (n); y-axis **c** percent of total time spent in center (%); The detailed protocols can be found in MPD

Open Field tests are widely used as assay to measure spontaneous locomotor and exploratory activity as well as anxiety in a novel environment. Figure 6 illustrates the data representation that can be found in MPD for the parameter measurements from this project (GMC01): ‘distance traveled total, 20 min test’ (Fig. 6a), ‘rearing activity, number of rears total, 20 min test’ (Fig. 6b), and ‘percentage of total time spent in the center, 20 min test’ (Fig. 6c). We found that spontaneous locomotor activity measured in the Open Field test was highest in NOD/ShiLtJ, followed by C57BL/6J, and lowest in A/J and NZO/HILtJ mice. The other strains were intermediate; from most to least active: WSB/EiJ, CAST/EiJ, PWK/PhJ and 129S1/SvImJ (Fig. 6a). With respect to rearing activity in the open field (Fig. 6b) A/J and 129S1/SvImJ were engaging the least while NOD/ShiLtJ, PWK/PhJ, CAST/EiJ and C57BL/6J mice were rearing at comparable frequencies. With respect to anxiety-related behavior measured by center time (%) in the central, aversive zone of the Open Field (Fig. 6c), A/J and 129S1/SvImJ mice spent the least time in the center, while NOD/ShiLtJ spent the most time in the center.

### Acoustic startle reactivity and its prepulse inhibition

**Fig. 7:**
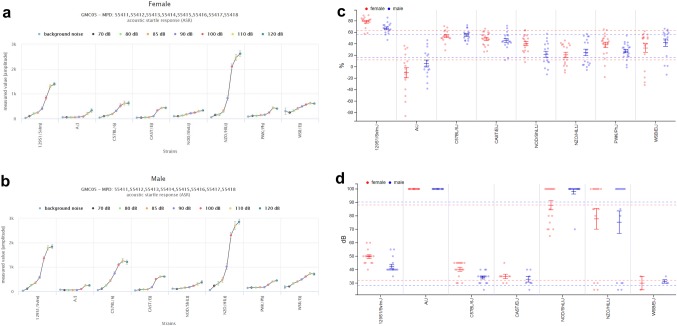
MPD view of project GMC05. GMC05—acoustic startle response MPD:55,411 ASR acoustic startle response (ASR)[amplitude] **a** females, **b** males; **c** GMC05—percentage prepulse inhibition (PPI) evoked by 110 dB sound pressure level with global prepulse stimulus; **d** GMC17—auditory brainstem response (ABR) threshold, sound pressure level, click stimulus. Note that PWK/PhJ was not measured for ABR due to anesthesia intolerance. Each dot represents the value for a single mouse, males in blue, females in red. Solid vertical bars show the means and standard error of mean (SEM) for each strain (red for females, blue for males). Stippled lines indicate the overall mean per sex and standard deviation (SD). x-axis: strain names; y-axis **a** amplitude measured (db, decibel); y-axis **b** amplitude measured (db); y-axis **c** percent (%); y-axis **d** decibel (db) The detailed protocols can be found in MPD

Prepulse inhibition of the acoustic startle reflex is a reliable measure of sensorimotor gating that is highly conserved across species. Dysfunctions in prepulse inhibition are prominent in several neurodevelopmental psychiatric disorders, e.g., schizophrenia amongst others. As shown in Figs. 7a and b, NZO/HILtJ mice demonstrated the highest startle response relative to all other strains (GMC05). NOD/ShiLtJ, WSB/EiJ, CAST/EiJ, PWK/PhJ and A/J mice showed comparably low responses, while C57BL/6J and 129S1/SvImJ mice had intermediate values. The highest prepulse inhibition responses were observed in 129S1/SvImJ, with C57BL/6J mice ranking the second highest, and A/J mice showing the lowest PPI levels (Fig. 7c). The other mouse strains had intermediate values and within a broadly similar range.

### Hearing sensitivity

Hearing loss is a common condition in humans that can be caused by many environmental and genetic factors. Here, we measured auditory brainstem response (ABR) to evaluate hearing sensitivity in the eight founder strains. ABR was performed by applying different sound stimuli (one broadband click and five pure tones) to anesthetized mice and determination of the critical sound pressure threshold needed for ABR response was determined. The differences in startle responses in the project GMC05 “acoustic startle reactivity and its pre-pulse inhibition” were reflected by the hearing sensitivity of the different strains: A/J and NOD/ShiLtJ showing low startle response seemed to be nearly insensitive for all ABR frequencies tested. However, some of the NZO/HILtJ mice were nearly deaf, while others were still within physiological ranges when measuring auditory brainstem response to a click stimulus (Fig. 7d).

### Grip strength

**Fig. 8:**
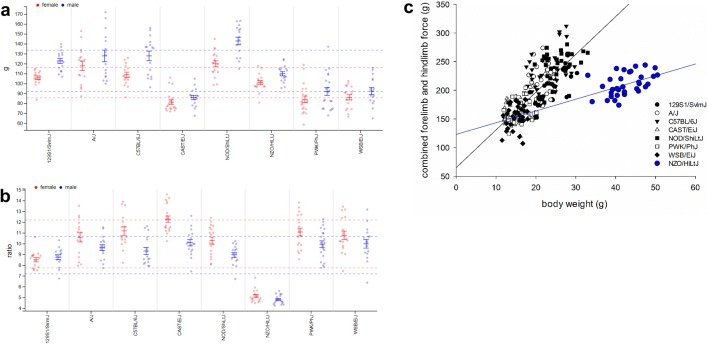
MPD view of project GMC02. **a** GMC02—forelimb grip strength, mean; **b** GMC02—forelimb and hindlimb grip strength (mean) divided by body weight; **c** Correlation of mean grip strength of all paws pooled for both sexes to body weight (black line for all strains except NZO/HILtJ), NZO/HILtJ: blue symbols and line. Each dot represents the value for a single mouse, males in blue, females in red. Solid vertical bars show the means and standard error of mean (SEM) for each strain (red for females, blue for males). Stippled lines indicate the overall mean per sex and standard deviation (SD). x-axis in a and b: strain names; y-axis **a** grip strength force meter in gram (g); y-axis **b** ratio of grip strength to body weight (ratio); x-axis in **c** body weight in gram (g); y-axis **c** grip strength force meter in gram (g); The detailed protocols can be found in MPD

Grip strength maybe affected by muscle function itself, the neuromuscular control of the muscle as well as by energy metabolism parameters. To assess muscle function, grip strength was measured (GMC02). Grip strength was highest in NOD/ShiLtJ and lowest in CAST/EiJ mice (Fig. 8a). On the other hand, NOD/ShiLtJ mice had also high body weight (after NZO/HILtJ) whereas CAST/EiJ had lowest body weight (ratio grip strength and body weight indicated in Fig. 8b). Including body weight as a covariate revealed a statistically significant correlation for grip strength and body weight, as shown in Fig. 8c. NZO/HILtJ clustered differently indicating that they were weak given their body weight (Fig. 8c) since these mice have a large amount of body fat and a lean mass more similar to NOD/ShiLtJ mice (data not shown). On the other hand, NZO/HILtJ mice develop obesity and glucose intolerance and therefore muscle function could be impaired directly as well. Often, mutant mouse lines show differences in body weight (Reed et al. 2008) and these differences might correlate with other parameters as well like bone mineral density or lean mass (Karp et al. 2012; Oellrich et al. 2016). Muscle strength had not been reported yet for NZO/HILtJ mice but it had been shown that endurance as well as activity was reduced in NZO/HILtJ mice (Courtney and Massett 2012).

### Neurological analysis

For basic neurobehavioral assessment and an overall visual inspection a SHIRPA [SmithKline Beecham, Harwell, Imperial College, Royal London Hospital, phenotype assessment (Rogers et al. 2001)] protocol was used in a modified form for rating observations for abnormalities of general appearance, movement and some reflexes (GMC03, data not shown). Differences were detected between several strains: less tail elevation in NZO/HILtJ and A/J, differences in pelvic elevation (less in NZO/HILtJ, PWK/PhJ, A/J and 129S1/SvImJ), less startle response in A/J (see hearing sensitivity results), less transfer arousal in A/J and NZO/HILtJ. Locomotor activity was reduced in NZO/HILtJ, A/J, 129S1/SvImJ and PWK/PhJ mice compared to C57BL/6J (A/J < NZO/HILtJ < 129S1/SvImJ < PWK/PhJ < WSB/EiJ < CAST/EiJ < C57BL/6J < NOD/ShiLtJ, data not shown).

### Eye analysis

The visual capability of a mouse may influence many other traits. Therefore, it is important to consider also eye morphology and visual capability. In order to identify differences in eye size, morphology of the anterior and posterior segment of the eye, as well as visual acuity between the strains, the animals were tested in the virtual drum after that examined with the Scheimpflug rotating camera, laser interference biometry (LIB) and optical coherence tomography (OCT; GMC14, data not shown). The eye screen identified subtle differences between the strains (data not shown). Specifically, Scheimpflug imaging for the anterior eye segment showed higher lens density for the A/J mice, followed by the NOD/ShiLtJ and 129S1/SvImJ animals whereas the WSB/EiJ, C57BL/6J, CAST/EiJ and NZO/HILtJ showed almost comparable lens density. The posterior part of the eyeball (fundus), was visualized by the means of OCT. OCT examination revealed normally developed retinal layers in all mouse strains. The number of the retinal main blood vessels was comparable between all strains. The strains with the highest retinal thickness were the C57BL/6J, A/J and 129S1/SvImJ, followed by the PWK/PhJ, NZO/HILtJ, CAST/EiJ and WSB/EiJ strains that have comparable retinal thicknesses. Analysis of the size of the ocular components by LIB, in the CC strains, indicated a variable strain dependent eye axial size, suggesting a significant role of genetic background in eye development. For example, the NOD/ShiLtJ mice had the longest eye axial length, followed by NZO/HILtJ mice. The other mouse strains presented comparative eye sizes: C57BL/6J > A/J > 129S1/SvImJ > WSB/EiJ > PWK/PhJ > CAST/EiJ. Visual acuity testing was not performed in all mouse strains e.g., due to hyperactivity observed for PWK/PhJ, CAST/EiJ, and WSB/EiJ. In addition, the NOD/ShiLtJ and A/J strains were not tested in the virtual drum because of the albino background, known to be characterized by reduced visual acuity due to melanin synthesis disorders that predisposes the visual system to abnormalities affecting the retina and the retinofugal projections. Of the drum-tested strains, C57BL/6J mice showed weaker visual acuity compared to the NZO/HILtJ and 129S1/SvImJ mice. 129S1/SvImJ mice were found to have a comparable visual acuity to C57BL/6J mice (Wong and Brown 2006).

### Clinical chemistry

**Fig. 9:**
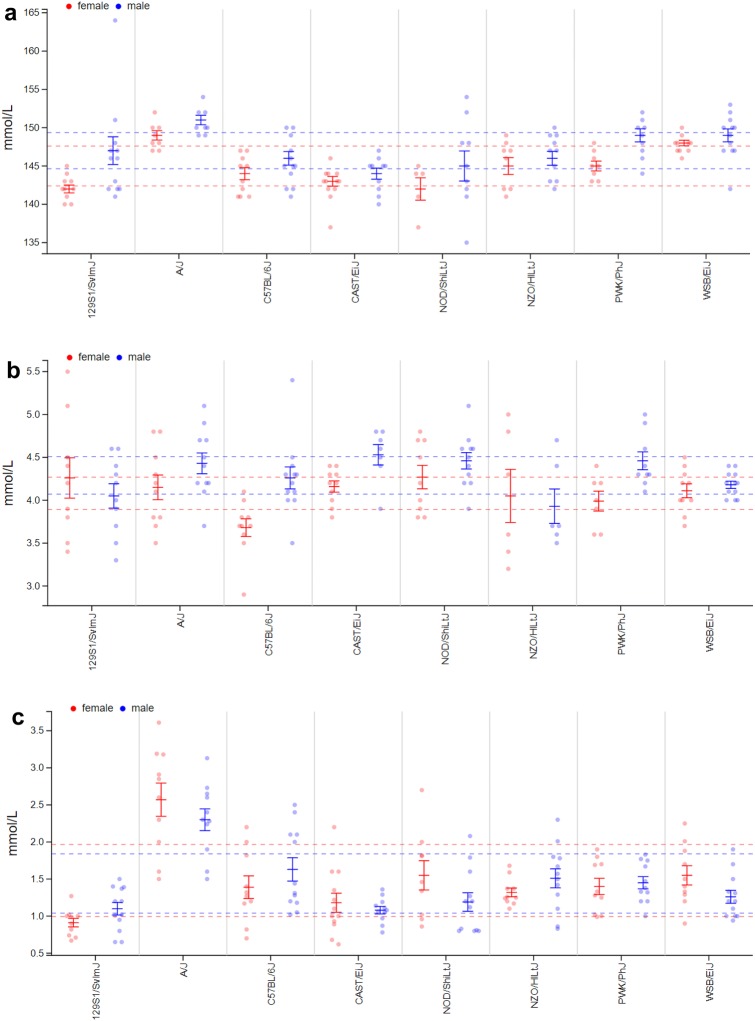
MPD view of project GMC16. **a** GMC16—sodium (plasma Na) at age 20–21 weeks; **b** GMC16—potassium (plasma K) at age 20–21 weeks; **c** GMC16—phosphorus (plasma phosphate) at age 20–21 weeks. Each dot represents the value for a single mouse, males in blue, females in red. Solid vertical bars show the means and standard error of mean (SEM) for each strain (red for females, blue for males). Stippled lines indicate the overall mean per sex and standard deviation (SD). x-axis: strain names; y-axis **a** plasma Na at age 20–21 weeks in mMol per liter (mmol/L); y-axis **b** plasma K at age 20–21 weeks in mMol per liter (mmol/L); y-axis **c** plasma phosphate at age 20–21 weeks in mMol per liter (mmol/L); The detailed protocols can be found in MPD

An assessment of blood chemistry parameters provides a good overview of the metabolic state, organ functions as well as electrolyte and mineral homeostasis. The studies can provide hints towards genetically determined disease susceptibilities. Plasma clinical chemistry had been measured for the Collaborative Cross founder strains before in two studies published in the Mouse Phenome Database (CGDpheno3, Chesler2). Our studies confirm many of the findings from these studies (GMC16). For example, we also found extremely high cholesterol levels in NZO/HILtJ, and compared to C57BL/6J elevated levels in 129S1/SvImJ and WSB/EiJ animals, while PWK/PhJ, A/J and CAST/EiJ showed lower levels. Similarly, already published strain-related differences in glucose, triglyceride, calcium and urea levels were mostly confirmed. However, the parameters presented in these two studies do not include phosphate values, enzyme activities or parameters related to iron metabolism. For these parameters comparative data from literature are rare. One of the most striking results was our findings concerning parameters of mineral metabolism in A/J mice. Compared to all other strains, A/J mice exhibited slightly elevated sodium levels and significant hyperphoshatemia, while no strong deviations from the other strains were observed for potassium levels (Fig. 9a, b, c). A/J mice also differed from other strains by showing an inverted sex difference for alkaline phosphatase activity in this strain with higher values in males than in females, while for all other strains there was no difference or inverse relationships.

### Glucose tolerance test

Diabetes is a common metabolic disorder in humans. Different types of monogenic or complex genesis can be differentiated. The glucose tolerance test is the standard mean to identify diabetes or pre-diabetic states in men and mice. In addition, subtle differences in the regulation of glucose metabolism, possibly affecting general metabolic state, the response to diet challenges and susceptibility to age-related diseases, can be detected by this test in mice. Intraperitoneal glucose tolerance tests were performed at the age of 13–14 weeks. The results confirmed several well-known strain-specific characteristics, such as impaired glucose tolerance in NZO/HILtJ and NOD/ShiLtJ mice (Chen et al. 2018; Kleinert et al. 2018) and references therein) (GMC11, data not shown). In contrast, 129S1/SvImJ mice showed very low basal fasting glucose levels and low AUC values, which is in line with the observation of low endogenous glucose production in fasting 129S1/SvImJ mice (Burgess et al. 2005). Due to low body mass in wild-derived strains, only male animals of the PWK/PhJ strain could be tested for glucose tolerance. These animals showed a similar phenotype as C57BL/6J mice. For NZO/HILtJ, lean and fat measurement data have been deposited previously in MPD (Multi-system survey of mouse physiology in 72 inbred strains of mice MPD:CGDpheno1; Mouse Phenome Database web resource; RRID:SCR_003212; https://phenome.jax.org), indicating a high body fat proportion. This is important to consider, since body composition besides other factors also affects the outcome of glucose tolerance tests (Jorgensen et al. 2017).

### Pathology screen and heart weight

**Fig. 10:**
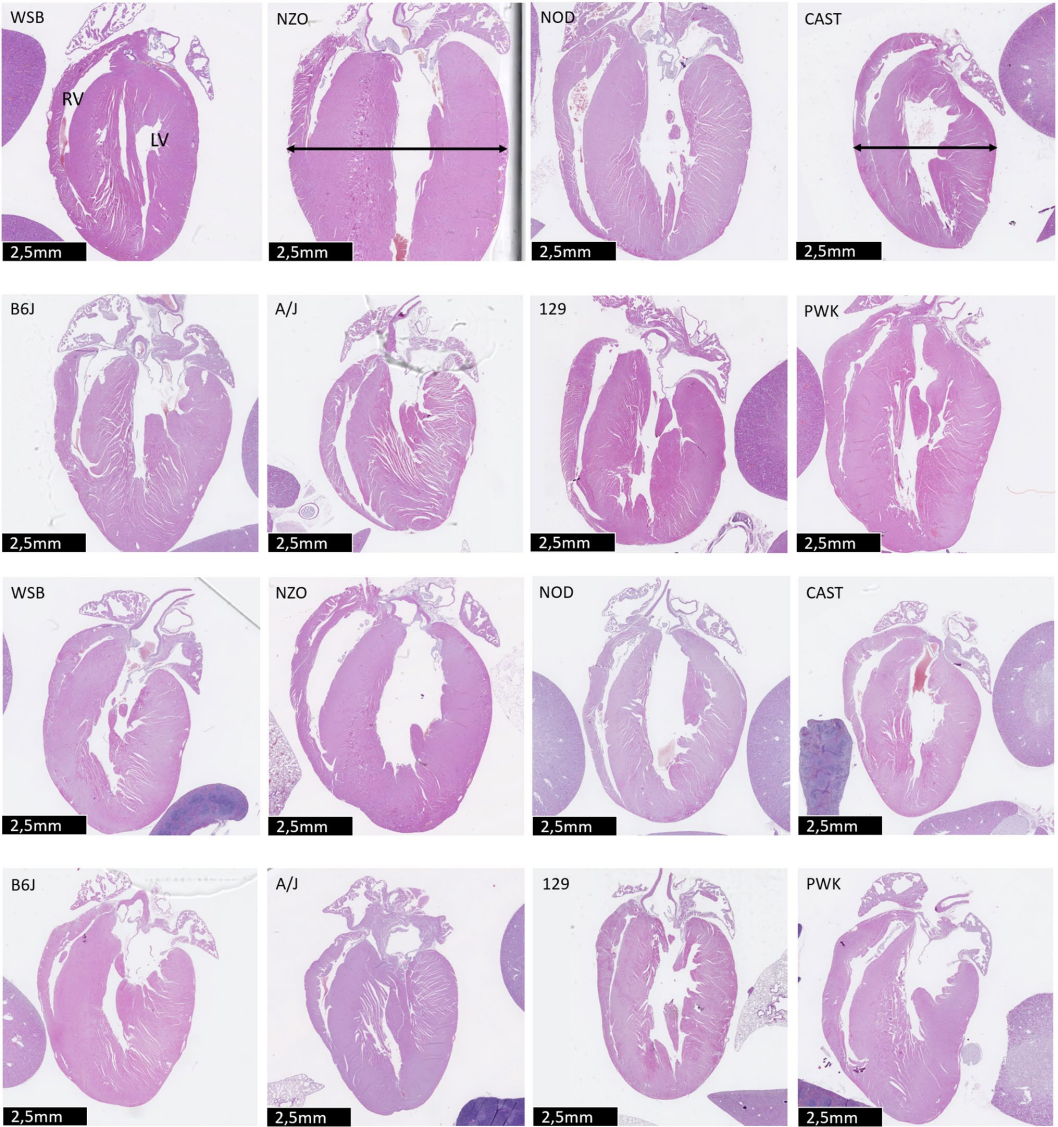
Histological sections of hearts from CC founder strains. Histological sections show the left (LV) and right (RV) ventricular free walls, the left and right auricles and part of the aorta root. The upper two rows display heart histology of male mice, the lower two rows histology of female mice. Note the differences in thickening of the LV myocardium between NZO/HILtJ (7 mm) and CAST/EiJ (4.5 mm) strains (black arrows)

Light microscopy histological analysis of 30 examined organs did not reveal new strain-specific changes. Hearts of female and male NZO/HILtJ mice appeared hypertrophic when compared to the hearts of the other mouse strains (Fig. 10) but NZO/HILtJ were also bigger and heavier. Therefore, understanding the influence of covariates was of special interest.

**Fig. 11:**
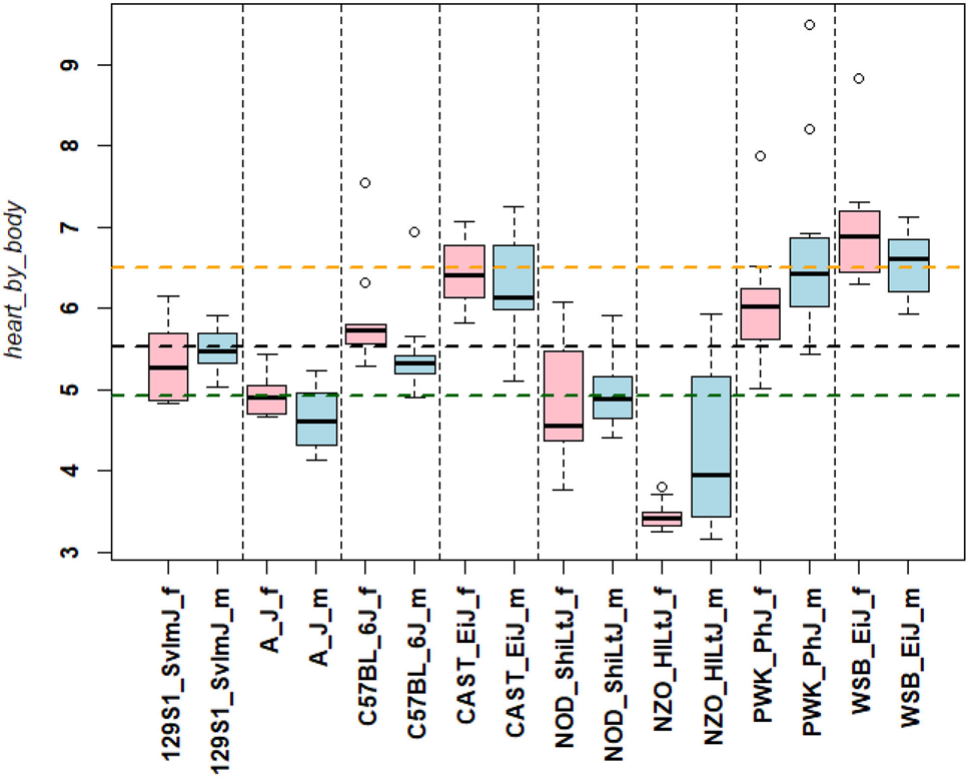
Correlation of heart to body weight for project GMC21. Data for GMC21 were downloaded from MPD (freeze from 28th August 2018). The histogram illustrates heart to body weight ratios for male (_m) and female (_f) mice from each strain. Box plot centerline: median, box plot limits: upper and lower quartiles, box plot whiskers: 1.5 × interquartile range

The analysis of heart weight represents an ideal example to illustrate how data from MPD can be further explored to obtain more insights into strain differences and the influence of covariates. Project GMC21 measured heart weight together with several covariates, sex, body weight and tibia length. Heart weight/tibia lengths ratio is a parameter often used for investigation of cardiac hypertrophy. The optimized ANOVA model (see M&M) showed that all three parameters (strain, sex and body weight) were significant (Table S8). Also, wild-derived strains were significantly different from the laboratory strains and each of the disease models was different from all other strains. These findings are illustrated in Fig. 11 where heart weight was corrected for body weight. The wild-derived strains exhibited significantly higher heart weights (p < 0.001) compared to the classical laboratory strains (Fig. 11). Also, the disease model strains NOD/ShiLtJ and NZO/HILtJ differed (individually) from all other strains (*p* < 0.001) with NZO/HILtJ females showing a decrease in heart weight/body ratio. In summary, this example demonstrates how raw data from MPD can be used by special interest groups for further analysis of strain differences and variables that control such differences.

## Discussion

Here, we present the first comprehensive phenotyping analysis of the CC founder strains using a standardized phenotyping pipeline. Three hundred and three (303) parameters were measured for all eight CC founder strains over a period of 15 months. We aimed for large group sizes (most parameters were measured for 16 animals for all strains and both sexes). This allowed us to obtain statistically robust results that include contrasting groups of strains, single strains to all others, effects of sex and other covariates.

All primary data can be accessed and downloaded from the public MPD database. It thus provides a highly valuable public resource for physiological, morphological and behavioral phenotype data of the CC founder and will serve as an important reference for baseline values to better understand phenotypes in the recombinant inbred CC strain collection (The Collaborative Cross Consortium 2012), the outbred Diversity Outcross resource (DO, (Churchill et al. 2012; Svenson et al. 2012), F1 mice generated from CC strains, and future resources that will be generated from the CC founder strains.

MPD also provides online visualization and a first line statistical analysis of the data allowing an easily accessible overview for each phenotypic measurement also for less experienced users or less sophisticated analyses. In addition, the raw data sets can be directly downloaded from the MPD database for further detailed analyses of individual traits, correlation studies between traits and groups or comparisons to other data sets.

Our study corroborates and extends phenotypic characteristics of the CC founder strains that were described before, like spontaneous locomotor activities, motor skills and grip strength. In addition, several differences between strains and sexes have not been reported before, like deafness of NZO/HILtJ mice and heart to body weight ratios between laboratory and wild-derived strains. Below, we discuss some of our findings without trying to be comprehensive but rather to demonstrate for a few examples the value of the data as resource for the scientific community.

The largest set of phenotypes was obtained for behavioral and neurological parameters. In our spontaneous locomotor activity analysis, the NOD/ShiLtJ was the most active, and A/J and NZO/HILtJ were the least active. These results are generally consistent with previously published open field analyses of the CC founder strains (Amrani et al. 1994; Lad et al. 2010; Logan et al. 2013; Takahashi et al. 2006), and a similar pattern of A/J < 129S1/SvImJ < C57BL/6J in terms of locomotor activity has also been observed elsewhere (Bohlen et al. 2014; Mandillo et al. 2008). Similarly, the strain ranking of anxiety-related behavior is in line with previous reports (Lad et al. 2010), e.g., it was shown that, and also consistently across different testing centers, C57BL/6J mice spend more time in the center of the open field than 129S1/SvImJ mice (Kulesskaya and Voikar 2014; Mandillo et al. 2008).

Differences in startle responses may be due to differences in hearing sensitivity, neuromuscular recruitment or anxiety-related behavior. In NZO/HILtJ mice, however, the measurement of ABR showed that some of the NZO/HILtJ mice were nearly deaf while others were still hearing well. The high startle response was possibly confounded by their higher body weight, since startle amplitudes reflect alterations in the weight placed on the highly sensitive measuring platform, and are thus influenced by body weight. While the body weight normalized analysis of this response brought the levels of NZO/HILtJ mice closer to the other lines, they still did not exhibit the low startle measurement that would be characteristic of a hearing impairment. This suggests that in this line, hearing loss manifested during the 7 weeks between ASR and ABR measurement, potentially enhanced by the noise exposure during the ASR test. Several inbred mouse strains exhibited a progressive, non-syndromic hearing loss with variable onset (Zheng et al. 1999), but a general hearing impairment has not been described before in NZO/HILt mice. Interestingly, these mice carry a mutation inactivating PCTP (Phosphatidylcholine transfer protein) (Pan et al. 2006) which is downregulated in auditory glia cells in response to loud noise exposure (Panganiban et al. 2018). Recent literature suggests a link for diabetes and hearing loss in men and mice (Akinpelu et al. 2014; Hong and Kang 2014; Horikawa et al. 2013) but we did not test for a correlation of pre-diabetic stages and hearing loss of NZO/HILtJ mice. The NOD/ShiLtJ and A/J mouse strains were deemed to be deaf as tested by ABR as described before for A/J and another sub-strain of NOD (NOD/LtJ (Johnson et al. 2006). Thus, the pattern of lower ASR in these mice was consistent with this finding. The WSB/EiJ, PWK/PhJ and CAST/EiJ mice also showed relatively low ASR. The pattern of higher ASR in the 129S1/SvImJ strain relative to the C57BL/6J strain (that undergoes progressive sensorineural hearing loss) has been described before (Johnson et al. 2006; Mandillo et al. 2008; Zheng et al. 1999). Hearing sensitivity was high for the wild-derived strains PWK/PhJ, WSB/EiJ and CAST/EiJ thus confirming current literature (Johnson et al. 2006).

Motor skills as evident from the rotarod performance were quite similar, but lowest in A/J mice and 129S1/SvImJ mice. This was also described before by Bohlen et al. (Bohlen et al. 2014). WSB/EiJ mice could not be measured at the accelerating rotarod since they were not cooperative and jumped immediately from the rod.

Plasma clinical chemistry had been measured for the Collaborative Cross founder strains before in two studies published on the Mouse Phenome Database (CGDpheno3, Chesler2), and our data confirm many of the findings of these studies, e.g., elevated cholesterol levels in NZO/HILtJ and 129S1/SvImJ mice in contrast to CAST/EiJ mice with low values (O'Connor et al. 2014). The same was true for several other parameters measured. For example glucose, triglycerides, creatinine, urea and electrolytes, that were also included in one of the studies published on MPD. There are only few previous publications comparing a broad range of clinical chemistry parameters for mouse-inbred strains, for example (Champy et al. 2008) comparing C57BL/6J, C3HeB/FeJ, BALB/cByJ and 129/SvPas mice. These studies included only a fraction of the strains tested in our study, and often even not the same sub-strains, which makes the comparison of results difficult. However, the mentioned study still reports similar results concerning the differences between C57BL/6J and the 129/SvPas strain as we found in our study. For example, it also described lower plasma phosphorus values than seen in C57BL/6J for the 129 strain. A/J mice exhibited slightly elevated sodium levels and significant hyperphoshatemia. There are no reports in the literature for plasma phosphorus levels in A/J mice. Another study in MPD (Yuan3: Aging study: Blood chemistry for 32 inbred strains of mice) compared 28 inbred strains including A/J for clinical blood chemistry at 6, 12 and 18 months of age. It is the only study in the MPD database including values of serum phosphorus measurements, and shows comparably high values for 6 months old male A/J mice, but not for females. Therefore, this study is the first one showing such a clear shift in plasma phosphate levels for A/J mice. The observation that plasma mineral levels in A/J mice differ from those measured in C57BL/6J animals, is interesting in the context of a recent study showing remarkable differences in bone structure between these strains (Mathis et al., 2019), since both observations point towards strong strain-related effects on the regulation of mineral and bone metabolism.

In summary, with our studies we provide a highly valuable public resource for the scientific community working with genetic reference populations that are derived from the Collaborative Cross founder mice such as CC strains, F1 and F2 populations as well as the DO (diversity outbred) mice. Our resource reports baseline values for the eight CC founder strains, obtained by standardized phenotype assays at the German Mouse Clinics. These data will represent an important reference for other phenotype analysis in CC founders and their derived populations.

## Materials and methods

### Ethics statement

Housing and handling of mice was according to the German Animal Welfare Act. All animal experiments were approved by the authority of the Regierung von Oberbayern.

### Mice

The CC founder strains (A/J, C57BL/6J, 129S1/SvImJ, NOD/ShiLtJ, NZO/HILtJ, CAST/EiJ, PWK/PhJ, WSB/EiJ) were purchased from The Jackson Laboratory (Bar Harbor, ME) and bred in our animal facility at the Helmholtz Centre, Braunschweig for two to six generations depending on the strain. All mice were maintained under specific pathogen free conditions and according to the German animal welfare law. At the HZI, mice were housed in IVC cages (Techniplast Sealsafe, Typ 1284L) and paper tissues as cage enrichments with a light–dark cycle of 14 h/10 h without changes to summer savings time. Mice were fed standard diet (Ssniff V1534-300). At the GMC mice were housed in individually ventilated caging (IVC) systems (IVC System Green Line, Tecniplast, Italy), with a 12/12 h light–dark cycle, and red houses as cage enrichment. The IVCs operate at positive pressure. Mice were fed with irradiated standard and breeding rodent diet (Altromin 1314) ad libitum unless indicated otherwise. At 7 weeks of age, up to five cohorts with about four animals per sex and strain were shipped to the Helmholtz Zentrum Munich. Mice were acclimatized for 2 weeks before testing started at 9 weeks of age.

### Phenotyping analysis

The phenotyping assays are described in detail for each project in the MPD database (https://phenome.jax.org/).

### Statistical analysis

For ANOVA, the standard first line analysis in MPD was used (see descriptions in MPD). It used a model that incorporated a combination of fixed factors: sex, strain, and/or label. Label refers to a fixed factor with at least two levels (for example when measurements were taken at different times, e.g., for GMC01, distance traveled, successive 5 min intervals). If more than one fixed factor was present, the interaction term(s) were included in the model. Data were not transformed and were analyzed with the assumption that model residuals were normally distributed. When the label term referred to a repeated measure, a repeated measures ANOVA was used with the same fixed factor setup and subjects set as a random model factor. Repeated measures data were assumed to meet the sphericity criteria. For a global overview, we extracted the ANOVA results for all parameters from MPD (freeze from dated 28.8.2018) and summarized them as follows: *p* values for individual parameters were adjusted for multiple testing using BH correction (Benjamini and Hochberg 1995)—separately for the fixed variables strain, sex and sex:strain interaction.

Second, a pairwise comparison was performed which was based on the IMPC statistical pipeline contrasting parameter measurements for each strain with C57BL/6J as reference (Kurbatova et al. 2015). Data were downloaded from MPD (version from 28th August 2018) and inspected for quality control measures such as missing, mislabeled values and/or dates etc. which were then corrected. An optimized Linear Mixed model (West et al. 2014) with Batch in the random effect were applied to the data, so that the C57B6/J was considered as the baseline group to compare with the other strains. The term “optimized” refers to a backward elimination approach to remove the terms that are not significant (at the level of 0.05) in the saturated model below:

1 $${\text{Response}}~\left( {{\text{parameter}}} \right) = {\text{strain}} + {\text{sex}} + {\text{strain}} \times {\text{sex}}\;{\text{interaction}} + {\text{body weight}} + {\text{batch}}~\left( {{\text{random}}\;{\text{effect}}} \right).$$

The analysis complies with the IMPC statistical pipeline and the implementation in the R package PhenStat (Kurbatova et al. 2015). The outcome of the statistical pipeline was then assigned a mammalian phenotype (MP) term using a modified version of the IMPC algorithm. The detailed results were deposited at the public repository RADAR (link see below). For a global overview, the results of the pairwise comparisons between strains (males and females combined) were then summarized as follows: *p* values from individual comparisons of parameters were adjusted for multiple testing using BH correction (Benjamini and Hochberg 1995). Summarization of ANOVA and pairwise comparison results were performed in R (version 3.4.0).

For the detailed analysis of the heart phenotype (project GMC21), we downloaded the raw data from MPD (freeze from 28th August 2018) comprising a total of 179 data points for 10–12 females and 12 males per strain. Log-transformation of the response variable (heart weight) yielded a normal distribution. We then used a linear regression model to describe the response variable (heart weight), starting with a model that only contained strain as the fixed variable since this variable was the focus of our study. Strain showed a strong significant effect. When sex was added to the model, its significance increased. The interaction strain*sex did not have a significant effect. Also, tibia length did not have a significant effect nor improve the model. However, body weight had a significant effect and further improved the model. The final model which best explained the data was the following: lm(log(heart_wt) ~ strain + sex + body weight. The model was then tested for normality, and eight data points were removed as outliers in two iterative steps to improve normality. This model was then used to determine strain contrasts.

## Data availability

The datasets generated or analyzed during the current study are available at the following public repositories. All primary data generated from this phenotyping project are available in 21 MPD projects (GMC01 to GMC21) at MPD (https://phenome.jax.org/). The raw data for each project are available for download at MPD (https://phenome.jax.org/). Additional datasets (download from MPD used for the analyses described in this manuscript, results from pairwise comparison of each strain with C57BL/6J as reference) were deposited at the public repository RADAR (https://www.radar-service.eu/radar/en/dataset/ItEsCrKZDKoHnjfW?token=aHyoEKSXTFPOnSOFQXXe).

## Electronic supplementary material

Below is the link to the electronic supplementary material.

Supplementary file1 (PDF 79 kb)

Supplementary file2 (PDF 300 kb)

Supplementary file3 (PDF 565 kb)

Supplementary file4 (PDF 664 kb)

Supplementary file5 (PDF 777 kb)

Supplementary file6 (PDF 120 kb)

Supplementary file7 (PDF 105 kb)

Supplementary file8 (PDF 107 kb)

Supplementary file9 (PDF 1125 kb)

Supplementary file10 (PDF 84 kb)

Supplementary file11 (PDF 78 kb)

Supplementary file12 (PDF 177 kb)

Supplementary file13 (PDF 171 kb)
