# Supplementary material for: A comprehensive and comparative phenotypic analysis of the collaborative founder strains identifies new and known phenotypes
Source: Mamm Genome. 2020 Feb 14;31(1):30–48. doi: 10.1007/s00335-020-09827-3 (PMC7060152; doi:10.1007/s00335-020-09827-3)
Supplement: Supplementary file 1 — Supplementary file1 (PDF 79 kb) [file 335_2020_9827_MOESM1_ESM.pdf]

Supplemental material

A comprehensive and comparative phenotypic analysis of the Collaborative Founder strains identifies new and known phenotypes

Heike Kollmus[1], Helmut Fuchs[2], Christoph Lengger[2], Hamed Haselimashhadi[3], Molly A. Bogue[4], Manuela A. Östereicher[2], Marion Horsch[2], Thure Adler[2], Juan Antonio Aguilar-Pimentel[2], Oana Veronica Amarie[2,5], Lore Becker[2], Johannes Beckers[2,6,7], Julia Calzada-Wack[2], Lillian Garrett[2,5], Wolfgang Hans[2], Sabine M. Hölter[2,5], Tanja Klein-Rodewald[2], Holger Maier[2], Philipp Mayer-Kuckuk[2], Gregor Miller[2], Kristin Moreth[2], Frauke Neff[2], Birgit Rathkolb[2,7,8], Ildikó Rácz[2#] , Jan Rozman[2,7], Nadine Spielmann[2], Irina Treise[2], Dirk Busch[2,9], Jochen Graw[5,13], Thomas Klopstock[10,11,12], Eckhard Wolf[8], Wolfgang Wurst[5,11,12,14], Ali Önder Yildirim[15], Jeremy Mason[3], Arturo Torres[3], Mouse Phenome Database Team[4], Rudi Balling[16], Terry Mehaan[3], Valerie Gailus-Durner[2], Klaus Schughart[*,1,17,18,19,20], Martin Hrabě de Angelis[*,2,6,7,19].

[1]*Department of Infection Genetics, Helmholtz Centre for Infection Research, Inhoffenstr.7, 38124 Braunschweig, Germany*

[2]*German Mouse Clinic, Institute of Experimental Genetics , Helmholtz Zentrum München, German Research Center for Environmental Health, Ingolstädter Landstrasse 1, 85764 Neuherberg, Germany*

[3]*European Molecular Biology Laboratory, European Bioinformatics Institute, Wellcome Trust Genome Campus, Hinxton, Cambridge CB10 1SD, UK*

[4]*The Jackson Laboratory, Bar Harbor, Maine 04609, USA*

[5]*Institute of Developmental Genetics, Helmholtz Zentrum München, German Research Center for Environmental Health, Ingolstädter Landstrasse 1, 85764 Neuherberg, Germany*

[6]*Chair of Experimental Genetics, School of Life Science Weihenstephan, Technische Universität München, Alte Akademie 8, 85354 Freising, Germany*

[7]*German Center for Diabetes Research (DZD), Ingolstädter Landstr. 1, 85764 Neuherberg, Germany*

[8]*Institute of Molecular Animal Breeding and Biotechnology, Gene Center, Ludwig-Maximilians-University München, Feodor-Lynen Str. 25, 81377 Munich, Germany*

[9]*Institute for Medical Microbiology, Immunology and Hygiene, Technische Universität München, Trogerstrasse 30, 81675 Munich, Germany.*

[10]*Dept. of Neurology, Friedrich-Baur-Institute, Klinikum der Ludwig-Maximilians-Universität München, Ziemssenstr. 1a, 80336 Munich, Germany*

[11]*Deutsches Zentrum für Neurodegenerative Erkrankungen (DZNE) Site Munich, Feodor-Lynen-Str. 17, 81377 Munich, Germany*

[12]*Munich Cluster for Systems Neurology (SyNergy), Adolf-Butenandt-Institut, Ludwig-Maximilians-Universität München, Feodor-Lynen-Str. 17, 81377 Munich, Germany*

[13]*retired*

[14]*Chair of Developmental Genetics, Technische Universität München-Weihenstephan, c/o Helmholtz Zentrum München, Ingolstädter Landstr. 1, 85764 Neuherberg, Germany*
[15]*Institute of Lung Biology and Disease , Helmholtz Zentrum München, German Research Center for Environmental Health, Ingolstädter Landstrasse 1, D-85764 Neuherberg, Germany and Member of the German Center for Lung Research*
[16]*Luxembourg Centre for Systems Biomedicine (LCSB), University of Luxembourg, Luxembourg*
[17]*University of Veterinary Medicine Hannover, Germany*
[18]*University of Tennessee Health Science Center, Memphis, TN, USA*
[19]*authors contributed equally*
[20]*author for correspondence* at: Helmholtz Centre for Infection Research, Dept. of Infection Genetics, Inhoffenstr.7, D-38124 Braunschweig, Germany
E-mail address: klaus.schughart@helmholtz-hzi.de
*equal contributions as last authors.
[#]present address: Clinic of Neurodegenerative Diseases and Gerontopsychiatry, University of Bonn Medical Center, Bonn, Germany

Fig. S1 Outline of the phenotyping pipeline

Shaded boxes show lower animal numbers than 16 mice per sex.

Fig. S2 Heat maps of GMC projects

Individual heat maps of all parameters from a given GMC project are shown.

Fig. S3 Correlation heat maps for all measured parameters

Heat map of Pearson correlations for all individual parameters are shown.

Fig. S4 Effect of batch and strain

For each parameter, the measurements with reference to batch and strain are plotted.

Table S1 Significant parameters from ANOVA for strain as explanatory variable

measnum: MPD ID of measurement; projsym: project in MPD; varname: parameter name; descrip: Description of parameter; units: measured units of parameter; aspect: explanatory variable; mean_sq: mean of squares; sum_sq: sum of squares; precise_pvalue_prf: p-value not corrected for multiple testing; adj.p.value.BH: p-value adjusted for multiple testing using BH correction.

Table S2 Significant parameters from ANOVA for sex as explanatory variable

measnum: MPD ID of measurement; projsym: project in MPD; varname: parameter name; descrip: Description of parameter; units: measured units of parameter; aspect: explanatory variable; mean_sq: mean of squares; sum_sq: sum of squares; precise_pvalue_prf: p-value not corrected for multiple testing; adj.p.value.BH: p-value adjusted for multiple testing using BH correction.

Table S3 Significant parameters from ANOVA for strain and sex interaction

measnum: MPD ID of measurement; projsym: project in MPD; varname: parameter name; descrip: Description of parameter; units: measured units of parameter; aspect: explanatory variable; mean_sq: mean of squares; sum_sq: sum of squares; precise_pvalue_prf: p-value not corrected for multiple testing; adj.p.value.BH: p-value adjusted for multiple testing using BH correction.

Table S4 Correlations between parameters

The table shows the results from the correlation analysis of all parameters used to generate the correlation heat map.

Table S5: ANOVA of comparisons between wild-derived, laboratory and diseases model strains

Results from ANOVA with parameter measurements as response variable and strain groups (wild-derived, laboratory and diseases model strains) as explanatory variables.

Table S6 Analysis of batch effects

Results from an ANOVA for all measured parameters as response variable and batch (shipment date) as explanatory variable (model: parameter ~ batch). p-values were not corrected for multiple testing.

Table S7 Examples of power calculations from selected trait data

Three significant traits from the pair-wise comparisons were selected from projects GMC1 and GMC16 and the group sizes were calculated with the R function power.t.test using the following settings: power=0.8, significance level (sig.level)=0.05, standard deviation (sd) as the mean of all sd values for the selected parameter over all strains, delta=difference in means between strain 129S1/SvlmJ and any other strain. For example for the comparison of center_distance_total between strain 129S1/SvlmJ and C57BL/6J, a group size of n=3 would have been enough to detect the observed difference between the two strains. For the comparison of center_distance_total between strain 129S1/SvlmJ and NZO/HlLtJ, a group size of n=20 would have been necessary to obtain the observed difference between the two strains. For the comparison of iron at day 21 between strain 129S1/SvlmJ and A/J, a group size of n=28 would have been necessary to obtain the observed difference between the two strains. For the comparison of iron at day 21 between strain 129S1/SvlmJ and NOD/ShiLtJ, the theoretical group size is n=2282. Thus, this difference is too small and not be significant between the two strains.

Table S8 Significance of explanatory variables from analysis of project GMC21 heart weight

The table shows the results from the statistical analysis obtained from the final linear model: lm(log(heart_wt) ~ strain + sex + bw). All three explanatory variables, strain, sex and body weight (bw), are significant.
