# Supplementary material for: A comprehensive and comparative phenotypic analysis of the collaborative founder strains identifies new and known phenotypes
Source: Mamm Genome. 2020 Feb 14;31(1):30–48. doi: 10.1007/s00335-020-09827-3 (PMC7060152; doi:10.1007/s00335-020-09827-3)
Supplement: Supplementary file 3 — Supplementary file3 (PDF 565 kb) [file 335_2020_9827_MOESM3_ESM.pdf]

Figure S2

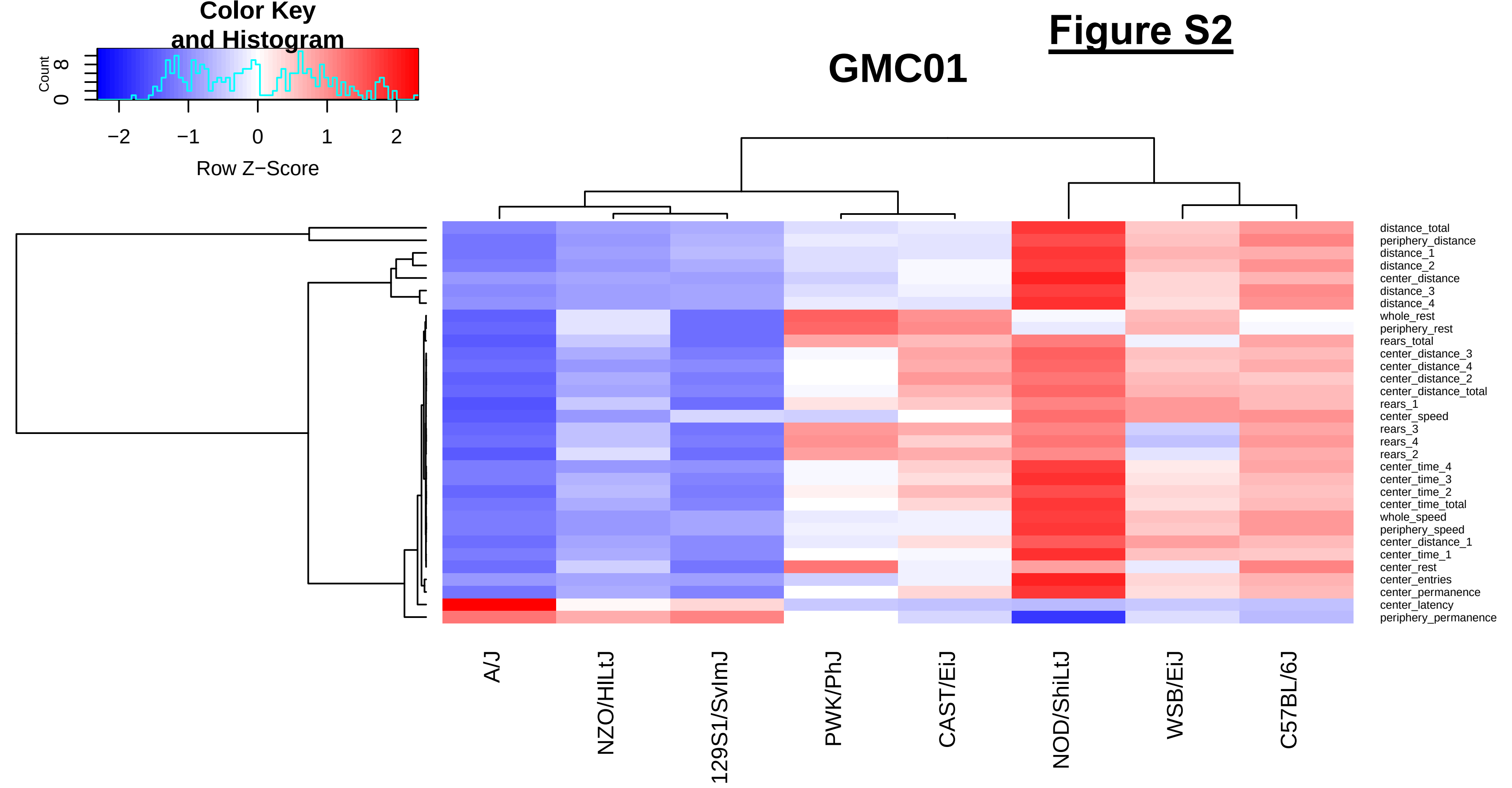
Color Key
and Histogram
Count
0
8
−2
−1
0
1
2
Row Z−Score
GMC01
distance_total
periphery_distance
distance_1
distance_2
center_distance
distance_3
distance_4
whole_rest
periphery_rest
rears_total
center_distance_3
center_distance_4
center_distance_2
center_distance_total
rears_1
center_speed
rears_3
rears_4
rears_2
center_time_4
center_time_3
center_time_2
center_time_total
whole_speed
periphery_speed
center_distance_1
center_time_1
center_rest
center_entries
center_permanence
center_latency
periphery_permanence
A/J
NZO/HlLtJ
129S1/SvImJ
PWK/PhJ
CAST/EiJ
NOD/ShiLtJ
WSB/EiJ
C57BL/6J

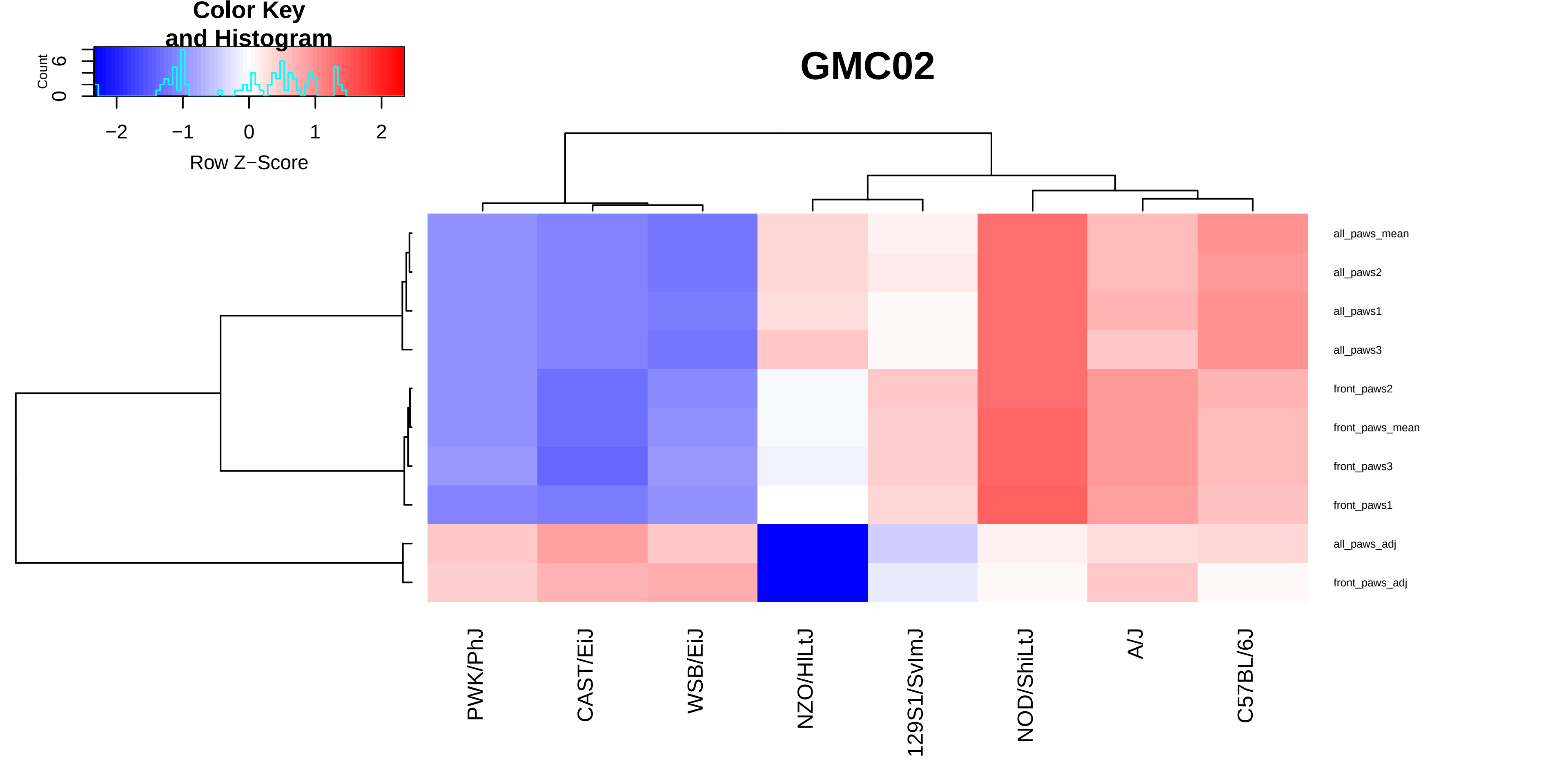

Color Key
and Histogram
Count
0
6
−2
−1
0
1
2
Row Z−Score
GMC02
all_paws_mean
all_paws2
all_paws1
all_paws3
front_paws2
front_paws_mean
front_paws3
front_paws1
all_paws_adj
front_paws_adj
PWK/PhJ
CAST/EiJ
WSB/EiJ
NZO/HlLtJ
129S1/SvImJ
NOD/ShiLtJ
A/J
C57BL/6J

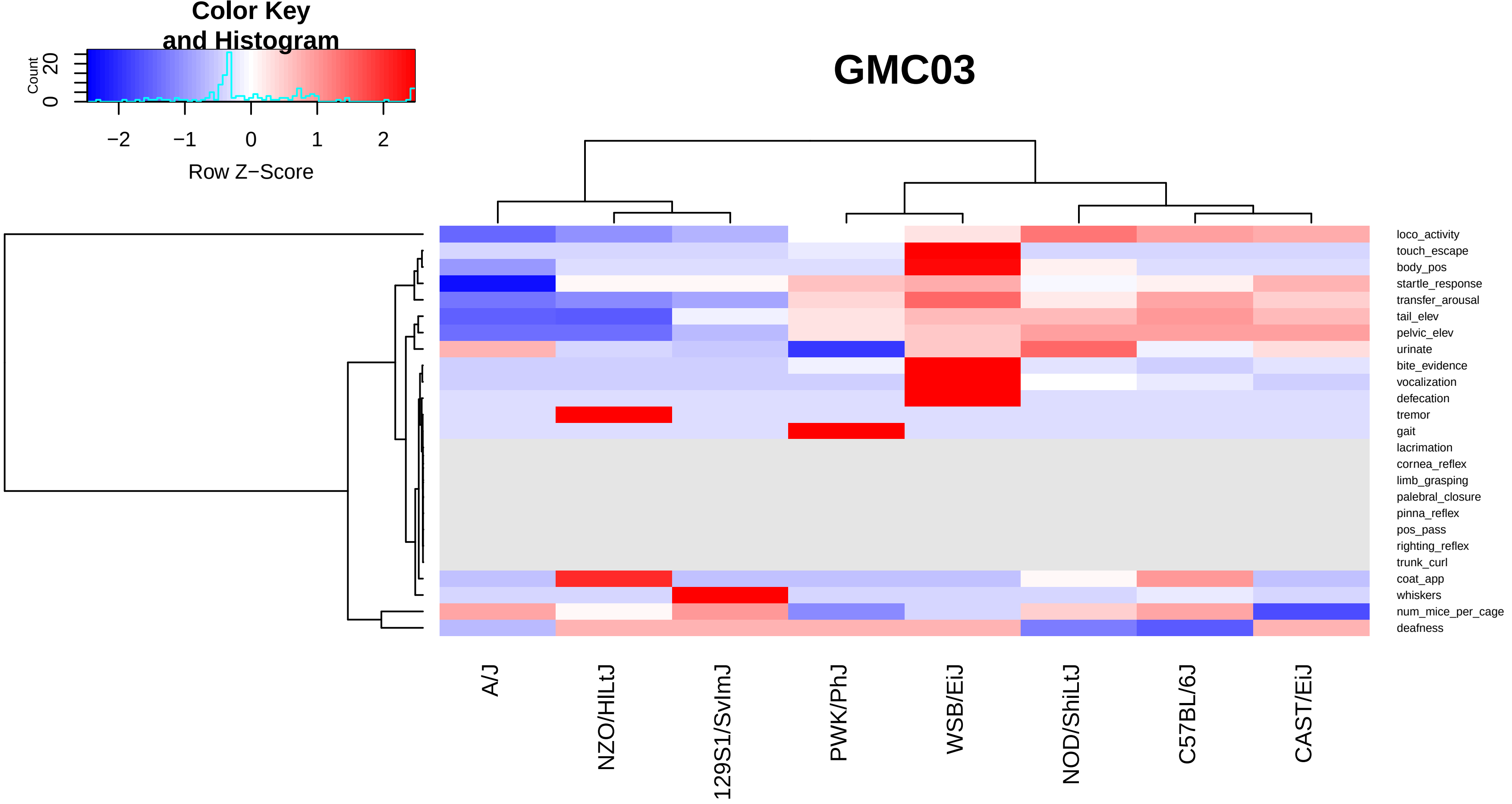

Color Key
and Histogram
Count
0
20
−2
−1
0
1
2
Row Z−Score
GMC03
loco_activity
touch_escape
body_pos
startle_response
transfer_arousal
tail_elev
pelvic_elev
urinate
bite_evidence
vocalization
defecation
tremor
gait
lacrimation
cornea_reflex
limb_grasping
palebral_closure
pinna_reflex
pos_pass
righting_reflex
trunk_curl
coat_app
whiskers
num_mice_per_cage
deafness
A/J
NZO/HlLtJ
129S1/SvImJ
PWK/PhJ
WSB/EiJ
NOD/ShiLtJ
C57BL/6J
CAST/EiJ

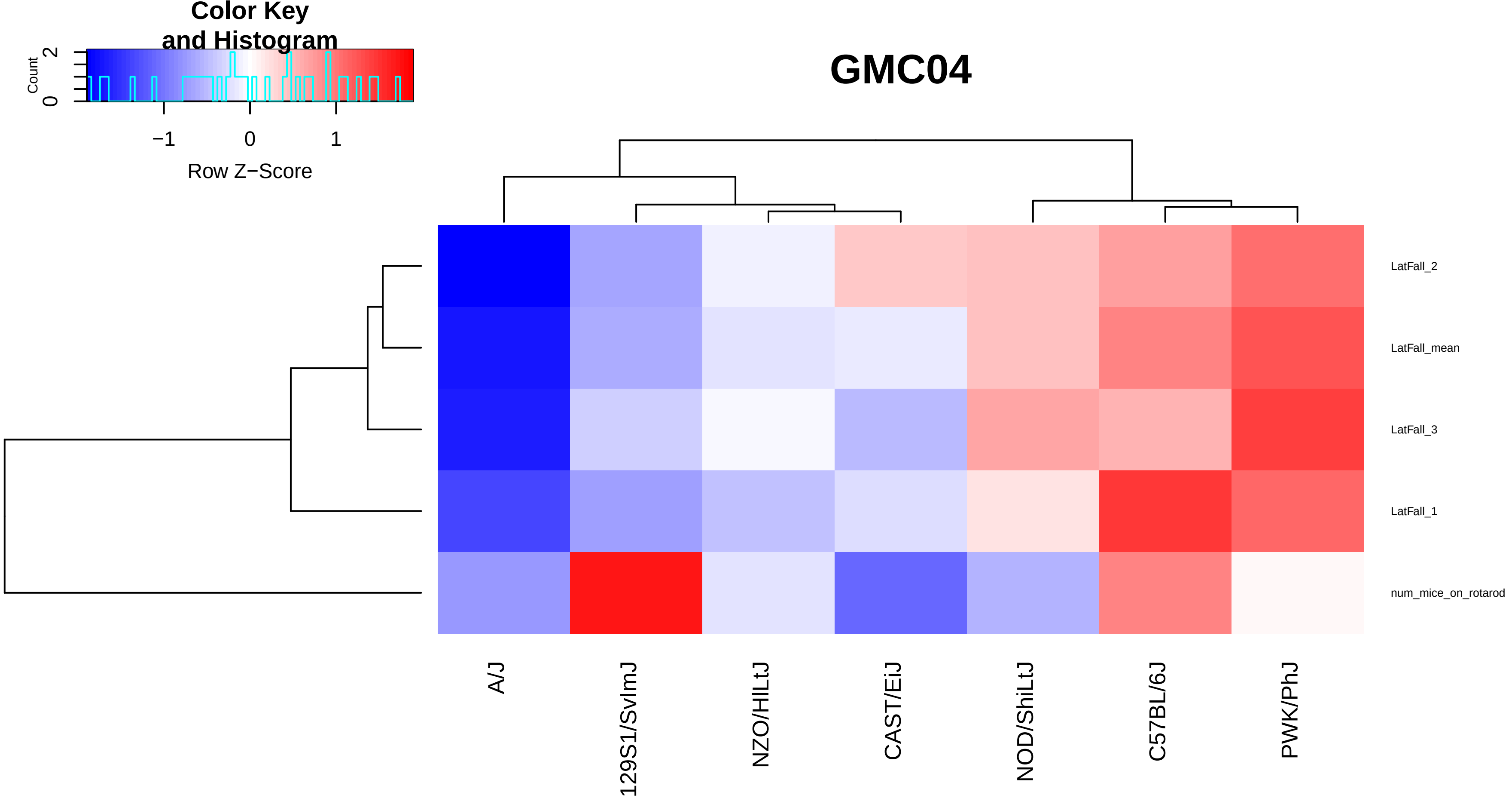
Color Key
and Histogram
Count
0
2
−1
0
1
Row Z−Score
GMC04
LatFall_2
LatFall_mean
LatFall_3
LatFall_1
num_mice_on_rotarod
A/J
129S1/SvImJ
NZO/HlLtJ
CAST/EiJ
NOD/ShiLtJ
C57BL/6J
PWK/PhJ

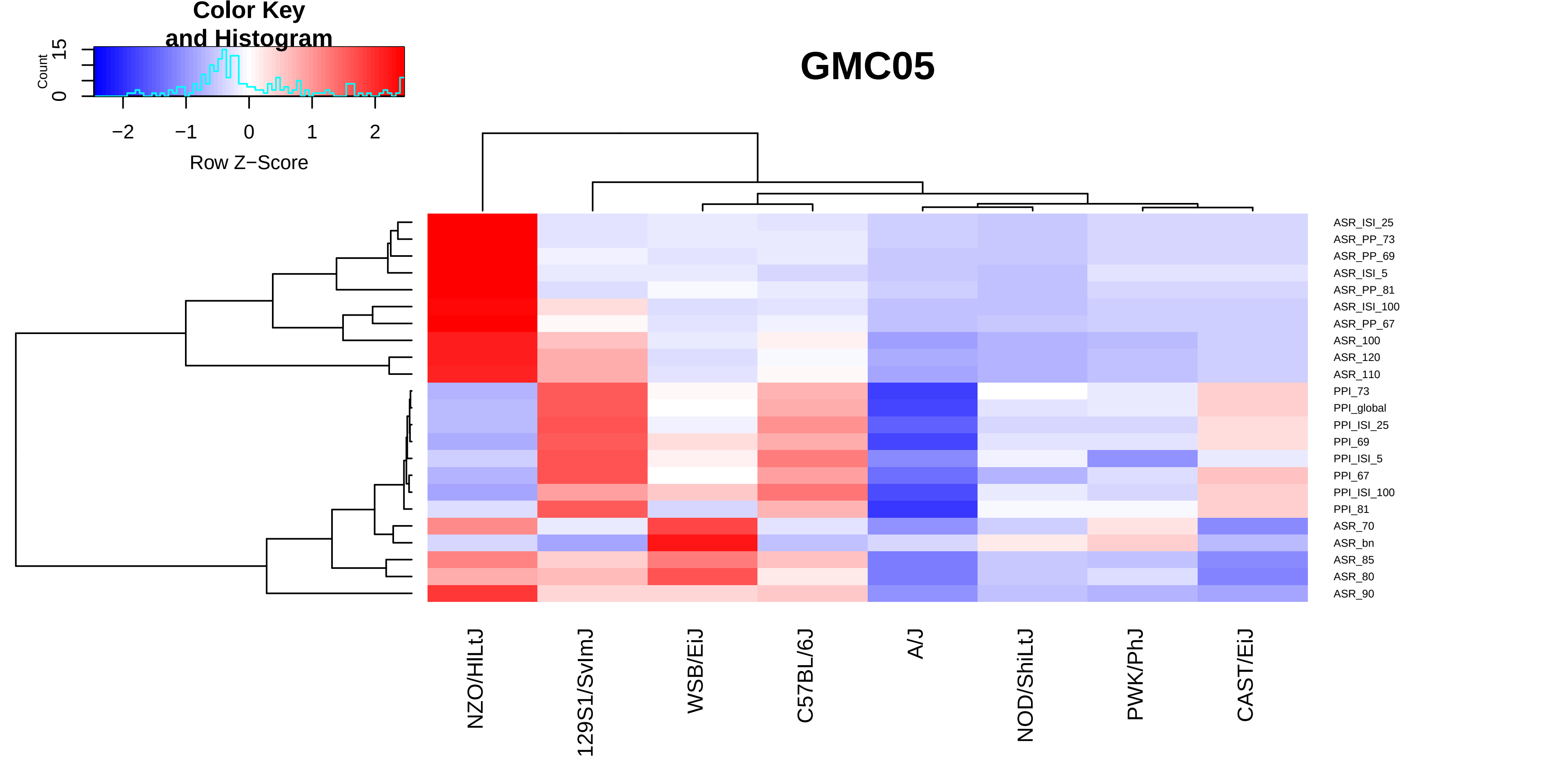
Color Key
and Histogram
Count
0
15
−2
−1
0
1
2
Row Z−Score
GMC05
ASR_ISI_25
ASR_PP_73
ASR_PP_69
ASR_ISI_5
ASR_PP_81
ASR_ISI_100
ASR_PP_67
ASR_100
ASR_120
ASR_110
PPI_73
PPI_global
PPI_ISI_25
PPI_69
PPI_ISI_5
PPI_67
PPI_ISI_100
PPI_81
ASR_70
ASR_bn
ASR_85
ASR_80
ASR_90
NZO/HILtJ
129S1/SvImJ
WSB/EiJ
C57BL/6J
A/J
NOD/ShiLtJ
PWK/PhJ
CAST/EiJ

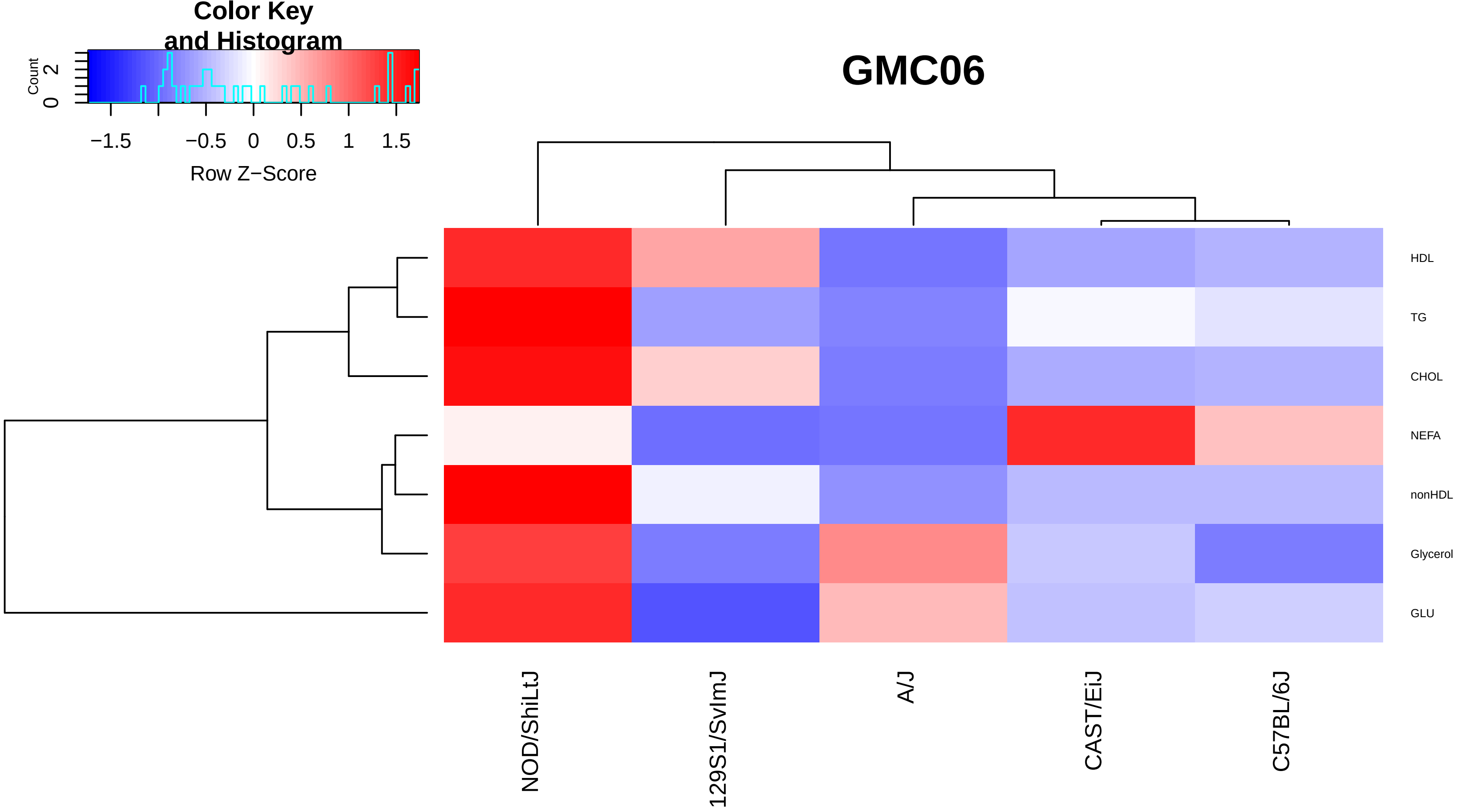

Color Key
and Histogram
Count
0
2
−1.5
−0.5
0
0.5
1
1.5
Row Z−Score
GMC06
HDL
TG
CHOL
NEFA
nonHDL
Glycerol
GLU
NOD/ShiLtJ
129S1/SvImJ
A/J
CAST/EiJ
C57BL/6J

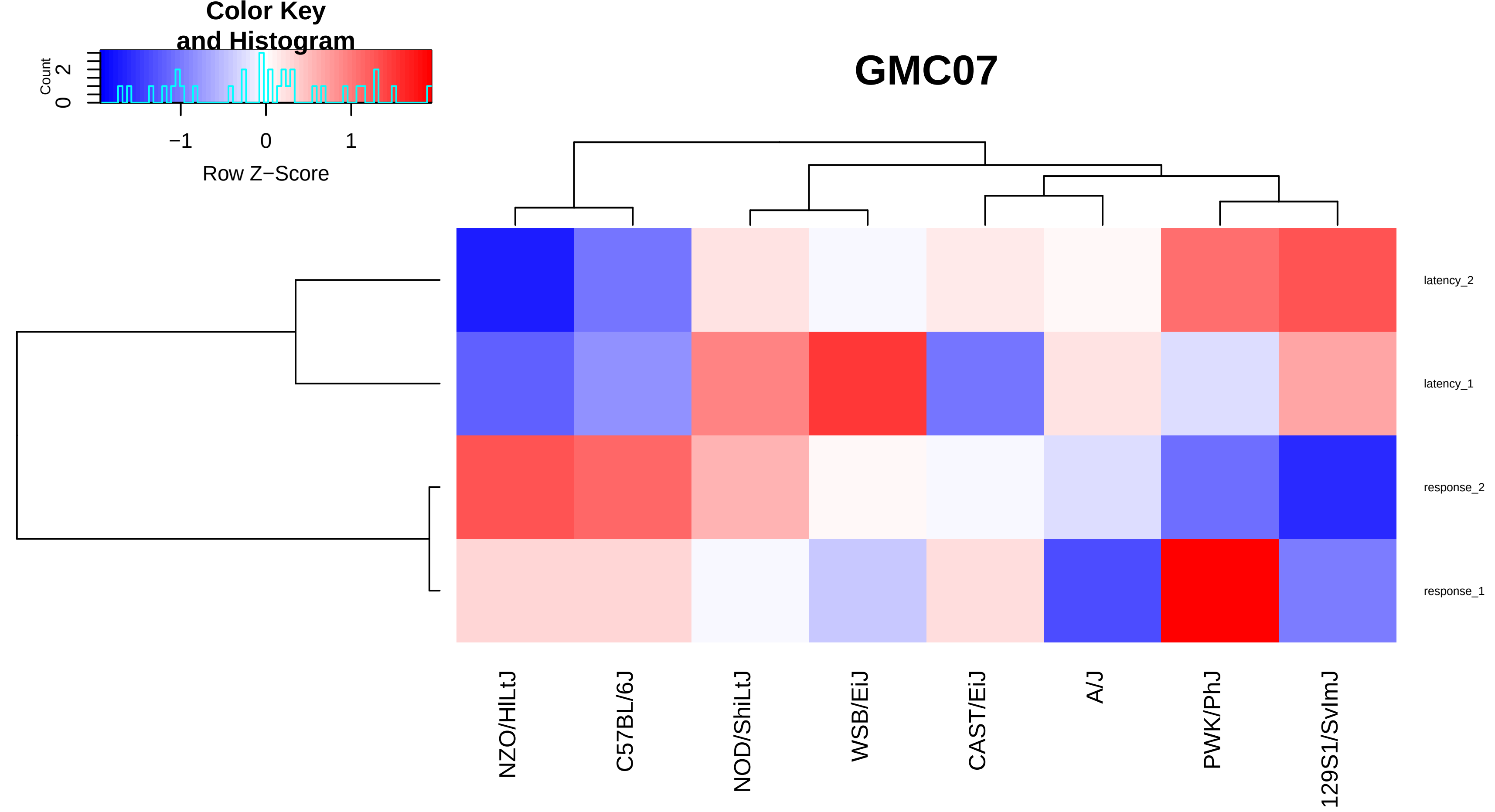
Color Key
and Histogram
Count
0
2
−1
0
1
Row Z−Score
GMC07
latency_2
latency_1
response_2
response_1
NZO/HlLtJ
C57BL/6J
NOD/ShiLtJ
WSB/EiJ
CAST/EiJ
A/J
PWK/PhJ
129S1/SvImJ

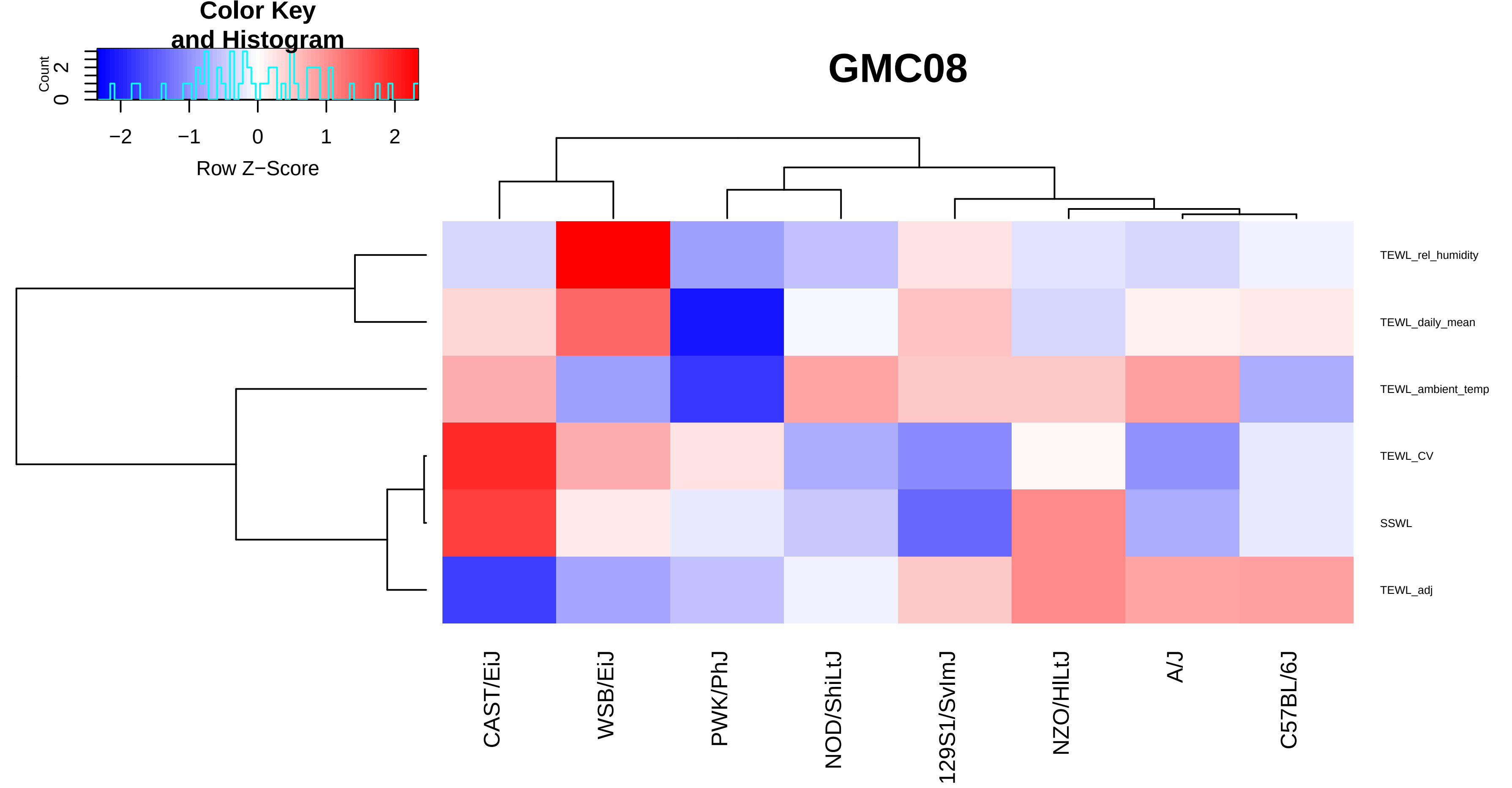

Color Key
and Histogram
Count
0
2
−2
−1
0
1
2
Row Z−Score
GMC08
TEWL_rel_humidity
TEWL_daily_mean
TEWL_ambient_temp
TEWL_CV
SSWL
TEWL_adj
CAST/EiJ
WSB/EiJ
PWK/PhJ
NOD/ShiLtJ
129S1/SvImJ
NZO/HlLtJ
A/J
C57BL/6J

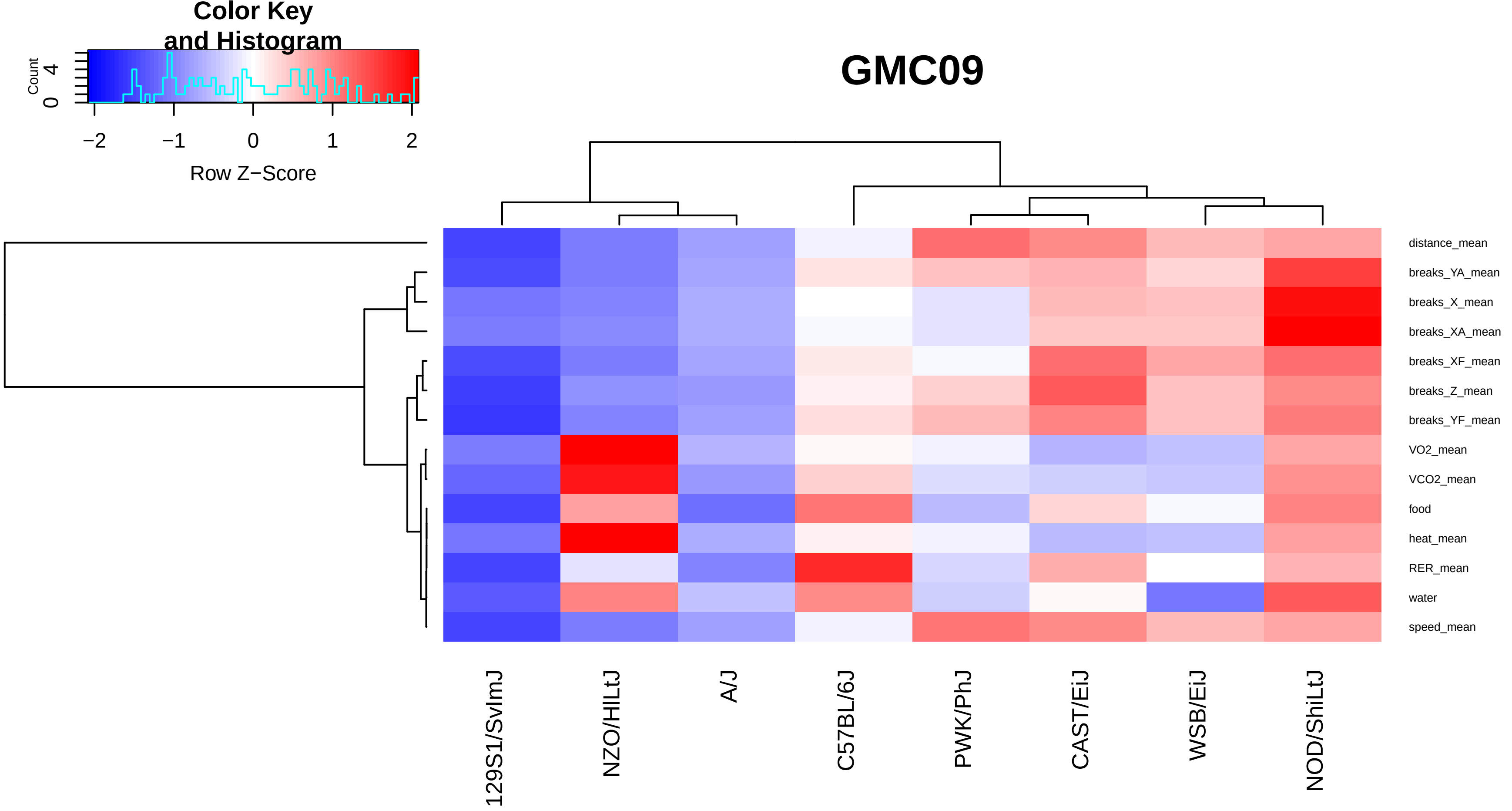

Color Key
and Histogram
Count
0
4
−2
−1
0
1
2
Row Z−Score
GMC09
distance_mean
breaks_YA_mean
breaks_X_mean
breaks_XA_mean
breaks_XF_mean
breaks_Z_mean
breaks_YF_mean
VO2_mean
VCO2_mean
food
heat_mean
RER_mean
water
speed_mean
129S1/SvImJ
NZO/HILtJ
A/J
C57BL/6J
PWK/PhJ
CAST/EiJ
WSB/EiJ
NOD/ShiLtJ

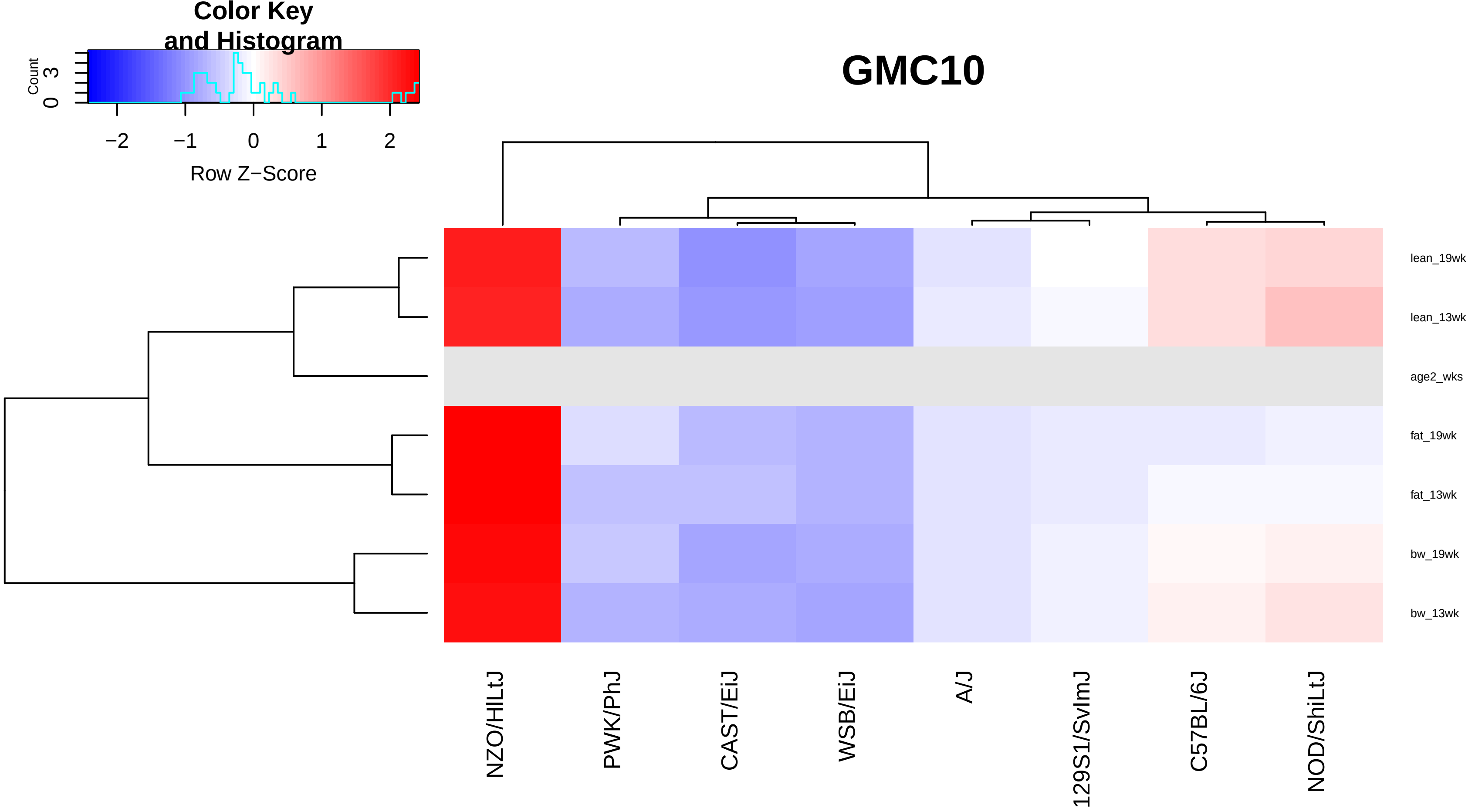
Color Key
and Histogram
Count
0
3
−2
−1
0
1
2
Row Z−Score
GMC10
lean_19wk
lean_13wk
age2_wks
fat_19wk
fat_13wk
bw_19wk
bw_13wk
NZO/HlLtJ
PWK/PhJ
CAST/EiJ
WSB/EiJ
A/J
129S1/SvImJ
C57BL/6J
NOD/ShiLtJ

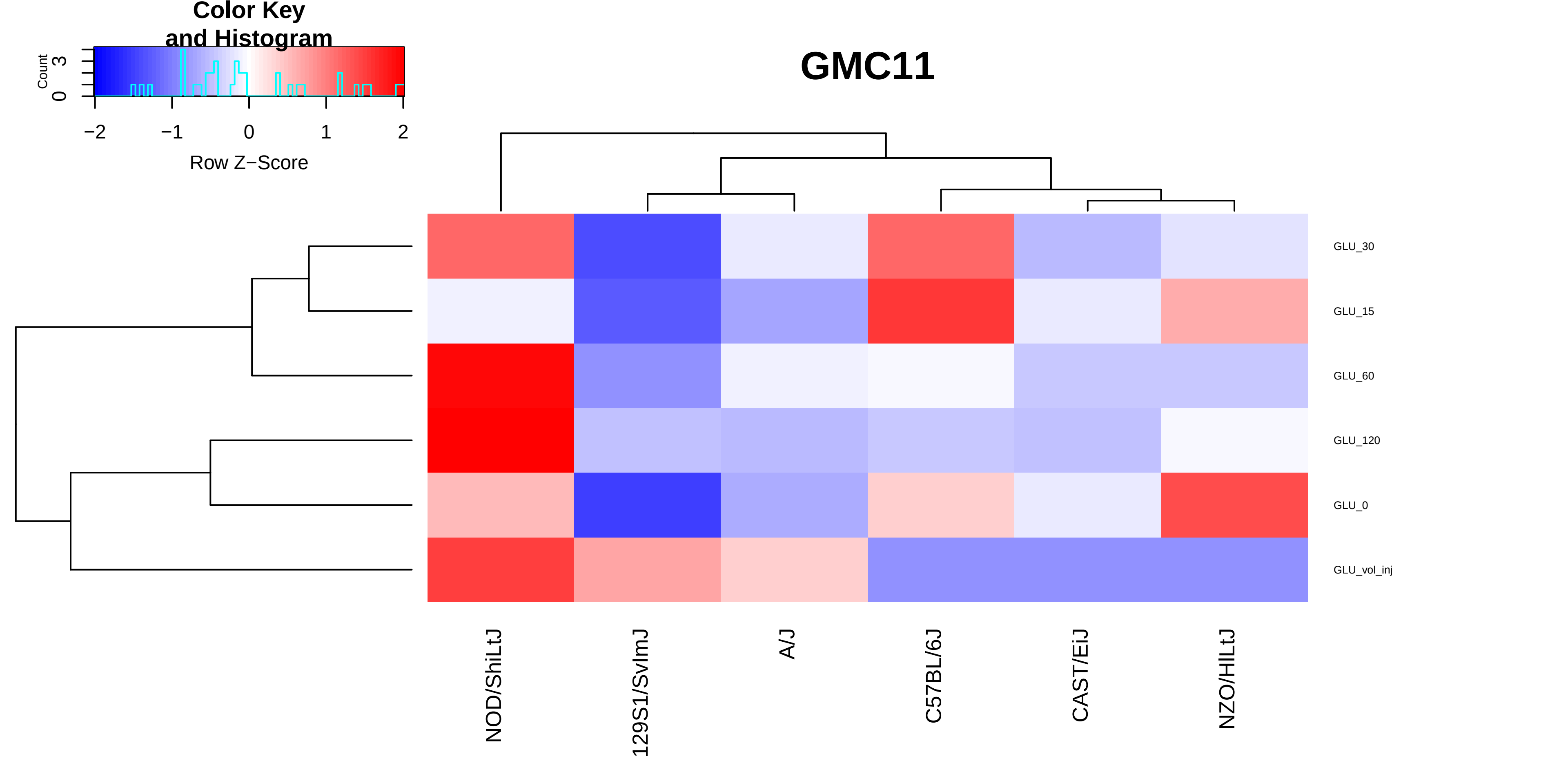

Color Key
and Histogram
Count
0
3
−2
−1
0
1
2
Row Z−Score
GMC11
GLU_30
GLU_15
GLU_60
GLU_120
GLU_0
GLU_vol_inj
NOD/ShiLtJ
129S1/SvImJ
A/J
C57BL/6J
CAST/EiJ
NZO/HlLtJ

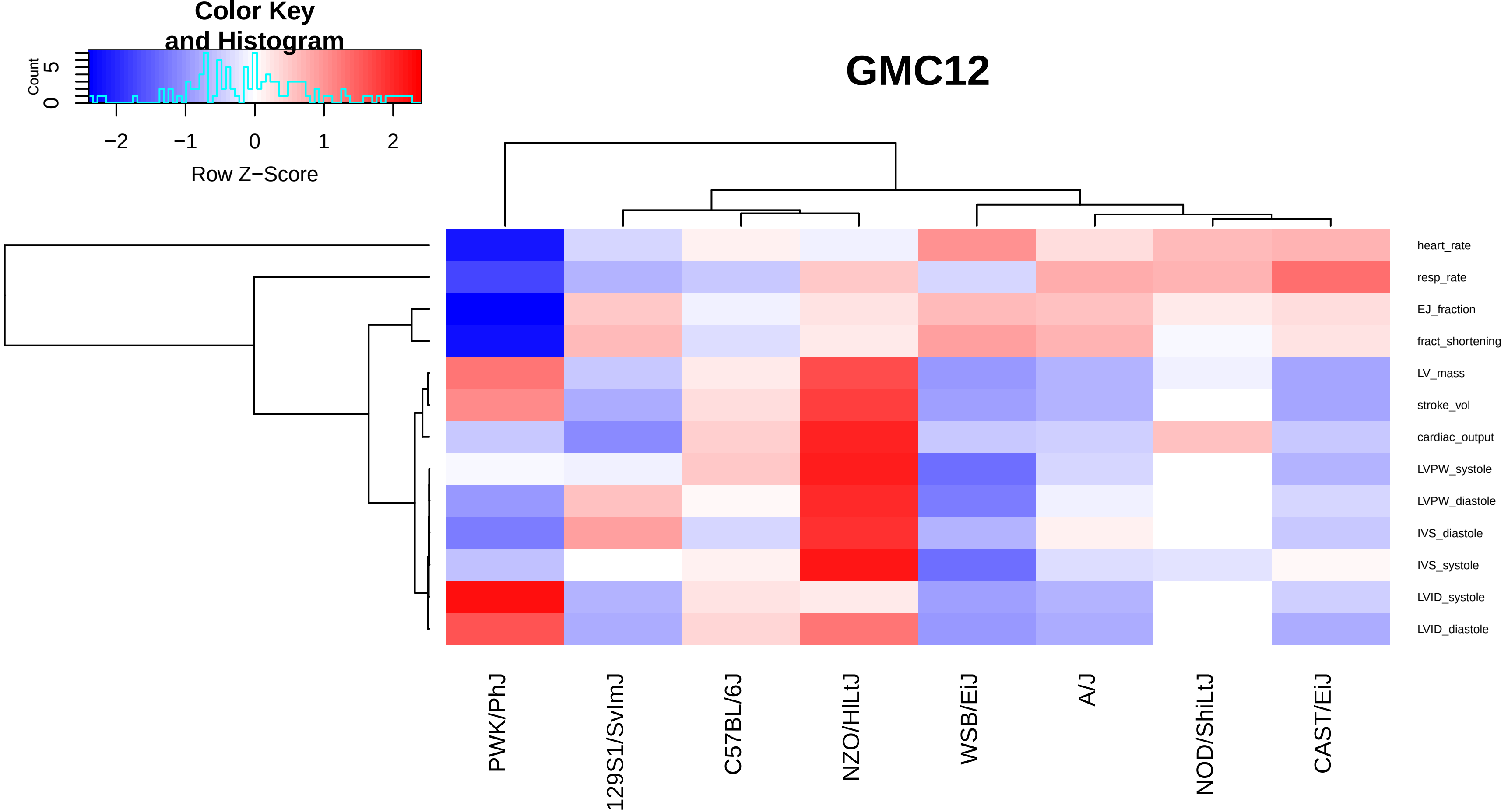
Color Key
and Histogram
Count
0
5
−2
−1
0
1
2
Row Z−Score
GMC12
heart_rate
resp_rate
EJ_fraction
fract_shortening
LV_mass
stroke_vol
cardiac_output
LVPW_systole
LVPW_diastole
IVS_diastole
IVS_systole
LVID_systole
LVID_diastole
PWK/PhJ
129S1/SvImJ
C57BL/6J
NZO/HlLtJ
WSB/EiJ
A/J
NOD/ShiLtJ
CAST/EiJ

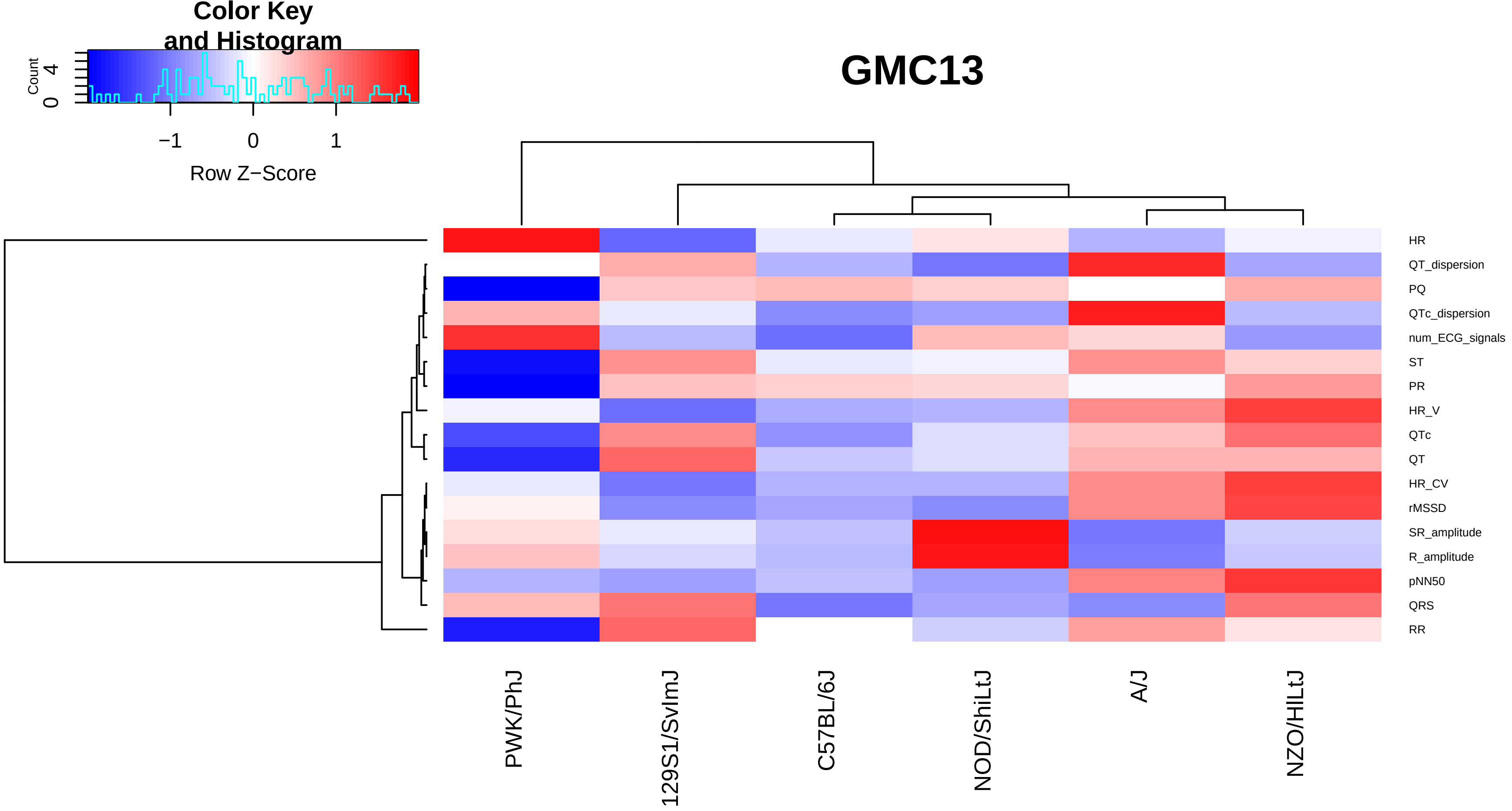

Color Key
and Histogram
Count
0
4
−1
0
1
Row Z−Score
GMC13
HR
QT_dispersion
PQ
QTc_dispersion
num_ECG_signals
ST
PR
HR_V
QTc
QT
HR_CV
rMSSD
SR_amplitude
R_amplitude
pNN50
QRS
RR
PWK/PhJ
129S1/SvImJ
C57BL/6J
NOD/ShiLtJ
A/J
NZO/HlLtJ

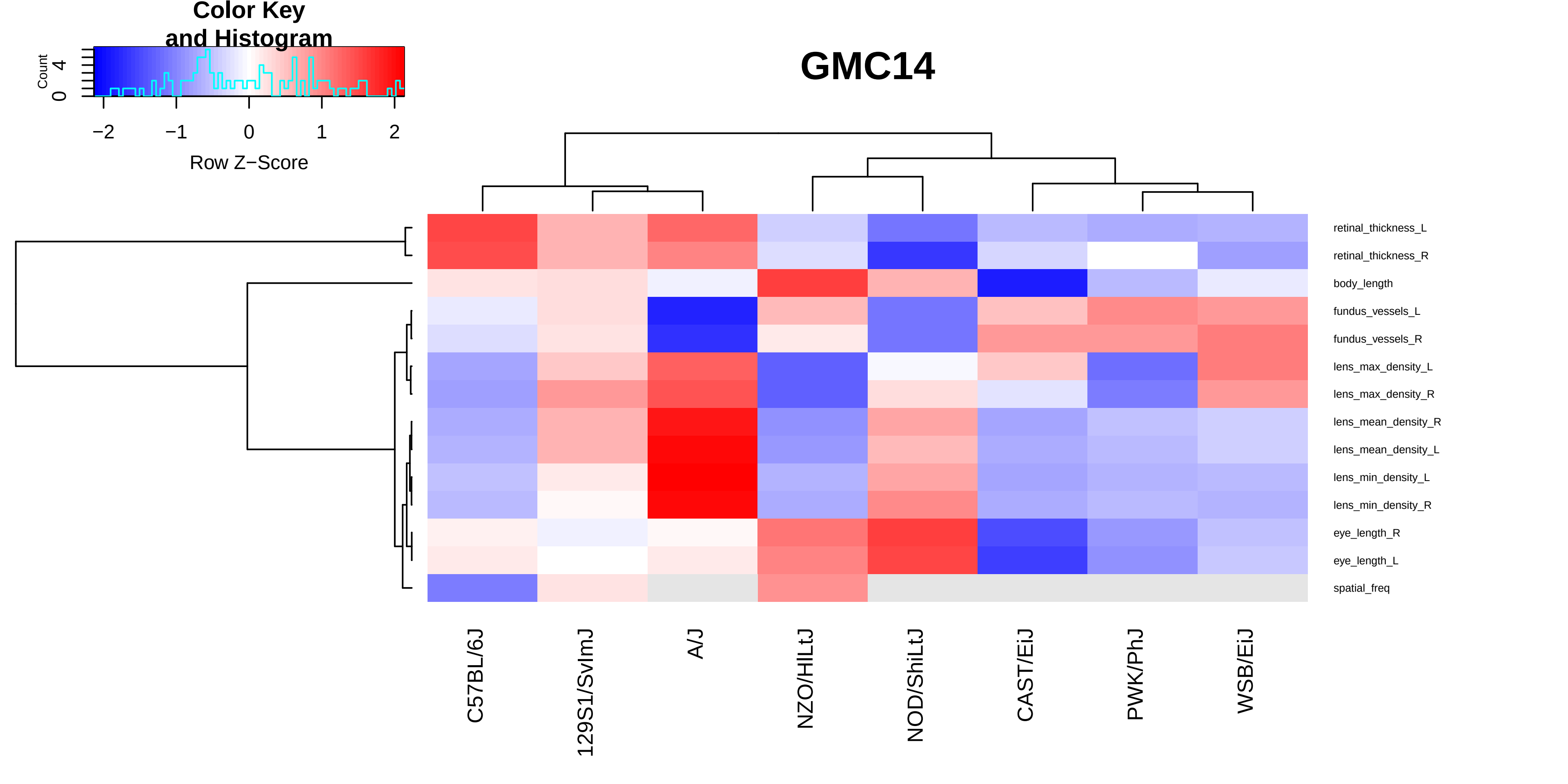

Color Key
and Histogram
Count
0
4
−2
−1
0
1
2
Row Z−Score
GMC14
retinal_thickness_L
retinal_thickness_R
body_length
fundus_vessels_L
fundus_vessels_R
lens_max_density_L
lens_max_density_R
lens_mean_density_R
lens_mean_density_L
lens_min_density_L
lens_min_density_R
eye_length_R
eye_length_L
spatial_freq
C57BL/6J
129S1/SvImJ
A/J
NZO/HlLtJ
NOD/ShiLtJ
CAST/EiJ
PWK/PhJ
WSB/EiJ

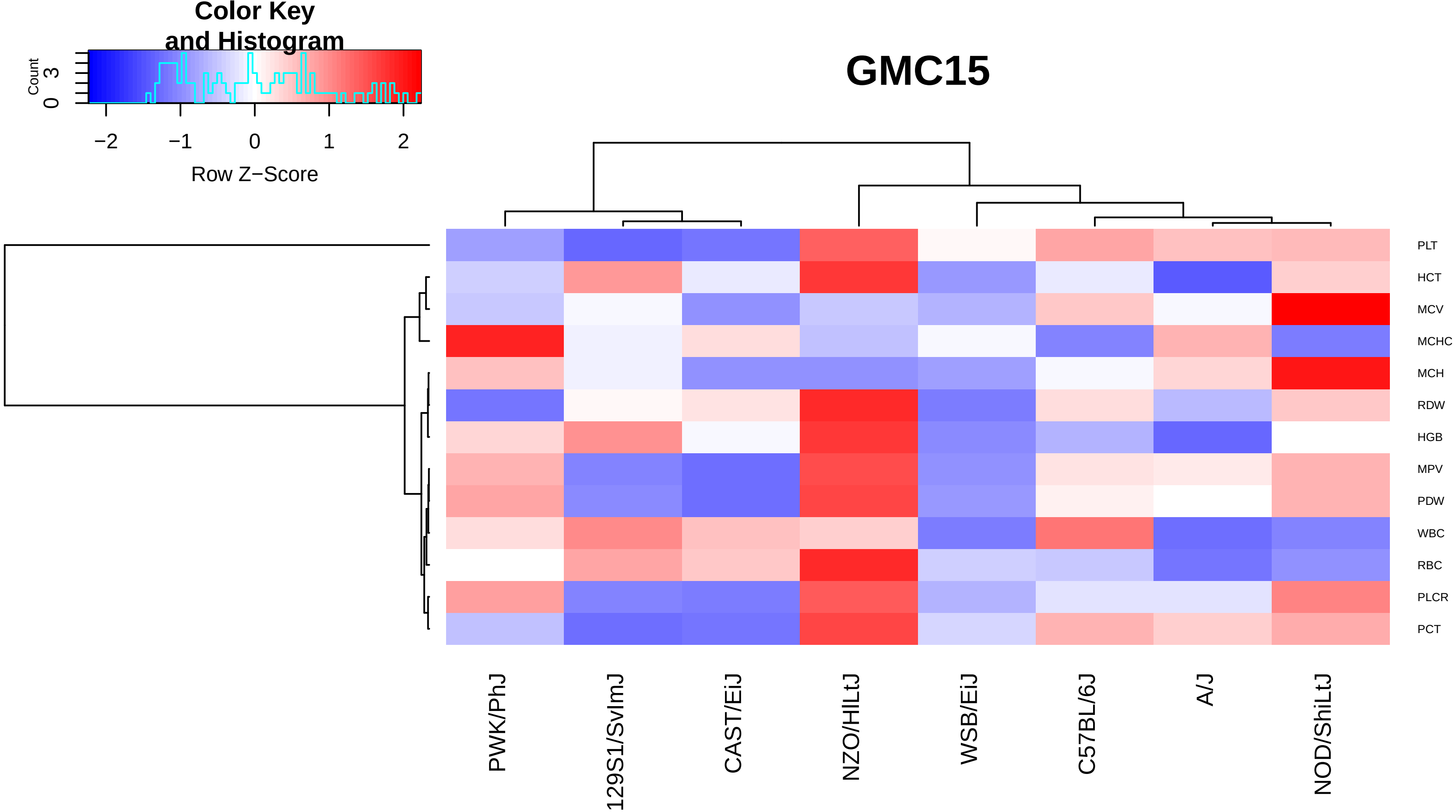

Color Key
and Histogram
Count
0
3
−2
−1
0
1
2
Row Z−Score
GMC15
PLT
HCT
MCV
MCHC
MCH
RDW
HGB
MPV
PDW
WBC
RBC
PLCR
PCT
PWK/PhJ
129S1/SvImJ
CAST/EiJ
NZO/HlLtJ
WSB/EiJ
C57BL/6J
A/J
NOD/ShiLtJ

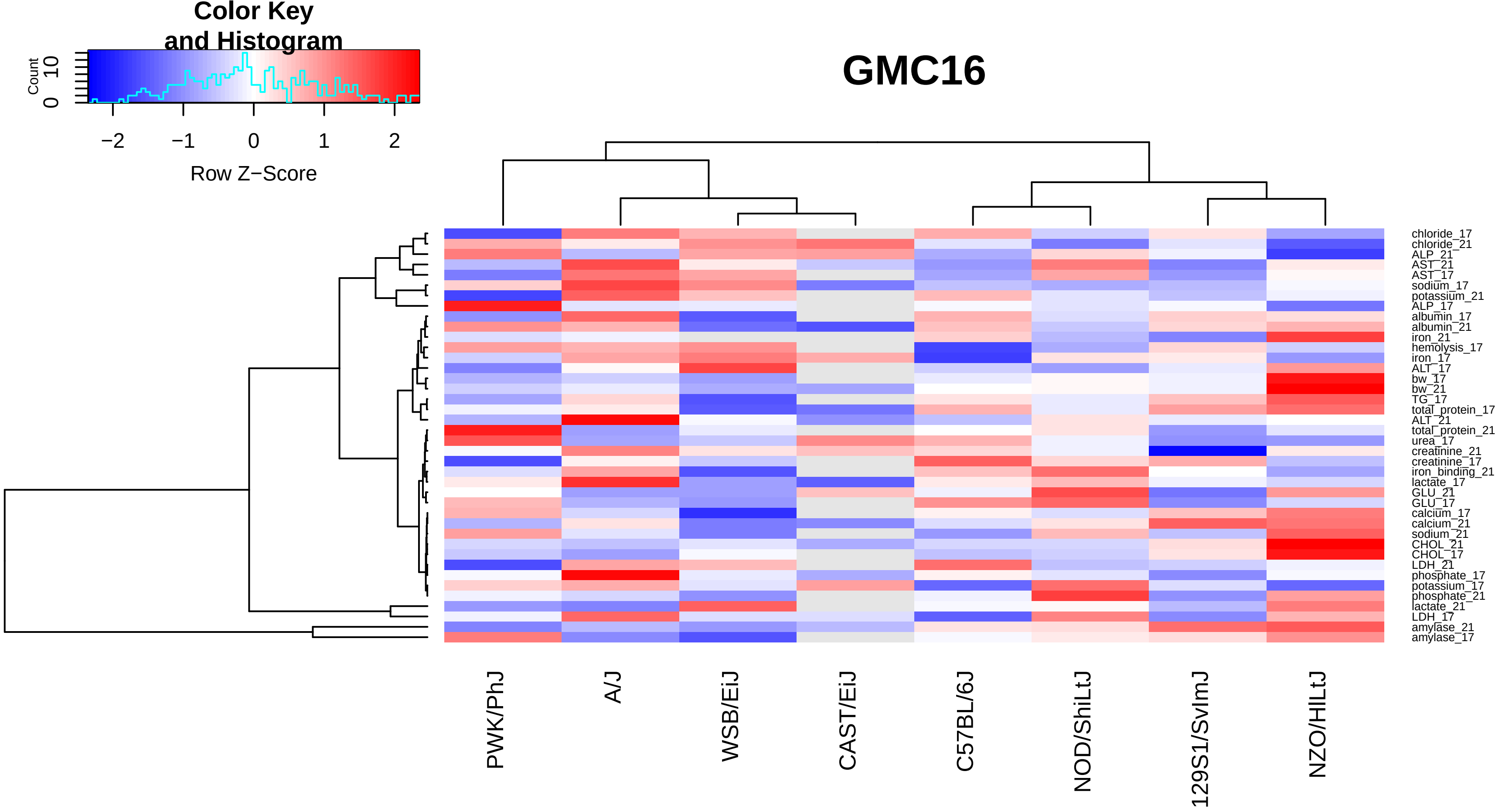

Color Key
and Histogram
Count
0
10
−2
−1
0
1
2
Row Z−Score
GMC16
chloride_17
chloride_21
ALP_21
AST_21
AST_17
sodium_17
potassium_21
ALP_17
albumin_17
albumin_21
iron_21
hemolysis_17
iron_17
ALT_17
bw_17
bw_21
TG_17
total_protein_17
ALT_21
total_protein_21
urea_17
creatinine_21
creatinine_17
iron_binding_21
lactate_17
GLU_21
GLU_17
calcium_17
calcium_21
sodium_21
CHOL_21
CHOL_17
LDH_21
phosphate_17
potassium_17
phosphate_21
lactate_21
LDH_17
amylase_21
amylase_17
PWK/PhJ
A/J
WSB/EiJ
CAST/EiJ
C57BL/6J
NOD/ShiLtJ
129S1/SvImJ
NZO/HlLtJ

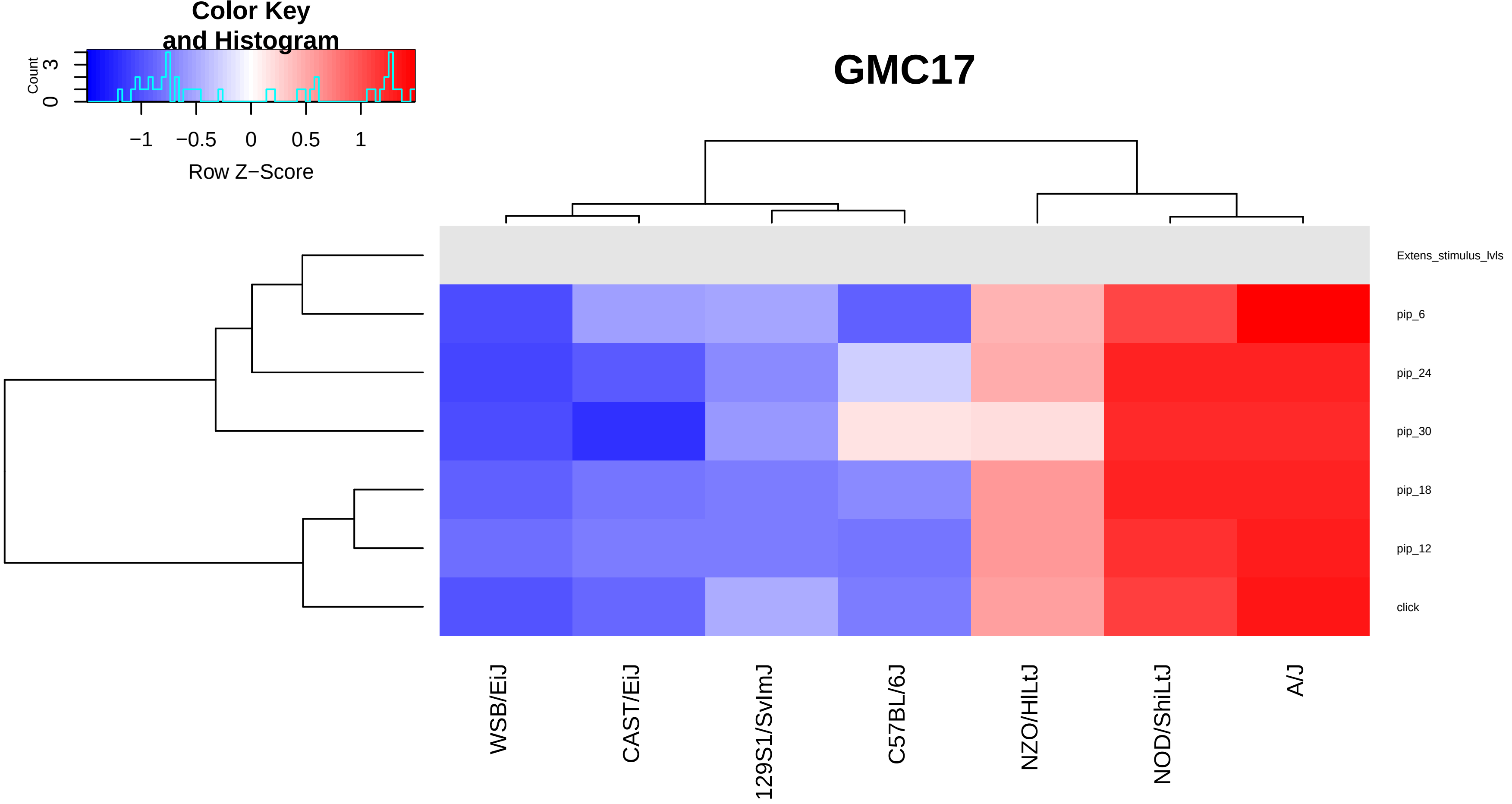

Color Key
and Histogram
Count
0
3
−1
−0.5
0
0.5
1
Row Z−Score
GMC17
Extens_stimulus_lvls
pip_6
pip_24
pip_30
pip_18
pip_12
click
WSB/EiJ
CAST/EiJ
129S1/SvImJ
C57BL/6J
NZO/HlLtJ
NOD/ShiLtJ
A/J

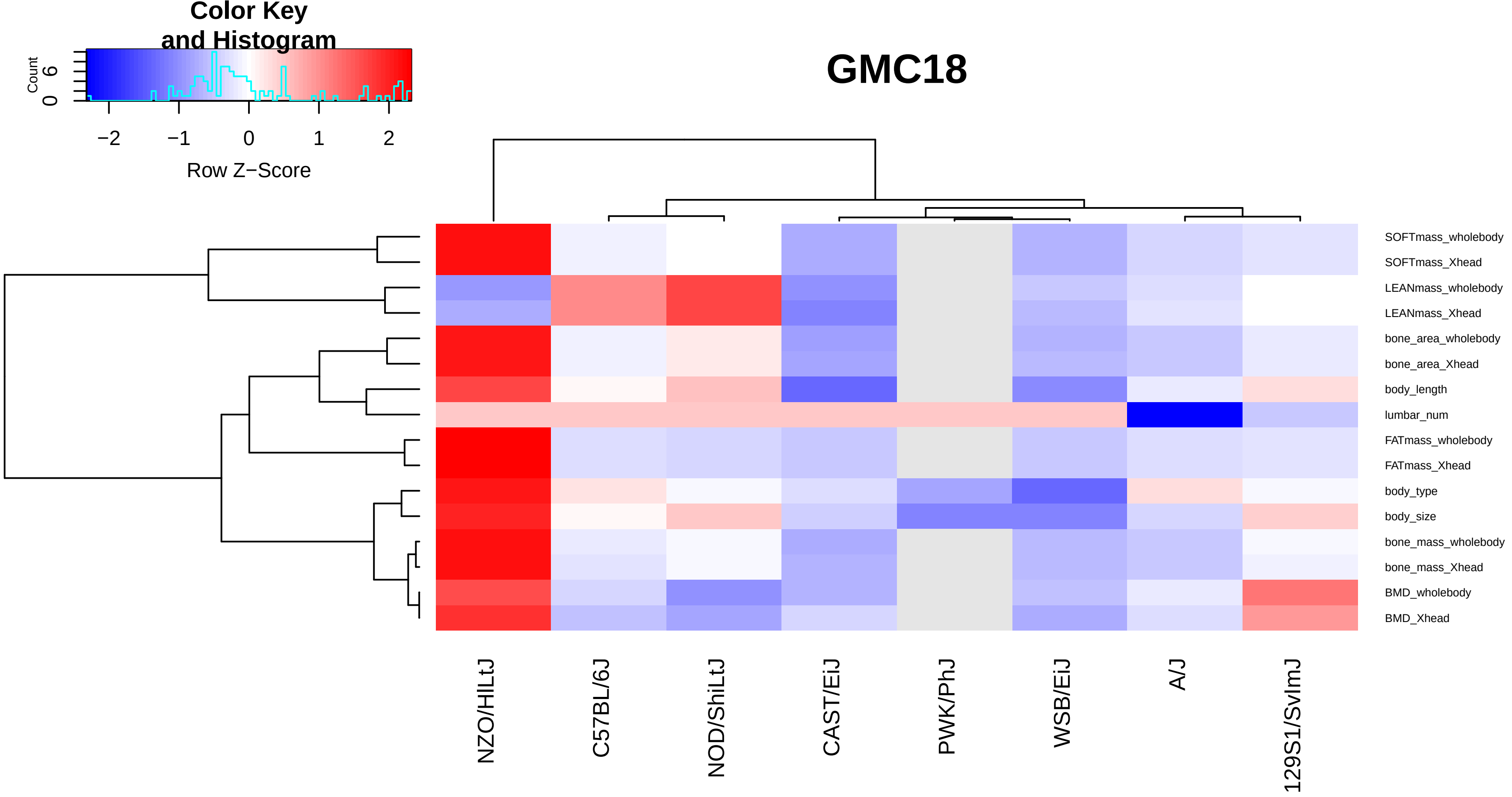

Color Key
and Histogram
Count
0
6
−2
−1
0
1
2
Row Z−Score
GMC18
SOFTmass_wholebody
SOFTmass_Xhead
LEANmass_wholebody
LEANmass_Xhead
bone_area_wholebody
bone_area_Xhead
body_length
lumbar_num
FATmass_wholebody
FATmass_Xhead
body_type
body_size
bone_mass_wholebody
bone_mass_Xhead
BMD_wholebody
BMD_Xhead
NZO/HlLtJ
C57BL/6J
NOD/ShiLtJ
CAST/EiJ
PWK/PhJ
WSB/EiJ
A/J
129S1/SvImJ

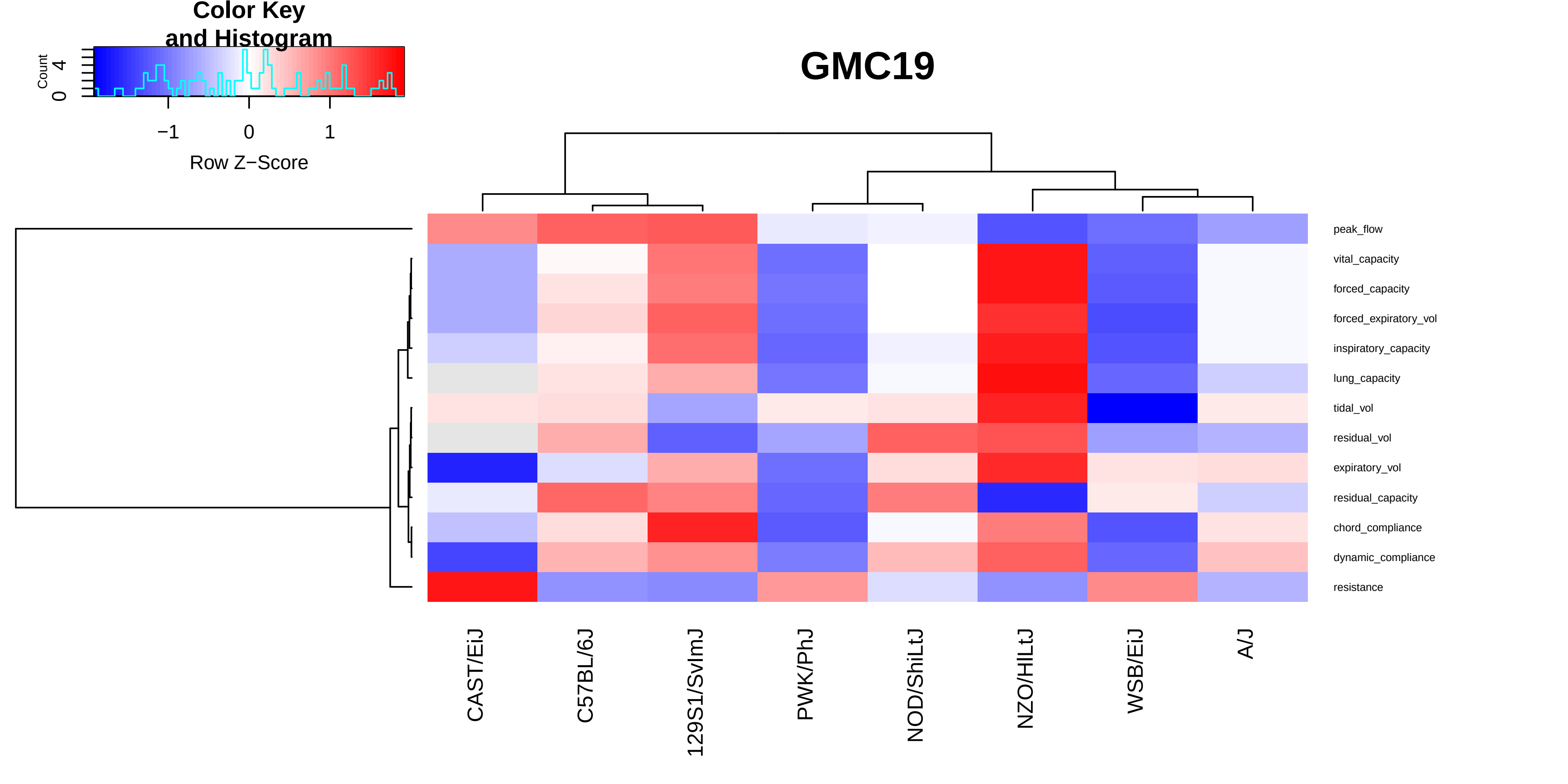
Color Key
and Histogram
Count
0
4
−1
0
1
Row Z−Score
GMC19
peak_flow
vital_capacity
forced_capacity
forced_expiratory_vol
inspiratory_capacity
lung_capacity
tidal_vol
residual_vol
expiratory_vol
residual_capacity
chord_compliance
dynamic_compliance
resistance
CAST/EiJ
C57BL/6J
129S1/SvImJ
PWK/PhJ
NOD/ShiLtJ
NZO/HlLtJ
WSB/EiJ
A/J

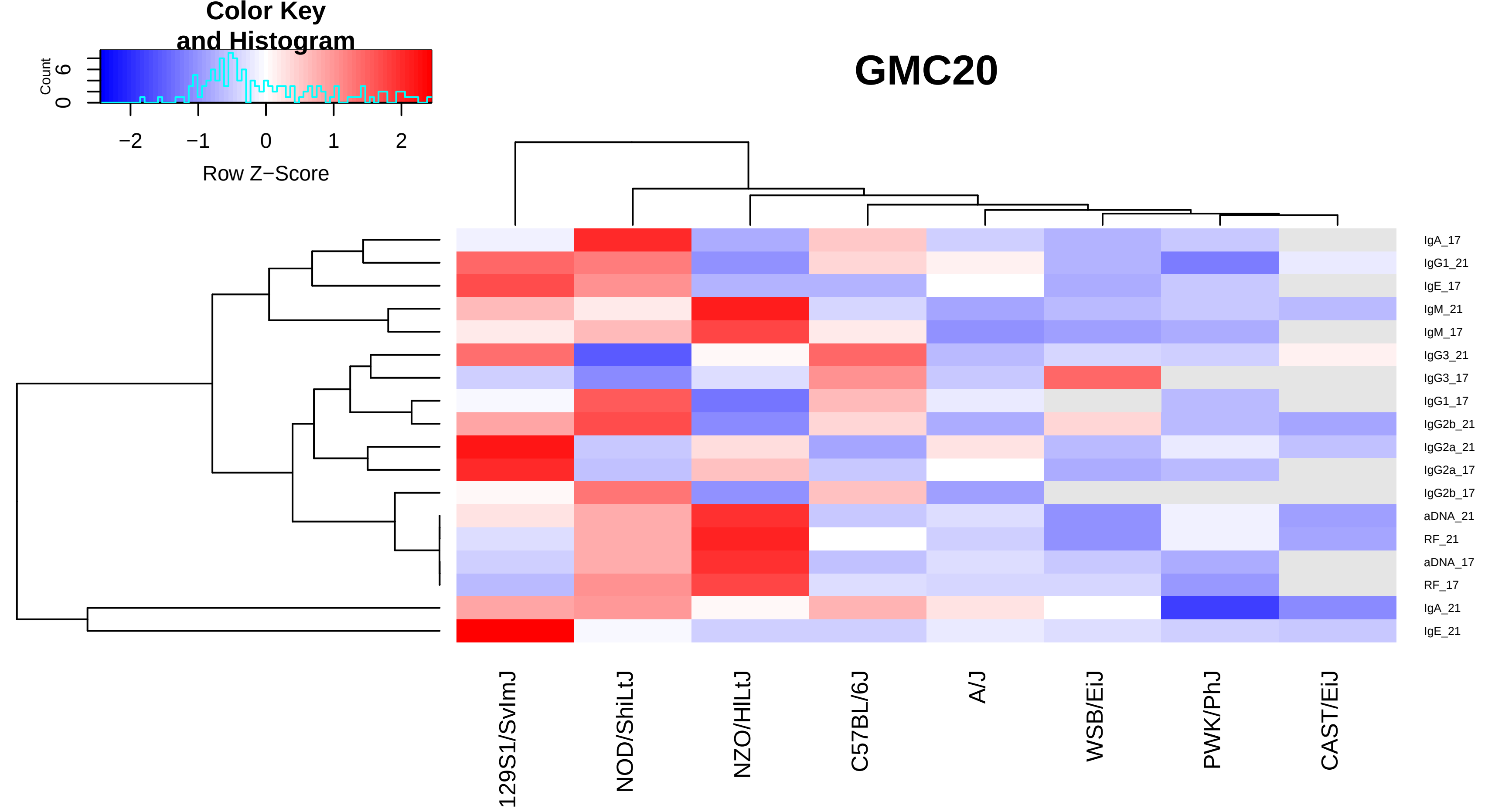
Color Key
and Histogram
Count
0
6
−2
−1
0
1
2
Row Z−Score
GMC20
IgA_17
IgG1_21
IgE_17
IgM_21
IgM_17
IgG3_21
IgG3_17
IgG1_17
IgG2b_21
IgG2a_21
IgG2a_17
IgG2b_17
aDNA_21
RF_21
aDNA_17
RF_17
IgA_21
IgE_21
129S1/SvImJ
NOD/ShiLtJ
NZO/HlLtJ
C57BL/6J
A/J
WSB/EiJ
PWK/PhJ
CAST/EiJ

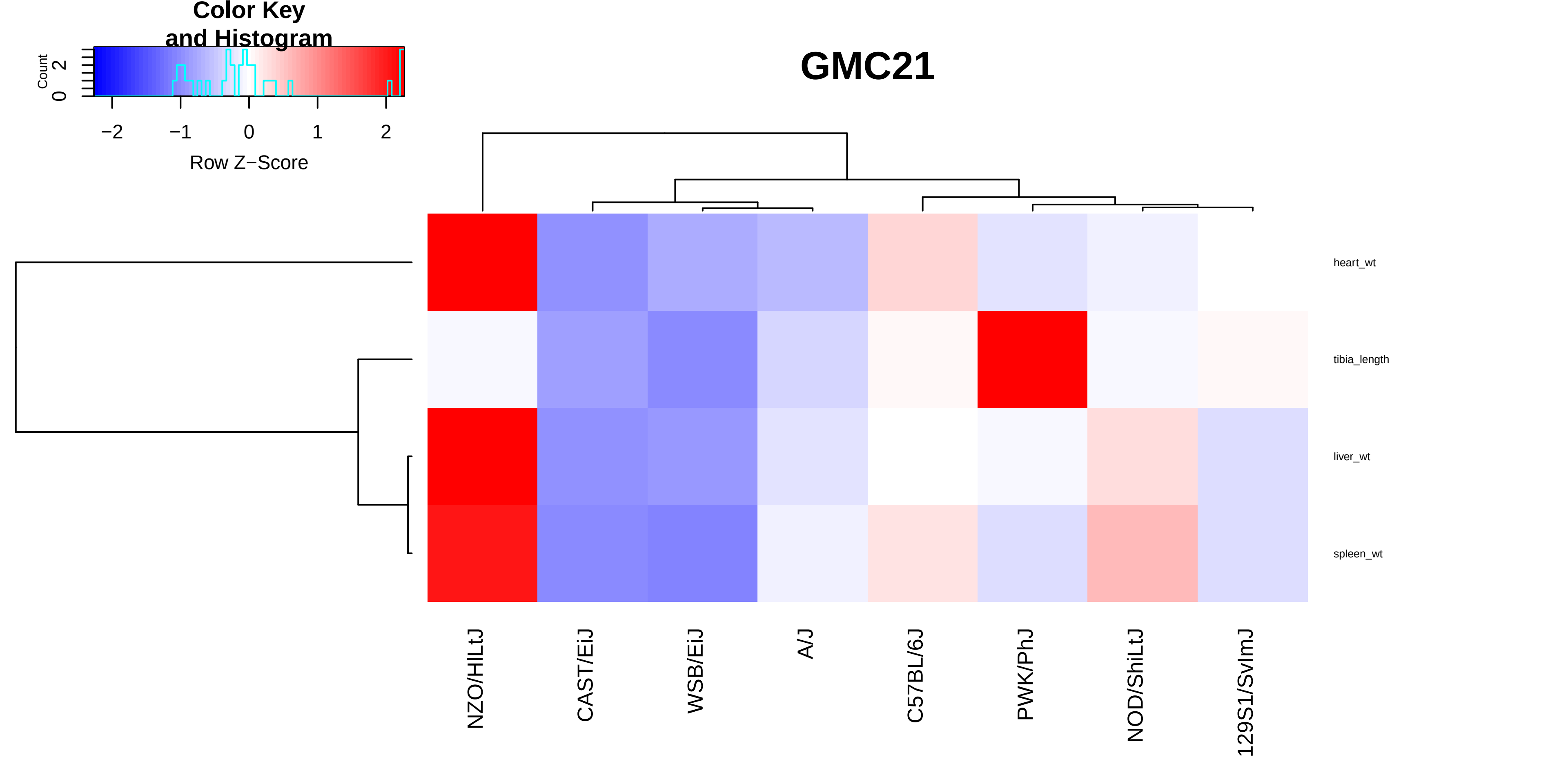

Color Key
and Histogram
Count
0
2
−2
−1
0
1
2
Row Z−Score
GMC21
heart_wt
tibia_length
liver_wt
spleen_wt
NZO/HlLtJ
CAST/EiJ
WSB/EiJ
A/J
C57BL/6J
PWK/PhJ
NOD/ShiLtJ
129S1/SvImJ
