# Supplementary figures and images for: A comprehensive and comparative phenotypic analysis of the collaborative founder strains identifies new and known phenotypes
Source: Mamm Genome. 2020 Feb 14;31(1):30–48. doi: 10.1007/s00335-020-09827-3 (PMC7060152; doi:10.1007/s00335-020-09827-3)

Correlation

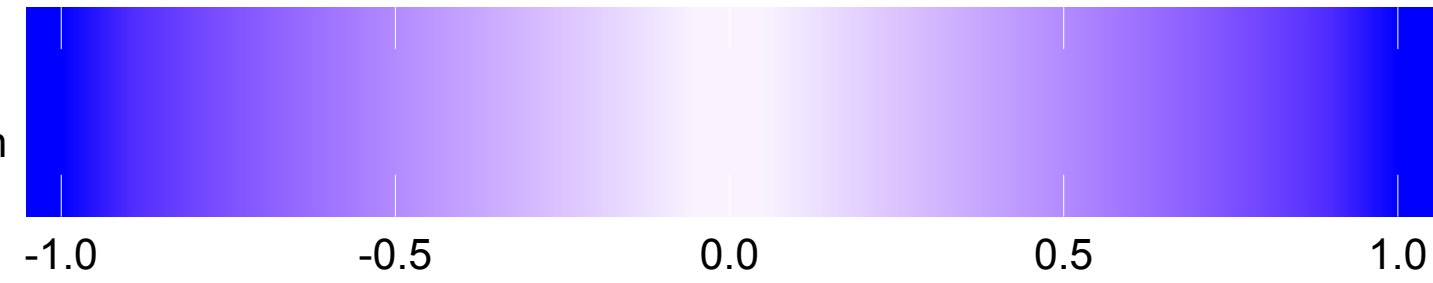

Figure S3

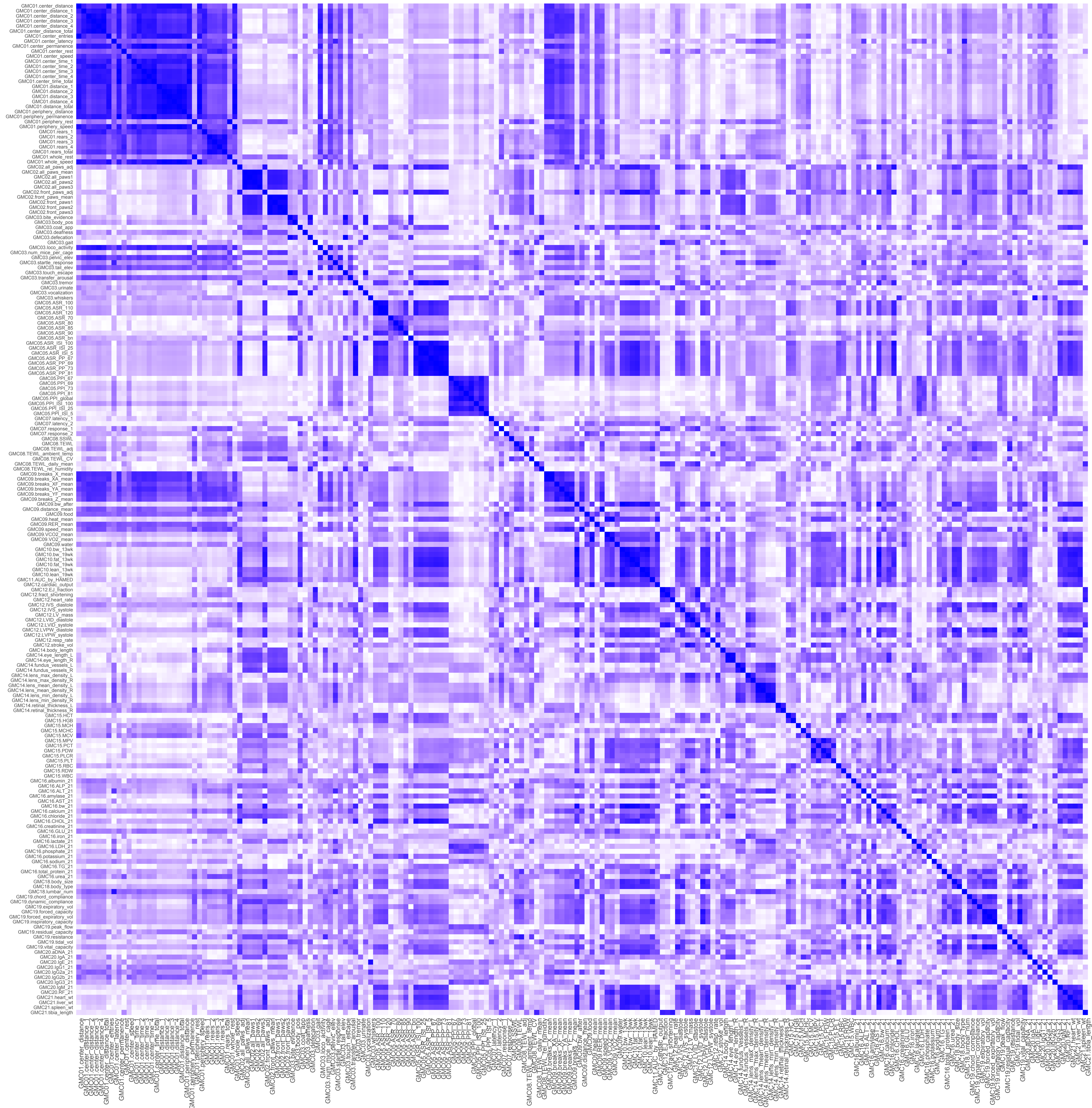

Supplement: Supplementary file 4 — Supplementary file4 (PDF 664 kb) [file 335_2020_9827_MOESM4_ESM.pdf]

### Table S4

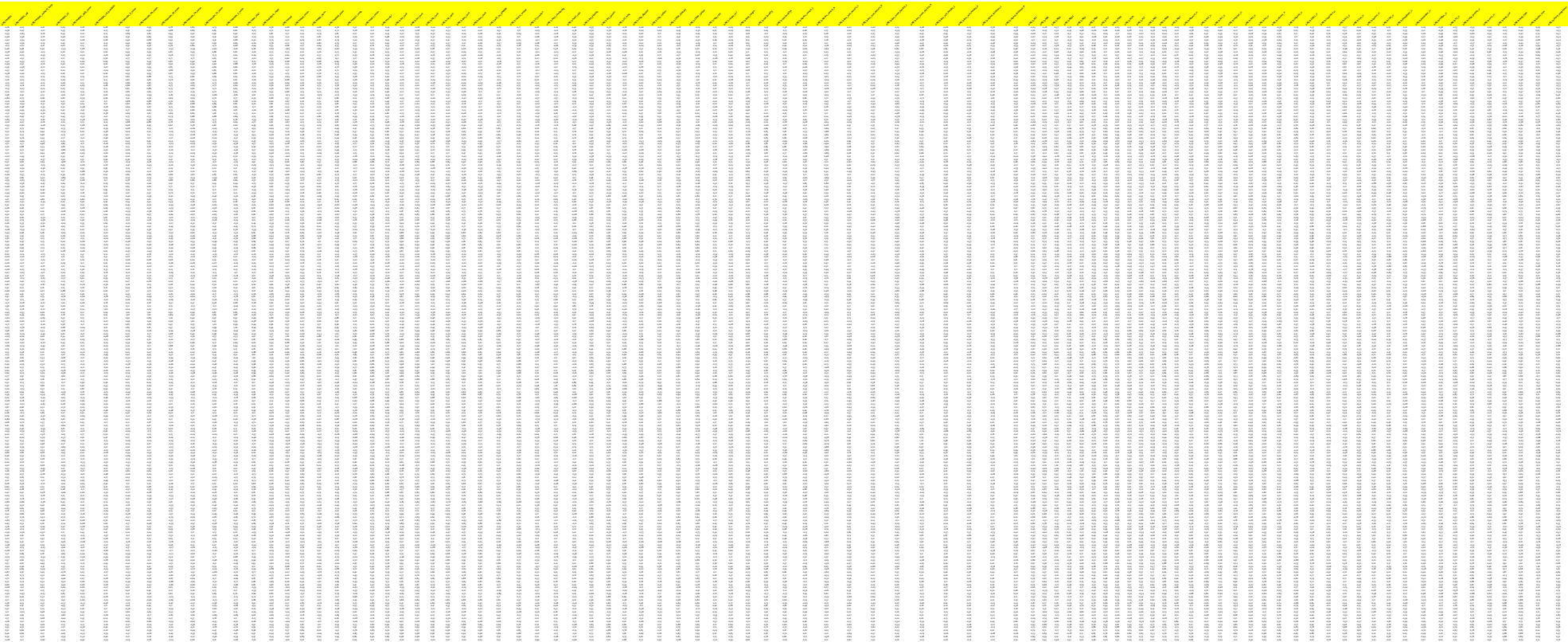

|   |   |   |   |   |   |   |   |   |    |    |    |    |    |    |    |    |    |    |    |    |    |    |    |    |    |    |    |    |    |    |    |    |    |    |    |    |    |    |    |    |    |    |    |    |    |    |    |    |    |    |    |    |    |    |    |    |    |    |    |    |    |    |    |    |    |    |    |    |    |    |    |    |    |    |    |    |    |    |    |    |    |    |    |    |    |    |    |    |    |    |    |    |    |    |    |    |    |    |     |     |     |     |     |     |     |     |     |     |     |     |     |     |     |     |     |     |     |     |     |     |     |     |     |     |     |     |     |     |     |     |     |     |     |     |     |     |     |     |     |     |     |     |     |     |     |     |     |     |     |     |     |     |     |     |     |     |     |     |     |     |     |     |     |     |     |     |     |     |     |     |     |     |     |     |     |     |     |     |     |     |     |     |     |     |     |     |     |     |     |     |     |     |     |     |     |     |     |     |     |     |     |     |     |     |     |     |     |     |     |     |     |     |     |     |     |     |     |     |     |     |     |     |     |     |     |     |     |     |     |     |     |     |     |     |     |     |     |     |     |     |     |     |     |     |     |     |     |     |     |     |     |     |     |     |     |     |     |     |     |     |     |     |     |     |     |     |     |     |     |     |     |     |     |     |     |     |     |     |     |     |     |     |     |     |     |     |     |     |     |     |     |     |     |     |     |     |     |     |     |     |     |     |     |     |     |     |     |     |     |     |     |     |     |     |     |     |     |     |     |     |     |     |     |     |     |     |     |     |     |     |     |     |     |     |     |     |     |     |     |     |     |     |     |     |     |     |     |     |     |     |     |     |     |     |     |     |     |     |     |     |     |     |     |     |     |     |     |     |     |     |     |     |     |     |     |     |     |     |     |     |     |     |     |     |     |     |     |     |     |     |     |     |     |     |     |     |     |     |     |     |     |     |     |     |     |     |     |     |     |     |     |     |     |     |     |     |     |     |     |     |     |     |     |     |     |     |     |     |     |     |     |     |     |     |     |     |     |     |     |     |     |     |     |     |     |     |     |     |     |     |     |     |     |     |     |     |     |     |     |     |     |     |     |     |     |     |     |     |     |     |     |     |     |     |     |     |     |     |     |     |     |     |     |     |     |     |     |     |     |     |     |     |     |     |     |     |     |     |     |     |     |     |     |     |     |     |     |     |     |     |     |     |     |     |     |     |     |     |     |     |     |     |     |     |     |     |     |     |     |     |     |     |     |     |     |     |     |     |     |     |     |     |     |     |     |     |     |     |     |     |     |     |     |     |     |     |     |     |     |     |     |     |     |     |     |     |     |     |     |     |     |     |     |     |     |     |     |     |     |     |     |     |     |     |     |     |     |     |     |     |     |     |     |     |     |     |     |     |     |     |     |     |     |     |     |     |     |     |     |     |     |     |     |     |     |     |     |     |     |     |     |     |     |     |     |     |     |     |     |     |     |     |     |     |     |     |     |     |     |     |     |     |     |     |     |     |     |     |     |     |     |     |     |     |     |     |     |     |     |     |     |     |     |     |     |     |     |     |     |     |     |     |     |     |     |     |     |     |     |     |     |     |     |     |     |     |     |     |     |     |     |     |     |     |     |     |     |     |     |     |     |     |     |     |     |     |     |     |     |     |     |     |     |     |     |     |     |     |     |     |     |     |     |     |     |     |     |     |     |     |     |     |     |     |     |     |     |     |     |     |     |     |     |     |     |     |     |     |     |     |     |     |     |     |     |     |     |     |     |     |     |     |     |     |     |     |     |     |     |     |     |     |     |     |     |     |     |     |     |     |     |     |     |     |     |     |     |     |     |     |     |     |     |     |     |     |     |     |     |     |     |     |     |     |     |     |     |     |     |     |     |     |     |     |     |     |     |     |     |     |     |     |     |     |     |     |     |     |     |     |     |     |     |     |     |     |     |     |     |     |     |     |     |     |     |     |     |     |     |     |     |     |     |     |     |     |     |     |     |     |     |     |     |     |     |     |     |     |     |     |     |     |     |     |     |     |     |     |     |     |     |     |     |     |     |     |     |     |     |     |     |     |     |     |     |     |     |     |     |     |     |     |     |     |     |     |     |     |     |     |     |     |     |     |     |     |     |     |     |     |     |     |     |     |     |     |     |     |     |     |     |     |     |     |     |     |     |     |     |     |     |     |     |     |     |     |     |     |     |     |     |     |     |     |     |     |     |     |     |     |     |     |     |     |     |     |     |     |     |     |     |     |     |     |     |     |     |     |     |     |     |     |     |     |     |     |     |     |     |     |     |     |     |     |     |     |     |     |      |
|---|---|---|---|---|---|---|---|---|----|----|----|----|----|----|----|----|----|----|----|----|----|----|----|----|----|----|----|----|----|----|----|----|----|----|----|----|----|----|----|----|----|----|----|----|----|----|----|----|----|----|----|----|----|----|----|----|----|----|----|----|----|----|----|----|----|----|----|----|----|----|----|----|----|----|----|----|----|----|----|----|----|----|----|----|----|----|----|----|----|----|----|----|----|----|----|----|----|----|-----|-----|-----|-----|-----|-----|-----|-----|-----|-----|-----|-----|-----|-----|-----|-----|-----|-----|-----|-----|-----|-----|-----|-----|-----|-----|-----|-----|-----|-----|-----|-----|-----|-----|-----|-----|-----|-----|-----|-----|-----|-----|-----|-----|-----|-----|-----|-----|-----|-----|-----|-----|-----|-----|-----|-----|-----|-----|-----|-----|-----|-----|-----|-----|-----|-----|-----|-----|-----|-----|-----|-----|-----|-----|-----|-----|-----|-----|-----|-----|-----|-----|-----|-----|-----|-----|-----|-----|-----|-----|-----|-----|-----|-----|-----|-----|-----|-----|-----|-----|-----|-----|-----|-----|-----|-----|-----|-----|-----|-----|-----|-----|-----|-----|-----|-----|-----|-----|-----|-----|-----|-----|-----|-----|-----|-----|-----|-----|-----|-----|-----|-----|-----|-----|-----|-----|-----|-----|-----|-----|-----|-----|-----|-----|-----|-----|-----|-----|-----|-----|-----|-----|-----|-----|-----|-----|-----|-----|-----|-----|-----|-----|-----|-----|-----|-----|-----|-----|-----|-----|-----|-----|-----|-----|-----|-----|-----|-----|-----|-----|-----|-----|-----|-----|-----|-----|-----|-----|-----|-----|-----|-----|-----|-----|-----|-----|-----|-----|-----|-----|-----|-----|-----|-----|-----|-----|-----|-----|-----|-----|-----|-----|-----|-----|-----|-----|-----|-----|-----|-----|-----|-----|-----|-----|-----|-----|-----|-----|-----|-----|-----|-----|-----|-----|-----|-----|-----|-----|-----|-----|-----|-----|-----|-----|-----|-----|-----|-----|-----|-----|-----|-----|-----|-----|-----|-----|-----|-----|-----|-----|-----|-----|-----|-----|-----|-----|-----|-----|-----|-----|-----|-----|-----|-----|-----|-----|-----|-----|-----|-----|-----|-----|-----|-----|-----|-----|-----|-----|-----|-----|-----|-----|-----|-----|-----|-----|-----|-----|-----|-----|-----|-----|-----|-----|-----|-----|-----|-----|-----|-----|-----|-----|-----|-----|-----|-----|-----|-----|-----|-----|-----|-----|-----|-----|-----|-----|-----|-----|-----|-----|-----|-----|-----|-----|-----|-----|-----|-----|-----|-----|-----|-----|-----|-----|-----|-----|-----|-----|-----|-----|-----|-----|-----|-----|-----|-----|-----|-----|-----|-----|-----|-----|-----|-----|-----|-----|-----|-----|-----|-----|-----|-----|-----|-----|-----|-----|-----|-----|-----|-----|-----|-----|-----|-----|-----|-----|-----|-----|-----|-----|-----|-----|-----|-----|-----|-----|-----|-----|-----|-----|-----|-----|-----|-----|-----|-----|-----|-----|-----|-----|-----|-----|-----|-----|-----|-----|-----|-----|-----|-----|-----|-----|-----|-----|-----|-----|-----|-----|-----|-----|-----|-----|-----|-----|-----|-----|-----|-----|-----|-----|-----|-----|-----|-----|-----|-----|-----|-----|-----|-----|-----|-----|-----|-----|-----|-----|-----|-----|-----|-----|-----|-----|-----|-----|-----|-----|-----|-----|-----|-----|-----|-----|-----|-----|-----|-----|-----|-----|-----|-----|-----|-----|-----|-----|-----|-----|-----|-----|-----|-----|-----|-----|-----|-----|-----|-----|-----|-----|-----|-----|-----|-----|-----|-----|-----|-----|-----|-----|-----|-----|-----|-----|-----|-----|-----|-----|-----|-----|-----|-----|-----|-----|-----|-----|-----|-----|-----|-----|-----|-----|-----|-----|-----|-----|-----|-----|-----|-----|-----|-----|-----|-----|-----|-----|-----|-----|-----|-----|-----|-----|-----|-----|-----|-----|-----|-----|-----|-----|-----|-----|-----|-----|-----|-----|-----|-----|-----|-----|-----|-----|-----|-----|-----|-----|-----|-----|-----|-----|-----|-----|-----|-----|-----|-----|-----|-----|-----|-----|-----|-----|-----|-----|-----|-----|-----|-----|-----|-----|-----|-----|-----|-----|-----|-----|-----|-----|-----|-----|-----|-----|-----|-----|-----|-----|-----|-----|-----|-----|-----|-----|-----|-----|-----|-----|-----|-----|-----|-----|-----|-----|-----|-----|-----|-----|-----|-----|-----|-----|-----|-----|-----|-----|-----|-----|-----|-----|-----|-----|-----|-----|-----|-----|-----|-----|-----|-----|-----|-----|-----|-----|-----|-----|-----|-----|-----|-----|-----|-----|-----|-----|-----|-----|-----|-----|-----|-----|-----|-----|-----|-----|-----|-----|-----|-----|-----|-----|-----|-----|-----|-----|-----|-----|-----|-----|-----|-----|-----|-----|-----|-----|-----|-----|-----|-----|-----|-----|-----|-----|-----|-----|-----|-----|-----|-----|-----|-----|-----|-----|-----|-----|-----|-----|-----|-----|-----|-----|-----|-----|-----|-----|-----|-----|-----|-----|-----|-----|-----|-----|-----|-----|-----|-----|-----|-----|-----|-----|-----|-----|-----|-----|-----|-----|-----|-----|-----|-----|-----|-----|-----|-----|-----|-----|-----|-----|-----|-----|-----|-----|-----|-----|-----|-----|-----|-----|-----|-----|-----|-----|-----|-----|-----|-----|-----|-----|-----|-----|-----|-----|-----|-----|-----|-----|-----|-----|-----|-----|-----|-----|-----|-----|-----|-----|-----|-----|-----|-----|-----|-----|-----|-----|-----|-----|-----|-----|-----|-----|-----|-----|-----|-----|-----|-----|-----|-----|-----|-----|-----|-----|-----|-----|-----|-----|-----|-----|-----|-----|-----|-----|-----|-----|-----|-----|-----|-----|-----|-----|-----|-----|-----|-----|-----|-----|-----|-----|-----|-----|-----|-----|-----|-----|-----|-----|-----|-----|-----|-----|-----|-----|-----|-----|-----|-----|-----|-----|-----|-----|-----|-----|-----|-----|-----|-----|-----|-----|-----|-----|-----|-----|-----|-----|-----|-----|-----|-----|-----|-----|-----|-----|-----|-----|------|
| 1 | 2 | 3 | 4 | 5 | 6 | 7 | 8 | 9 | 10 | 11 | 12 | 13 | 14 | 15 | 16 | 17 | 18 | 19 | 20 | 21 | 22 | 23 | 24 | 25 | 26 | 27 | 28 | 29 | 30 | 31 | 32 | 33 | 34 | 35 | 36 | 37 | 38 | 39 | 40 | 41 | 42 | 43 | 44 | 45 | 46 | 47 | 48 | 49 | 50 | 51 | 52 | 53 | 54 | 55 | 56 | 57 | 58 | 59 | 60 | 61 | 62 | 63 | 64 | 65 | 66 | 67 | 68 | 69 | 70 | 71 | 72 | 73 | 74 | 75 | 76 | 77 | 78 | 79 | 80 | 81 | 82 | 83 | 84 | 85 | 86 | 87 | 88 | 89 | 90 | 91 | 92 | 93 | 94 | 95 | 96 | 97 | 98 | 99 | 100 | 101 | 102 | 103 | 104 | 105 | 106 | 107 | 108 | 109 | 110 | 111 | 112 | 113 | 114 | 115 | 116 | 117 | 118 | 119 | 120 | 121 | 122 | 123 | 124 | 125 | 126 | 127 | 128 | 129 | 130 | 131 | 132 | 133 | 134 | 135 | 136 | 137 | 138 | 139 | 140 | 141 | 142 | 143 | 144 | 145 | 146 | 147 | 148 | 149 | 150 | 151 | 152 | 153 | 154 | 155 | 156 | 157 | 158 | 159 | 160 | 161 | 162 | 163 | 164 | 165 | 166 | 167 | 168 | 169 | 170 | 171 | 172 | 173 | 174 | 175 | 176 | 177 | 178 | 179 | 180 | 181 | 182 | 183 | 184 | 185 | 186 | 187 | 188 | 189 | 190 | 191 | 192 | 193 | 194 | 195 | 196 | 197 | 198 | 199 | 200 | 201 | 202 | 203 | 204 | 205 | 206 | 207 | 208 | 209 | 210 | 211 | 212 | 213 | 214 | 215 | 216 | 217 | 218 | 219 | 220 | 221 | 222 | 223 | 224 | 225 | 226 | 227 | 228 | 229 | 230 | 231 | 232 | 233 | 234 | 235 | 236 | 237 | 238 | 239 | 240 | 241 | 242 | 243 | 244 | 245 | 246 | 247 | 248 | 249 | 250 | 251 | 252 | 253 | 254 | 255 | 256 | 257 | 258 | 259 | 260 | 261 | 262 | 263 | 264 | 265 | 266 | 267 | 268 | 269 | 270 | 271 | 272 | 273 | 274 | 275 | 276 | 277 | 278 | 279 | 280 | 281 | 282 | 283 | 284 | 285 | 286 | 287 | 288 | 289 | 290 | 291 | 292 | 293 | 294 | 295 | 296 | 297 | 298 | 299 | 300 | 301 | 302 | 303 | 304 | 305 | 306 | 307 | 308 | 309 | 310 | 311 | 312 | 313 | 314 | 315 | 316 | 317 | 318 | 319 | 320 | 321 | 322 | 323 | 324 | 325 | 326 | 327 | 328 | 329 | 330 | 331 | 332 | 333 | 334 | 335 | 336 | 337 | 338 | 339 | 340 | 341 | 342 | 343 | 344 | 345 | 346 | 347 | 348 | 349 | 350 | 351 | 352 | 353 | 354 | 355 | 356 | 357 | 358 | 359 | 360 | 361 | 362 | 363 | 364 | 365 | 366 | 367 | 368 | 369 | 370 | 371 | 372 | 373 | 374 | 375 | 376 | 377 | 378 | 379 | 380 | 381 | 382 | 383 | 384 | 385 | 386 | 387 | 388 | 389 | 390 | 391 | 392 | 393 | 394 | 395 | 396 | 397 | 398 | 399 | 400 | 401 | 402 | 403 | 404 | 405 | 406 | 407 | 408 | 409 | 410 | 411 | 412 | 413 | 414 | 415 | 416 | 417 | 418 | 419 | 420 | 421 | 422 | 423 | 424 | 425 | 426 | 427 | 428 | 429 | 430 | 431 | 432 | 433 | 434 | 435 | 436 | 437 | 438 | 439 | 440 | 441 | 442 | 443 | 444 | 445 | 446 | 447 | 448 | 449 | 450 | 451 | 452 | 453 | 454 | 455 | 456 | 457 | 458 | 459 | 460 | 461 | 462 | 463 | 464 | 465 | 466 | 467 | 468 | 469 | 470 | 471 | 472 | 473 | 474 | 475 | 476 | 477 | 478 | 479 | 480 | 481 | 482 | 483 | 484 | 485 | 486 | 487 | 488 | 489 | 490 | 491 | 492 | 493 | 494 | 495 | 496 | 497 | 498 | 499 | 500 | 501 | 502 | 503 | 504 | 505 | 506 | 507 | 508 | 509 | 510 | 511 | 512 | 513 | 514 | 515 | 516 | 517 | 518 | 519 | 520 | 521 | 522 | 523 | 524 | 525 | 526 | 527 | 528 | 529 | 530 | 531 | 532 | 533 | 534 | 535 | 536 | 537 | 538 | 539 | 540 | 541 | 542 | 543 | 544 | 545 | 546 | 547 | 548 | 549 | 550 | 551 | 552 | 553 | 554 | 555 | 556 | 557 | 558 | 559 | 560 | 561 | 562 | 563 | 564 | 565 | 566 | 567 | 568 | 569 | 570 | 571 | 572 | 573 | 574 | 575 | 576 | 577 | 578 | 579 | 580 | 581 | 582 | 583 | 584 | 585 | 586 | 587 | 588 | 589 | 590 | 591 | 592 | 593 | 594 | 595 | 596 | 597 | 598 | 599 | 600 | 601 | 602 | 603 | 604 | 605 | 606 | 607 | 608 | 609 | 610 | 611 | 612 | 613 | 614 | 615 | 616 | 617 | 618 | 619 | 620 | 621 | 622 | 623 | 624 | 625 | 626 | 627 | 628 | 629 | 630 | 631 | 632 | 633 | 634 | 635 | 636 | 637 | 638 | 639 | 640 | 641 | 642 | 643 | 644 | 645 | 646 | 647 | 648 | 649 | 650 | 651 | 652 | 653 | 654 | 655 | 656 | 657 | 658 | 659 | 660 | 661 | 662 | 663 | 664 | 665 | 666 | 667 | 668 | 669 | 670 | 671 | 672 | 673 | 674 | 675 | 676 | 677 | 678 | 679 | 680 | 681 | 682 | 683 | 684 | 685 | 686 | 687 | 688 | 689 | 690 | 691 | 692 | 693 | 694 | 695 | 696 | 697 | 698 | 699 | 700 | 701 | 702 | 703 | 704 | 705 | 706 | 707 | 708 | 709 | 710 | 711 | 712 | 713 | 714 | 715 | 716 | 717 | 718 | 719 | 720 | 721 | 722 | 723 | 724 | 725 | 726 | 727 | 728 | 729 | 730 | 731 | 732 | 733 | 734 | 735 | 736 | 737 | 738 | 739 | 740 | 741 | 742 | 743 | 744 | 745 | 746 | 747 | 748 | 749 | 750 | 751 | 752 | 753 | 754 | 755 | 756 | 757 | 758 | 759 | 760 | 761 | 762 | 763 | 764 | 765 | 766 | 767 | 768 | 769 | 770 | 771 | 772 | 773 | 774 | 775 | 776 | 777 | 778 | 779 | 780 | 781 | 782 | 783 | 784 | 785 | 786 | 787 | 788 | 789 | 790 | 791 | 792 | 793 | 794 | 795 | 796 | 797 | 798 | 799 | 800 | 801 | 802 | 803 | 804 | 805 | 806 | 807 | 808 | 809 | 810 | 811 | 812 | 813 | 814 | 815 | 816 | 817 | 818 | 819 | 820 | 821 | 822 | 823 | 824 | 825 | 826 | 827 | 828 | 829 | 830 | 831 | 832 | 833 | 834 | 835 | 836 | 837 | 838 | 839 | 840 | 841 | 842 | 843 | 844 | 845 | 846 | 847 | 848 | 849 | 850 | 851 | 852 | 853 | 854 | 855 | 856 | 857 | 858 | 859 | 860 | 861 | 862 | 863 | 864 | 865 | 866 | 867 | 868 | 869 | 870 | 871 | 872 | 873 | 874 | 875 | 876 | 877 | 878 | 879 | 880 | 881 | 882 | 883 | 884 | 885 | 886 | 887 | 888 | 889 | 890 | 891 | 892 | 893 | 894 | 895 | 896 | 897 | 898 | 899 | 900 | 901 | 902 | 903 | 904 | 905 | 906 | 907 | 908 | 909 | 910 | 911 | 912 | 913 | 914 | 915 | 916 | 917 | 918 | 919 | 920 | 921 | 922 | 923 | 924 | 925 | 926 | 927 | 928 | 929 | 930 | 931 | 932 | 933 | 934 | 935 | 936 | 937 | 938 | 939 | 940 | 941 | 942 | 943 | 944 | 945 | 946 | 947 | 948 | 949 | 950 | 951 | 952 | 953 | 954 | 955 | 956 | 957 | 958 | 959 | 960 | 961 | 962 | 963 | 964 | 965 | 966 | 967 | 968 | 969 | 970 | 971 | 972 | 973 | 974 | 975 | 976 | 977 | 978 | 979 | 980 | 981 | 982 | 983 | 984 | 985 | 986 | 987 | 988 | 989 | 990 | 991 | 992 | 993 | 994 | 995 | 996 | 997 | 998 | 999 | 1000 |
|---|---|---|---|---|---|---|---|---|----|----|----|----|----|----|----|----|----|----|----|----|----|----|----|----|----|----|----|----|----|----|----|----|----|----|----|----|----|----|----|----|----|----|----|----|----|----|----|----|----|----|----|----|----|----|----|----|----|----|----|----|----|----|----|----|----|----|----|----|----|----|----|----|----|----|----|----|----|----|----|----|----|----|----|----|----|----|----|----|----|----|----|----|----|----|----|----|----|----|-----|-----|-----|-----|-----|-----|-----|-----|-----|-----|-----|-----|-----|-----|-----|-----|-----|-----|-----|-----|-----|-----|-----|-----|-----|-----|-----|-----|-----|-----|-----|-----|-----|-----|-----|-----|-----|-----|-----|-----|-----|-----|-----|-----|-----|-----|-----|-----|-----|-----|-----|-----|-----|-----|-----|-----|-----|-----|-----|-----|-----|-----|-----|-----|-----|-----|-----|-----|-----|-----|-----|-----|-----|-----|-----|-----|-----|-----|-----|-----|-----|-----|-----|-----|-----|-----|-----|-----|-----|-----|-----|-----|-----|-----|-----|-----|-----|-----|-----|-----|-----|-----|-----|-----|-----|-----|-----|-----|-----|-----|-----|-----|-----|-----|-----|-----|-----|-----|-----|-----|-----|-----|-----|-----|-----|-----|-----|-----|-----|-----|-----|-----|-----|-----|-----|-----|-----|-----|-----|-----|-----|-----|-----|-----|-----|-----|-----|-----|-----|-----|-----|-----|-----|-----|-----|-----|-----|-----|-----|-----|-----|-----|-----|-----|-----|-----|-----|-----|-----|-----|-----|-----|-----|-----|-----|-----|-----|-----|-----|-----|-----|-----|-----|-----|-----|-----|-----|-----|-----|-----|-----|-----|-----|-----|-----|-----|-----|-----|-----|-----|-----|-----|-----|-----|-----|-----|-----|-----|-----|-----|-----|-----|-----|-----|-----|-----|-----|-----|-----|-----|-----|-----|-----|-----|-----|-----|-----|-----|-----|-----|-----|-----|-----|-----|-----|-----|-----|-----|-----|-----|-----|-----|-----|-----|-----|-----|-----|-----|-----|-----|-----|-----|-----|-----|-----|-----|-----|-----|-----|-----|-----|-----|-----|-----|-----|-----|-----|-----|-----|-----|-----|-----|-----|-----|-----|-----|-----|-----|-----|-----|-----|-----|-----|-----|-----|-----|-----|-----|-----|-----|-----|-----|-----|-----|-----|-----|-----|-----|-----|-----|-----|-----|-----|-----|-----|-----|-----|-----|-----|-----|-----|-----|-----|-----|-----|-----|-----|-----|-----|-----|-----|-----|-----|-----|-----|-----|-----|-----|-----|-----|-----|-----|-----|-----|-----|-----|-----|-----|-----|-----|-----|-----|-----|-----|-----|-----|-----|-----|-----|-----|-----|-----|-----|-----|-----|-----|-----|-----|-----|-----|-----|-----|-----|-----|-----|-----|-----|-----|-----|-----|-----|-----|-----|-----|-----|-----|-----|-----|-----|-----|-----|-----|-----|-----|-----|-----|-----|-----|-----|-----|-----|-----|-----|-----|-----|-----|-----|-----|-----|-----|-----|-----|-----|-----|-----|-----|-----|-----|-----|-----|-----|-----|-----|-----|-----|-----|-----|-----|-----|-----|-----|-----|-----|-----|-----|-----|-----|-----|-----|-----|-----|-----|-----|-----|-----|-----|-----|-----|-----|-----|-----|-----|-----|-----|-----|-----|-----|-----|-----|-----|-----|-----|-----|-----|-----|-----|-----|-----|-----|-----|-----|-----|-----|-----|-----|-----|-----|-----|-----|-----|-----|-----|-----|-----|-----|-----|-----|-----|-----|-----|-----|-----|-----|-----|-----|-----|-----|-----|-----|-----|-----|-----|-----|-----|-----|-----|-----|-----|-----|-----|-----|-----|-----|-----|-----|-----|-----|-----|-----|-----|-----|-----|-----|-----|-----|-----|-----|-----|-----|-----|-----|-----|-----|-----|-----|-----|-----|-----|-----|-----|-----|-----|-----|-----|-----|-----|-----|-----|-----|-----|-----|-----|-----|-----|-----|-----|-----|-----|-----|-----|-----|-----|-----|-----|-----|-----|-----|-----|-----|-----|-----|-----|-----|-----|-----|-----|-----|-----|-----|-----|-----|-----|-----|-----|-----|-----|-----|-----|-----|-----|-----|-----|-----|-----|-----|-----|-----|-----|-----|-----|-----|-----|-----|-----|-----|-----|-----|-----|-----|-----|-----|-----|-----|-----|-----|-----|-----|-----|-----|-----|-----|-----|-----|-----|-----|-----|-----|-----|-----|-----|-----|-----|-----|-----|-----|-----|-----|-----|-----|-----|-----|-----|-----|-----|-----|-----|-----|-----|-----|-----|-----|-----|-----|-----|-----|-----|-----|-----|-----|-----|-----|-----|-----|-----|-----|-----|-----|-----|-----|-----|-----|-----|-----|-----|-----|-----|-----|-----|-----|-----|-----|-----|-----|-----|-----|-----|-----|-----|-----|-----|-----|-----|-----|-----|-----|-----|-----|-----|-----|-----|-----|-----|-----|-----|-----|-----|-----|-----|-----|-----|-----|-----|-----|-----|-----|-----|-----|-----|-----|-----|-----|-----|-----|-----|-----|-----|-----|-----|-----|-----|-----|-----|-----|-----|-----|-----|-----|-----|-----|-----|-----|-----|-----|-----|-----|-----|-----|-----|-----|-----|-----|-----|-----|-----|-----|-----|-----|-----|-----|-----|-----|-----|-----|-----|-----|-----|-----|-----|-----|-----|-----|-----|-----|-----|-----|-----|-----|-----|-----|-----|-----|-----|-----|-----|-----|-----|-----|-----|-----|-----|-----|-----|-----|-----|-----|-----|-----|-----|-----|-----|-----|-----|-----|-----|-----|-----|-----|-----|-----|-----|-----|-----|-----|-----|-----|-----|-----|-----|-----|-----|-----|-----|-----|-----|-----|-----|-----|-----|-----|-----|-----|-----|-----|-----|-----|-----|-----|-----|-----|-----|-----|-----|-----|-----|-----|-----|-----|-----|-----|-----|-----|-----|-----|-----|-----|-----|-----|-----|-----|-----|-----|-----|-----|-----|-----|-----|-----|-----|-----|-----|-----|-----|-----|-----|-----|-----|-----|-----|-----|-----|-----|-----|-----|-----|-----|-----|-----|-----|-----|-----|-----|-----|-----|-----|-----|-----|-----|-----|-----|-----|-----|-----|-----|-----|-----|-----|-----|-----|-----|-----|------|

Supplement: Supplementary file 9 — Supplementary file9 (PDF 1125 kb) [file 335_2020_9827_MOESM9_ESM.pdf]
