# Supplementary material for: A comprehensive and comparative phenotypic analysis of the collaborative founder strains identifies new and known phenotypes
Source: Mamm Genome. 2020 Feb 14;31(1):30–48. doi: 10.1007/s00335-020-09827-3 (PMC7060152; doi:10.1007/s00335-020-09827-3)
Supplement: Supplementary file 5 — Supplementary file5 (PDF 777 kb) [file 335_2020_9827_MOESM5_ESM.pdf]

Procedure: GMC01
Parameter: center_distance

Figure S4

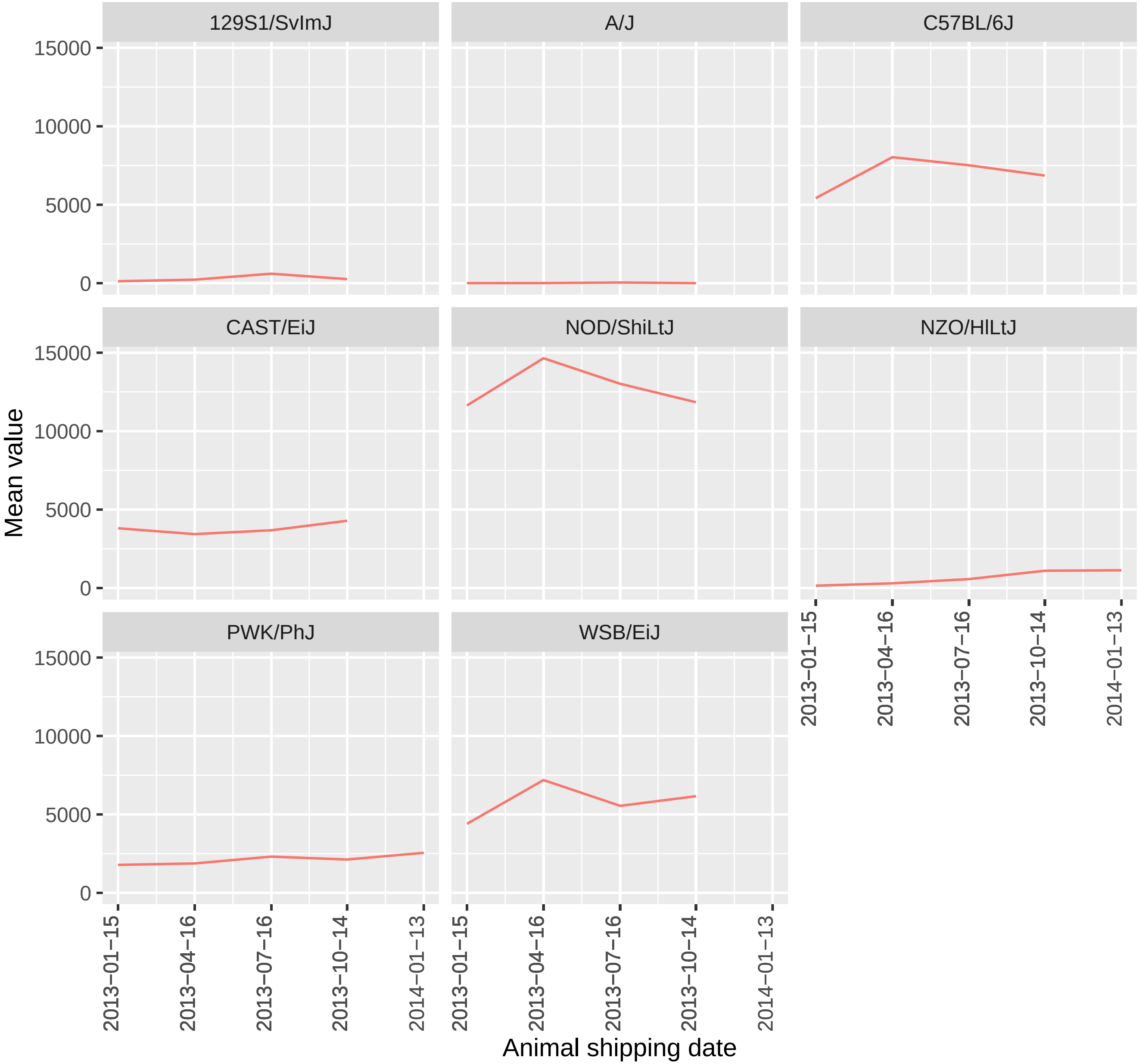

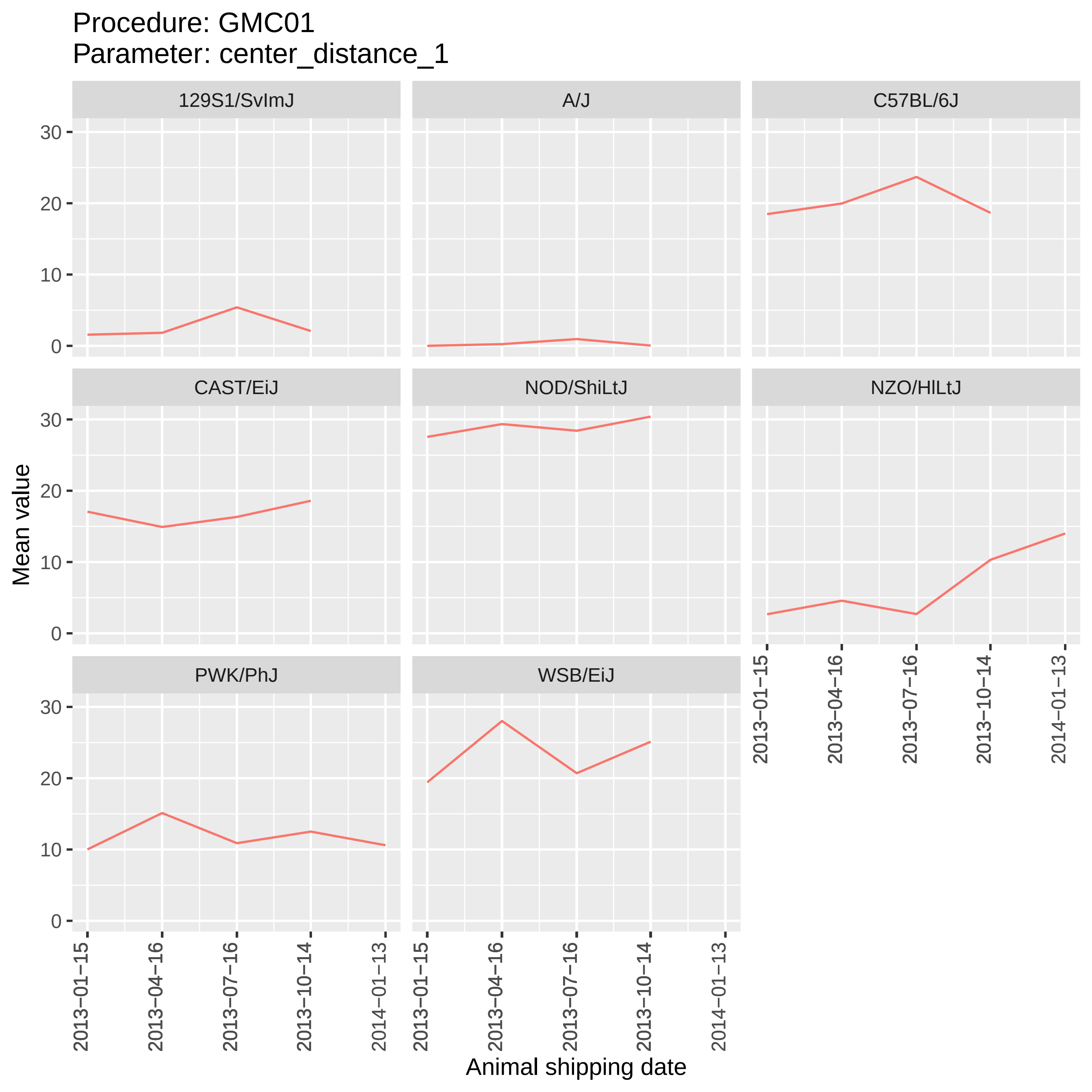

Procedure: GMC01
Parameter: center_distance_1
129S1/SvImJ
A/J
C57BL/6J
CAST/EiJ
NOD/ShiLtJ
NZO/HlLtJ
PWK/PhJ
WSB/EiJ
Mean value
0
10
20
30
2013−01−15
2013−04−16
2013−07−16
2013−10−14
2014−01−13
Animal shipping date

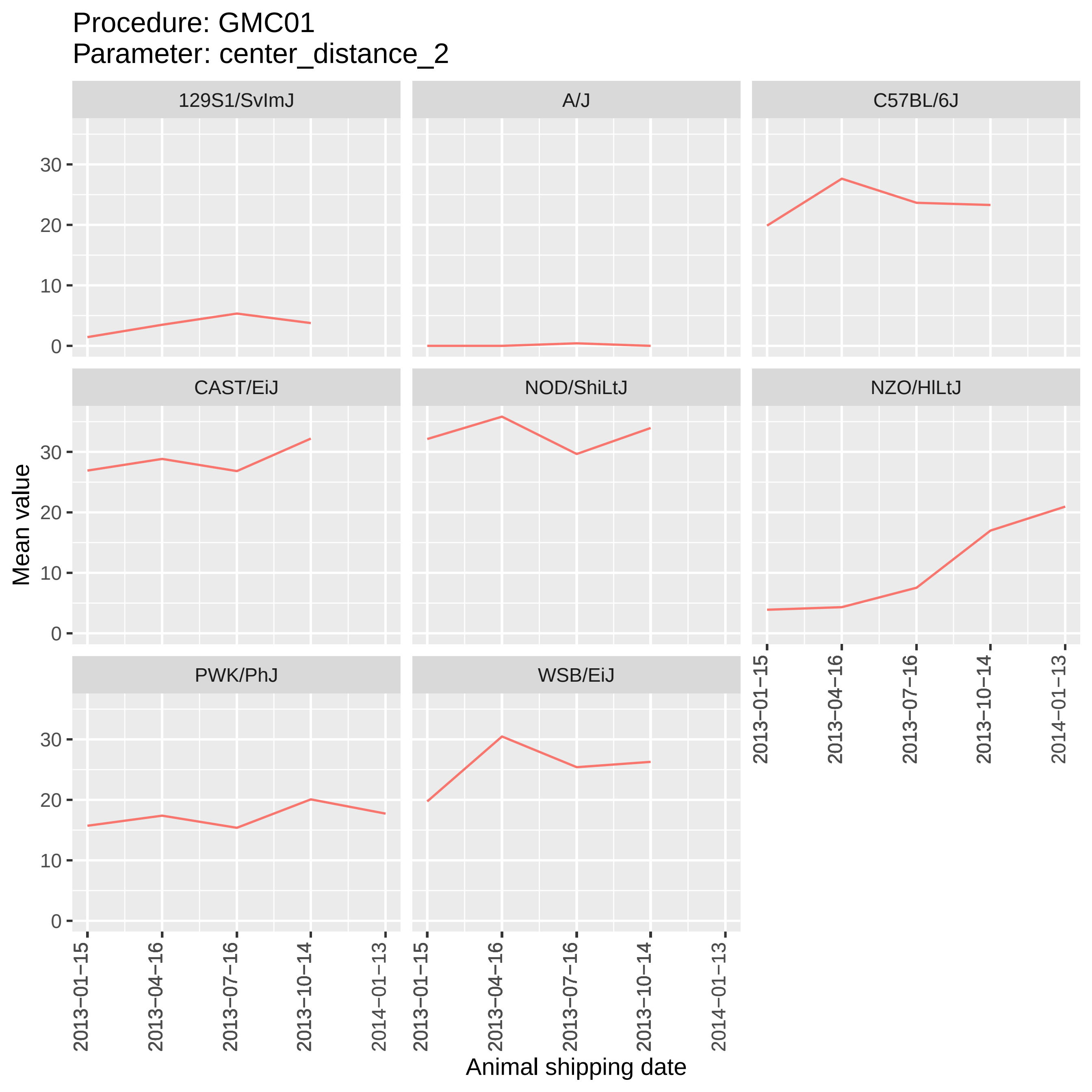

Procedure: GMC01
Parameter: center_distance_2
129S1/SvImJ
A/J
C57BL/6J
CAST/EiJ
NOD/ShiLtJ
NZO/HlLtJ
PWK/PhJ
WSB/EiJ
Mean value
0
10
20
30
2013−01−15
2013−04−16
2013−07−16
2013−10−14
2014−01−13
Animal shipping date

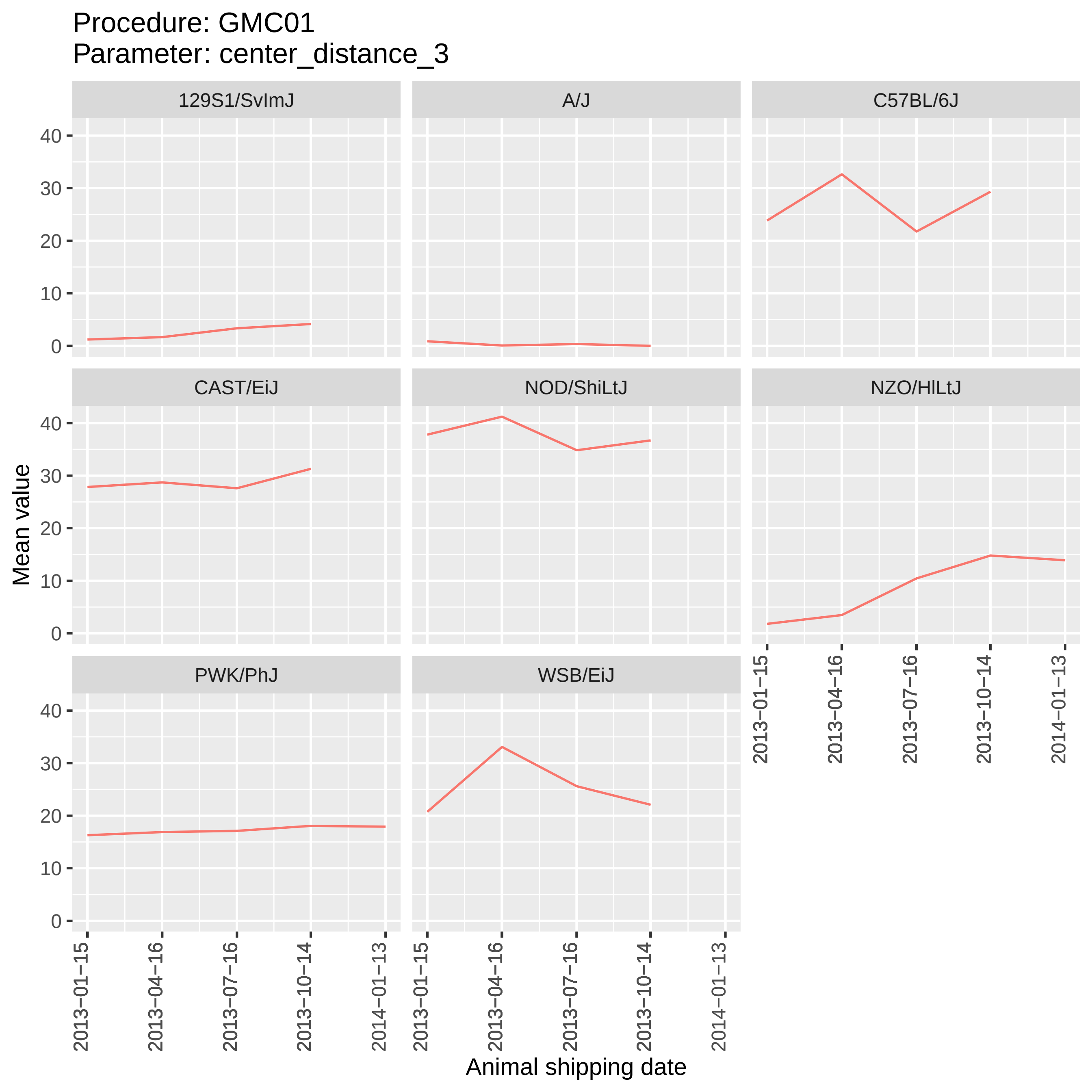

Procedure: GMC01
Parameter: center_distance_3
129S1/SvImJ
A/J
C57BL/6J
CAST/EiJ
NOD/ShiLtJ
NZO/HlLtJ
PWK/PhJ
WSB/EiJ
Mean value
0
10
20
30
40
2013−01−15
2013−04−16
2013−07−16
2013−10−14
2014−01−13
Animal shipping date

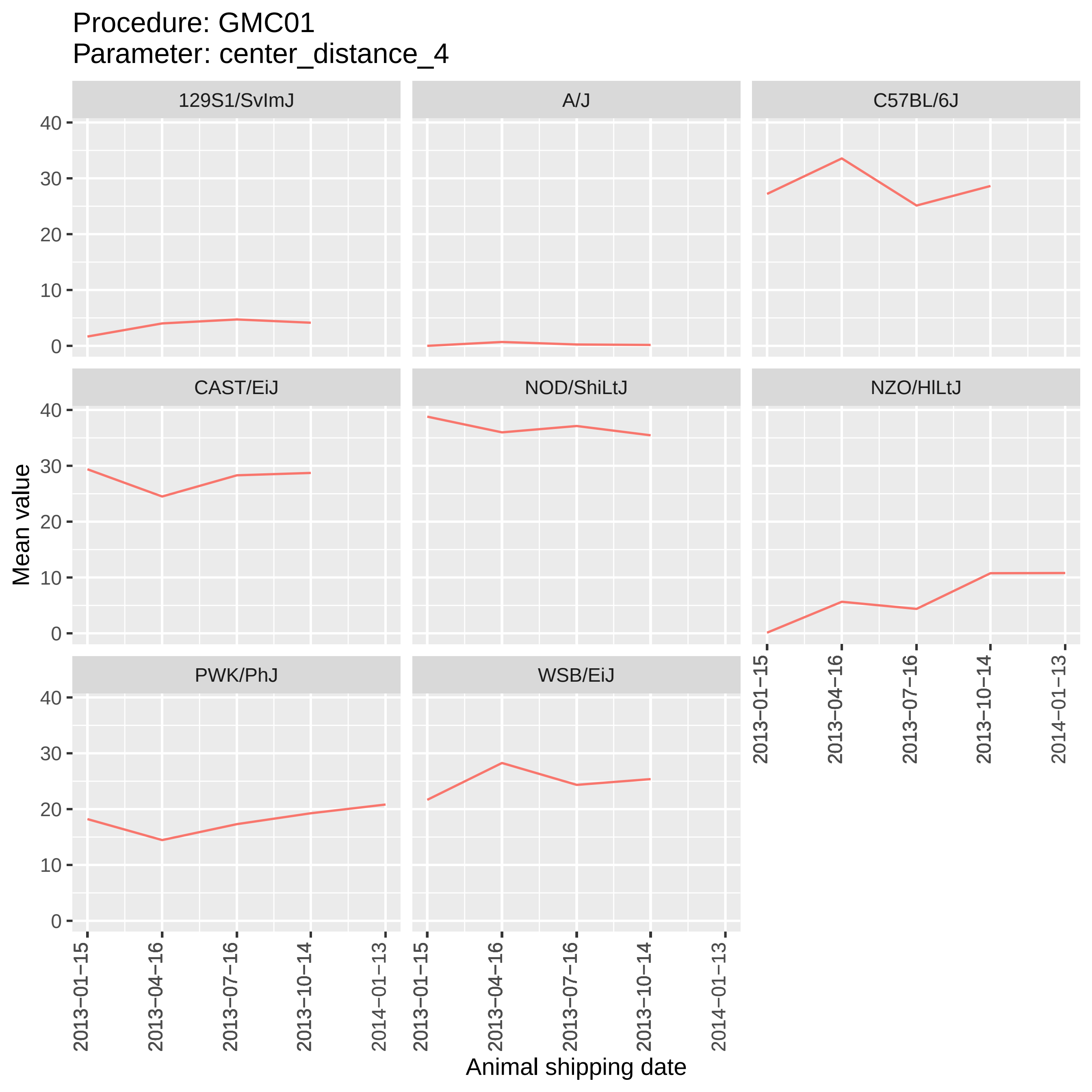

Procedure: GMC01
Parameter: center_distance_4
129S1/SvImJ
A/J
C57BL/6J
CAST/EiJ
NOD/ShiLtJ
NZO/HlLtJ
PWK/PhJ
WSB/EiJ
Mean value
0
10
20
30
40
2013−01−15
2013−04−16
2013−07−16
2013−10−14
2014−01−13
Animal shipping date

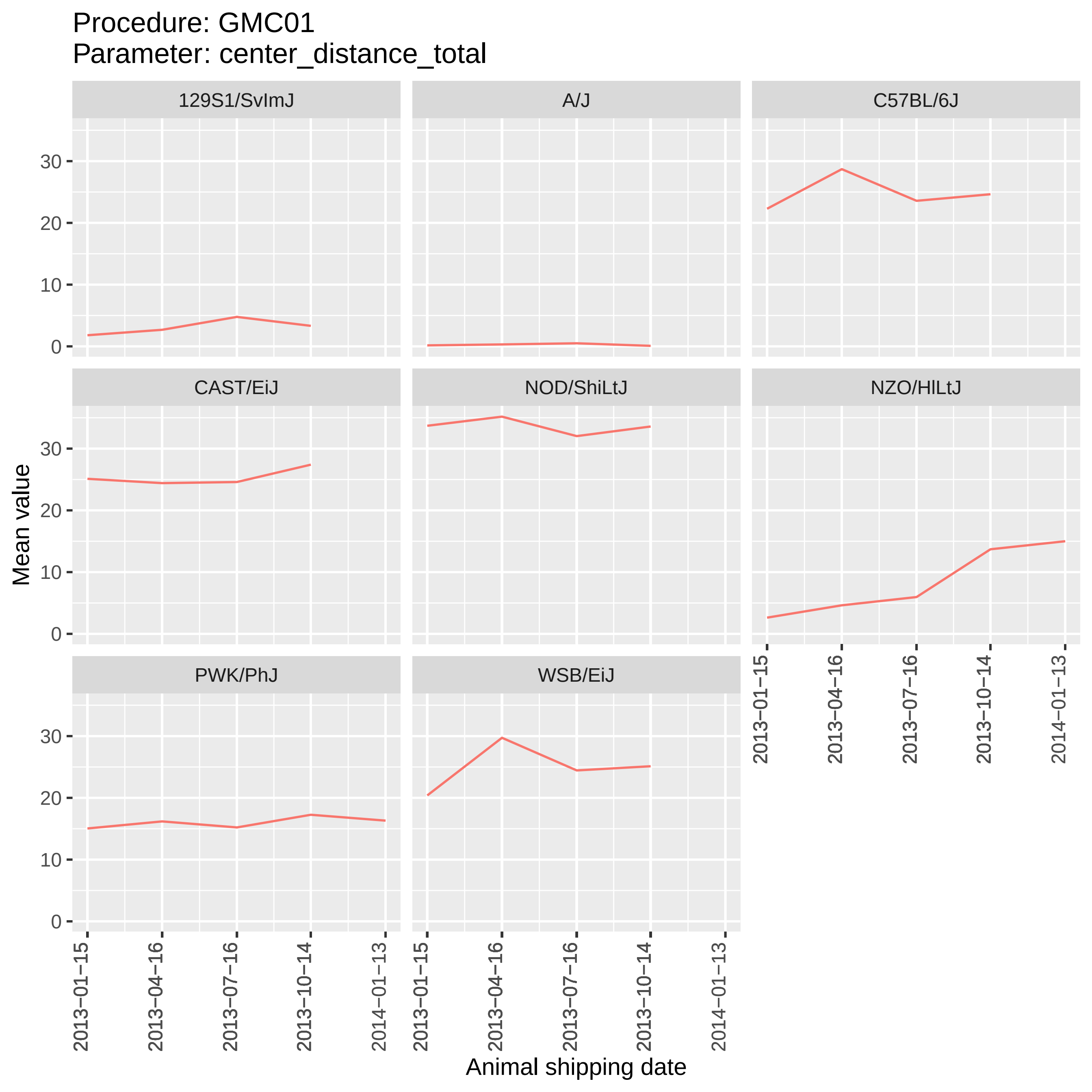
Procedure: GMC01
Parameter: center_distance_total
129S1/SvImJ
A/J
C57BL/6J
CAST/EiJ
NOD/ShiLtJ
NZO/HlLtJ
PWK/PhJ
WSB/EiJ
Mean value
0
10
20
30
2013−01−15
2013−04−16
2013−07−16
2013−10−14
2014−01−13
Animal shipping date

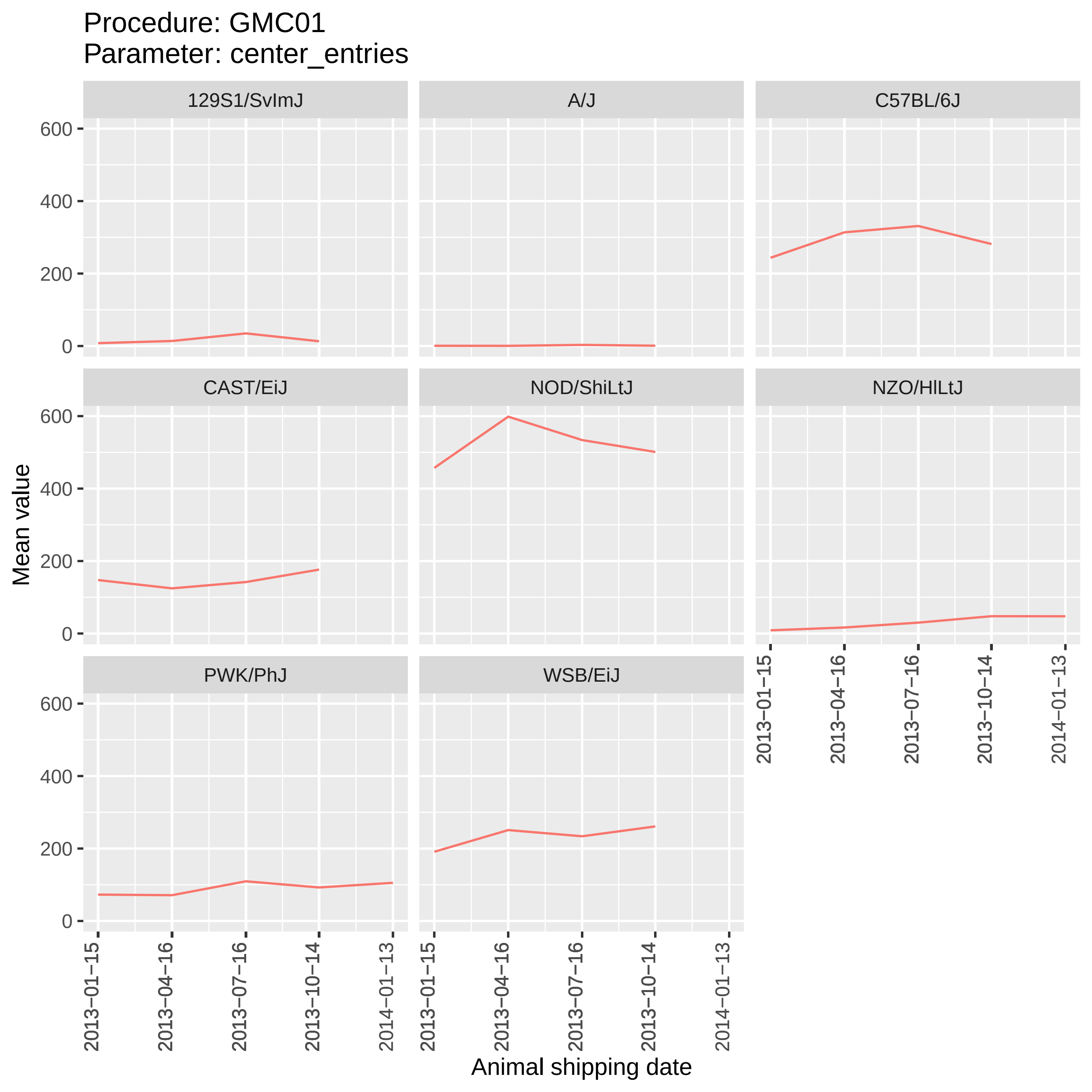

Procedure: GMC01
Parameter: center_entries
129S1/SvImJ
A/J
C57BL/6J
CAST/EiJ
NOD/ShiLtJ
NZO/HlLtJ
PWK/PhJ
WSB/EiJ
Mean value
0
200
400
600
2013−01−15
2013−04−16
2013−07−16
2013−10−14
2014−01−13
Animal shipping date

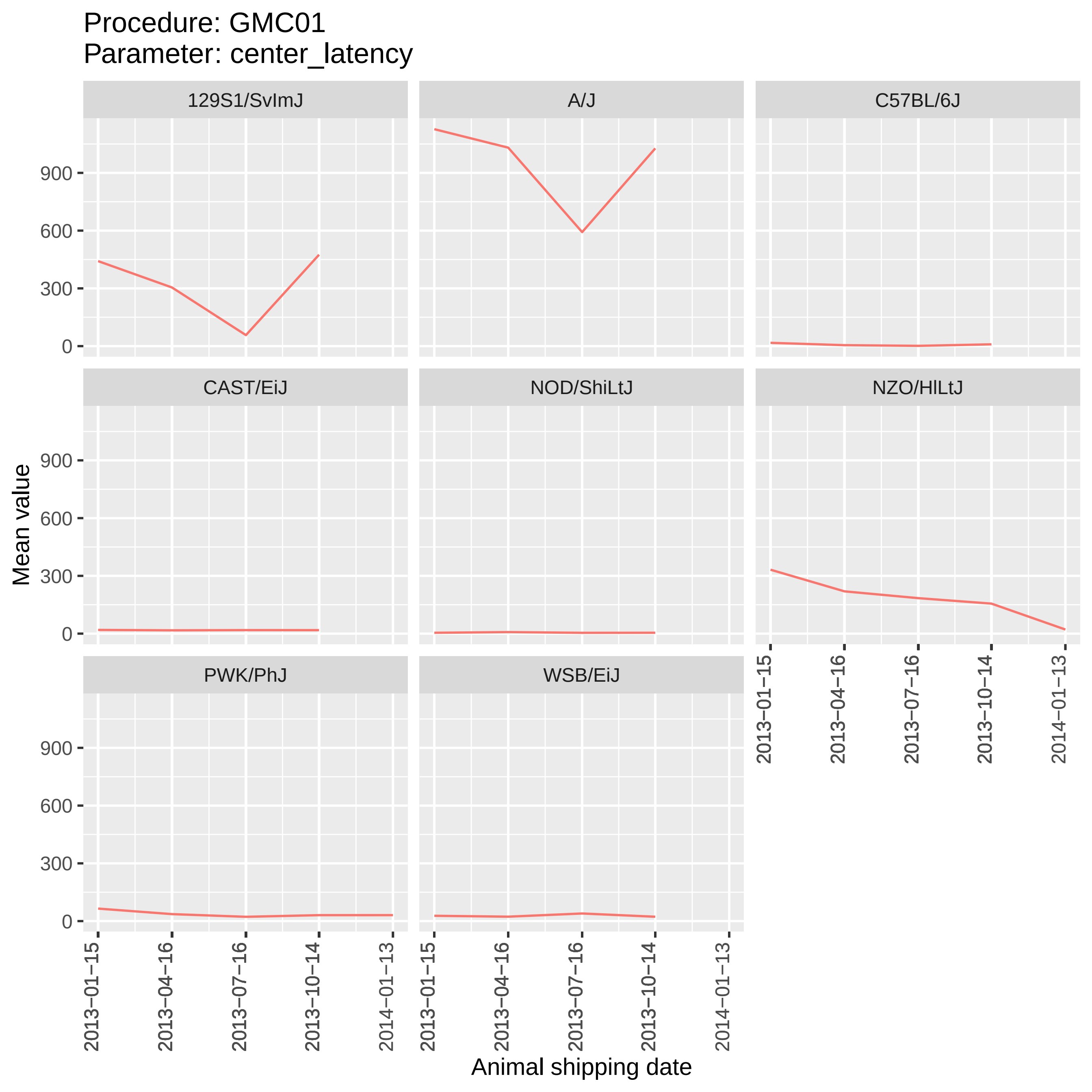

Procedure: GMC01
Parameter: center_latency
129S1/SvImJ
A/J
C57BL/6J
CAST/EiJ
NOD/ShiLtJ
NZO/HlLtJ
PWK/PhJ
WSB/EiJ
Mean value
0
300
600
900
2013−01−15
2013−04−16
2013−07−16
2013−10−14
2014−01−13
Animal shipping date

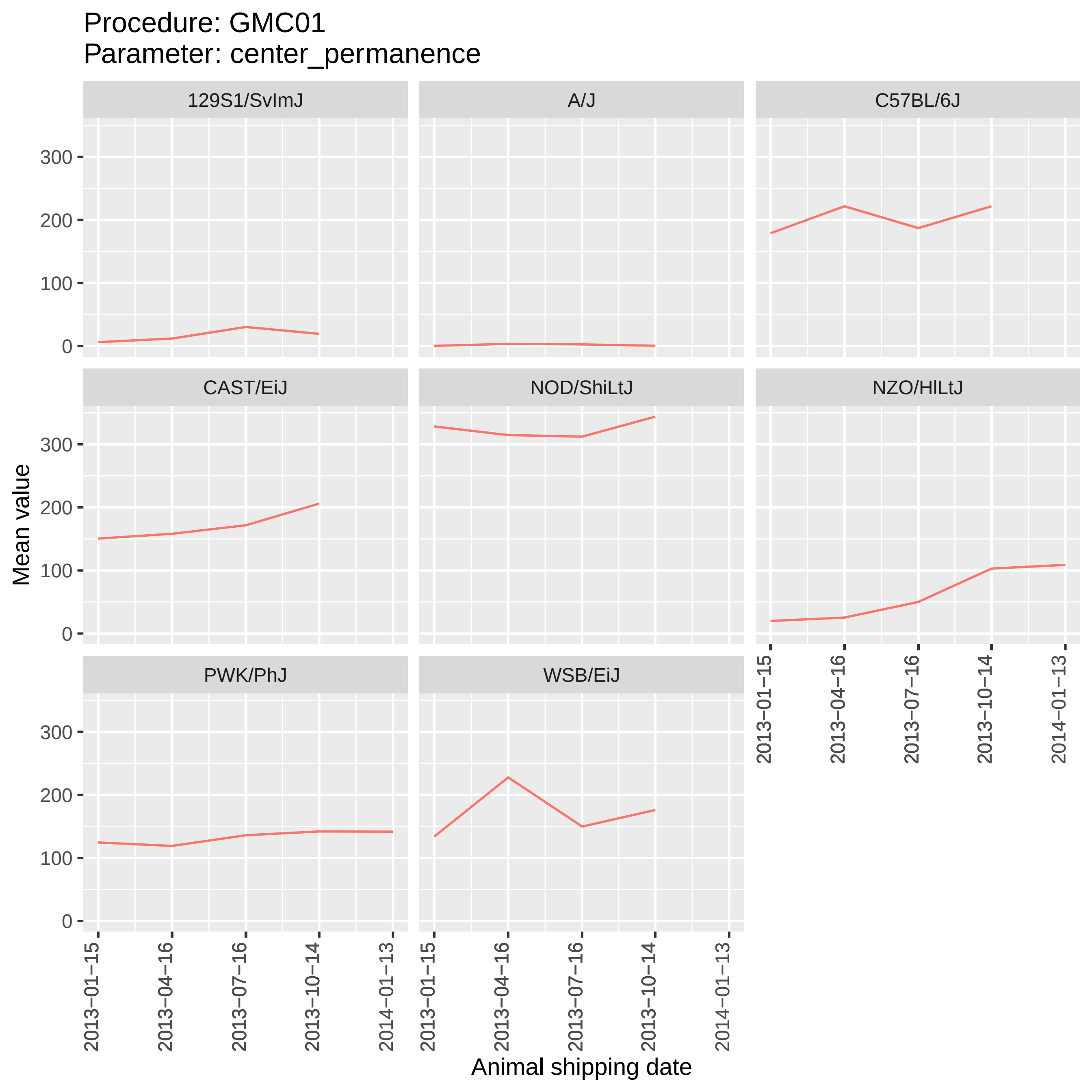

Procedure: GMC01
Parameter: center_permanence
129S1/SvImJ
A/J
C57BL/6J
CAST/EiJ
NOD/ShiLtJ
NZO/HlLtJ
PWK/PhJ
WSB/EiJ
Mean value
0
100
200
300
2013−01−15
2013−04−16
2013−07−16
2013−10−14
2014−01−13
Animal shipping date

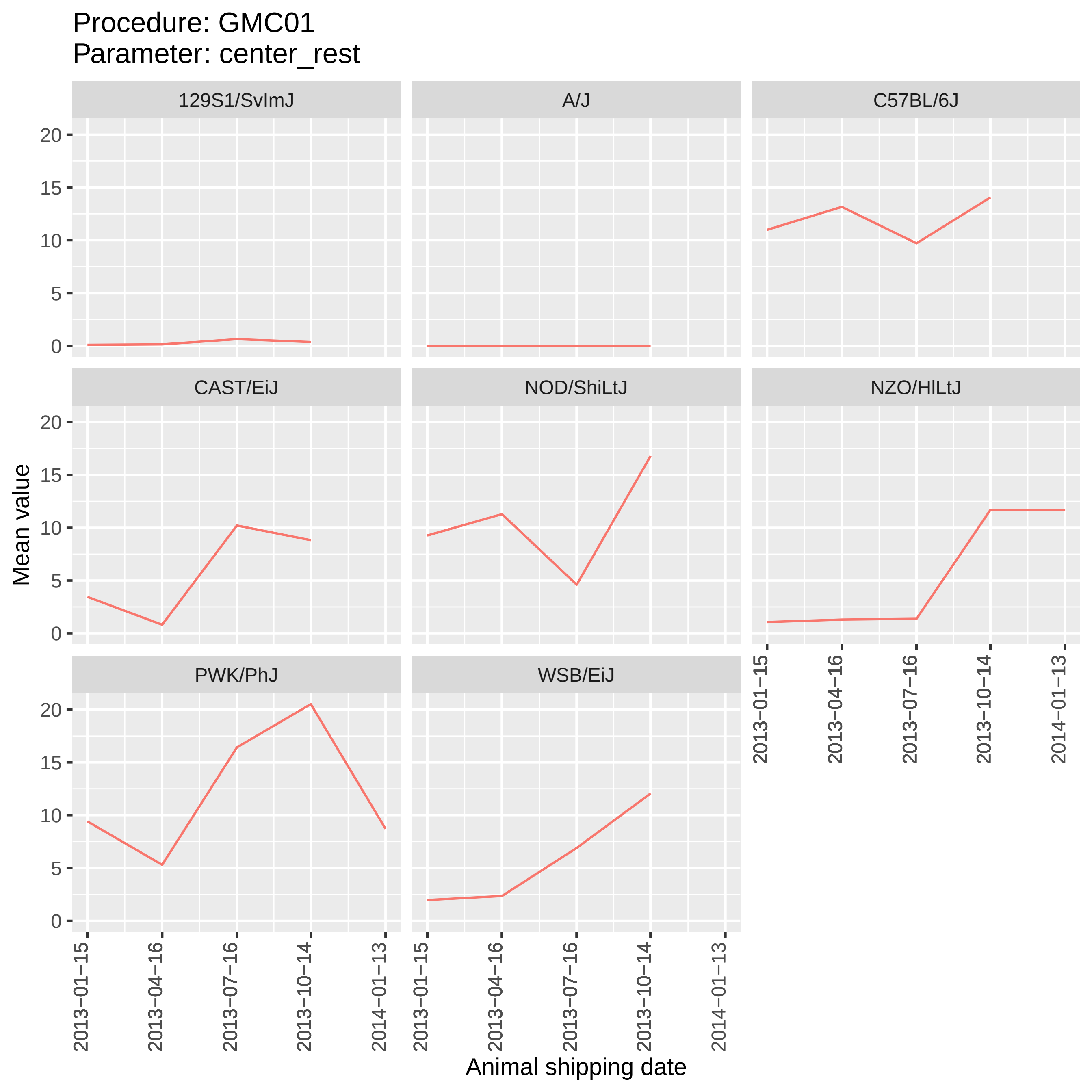
Procedure: GMC01
Parameter: center_rest
129S1/SvImJ
A/J
C57BL/6J
CAST/EiJ
NOD/ShiLtJ
NZO/HlLtJ
PWK/PhJ
WSB/EiJ
Mean value
0
5
10
15
20
2013−01−15
2013−04−16
2013−07−16
2013−10−14
2014−01−13
Animal shipping date

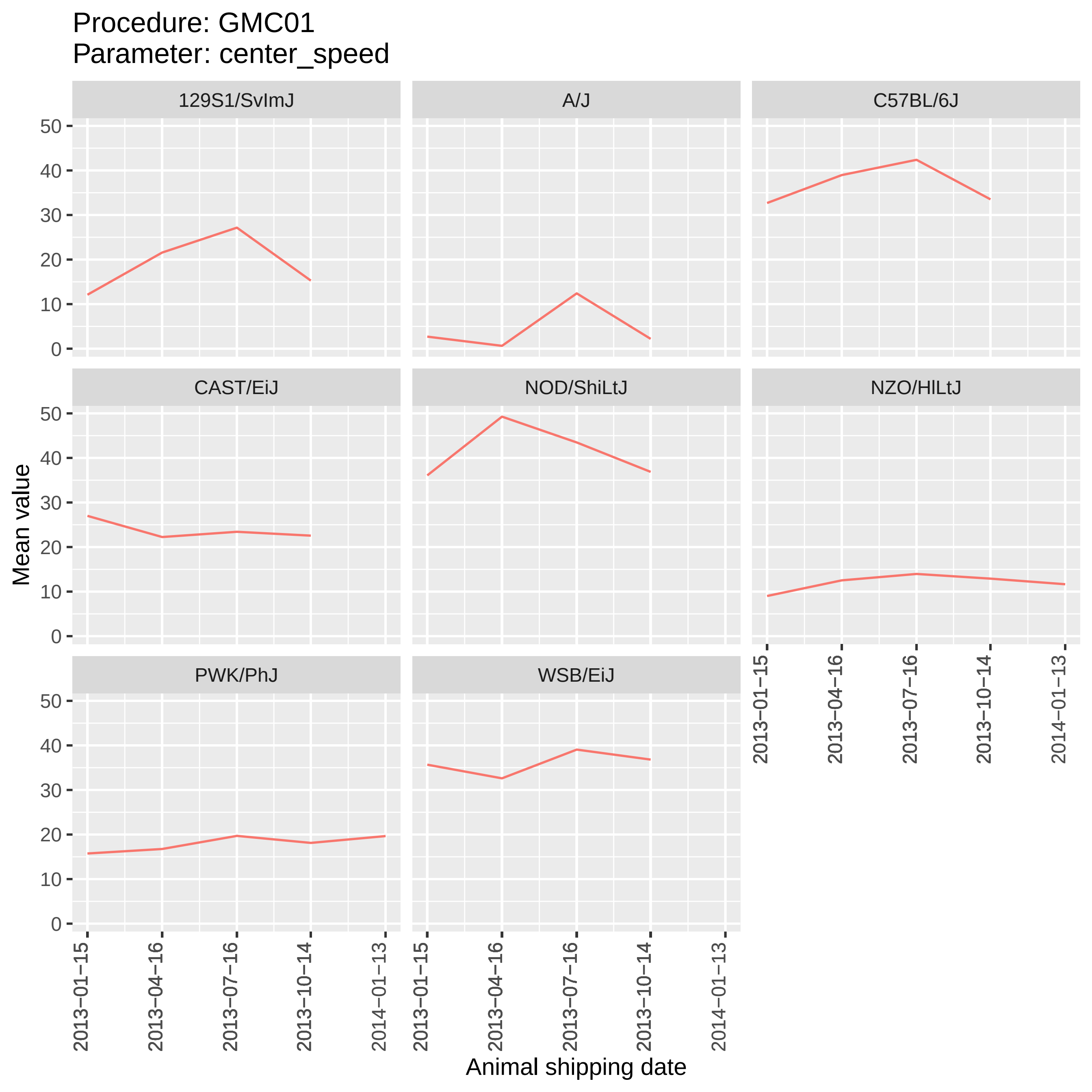
Procedure: GMC01
Parameter: center_speed
129S1/SvImJ
A/J
C57BL/6J
CAST/EiJ
NOD/ShiLtJ
NZO/HlLtJ
PWK/PhJ
WSB/EiJ
Mean value
0
10
20
30
40
50
2013−01−15
2013−04−16
2013−07−16
2013−10−14
2014−01−13
Animal shipping date

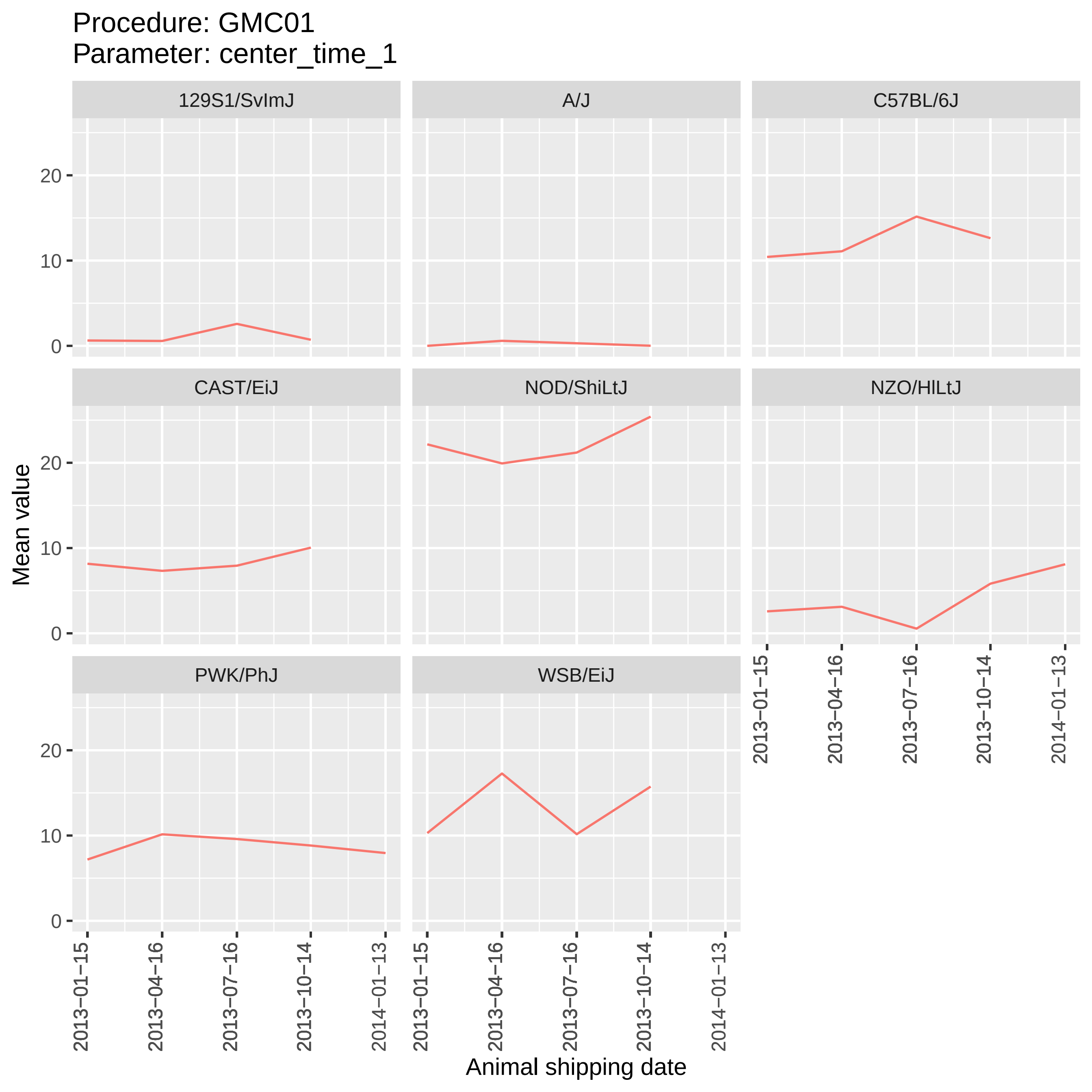

Procedure: GMC01
Parameter: center_time_1
129S1/SvImJ
A/J
C57BL/6J
CAST/EiJ
NOD/ShiLtJ
NZO/HlLtJ
PWK/PhJ
WSB/EiJ
Mean value
0
10
20
2013−01−15
2013−04−16
2013−07−16
2013−10−14
2014−01−13
Animal shipping date

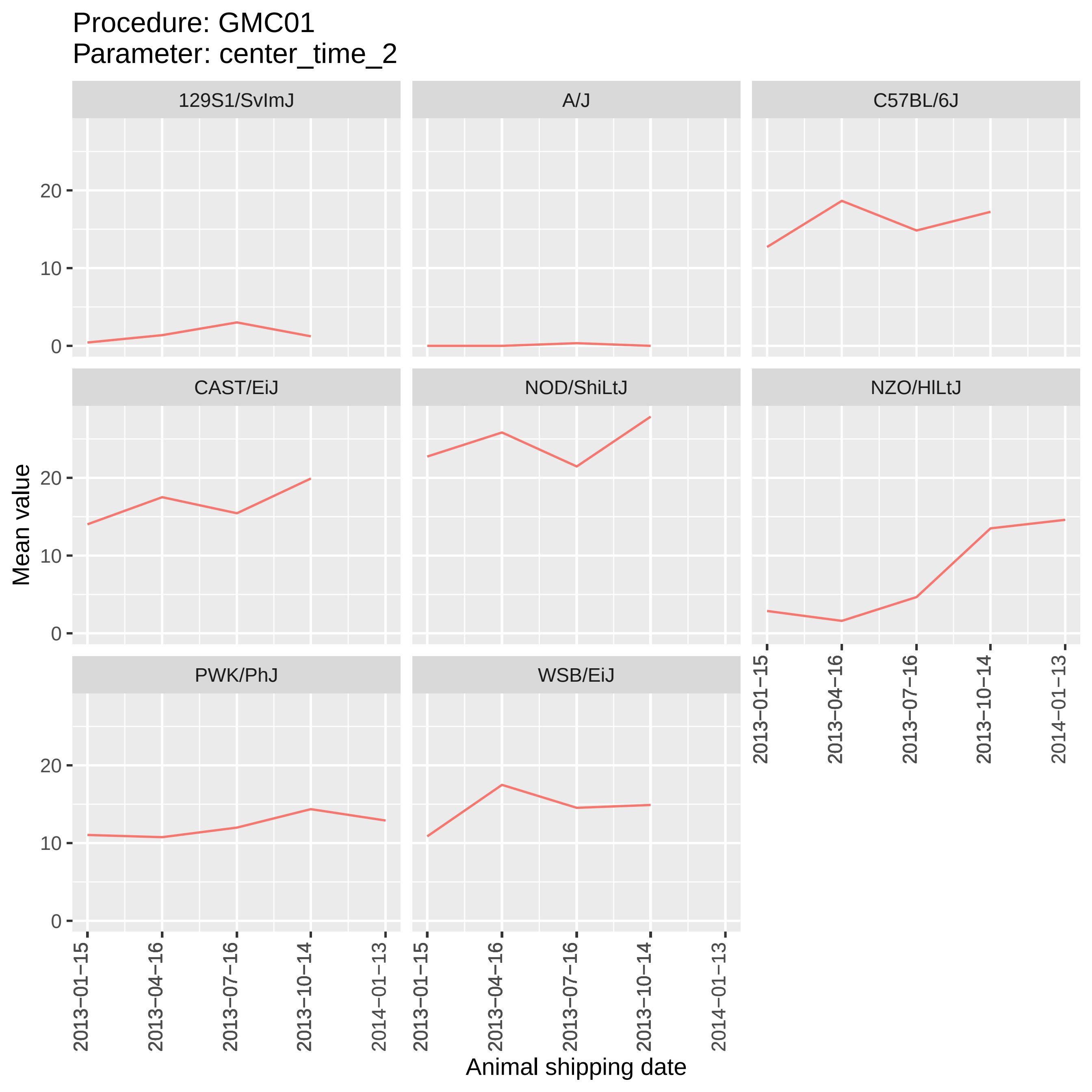
Procedure: GMC01
Parameter: center_time_2
129S1/SvImJ
A/J
C57BL/6J
CAST/EiJ
NOD/ShiLtJ
NZO/HlLtJ
PWK/PhJ
WSB/EiJ
Mean value
0
10
20
2013−01−15
2013−04−16
2013−07−16
2013−10−14
2014−01−13
Animal shipping date

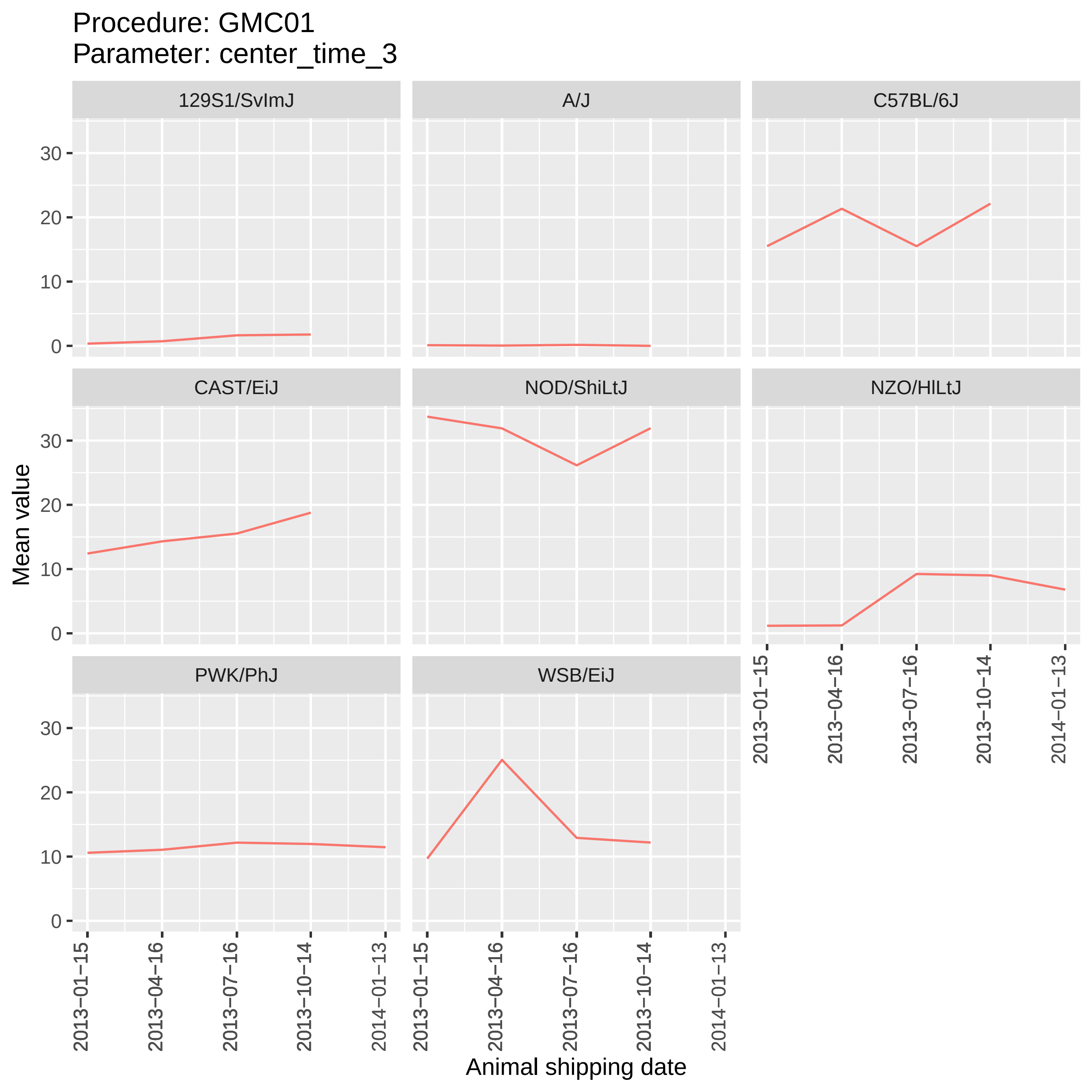

Procedure: GMC01
Parameter: center_time_3
129S1/SvImJ
A/J
C57BL/6J
CAST/EiJ
NOD/ShiLtJ
NZO/HlLtJ
PWK/PhJ
WSB/EiJ
Mean value
0
10
20
30
2013−01−15
2013−04−16
2013−07−16
2013−10−14
2014−01−13
Animal shipping date

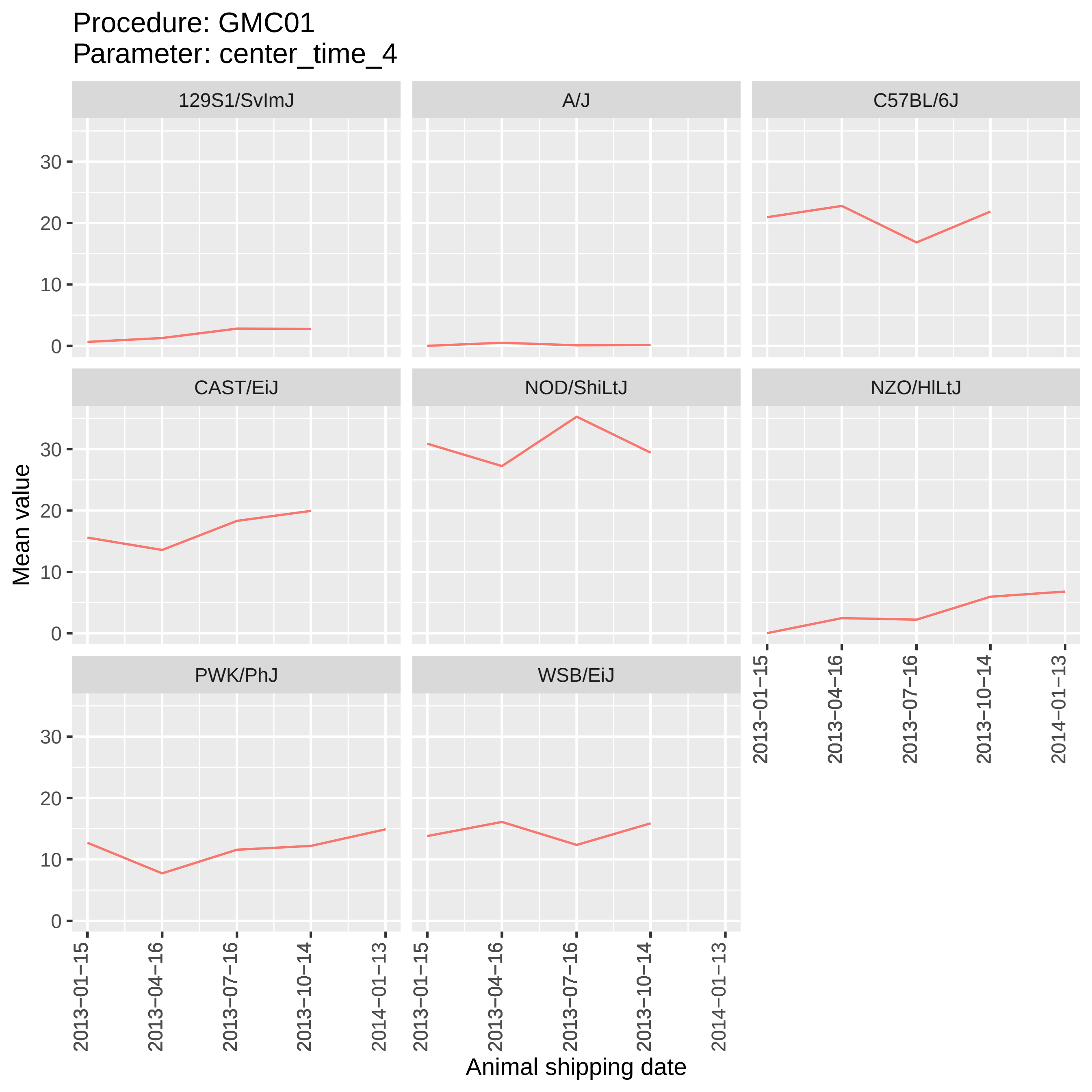
Procedure: GMC01
Parameter: center_time_4
129S1/SvImJ
A/J
C57BL/6J
CAST/EiJ
NOD/ShiLtJ
NZO/HlLtJ
PWK/PhJ
WSB/EiJ
Mean value
0
10
20
30
2013−01−15
2013−04−16
2013−07−16
2013−10−14
2014−01−13
Animal shipping date

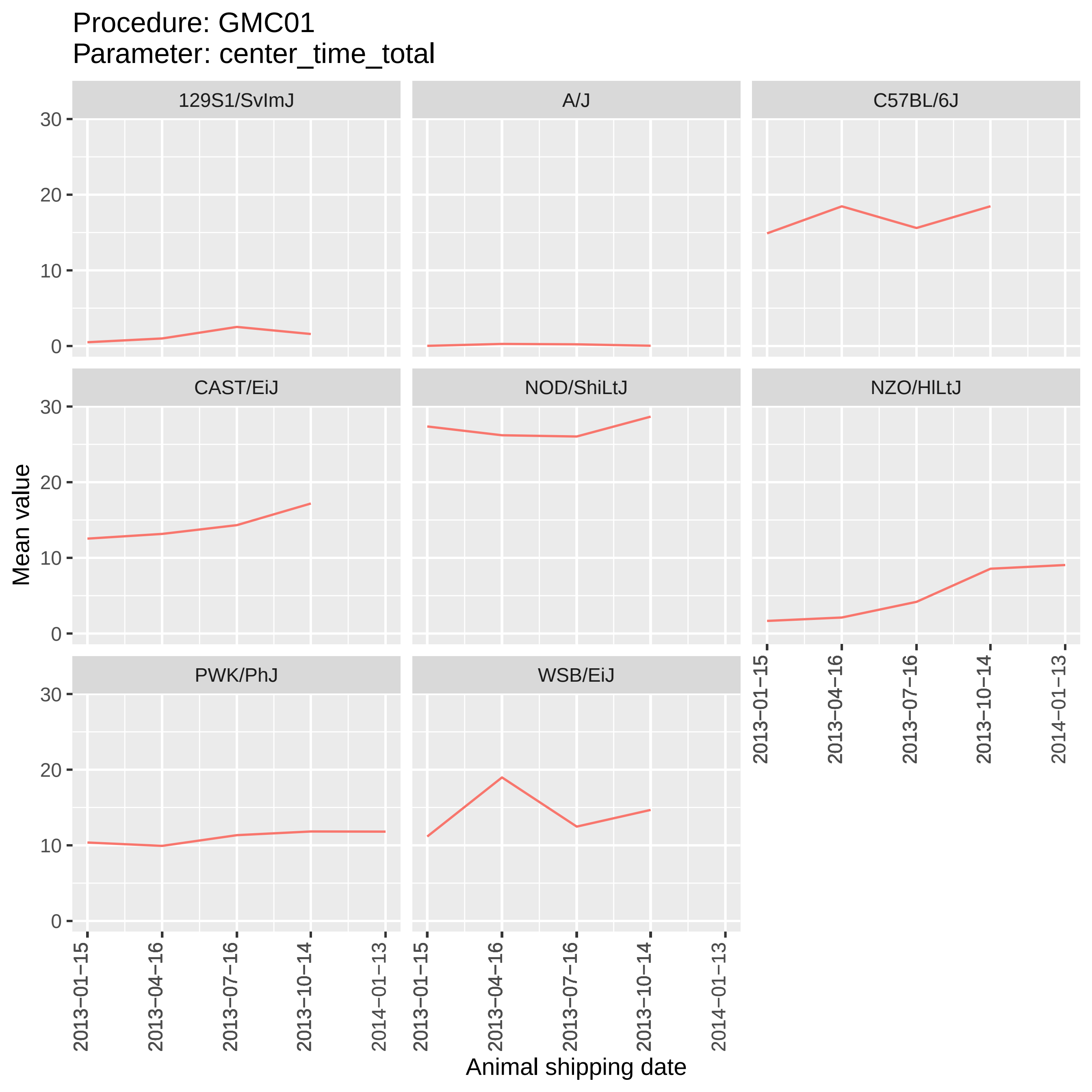

Procedure: GMC01
Parameter: center_time_total
129S1/SvImJ
A/J
C57BL/6J
CAST/EiJ
NOD/ShiLtJ
NZO/HlLtJ
PWK/PhJ
WSB/EiJ
Mean value
0
10
20
30
2013−01−15
2013−04−16
2013−07−16
2013−10−14
2014−01−13
Animal shipping date

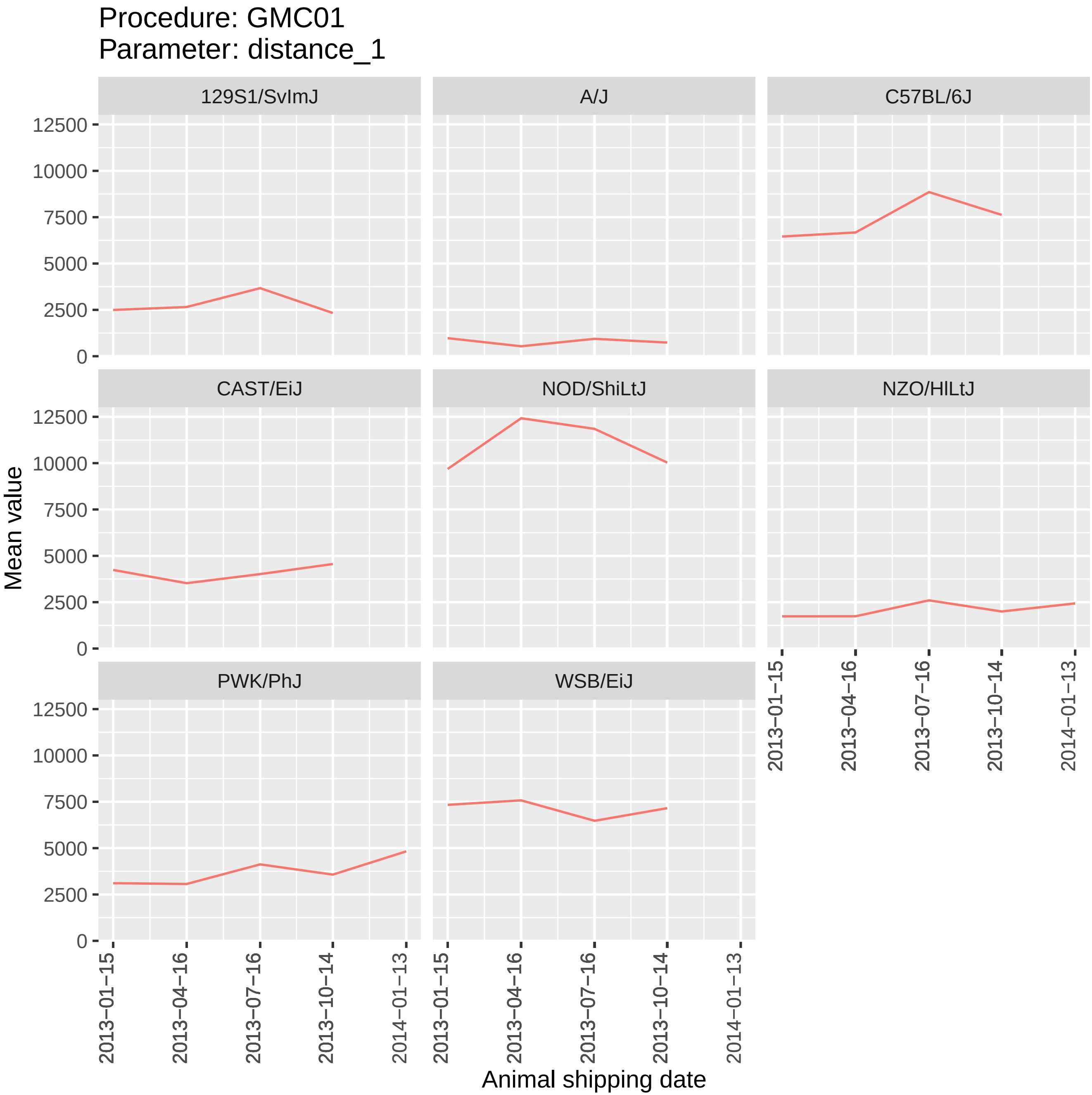

Procedure: GMC01
Parameter: distance_1
129S1/SvImJ
A/J
C57BL/6J
CAST/EiJ
NOD/ShiLtJ
NZO/HlLtJ
PWK/PhJ
WSB/EiJ
Mean value
0
2500
5000
7500
10000
12500
2013−01−15
2013−04−16
2013−07−16
2013−10−14
2014−01−13
Animal shipping date

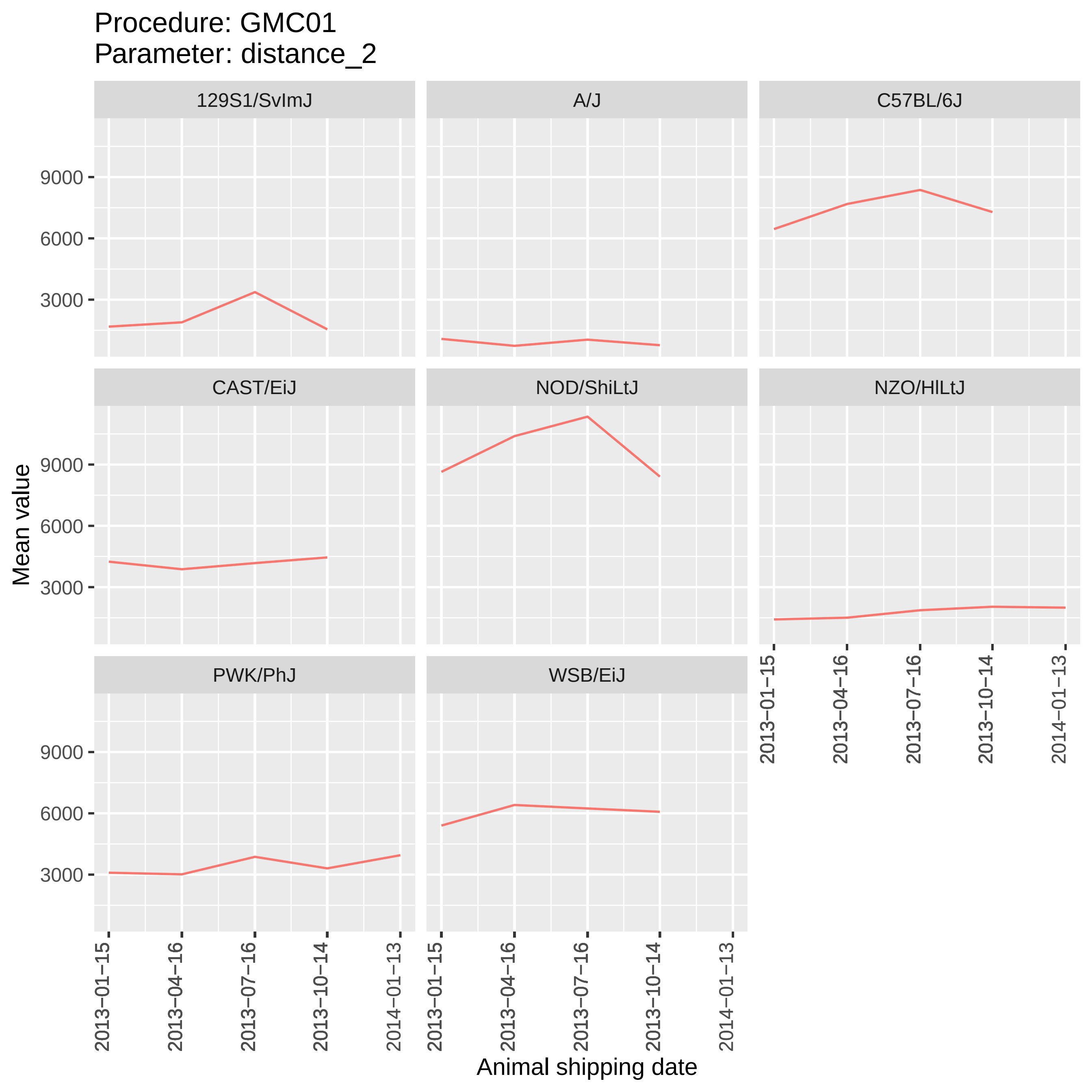

Procedure: GMC01
Parameter: distance_2
129S1/SvImJ
A/J
C57BL/6J
CAST/EiJ
NOD/ShiLtJ
NZO/HlLtJ
PWK/PhJ
WSB/EiJ
9000
6000
3000
Mean value
2013−01−15
2013−04−16
2013−07−16
2013−10−14
2014−01−13
Animal shipping date

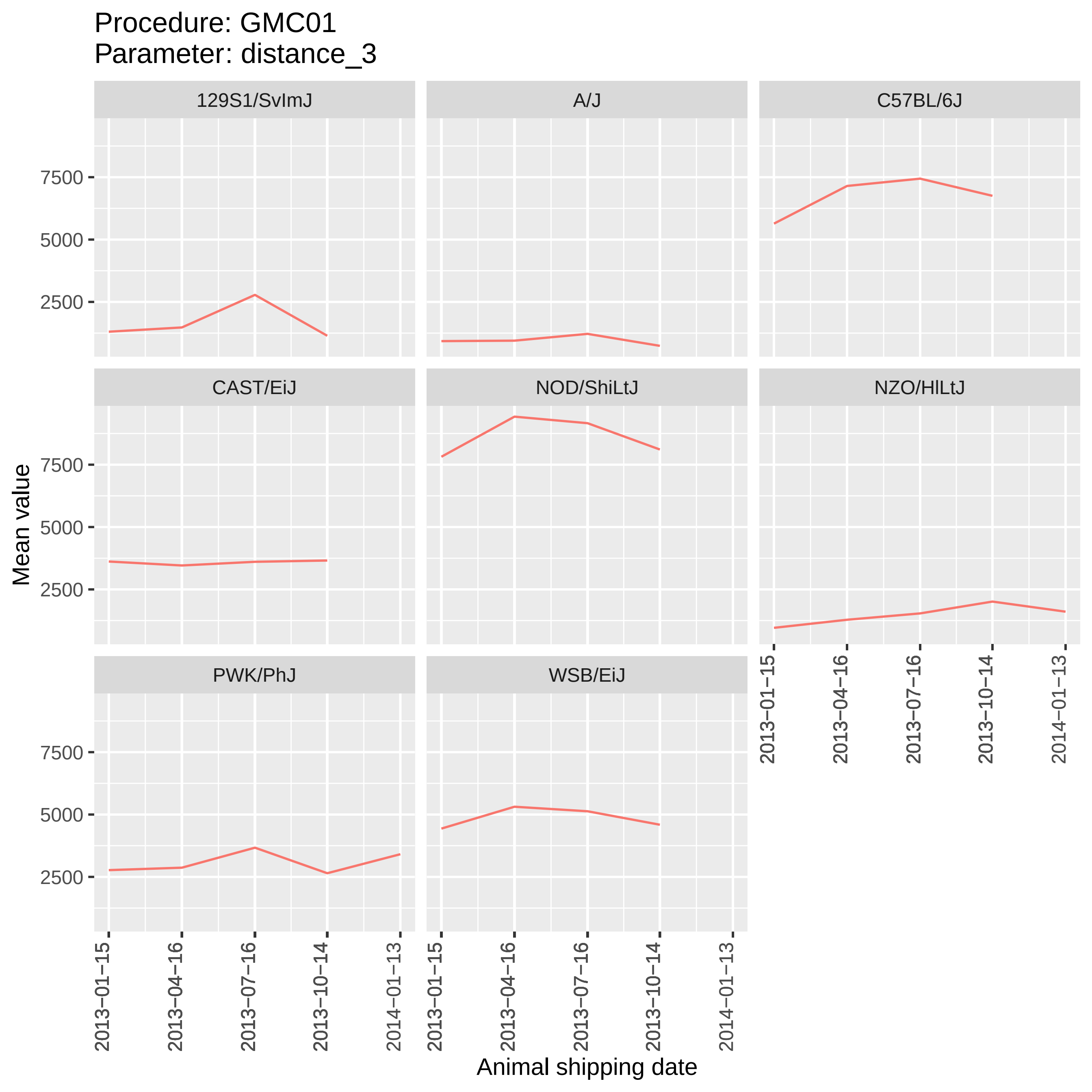
Procedure: GMC01
Parameter: distance_3
129S1/SvImJ
A/J
C57BL/6J
CAST/EiJ
NOD/ShiLtJ
NZO/HlLtJ
PWK/PhJ
WSB/EiJ
7500
5000
2500
Mean value
2013−01−15
2013−04−16
2013−07−16
2013−10−14
2014−01−13
Animal shipping date

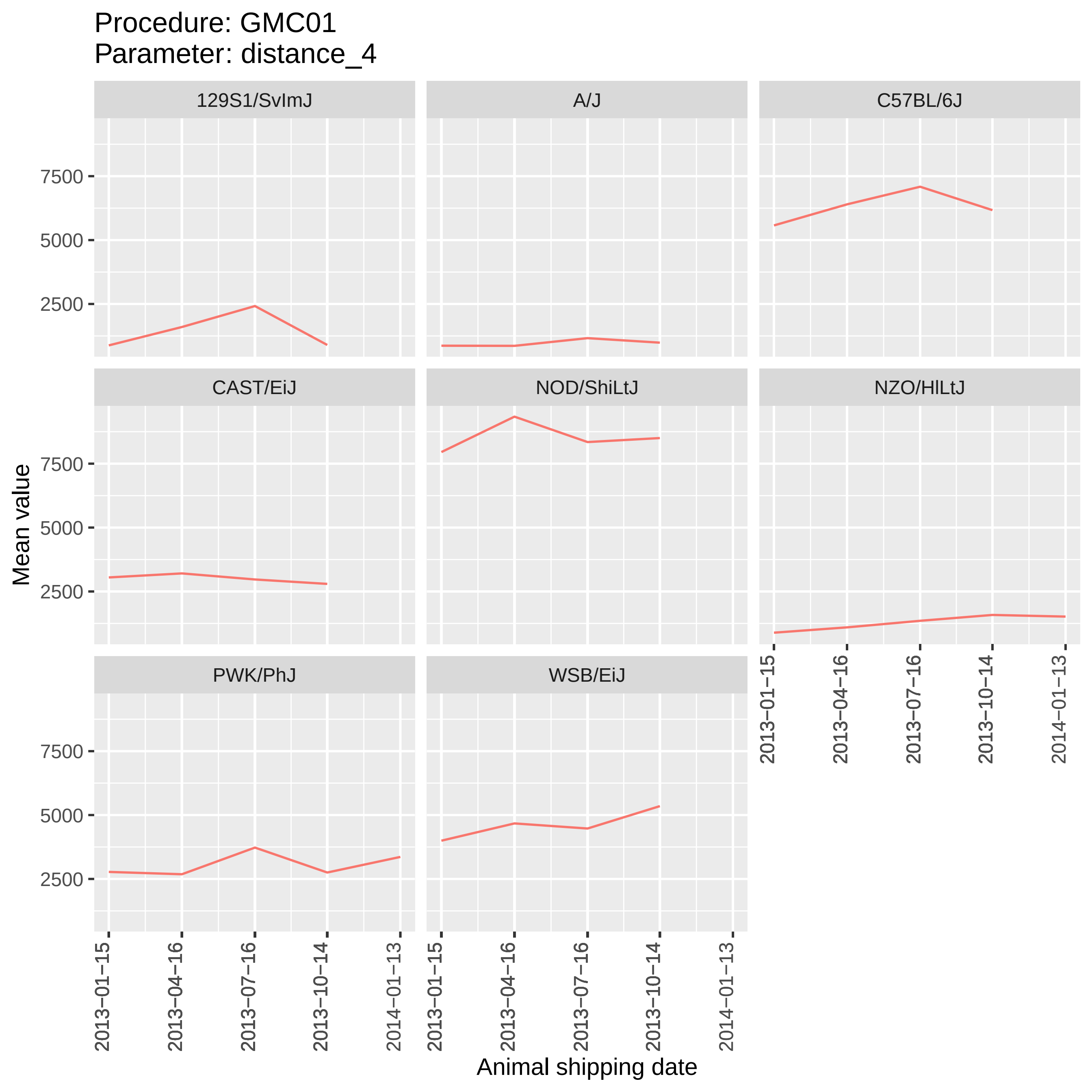

Procedure: GMC01
Parameter: distance_4
129S1/SvImJ
A/J
C57BL/6J
CAST/EiJ
NOD/ShiLtJ
NZO/HlLtJ
PWK/PhJ
WSB/EiJ
7500
5000
2500
2013−01−15
2013−04−16
2013−07−16
2013−10−14
2014−01−13
Mean value
Animal shipping date

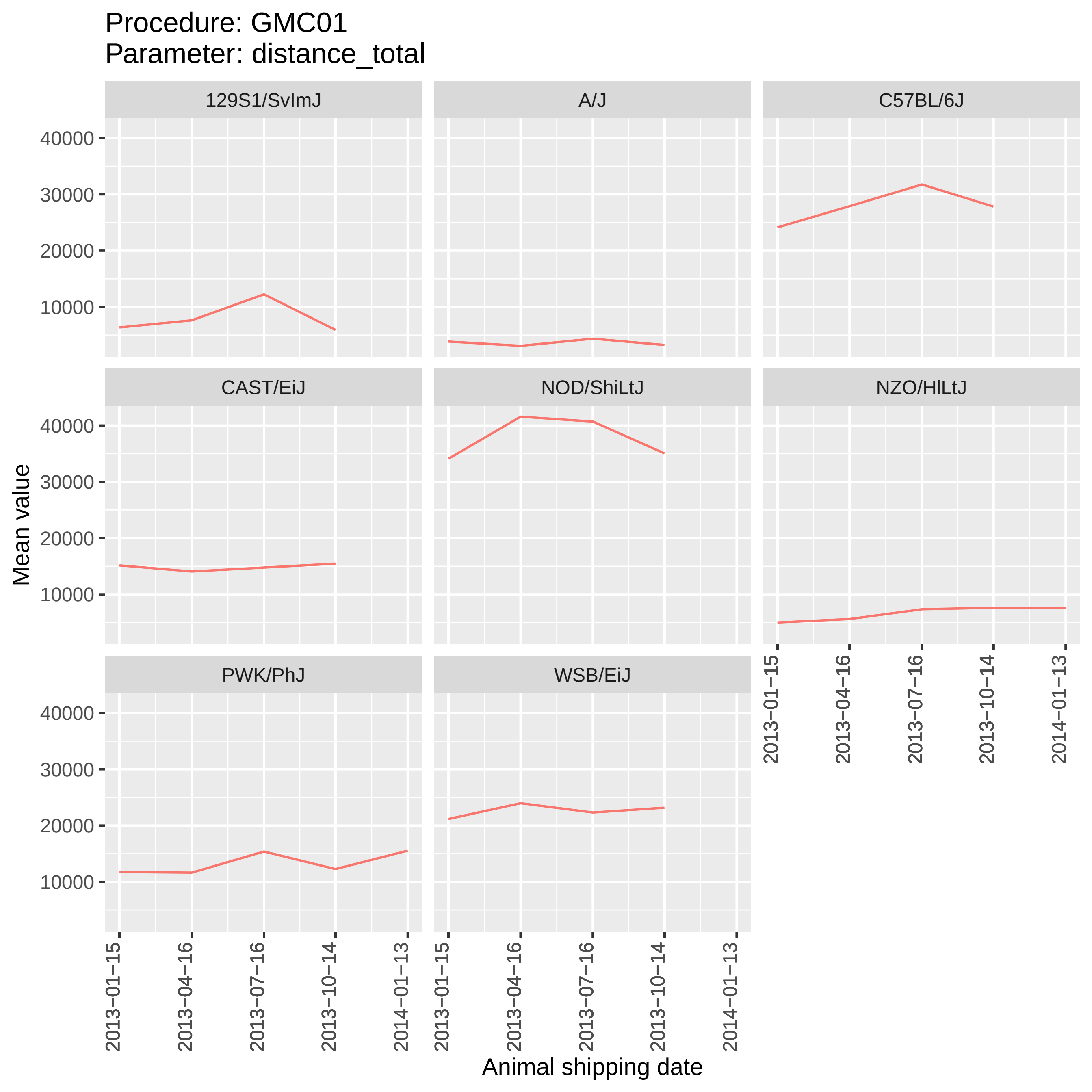
Procedure: GMC01
Parameter: distance_total
129S1/SvImJ
A/J
C57BL/6J
CAST/EiJ
NOD/ShiLtJ
NZO/HlLtJ
PWK/PhJ
WSB/EiJ
Mean value
10000
20000
30000
40000
2013−01−15
2013−04−16
2013−07−16
2013−10−14
2014−01−13
Animal shipping date

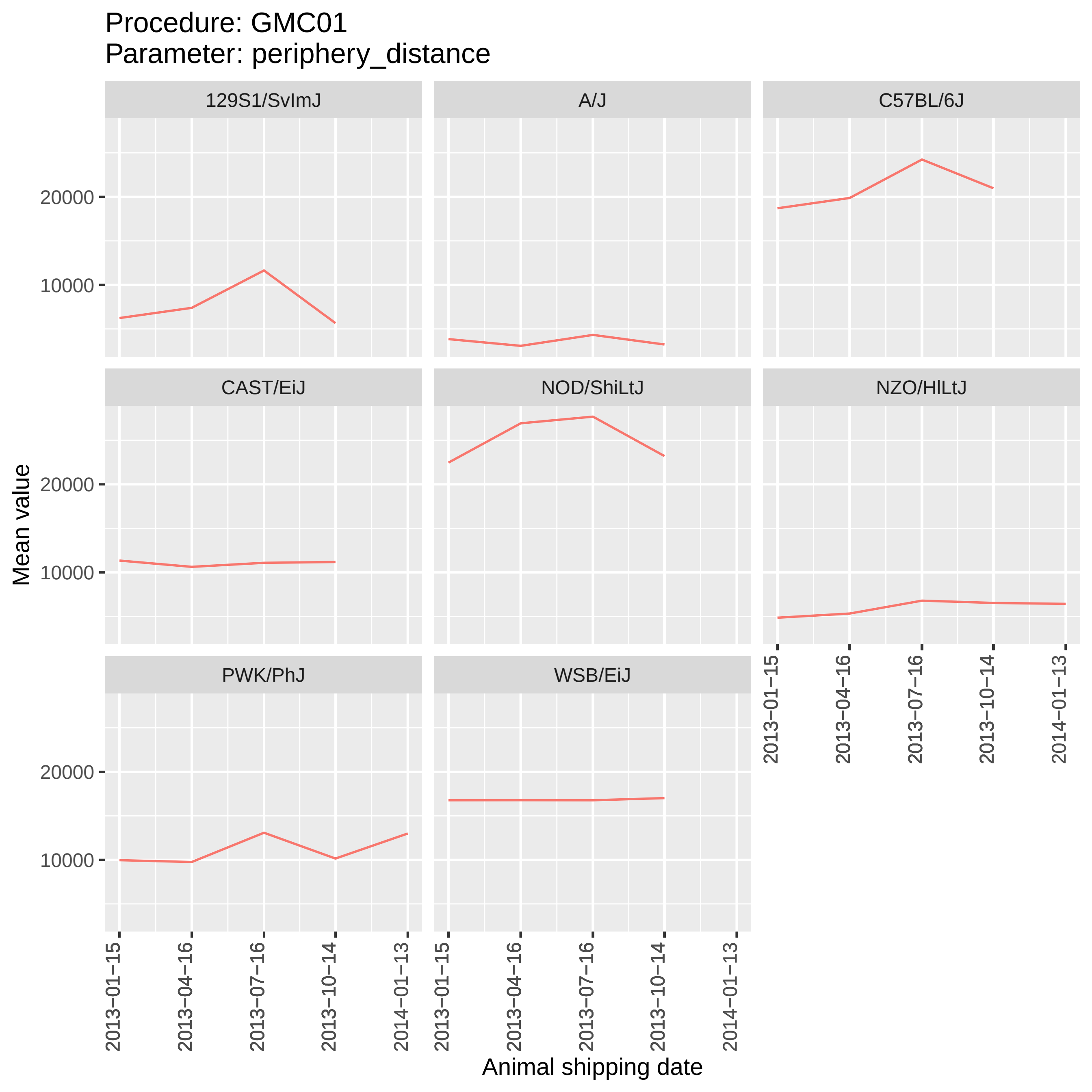

Procedure: GMC01
Parameter: periphery_distance
129S1/SvImJ
A/J
C57BL/6J
CAST/EiJ
NOD/ShiLtJ
NZO/HlLtJ
PWK/PhJ
WSB/EiJ
Mean value
20000
10000
2013−01−15
2013−04−16
2013−07−16
2013−10−14
2014−01−13
Animal shipping date

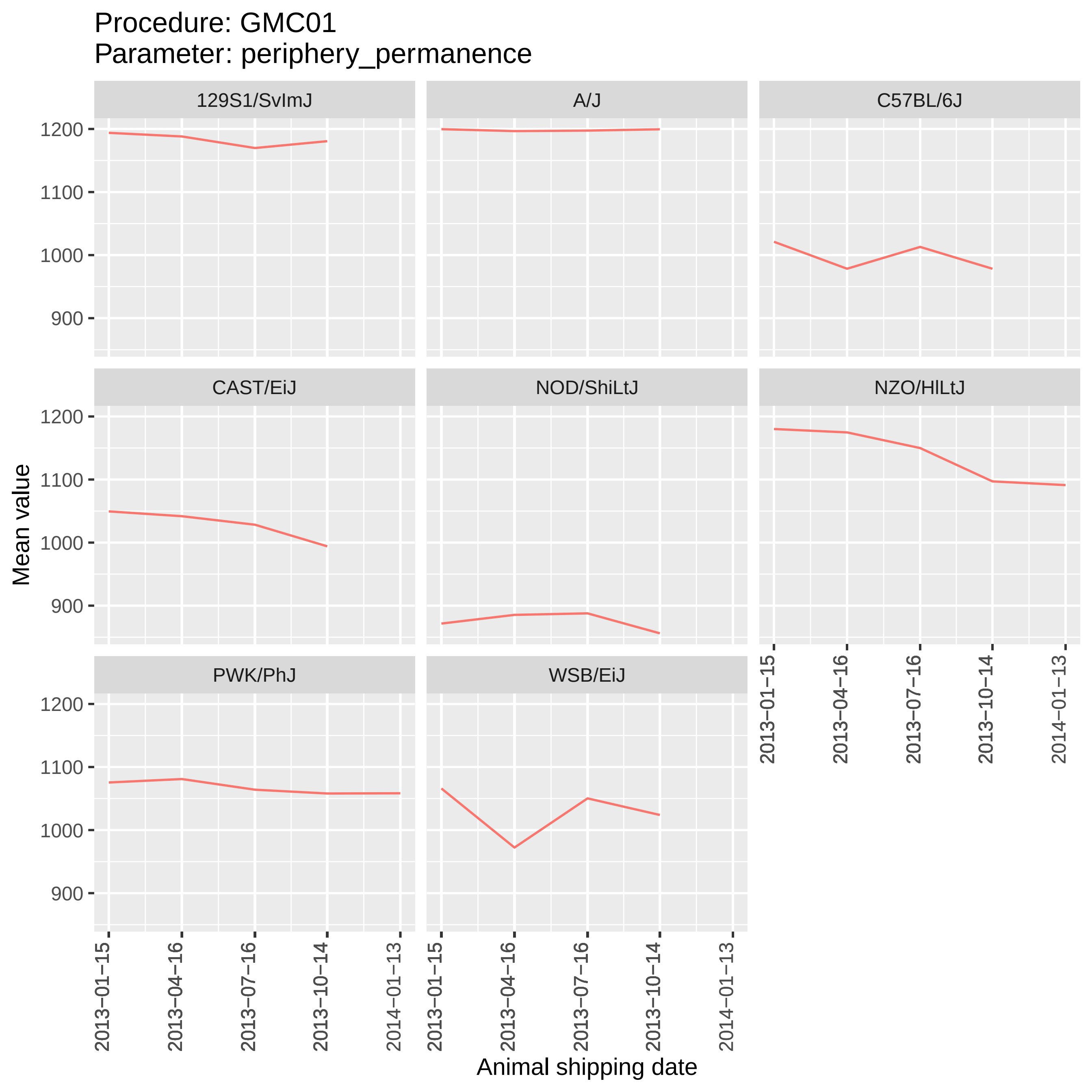

Procedure: GMC01
Parameter: periphery_permanence
129S1/SvImJ
A/J
C57BL/6J
CAST/EiJ
NOD/ShiLtJ
NZO/HlLtJ
PWK/PhJ
WSB/EiJ
Mean value
1200
1100
1000
900
2013−01−15
2013−04−16
2013−07−16
2013−10−14
2014−01−13
Animal shipping date

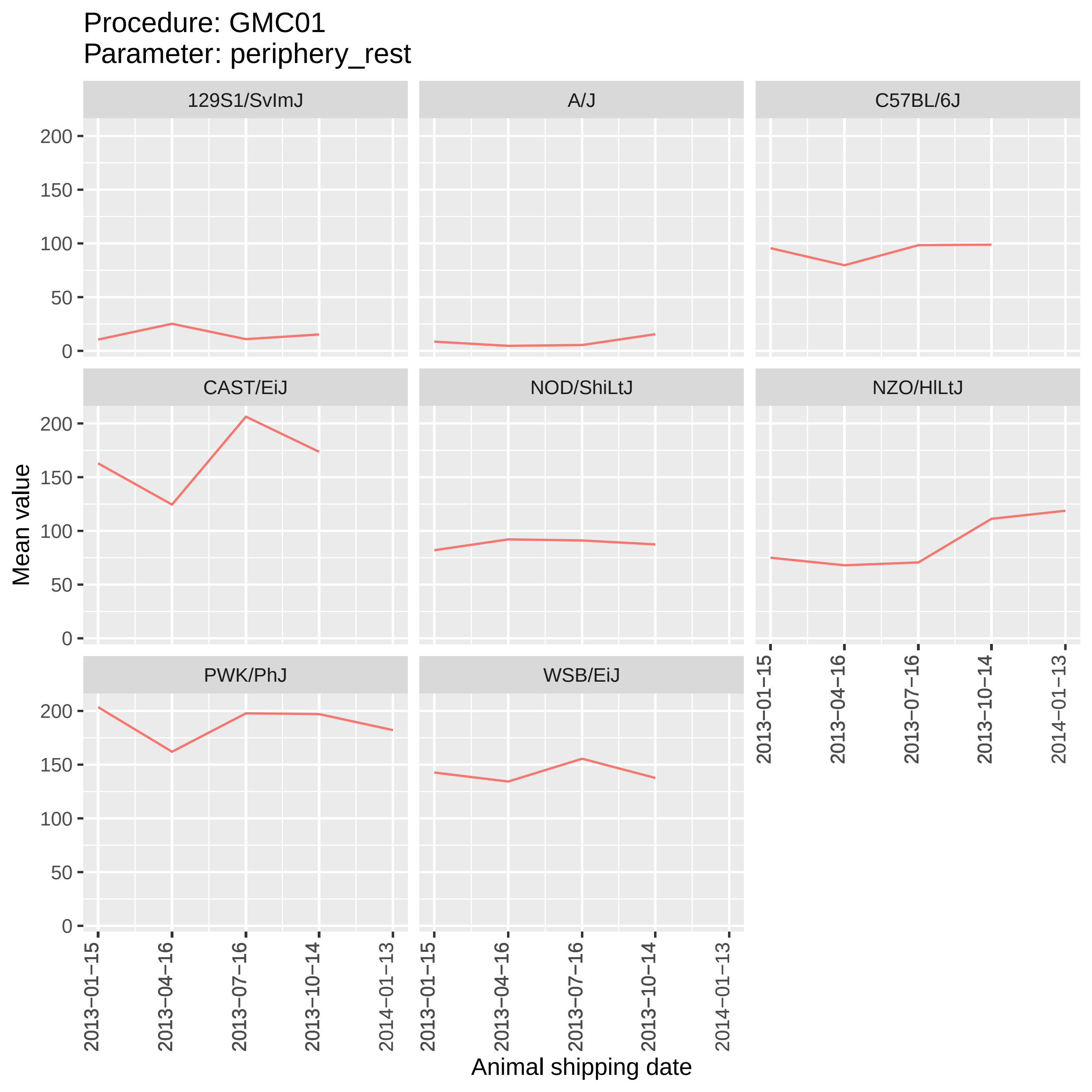

Procedure: GMC01
Parameter: periphery_rest
129S1/SvImJ
A/J
C57BL/6J
CAST/EiJ
NOD/ShiLtJ
NZO/HlLtJ
PWK/PhJ
WSB/EiJ
Mean value
0
50
100
150
200
2013−01−15
2013−04−16
2013−07−16
2013−10−14
2014−01−13
Animal shipping date

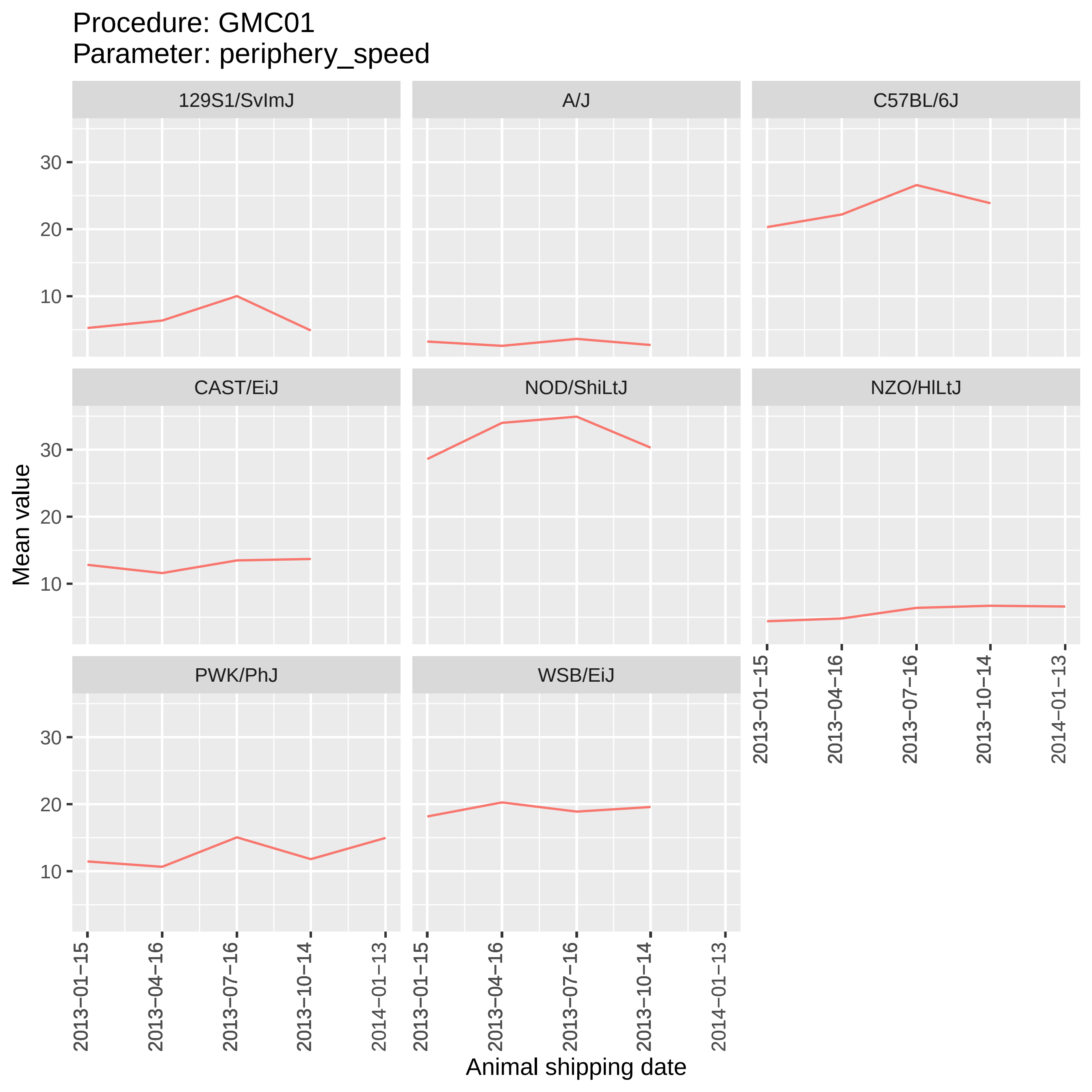

Procedure: GMC01
Parameter: periphery_speed
129S1/SvImJ
A/J
C57BL/6J
CAST/EiJ
NOD/ShiLtJ
NZO/HlLtJ
PWK/PhJ
WSB/EiJ
Mean value
10
20
30
2013−01−15
2013−04−16
2013−07−16
2013−10−14
2014−01−13
Animal shipping date

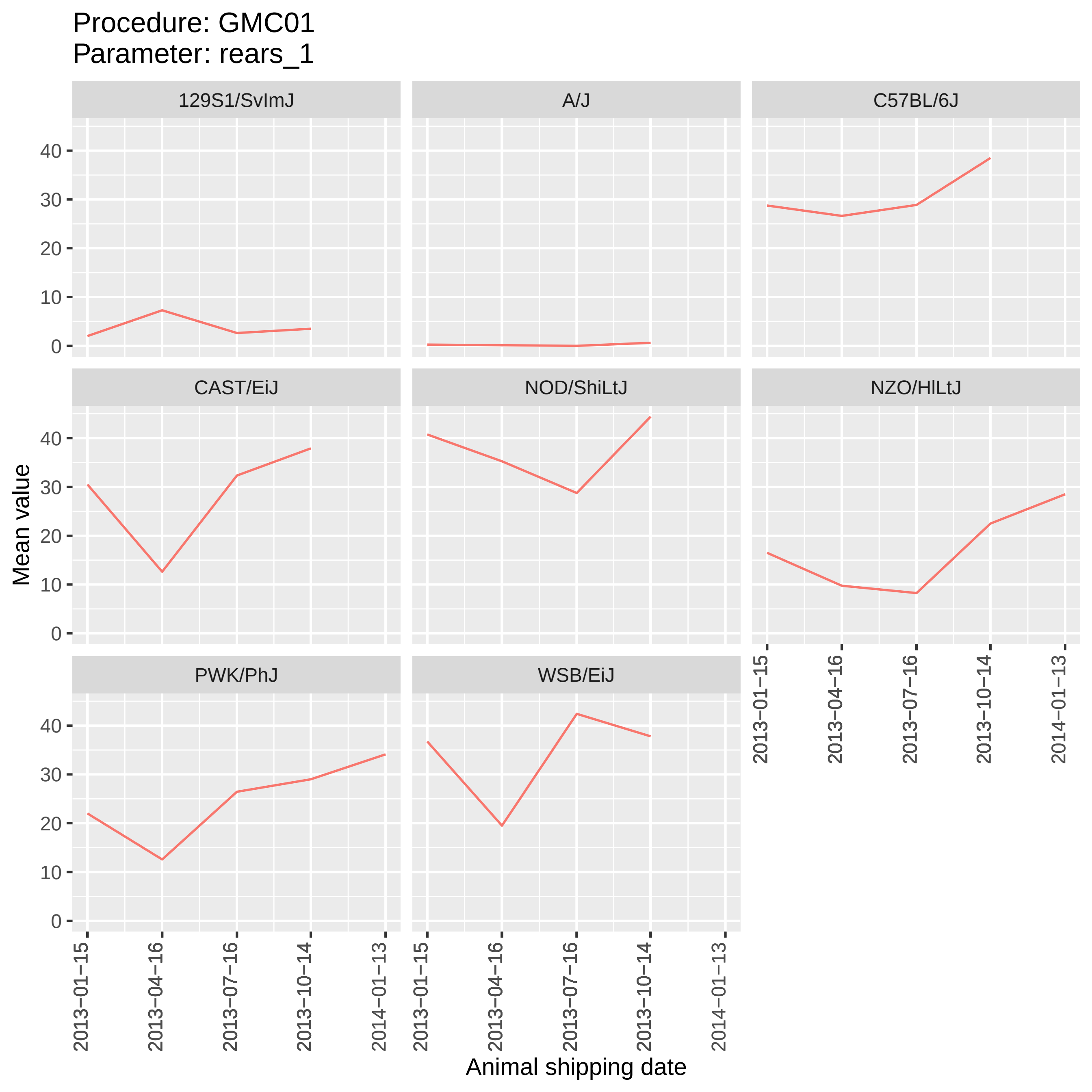

Procedure: GMC01
Parameter: rears_1
129S1/SvImJ
A/J
C57BL/6J
CAST/EiJ
NOD/ShiLtJ
NZO/HlLtJ
PWK/PhJ
WSB/EiJ
Mean value
0
10
20
30
40
2013−01−15
2013−04−16
2013−07−16
2013−10−14
2014−01−13
Animal shipping date

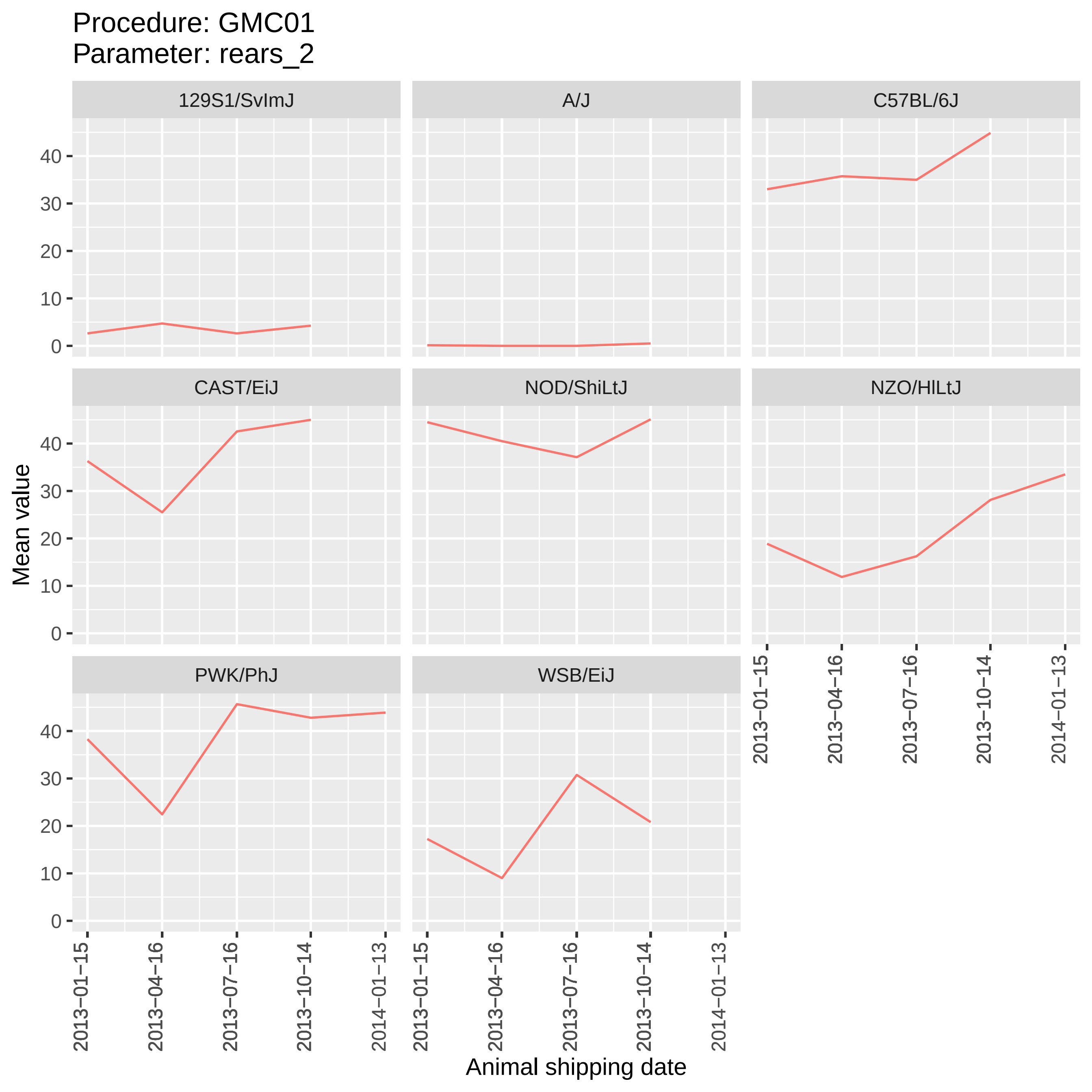

Procedure: GMC01
Parameter: rears_2
129S1/SvImJ
A/J
C57BL/6J
CAST/EiJ
NOD/ShiLtJ
NZO/HlLtJ
PWK/PhJ
WSB/EiJ
Mean value
0
10
20
30
40
2013−01−15
2013−04−16
2013−07−16
2013−10−14
2014−01−13
Animal shipping date

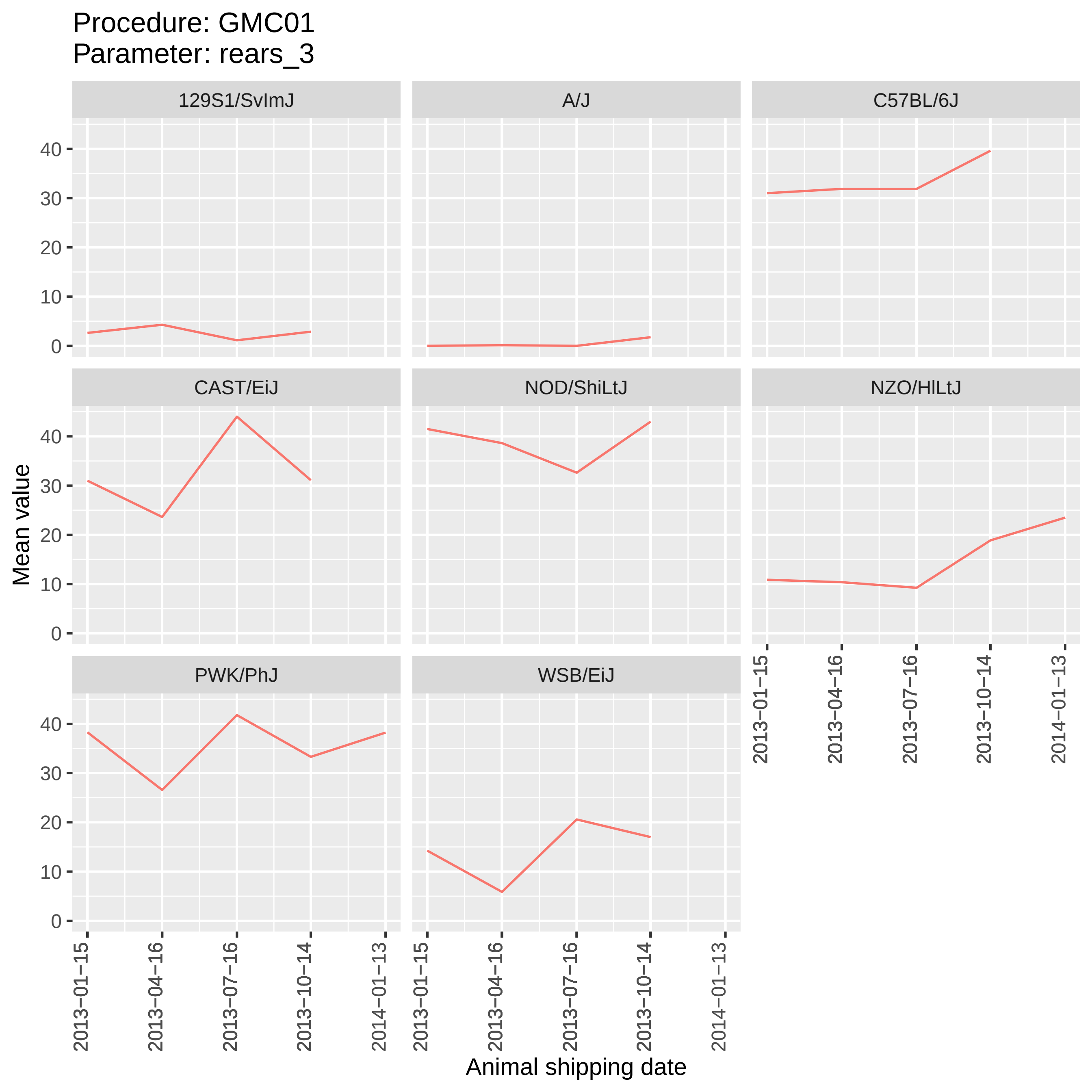

Procedure: GMC01
Parameter: rears_3
129S1/SvImJ
A/J
C57BL/6J
CAST/EiJ
NOD/ShiLtJ
NZO/HlLtJ
PWK/PhJ
WSB/EiJ
Mean value
0
10
20
30
40
2013−01−15
2013−04−16
2013−07−16
2013−10−14
2014−01−13
Animal shipping date

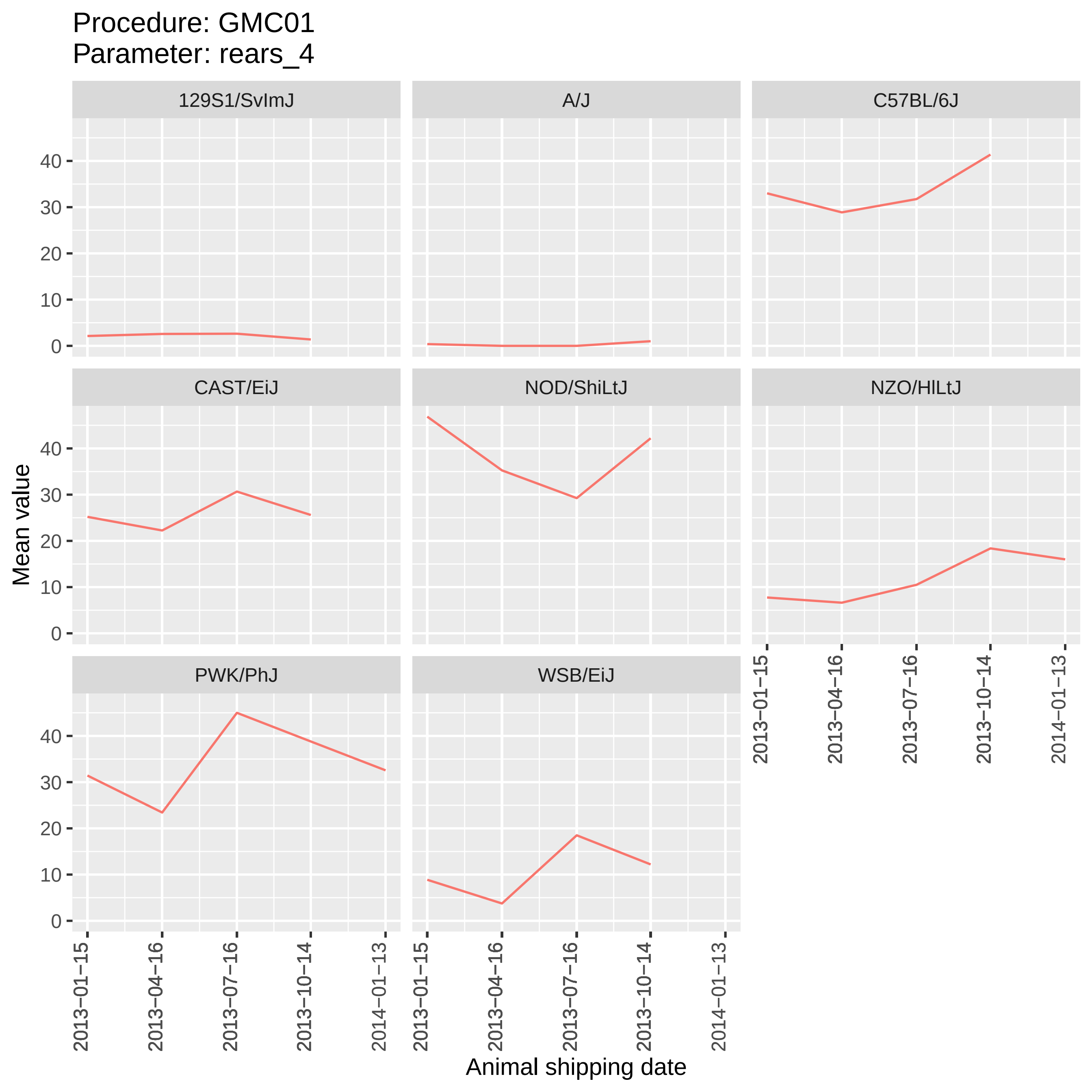

Procedure: GMC01
Parameter: rears_4
129S1/SvImJ
A/J
C57BL/6J
CAST/EiJ
NOD/ShiLtJ
NZO/HlLtJ
PWK/PhJ
WSB/EiJ
Mean value
0
10
20
30
40
2013−01−15
2013−04−16
2013−07−16
2013−10−14
2014−01−13
Animal shipping date

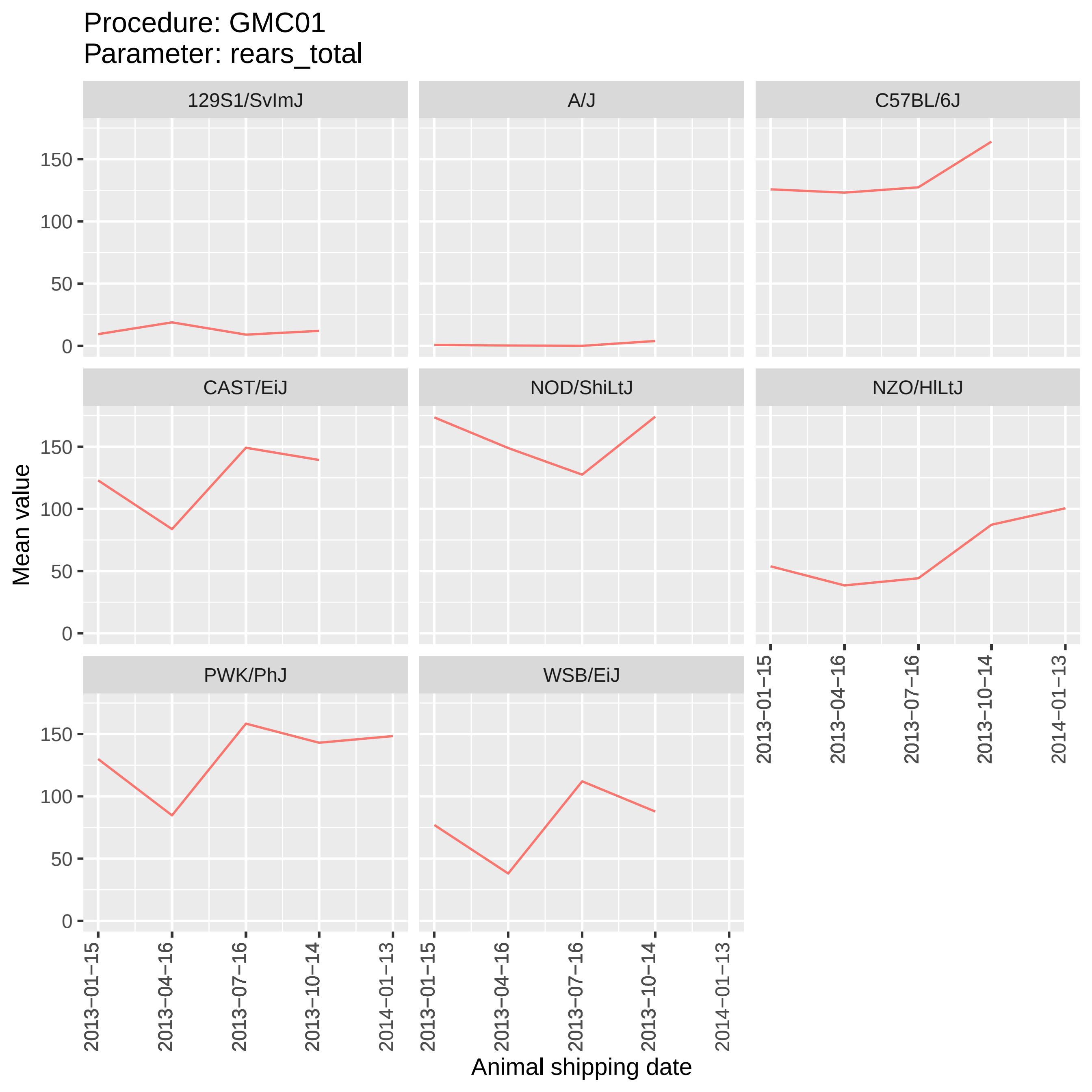

Procedure: GMC01
Parameter: rears_total
129S1/SvImJ
A/J
C57BL/6J
CAST/EiJ
NOD/ShiLtJ
NZO/HlLtJ
PWK/PhJ
WSB/EiJ
Mean value
0
50
100
150
2013−01−15
2013−04−16
2013−07−16
2013−10−14
2014−01−13
Animal shipping date

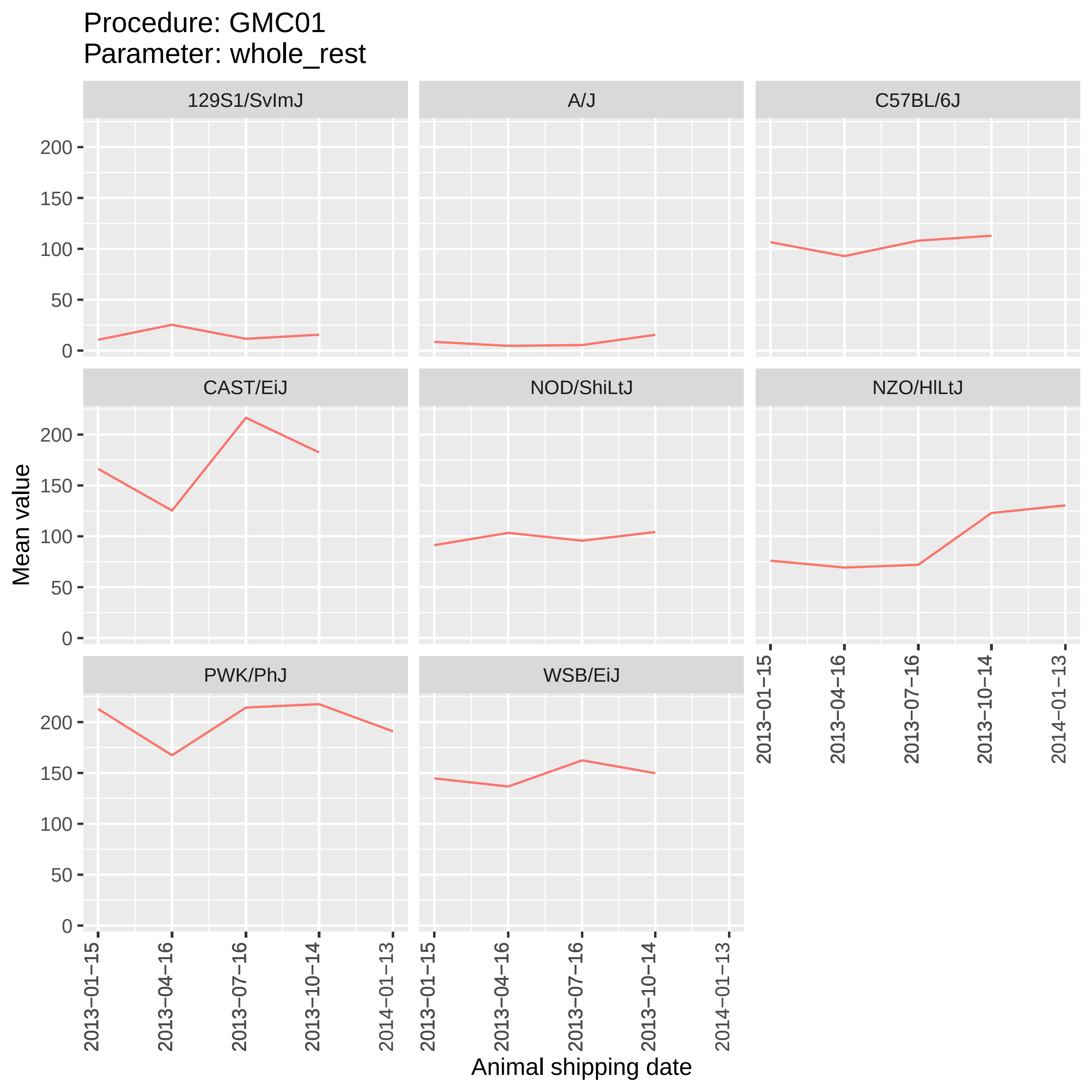

Procedure: GMC01
Parameter: whole_rest
129S1/SvImJ
A/J
C57BL/6J
CAST/EiJ
NOD/ShiLtJ
NZO/HlLtJ
PWK/PhJ
WSB/EiJ
Mean value
Animal shipping date
0
50
100
150
200
2013−01−15
2013−04−16
2013−07−16
2013−10−14
2014−01−13

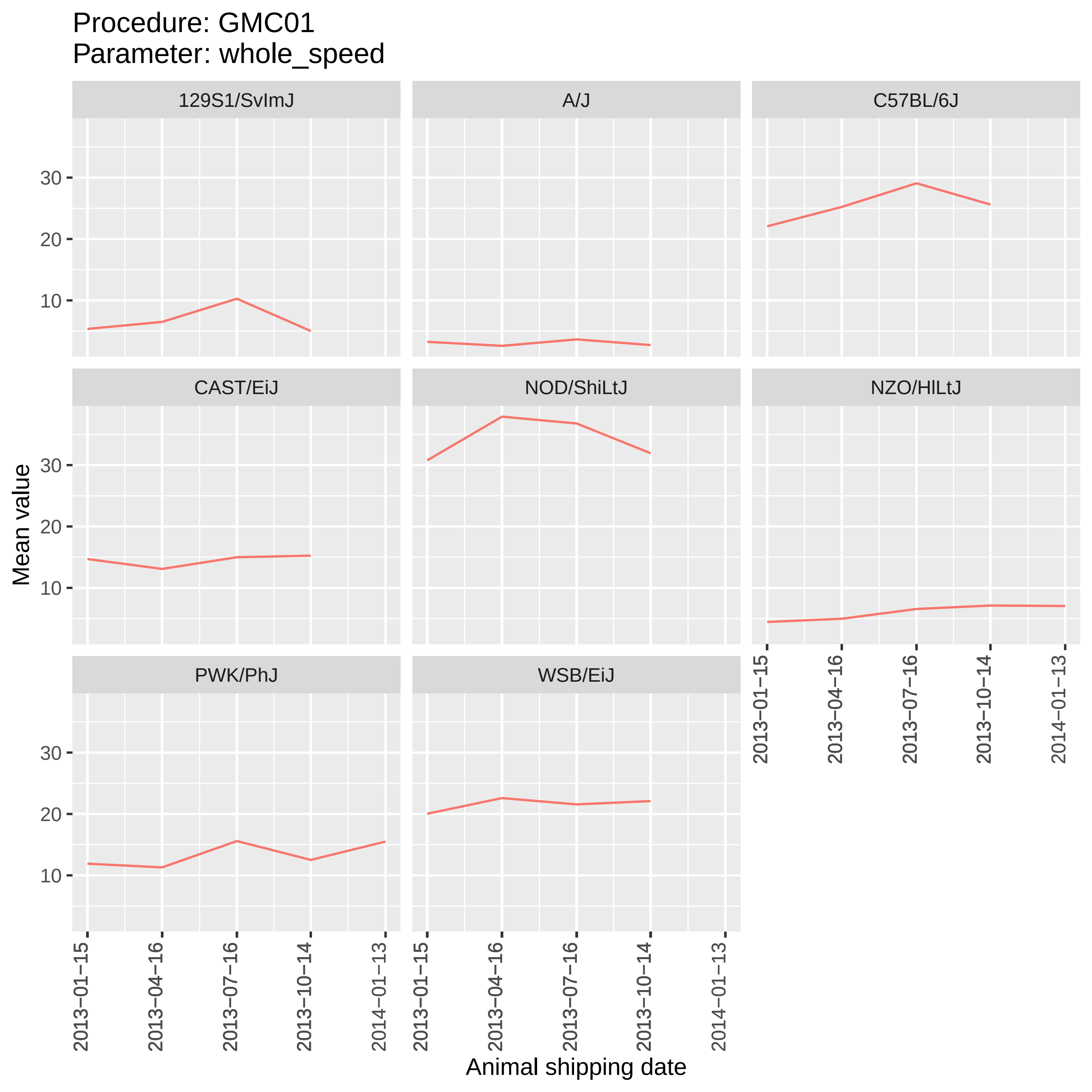
Procedure: GMC01
Parameter: whole_speed
129S1/SvImJ
A/J
C57BL/6J
CAST/EiJ
NOD/ShiLtJ
NZO/HlLtJ
PWK/PhJ
WSB/EiJ
Mean value
10
20
30
2013−01−15
2013−04−16
2013−07−16
2013−10−14
2014−01−13
Animal shipping date

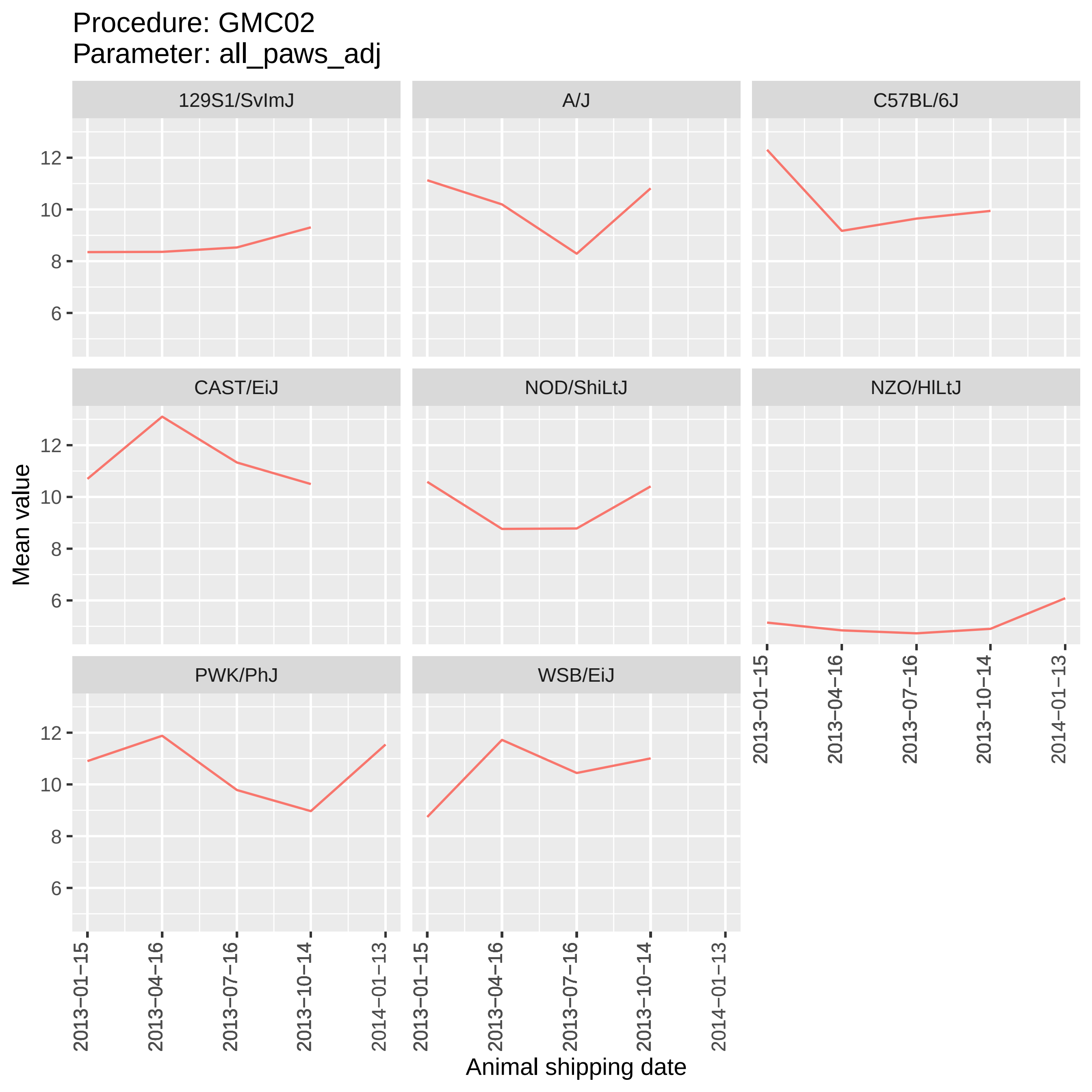

Procedure: GMC02
Parameter: all_paws_adj
129S1/SvImJ
A/J
C57BL/6J
CAST/EiJ
NOD/ShiLtJ
NZO/HlLtJ
PWK/PhJ
WSB/EiJ
Mean value
12
10
8
6
2013−01−15
2013−04−16
2013−07−16
2013−10−14
2014−01−13
Animal shipping date

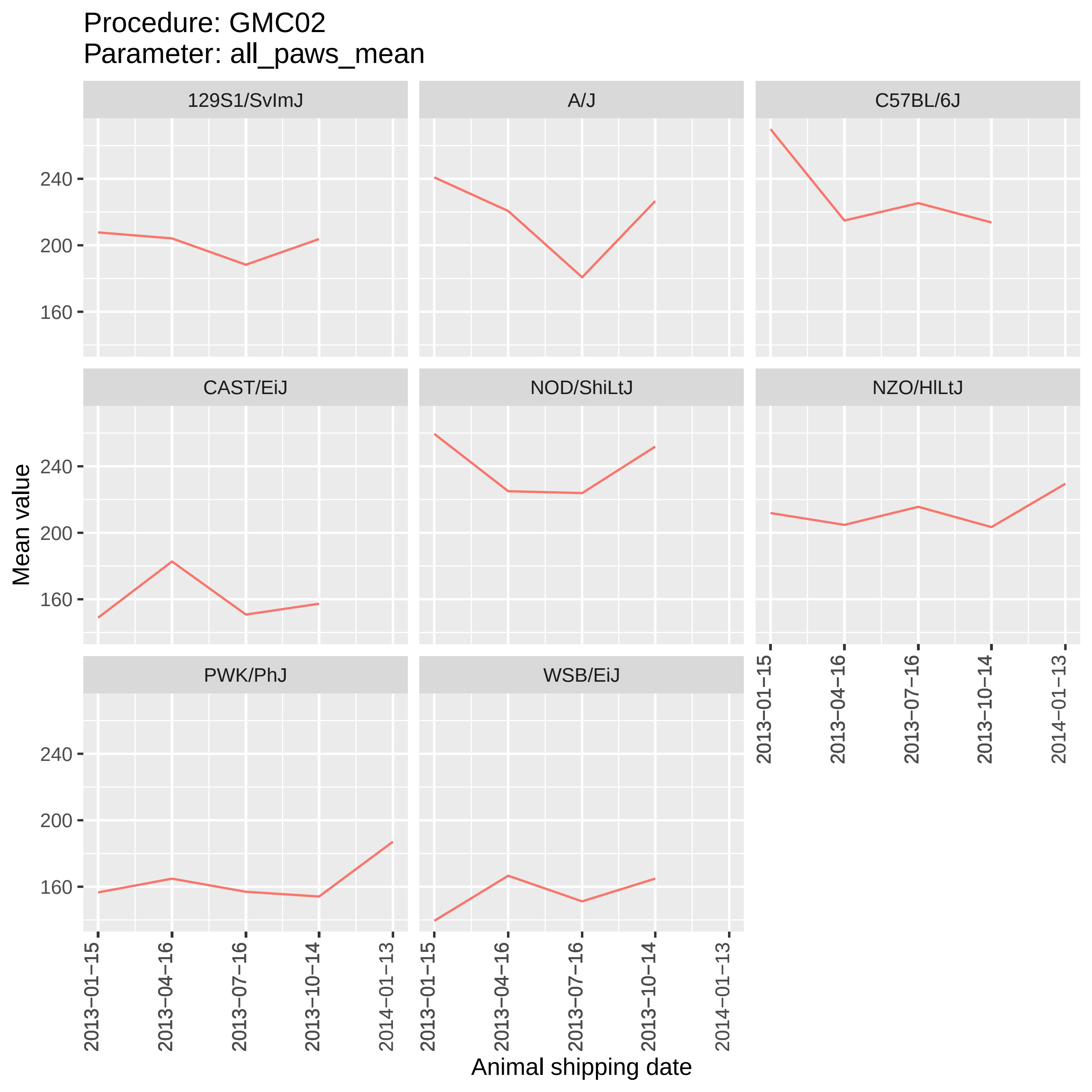
Procedure: GMC02
Parameter: all_paws_mean
129S1/SvImJ
A/J
C57BL/6J
CAST/EiJ
NOD/ShiLtJ
NZO/HlLtJ
PWK/PhJ
WSB/EiJ
Mean value
240
200
160
2013−01−15
2013−04−16
2013−07−16
2013−10−14
2014−01−13
Animal shipping date

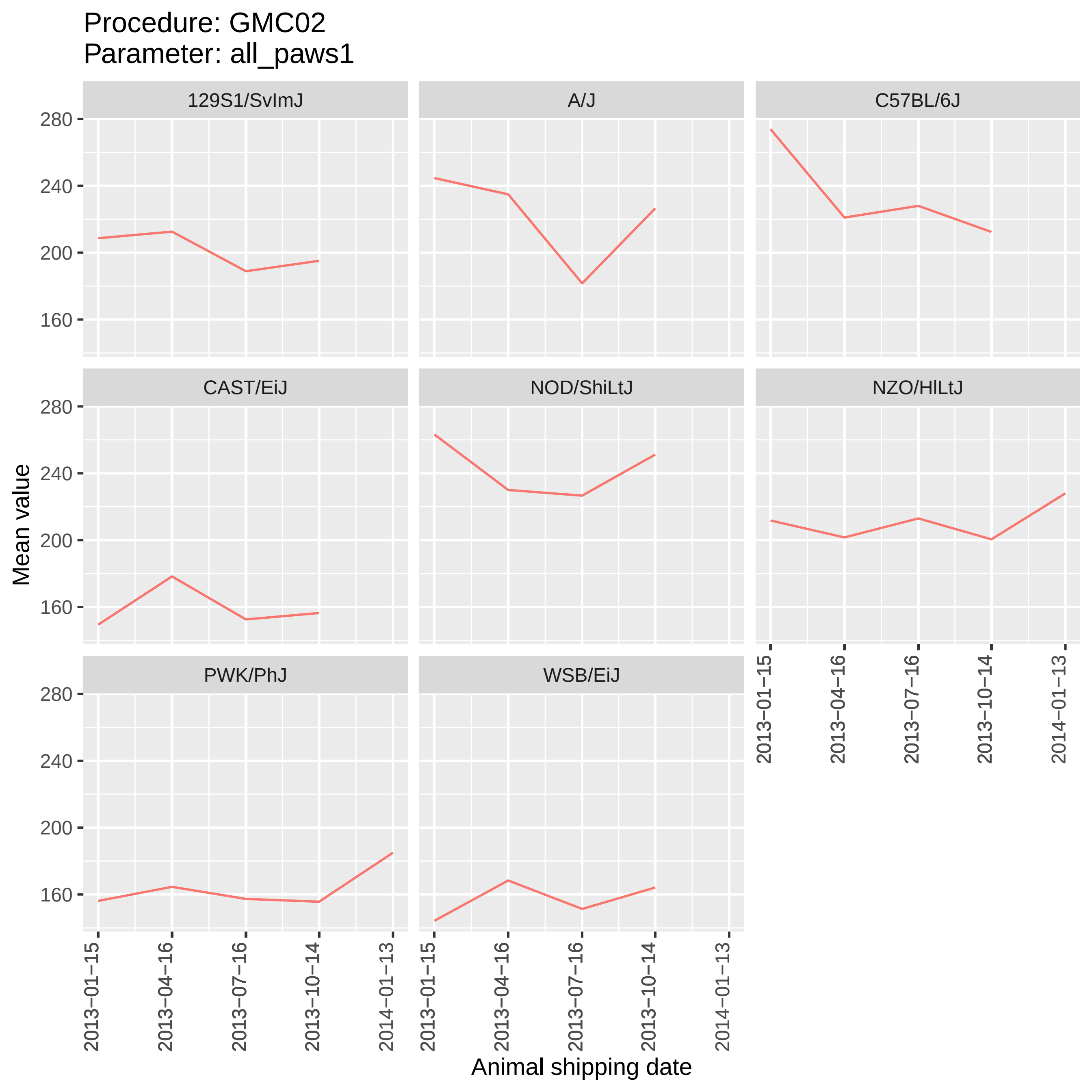

Procedure: GMC02
Parameter: all_paws1
129S1/SvImJ
A/J
C57BL/6J
CAST/EiJ
NOD/ShiLtJ
NZO/HlLtJ
PWK/PhJ
WSB/EiJ
Mean value
280
240
200
160
2013−01−15
2013−04−16
2013−07−16
2013−10−14
2014−01−13
Animal shipping date

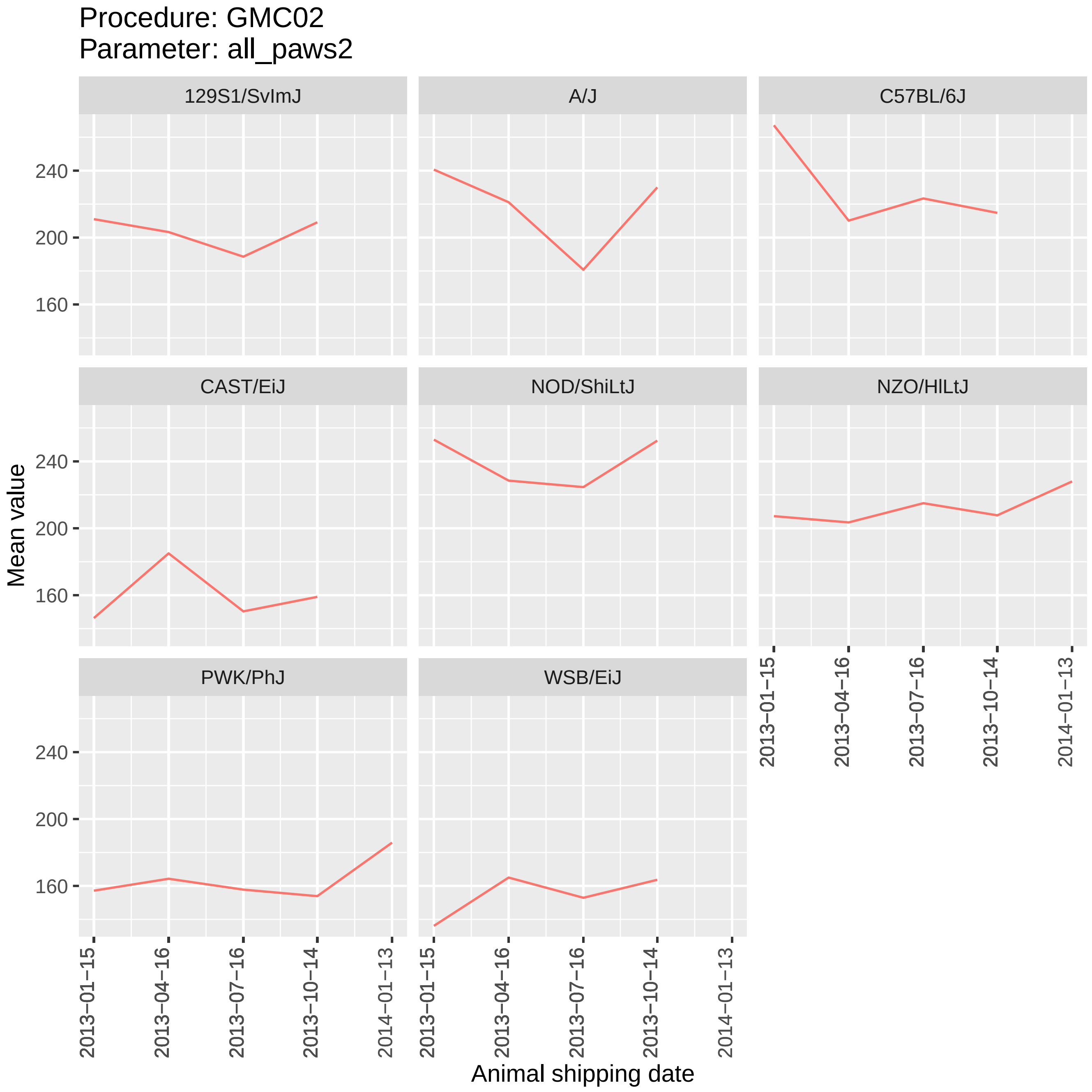
Procedure: GMC02
Parameter: all_paws2
129S1/SvImJ
A/J
C57BL/6J
CAST/EiJ
NOD/ShiLtJ
NZO/HlLtJ
PWK/PhJ
WSB/EiJ
Mean value
160
200
240
2013−01−15
2013−04−16
2013−07−16
2013−10−14
2014−01−13
Animal shipping date

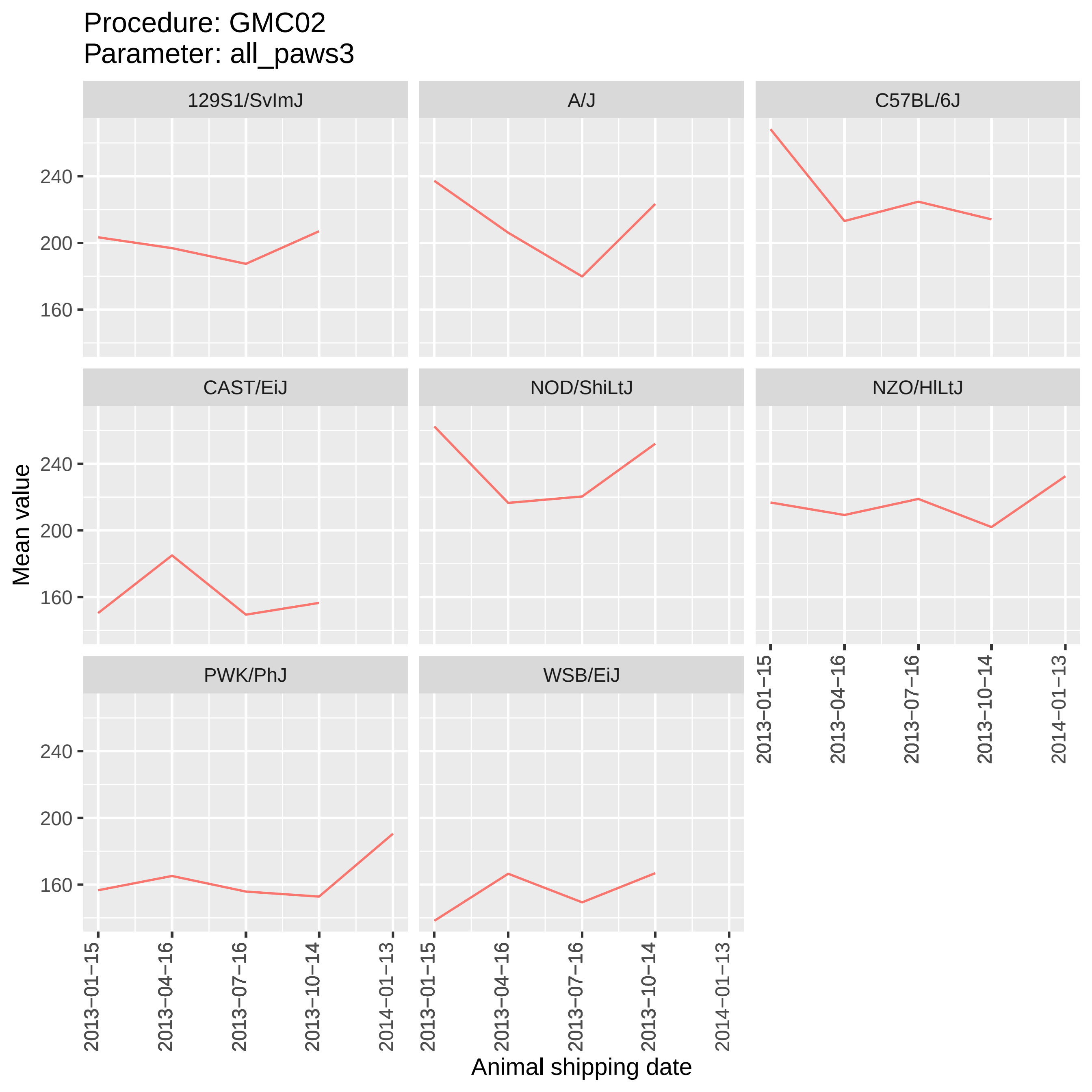

Procedure: GMC02
Parameter: all_paws3
129S1/SvImJ
A/J
C57BL/6J
CAST/EiJ
NOD/ShiLtJ
NZO/HlLtJ
PWK/PhJ
WSB/EiJ
Mean value
160
200
240
2013−01−15
2013−04−16
2013−07−16
2013−10−14
2014−01−13
Animal shipping date

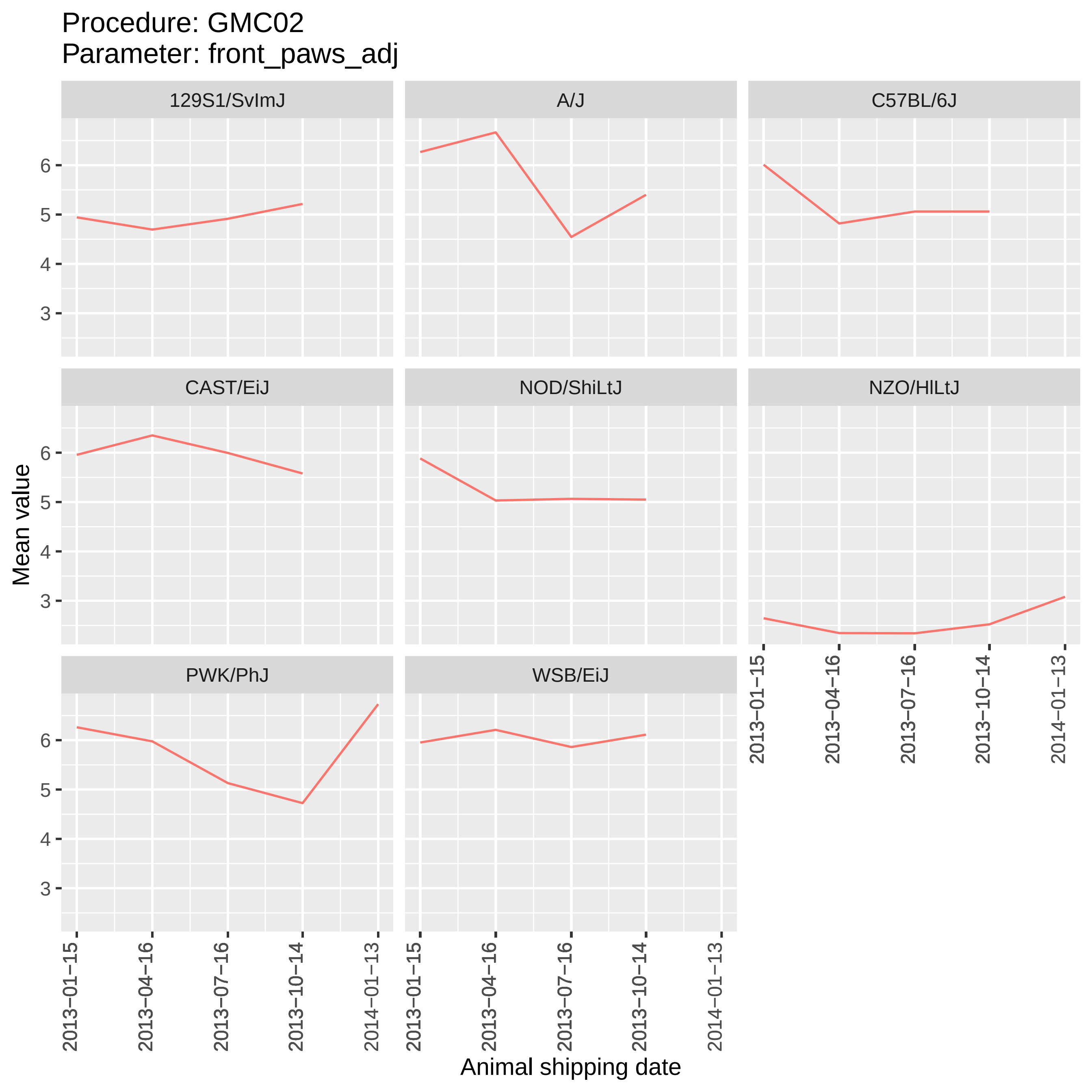

Procedure: GMC02
Parameter: front_paws_adj
129S1/SvImJ
A/J
C57BL/6J
CAST/EiJ
NOD/ShiLtJ
NZO/HlLtJ
PWK/PhJ
WSB/EiJ
Mean value
3
4
5
6
2013−01−15
2013−04−16
2013−07−16
2013−10−14
2014−01−13
Animal shipping date

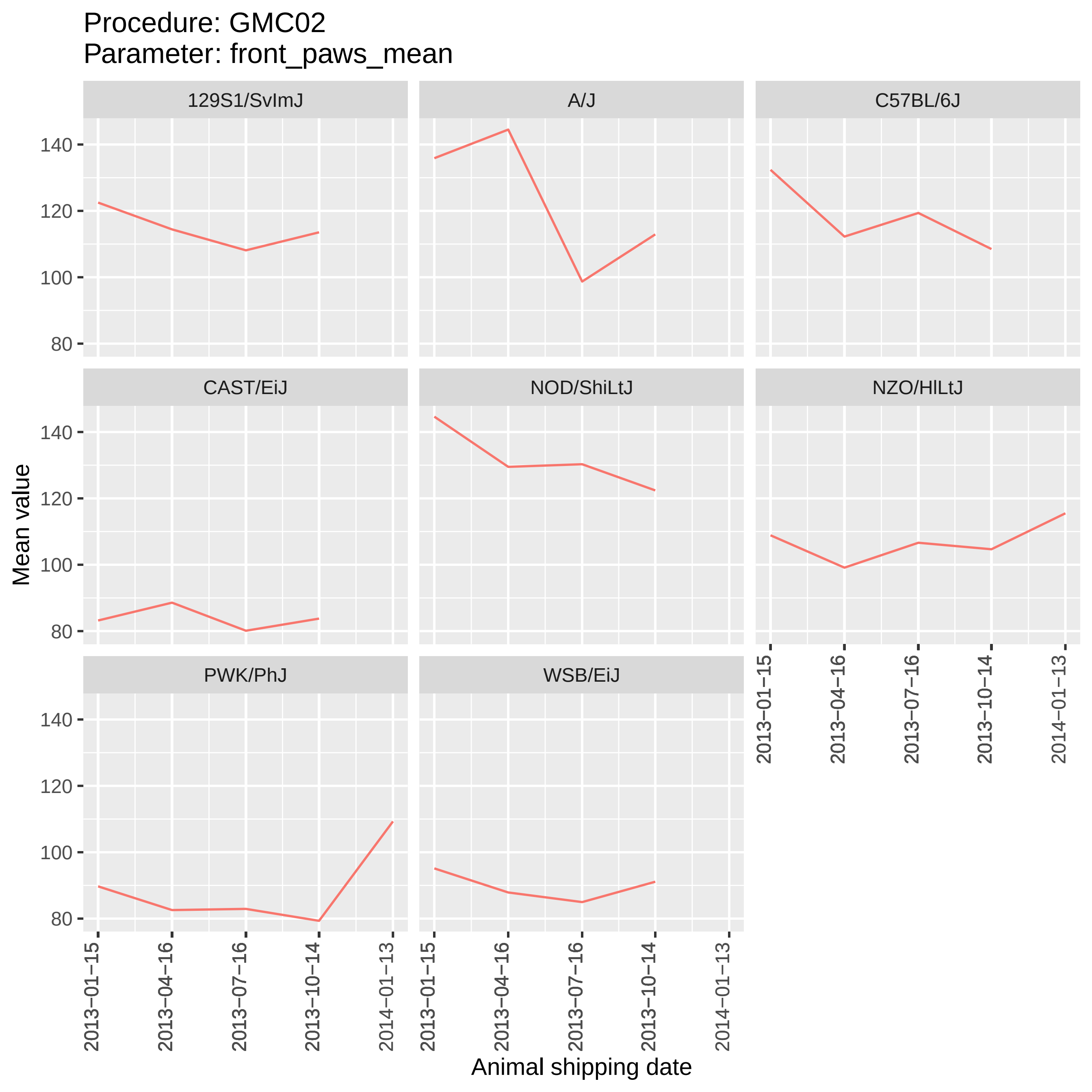
Procedure: GMC02
Parameter: front_paws_mean
129S1/SvImJ
A/J
C57BL/6J
CAST/EiJ
NOD/ShiLtJ
NZO/HlLtJ
PWK/PhJ
WSB/EiJ
Mean value
80
100
120
140
2013−01−15
2013−04−16
2013−07−16
2013−10−14
2014−01−13
Animal shipping date

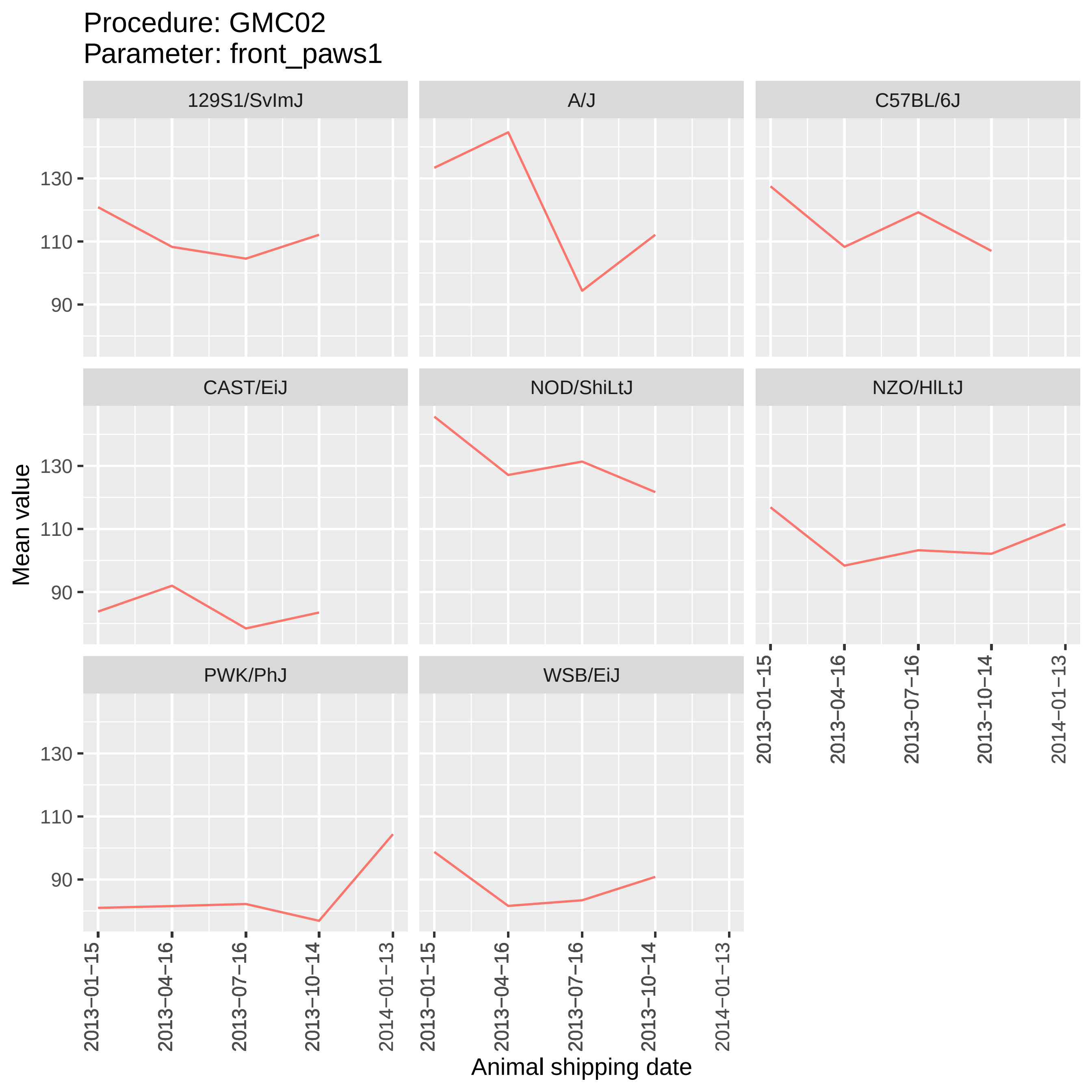

Procedure: GMC02
Parameter: front_paws1
129S1/SvImJ
A/J
C57BL/6J
CAST/EiJ
NOD/ShiLtJ
NZO/HlLtJ
PWK/PhJ
WSB/EiJ
Mean value
130
110
90
2013−01−15
2013−04−16
2013−07−16
2013−10−14
2014−01−13
Animal shipping date

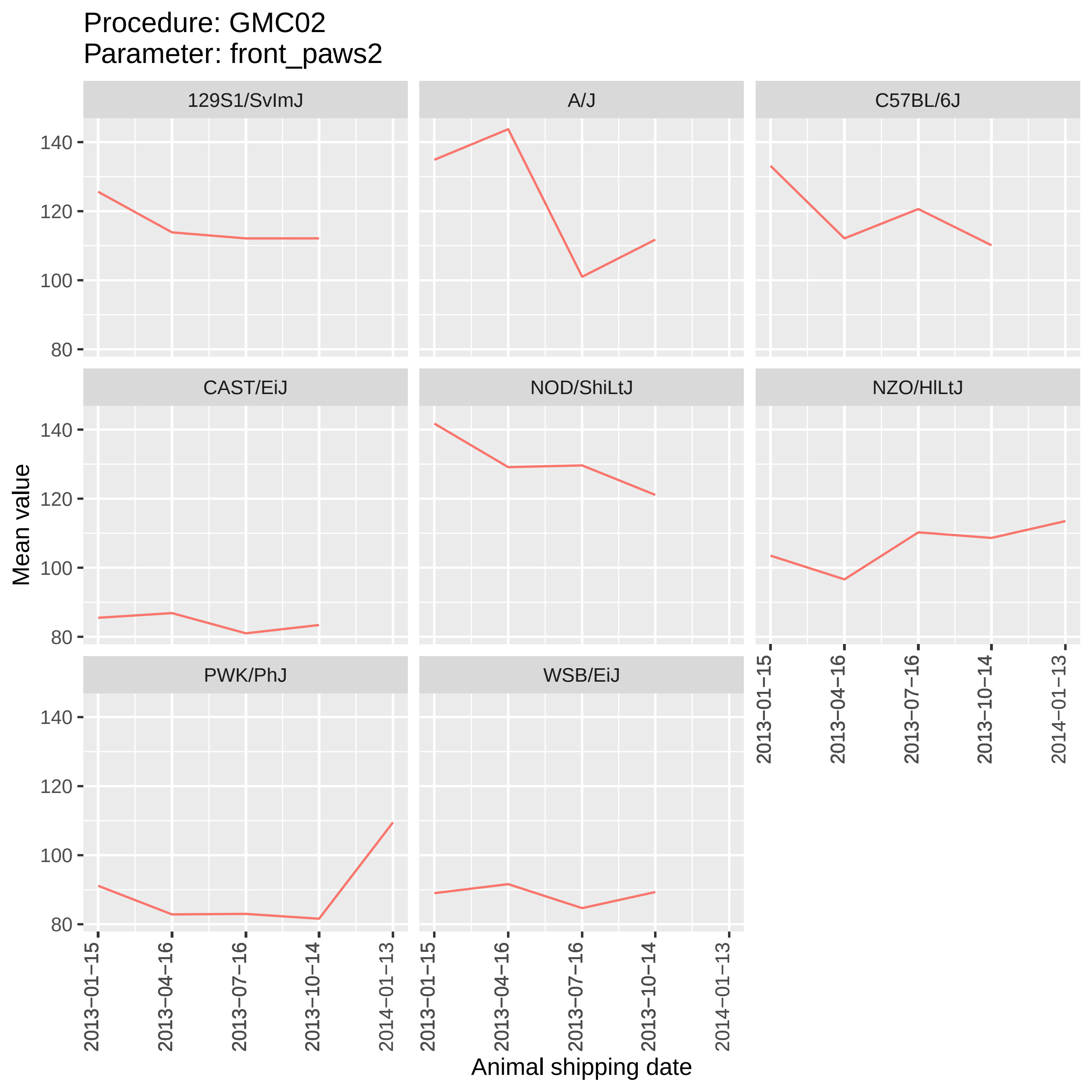
Procedure: GMC02
Parameter: front_paws2
129S1/SvImJ
A/J
C57BL/6J
CAST/EiJ
NOD/ShiLtJ
NZO/HlLtJ
PWK/PhJ
WSB/EiJ
Mean value
80
100
120
140
2013−01−15
2013−04−16
2013−07−16
2013−10−14
2014−01−13
Animal shipping date

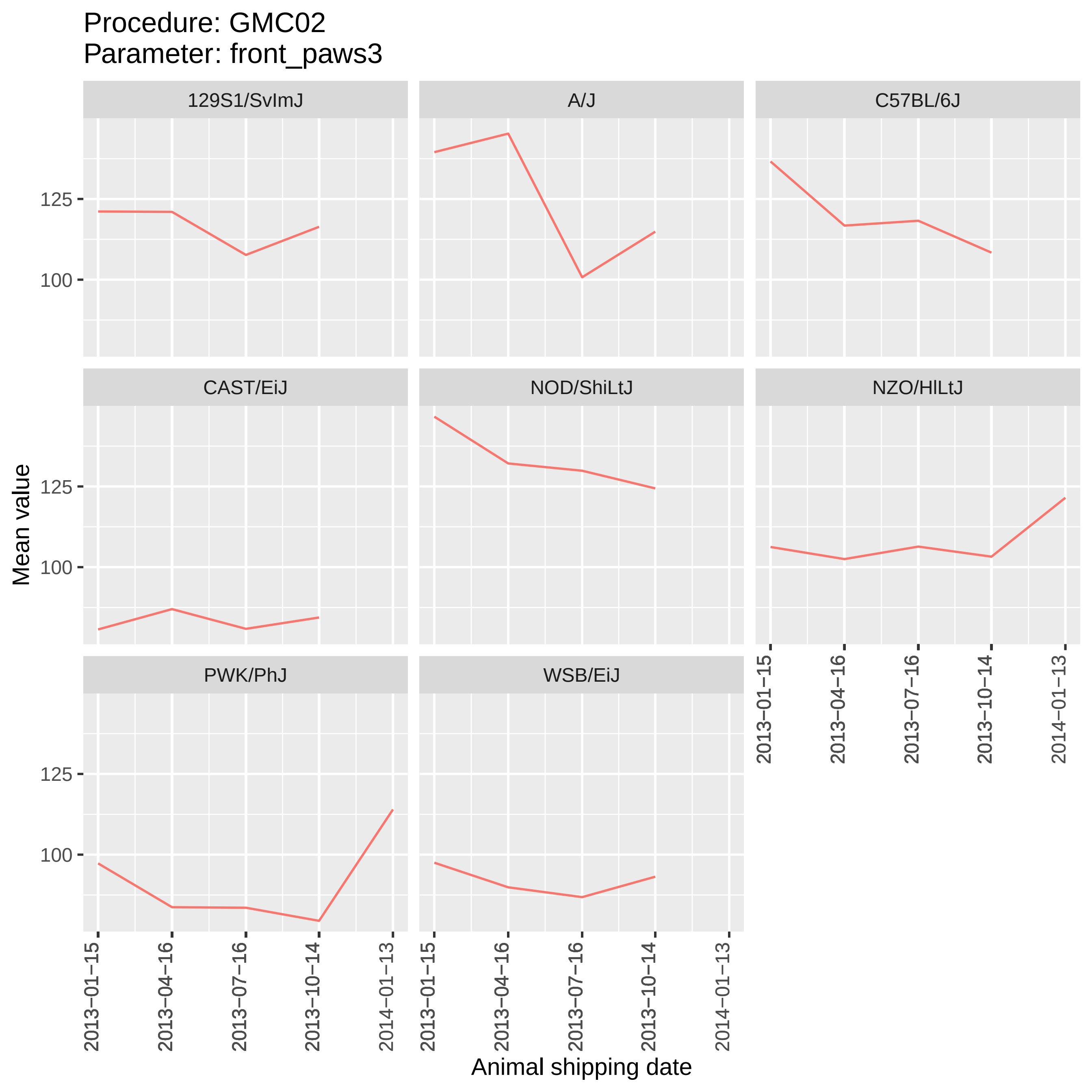
Procedure: GMC02
Parameter: front_paws3
129S1/SvImJ
A/J
C57BL/6J
CAST/EiJ
NOD/ShiLtJ
NZO/HlLtJ
PWK/PhJ
WSB/EiJ
125
100
Mean value
2013−01−15
2013−04−16
2013−07−16
2013−10−14
2014−01−13
Animal shipping date

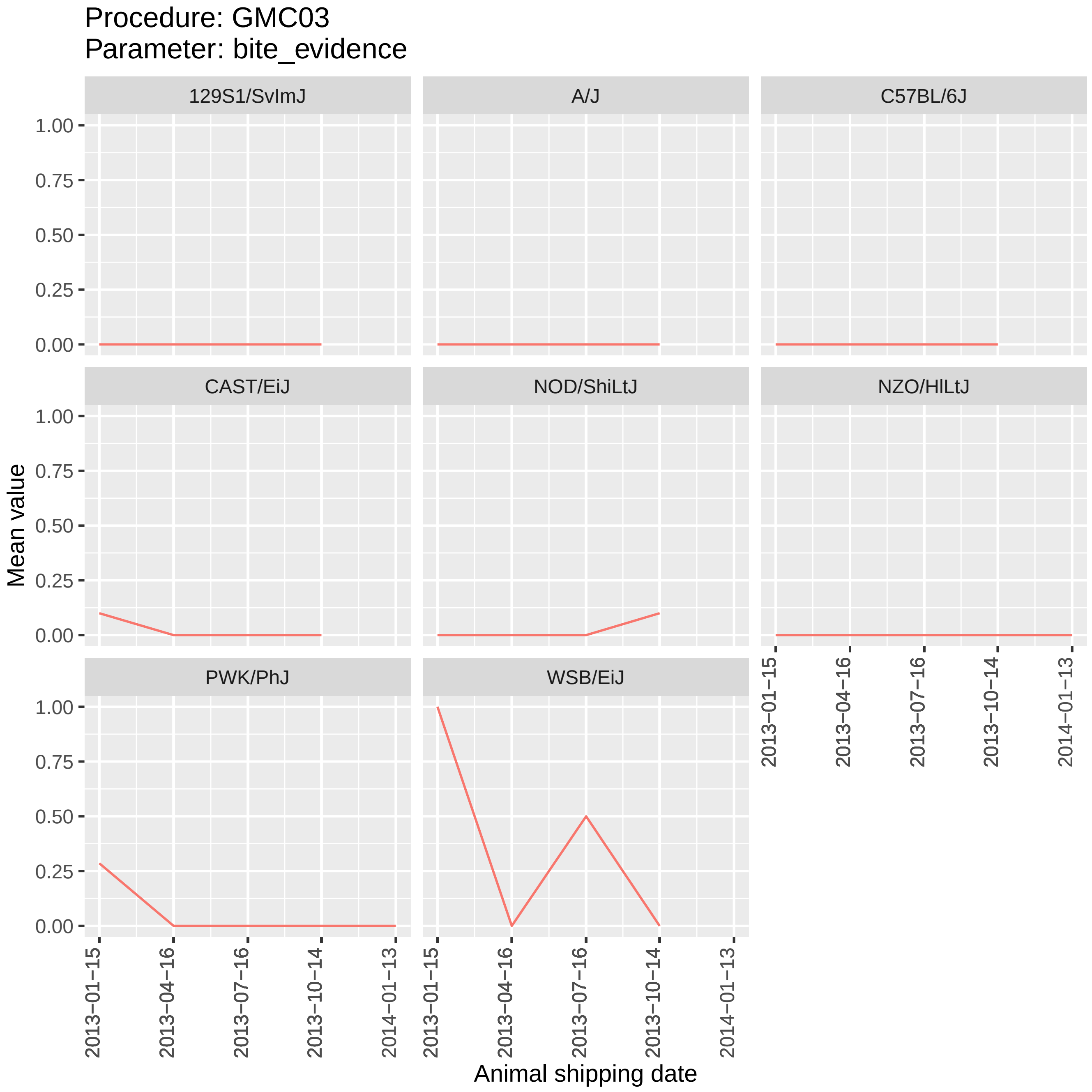
Procedure: GMC03
Parameter: bite_evidence
129S1/SvImJ
A/J
C57BL/6J
CAST/EiJ
NOD/ShiLtJ
NZO/HlLtJ
PWK/PhJ
WSB/EiJ
Mean value
1.00
0.75
0.50
0.25
0.00
2013−01−15
2013−04−16
2013−07−16
2013−10−14
2014−01−13
Animal shipping date

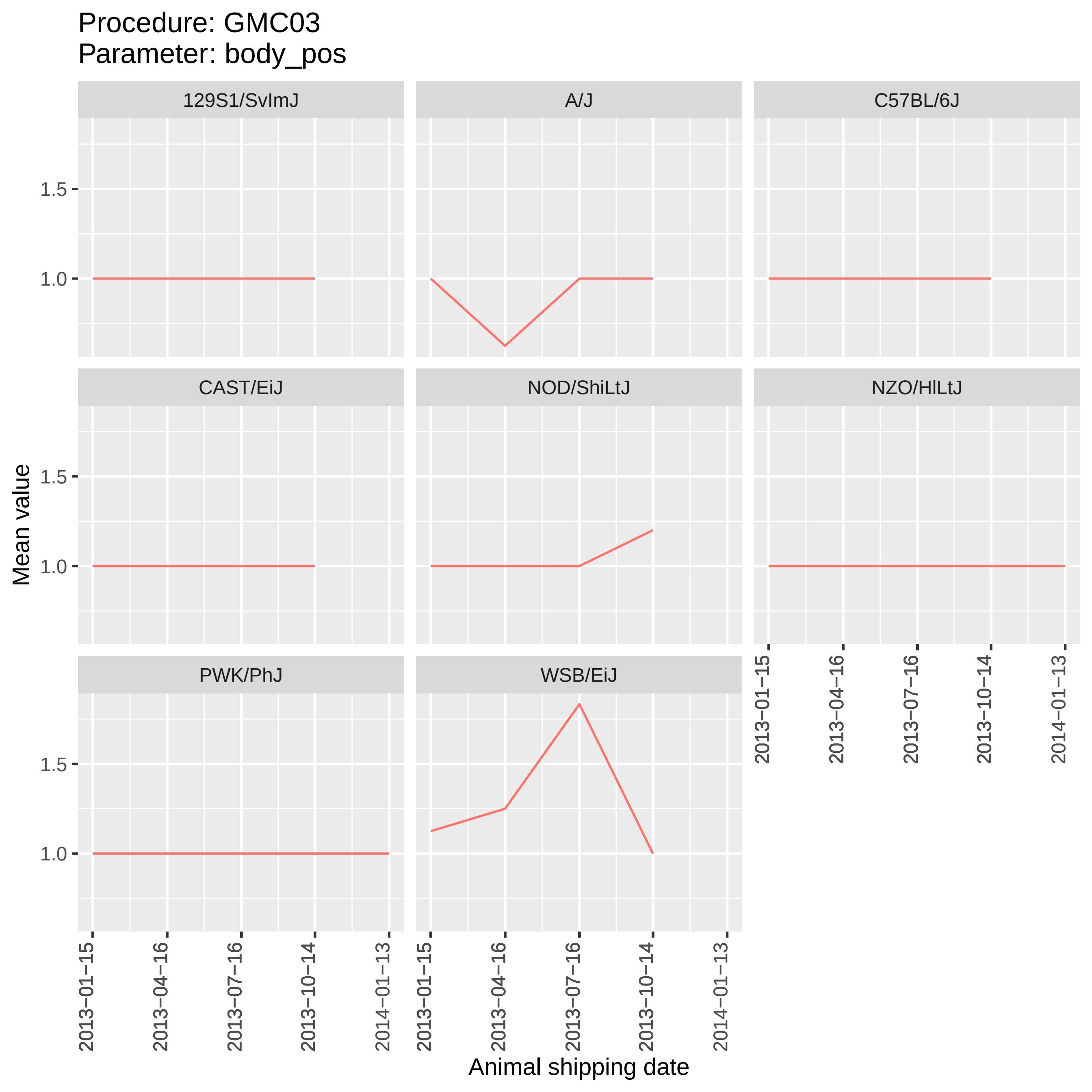

Procedure: GMC03
Parameter: body_pos
129S1/SvImJ
A/J
C57BL/6J
CAST/EiJ
NOD/ShiLtJ
NZO/HlLtJ
PWK/PhJ
WSB/EiJ
1.5
1.0
Mean value
2013−01−15
2013−04−16
2013−07−16
2013−10−14
2014−01−13
Animal shipping date

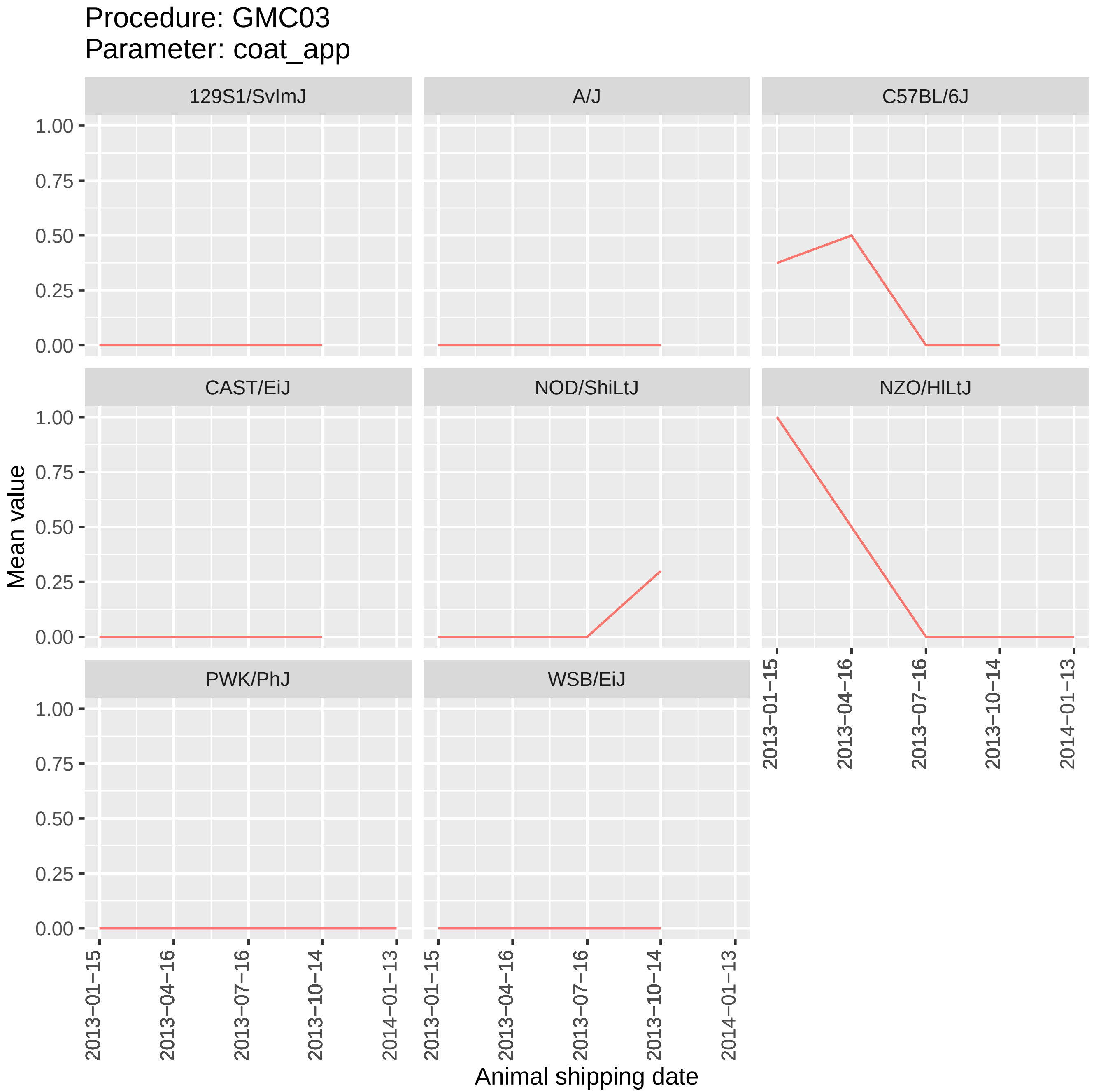

Procedure: GMC03
Parameter: coat_app
129S1/SvImJ
A/J
C57BL/6J
CAST/EiJ
NOD/ShiLtJ
NZO/HlLtJ
PWK/PhJ
WSB/EiJ
1.00
0.75
0.50
0.25
0.00
2013−01−15
2013−04−16
2013−07−16
2013−10−14
2014−01−13
Mean value
Animal shipping date

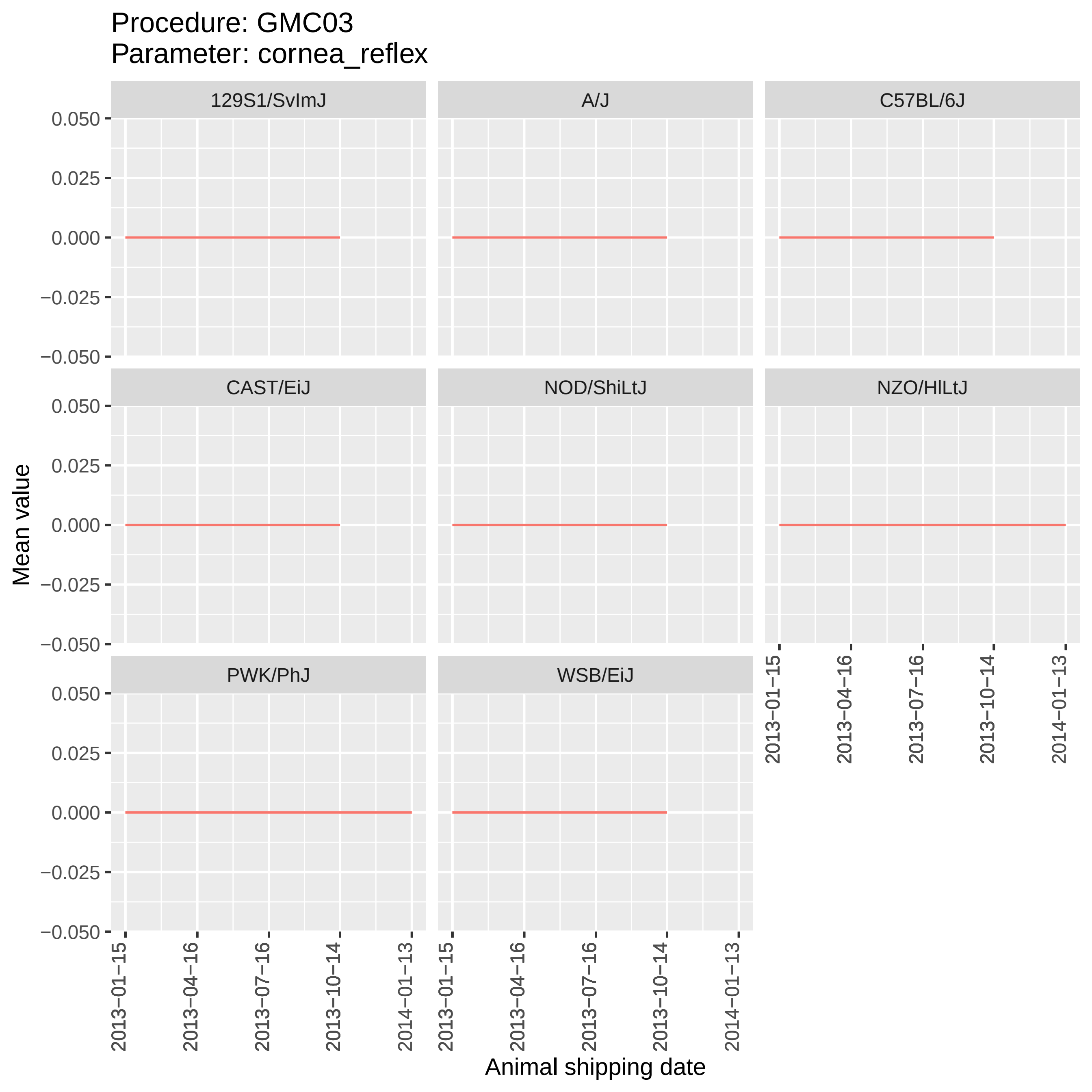
Procedure: GMC03
Parameter: cornea_reflex
129S1/SvImJ
A/J
C57BL/6J
CAST/EiJ
NOD/ShiLtJ
NZO/HlLtJ
PWK/PhJ
WSB/EiJ
0.050
0.025
0.000
−0.025
−0.050
Mean value
2013−01−15
2013−04−16
2013−07−16
2013−10−14
2014−01−13
Animal shipping date

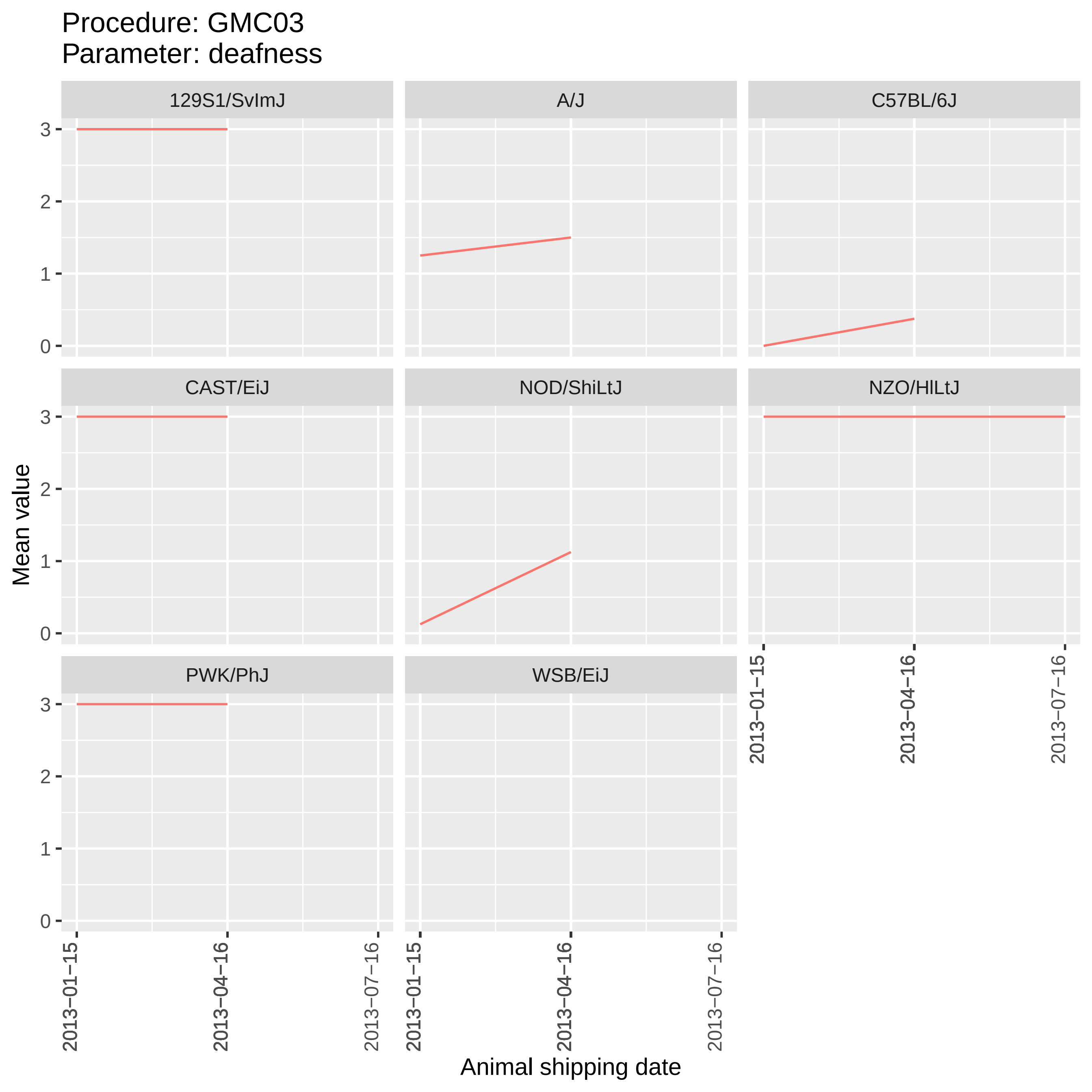

Procedure: GMC03
Parameter: deafness
129S1/SvImJ
A/J
C57BL/6J
CAST/EiJ
NOD/ShiLtJ
NZO/HlLtJ
PWK/PhJ
WSB/EiJ
Mean value
0
1
2
3
2013−01−15
2013−04−16
2013−07−16
Animal shipping date

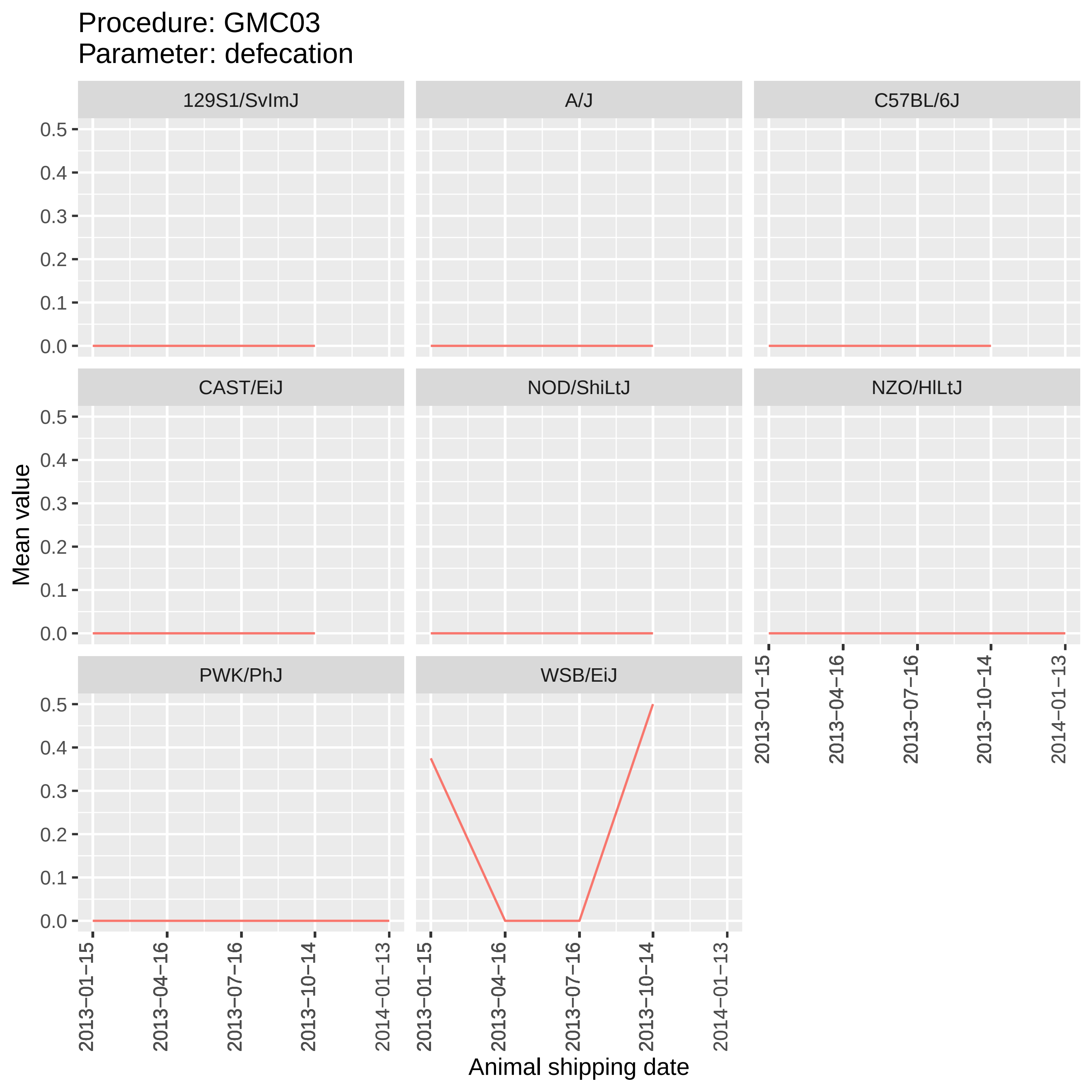

Procedure: GMC03
Parameter: defecation
129S1/SvImJ
A/J
C57BL/6J
CAST/EiJ
NOD/ShiLtJ
NZO/HlLtJ
PWK/PhJ
WSB/EiJ
Mean value
0.0
0.1
0.2
0.3
0.4
0.5
2013−01−15
2013−04−16
2013−07−16
2013−10−14
2014−01−13
Animal shipping date

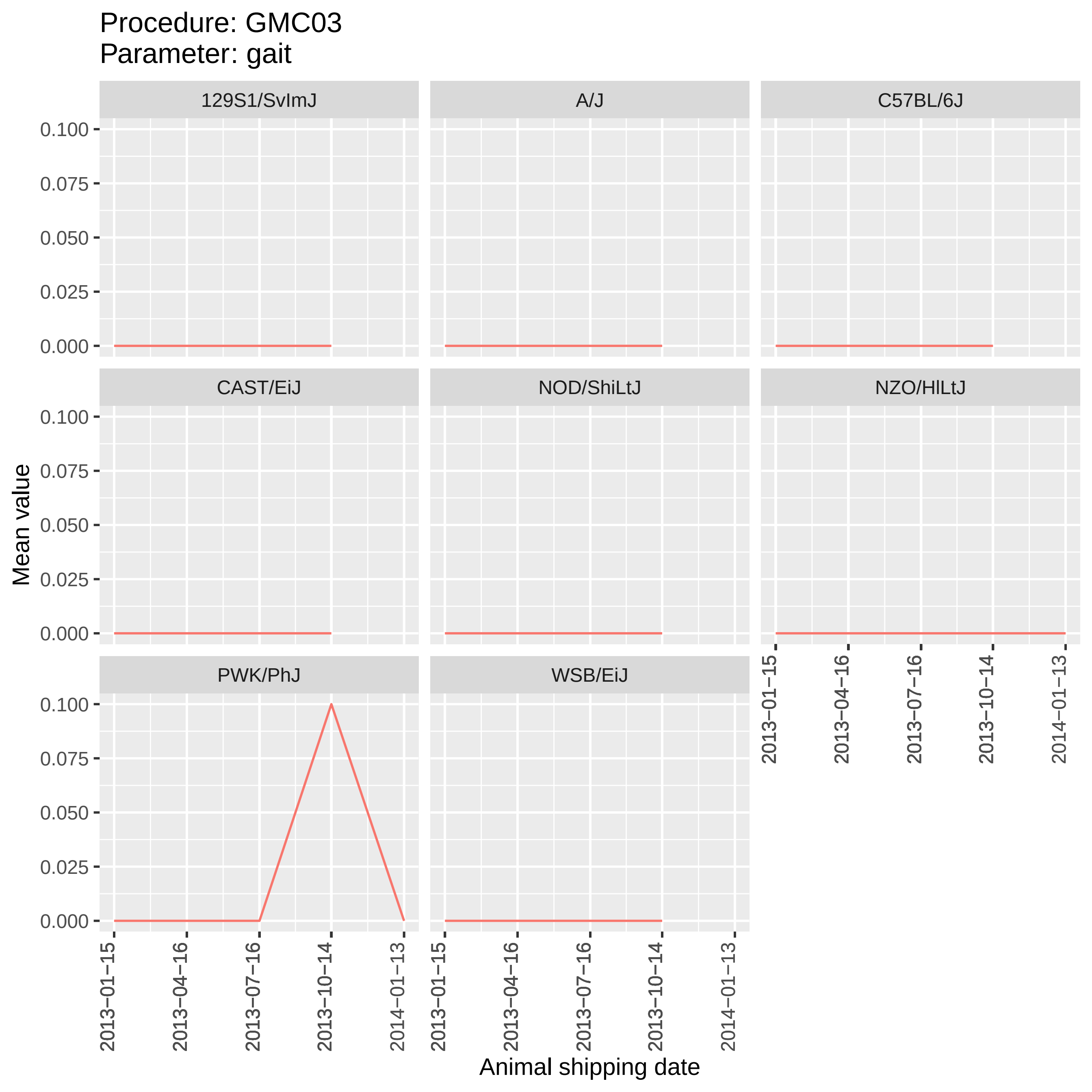

Procedure: GMC03
Parameter: gait
129S1/SvImJ
A/J
C57BL/6J
CAST/EiJ
NOD/ShiLtJ
NZO/HlLtJ
PWK/PhJ
WSB/EiJ
Mean value
0.100
0.075
0.050
0.025
0.000
2013−01−15
2013−04−16
2013−07−16
2013−10−14
2014−01−13
Animal shipping date

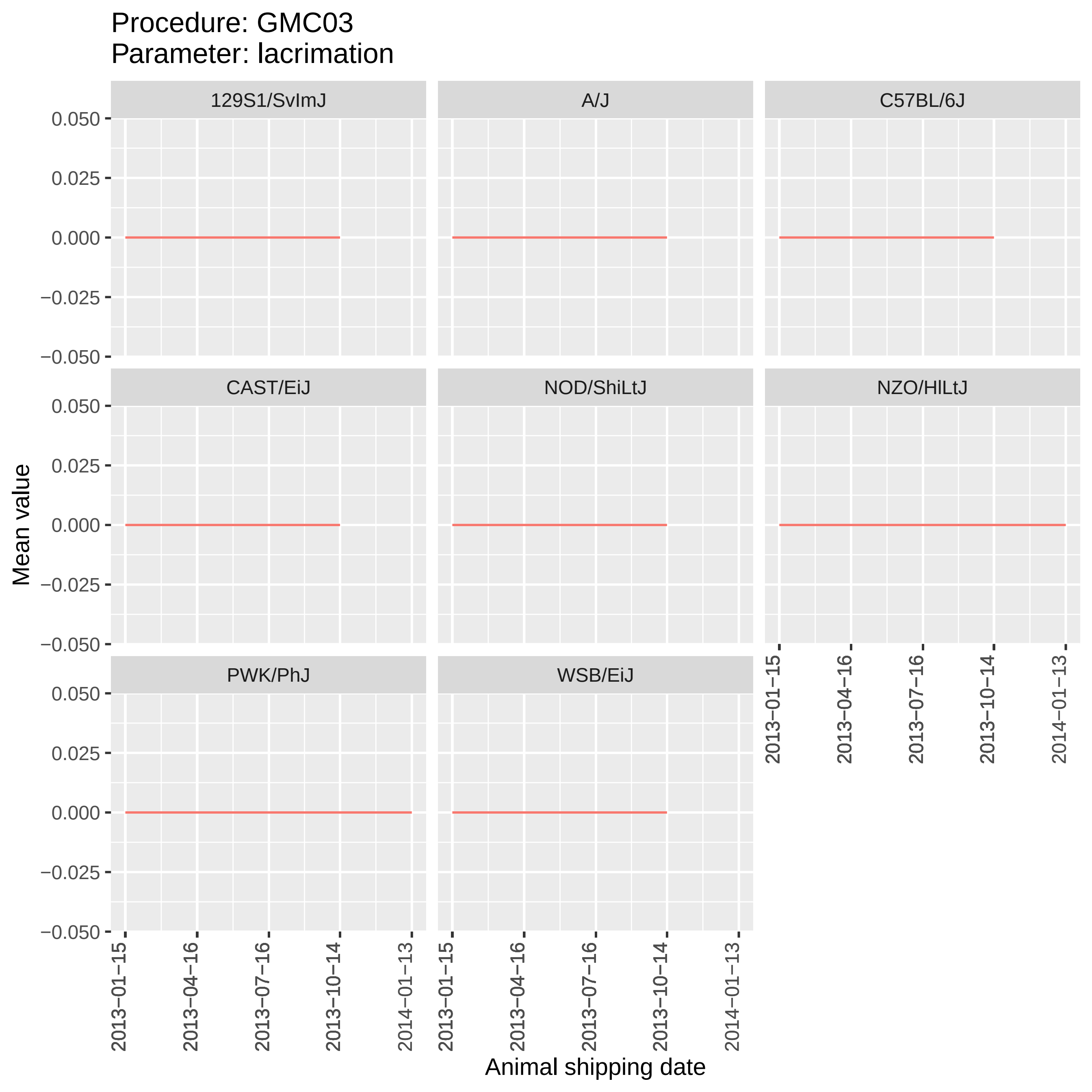

Procedure: GMC03
Parameter: lacrimation
129S1/SvImJ
A/J
C57BL/6J
CAST/EiJ
NOD/ShiLtJ
NZO/HlLtJ
PWK/PhJ
WSB/EiJ
0.050
0.025
0.000
−0.025
−0.050
Mean value
2013−01−15
2013−04−16
2013−07−16
2013−10−14
2014−01−13
Animal shipping date

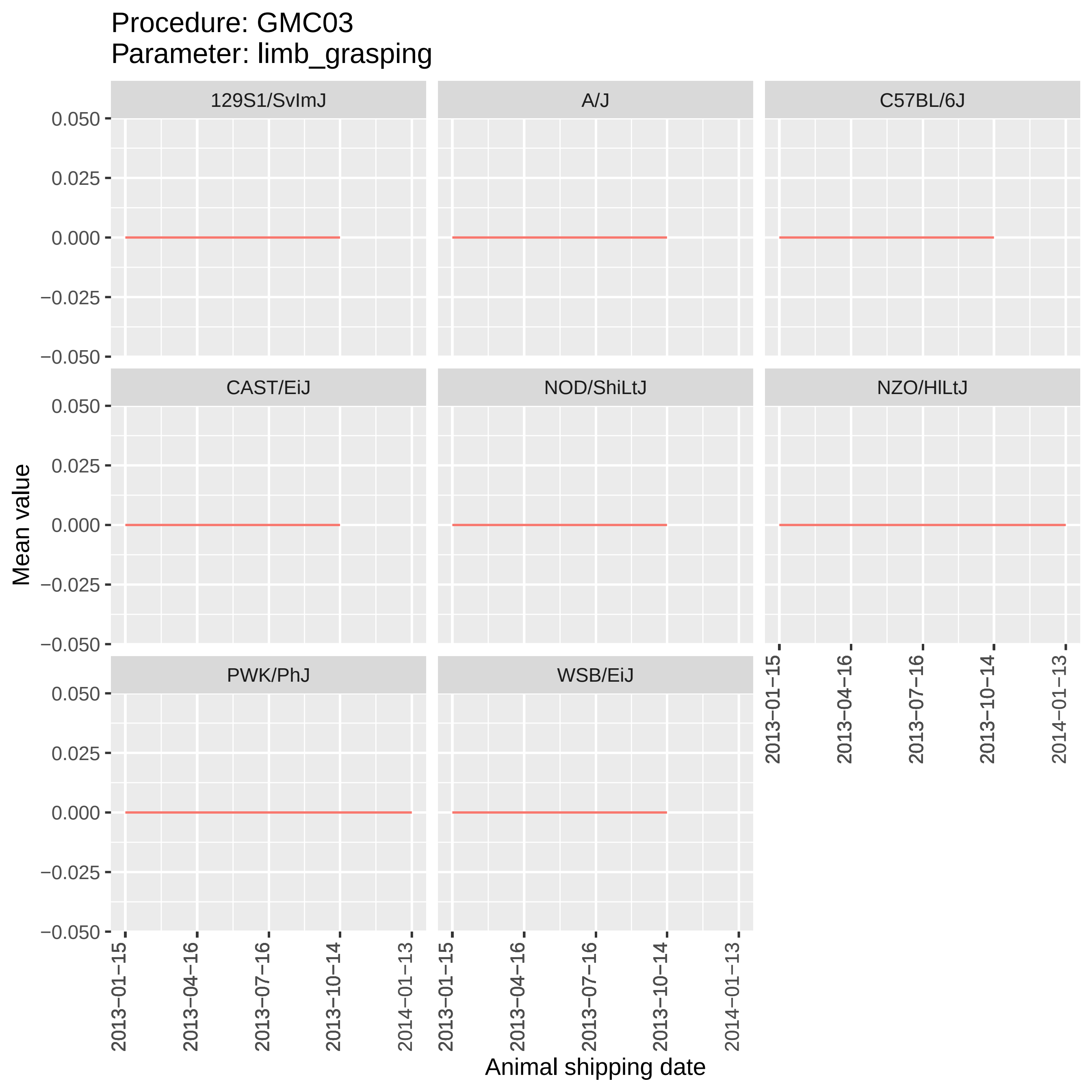
Procedure: GMC03
Parameter: limb_grasping
129S1/SvImJ
A/J
C57BL/6J
CAST/EiJ
NOD/ShiLtJ
NZO/HlLtJ
PWK/PhJ
WSB/EiJ
0.050
0.025
0.000
−0.025
−0.050
Mean value
2013−01−15
2013−04−16
2013−07−16
2013−10−14
2014−01−13
Animal shipping date

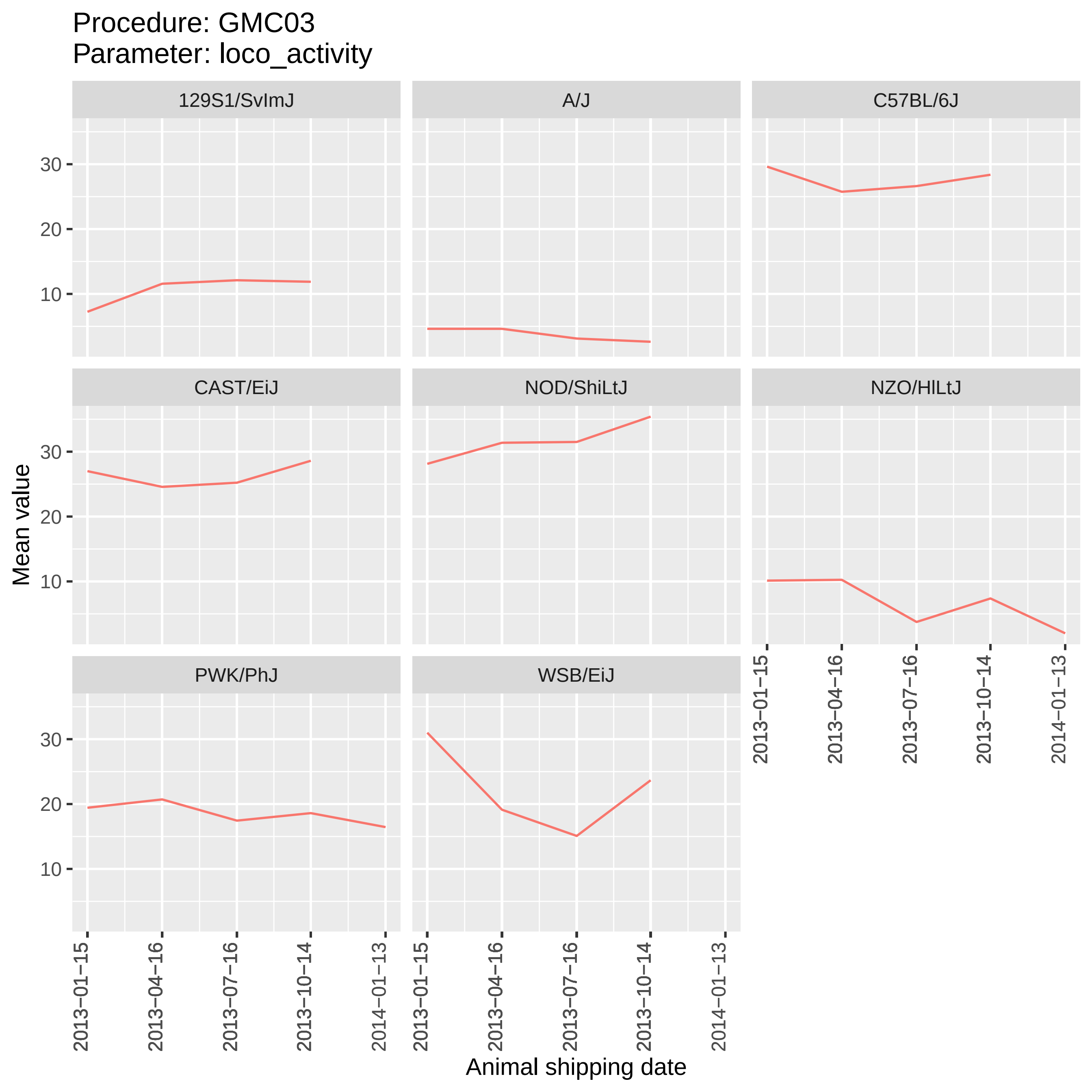

Procedure: GMC03
Parameter: loco_activity
129S1/SvImJ
A/J
C57BL/6J
CAST/EiJ
NOD/ShiLtJ
NZO/HlLtJ
PWK/PhJ
WSB/EiJ
Mean value
10
20
30
2013−01−15
2013−04−16
2013−07−16
2013−10−14
2014−01−13
Animal shipping date

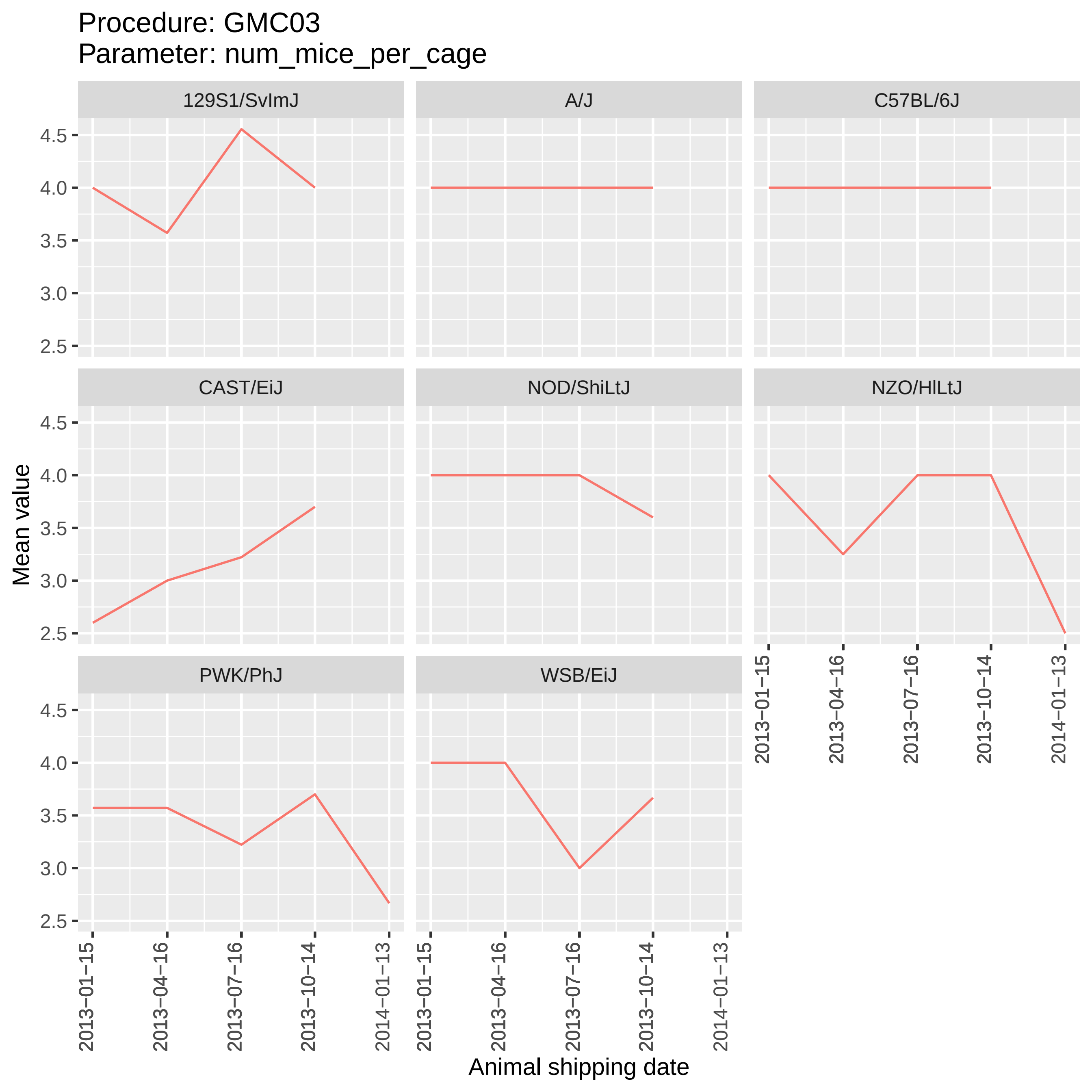

Procedure: GMC03
Parameter: num_mice_per_cage
129S1/SvImJ
A/J
C57BL/6J
CAST/EiJ
NOD/ShiLtJ
NZO/HlLtJ
PWK/PhJ
WSB/EiJ
Mean value
2.5
3.0
3.5
4.0
4.5
2013−01−15
2013−04−16
2013−07−16
2013−10−14
2014−01−13
Animal shipping date

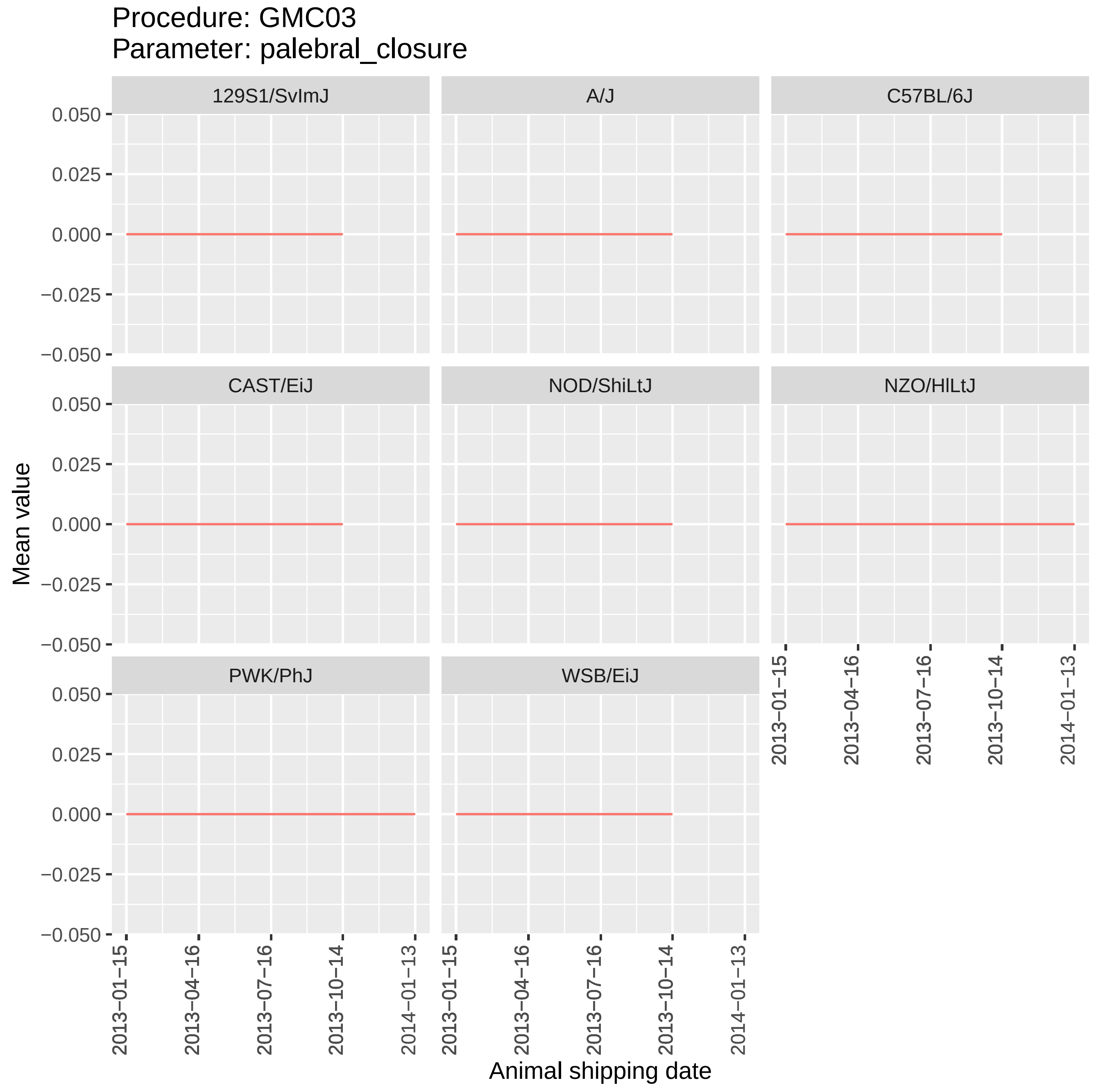

Procedure: GMC03
Parameter: palebral_closure
129S1/SvImJ
A/J
C57BL/6J
CAST/EiJ
NOD/ShiLtJ
NZO/HlLtJ
PWK/PhJ
WSB/EiJ
0.050
0.025
0.000
−0.025
−0.050
Mean value
2013−01−15
2013−04−16
2013−07−16
2013−10−14
2014−01−13
Animal shipping date

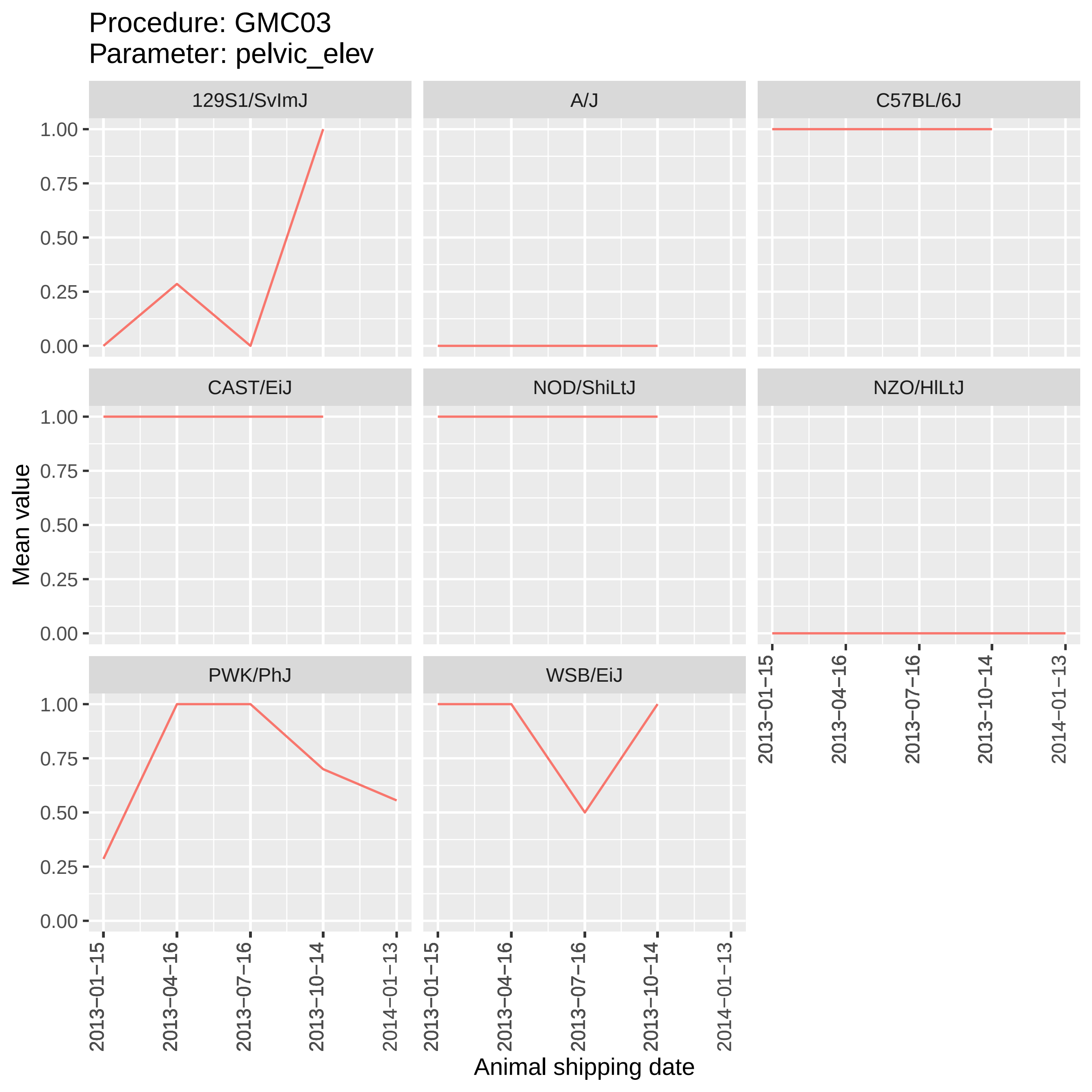

Procedure: GMC03
Parameter: pelvic_elev
129S1/SvImJ
A/J
C57BL/6J
CAST/EiJ
NOD/ShiLtJ
NZO/HlLtJ
PWK/PhJ
WSB/EiJ
1.00
0.75
0.50
0.25
0.00
Mean value
2013−01−15
2013−04−16
2013−07−16
2013−10−14
2014−01−13
Animal shipping date

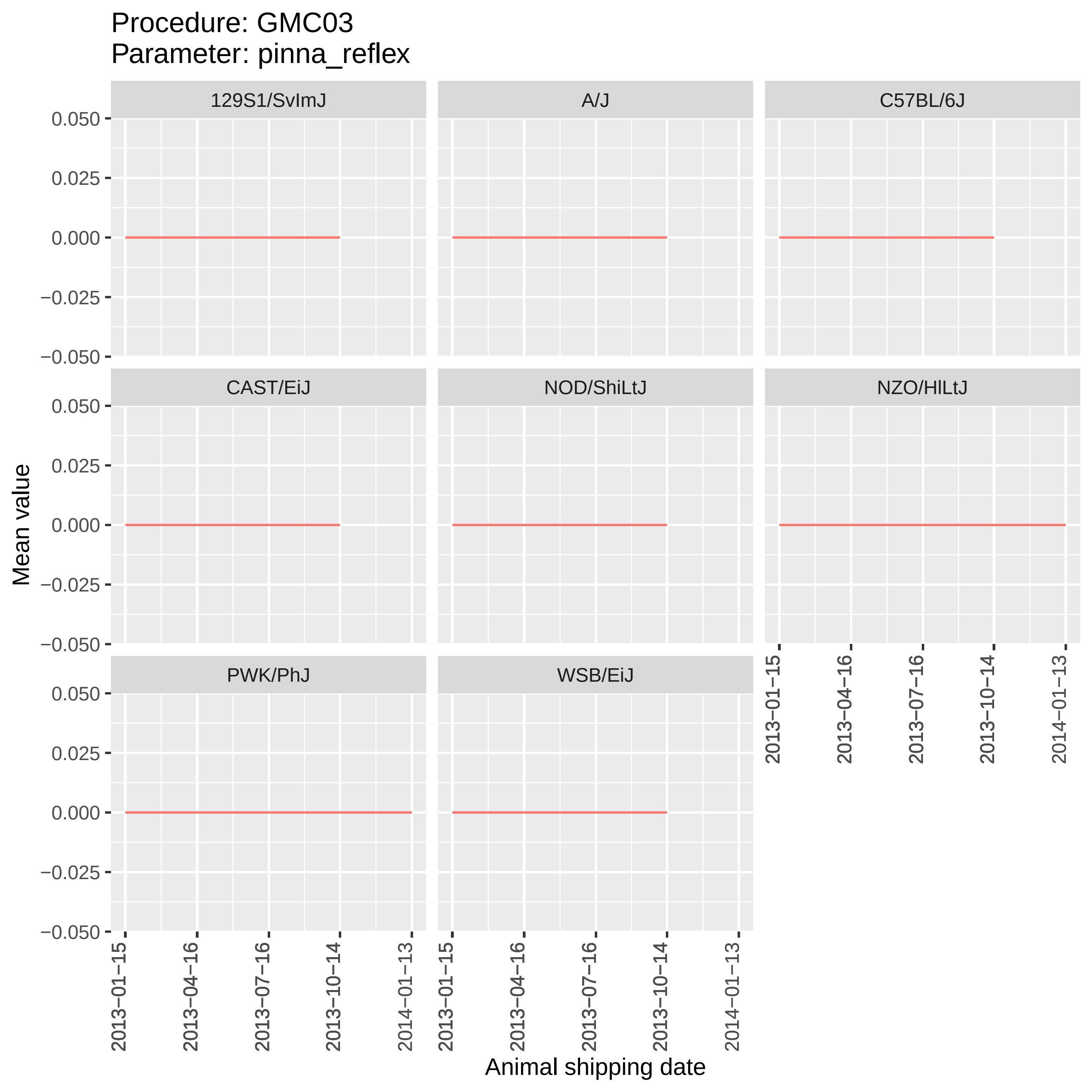
Procedure: GMC03
Parameter: pinna_reflex
129S1/SvImJ
A/J
C57BL/6J
CAST/EiJ
NOD/ShiLtJ
NZO/HlLtJ
PWK/PhJ
WSB/EiJ
0.050
0.025
0.000
−0.025
−0.050
Mean value
2013−01−15
2013−04−16
2013−07−16
2013−10−14
2014−01−13
Animal shipping date

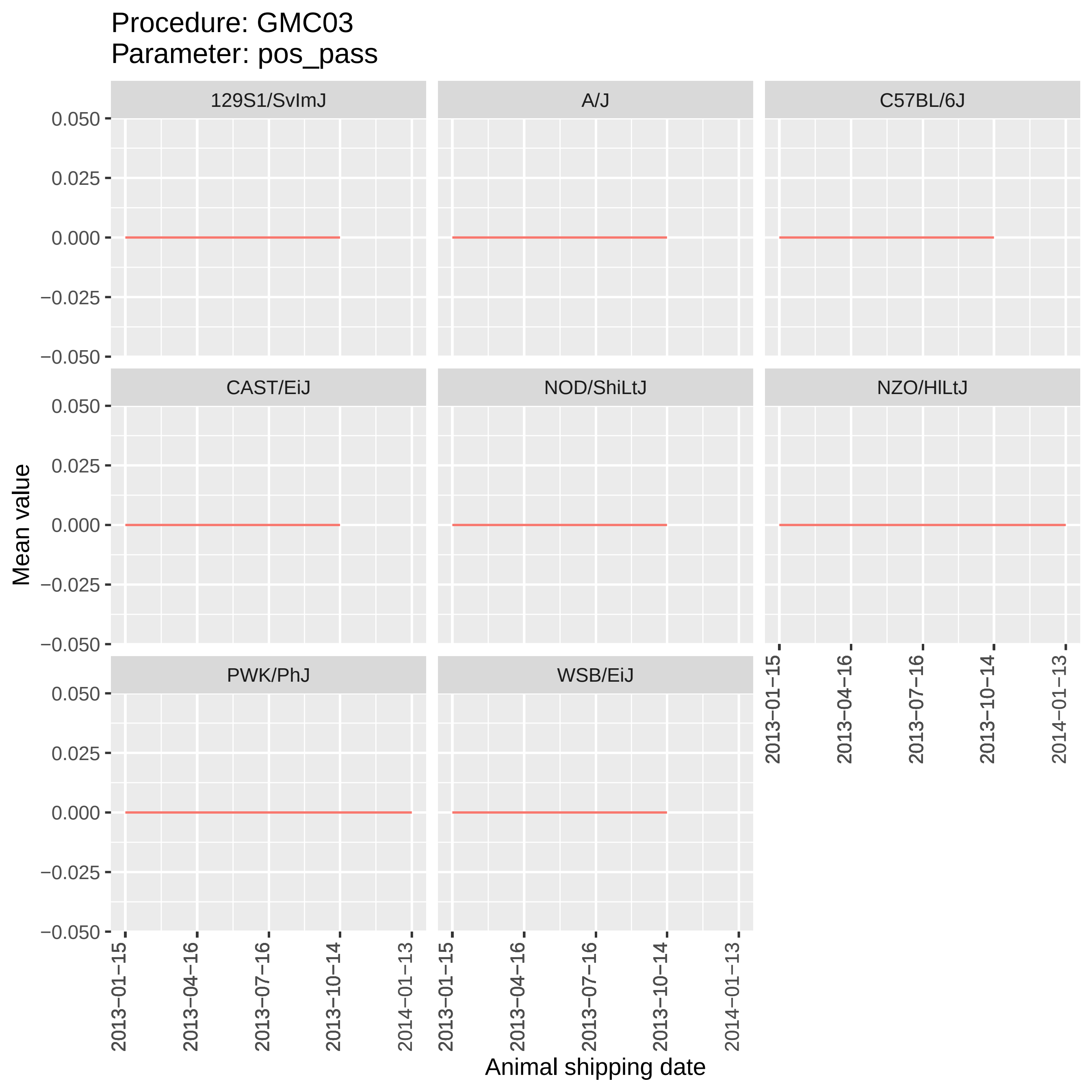
Procedure: GMC03
Parameter: pos_pass
129S1/SvImJ
A/J
C57BL/6J
CAST/EiJ
NOD/ShiLtJ
NZO/HlLtJ
PWK/PhJ
WSB/EiJ
0.050
0.025
0.000
−0.025
−0.050
Mean value
2013−01−15
2013−04−16
2013−07−16
2013−10−14
2014−01−13
Animal shipping date

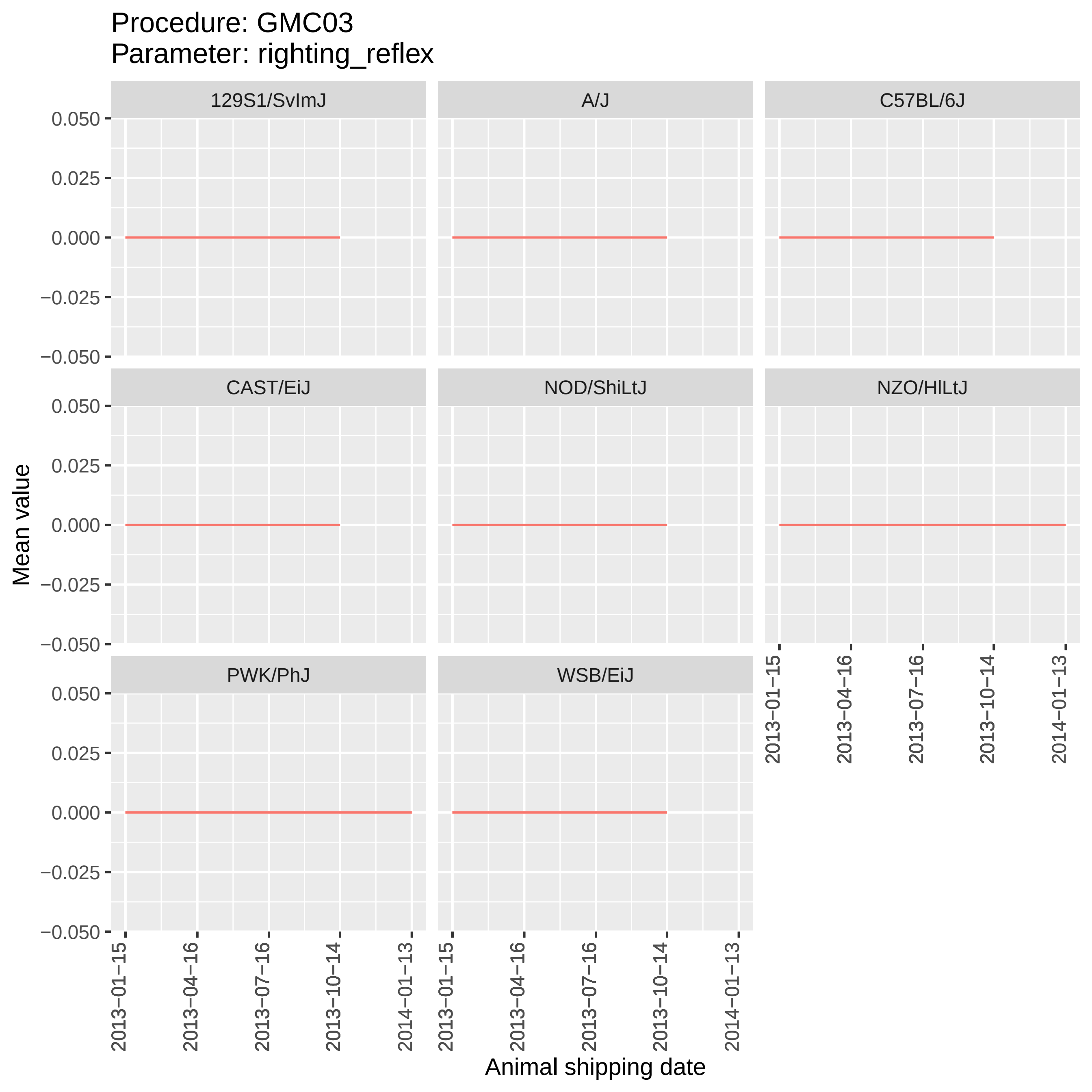

Procedure: GMC03
Parameter: righting_reflex
129S1/SvImJ
A/J
C57BL/6J
CAST/EiJ
NOD/ShiLtJ
NZO/HlLtJ
PWK/PhJ
WSB/EiJ
0.050
0.025
0.000
−0.025
−0.050
Mean value
2013−01−15
2013−04−16
2013−07−16
2013−10−14
2014−01−13
Animal shipping date

Procedure: GMC03
Parameter: startle_response

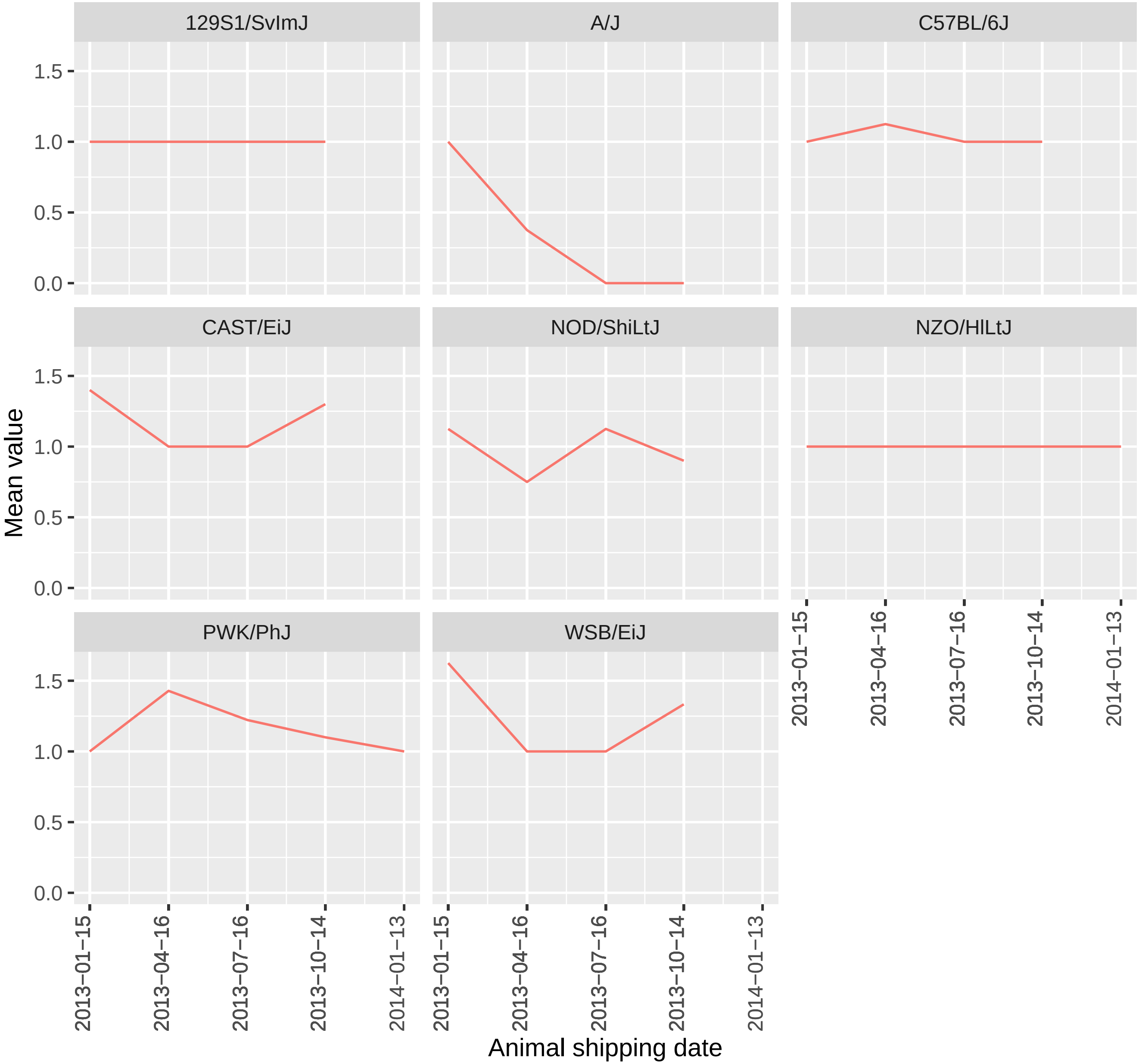

Procedure: GMC03
Parameter: tail_elev

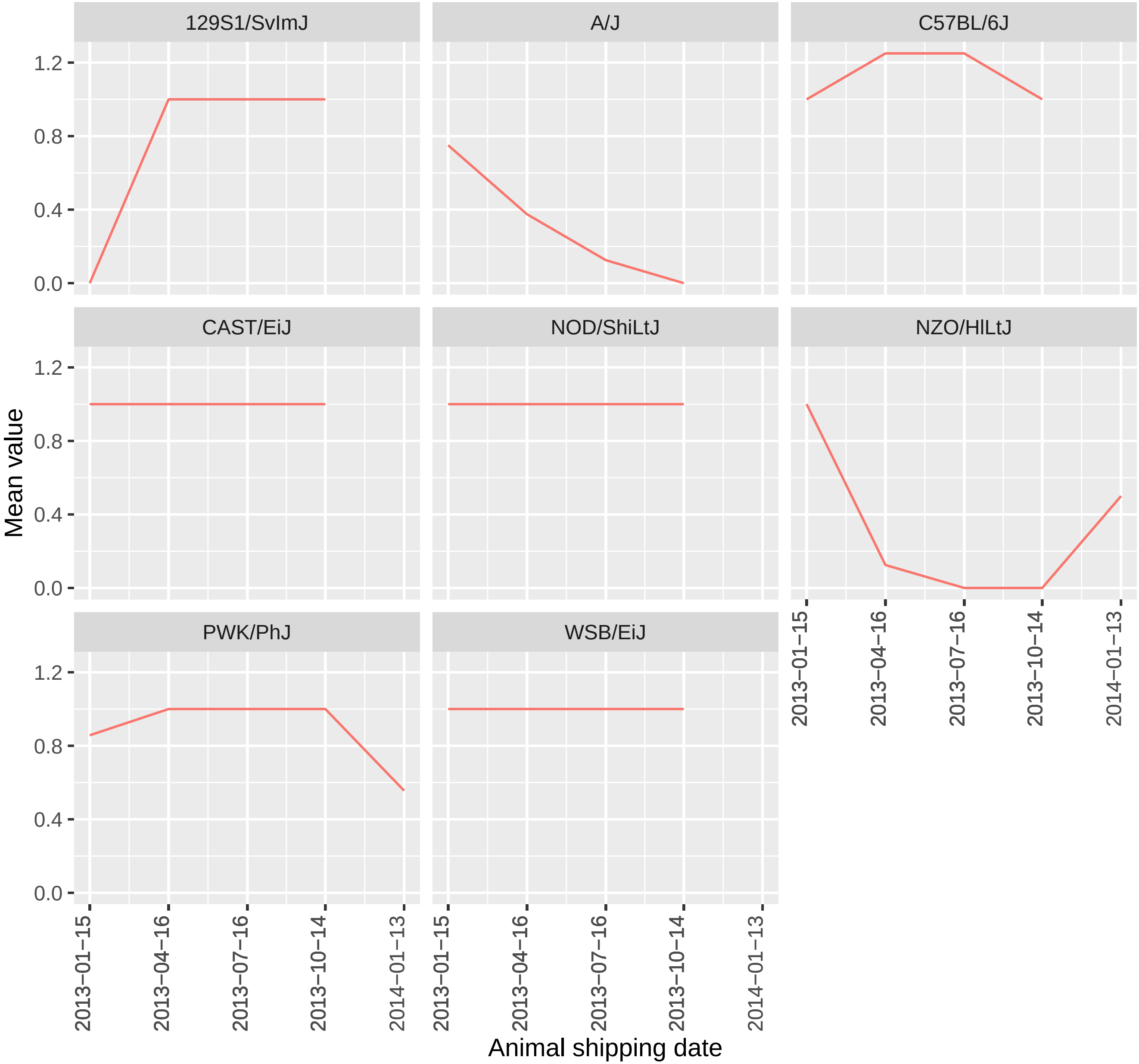

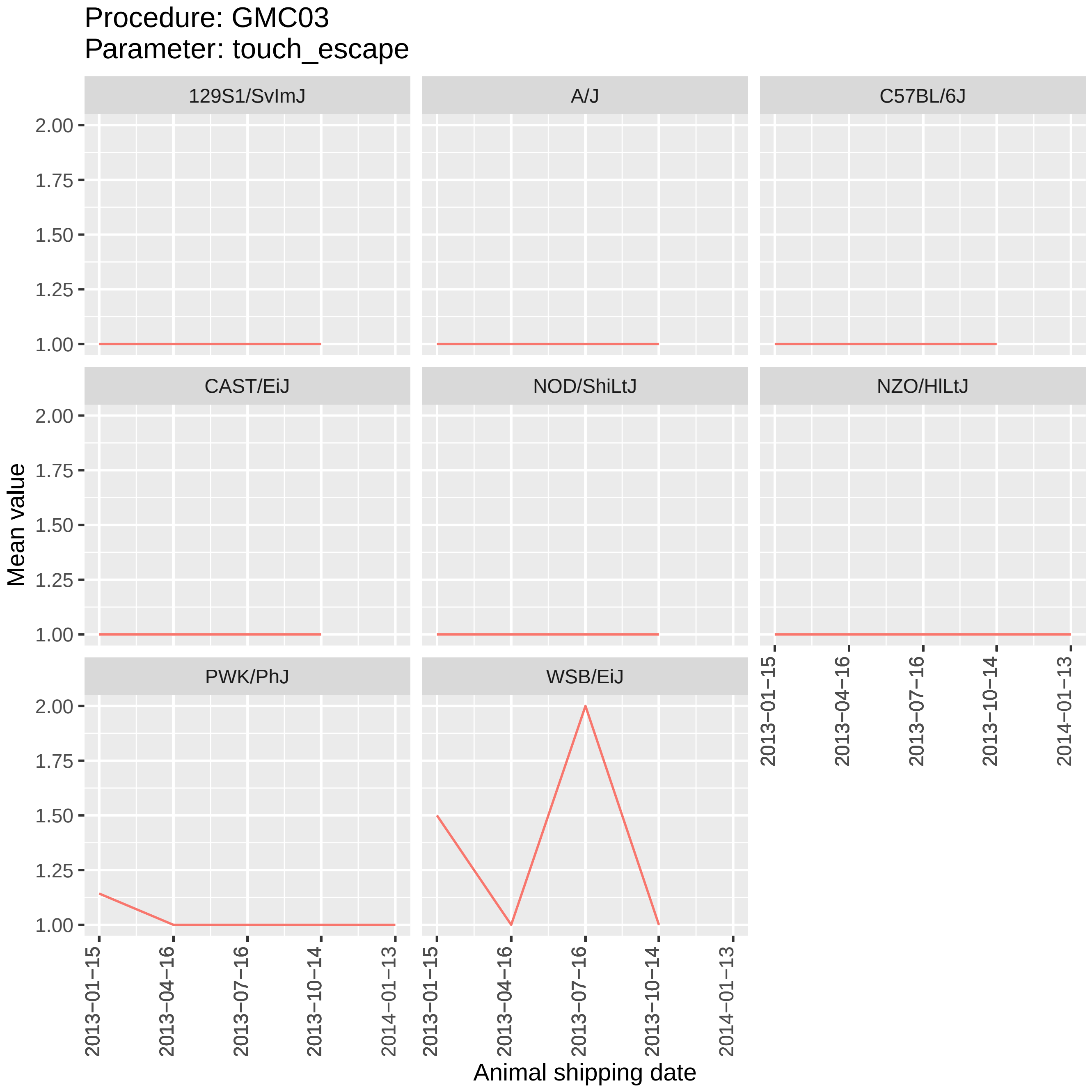

Procedure: GMC03
Parameter: touch_escape
129S1/SvImJ
A/J
C57BL/6J
CAST/EiJ
NOD/ShiLtJ
NZO/HlLtJ
PWK/PhJ
WSB/EiJ
Mean value
2.00
1.75
1.50
1.25
1.00
2013−01−15
2013−04−16
2013−07−16
2013−10−14
2014−01−13
Animal shipping date

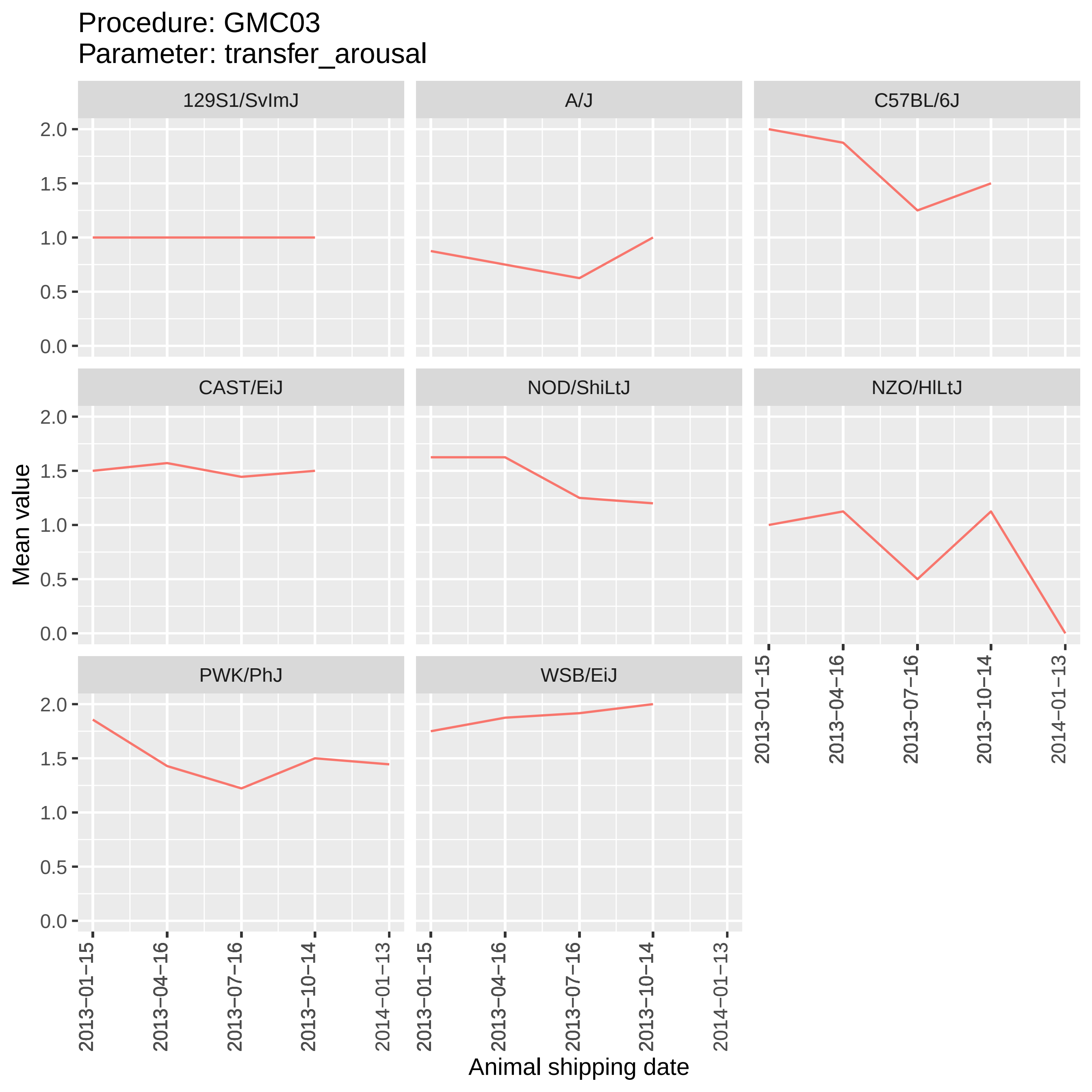

Procedure: GMC03
Parameter: transfer_arousal
129S1/SvImJ
A/J
C57BL/6J
CAST/EiJ
NOD/ShiLtJ
NZO/HlLtJ
PWK/PhJ
WSB/EiJ
Mean value
Animal shipping date
0.0
0.5
1.0
1.5
2.0
2013−01−15
2013−04−16
2013−07−16
2013−10−14
2014−01−13

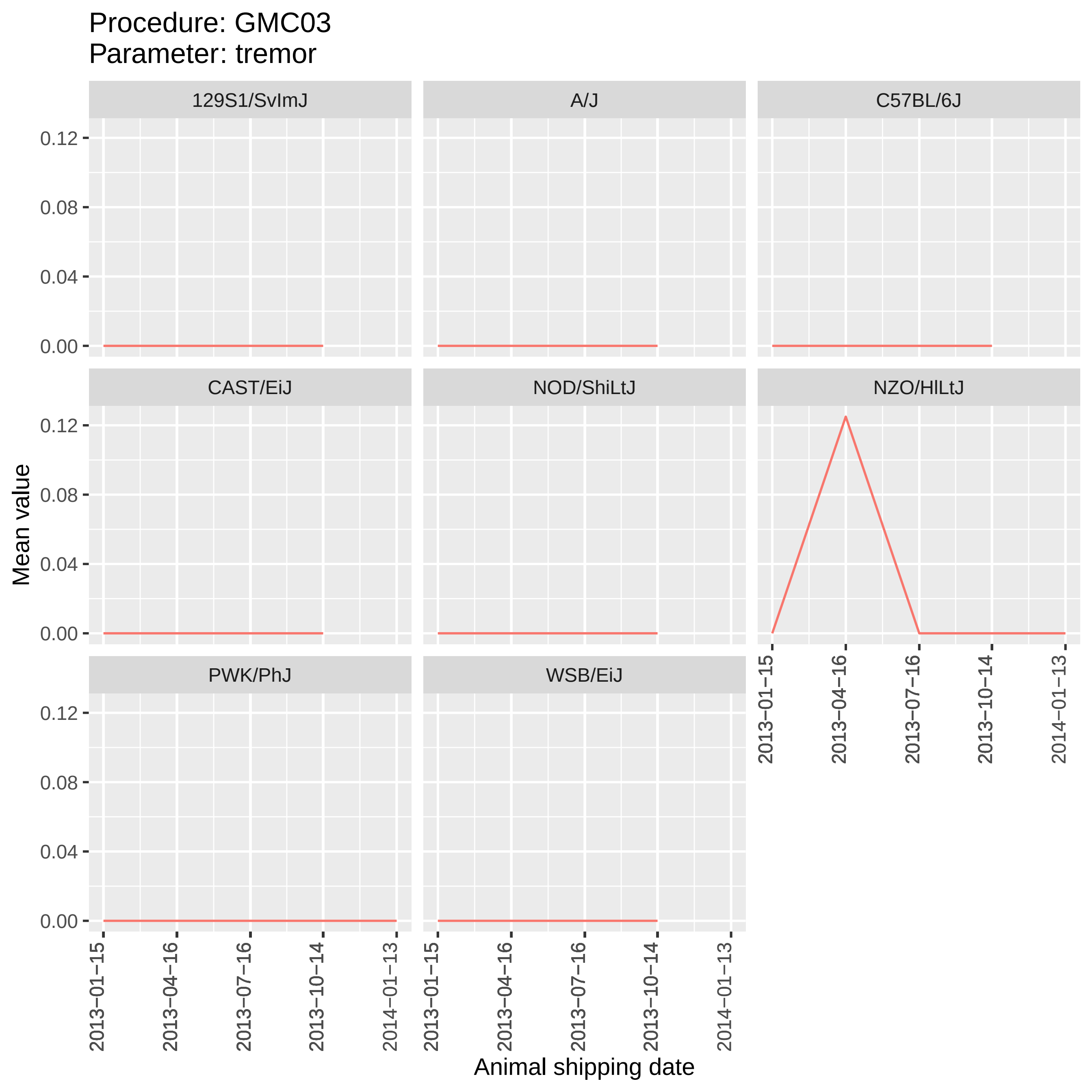

Procedure: GMC03
Parameter: tremor
129S1/SvImJ
A/J
C57BL/6J
CAST/EiJ
NOD/ShiLtJ
NZO/HlLtJ
PWK/PhJ
WSB/EiJ
0.12
0.08
0.04
0.00
Mean value
2013−01−15
2013−04−16
2013−07−16
2013−10−14
2014−01−13
Animal shipping date

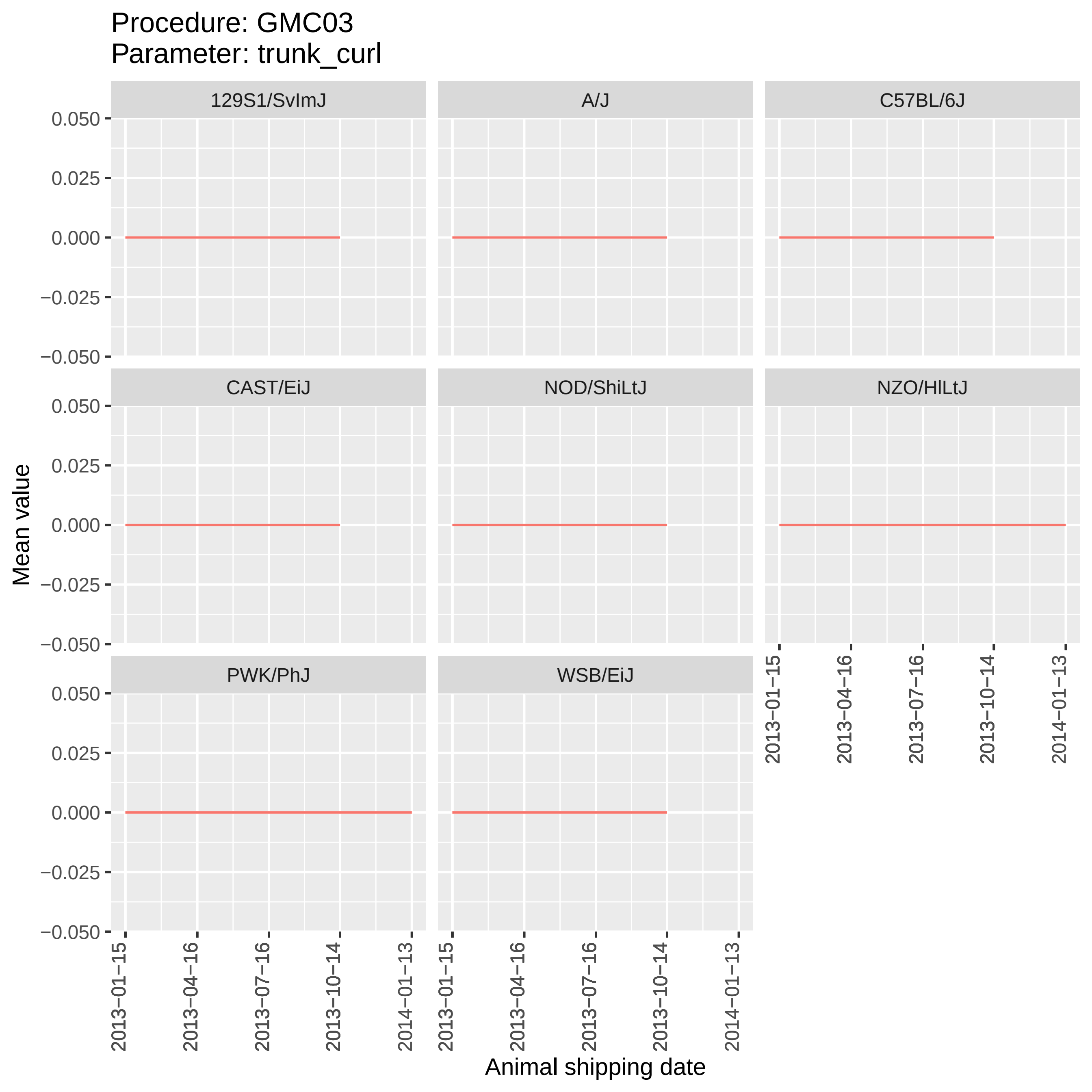

Procedure: GMC03
Parameter: trunk_curl
129S1/SvImJ
A/J
C57BL/6J
CAST/EiJ
NOD/ShiLtJ
NZO/HlLtJ
PWK/PhJ
WSB/EiJ
Mean value
0.050
0.025
0.000
−0.025
−0.050
2013−01−15
2013−04−16
2013−07−16
2013−10−14
2014−01−13
Animal shipping date

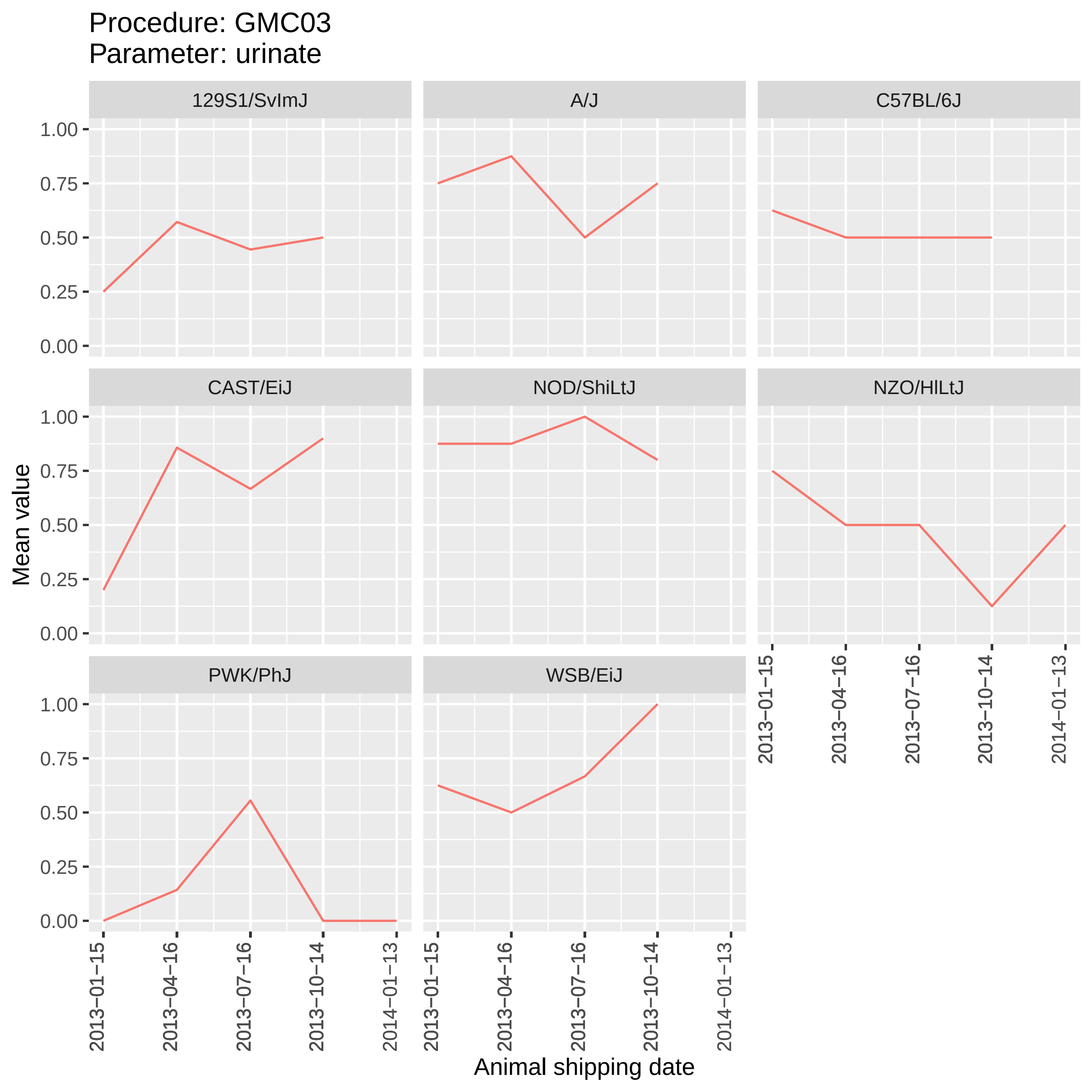

Procedure: GMC03
Parameter: urinate
129S1/SvImJ
A/J
C57BL/6J
CAST/EiJ
NOD/ShiLtJ
NZO/HlLtJ
PWK/PhJ
WSB/EiJ
Mean value
0.00
0.25
0.50
0.75
1.00
Animal shipping date
2013−01−15
2013−04−16
2013−07−16
2013−10−14
2014−01−13

Procedure: GMC03
Parameter: vocalization

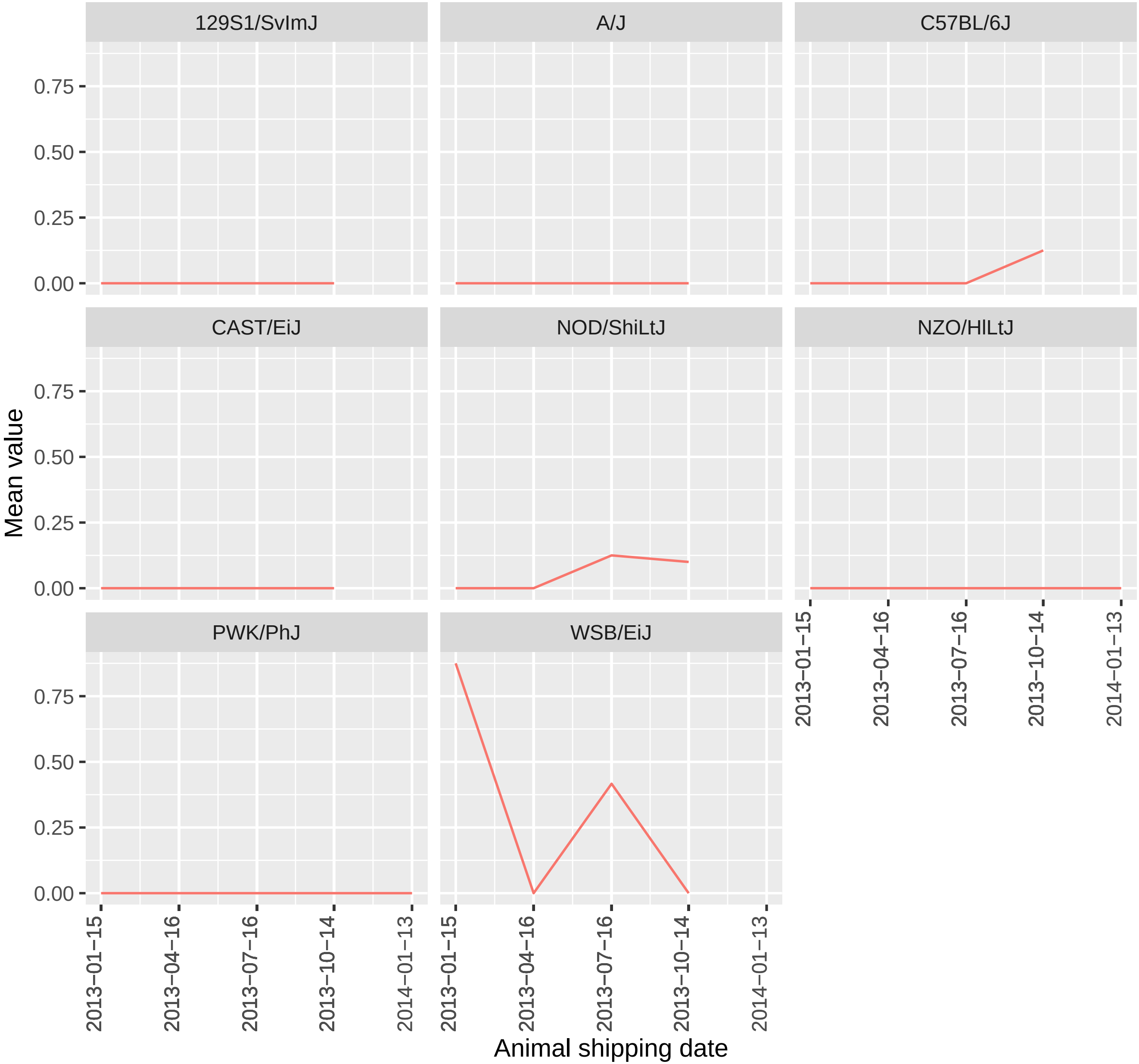

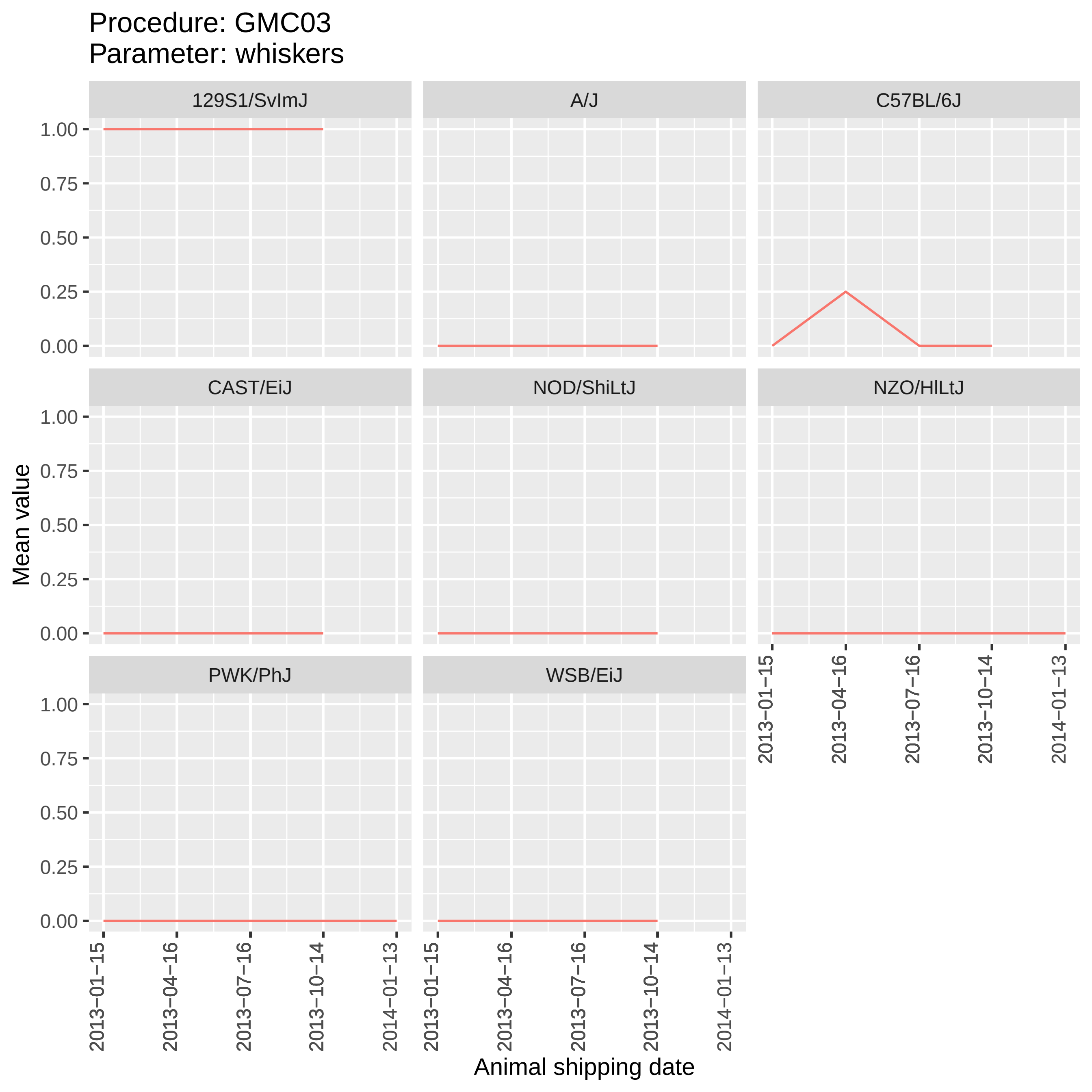
Procedure: GMC03
Parameter: whiskers
129S1/SvImJ
A/J
C57BL/6J
CAST/EiJ
NOD/ShiLtJ
NZO/HlLtJ
PWK/PhJ
WSB/EiJ
1.00
0.75
0.50
0.25
0.00
Mean value
2013−01−15
2013−04−16
2013−07−16
2013−10−14
2014−01−13
Animal shipping date

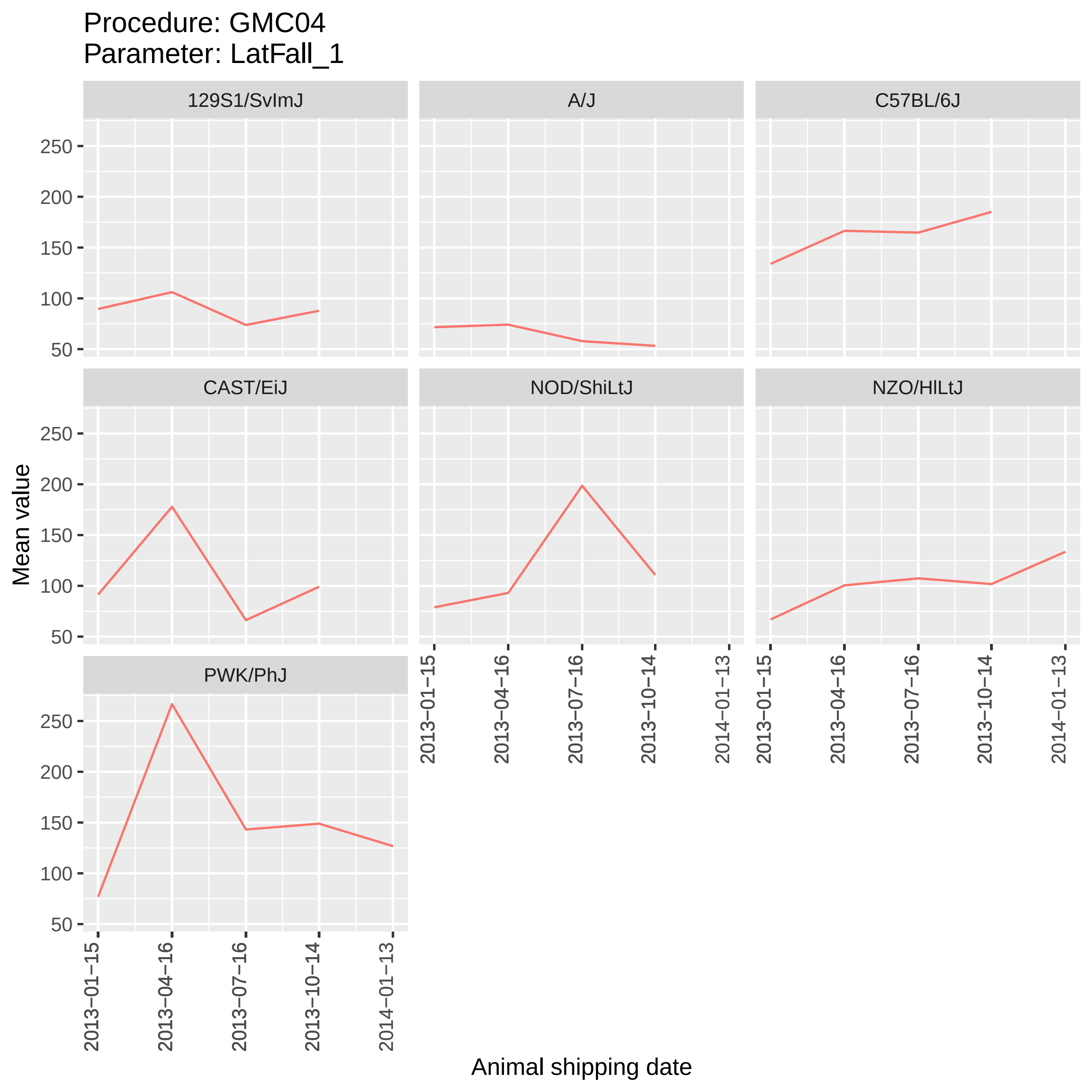

Procedure: GMC04
Parameter: LatFall_1
129S1/SvImJ
A/J
C57BL/6J
CAST/EiJ
NOD/ShiLtJ
NZO/HlLtJ
PWK/PhJ
Mean value
250
200
150
100
50
2013−01−15
2013−04−16
2013−07−16
2013−10−14
2014−01−13
Animal shipping date

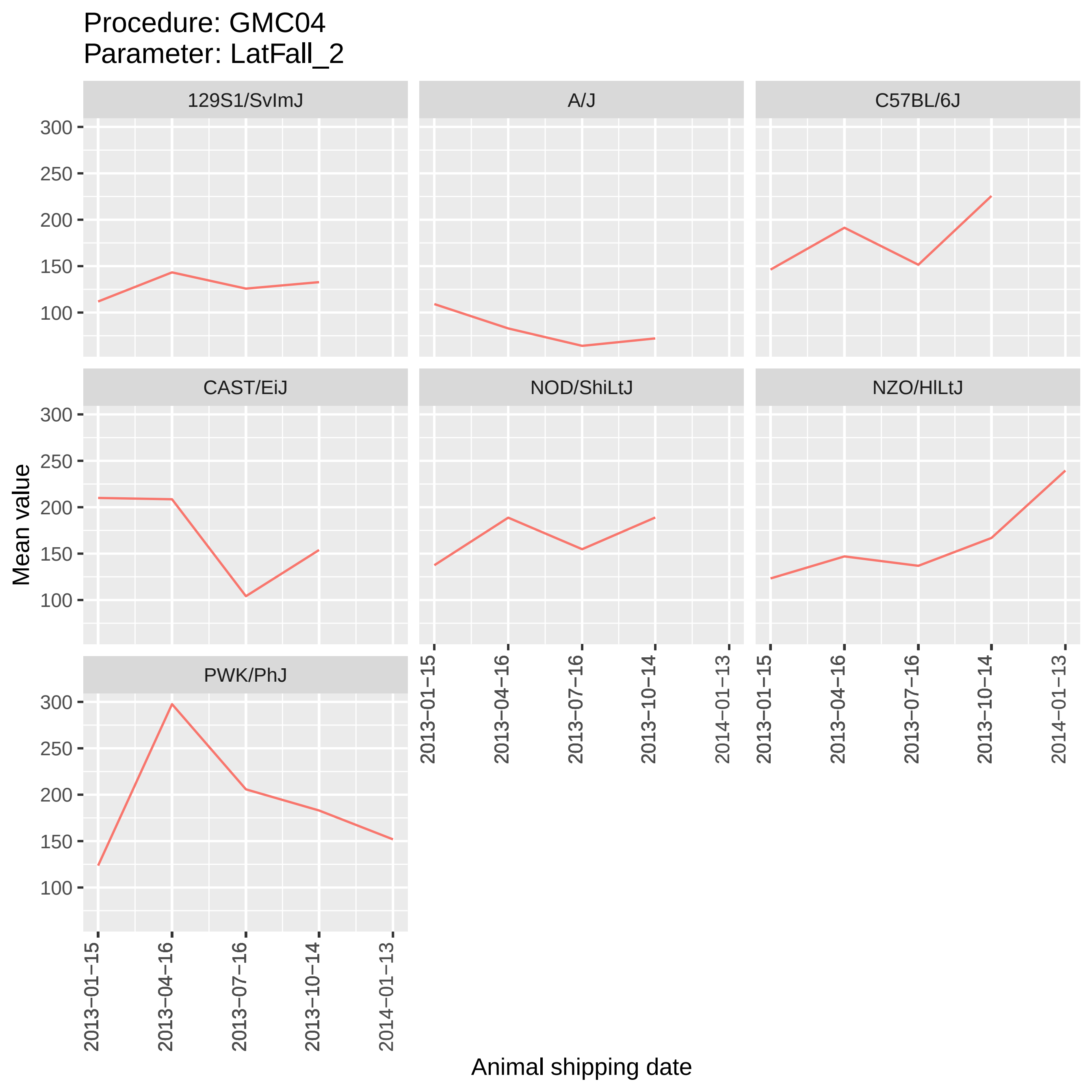
Procedure: GMC04
Parameter: LatFall_2
129S1/SvImJ
A/J
C57BL/6J
CAST/EiJ
NOD/ShiLtJ
NZO/HlLtJ
PWK/PhJ
Mean value
100
150
200
250
300
2013−01−15
2013−04−16
2013−07−16
2013−10−14
2014−01−13
Animal shipping date

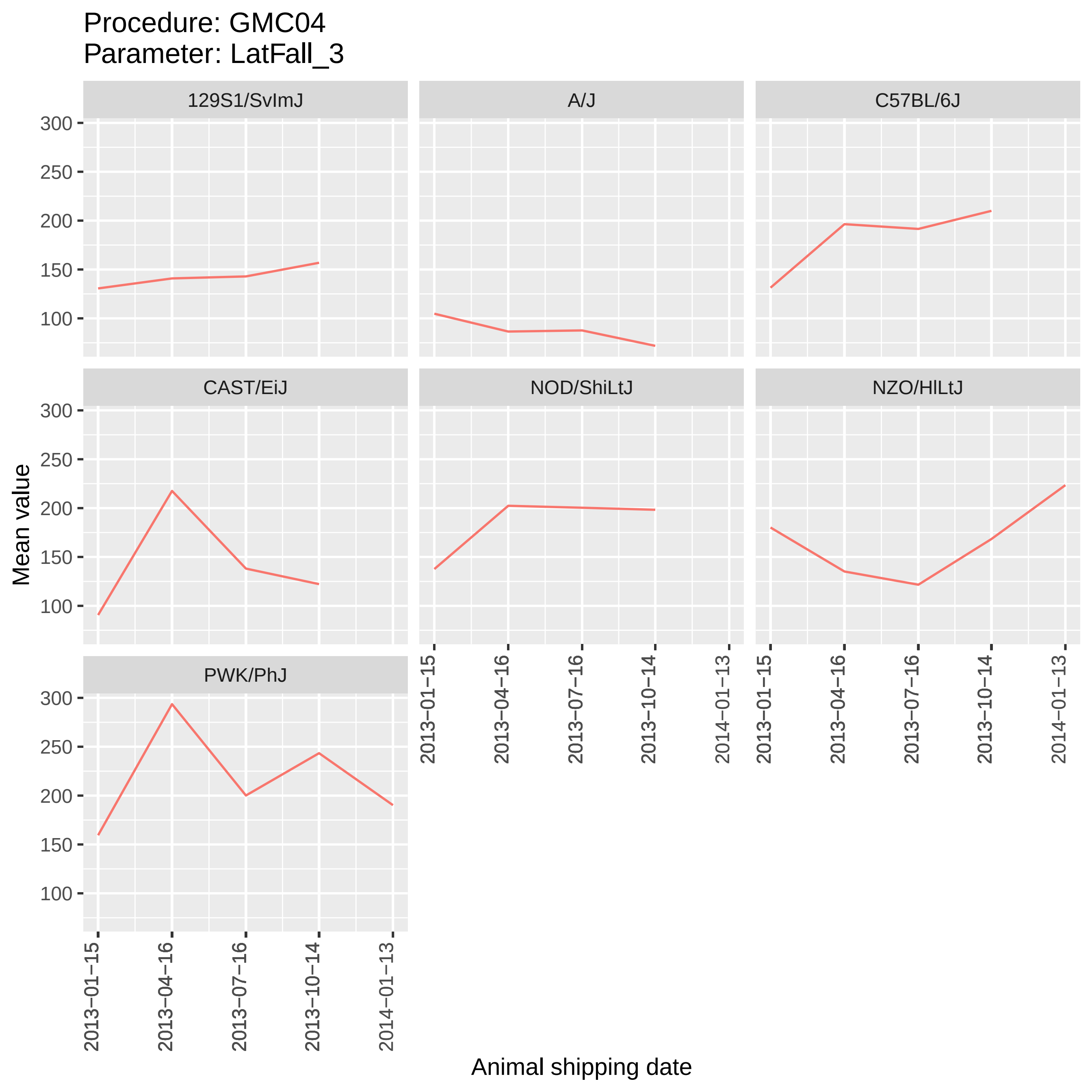

Procedure: GMC04
Parameter: LatFall_3
129S1/SvImJ
A/J
C57BL/6J
CAST/EiJ
NOD/ShiLtJ
NZO/HlLtJ
PWK/PhJ
Mean value
Animal shipping date
100
150
200
250
300
2013−01−15
2013−04−16
2013−07−16
2013−10−14
2014−01−13

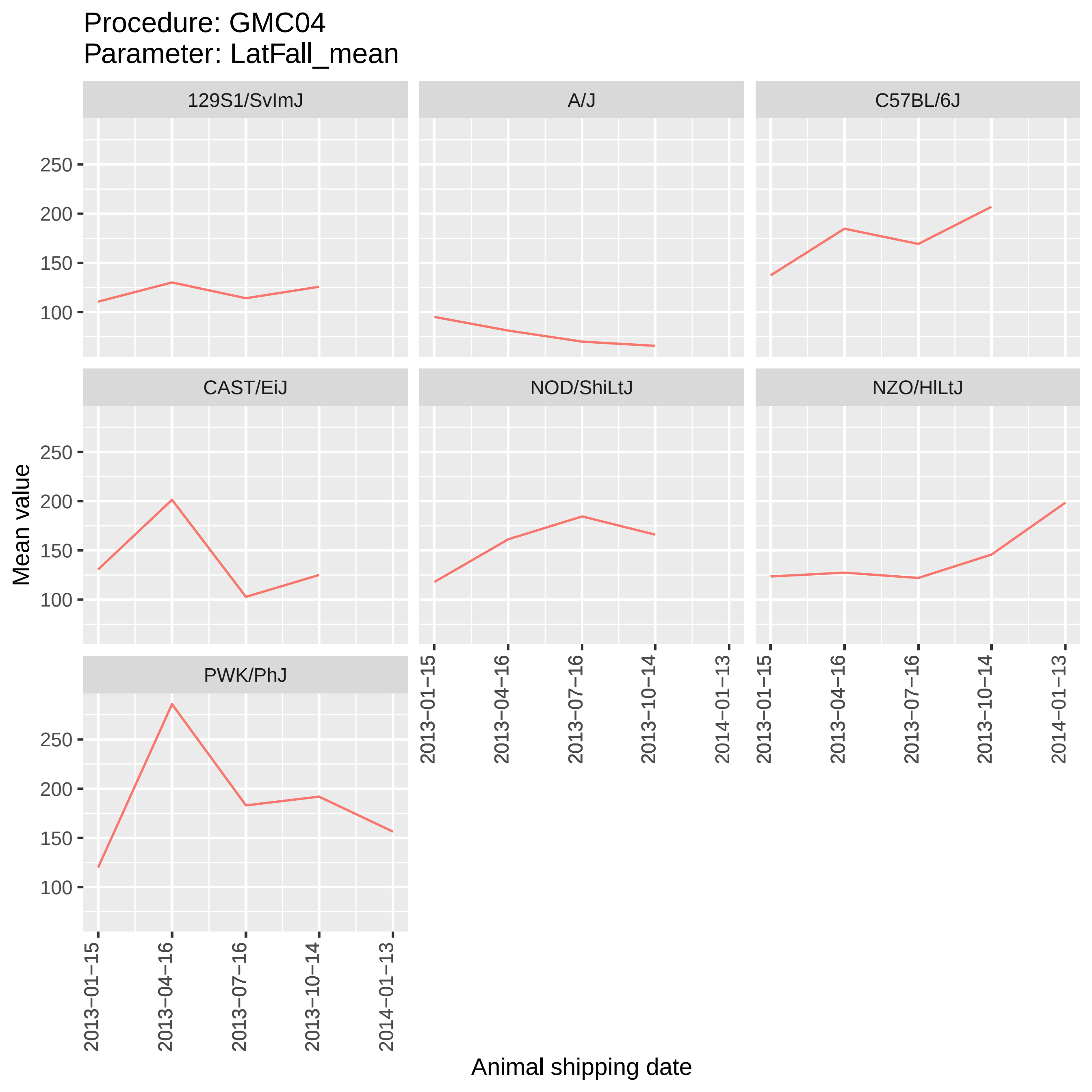

Procedure: GMC04
Parameter: LatFall_mean
129S1/SvImJ
A/J
C57BL/6J
CAST/EiJ
NOD/ShiLtJ
NZO/HlLtJ
PWK/PhJ
Mean value
100
150
200
250
2013−01−15
2013−04−16
2013−07−16
2013−10−14
2014−01−13
Animal shipping date

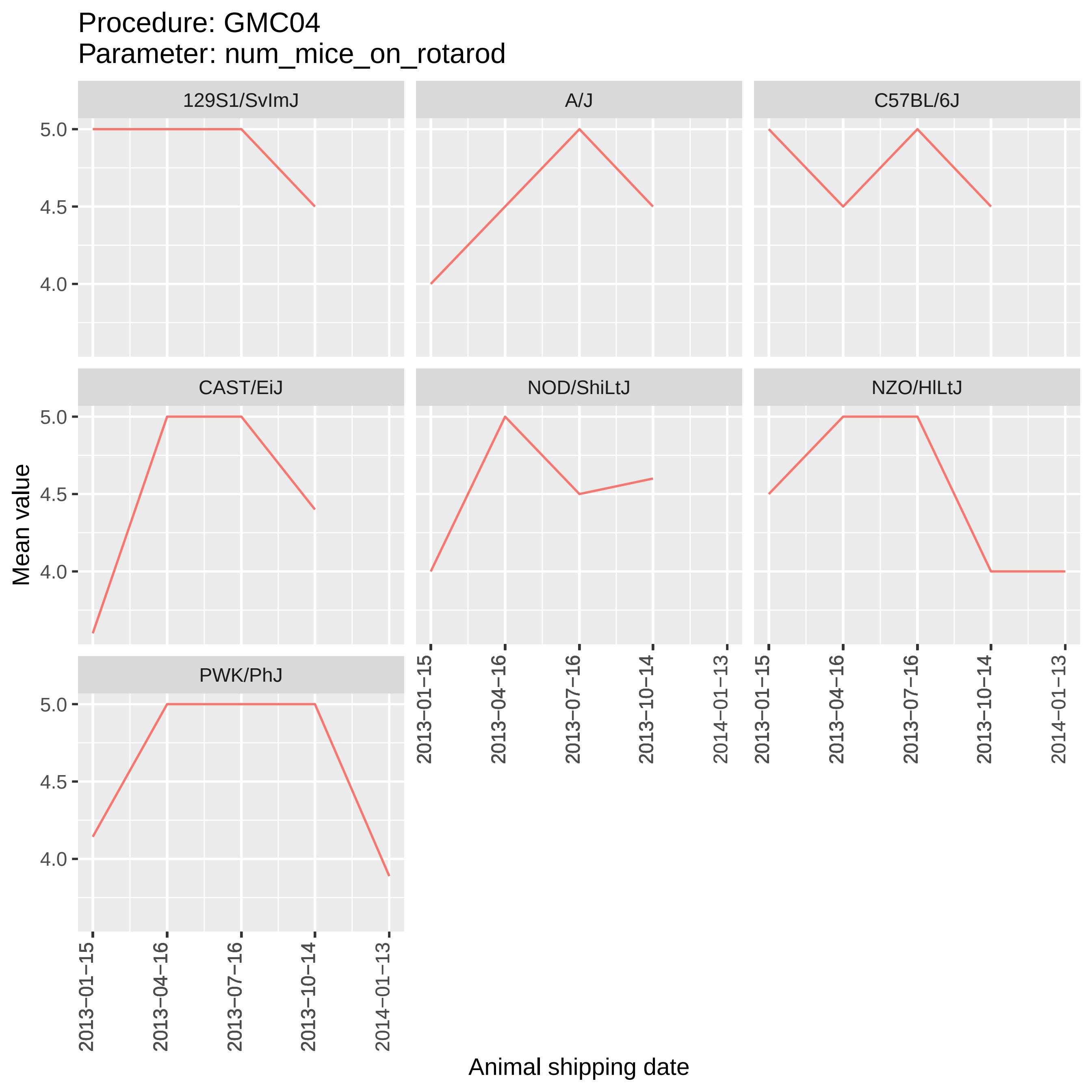
Procedure: GMC04
Parameter: num_mice_on_rotarod
129S1/SvImJ
A/J
C57BL/6J
CAST/EiJ
NOD/ShiLtJ
NZO/HlLtJ
PWK/PhJ
Mean value
5.0
4.5
4.0
2013−01−15
2013−04−16
2013−07−16
2013−10−14
2014−01−13
Animal shipping date

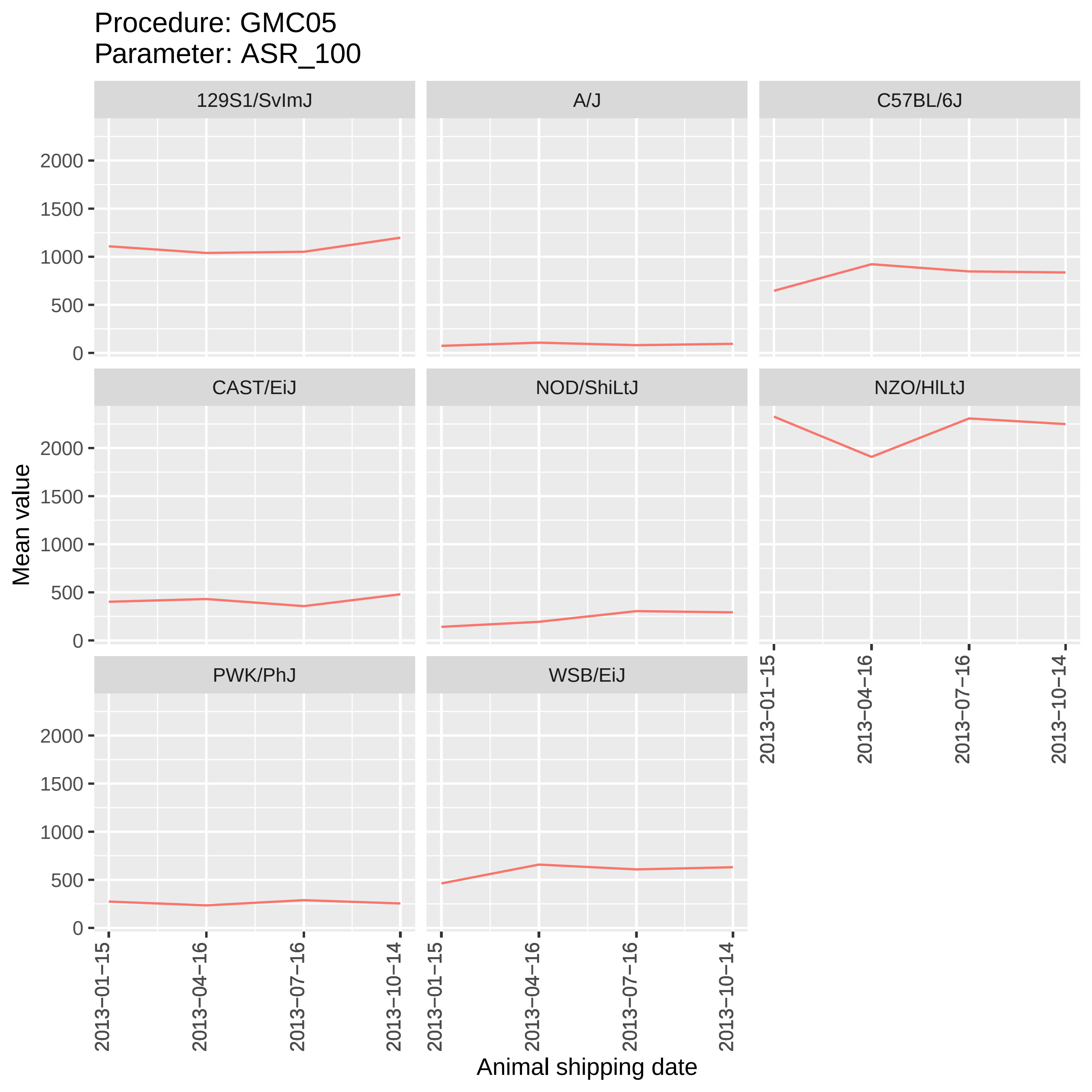
Procedure: GMC05
Parameter: ASR_100
129S1/SvImJ
A/J
C57BL/6J
CAST/EiJ
NOD/ShiLtJ
NZO/HlLtJ
PWK/PhJ
WSB/EiJ
Mean value
0
500
1000
1500
2000
2013−01−15
2013−04−16
2013−07−16
2013−10−14
Animal shipping date

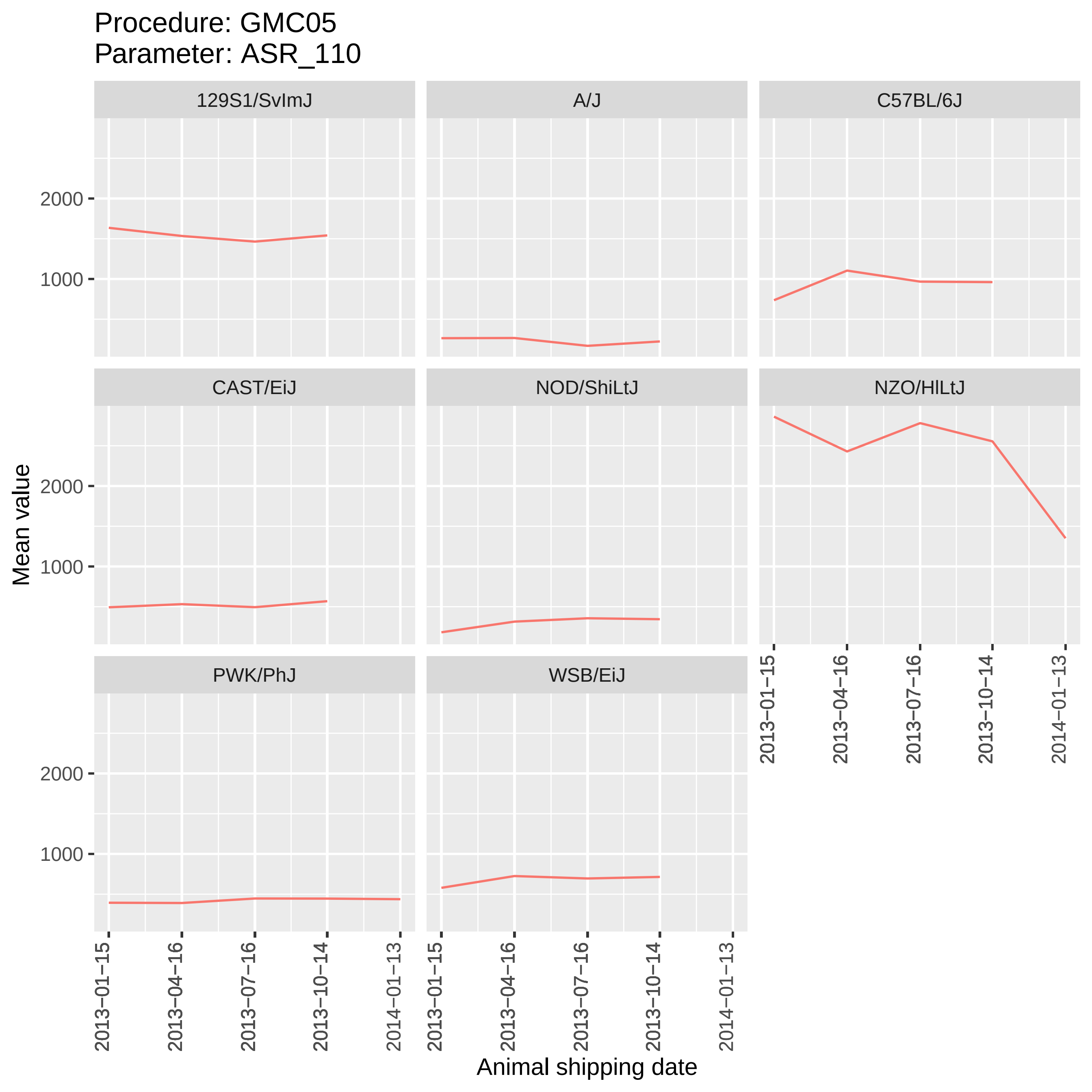

Procedure: GMC05
Parameter: ASR_110
129S1/SvImJ
A/J
C57BL/6J
CAST/EiJ
NOD/ShiLtJ
NZO/HlLtJ
PWK/PhJ
WSB/EiJ
Mean value
2000
1000
2013−01−15
2013−04−16
2013−07−16
2013−10−14
2014−01−13
Animal shipping date

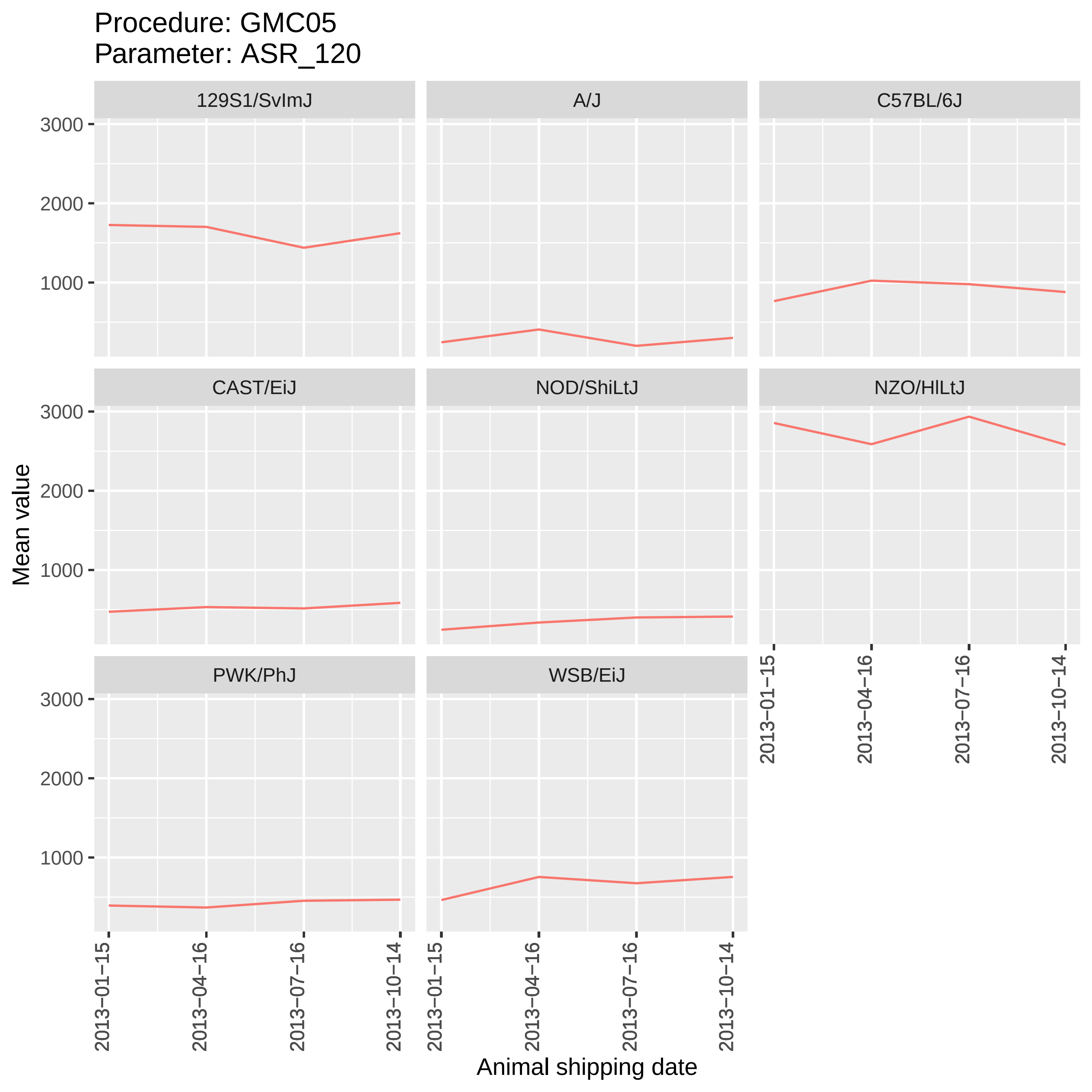

Procedure: GMC05
Parameter: ASR_120
129S1/SvImJ
A/J
C57BL/6J
CAST/EiJ
NOD/ShiLtJ
NZO/HlLtJ
PWK/PhJ
WSB/EiJ
3000
2000
1000
Mean value
2013−01−15
2013−04−16
2013−07−16
2013−10−14
Animal shipping date

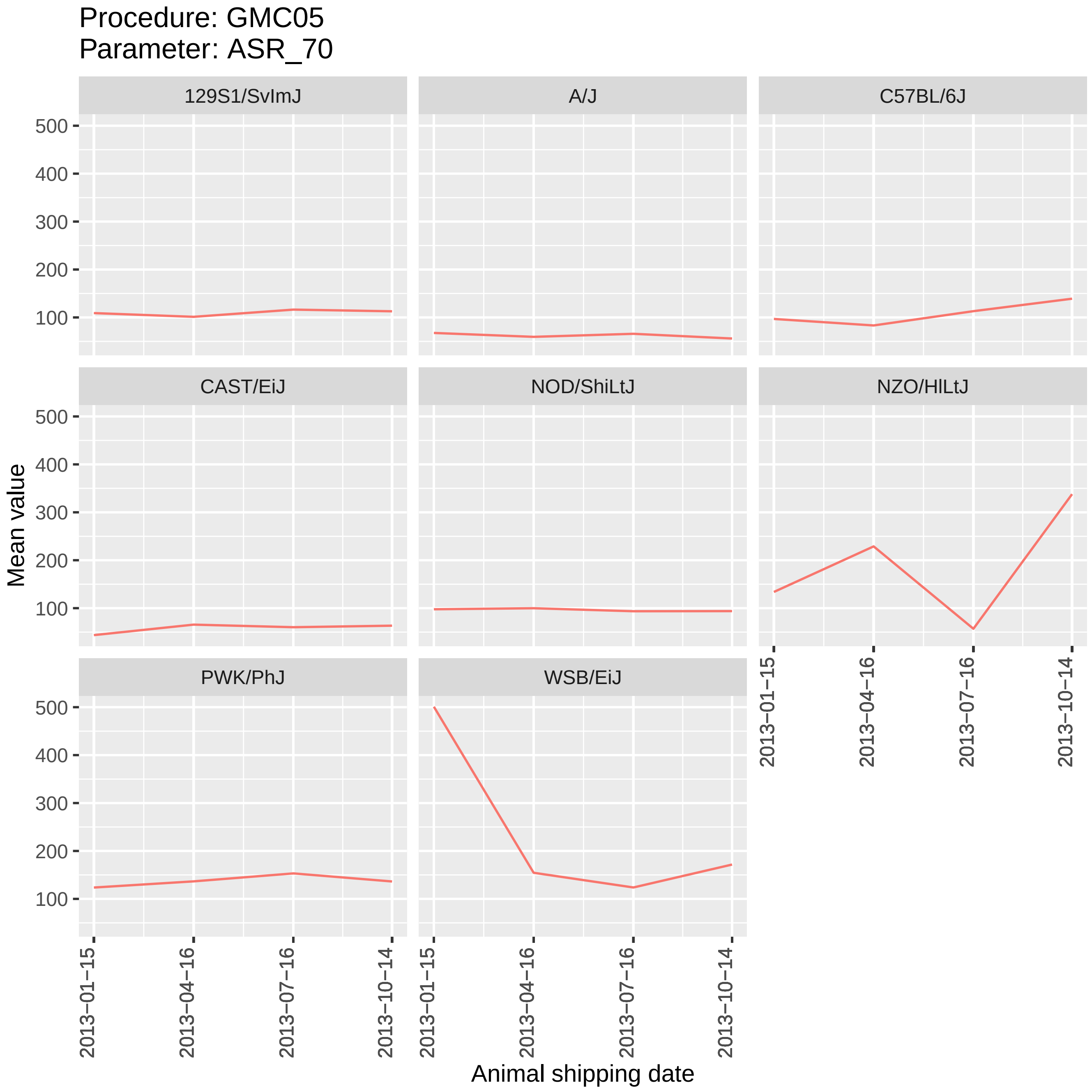

Procedure: GMC05
Parameter: ASR_70
129S1/SvImJ
A/J
C57BL/6J
CAST/EiJ
NOD/ShiLtJ
NZO/HlLtJ
PWK/PhJ
WSB/EiJ
Mean value
100
200
300
400
500
2013−01−15
2013−04−16
2013−07−16
2013−10−14
Animal shipping date

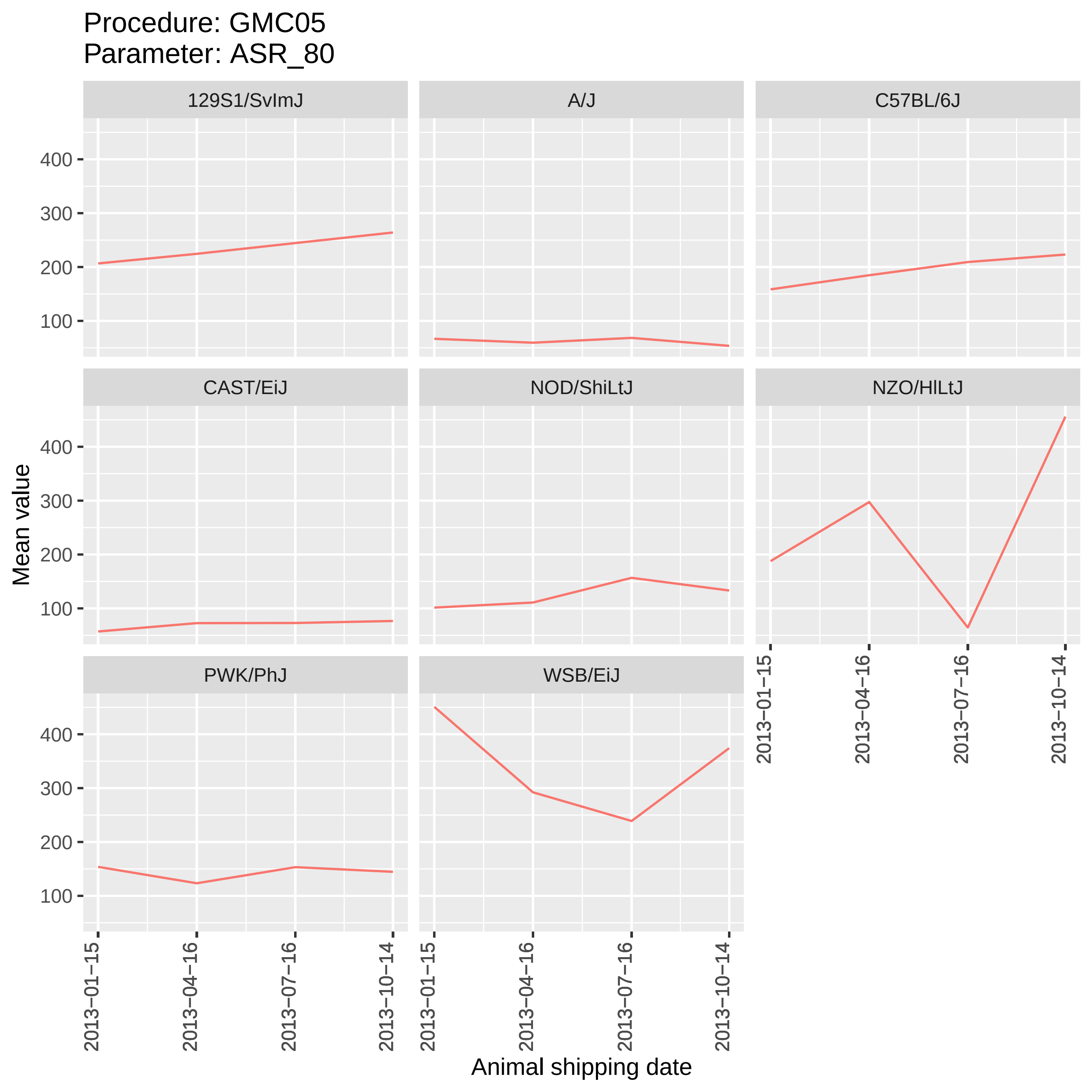

Procedure: GMC05
Parameter: ASR_80
129S1/SvImJ
A/J
C57BL/6J
CAST/EiJ
NOD/ShiLtJ
NZO/HlLtJ
PWK/PhJ
WSB/EiJ
Mean value
100
200
300
400
2013−01−15
2013−04−16
2013−07−16
2013−10−14
Animal shipping date

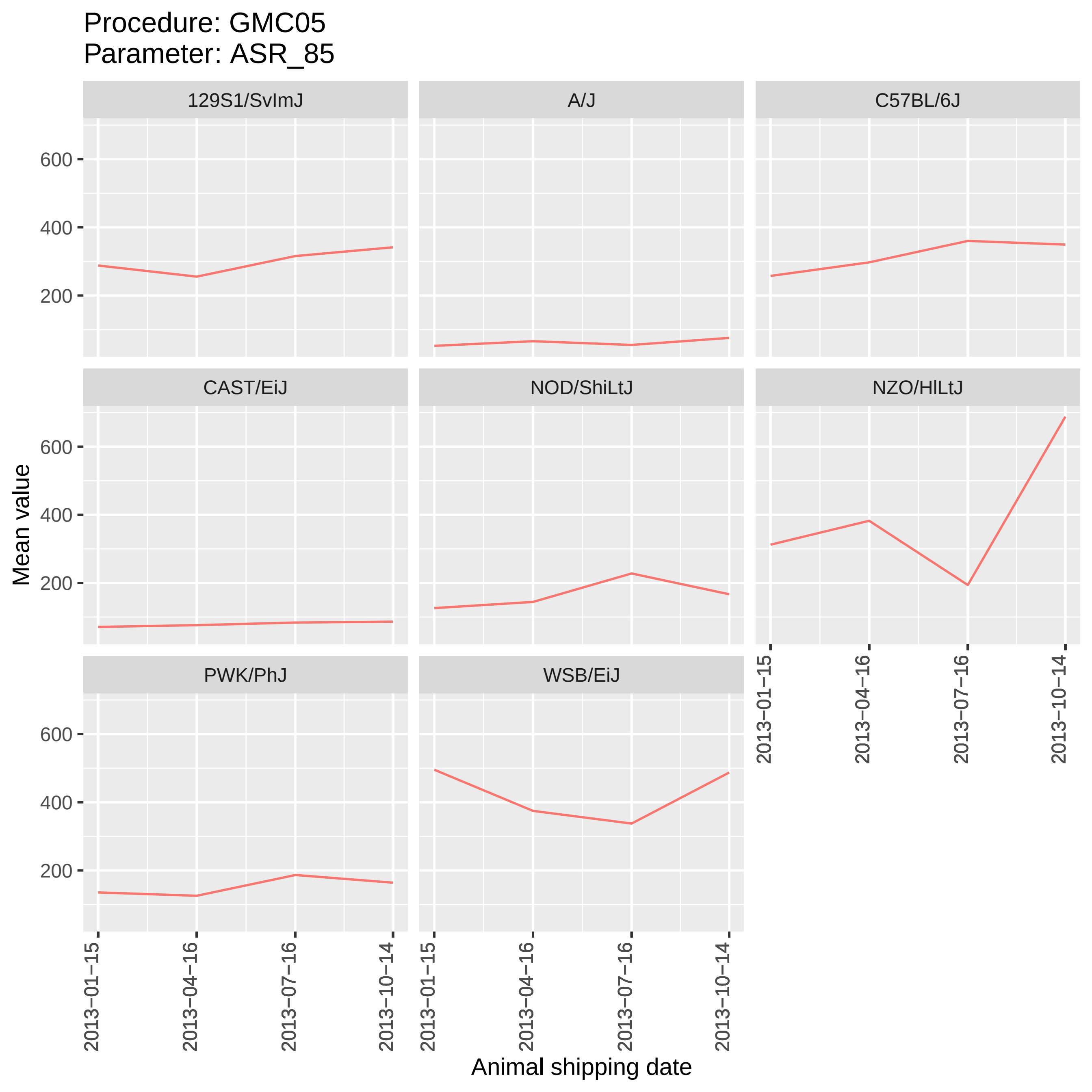
Procedure: GMC05
Parameter: ASR_85
129S1/SvImJ
A/J
C57BL/6J
CAST/EiJ
NOD/ShiLtJ
NZO/HlLtJ
PWK/PhJ
WSB/EiJ
600
400
200
Mean value
2013−01−15
2013−04−16
2013−07−16
2013−10−14
Animal shipping date

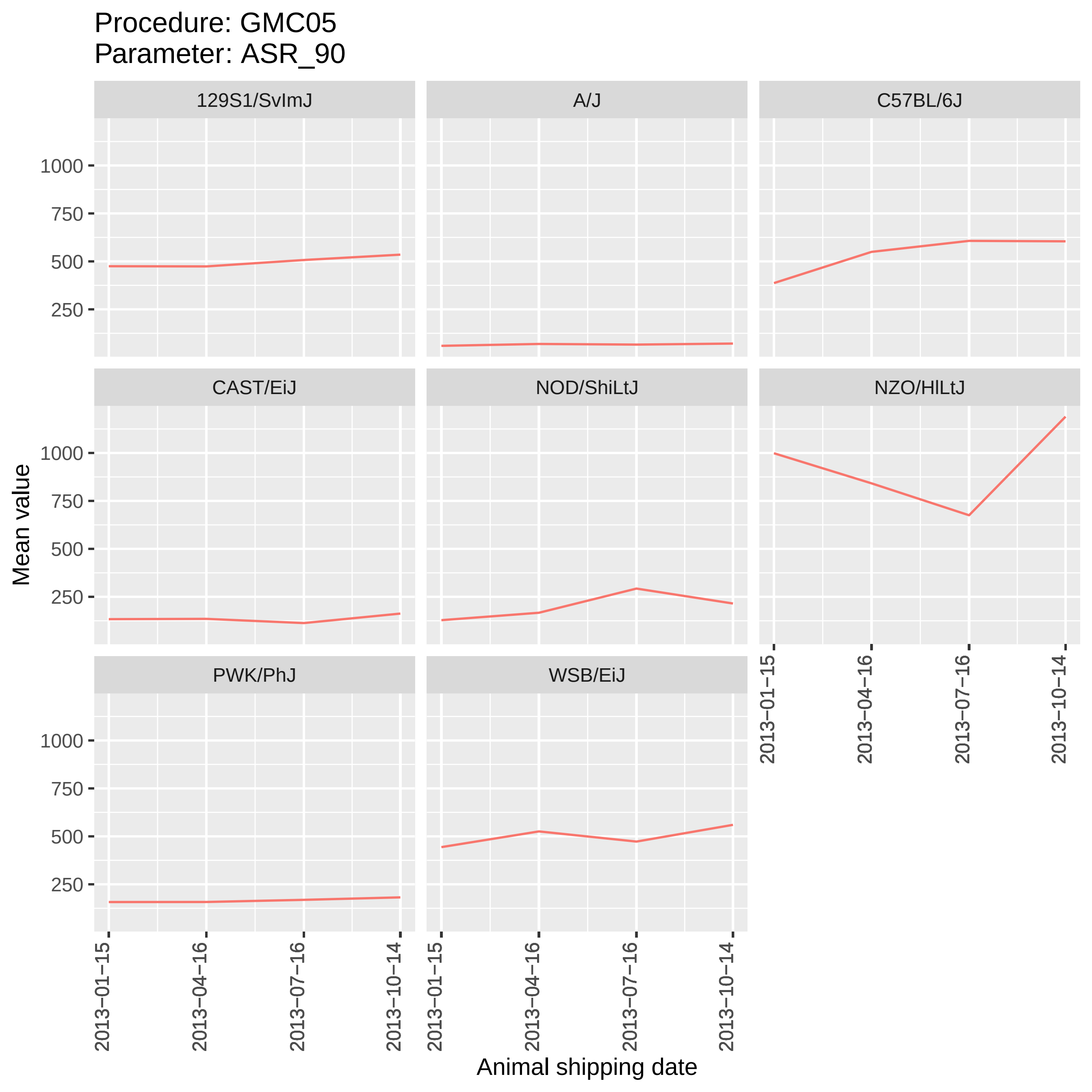

Procedure: GMC05
Parameter: ASR_90
129S1/SvImJ
A/J
C57BL/6J
CAST/EiJ
NOD/ShiLtJ
NZO/HlLtJ
PWK/PhJ
WSB/EiJ
1000
750
500
250
Mean value
2013−01−15
2013−04−16
2013−07−16
2013−10−14
Animal shipping date

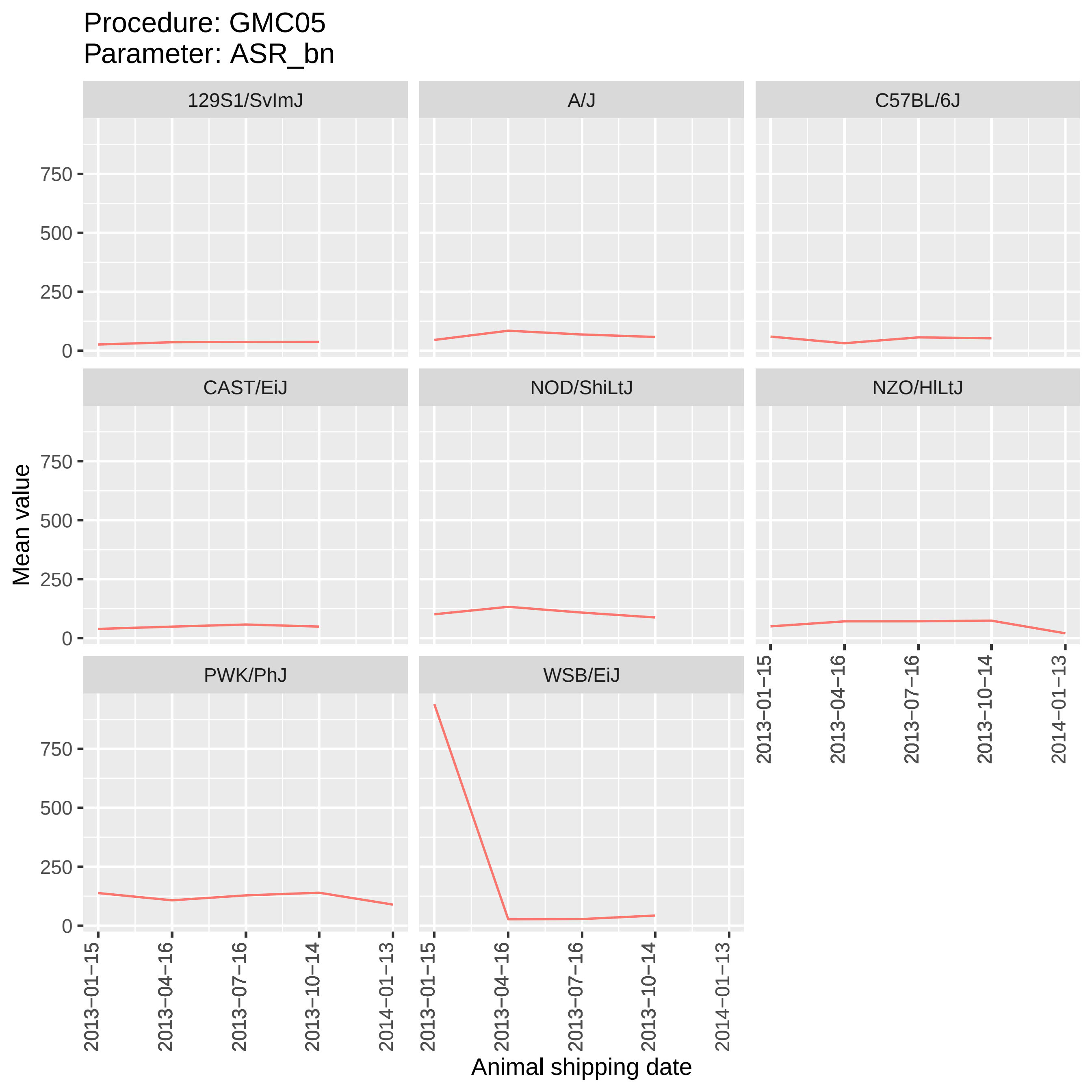

Procedure: GMC05
Parameter: ASR_bn
129S1/SvImJ
A/J
C57BL/6J
CAST/EiJ
NOD/ShiLtJ
NZO/HlLtJ
PWK/PhJ
WSB/EiJ
Mean value
0
250
500
750
2013−01−15
2013−04−16
2013−07−16
2013−10−14
2014−01−13
Animal shipping date

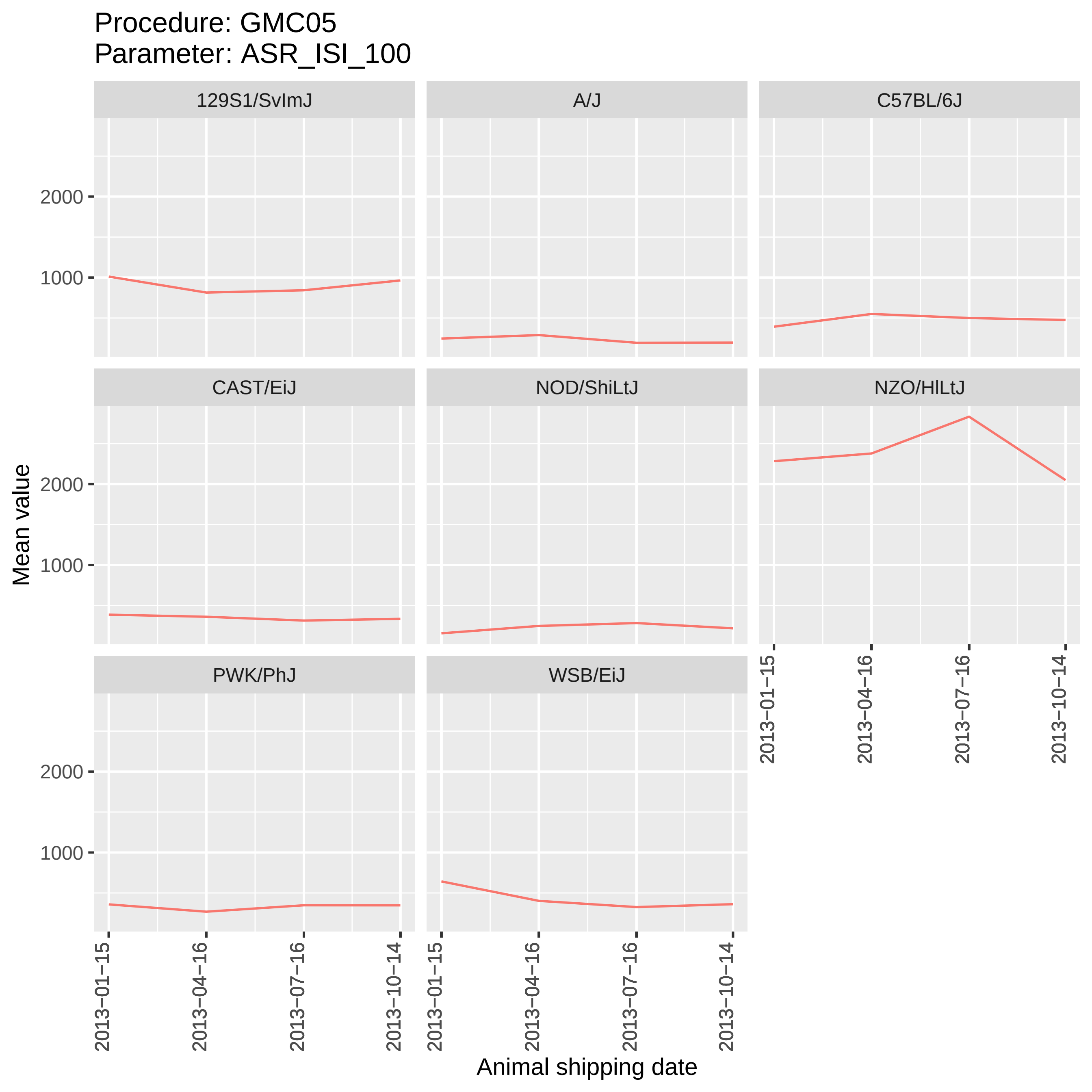

Procedure: GMC05
Parameter: ASR_ISI_100
129S1/SvImJ
A/J
C57BL/6J
CAST/EiJ
NOD/ShiLtJ
NZO/HlLtJ
PWK/PhJ
WSB/EiJ
Mean value
2000
1000
2013−01−15
2013−04−16
2013−07−16
2013−10−14
Animal shipping date

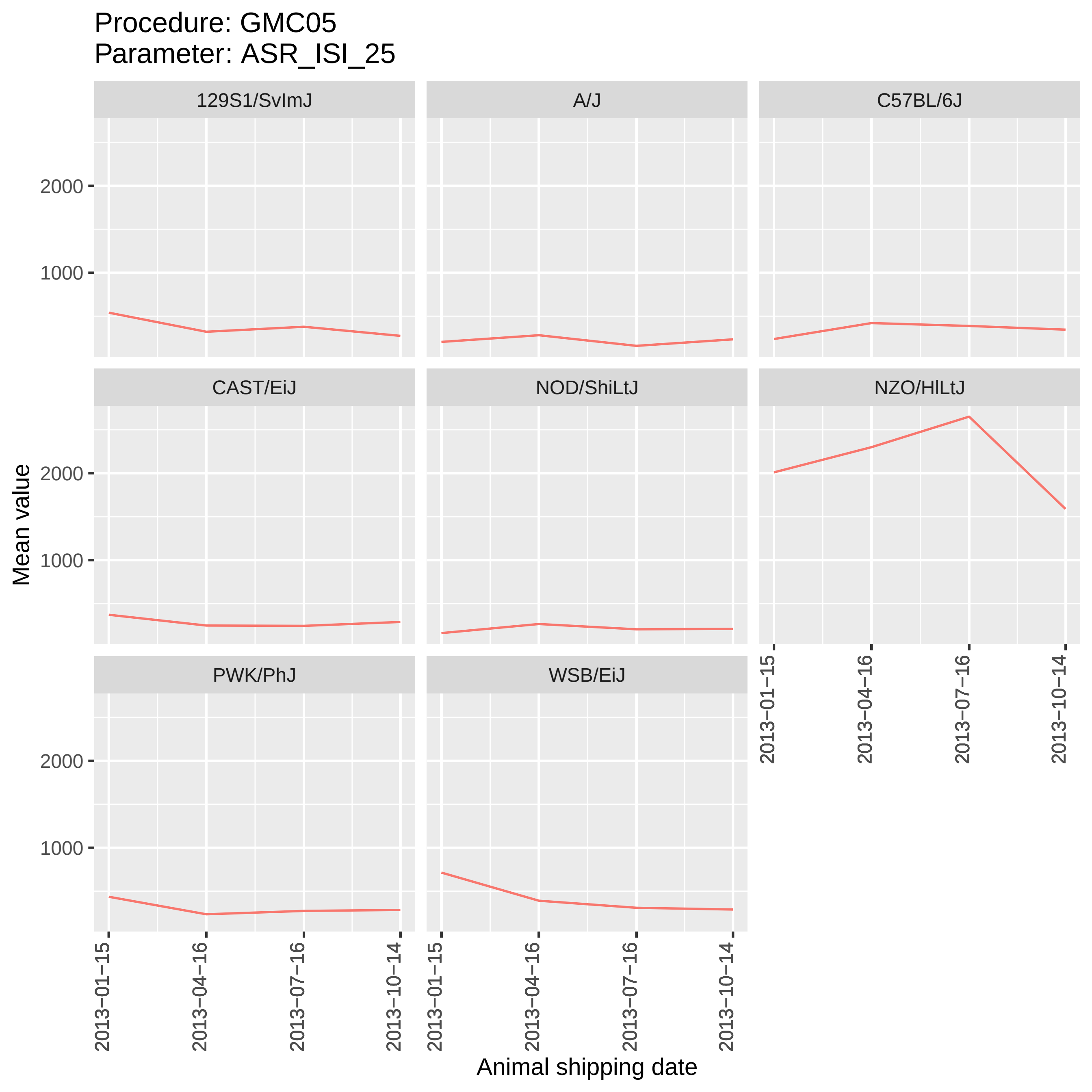

Procedure: GMC05
Parameter: ASR_ISI_25
129S1/SvImJ
A/J
C57BL/6J
CAST/EiJ
NOD/ShiLtJ
NZO/HlLtJ
PWK/PhJ
WSB/EiJ
Mean value
2000
1000
2013−01−15
2013−04−16
2013−07−16
2013−10−14
Animal shipping date

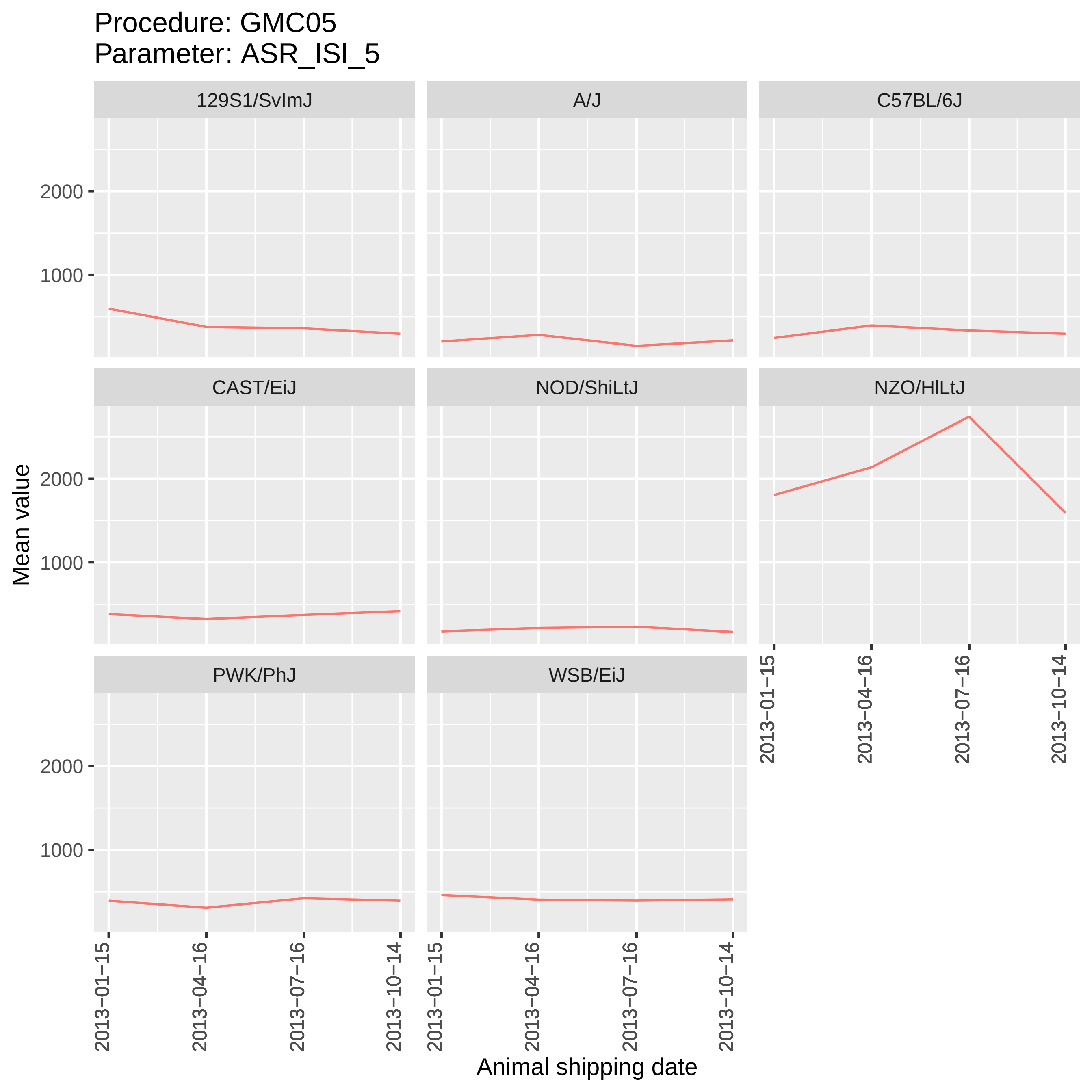

Procedure: GMC05
Parameter: ASR_ISI_5
129S1/SvImJ
A/J
C57BL/6J
CAST/EiJ
NOD/ShiLtJ
NZO/HlLtJ
PWK/PhJ
WSB/EiJ
Mean value
2000
1000
2013−01−15
2013−04−16
2013−07−16
2013−10−14
Animal shipping date

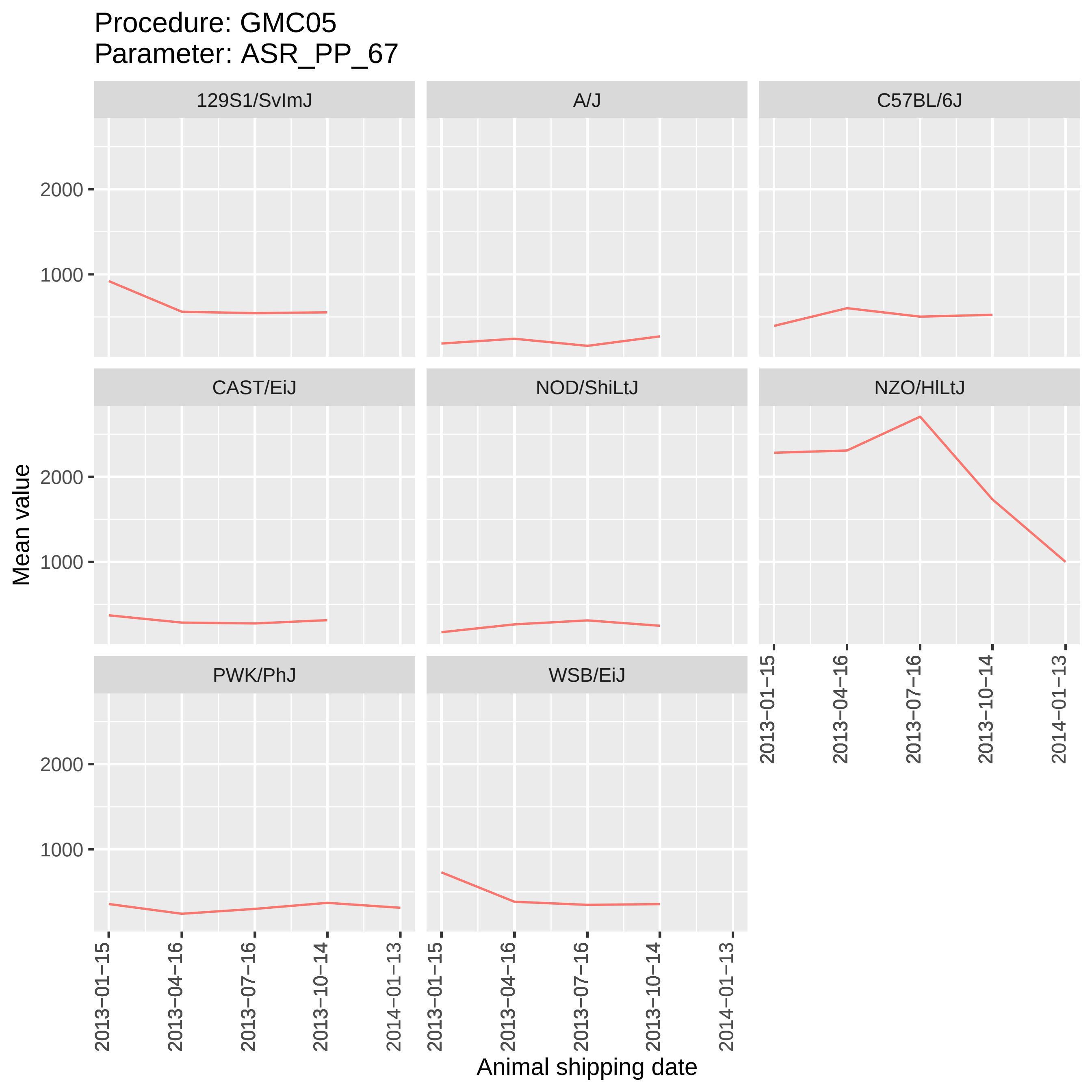

Procedure: GMC05
Parameter: ASR_PP_67
129S1/SvImJ
A/J
C57BL/6J
CAST/EiJ
NOD/ShiLtJ
NZO/HlLtJ
PWK/PhJ
WSB/EiJ
Mean value
2000
1000
2013−01−15
2013−04−16
2013−07−16
2013−10−14
2014−01−13
Animal shipping date

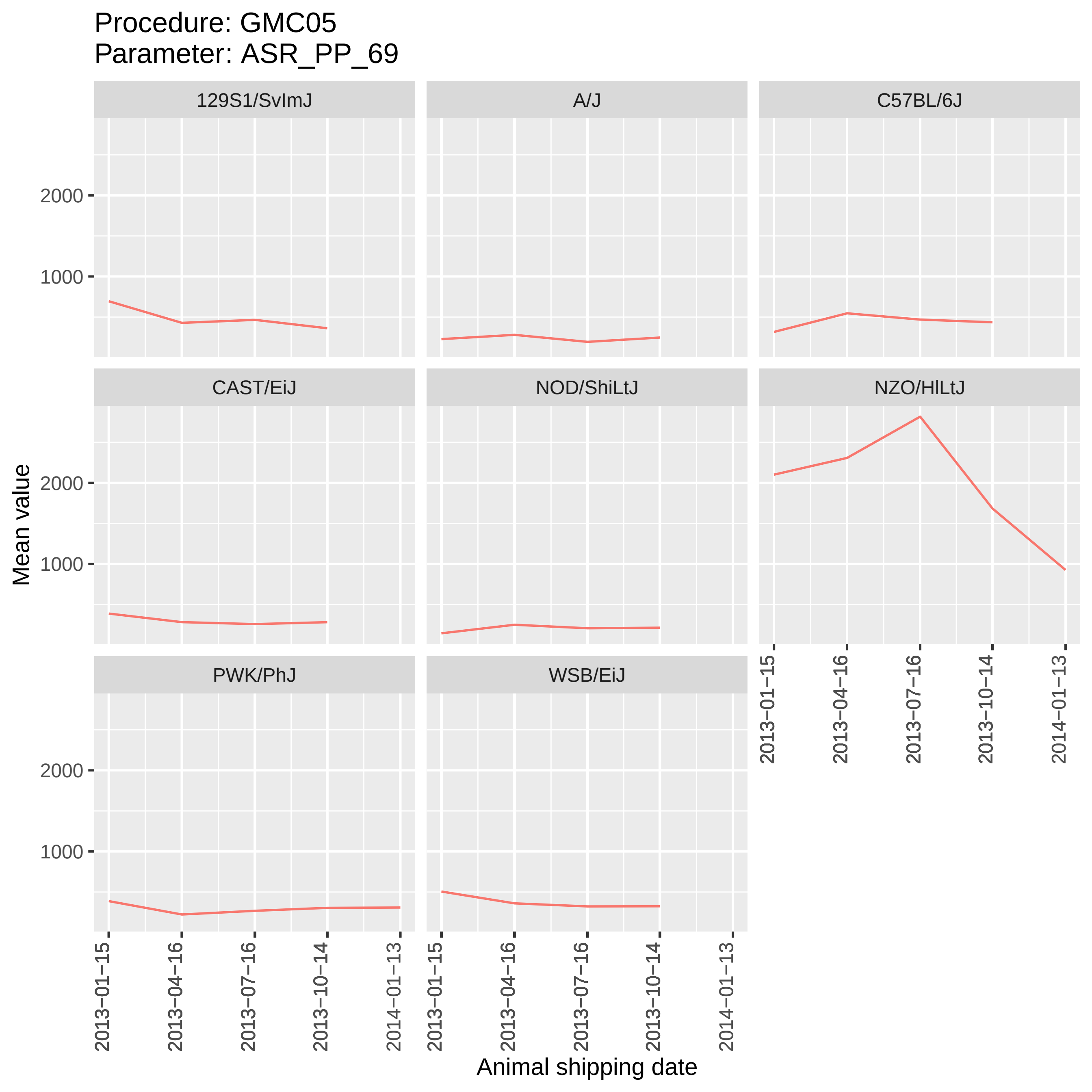

Procedure: GMC05
Parameter: ASR_PP_69
129S1/SvImJ
A/J
C57BL/6J
CAST/EiJ
NOD/ShiLtJ
NZO/HlLtJ
PWK/PhJ
WSB/EiJ
Mean value
1000
2000
2013−01−15
2013−04−16
2013−07−16
2013−10−14
2014−01−13
Animal shipping date

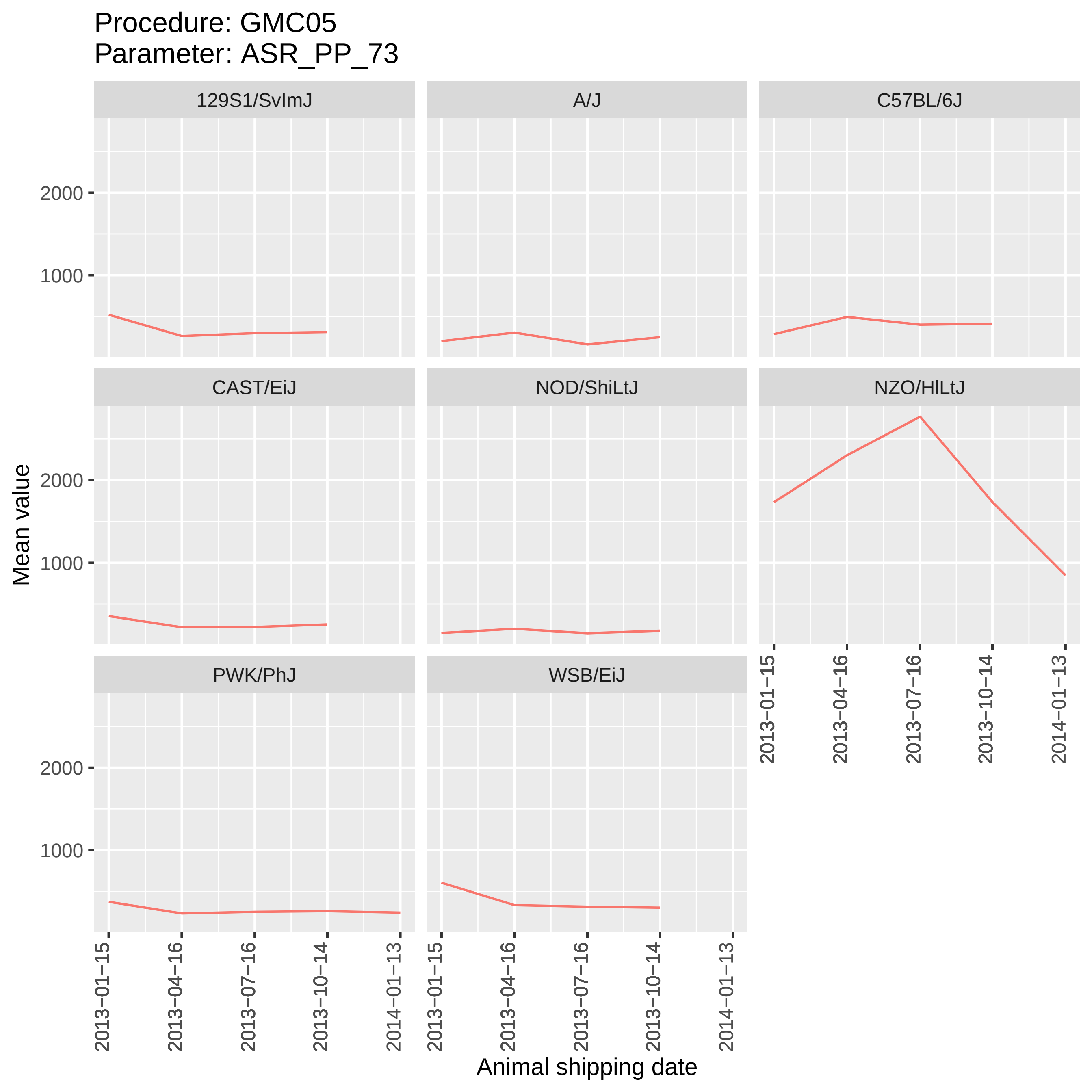
Procedure: GMC05
Parameter: ASR_PP_73
129S1/SvImJ
A/J
C57BL/6J
CAST/EiJ
NOD/ShiLtJ
NZO/HlLtJ
PWK/PhJ
WSB/EiJ
Mean value
1000
2000
2013−01−15
2013−04−16
2013−07−16
2013−10−14
2014−01−13
Animal shipping date

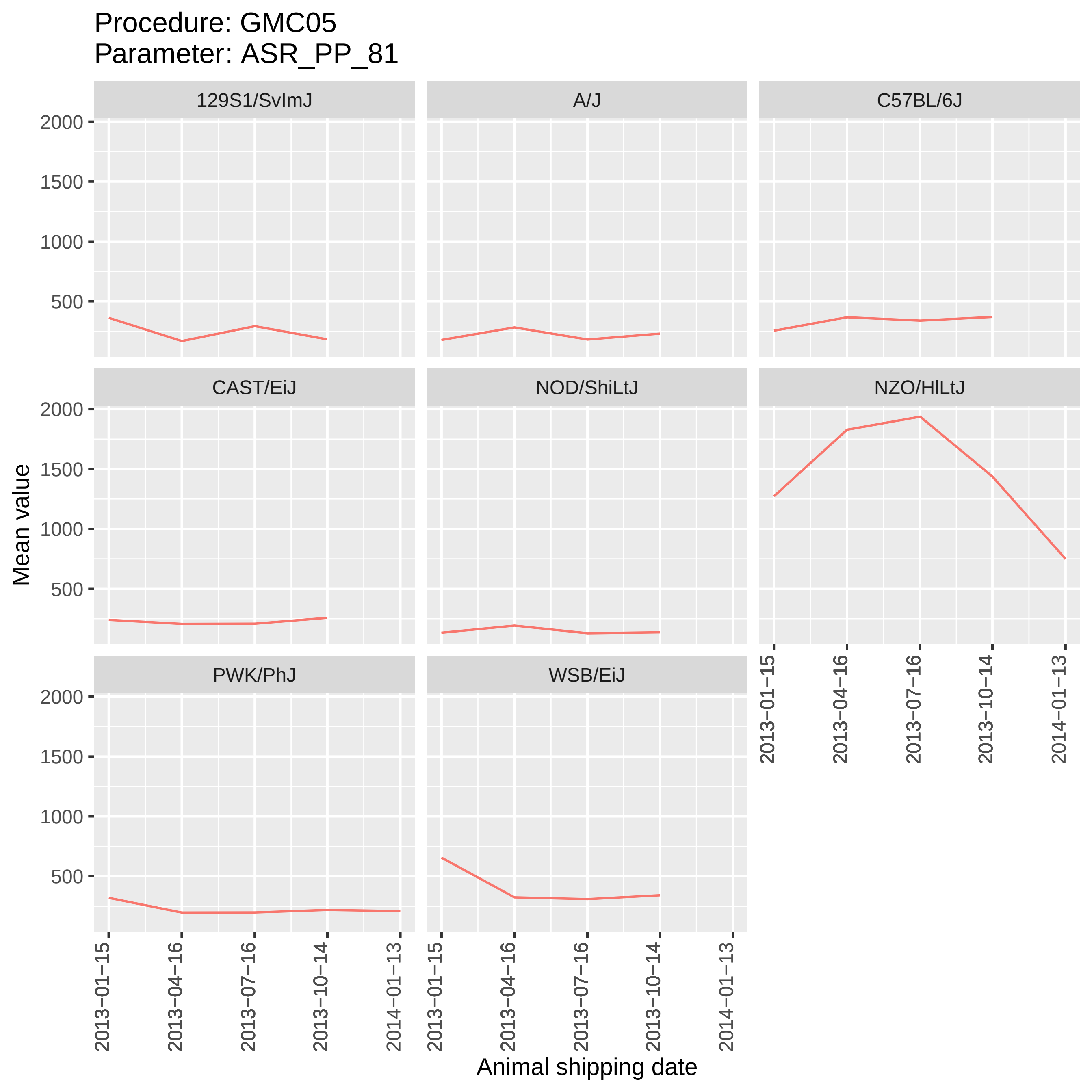

Procedure: GMC05
Parameter: ASR_PP_81
129S1/SvImJ
A/J
C57BL/6J
CAST/EiJ
NOD/ShiLtJ
NZO/HlLtJ
PWK/PhJ
WSB/EiJ
Mean value
500
1000
1500
2000
2013−01−15
2013−04−16
2013−07−16
2013−10−14
2014−01−13
Animal shipping date

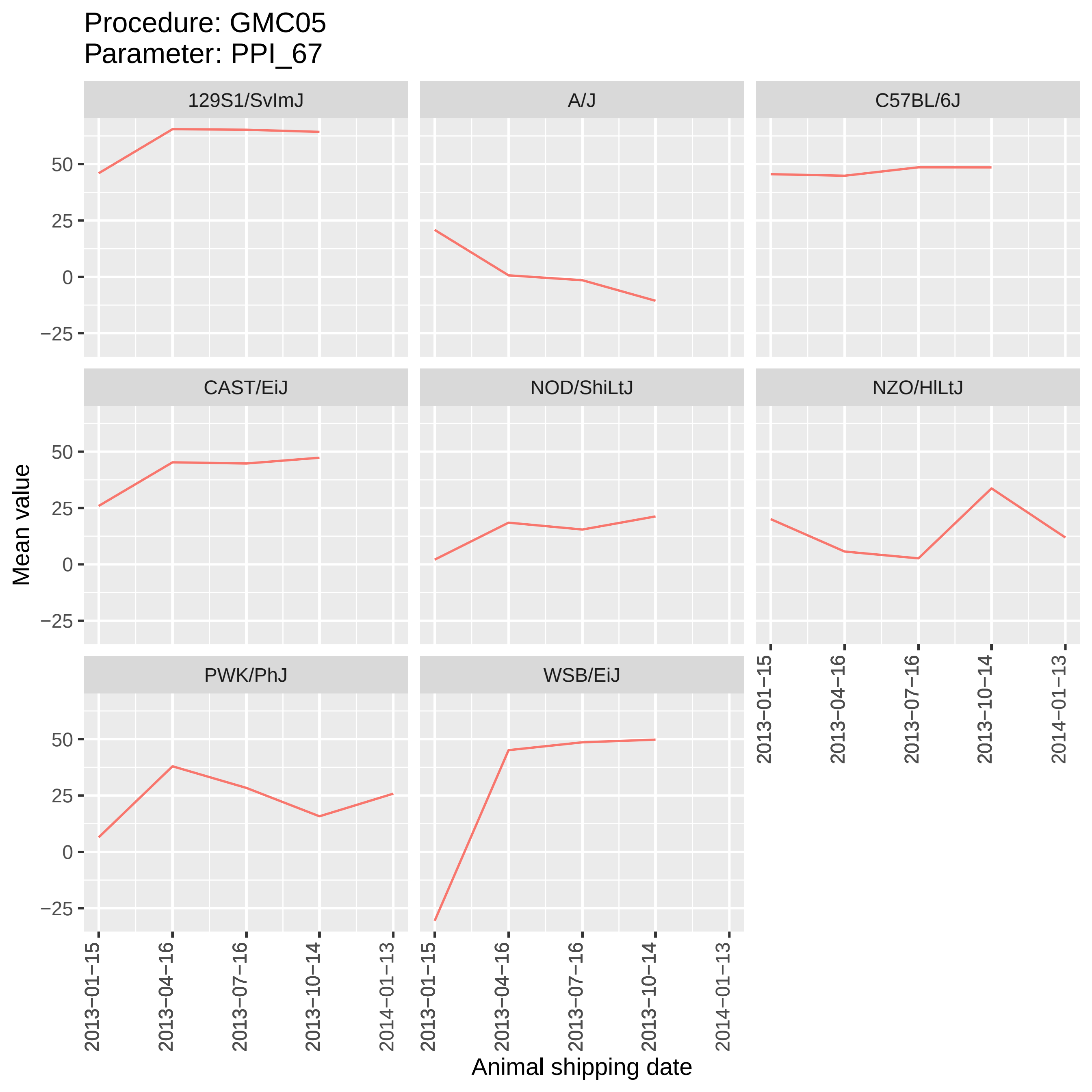
Procedure: GMC05
Parameter: PPI_67
129S1/SvImJ
A/J
C57BL/6J
CAST/EiJ
NOD/ShiLtJ
NZO/HlLtJ
PWK/PhJ
WSB/EiJ
Mean value
50
25
0
−25
2013−01−15
2013−04−16
2013−07−16
2013−10−14
2014−01−13
Animal shipping date

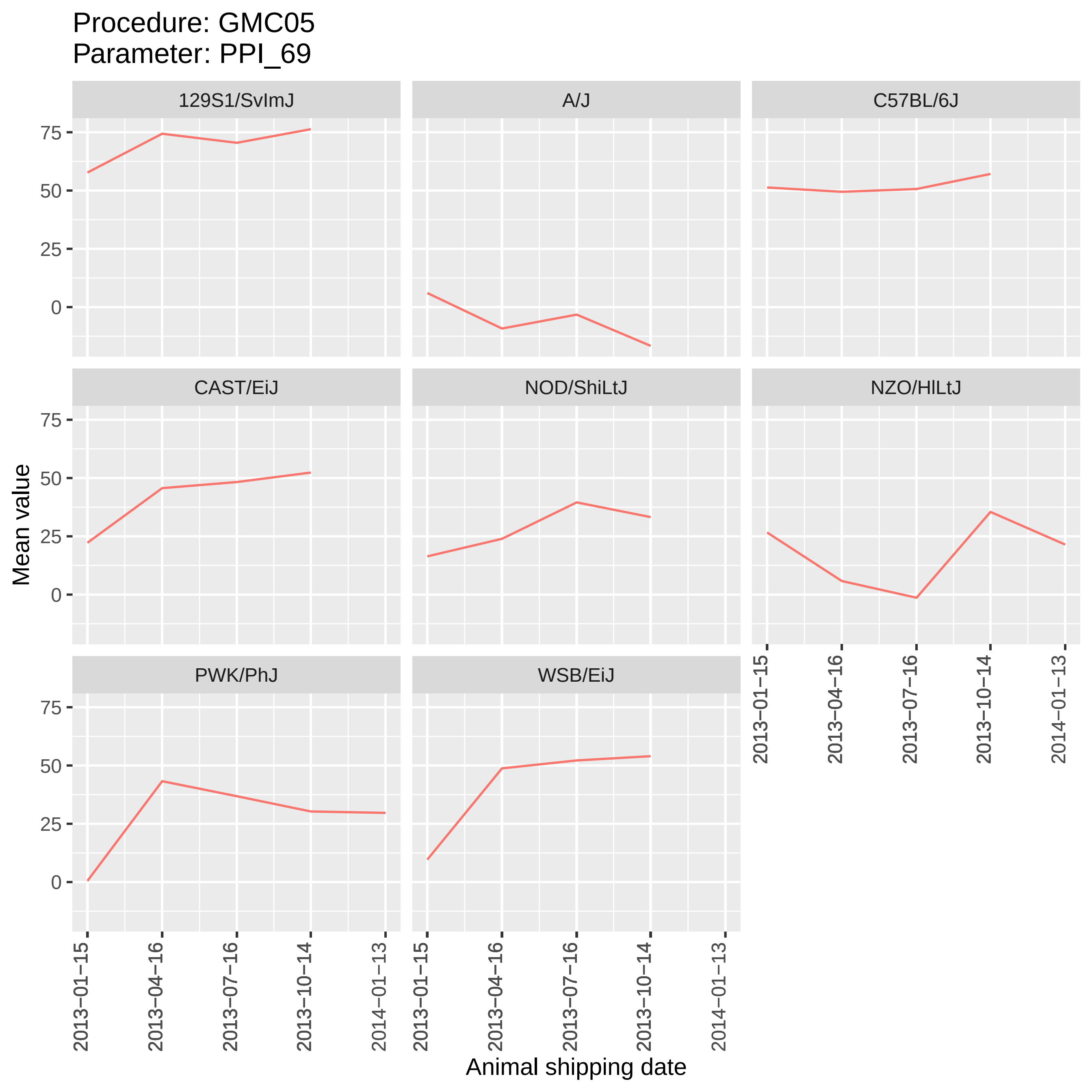

Procedure: GMC05
Parameter: PPI_69
129S1/SvImJ
A/J
C57BL/6J
CAST/EiJ
NOD/ShiLtJ
NZO/HlLtJ
PWK/PhJ
WSB/EiJ
Mean value
75
50
25
0
2013−01−15
2013−04−16
2013−07−16
2013−10−14
2014−01−13
Animal shipping date

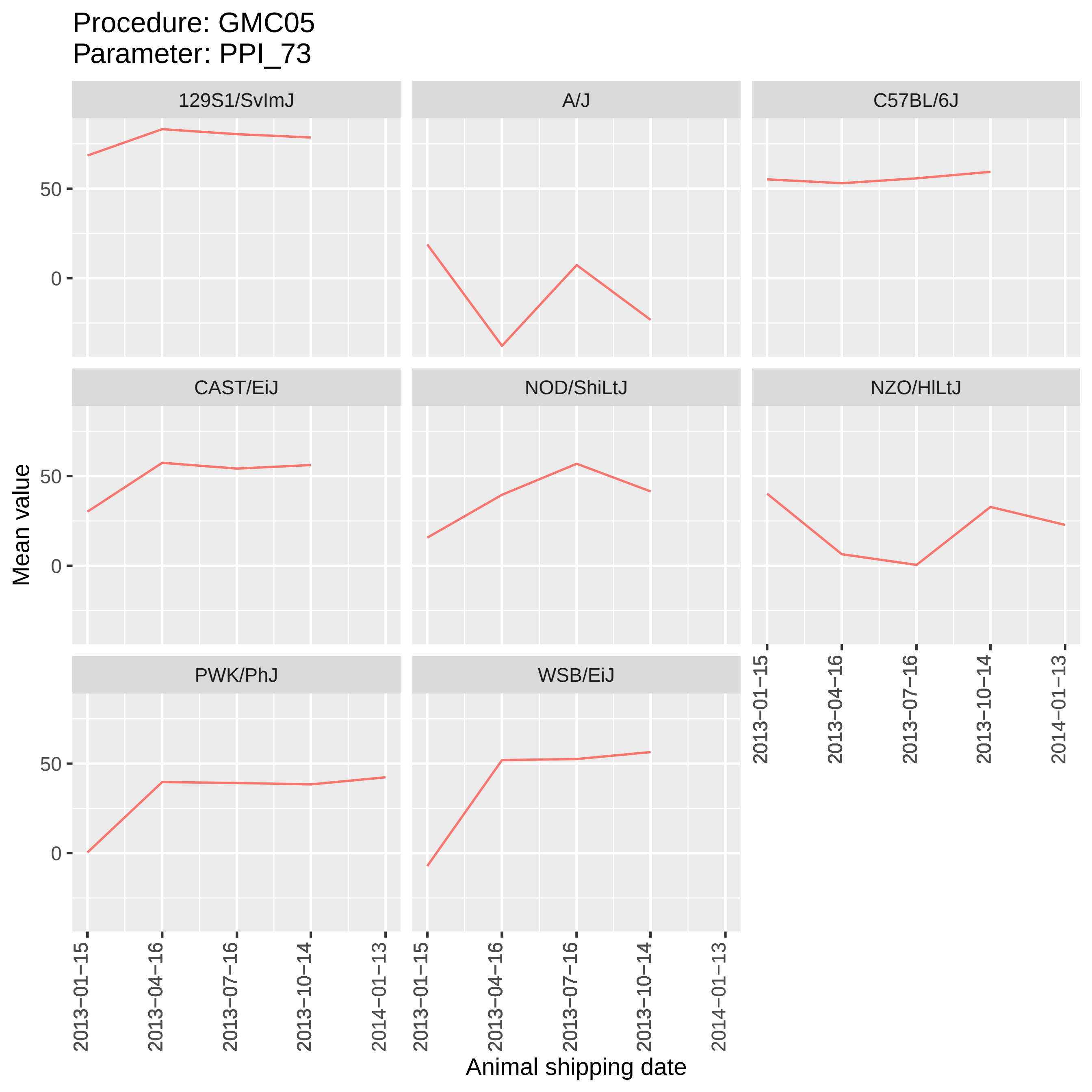

Procedure: GMC05
Parameter: PPI_73
129S1/SvImJ
A/J
C57BL/6J
CAST/EiJ
NOD/ShiLtJ
NZO/HlLtJ
PWK/PhJ
WSB/EiJ
Mean value
50
0
2013−01−15
2013−04−16
2013−07−16
2013−10−14
2014−01−13
Animal shipping date

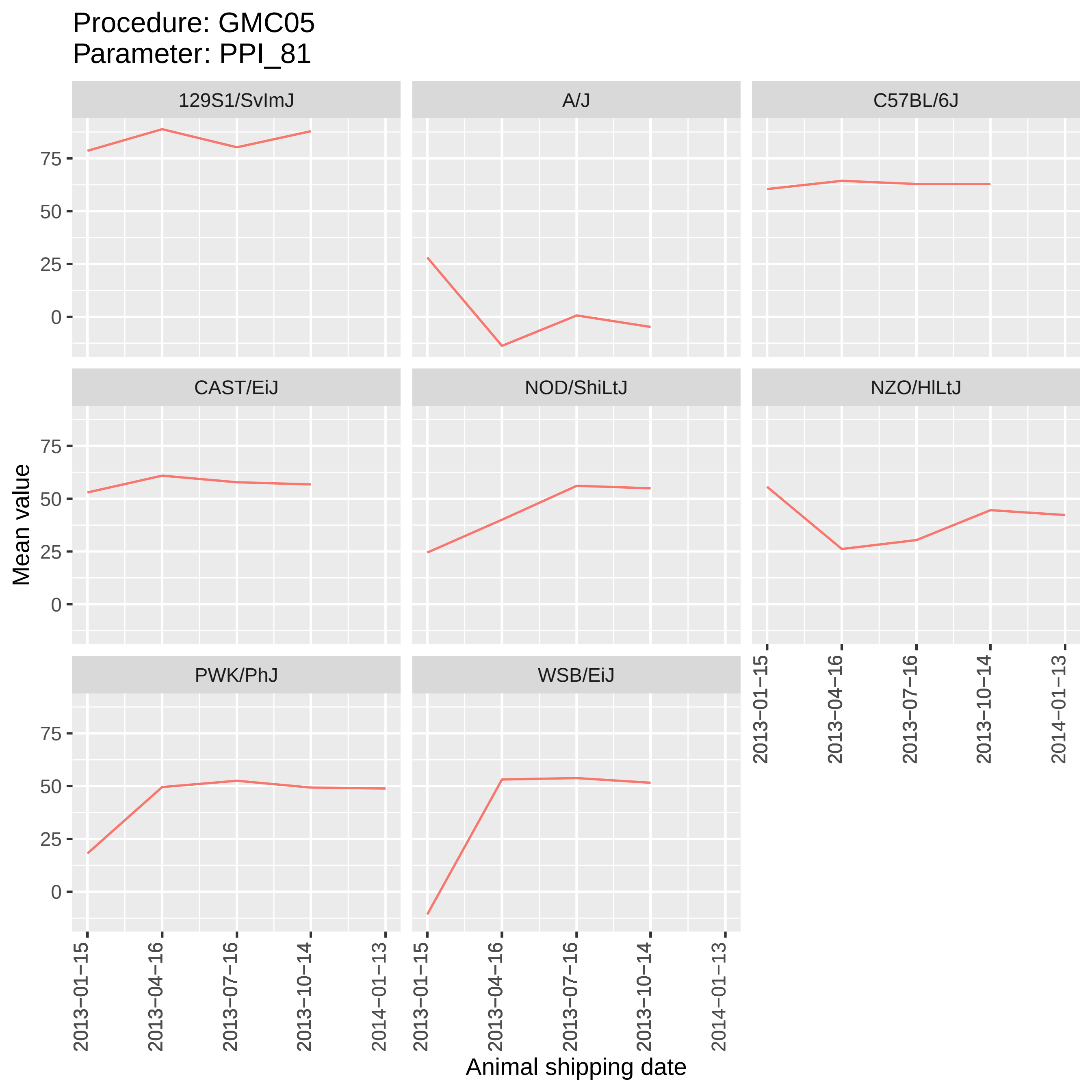

Procedure: GMC05
Parameter: PPI_81
129S1/SvImJ
A/J
C57BL/6J
CAST/EiJ
NOD/ShiLtJ
NZO/HlLtJ
PWK/PhJ
WSB/EiJ
Mean value
0
25
50
75
2013−01−15
2013−04−16
2013−07−16
2013−10−14
2014−01−13
Animal shipping date

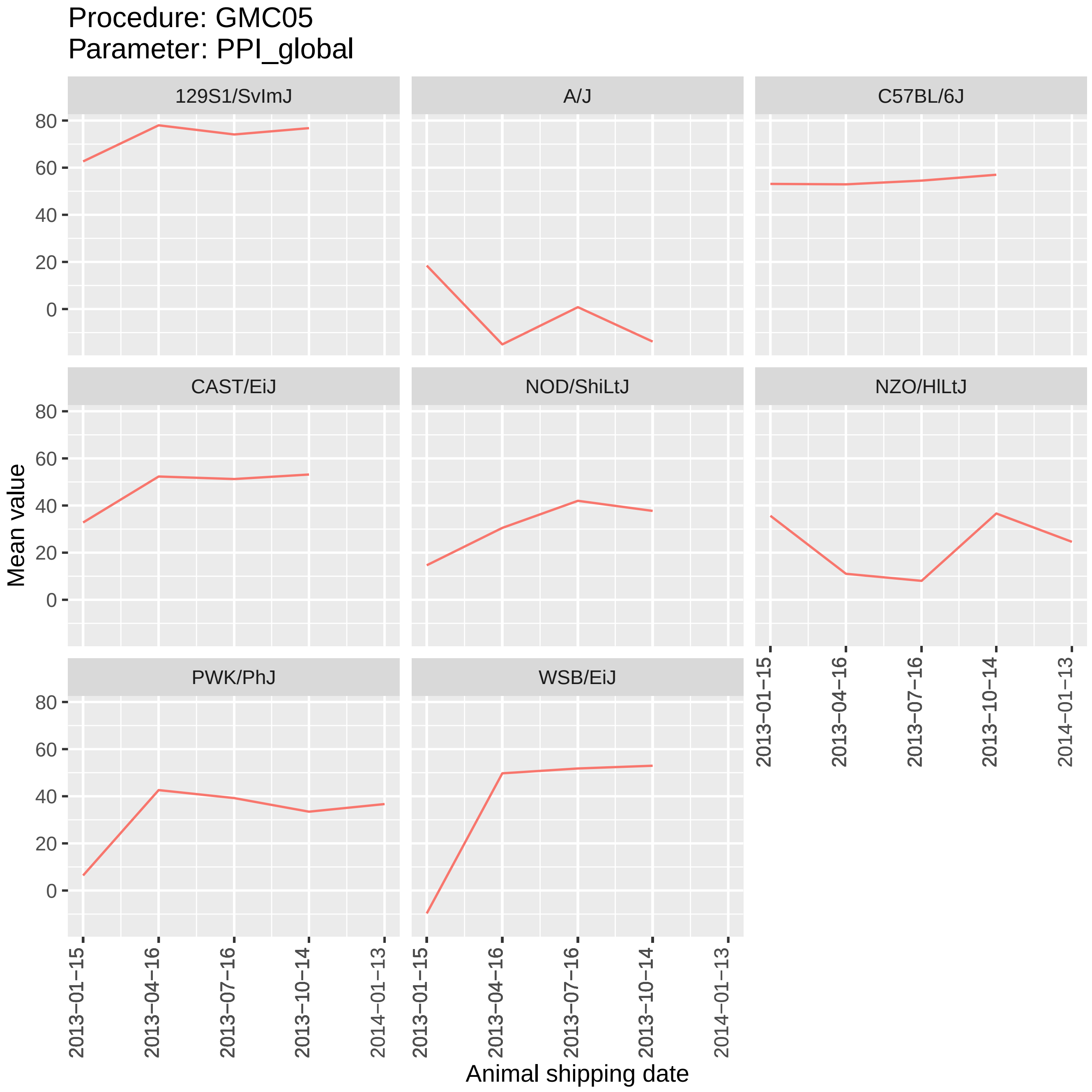

Procedure: GMC05
Parameter: PPI_global
129S1/SvImJ
A/J
C57BL/6J
CAST/EiJ
NOD/ShiLtJ
NZO/HlLtJ
PWK/PhJ
WSB/EiJ
Mean value
80
60
40
20
0
2013−01−15
2013−04−16
2013−07−16
2013−10−14
2014−01−13
Animal shipping date

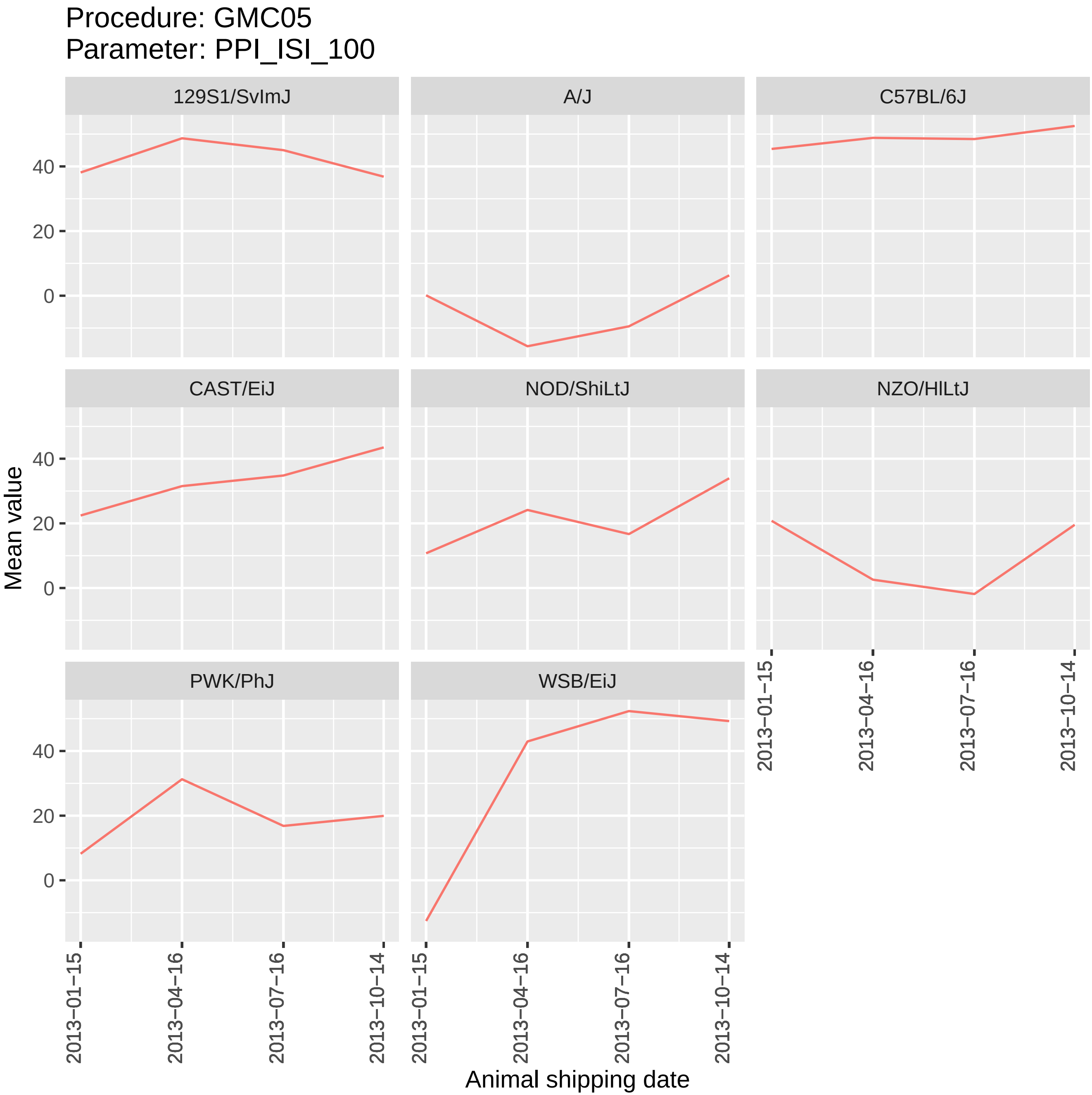

Procedure: GMC05
Parameter: PPI_ISI_100
129S1/SvImJ
A/J
C57BL/6J
CAST/EiJ
NOD/ShiLtJ
NZO/HlLtJ
PWK/PhJ
WSB/EiJ
Mean value
0
20
40
2013−01−15
2013−04−16
2013−07−16
2013−10−14
Animal shipping date

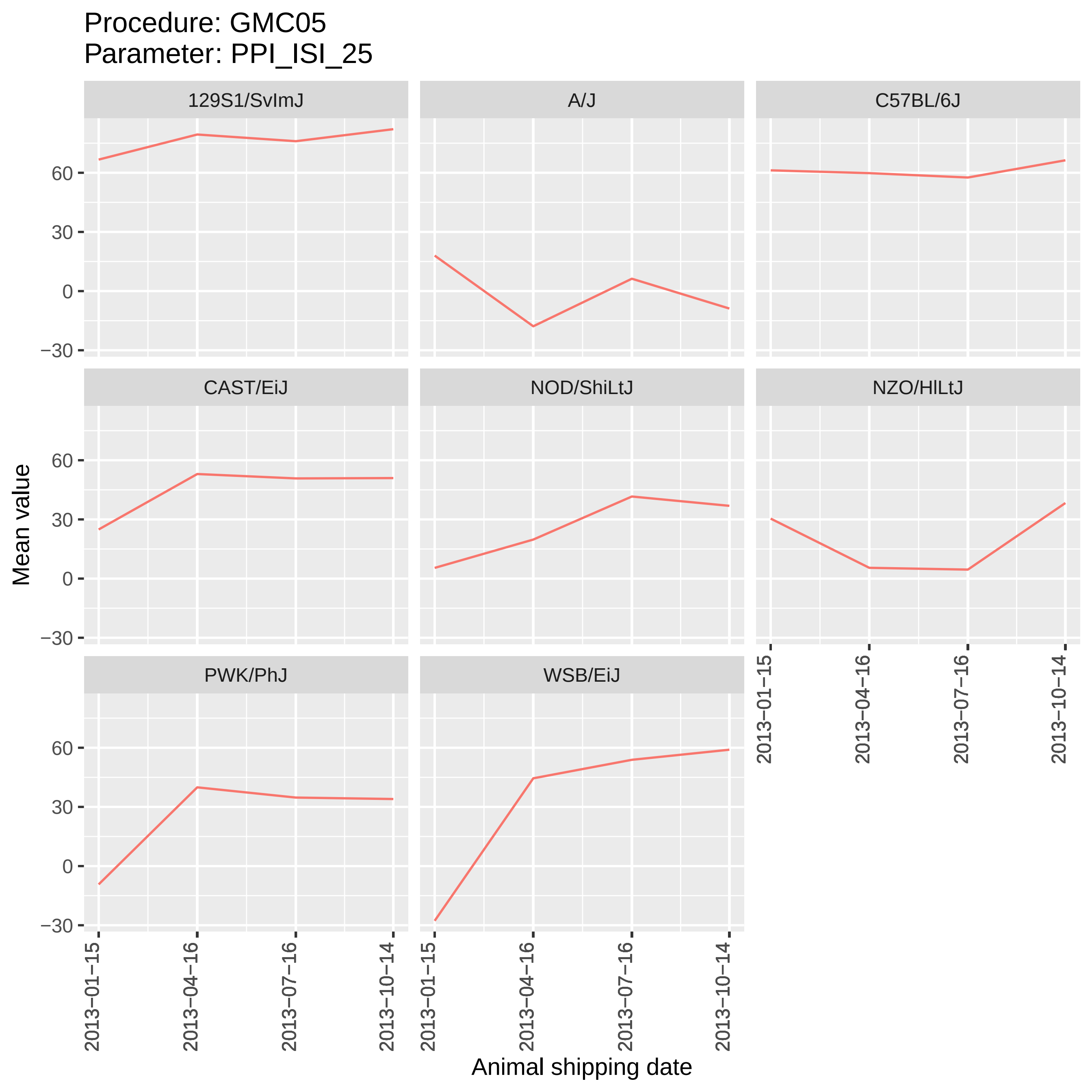

Procedure: GMC05
Parameter: PPI_ISI_25
129S1/SvImJ
A/J
C57BL/6J
CAST/EiJ
NOD/ShiLtJ
NZO/HlLtJ
PWK/PhJ
WSB/EiJ
Mean value
60
30
0
−30
2013−01−15
2013−04−16
2013−07−16
2013−10−14
Animal shipping date

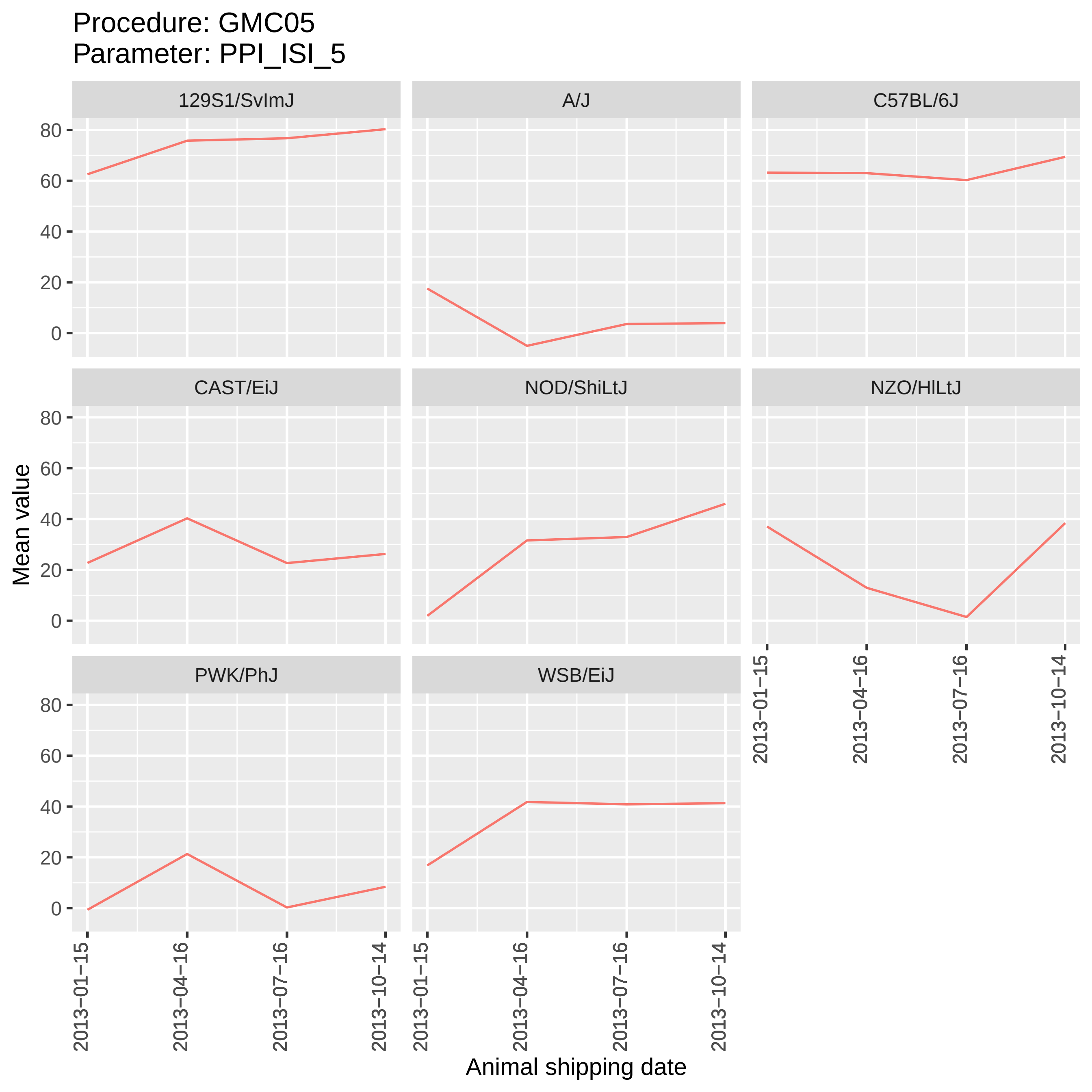
Procedure: GMC05
Parameter: PPI_ISI_5
129S1/SvImJ
A/J
C57BL/6J
CAST/EiJ
NOD/ShiLtJ
NZO/HlLtJ
PWK/PhJ
WSB/EiJ
Mean value
0
20
40
60
80
2013−01−15
2013−04−16
2013−07−16
2013−10−14
Animal shipping date

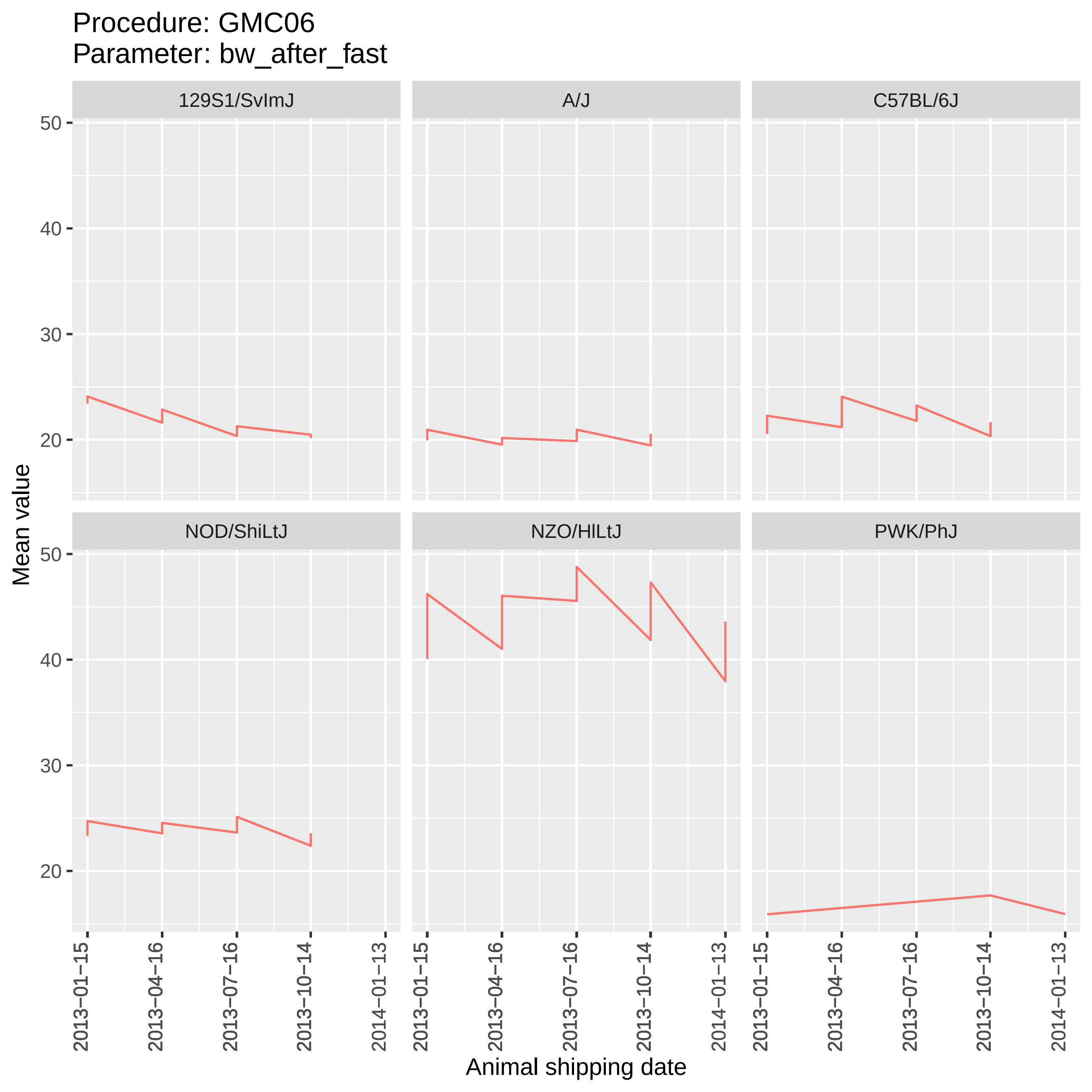

Procedure: GMC06
Parameter: bw_after_fast
129S1/SvImJ
A/J
C57BL/6J
NOD/ShiLtJ
NZO/HlLtJ
PWK/PhJ
Mean value
50
40
30
20
2013−01−15
2013−04−16
2013−07−16
2013−10−14
2014−01−13
Animal shipping date

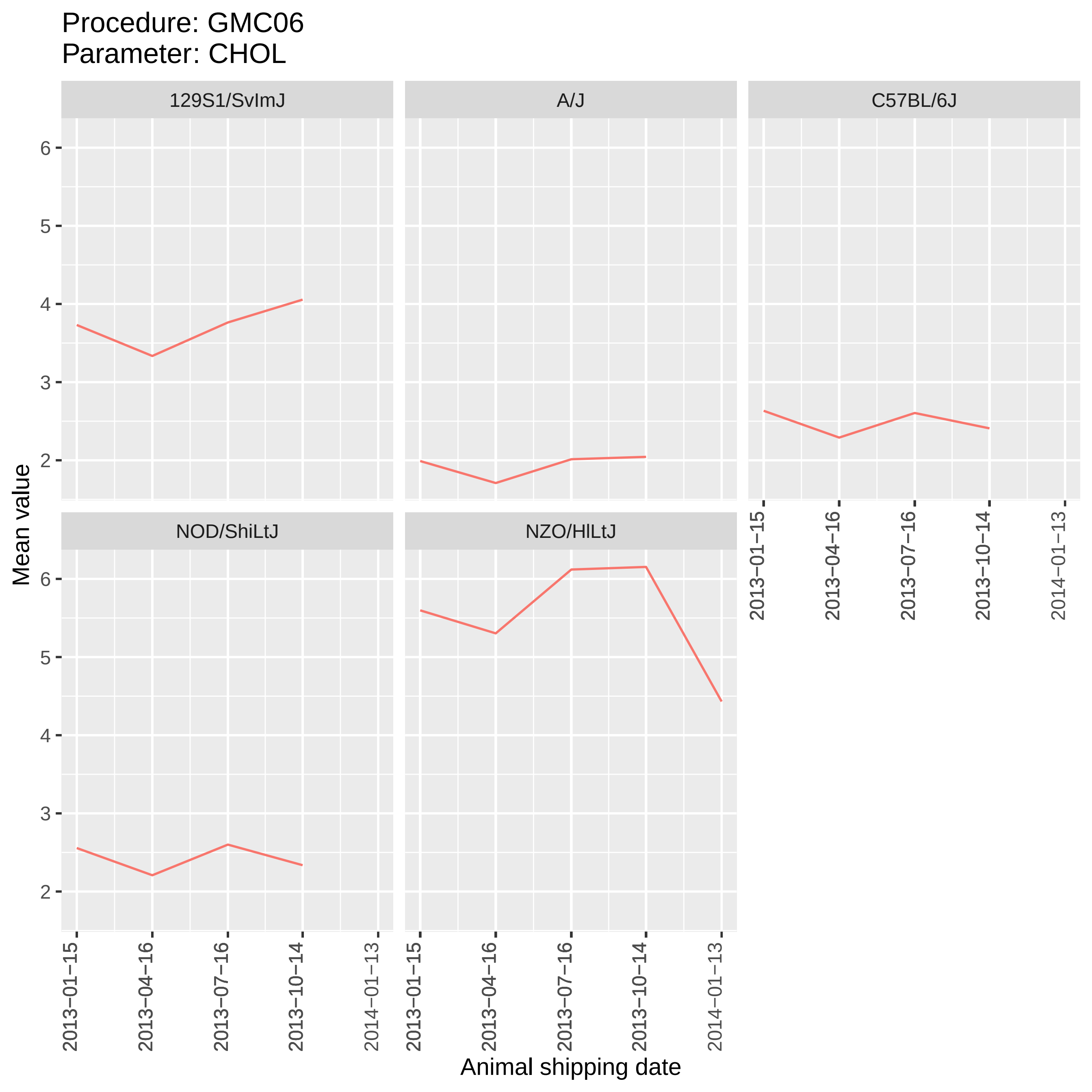

Procedure: GMC06
Parameter: CHOL
129S1/SvImJ
A/J
C57BL/6J
NOD/ShiLtJ
NZO/HlLtJ
Mean value
Animal shipping date
2
3
4
5
6
2013−01−15
2013−04−16
2013−07−16
2013−10−14
2014−01−13

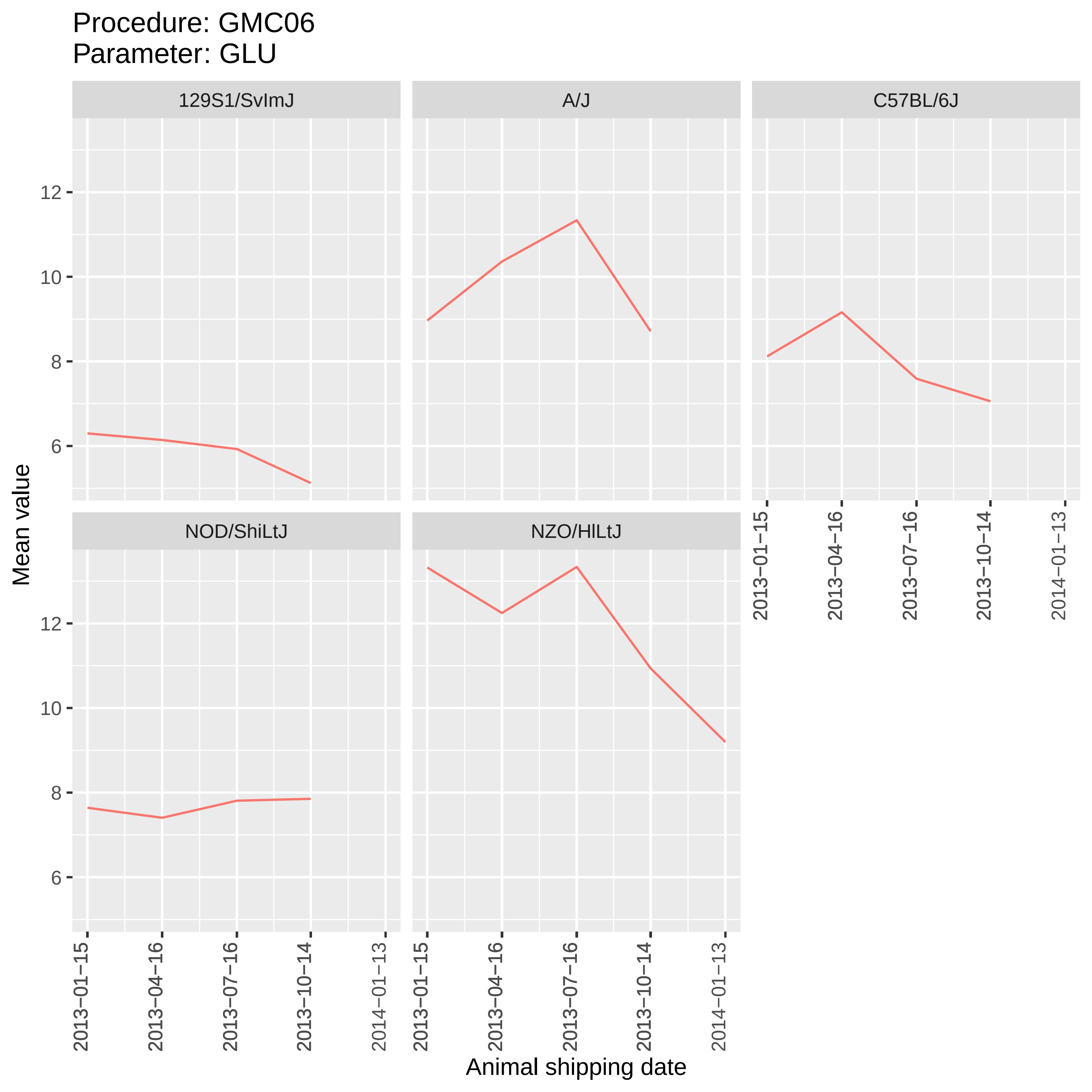

Procedure: GMC06
Parameter: GLU
129S1/SvImJ
A/J
C57BL/6J
NOD/ShiLtJ
NZO/HlLtJ
Mean value
12
10
8
6
2013−01−15
2013−04−16
2013−07−16
2013−10−14
2014−01−13
Animal shipping date

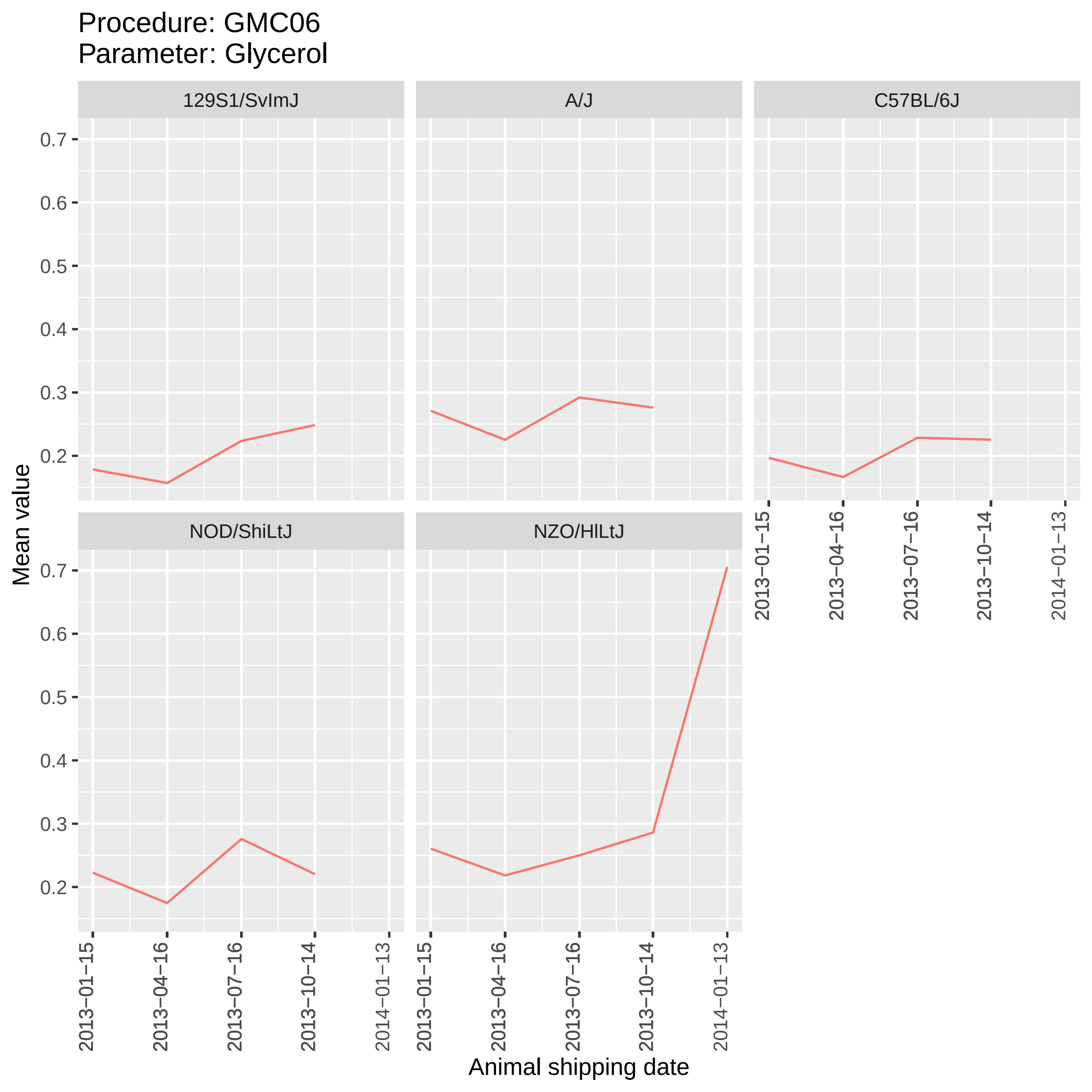

Procedure: GMC06
Parameter: Glycerol
129S1/SvImJ
A/J
C57BL/6J
NOD/ShiLtJ
NZO/HlLtJ
Mean value
0.7
0.6
0.5
0.4
0.3
0.2
2013−01−15
2013−04−16
2013−07−16
2013−10−14
2014−01−13
Animal shipping date

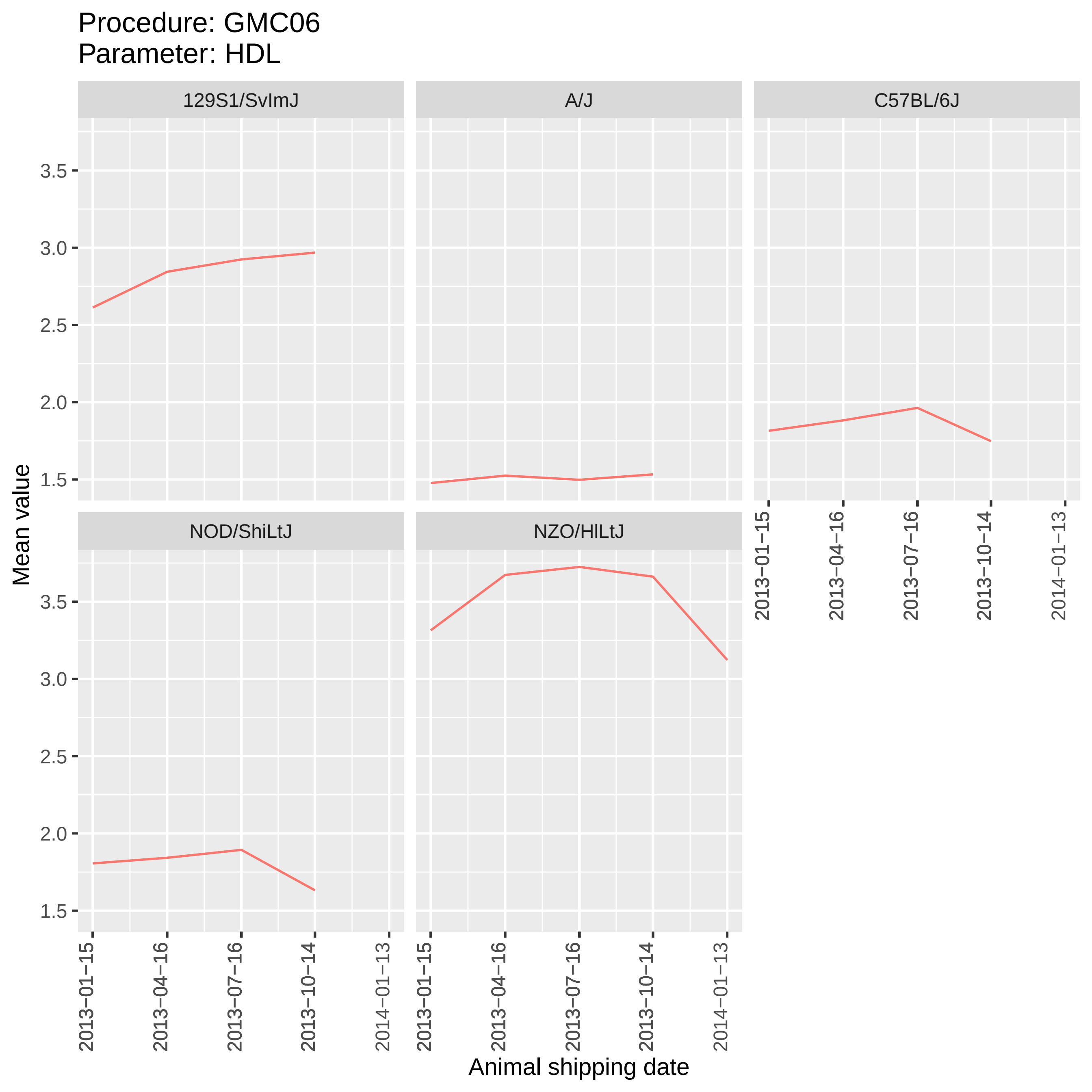

Procedure: GMC06
Parameter: HDL
129S1/SvImJ
A/J
C57BL/6J
NOD/ShiLtJ
NZO/HlLtJ
Mean value
3.5
3.0
2.5
2.0
1.5
2013−01−15
2013−04−16
2013−07−16
2013−10−14
2014−01−13
Animal shipping date

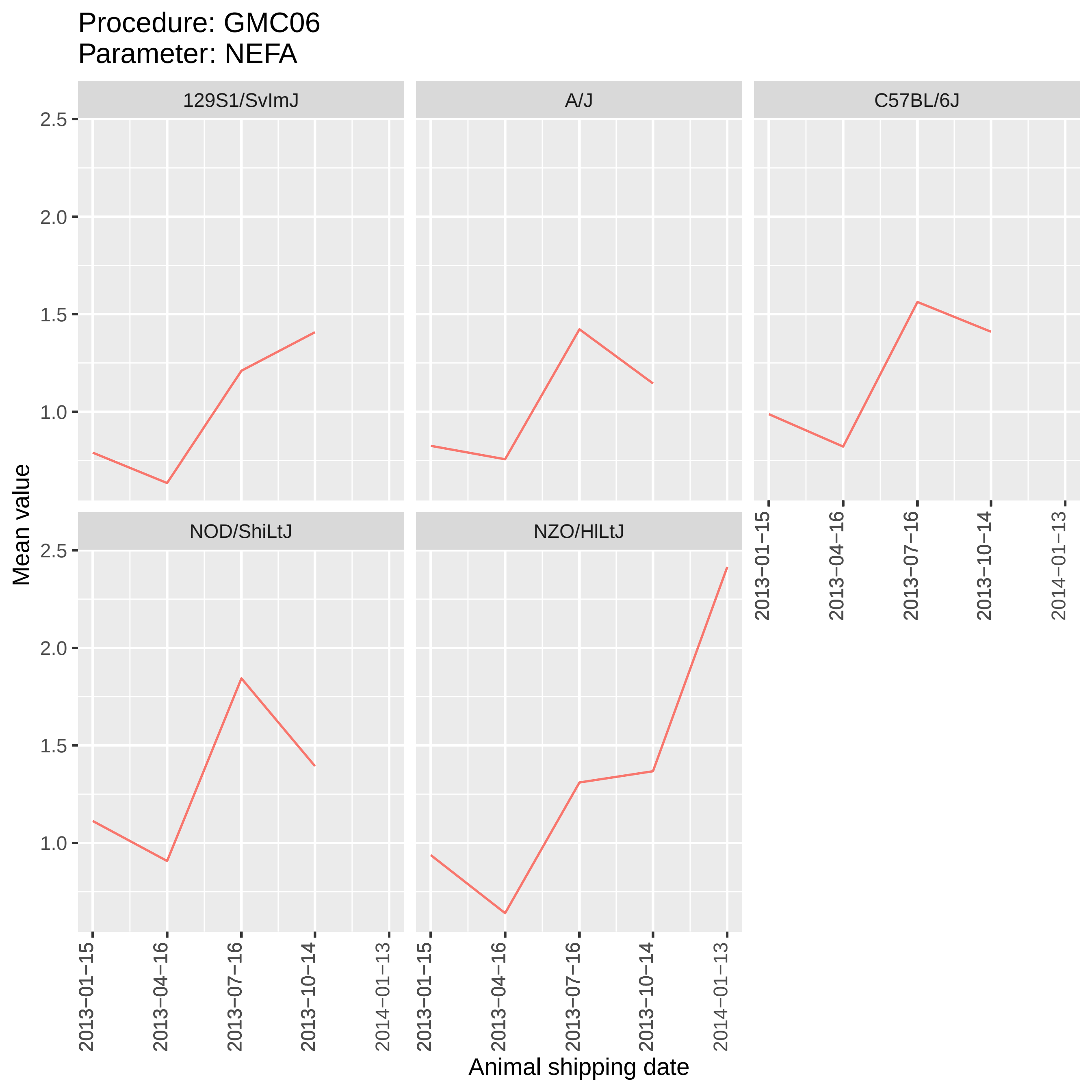
Procedure: GMC06
Parameter: NEFA
129S1/SvImJ
A/J
C57BL/6J
NOD/ShiLtJ
NZO/HlLtJ
Mean value
2.5
2.0
1.5
1.0
2013−01−15
2013−04−16
2013−07−16
2013−10−14
2014−01−13
Animal shipping date

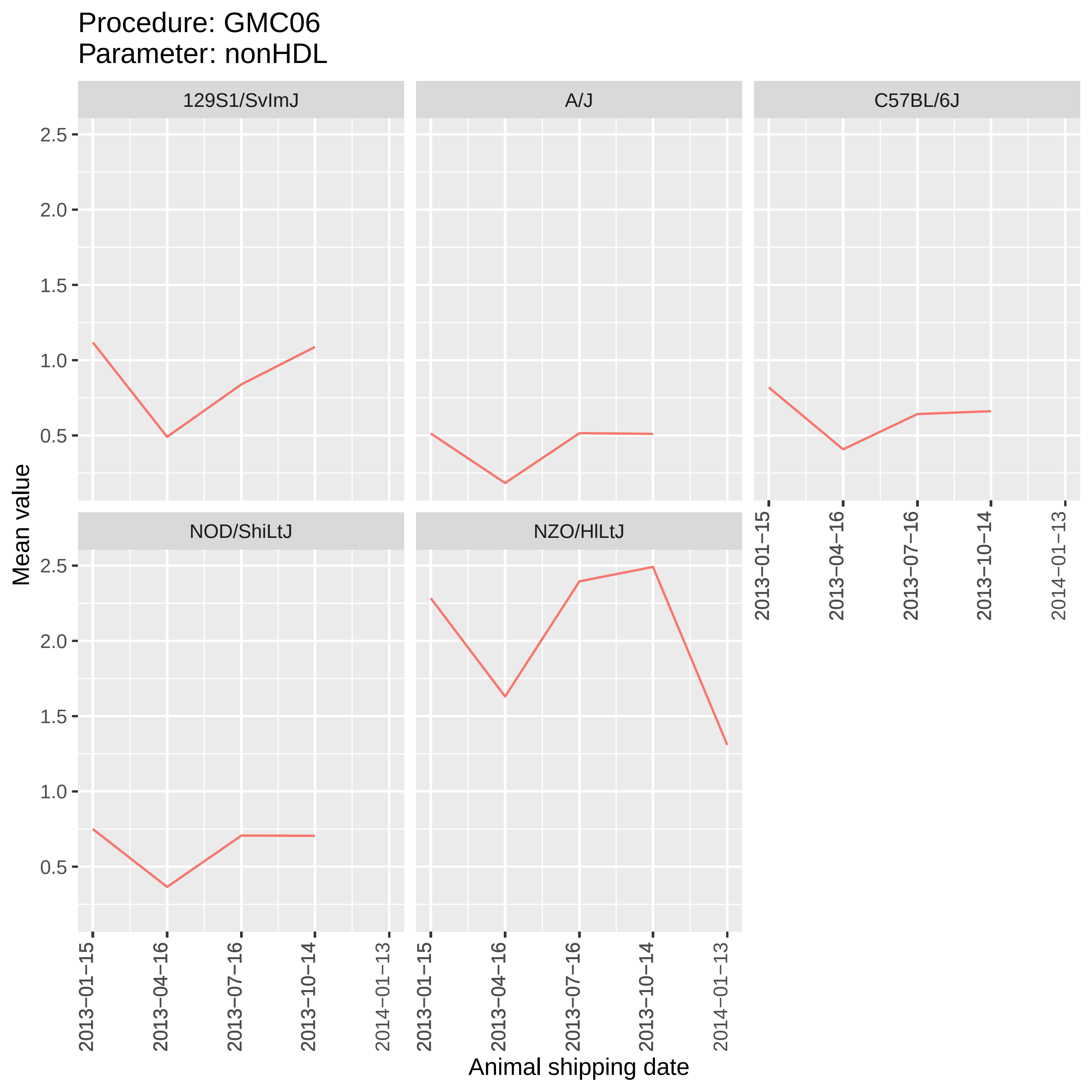

Procedure: GMC06
Parameter: nonHDL
129S1/SvImJ
A/J
C57BL/6J
NOD/ShiLtJ
NZO/HlLtJ
Mean value
2.5
2.0
1.5
1.0
0.5
2013−01−15
2013−04−16
2013−07−16
2013−10−14
2014−01−13
Animal shipping date

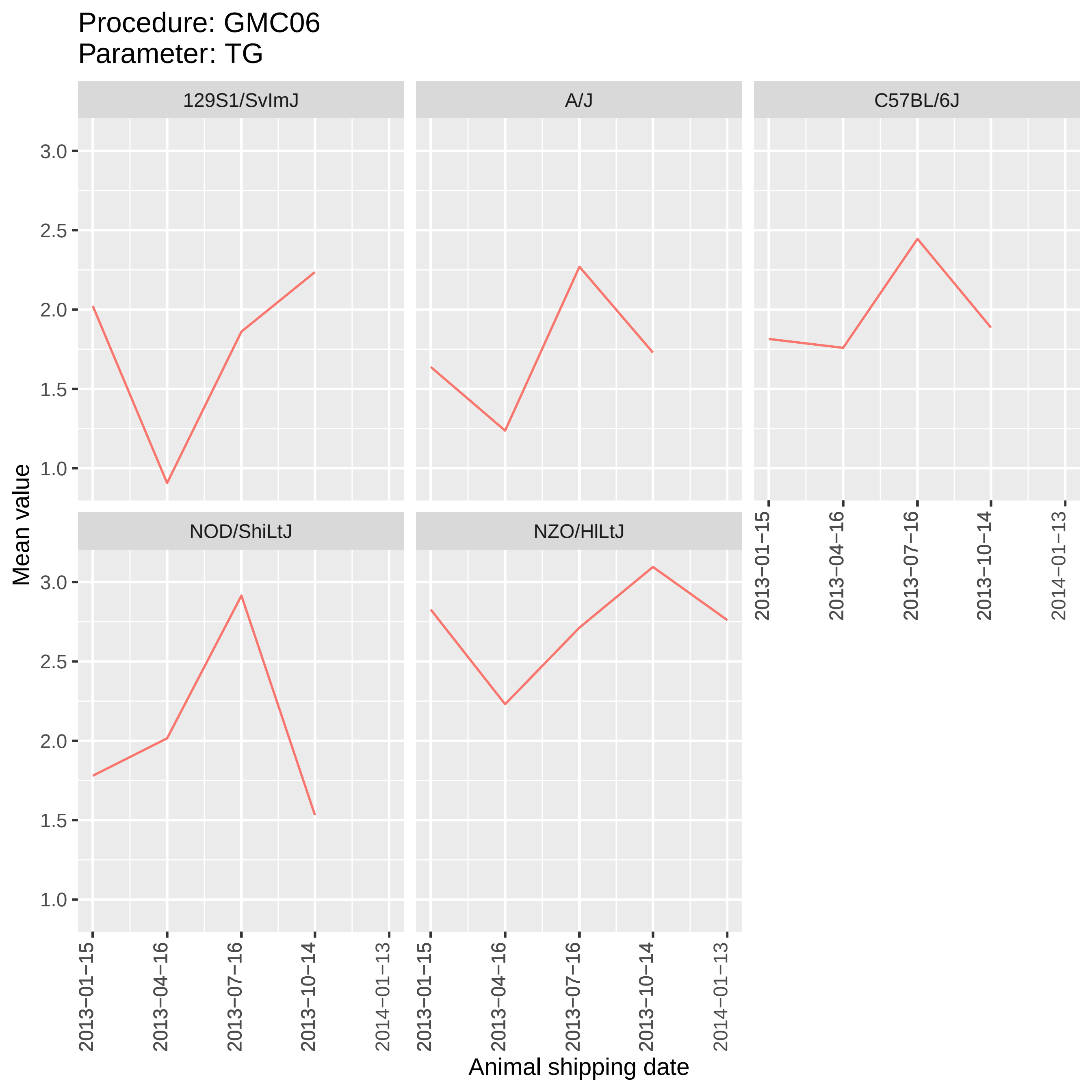

Procedure: GMC06
Parameter: TG
129S1/SvImJ
A/J
C57BL/6J
NOD/ShiLtJ
NZO/HlLtJ
Mean value
3.0
2.5
2.0
1.5
1.0
2013−01−15
2013−04−16
2013−07−16
2013−10−14
2014−01−13
Animal shipping date

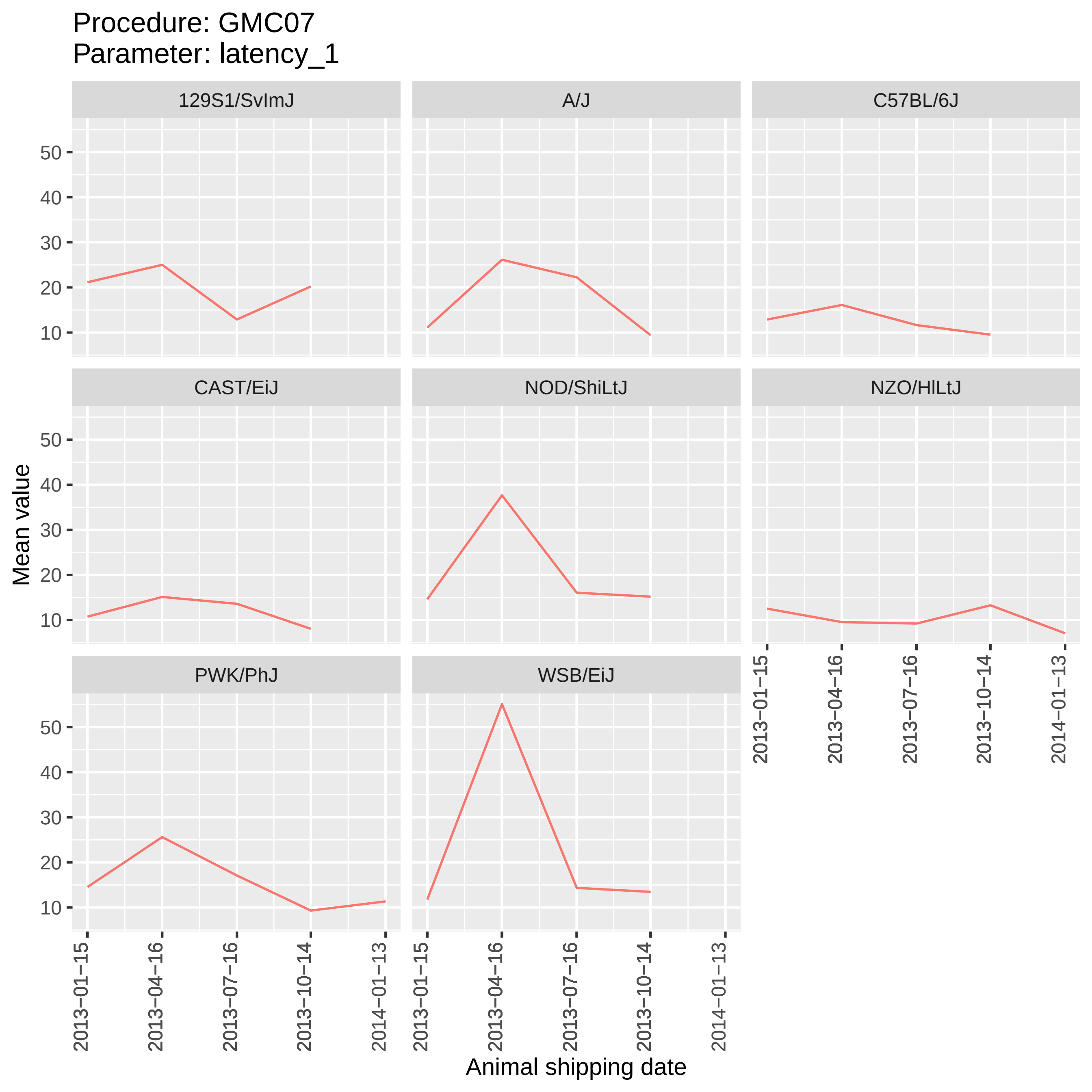

Procedure: GMC07
Parameter: latency_1
129S1/SvImJ
A/J
C57BL/6J
CAST/EiJ
NOD/ShiLtJ
NZO/HlLtJ
PWK/PhJ
WSB/EiJ
Mean value
10
20
30
40
50
2013−01−15
2013−04−16
2013−07−16
2013−10−14
2014−01−13
Animal shipping date

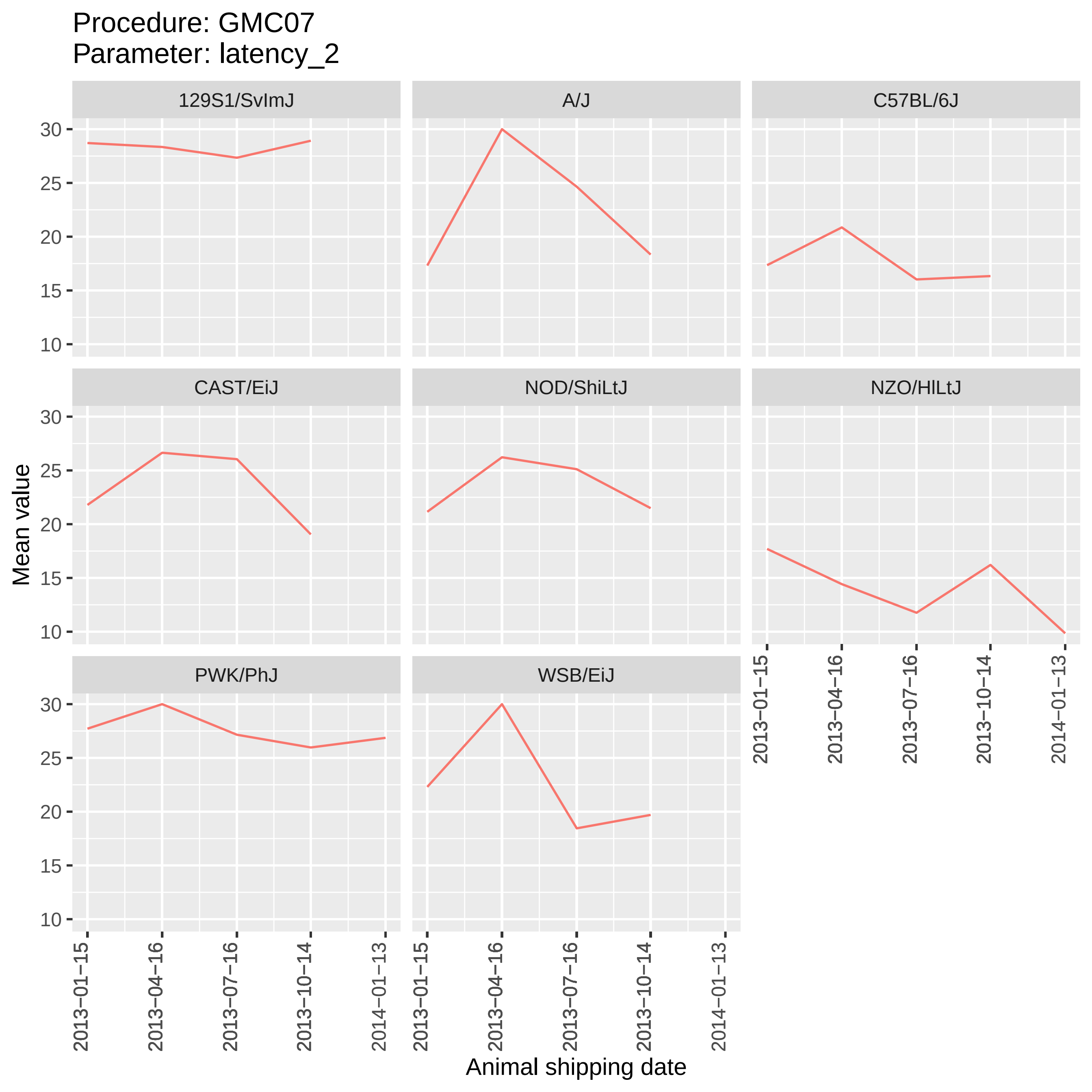

Procedure: GMC07
Parameter: latency_2
129S1/SvImJ
A/J
C57BL/6J
CAST/EiJ
NOD/ShiLtJ
NZO/HlLtJ
PWK/PhJ
WSB/EiJ
Mean value
10
15
20
25
30
2013−01−15
2013−04−16
2013−07−16
2013−10−14
2014−01−13
Animal shipping date

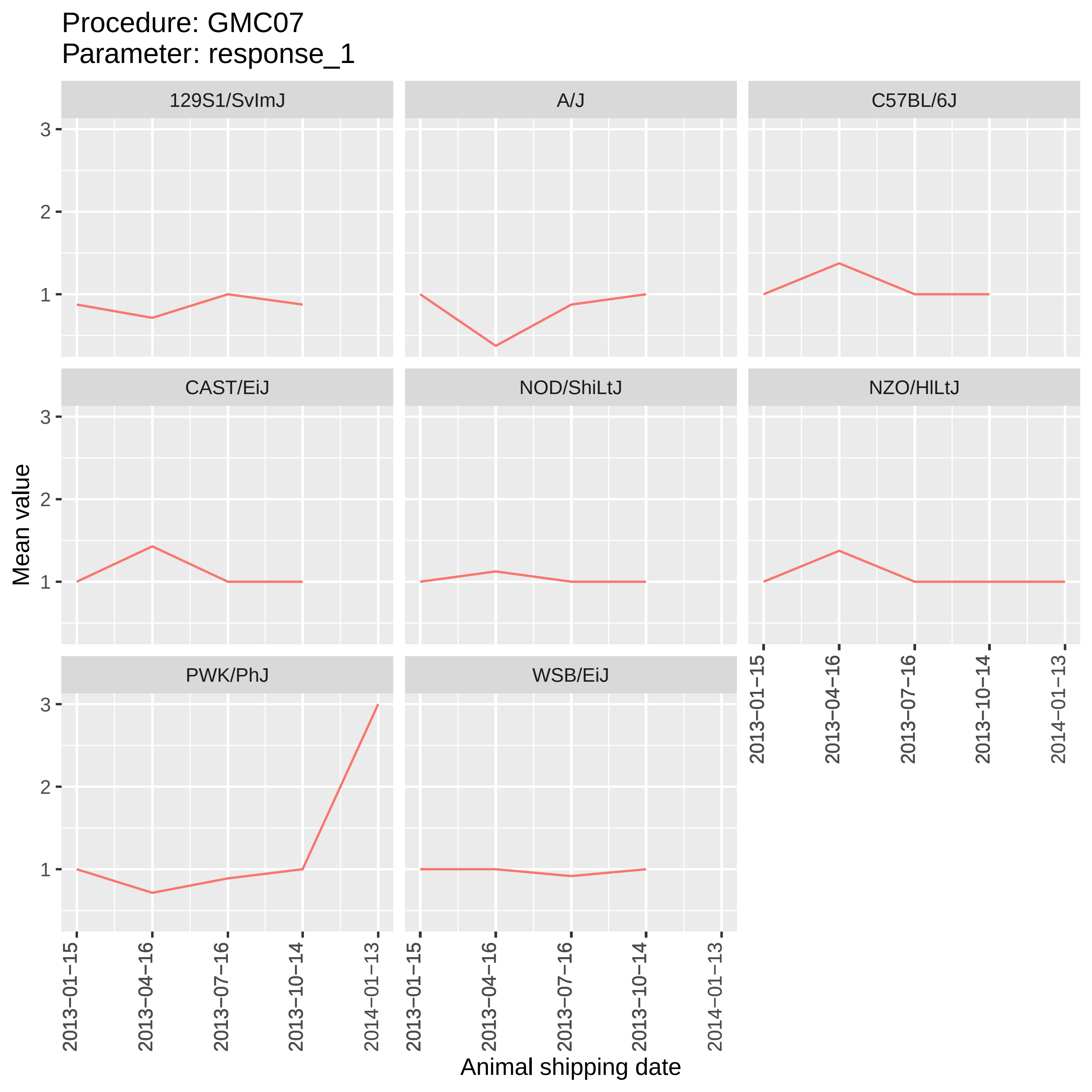
Procedure: GMC07
Parameter: response_1
129S1/SvImJ
A/J
C57BL/6J
CAST/EiJ
NOD/ShiLtJ
NZO/HlLtJ
PWK/PhJ
WSB/EiJ
Mean value
1
2
3
2013−01−15
2013−04−16
2013−07−16
2013−10−14
2014−01−13
Animal shipping date

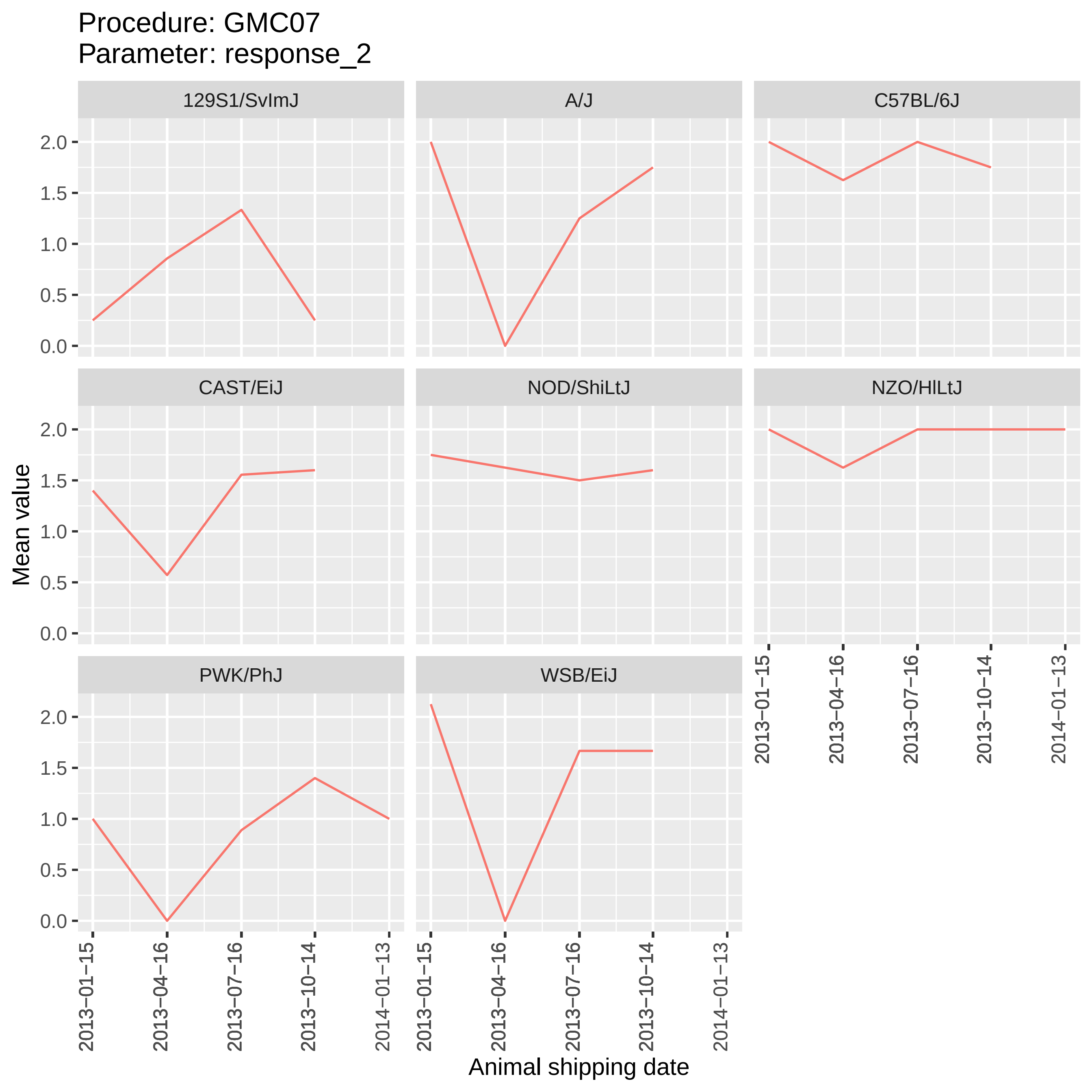

Procedure: GMC07
Parameter: response_2
129S1/SvImJ
A/J
C57BL/6J
CAST/EiJ
NOD/ShiLtJ
NZO/HlLtJ
PWK/PhJ
WSB/EiJ
Mean value
0.0
0.5
1.0
1.5
2.0
2013−01−15
2013−04−16
2013−07−16
2013−10−14
2014−01−13
Animal shipping date

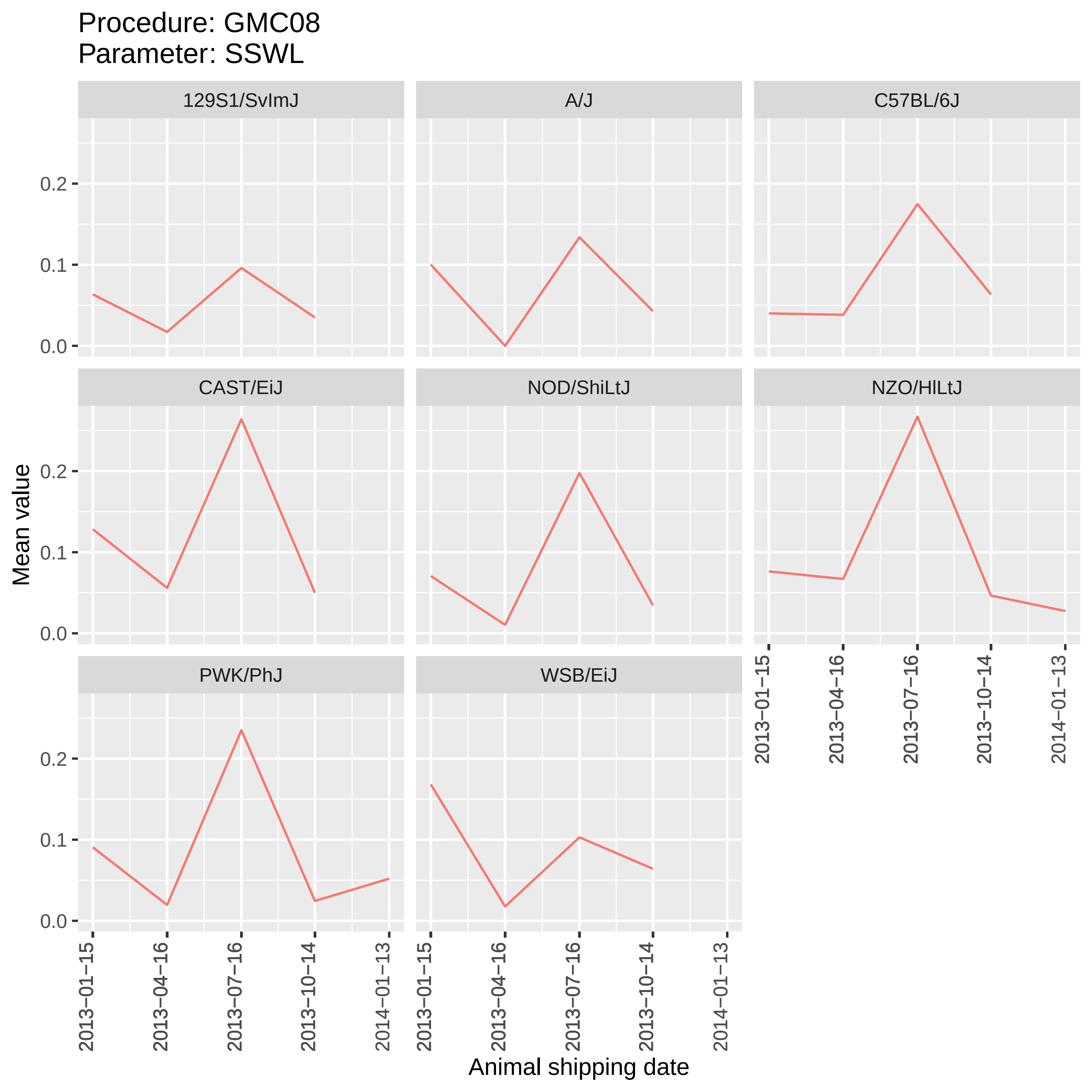

Procedure: GMC08
Parameter: SSWL
129S1/SvImJ
A/J
C57BL/6J
CAST/EiJ
NOD/ShiLtJ
NZO/HlLtJ
PWK/PhJ
WSB/EiJ
Mean value
0.0
0.1
0.2
2013−01−15
2013−04−16
2013−07−16
2013−10−14
2014−01−13
Animal shipping date

Procedure: GMC08
Parameter: TEWL

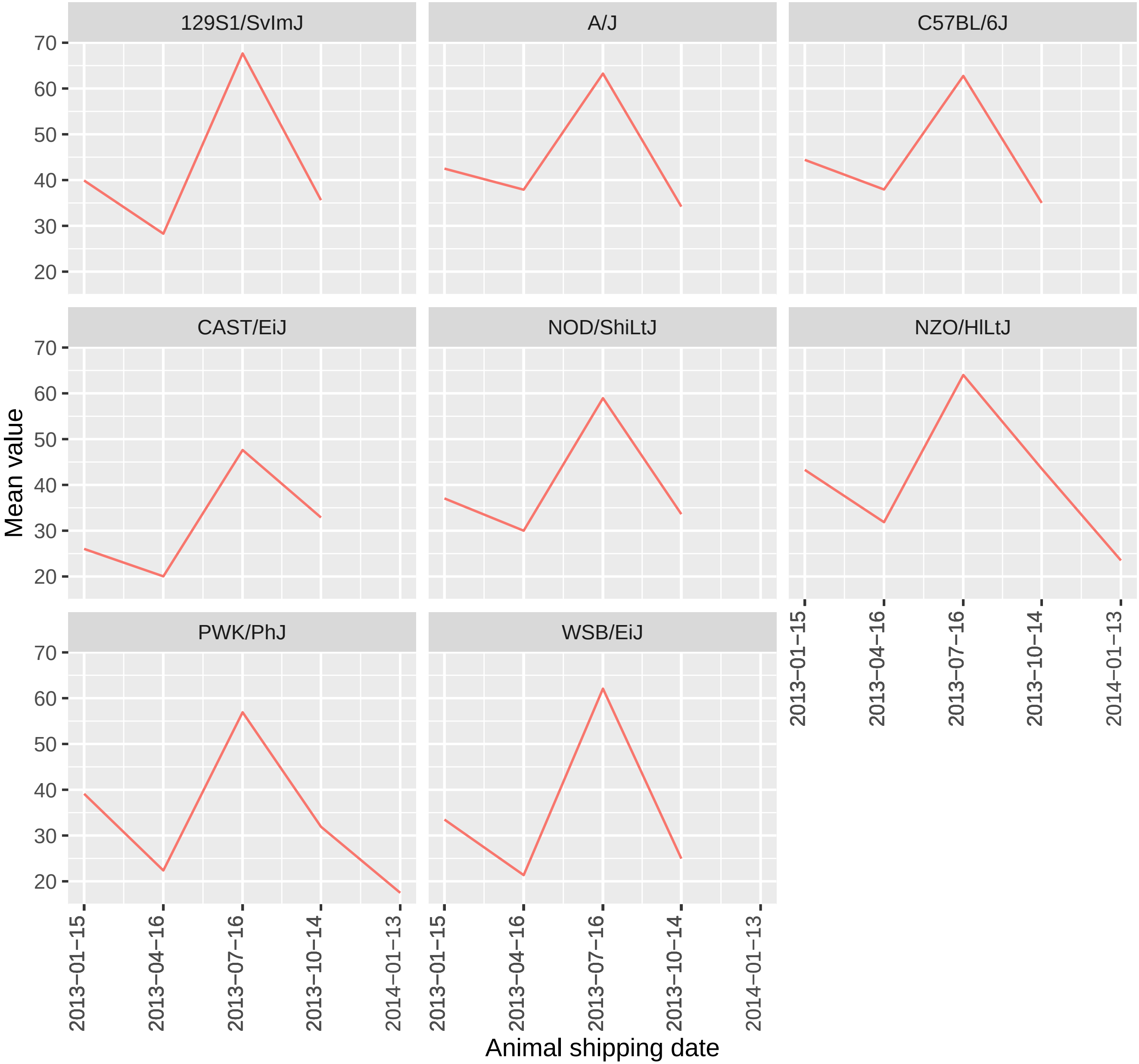

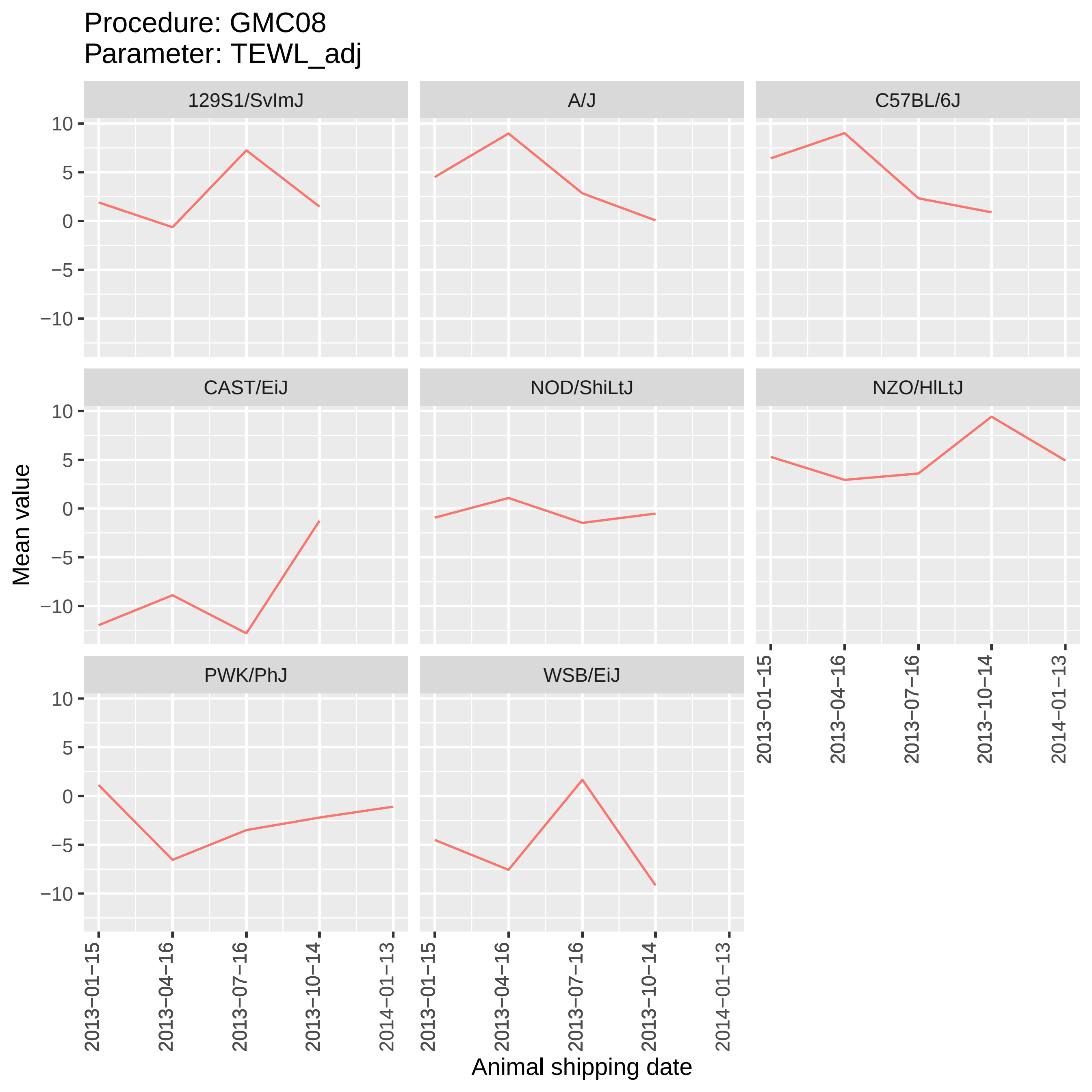

Procedure: GMC08
Parameter: TEWL_adj
129S1/SvImJ
A/J
C57BL/6J
CAST/EiJ
NOD/ShiLtJ
NZO/HlLtJ
PWK/PhJ
WSB/EiJ
10
5
0
−5
−10
Mean value
2013−01−15
2013−04−16
2013−07−16
2013−10−14
2014−01−13
Animal shipping date

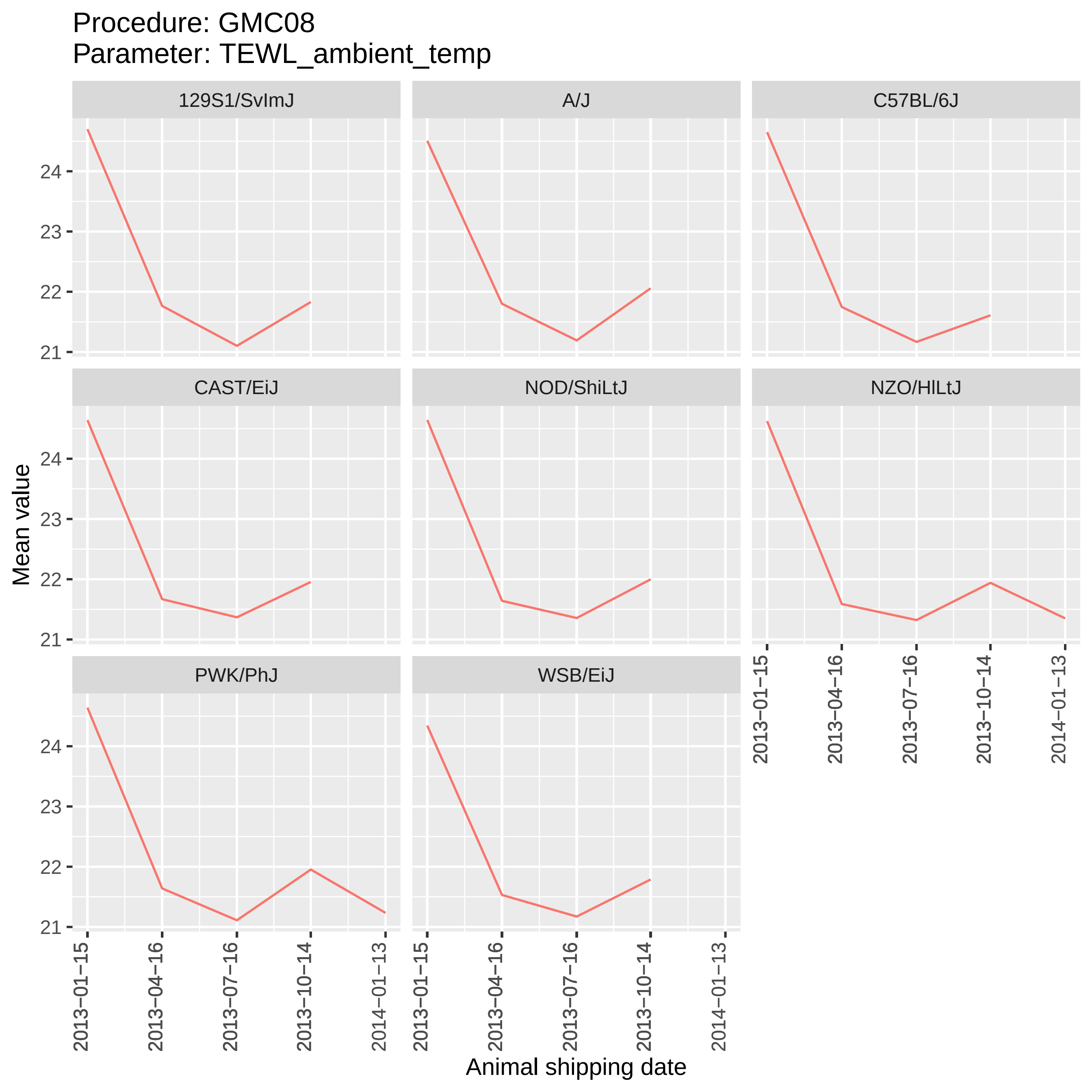

Procedure: GMC08
Parameter: TEWL_ambient_temp
129S1/SvImJ
A/J
C57BL/6J
CAST/EiJ
NOD/ShiLtJ
NZO/HlLtJ
PWK/PhJ
WSB/EiJ
Mean value
21
22
23
24
2013−01−15
2013−04−16
2013−07−16
2013−10−14
2014−01−13
Animal shipping date

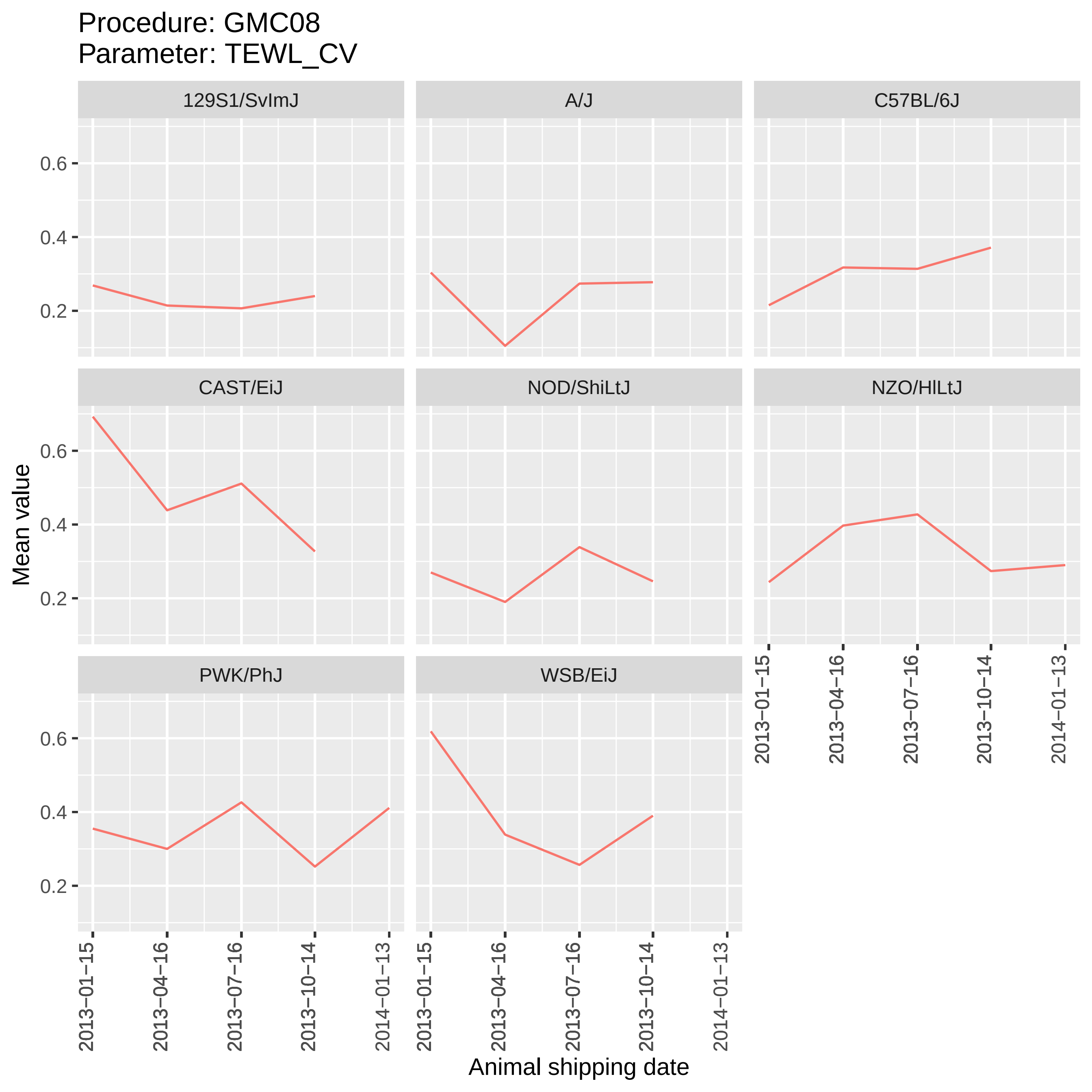

Procedure: GMC08
Parameter: TEWL_CV
129S1/SvImJ
A/J
C57BL/6J
CAST/EiJ
NOD/ShiLtJ
NZO/HlLtJ
PWK/PhJ
WSB/EiJ
Mean value
0.2
0.4
0.6
2013−01−15
2013−04−16
2013−07−16
2013−10−14
2014−01−13
Animal shipping date

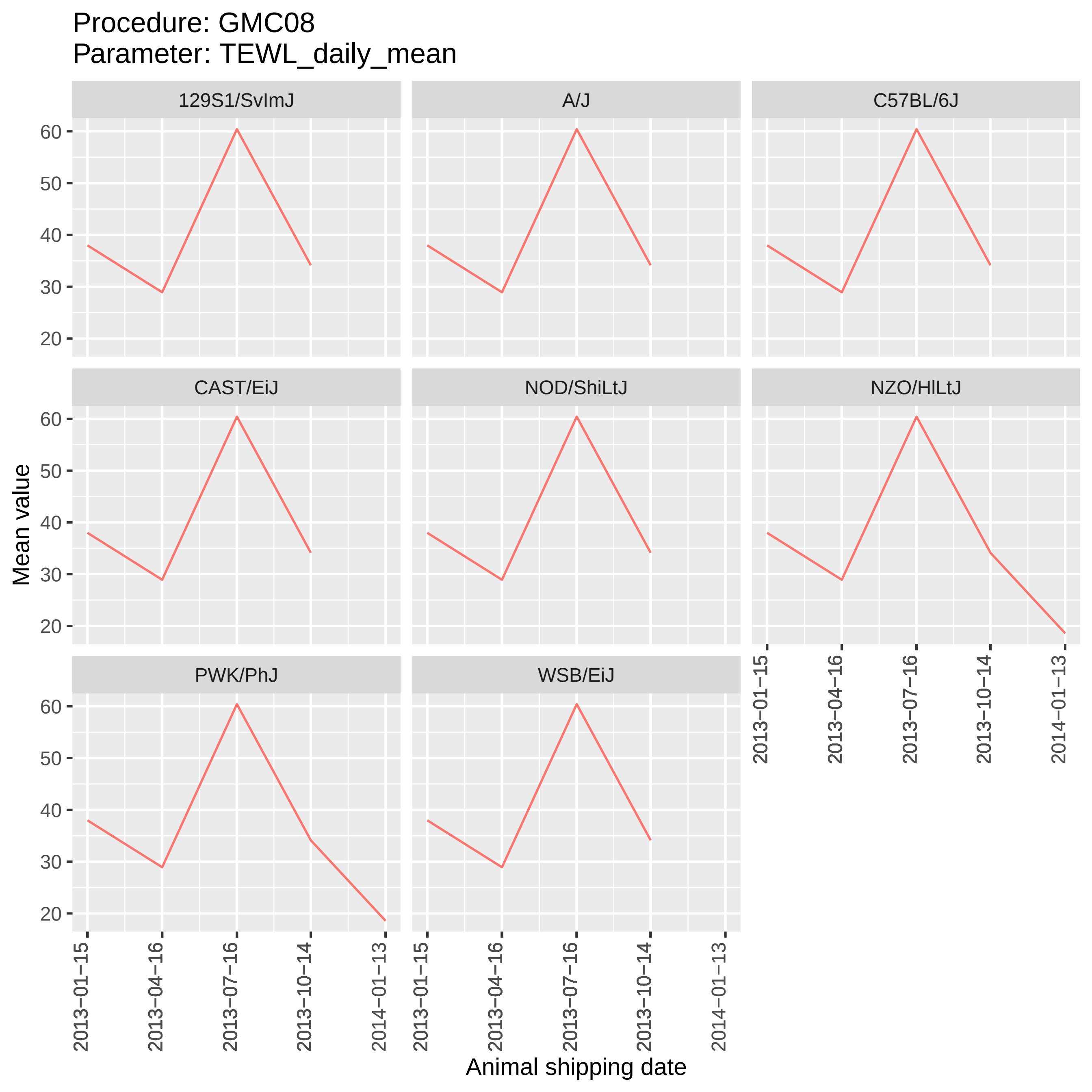

Procedure: GMC08
Parameter: TEWL_daily_mean
129S1/SvImJ
A/J
C57BL/6J
CAST/EiJ
NOD/ShiLtJ
NZO/HlLtJ
PWK/PhJ
WSB/EiJ
Mean value
20
30
40
50
60
2013−01−15
2013−04−16
2013−07−16
2013−10−14
2014−01−13
Animal shipping date

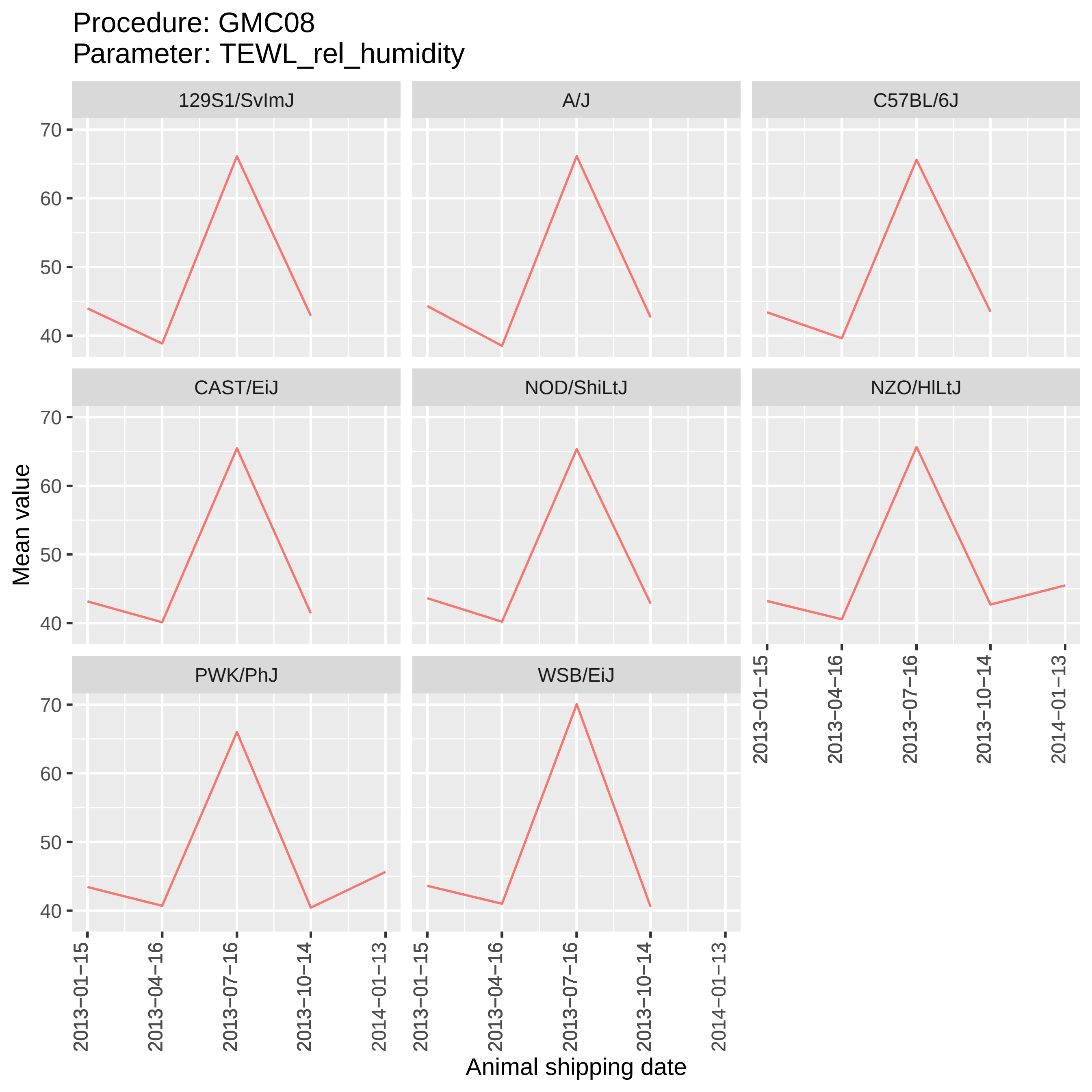

Procedure: GMC08
Parameter: TEWL_rel_humidity
129S1/SvImJ
A/J
C57BL/6J
CAST/EiJ
NOD/ShiLtJ
NZO/HlLtJ
PWK/PhJ
WSB/EiJ
Mean value
70
60
50
40
2013−01−15
2013−04−16
2013−07−16
2013−10−14
2014−01−13
Animal shipping date

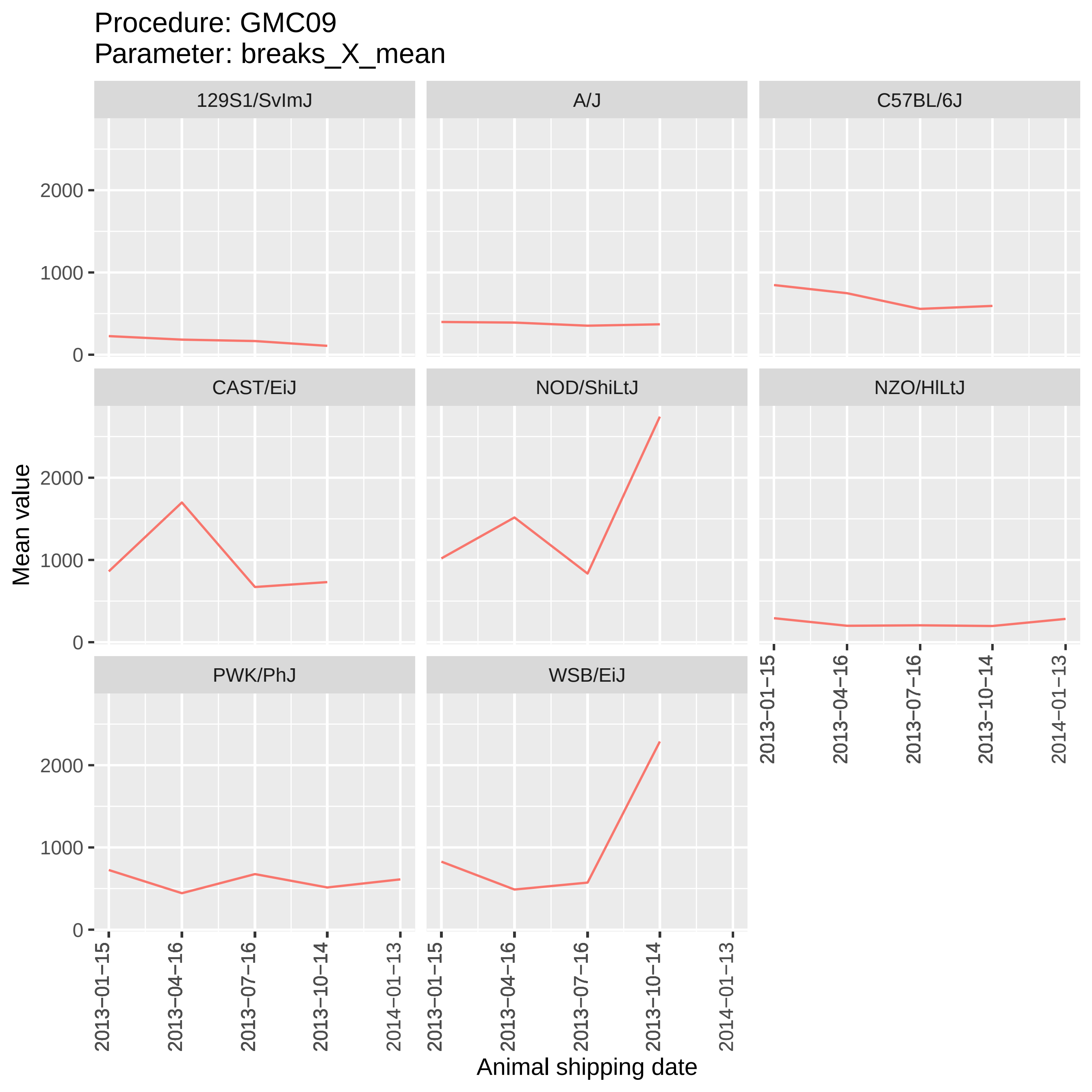

Procedure: GMC09
Parameter: breaks_X_mean
129S1/SvImJ
A/J
C57BL/6J
CAST/EiJ
NOD/ShiLtJ
NZO/HlLtJ
PWK/PhJ
WSB/EiJ
Mean value
0
1000
2000
2013−01−15
2013−04−16
2013−07−16
2013−10−14
2014−01−13
Animal shipping date

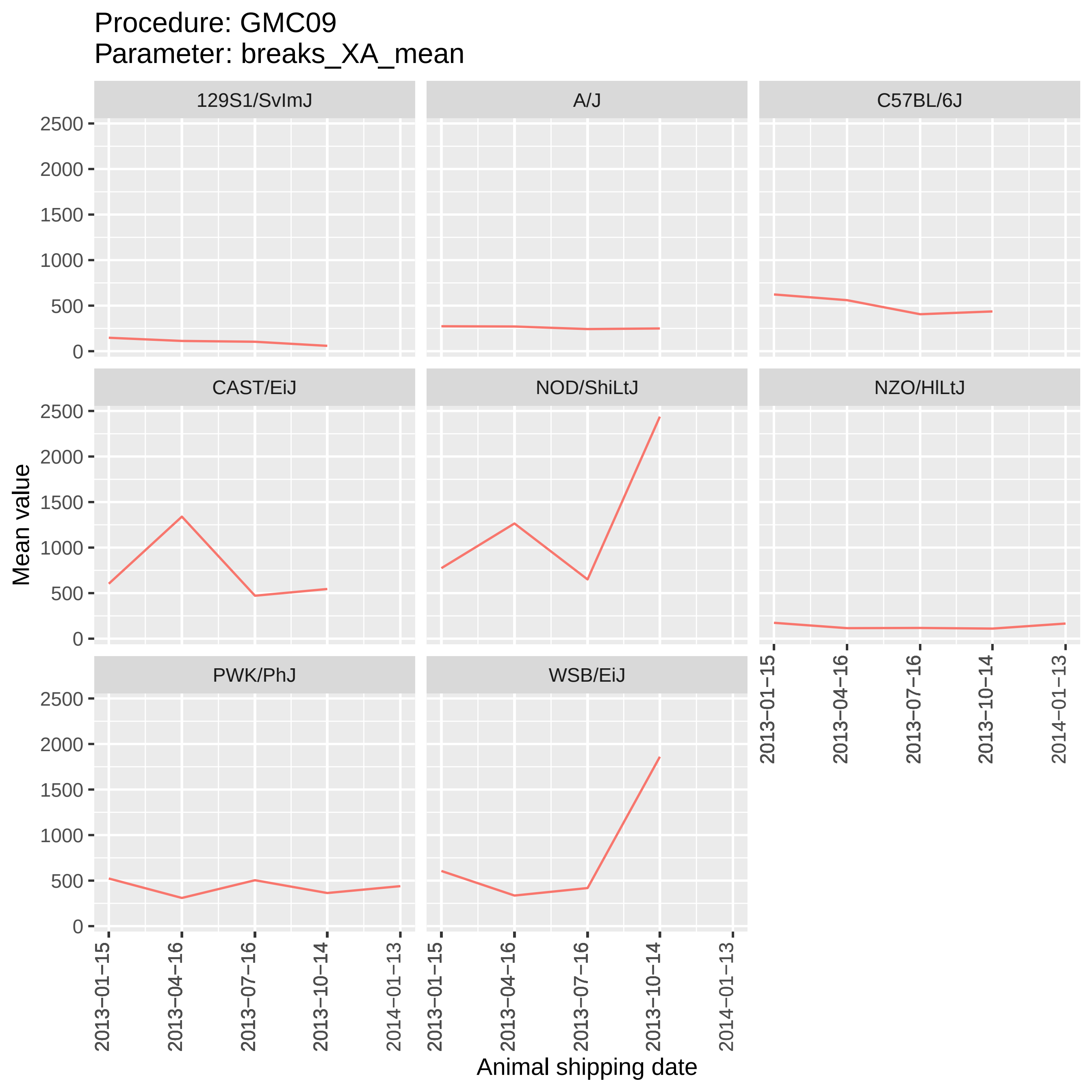

Procedure: GMC09
Parameter: breaks_XA_mean
129S1/SvImJ
A/J
C57BL/6J
CAST/EiJ
NOD/ShiLtJ
NZO/HlLtJ
PWK/PhJ
WSB/EiJ
0
500
1000
1500
2000
2500
2013−01−15
2013−04−16
2013−07−16
2013−10−14
2014−01−13
Mean value
Animal shipping date

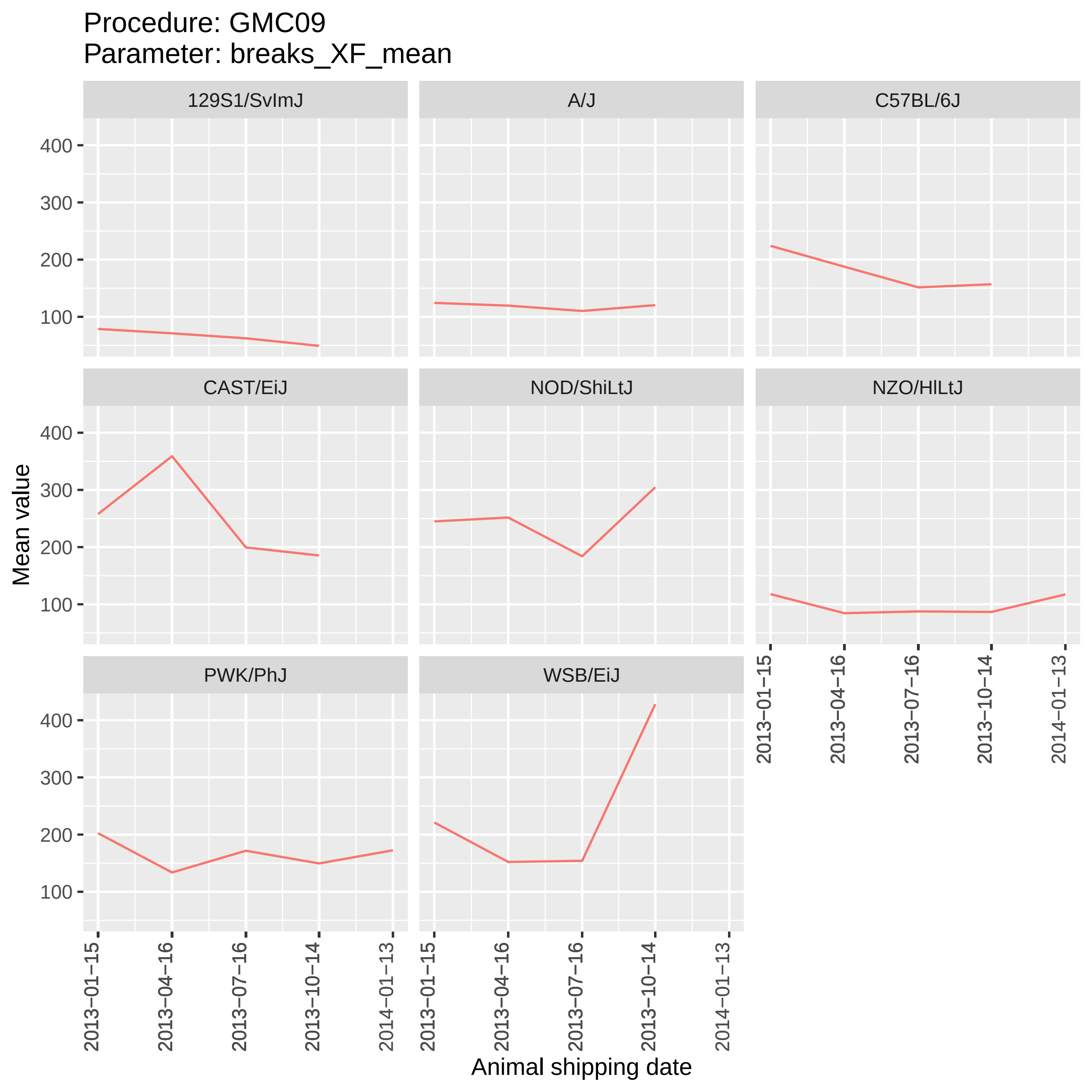
Procedure: GMC09
Parameter: breaks_XF_mean
129S1/SvImJ
A/J
C57BL/6J
CAST/EiJ
NOD/ShiLtJ
NZO/HlLtJ
PWK/PhJ
WSB/EiJ
Mean value
100
200
300
400
2013−01−15
2013−04−16
2013−07−16
2013−10−14
2014−01−13
Animal shipping date

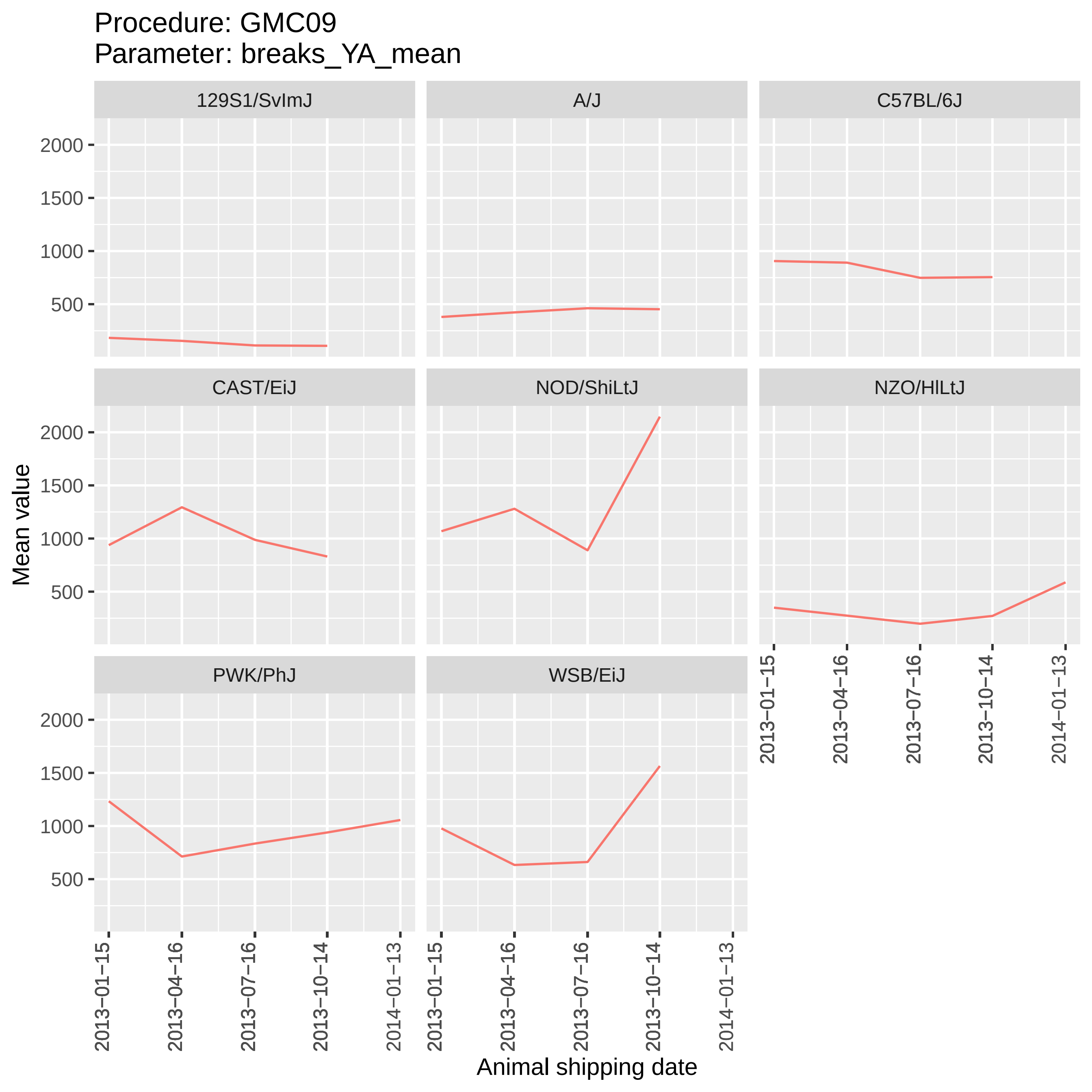
Procedure: GMC09
Parameter: breaks_YA_mean
129S1/SvImJ
A/J
C57BL/6J
CAST/EiJ
NOD/ShiLtJ
NZO/HlLtJ
PWK/PhJ
WSB/EiJ
Mean value
500
1000
1500
2000
2013−01−15
2013−04−16
2013−07−16
2013−10−14
2014−01−13
Animal shipping date

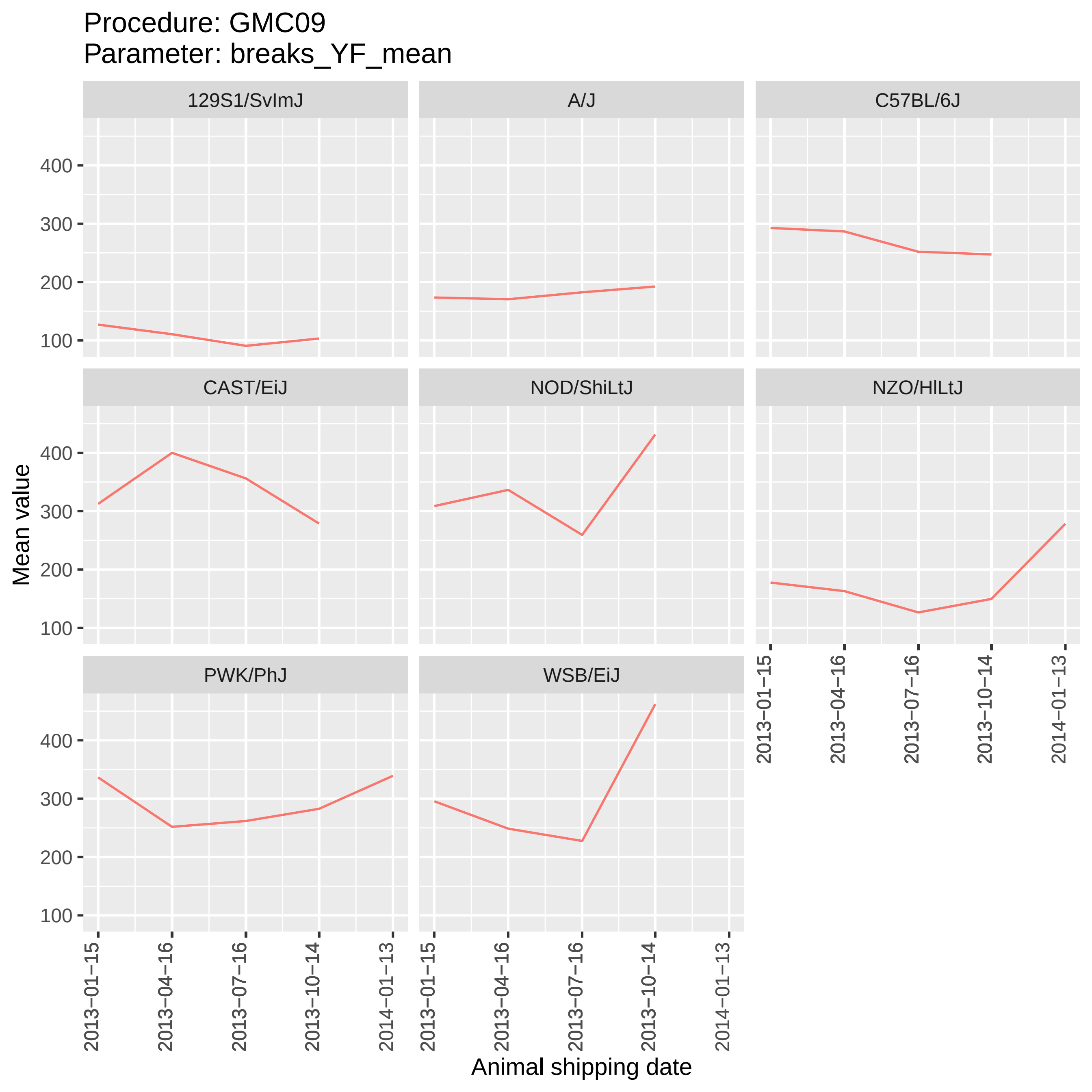
Procedure: GMC09
Parameter: breaks_YF_mean
129S1/SvImJ
A/J
C57BL/6J
CAST/EiJ
NOD/ShiLtJ
NZO/HlLtJ
PWK/PhJ
WSB/EiJ
Mean value
100
200
300
400
2013−01−15
2013−04−16
2013−07−16
2013−10−14
2014−01−13
Animal shipping date

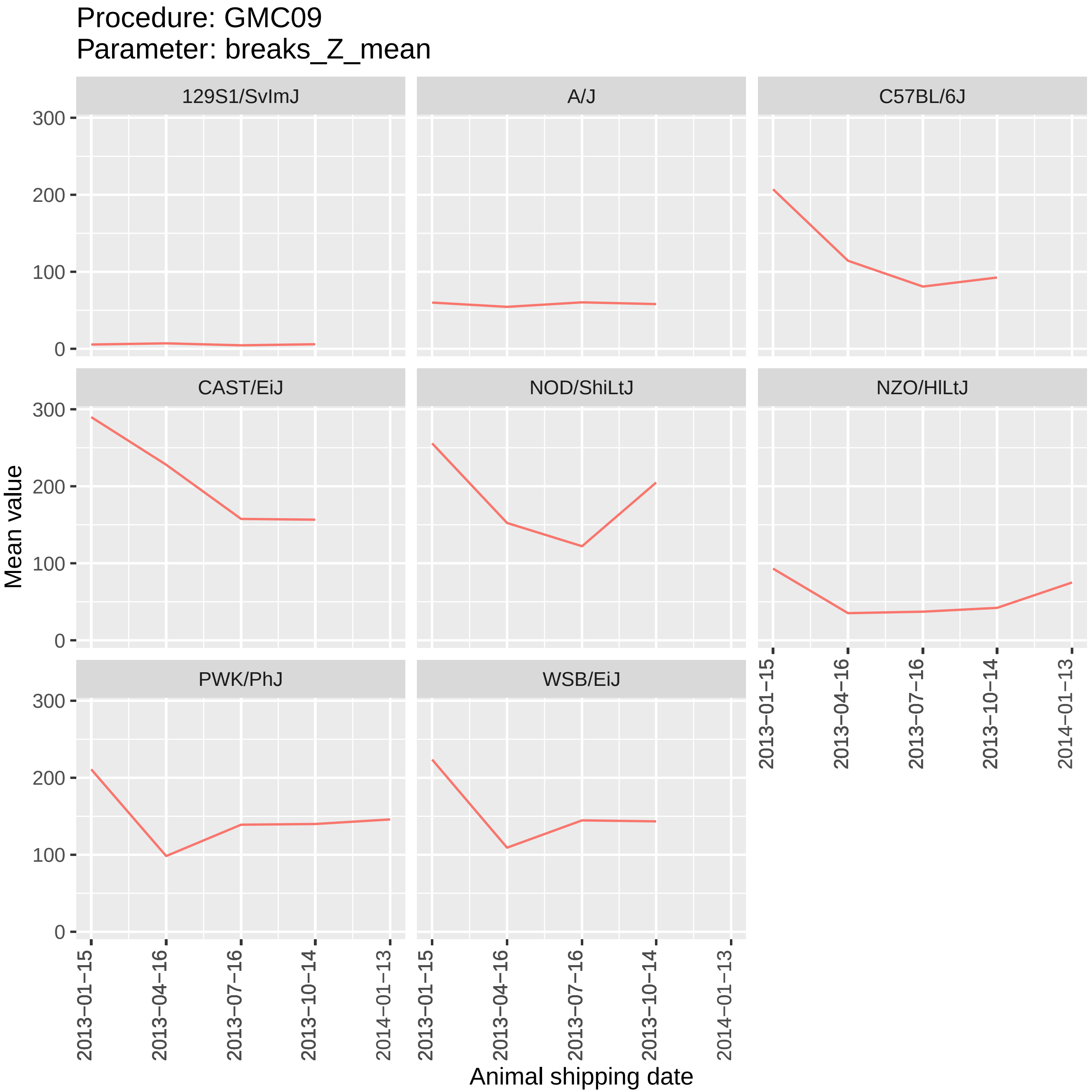

Procedure: GMC09
Parameter: breaks_Z_mean
129S1/SvImJ
A/J
C57BL/6J
CAST/EiJ
NOD/ShiLtJ
NZO/HlLtJ
PWK/PhJ
WSB/EiJ
Mean value
Animal shipping date
0
100
200
300
2013−01−15
2013−04−16
2013−07−16
2013−10−14
2014−01−13

Procedure: GMC09
Parameter: bw_after

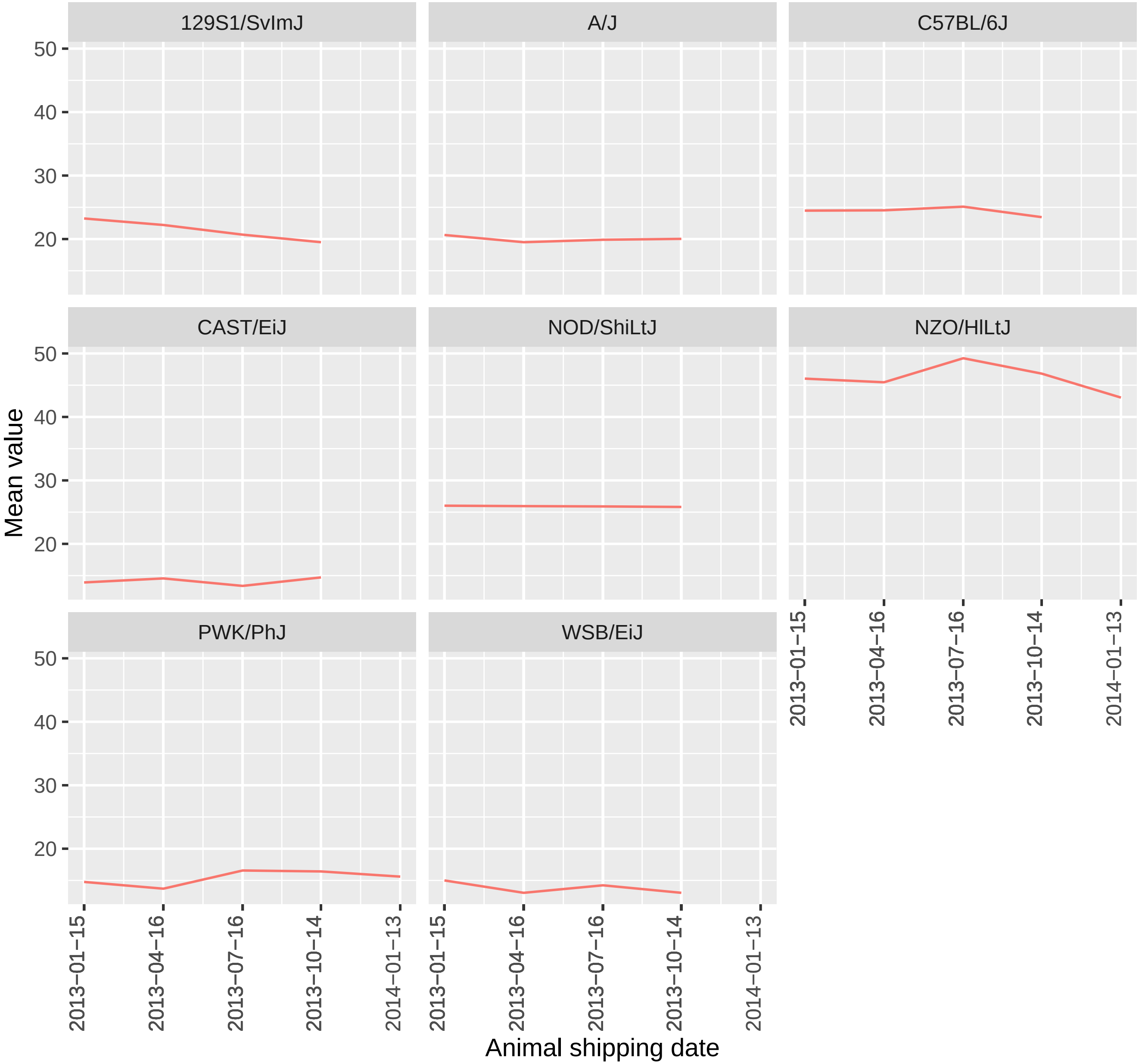

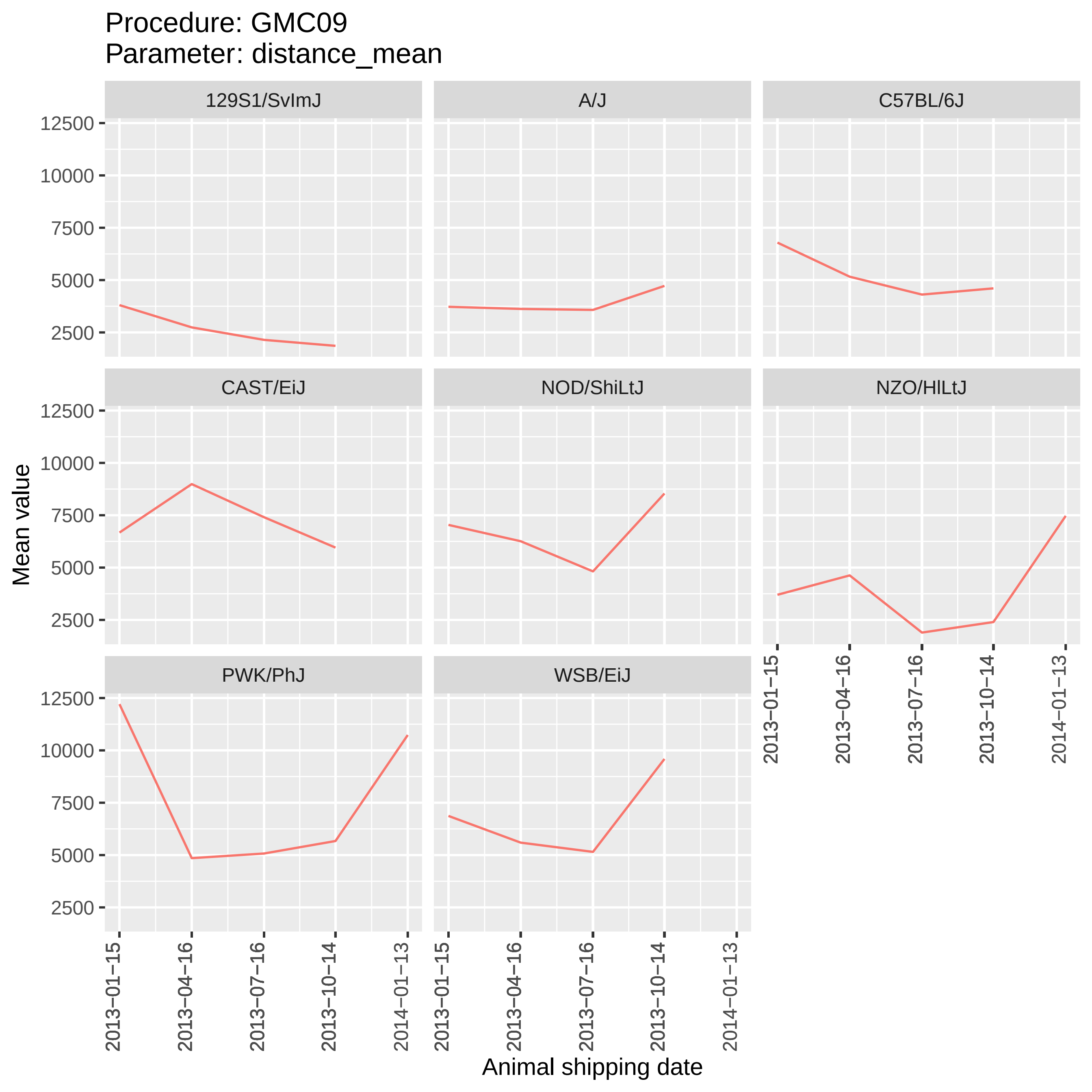

Procedure: GMC09
Parameter: distance_mean
129S1/SvImJ
A/J
C57BL/6J
CAST/EiJ
NOD/ShiLtJ
NZO/HlLtJ
PWK/PhJ
WSB/EiJ
12500
10000
7500
5000
2500
Mean value
2013−01−15
2013−04−16
2013−07−16
2013−10−14
2014−01−13
Animal shipping date

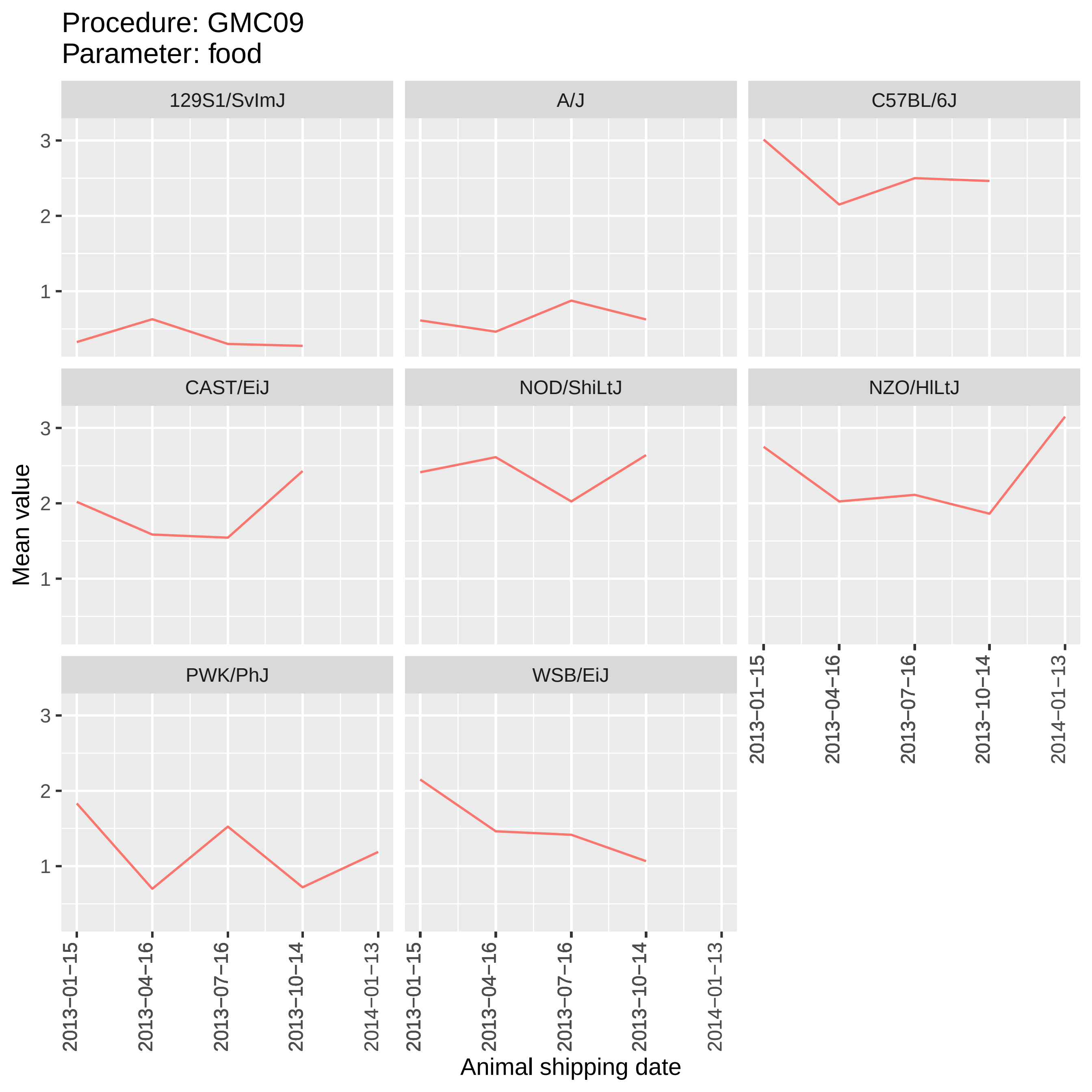
Procedure: GMC09
Parameter: food
129S1/SvImJ
A/J
C57BL/6J
CAST/EiJ
NOD/ShiLtJ
NZO/HlLtJ
PWK/PhJ
WSB/EiJ
Mean value
1
2
3
2013−01−15
2013−04−16
2013−07−16
2013−10−14
2014−01−13
Animal shipping date

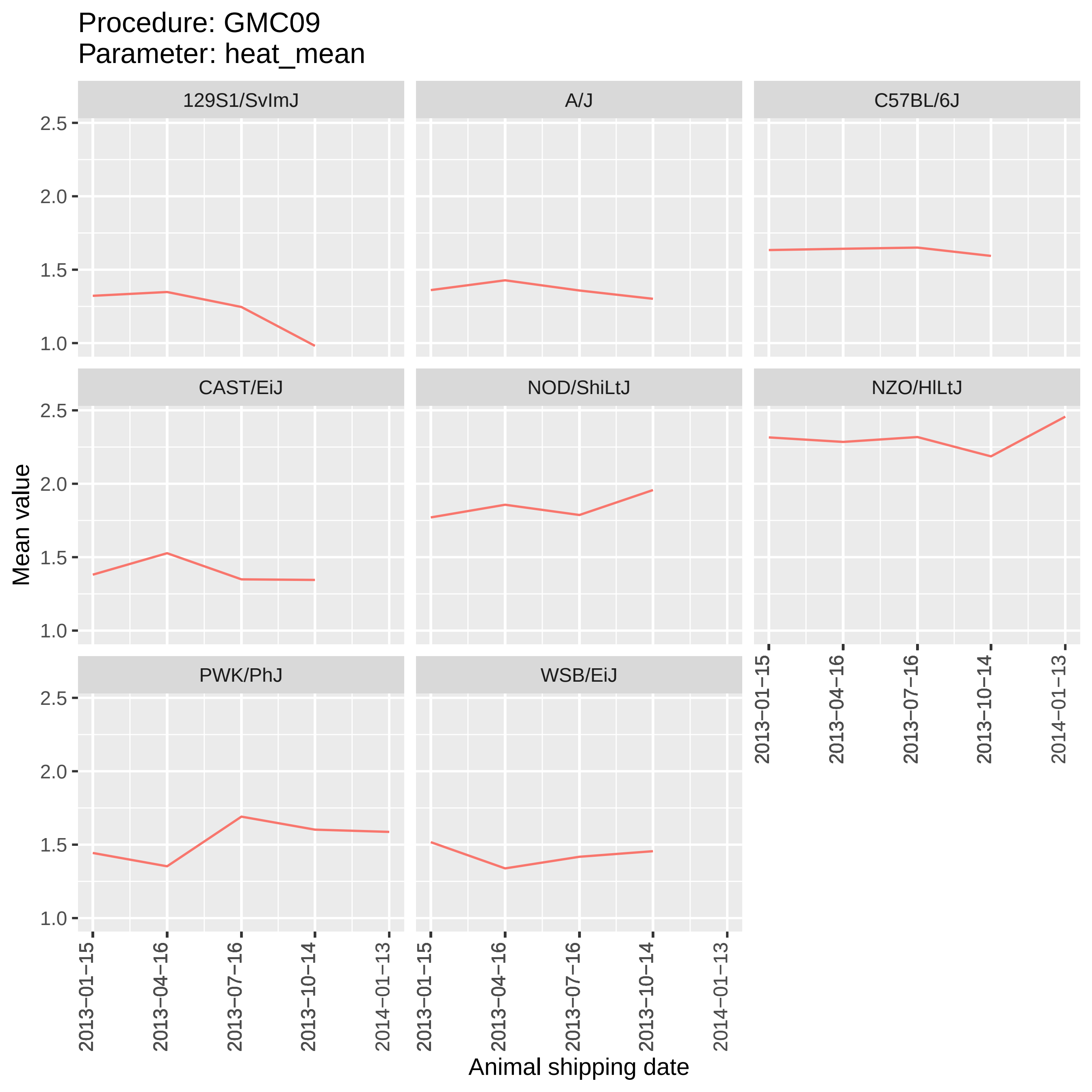

Procedure: GMC09
Parameter: heat_mean
129S1/SvImJ
A/J
C57BL/6J
CAST/EiJ
NOD/ShiLtJ
NZO/HlLtJ
PWK/PhJ
WSB/EiJ
2.5
2.0
1.5
1.0
Mean value
2013−01−15
2013−04−16
2013−07−16
2013−10−14
2014−01−13
Animal shipping date

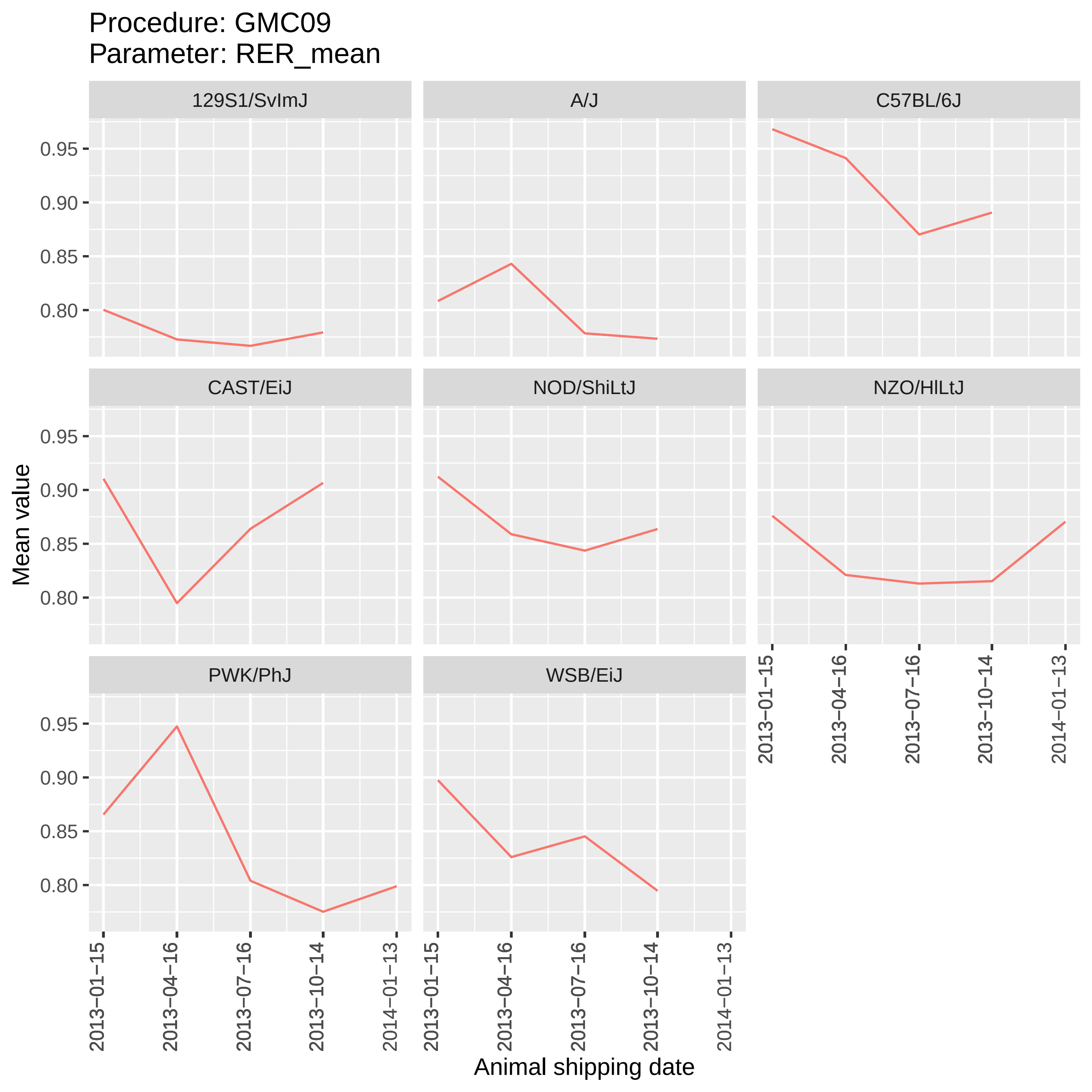

Procedure: GMC09
Parameter: RER_mean
129S1/SvImJ
A/J
C57BL/6J
CAST/EiJ
NOD/ShiLtJ
NZO/HlLtJ
PWK/PhJ
WSB/EiJ
0.95
0.90
0.85
0.80
Mean value
2013−01−15
2013−04−16
2013−07−16
2013−10−14
2014−01−13
Animal shipping date

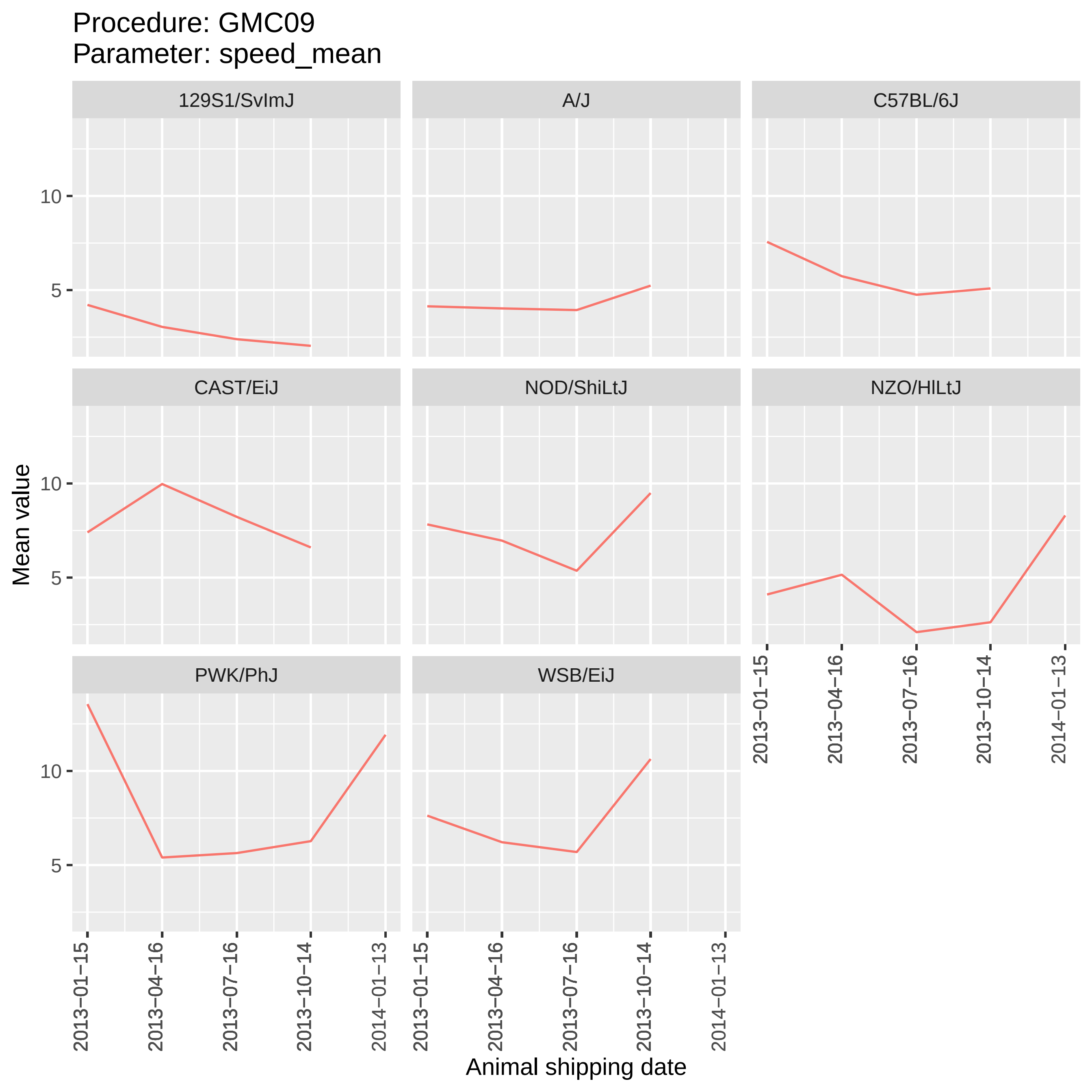

Procedure: GMC09
Parameter: speed_mean
129S1/SvImJ
A/J
C57BL/6J
CAST/EiJ
NOD/ShiLtJ
NZO/HlLtJ
PWK/PhJ
WSB/EiJ
Mean value
10
5
2013−01−15
2013−04−16
2013−07−16
2013−10−14
2014−01−13
Animal shipping date

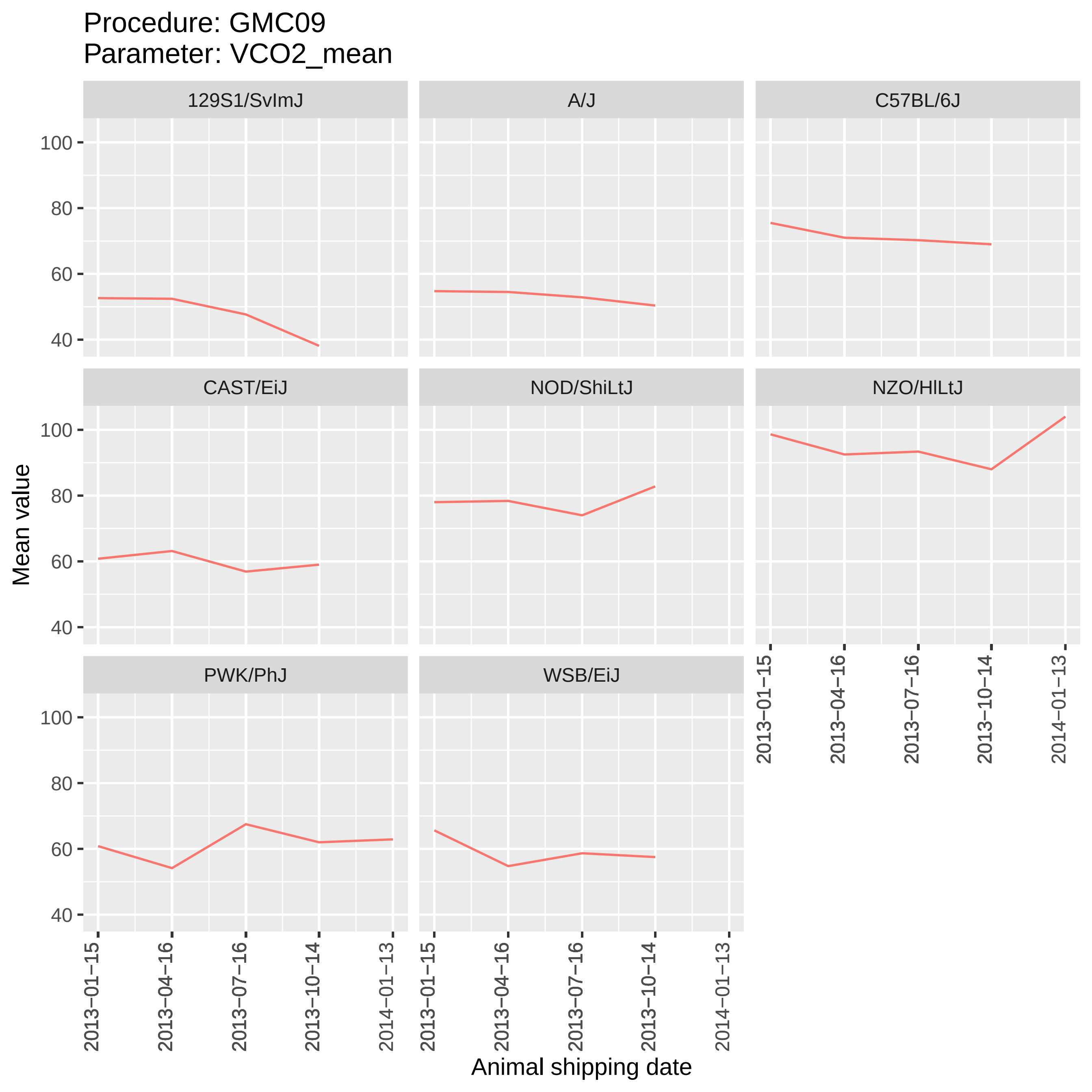
Procedure: GMC09
Parameter: VCO2_mean
129S1/SvImJ
A/J
C57BL/6J
CAST/EiJ
NOD/ShiLtJ
NZO/HlLtJ
PWK/PhJ
WSB/EiJ
Mean value
100
80
60
40
2013−01−15
2013−04−16
2013−07−16
2013−10−14
2014−01−13
Animal shipping date

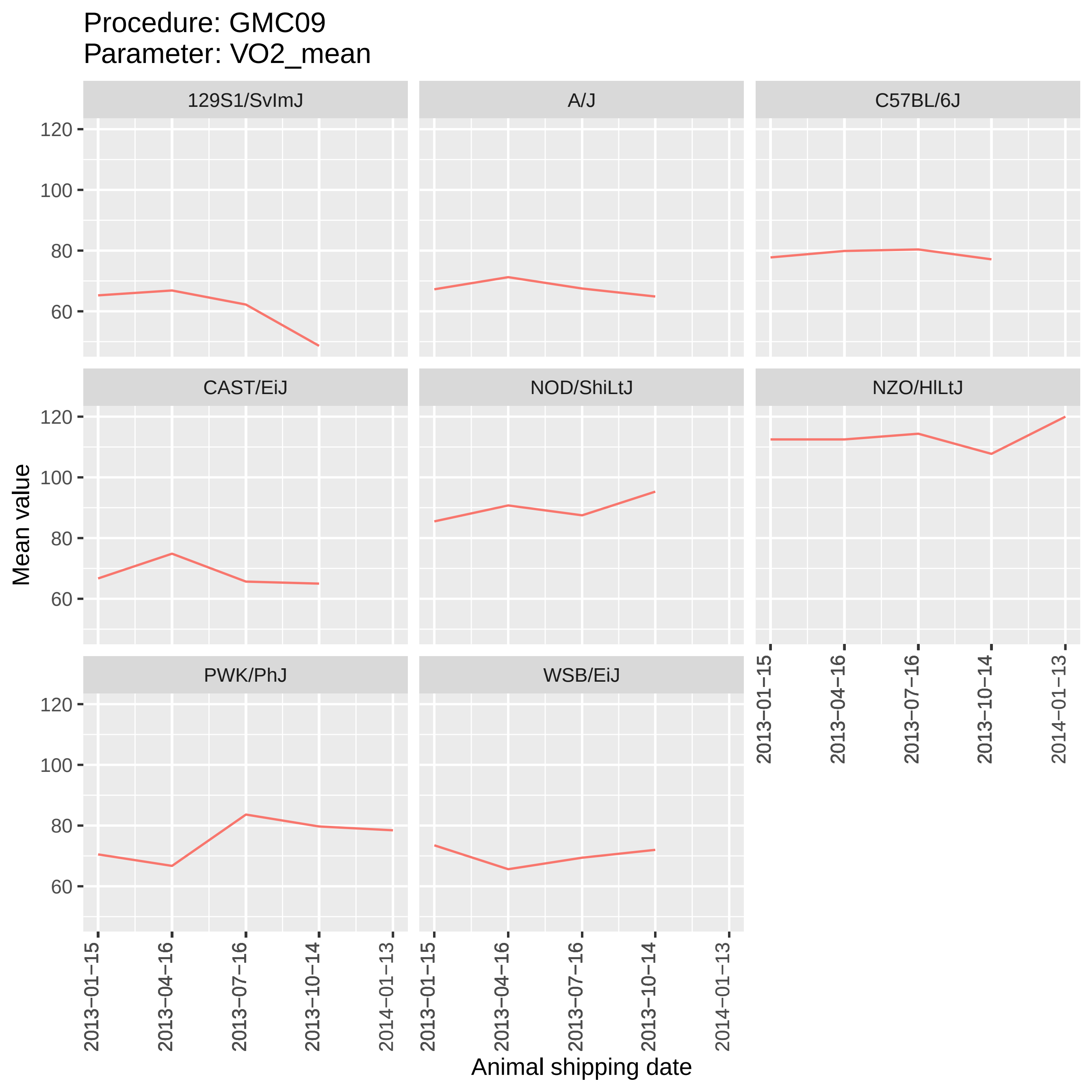

Procedure: GMC09
Parameter: VO2_mean
129S1/SvImJ
A/J
C57BL/6J
CAST/EiJ
NOD/ShiLtJ
NZO/HlLtJ
PWK/PhJ
WSB/EiJ
Mean value
120
100
80
60
2013−01−15
2013−04−16
2013−07−16
2013−10−14
2014−01−13
Animal shipping date

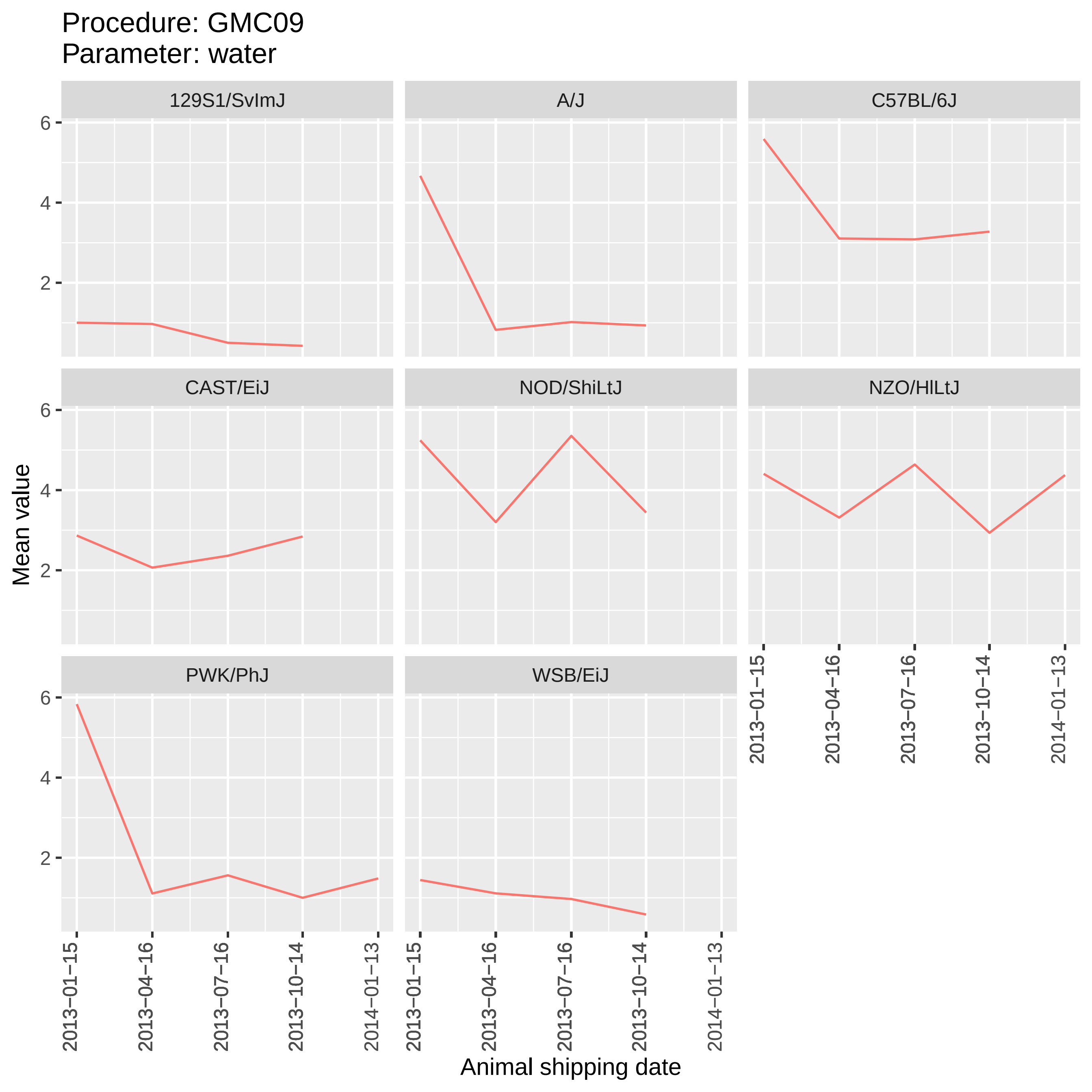

Procedure: GMC09
Parameter: water
129S1/SvImJ
A/J
C57BL/6J
CAST/EiJ
NOD/ShiLtJ
NZO/HlLtJ
PWK/PhJ
WSB/EiJ
Mean value
6
4
2
2013−01−15
2013−04−16
2013−07−16
2013−10−14
2014−01−13
Animal shipping date

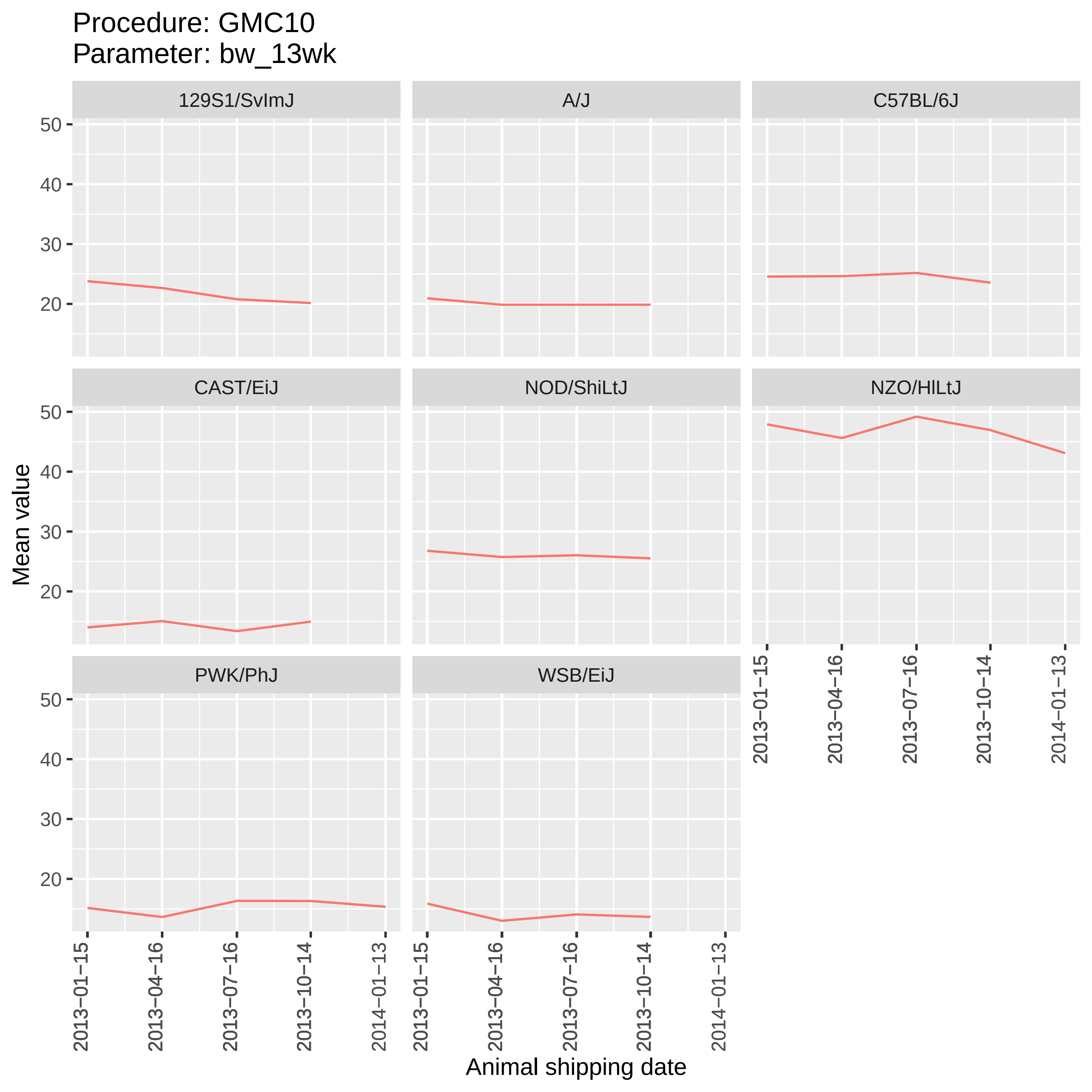
Procedure: GMC10
Parameter: bw_13wk
129S1/SvImJ
A/J
C57BL/6J
CAST/EiJ
NOD/ShiLtJ
NZO/HlLtJ
PWK/PhJ
WSB/EiJ
Mean value
50
40
30
20
2013−01−15
2013−04−16
2013−07−16
2013−10−14
2014−01−13
Animal shipping date

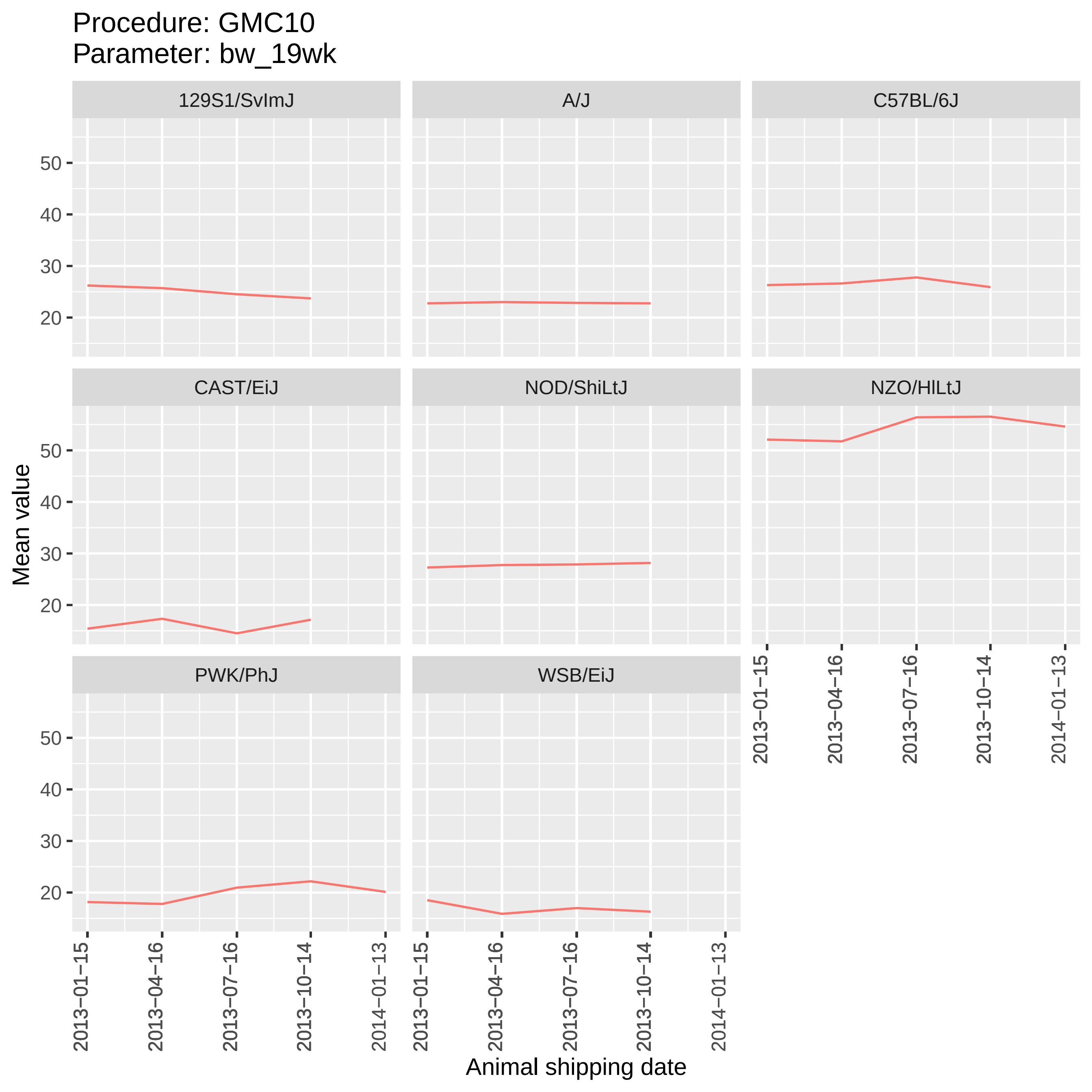

Procedure: GMC10
Parameter: bw_19wk
129S1/SvImJ
A/J
C57BL/6J
CAST/EiJ
NOD/ShiLtJ
NZO/HlLtJ
PWK/PhJ
WSB/EiJ
Mean value
20
30
40
50
2013−01−15
2013−04−16
2013−07−16
2013−10−14
2014−01−13
Animal shipping date

Procedure: GMC10
Parameter: fat_13wk

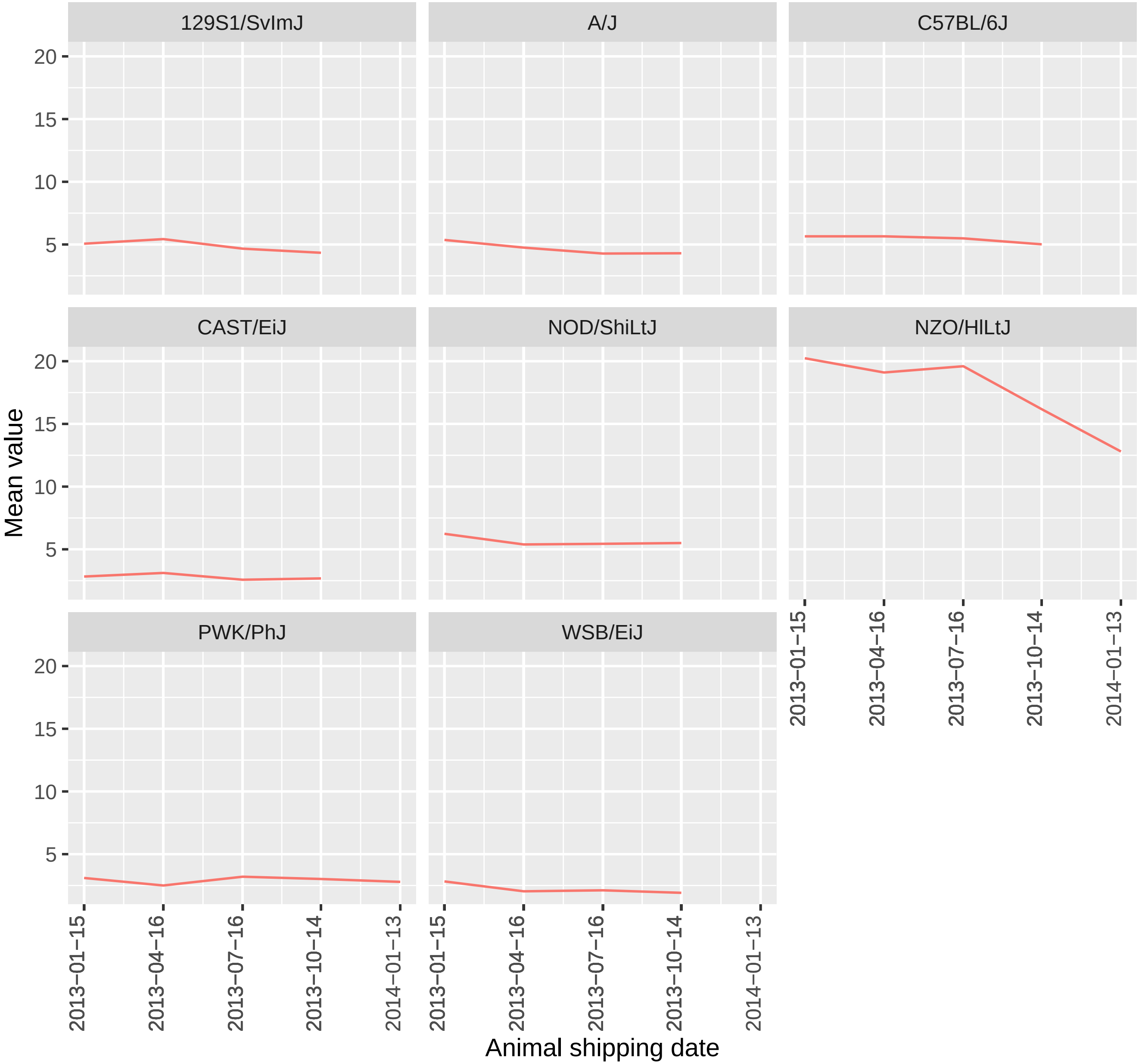

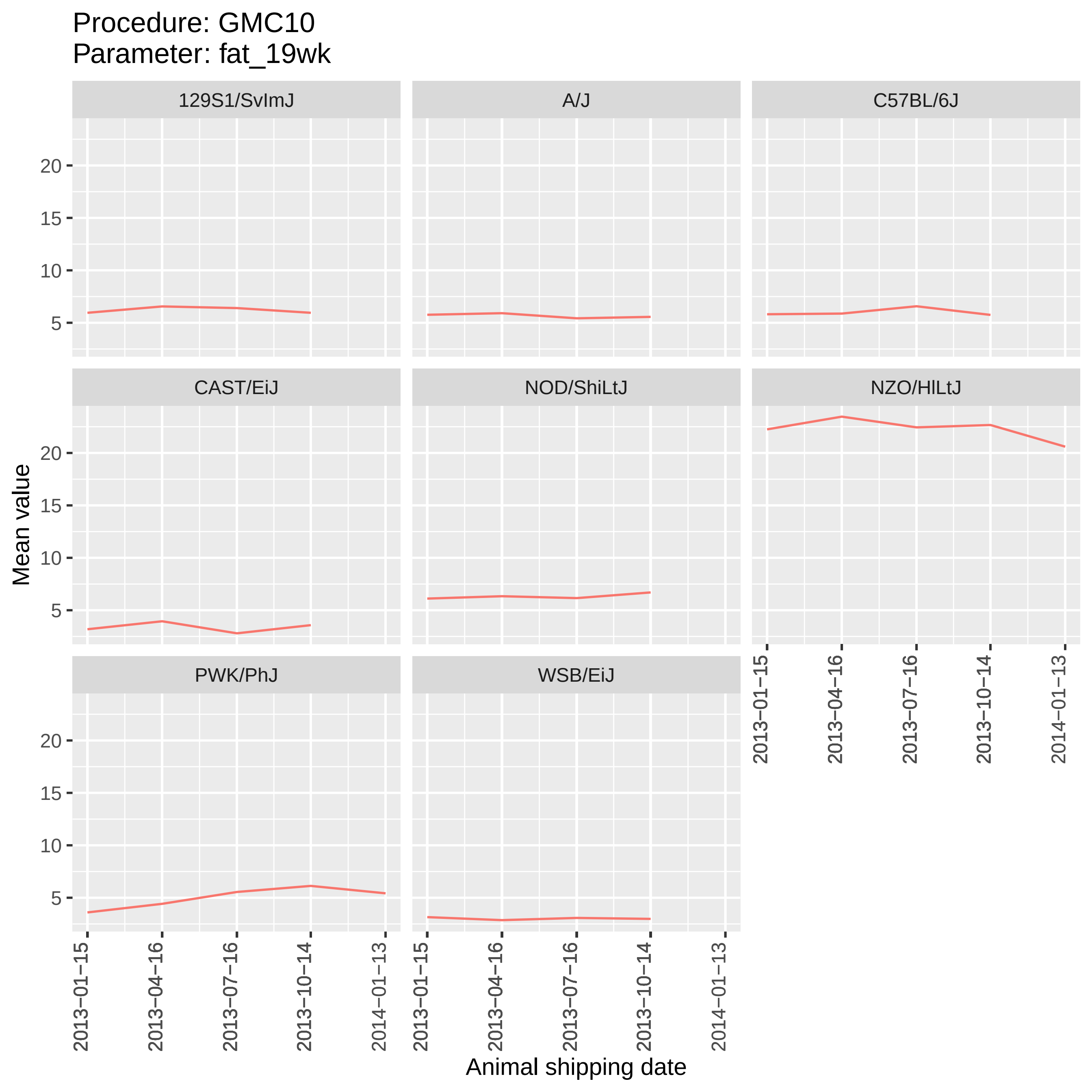

Procedure: GMC10
Parameter: fat_19wk
129S1/SvImJ
A/J
C57BL/6J
CAST/EiJ
NOD/ShiLtJ
NZO/HlLtJ
PWK/PhJ
WSB/EiJ
Mean value
20
15
10
5
2013−01−15
2013−04−16
2013−07−16
2013−10−14
2014−01−13
Animal shipping date

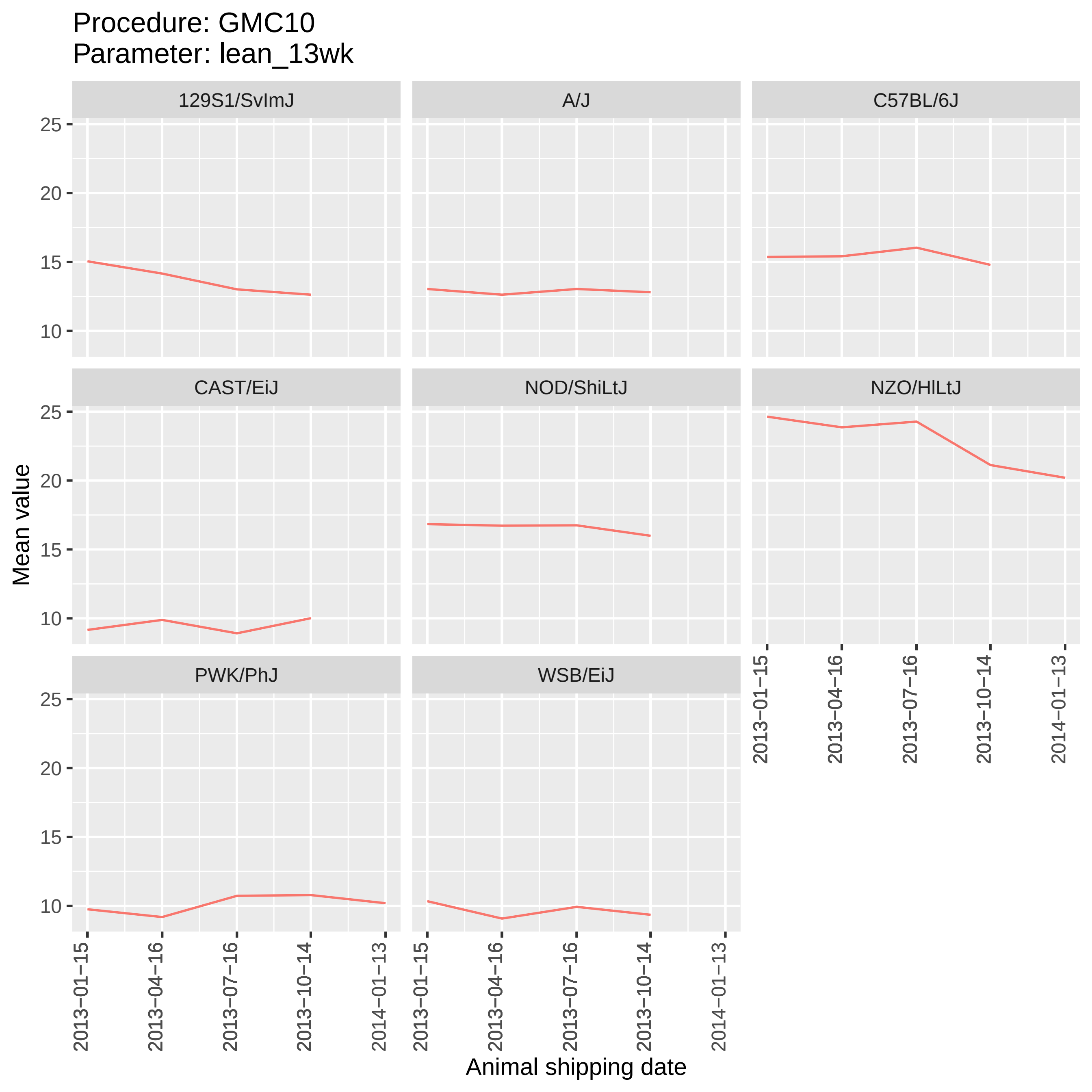
Procedure: GMC10
Parameter: lean_13wk
129S1/SvImJ
A/J
C57BL/6J
CAST/EiJ
NOD/ShiLtJ
NZO/HlLtJ
PWK/PhJ
WSB/EiJ
Mean value
10
15
20
25
2013−01−15
2013−04−16
2013−07−16
2013−10−14
2014−01−13
Animal shipping date

Procedure: GMC10
Parameter: lean_19wk

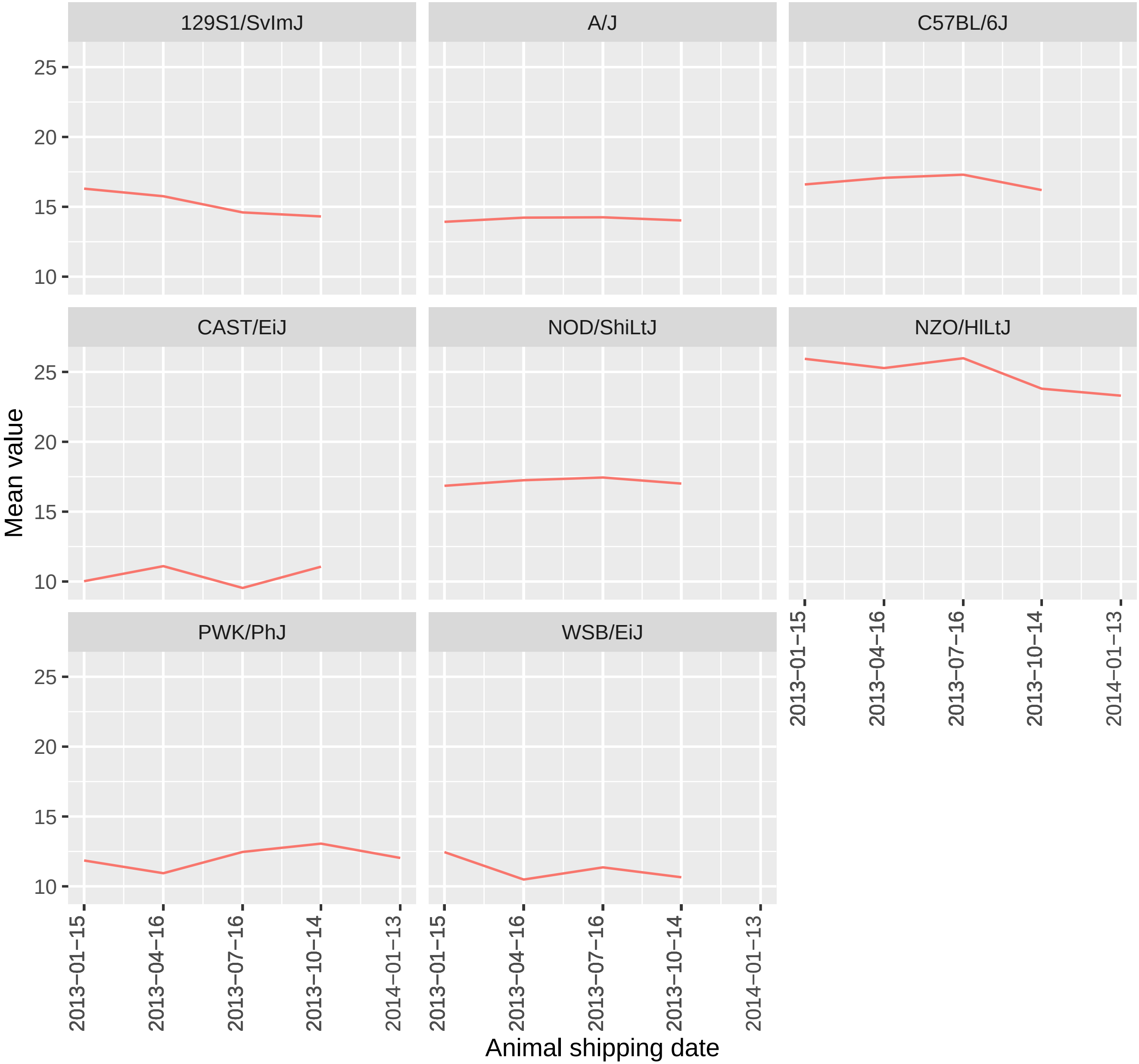

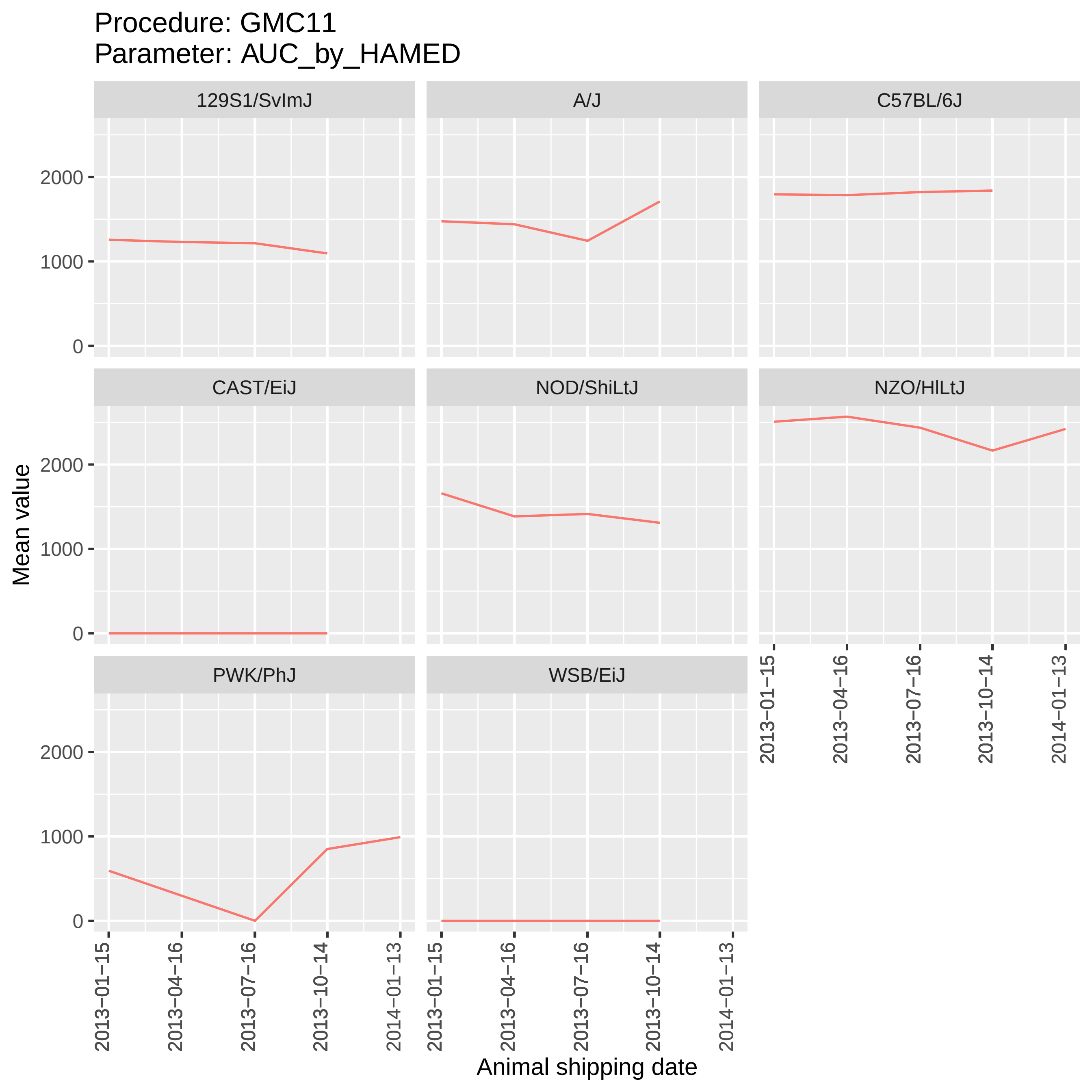

Procedure: GMC11
Parameter: AUC_by_HAMED
129S1/SvImJ
A/J
C57BL/6J
CAST/EiJ
NOD/ShiLtJ
NZO/HlLtJ
PWK/PhJ
WSB/EiJ
Mean value
0
1000
2000
2013−01−15
2013−04−16
2013−07−16
2013−10−14
2014−01−13
Animal shipping date

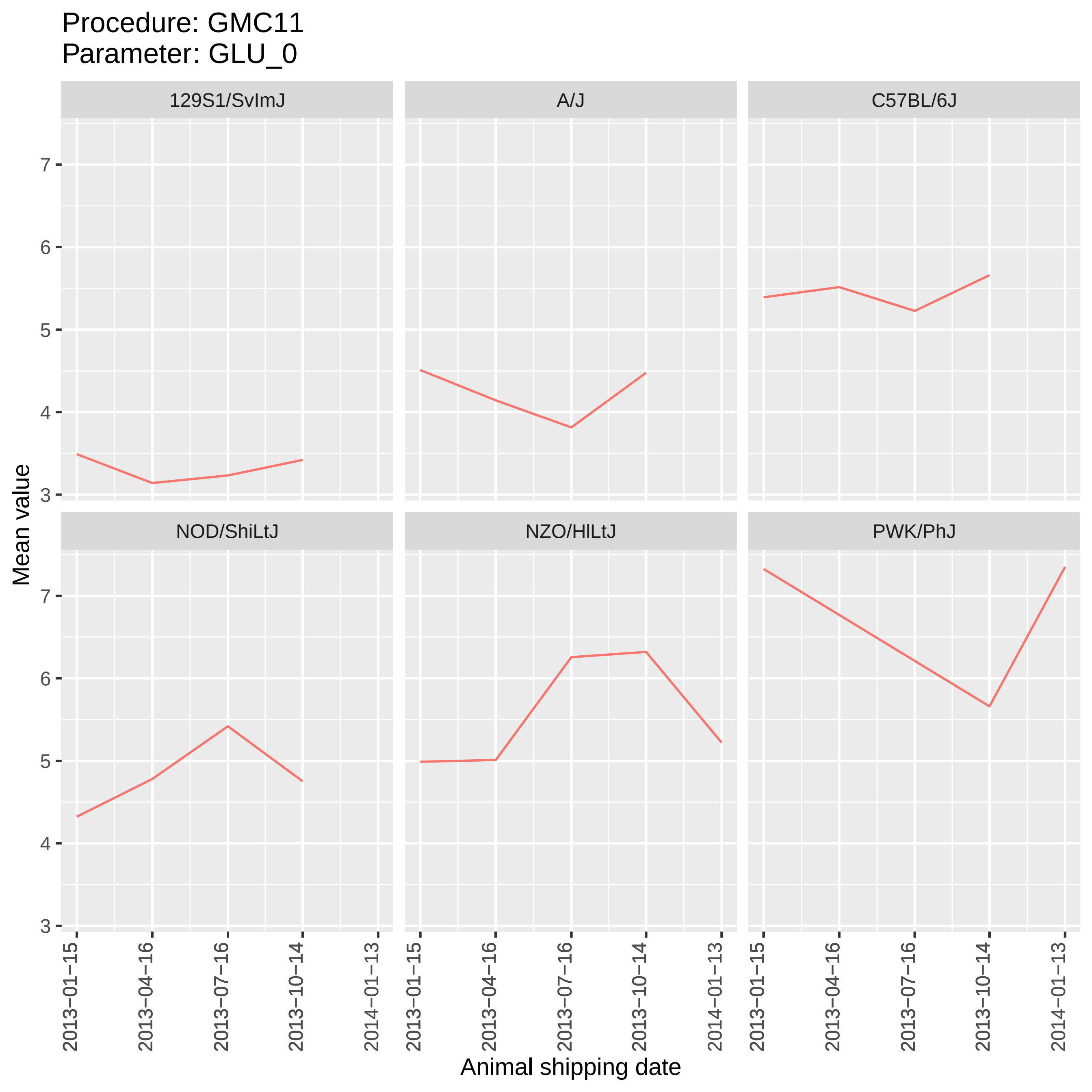
Procedure: GMC11
Parameter: GLU_0
129S1/SvImJ
A/J
C57BL/6J
NOD/ShiLtJ
NZO/HlLtJ
PWK/PhJ
Mean value
3
4
5
6
7
2013−01−15
2013−04−16
2013−07−16
2013−10−14
2014−01−13
Animal shipping date

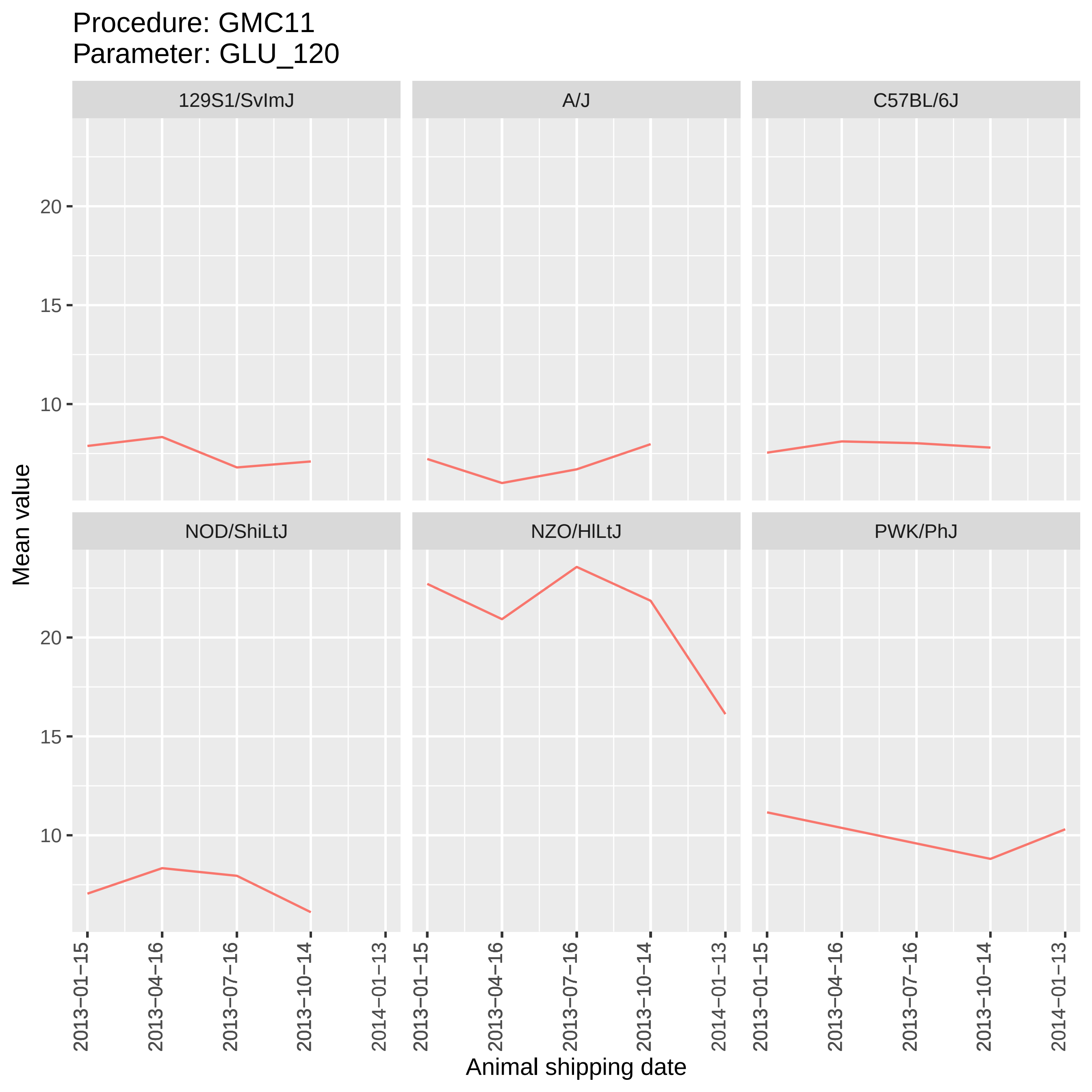

Procedure: GMC11
Parameter: GLU_120
129S1/SvImJ
A/J
C57BL/6J
NOD/ShiLtJ
NZO/HlLtJ
PWK/PhJ
Mean value
20
15
10
2013−01−15
2013−04−16
2013−07−16
2013−10−14
2014−01−13
Animal shipping date

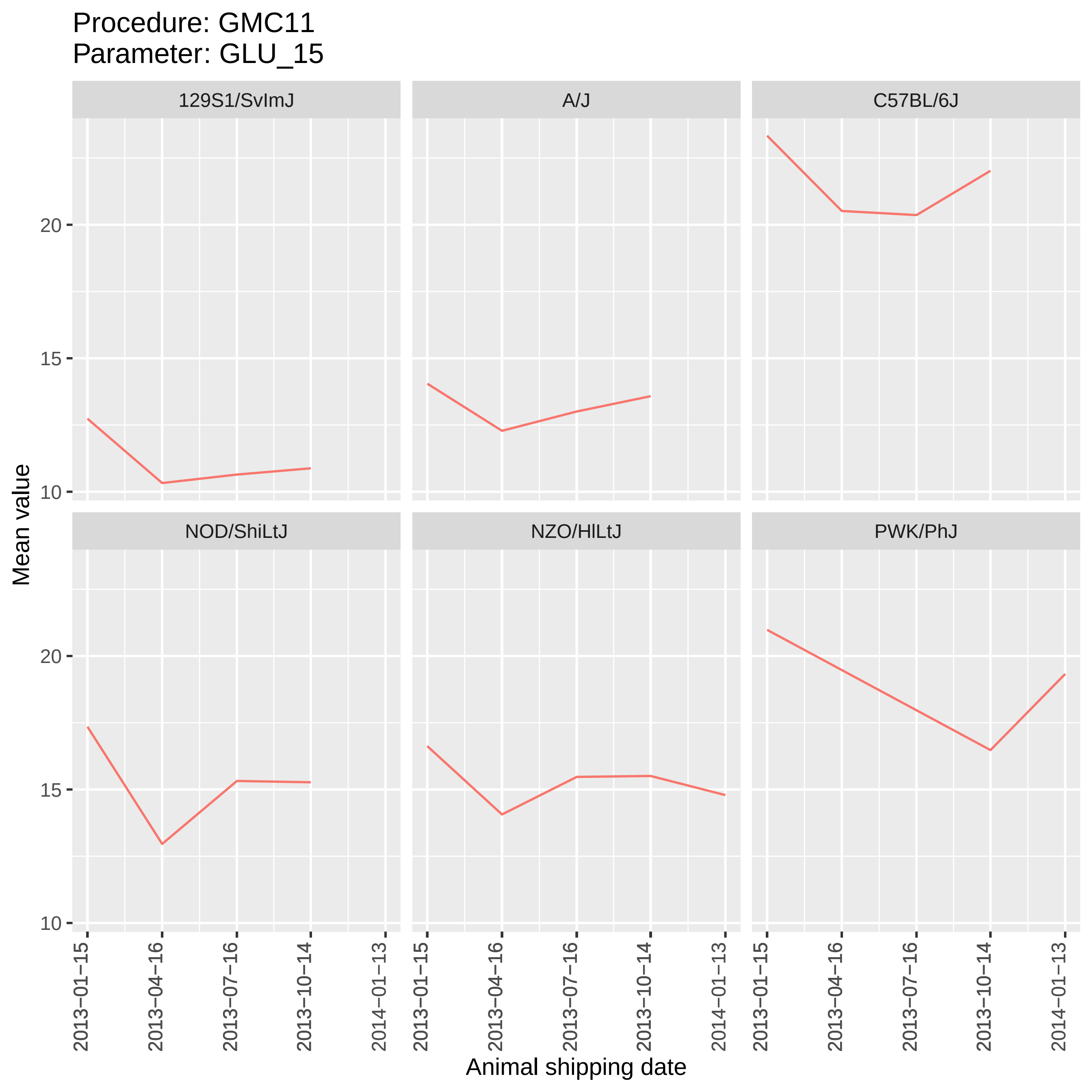

Procedure: GMC11
Parameter: GLU_15
129S1/SvImJ
A/J
C57BL/6J
NOD/ShiLtJ
NZO/HlLtJ
PWK/PhJ
Mean value
10
15
20
2013−01−15
2013−04−16
2013−07−16
2013−10−14
2014−01−13
Animal shipping date

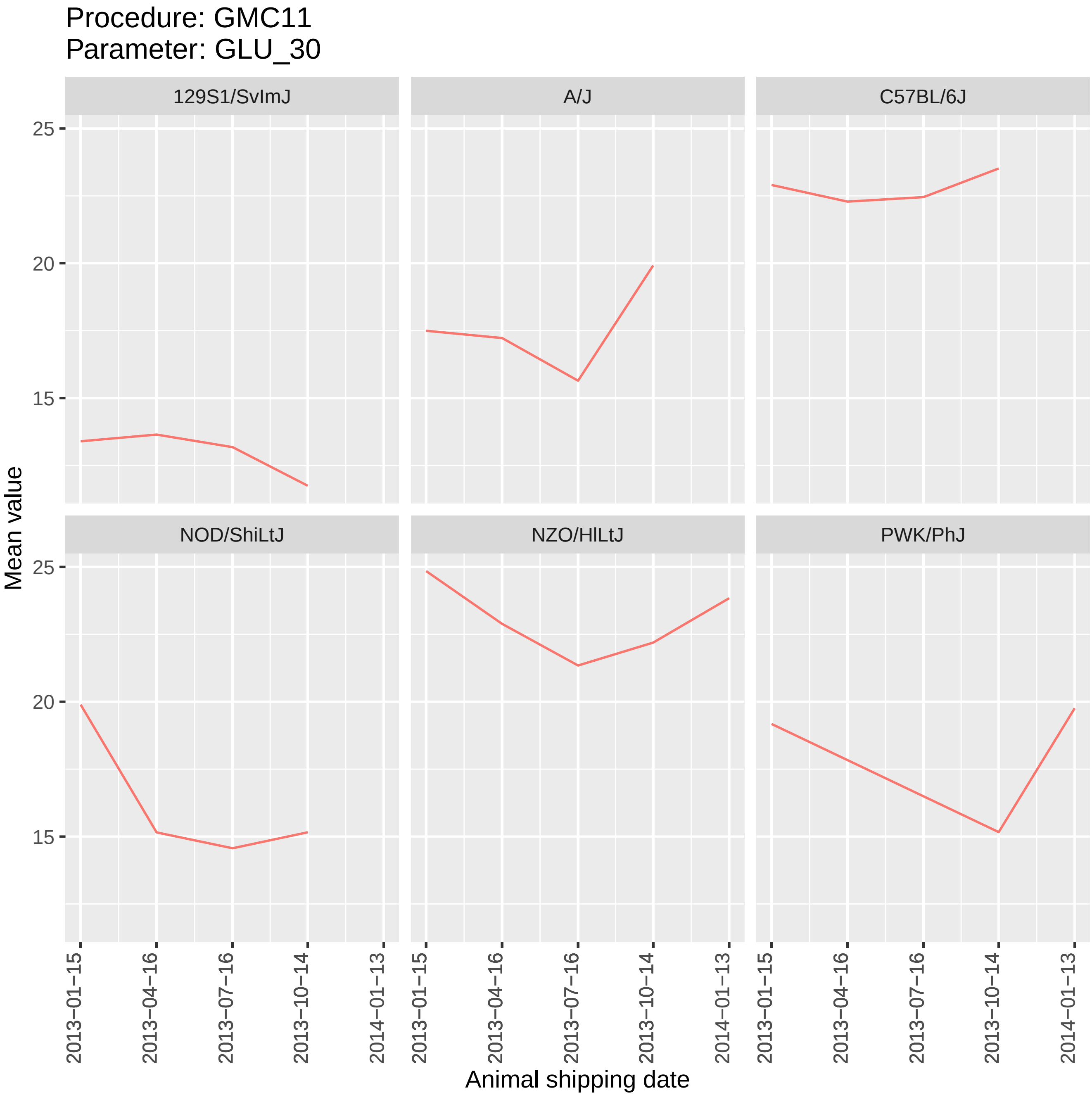

Procedure: GMC11
Parameter: GLU_30
129S1/SvImJ
A/J
C57BL/6J
NOD/ShiLtJ
NZO/HlLtJ
PWK/PhJ
Mean value
25
20
15
2013−01−15
2013−04−16
2013−07−16
2013−10−14
2014−01−13
Animal shipping date

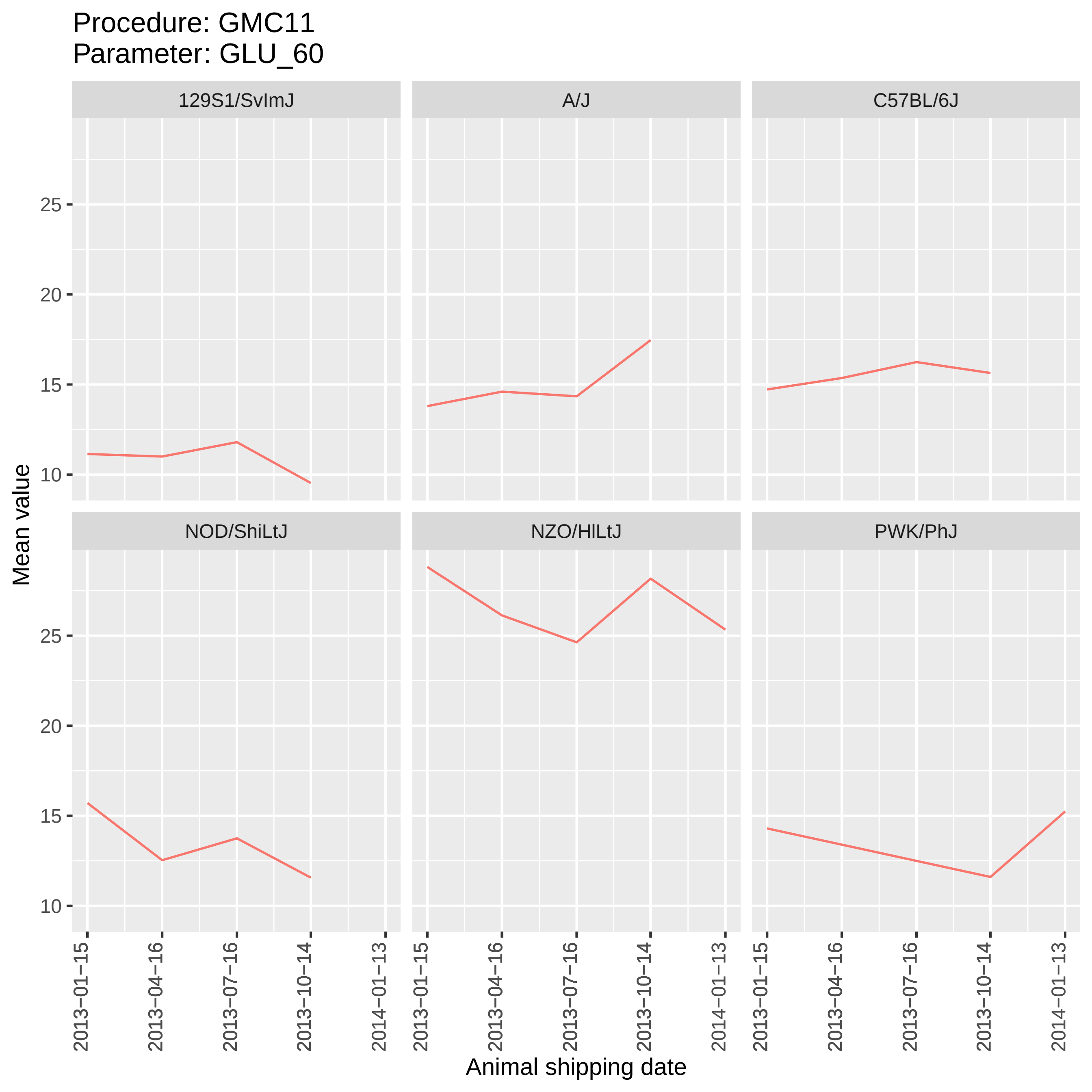

Procedure: GMC11
Parameter: GLU_60
129S1/SvImJ
A/J
C57BL/6J
NOD/ShiLtJ
NZO/HlLtJ
PWK/PhJ
Mean value
25
20
15
10
2013−01−15
2013−04−16
2013−07−16
2013−10−14
2014−01−13
Animal shipping date

Procedure: GMC11
Parameter: GLU_vol_inj

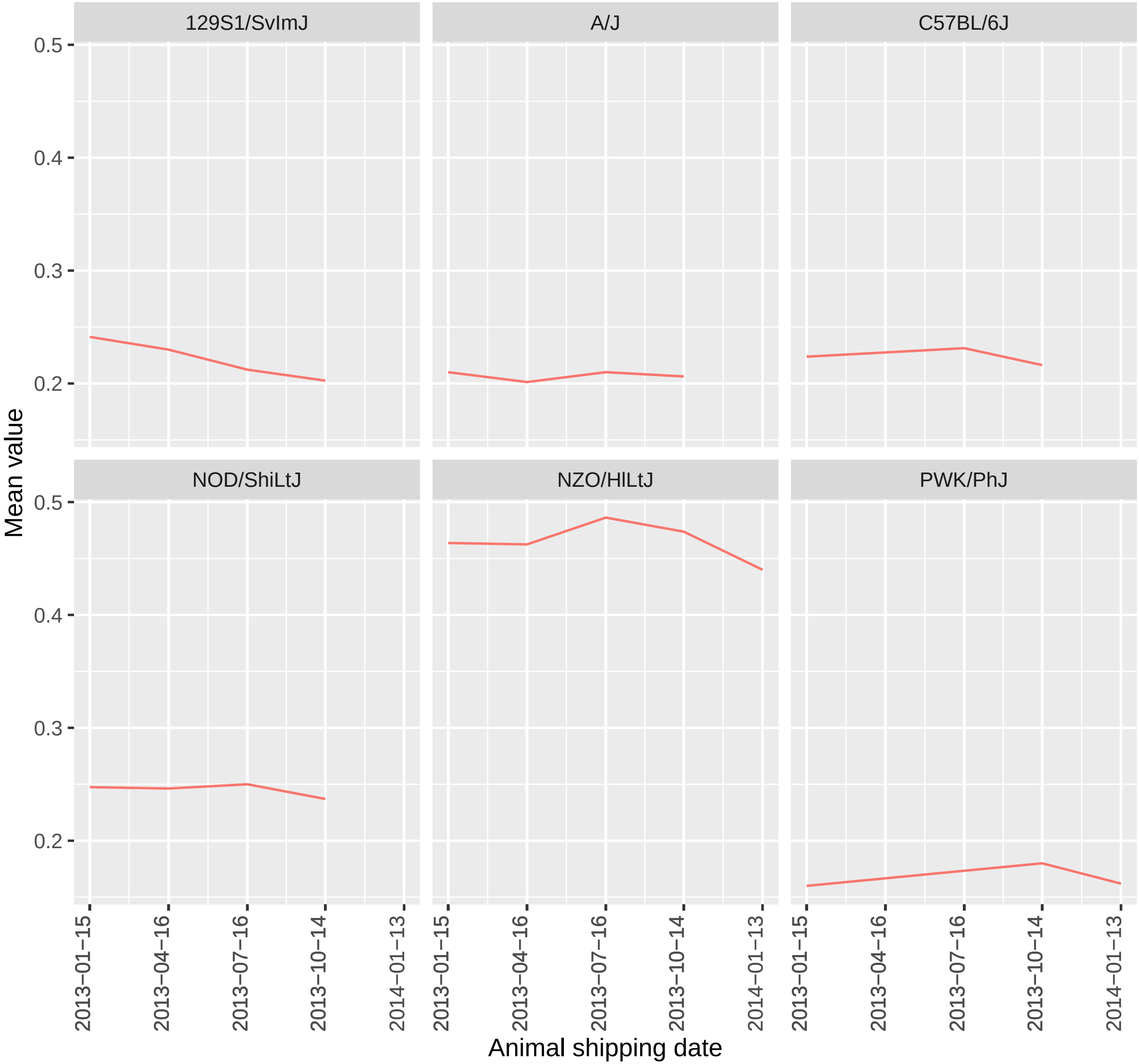

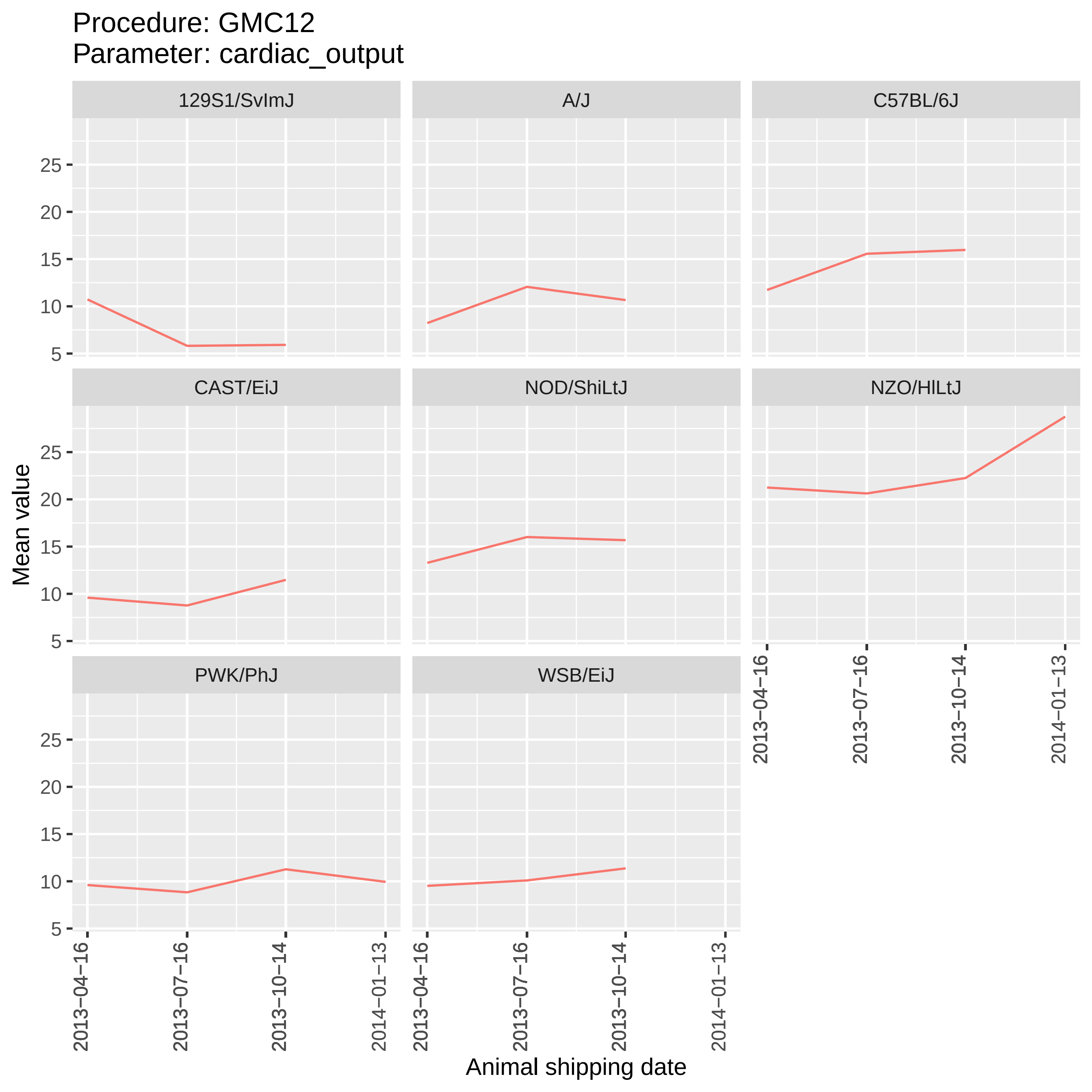

Procedure: GMC12
Parameter: cardiac_output
129S1/SvImJ
A/J
C57BL/6J
CAST/EiJ
NOD/ShiLtJ
NZO/HlLtJ
PWK/PhJ
WSB/EiJ
Mean value
5
10
15
20
25
2013−04−16
2013−07−16
2013−10−14
2014−01−13
Animal shipping date

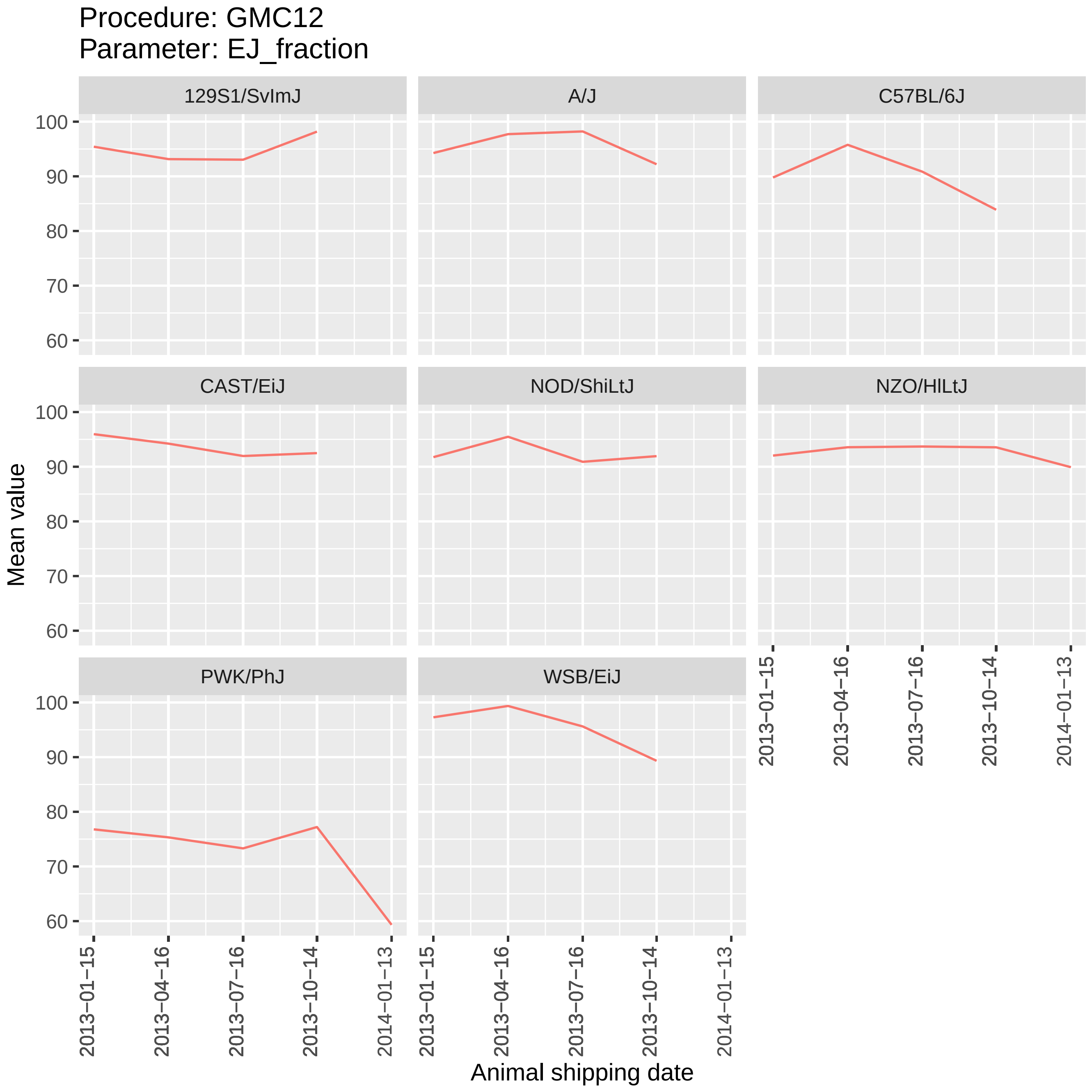
Procedure: GMC12
Parameter: EJ_fraction
129S1/SvImJ
A/J
C57BL/6J
CAST/EiJ
NOD/ShiLtJ
NZO/HlLtJ
PWK/PhJ
WSB/EiJ
Mean value
100
90
80
70
60
2013−01−15
2013−04−16
2013−07−16
2013−10−14
2014−01−13
Animal shipping date

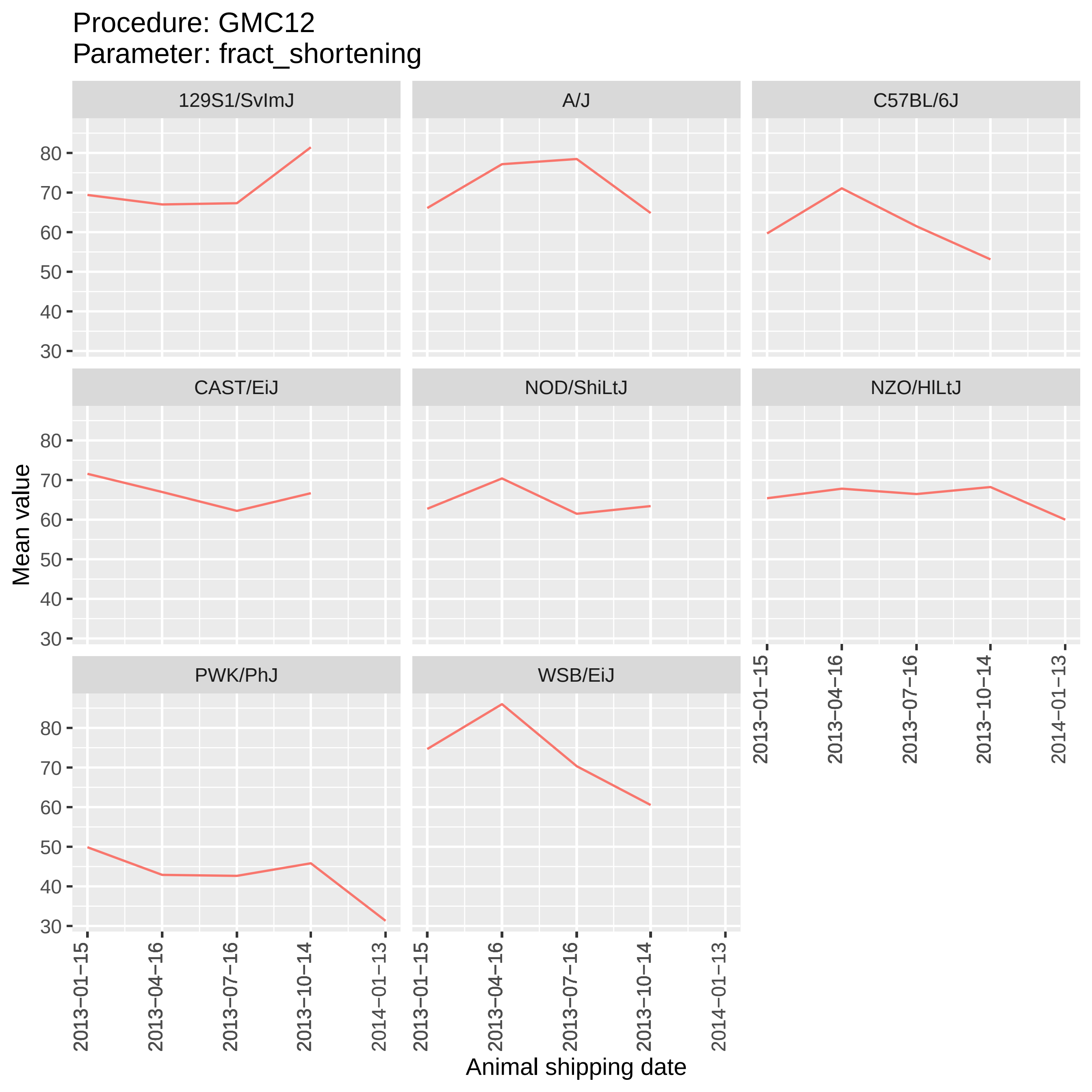

Procedure: GMC12
Parameter: fract_shortening
129S1/SvImJ
A/J
C57BL/6J
CAST/EiJ
NOD/ShiLtJ
NZO/HlLtJ
PWK/PhJ
WSB/EiJ
Mean value
30
40
50
60
70
80
2013−01−15
2013−04−16
2013−07−16
2013−10−14
2014−01−13
Animal shipping date

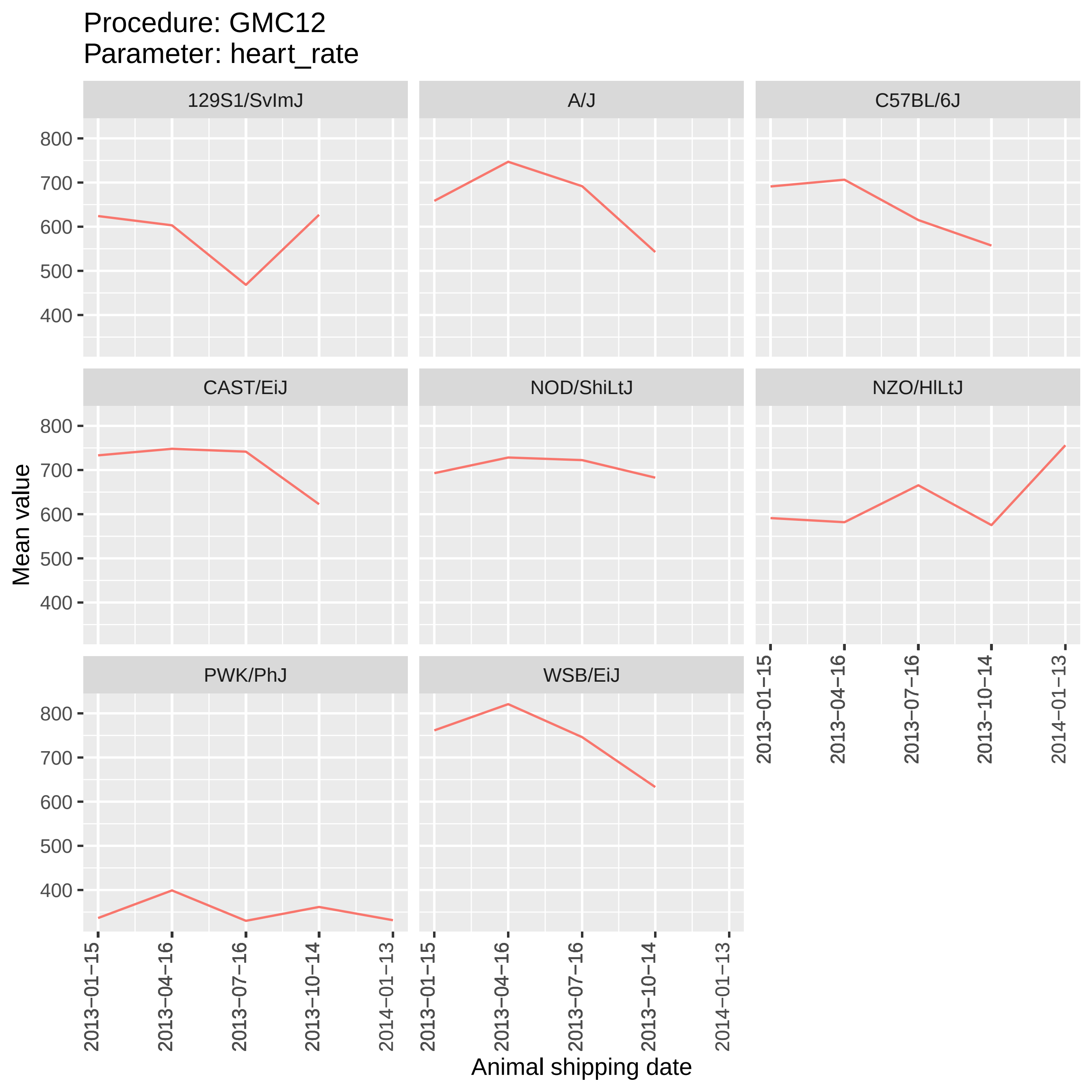

Procedure: GMC12
Parameter: heart_rate
129S1/SvImJ
A/J
C57BL/6J
CAST/EiJ
NOD/ShiLtJ
NZO/HlLtJ
PWK/PhJ
WSB/EiJ
Mean value
400
500
600
700
800
2013−01−15
2013−04−16
2013−07−16
2013−10−14
2014−01−13
Animal shipping date

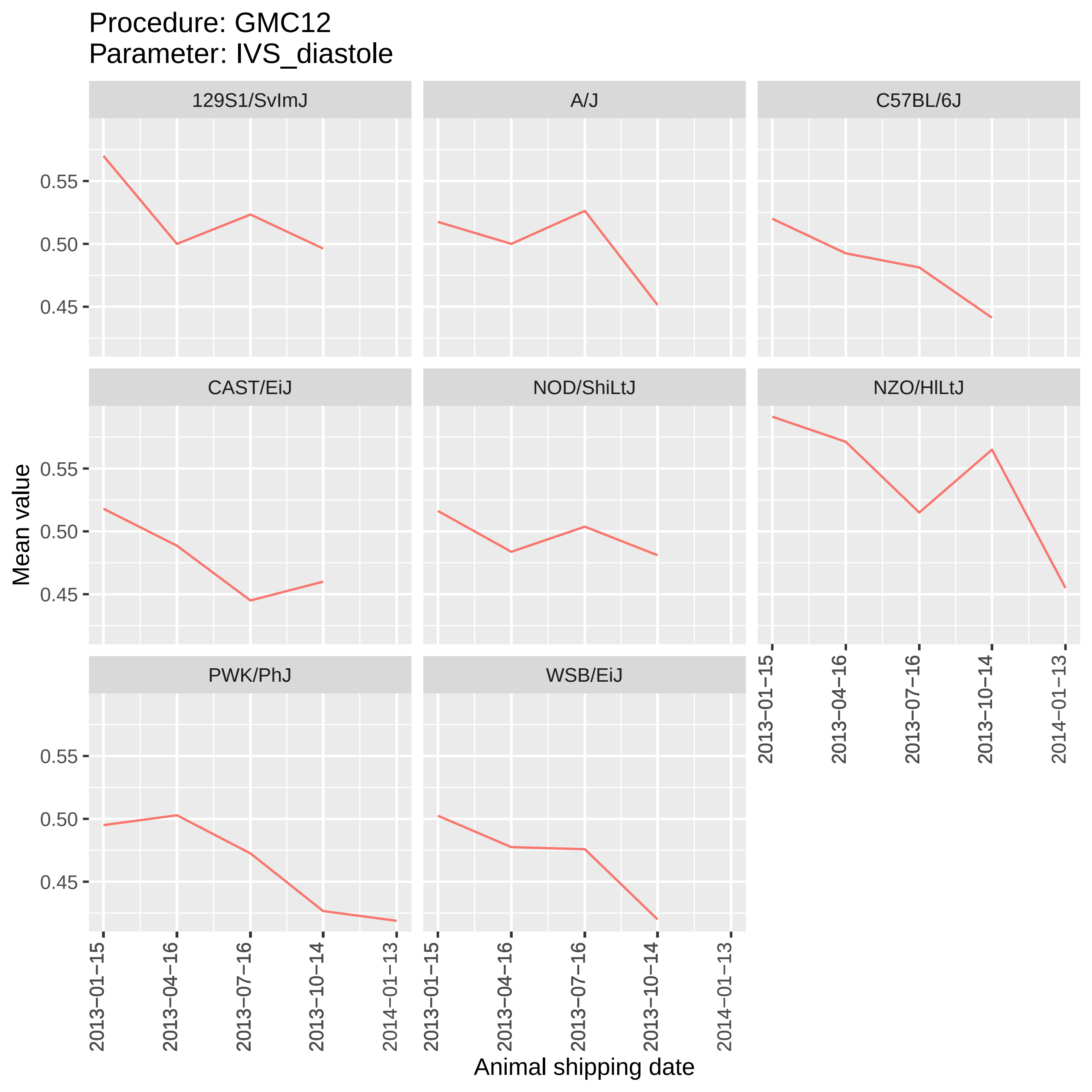

Procedure: GMC12
Parameter: IVS_diastole
129S1/SvImJ
A/J
C57BL/6J
CAST/EiJ
NOD/ShiLtJ
NZO/HlLtJ
PWK/PhJ
WSB/EiJ
0.55
0.50
0.45
Mean value
2013−01−15
2013−04−16
2013−07−16
2013−10−14
2014−01−13
Animal shipping date

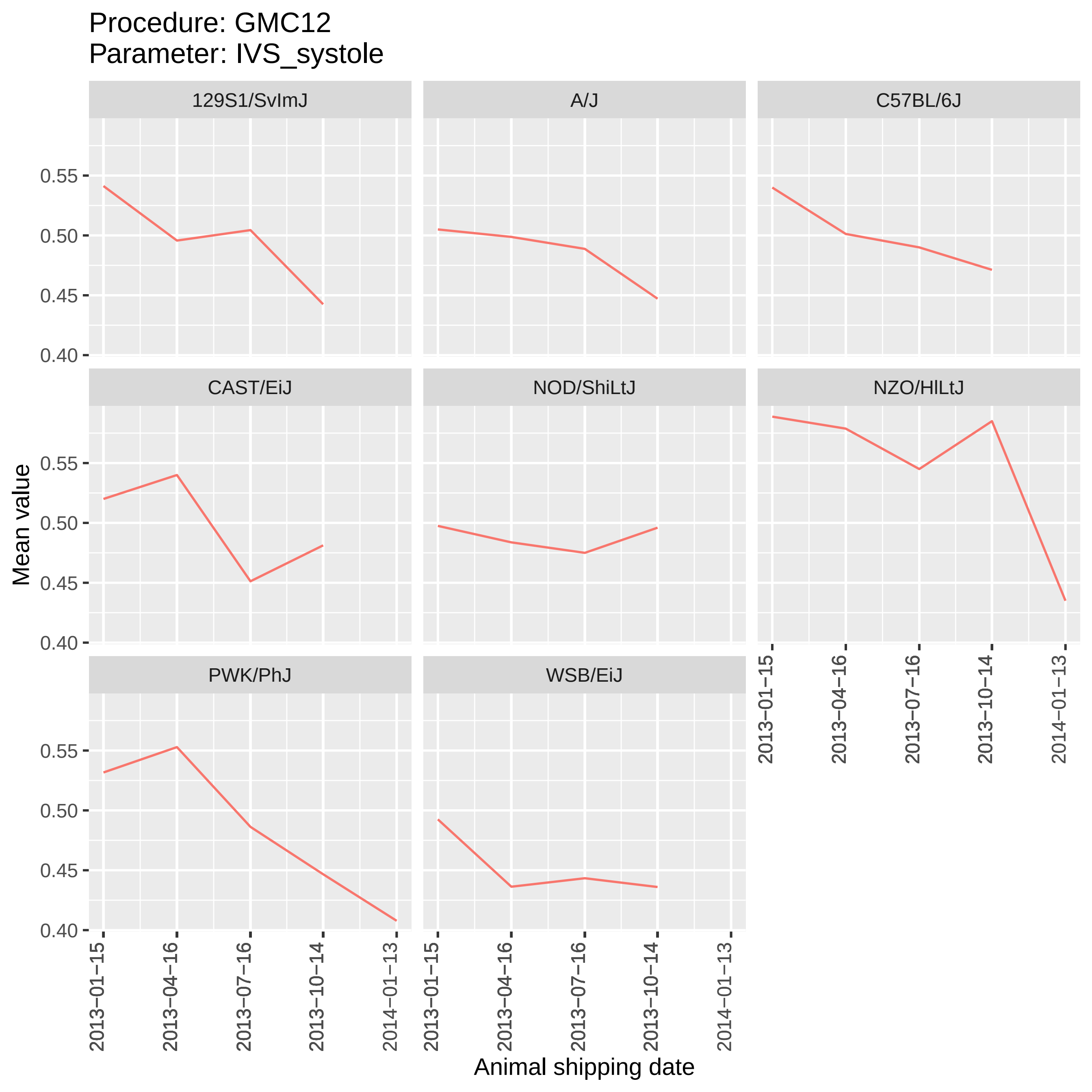

Procedure: GMC12
Parameter: IVS_systole
129S1/SvImJ
A/J
C57BL/6J
CAST/EiJ
NOD/ShiLtJ
NZO/HlLtJ
PWK/PhJ
WSB/EiJ
Mean value
0.40
0.45
0.50
0.55
2013−01−15
2013−04−16
2013−07−16
2013−10−14
2014−01−13
Animal shipping date

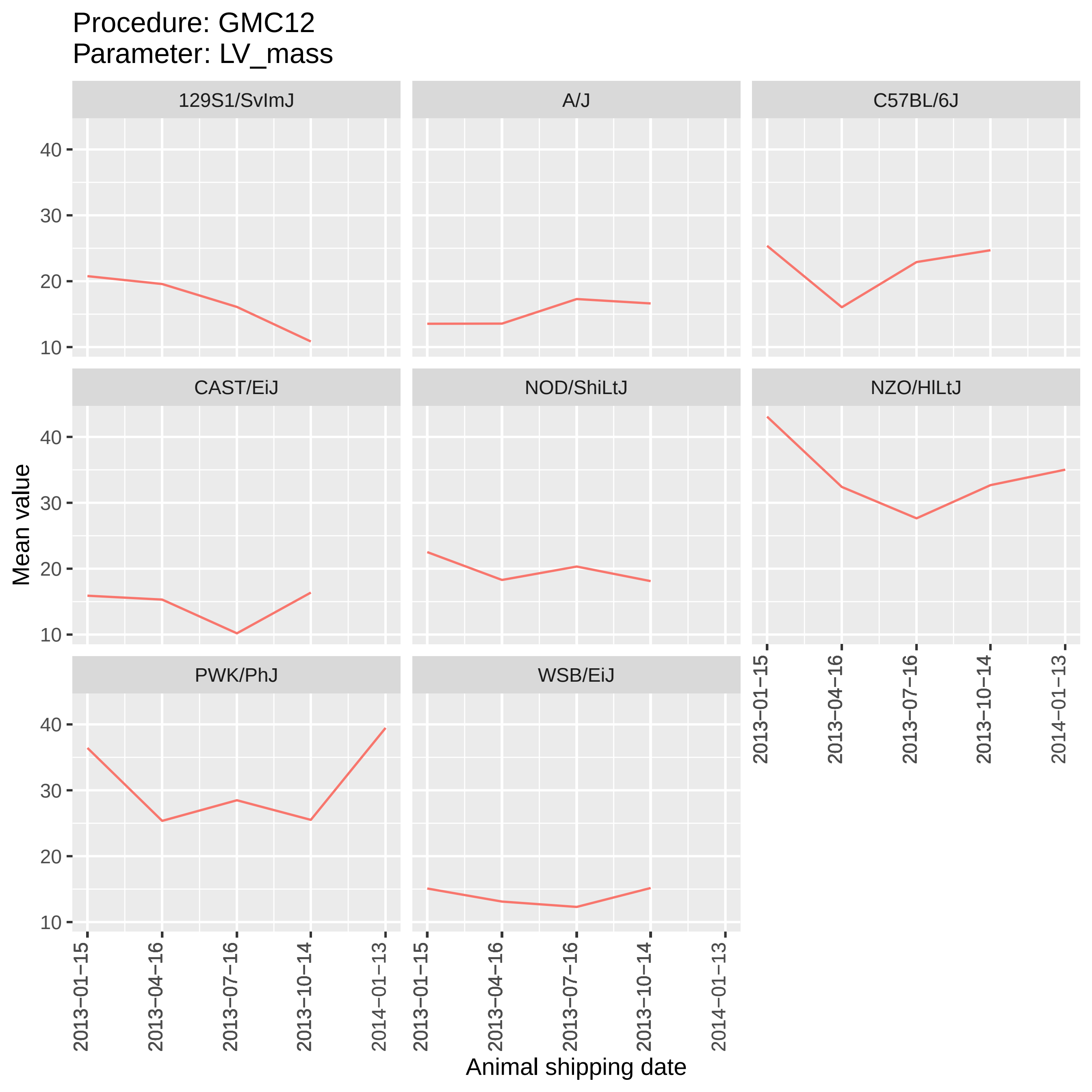

Procedure: GMC12
Parameter: LV_mass
129S1/SvImJ
A/J
C57BL/6J
CAST/EiJ
NOD/ShiLtJ
NZO/HlLtJ
PWK/PhJ
WSB/EiJ
Mean value
10
20
30
40
2013−01−15
2013−04−16
2013−07−16
2013−10−14
2014−01−13
Animal shipping date

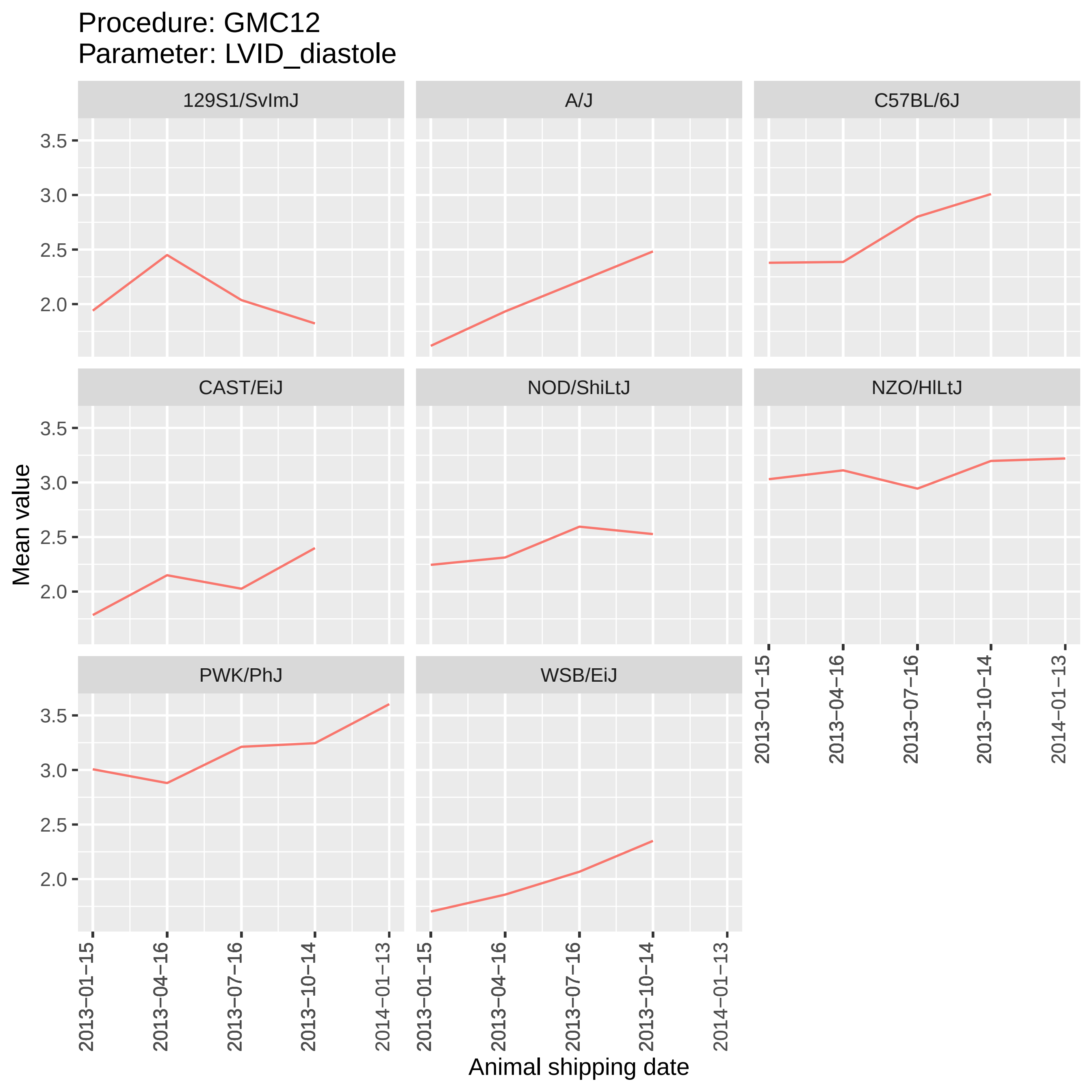
Procedure: GMC12
Parameter: LVID_diastole
129S1/SvImJ
A/J
C57BL/6J
CAST/EiJ
NOD/ShiLtJ
NZO/HlLtJ
PWK/PhJ
WSB/EiJ
Mean value
2.0
2.5
3.0
3.5
2013−01−15
2013−04−16
2013−07−16
2013−10−14
2014−01−13
Animal shipping date

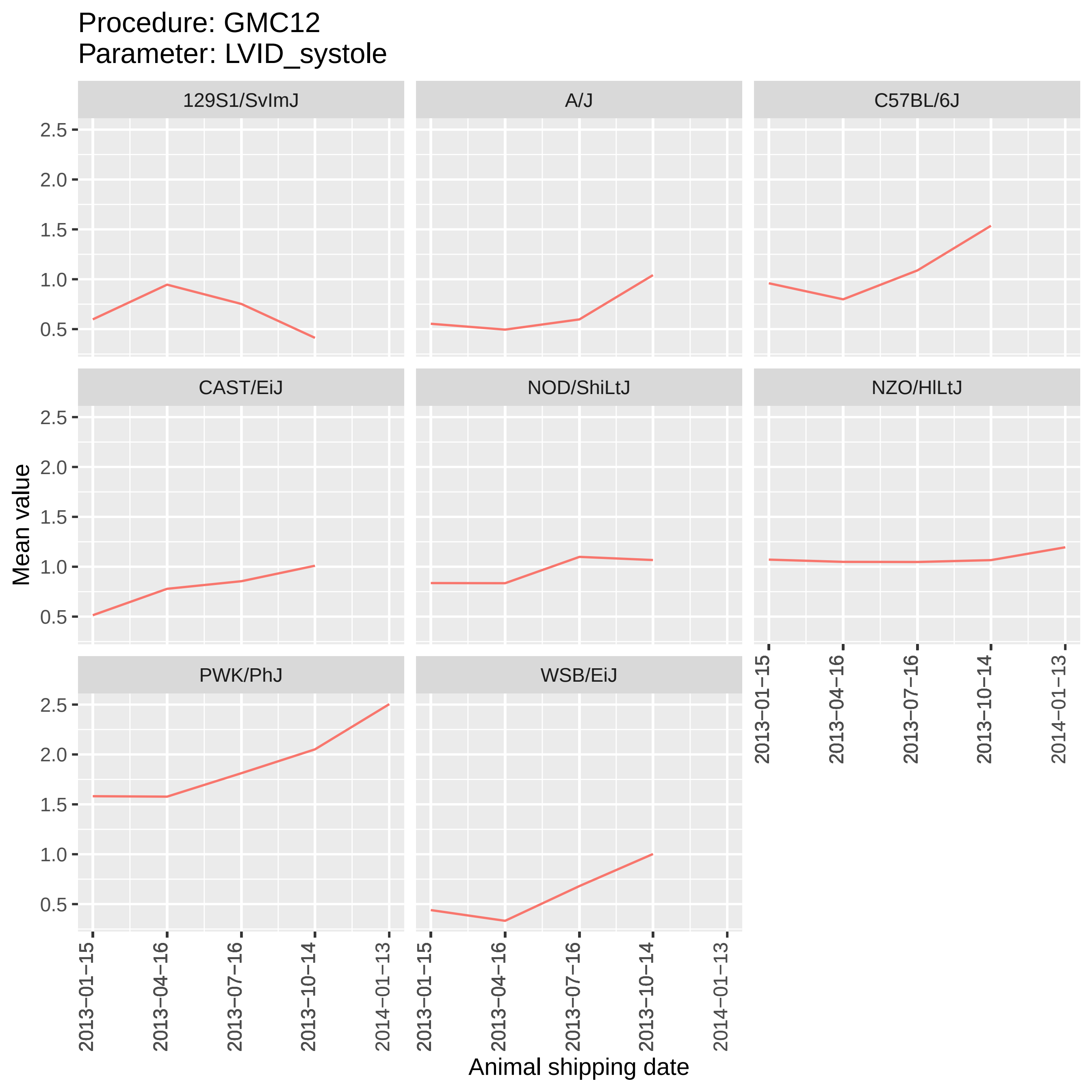
Procedure: GMC12
Parameter: LVID_systole
129S1/SvImJ
A/J
C57BL/6J
CAST/EiJ
NOD/ShiLtJ
NZO/HlLtJ
PWK/PhJ
WSB/EiJ
Mean value
2.5
2.0
1.5
1.0
0.5
2013−01−15
2013−04−16
2013−07−16
2013−10−14
2014−01−13
Animal shipping date

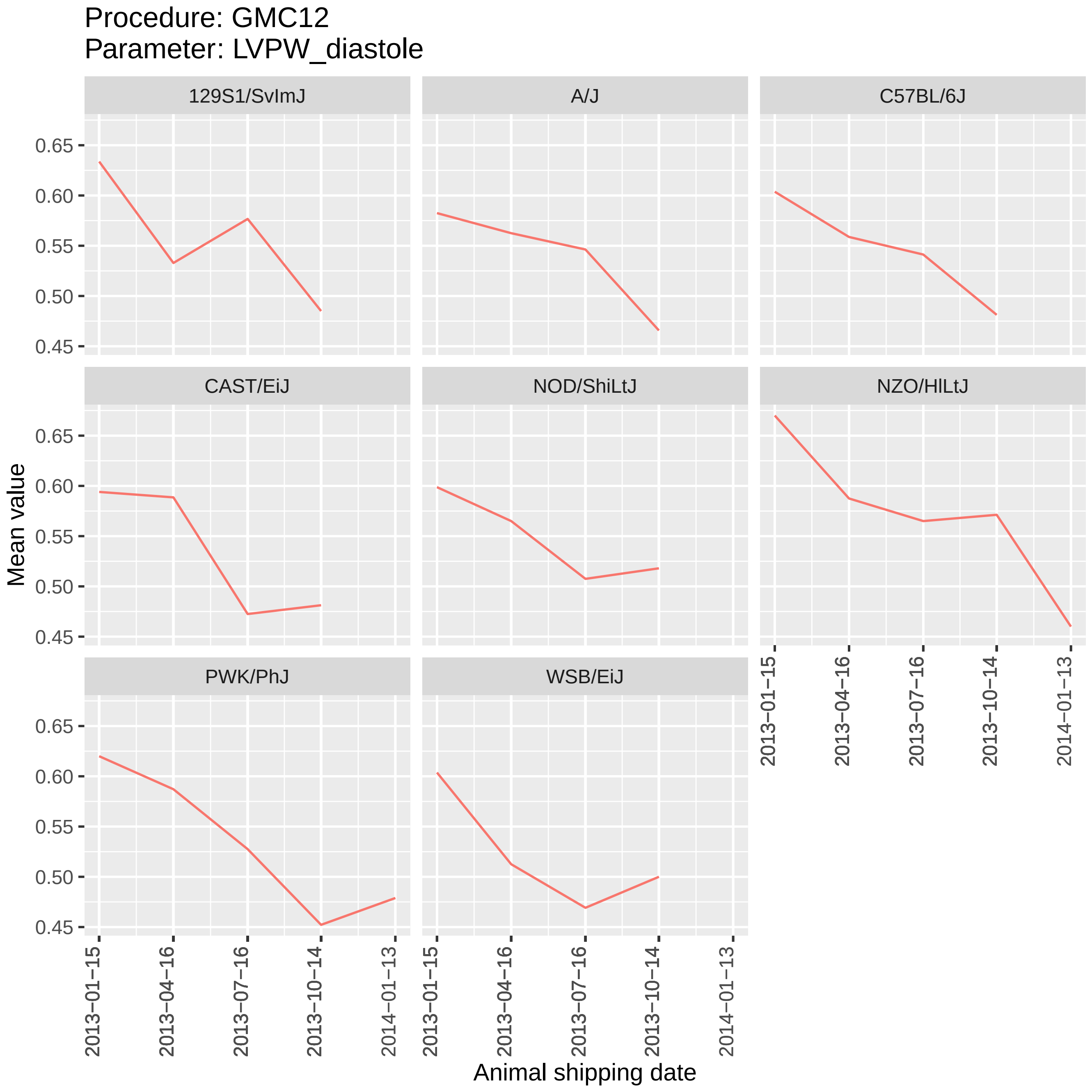

Procedure: GMC12
Parameter: LVPW_diastole
129S1/SvImJ
A/J
C57BL/6J
CAST/EiJ
NOD/ShiLtJ
NZO/HlLtJ
PWK/PhJ
WSB/EiJ
Mean value
0.45
0.50
0.55
0.60
0.65
2013−01−15
2013−04−16
2013−07−16
2013−10−14
2014−01−13
Animal shipping date

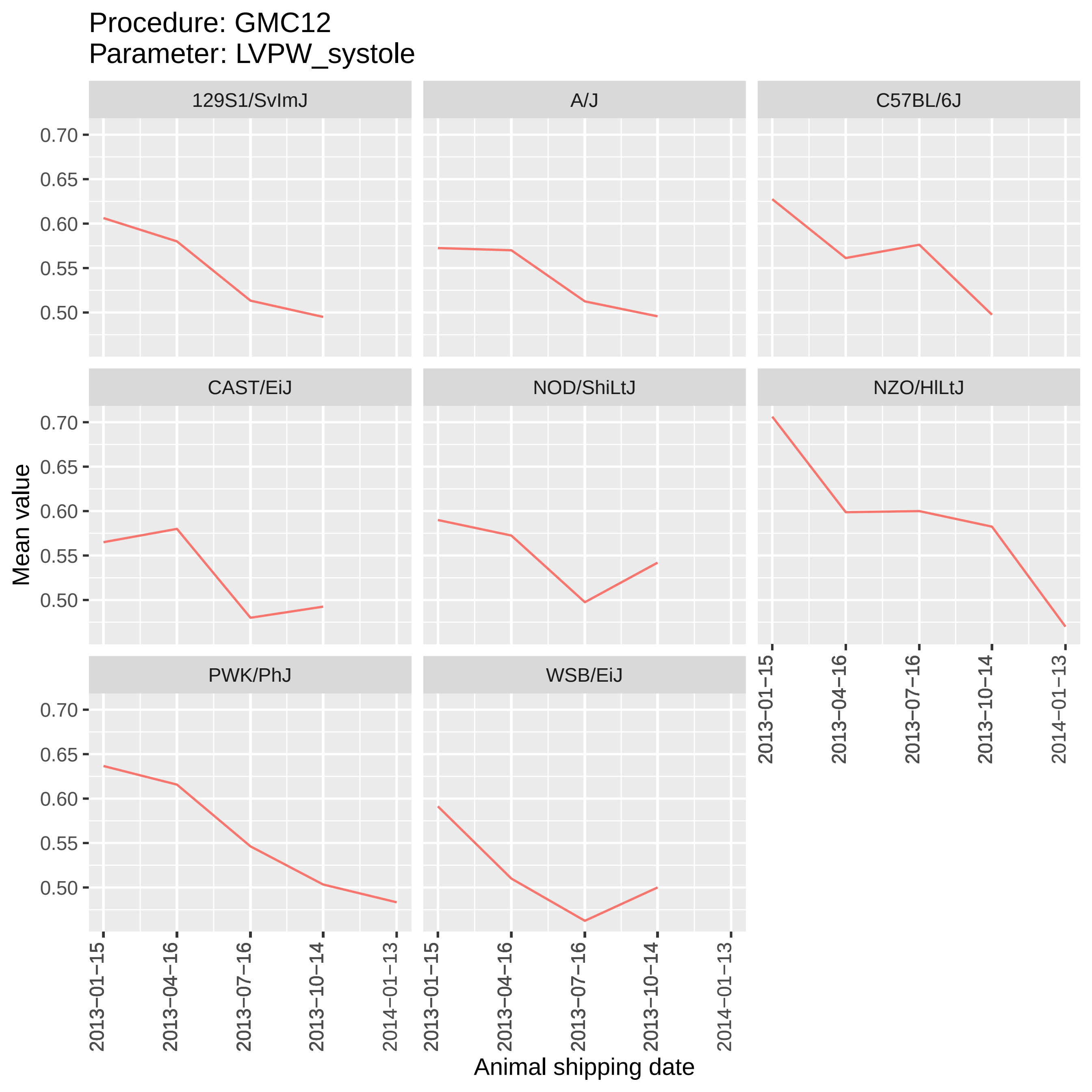

Procedure: GMC12
Parameter: LVPW_systole
129S1/SvImJ
A/J
C57BL/6J
CAST/EiJ
NOD/ShiLtJ
NZO/HlLtJ
PWK/PhJ
WSB/EiJ
Mean value
0.70
0.65
0.60
0.55
0.50
2013−01−15
2013−04−16
2013−07−16
2013−10−14
2014−01−13
Animal shipping date

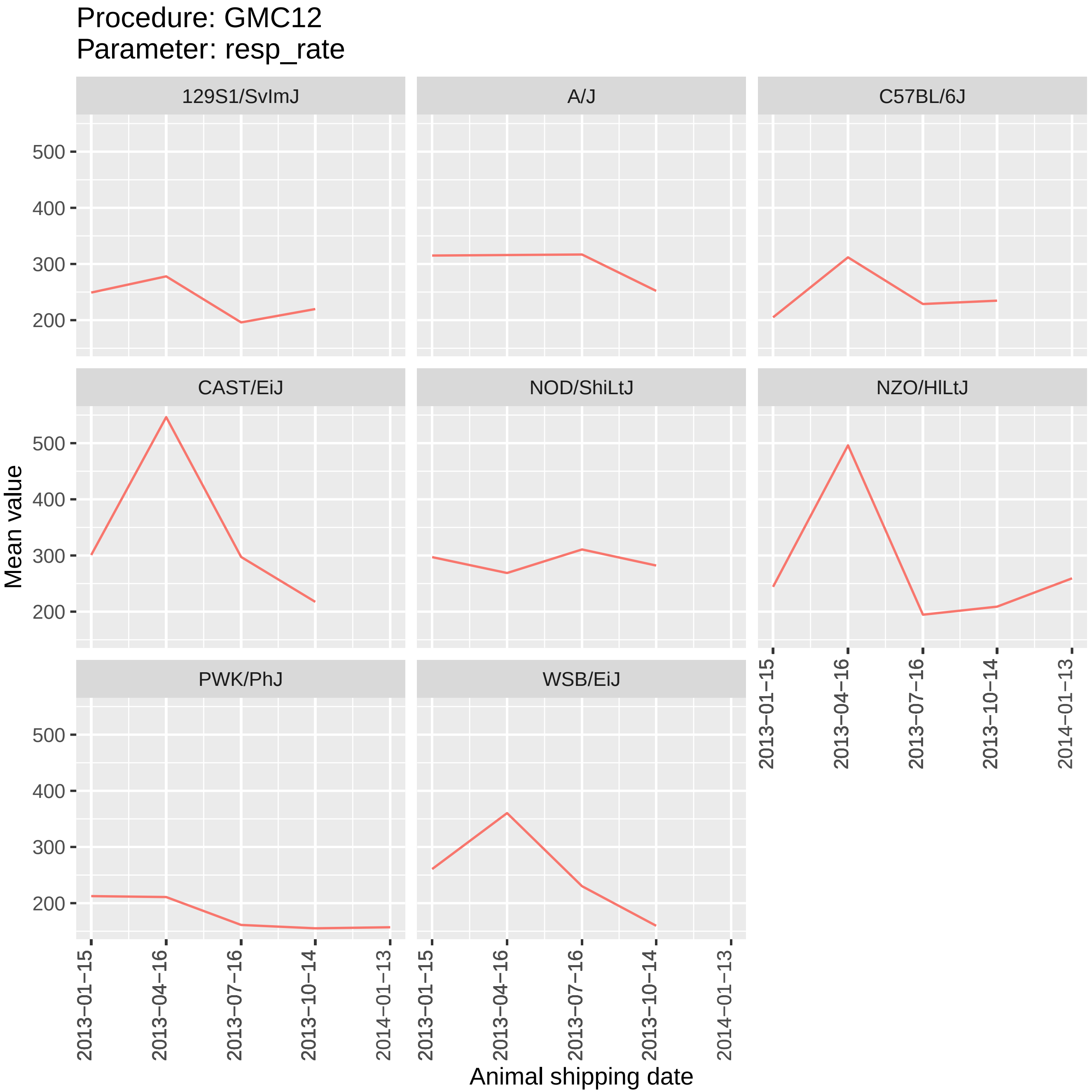

Procedure: GMC12
Parameter: resp_rate
129S1/SvImJ
A/J
C57BL/6J
CAST/EiJ
NOD/ShiLtJ
NZO/HlLtJ
PWK/PhJ
WSB/EiJ
Mean value
500
400
300
200
2013−01−15
2013−04−16
2013−07−16
2013−10−14
2014−01−13
Animal shipping date

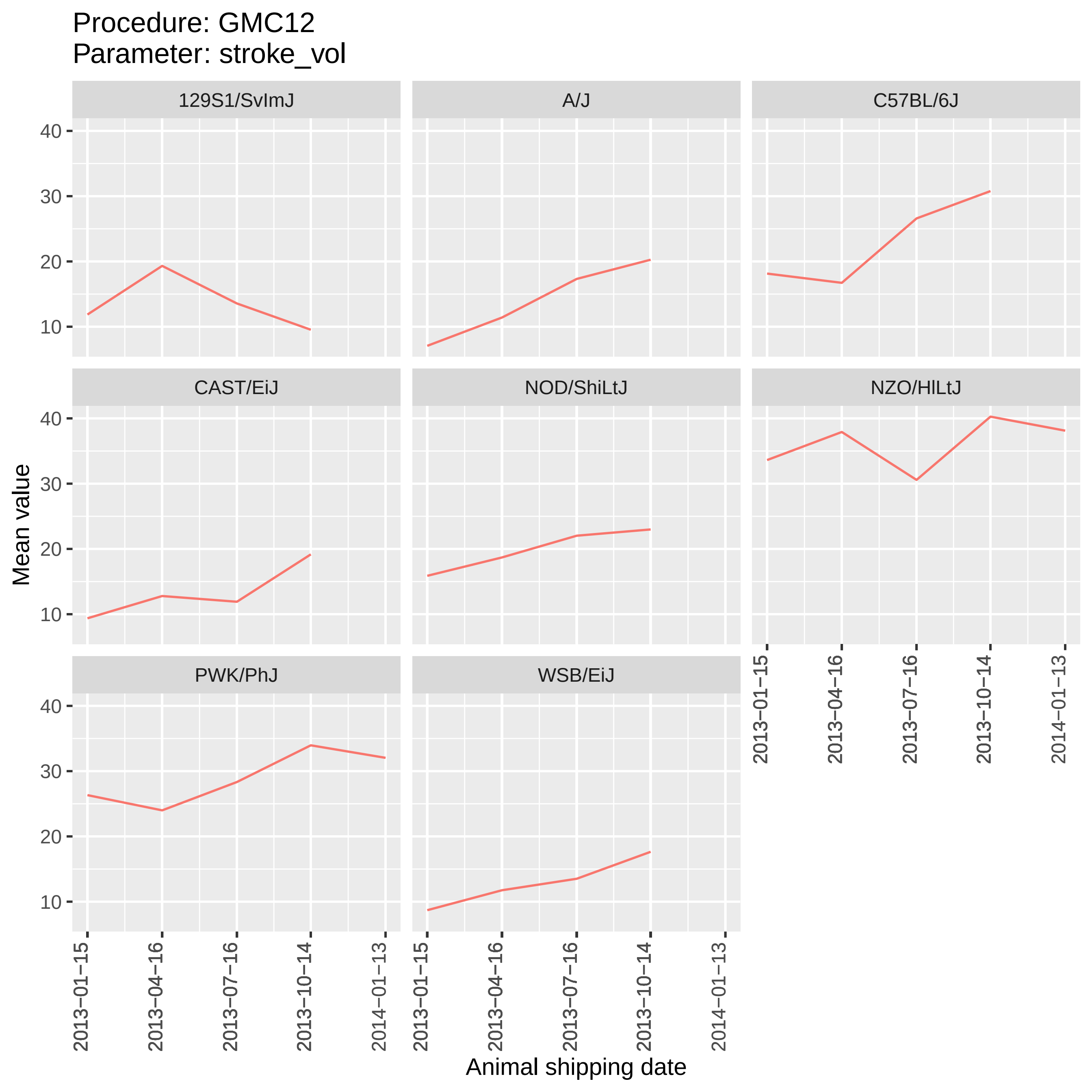

Procedure: GMC12
Parameter: stroke_vol
129S1/SvImJ
A/J
C57BL/6J
CAST/EiJ
NOD/ShiLtJ
NZO/HlLtJ
PWK/PhJ
WSB/EiJ
Mean value
10
20
30
40
2013−01−15
2013−04−16
2013−07−16
2013−10−14
2014−01−13
Animal shipping date

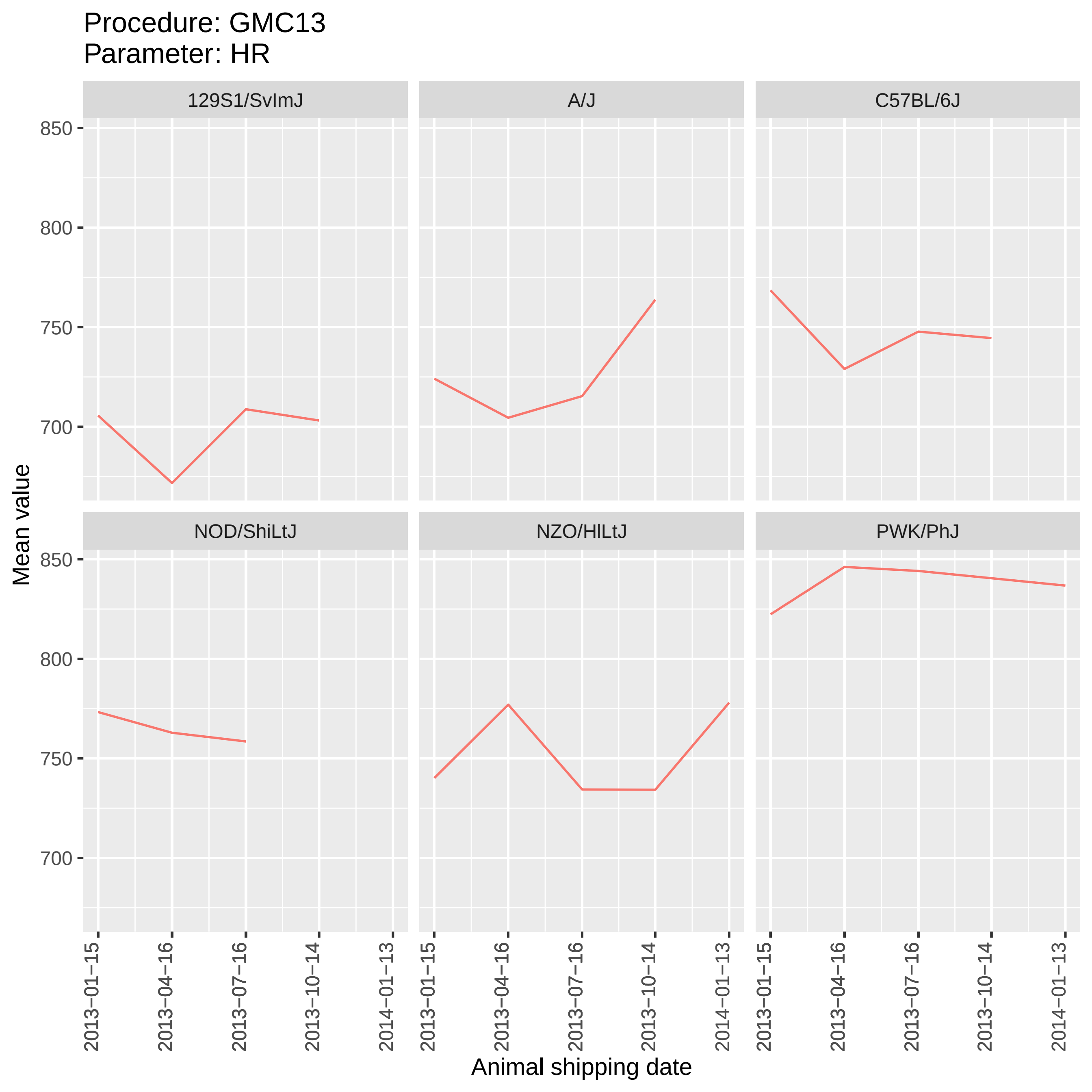

Procedure: GMC13
Parameter: HR
129S1/SvImJ
A/J
C57BL/6J
NOD/ShiLtJ
NZO/HlLtJ
PWK/PhJ
Mean value
850
800
750
700
2013−01−15
2013−04−16
2013−07−16
2013−10−14
2014−01−13
Animal shipping date

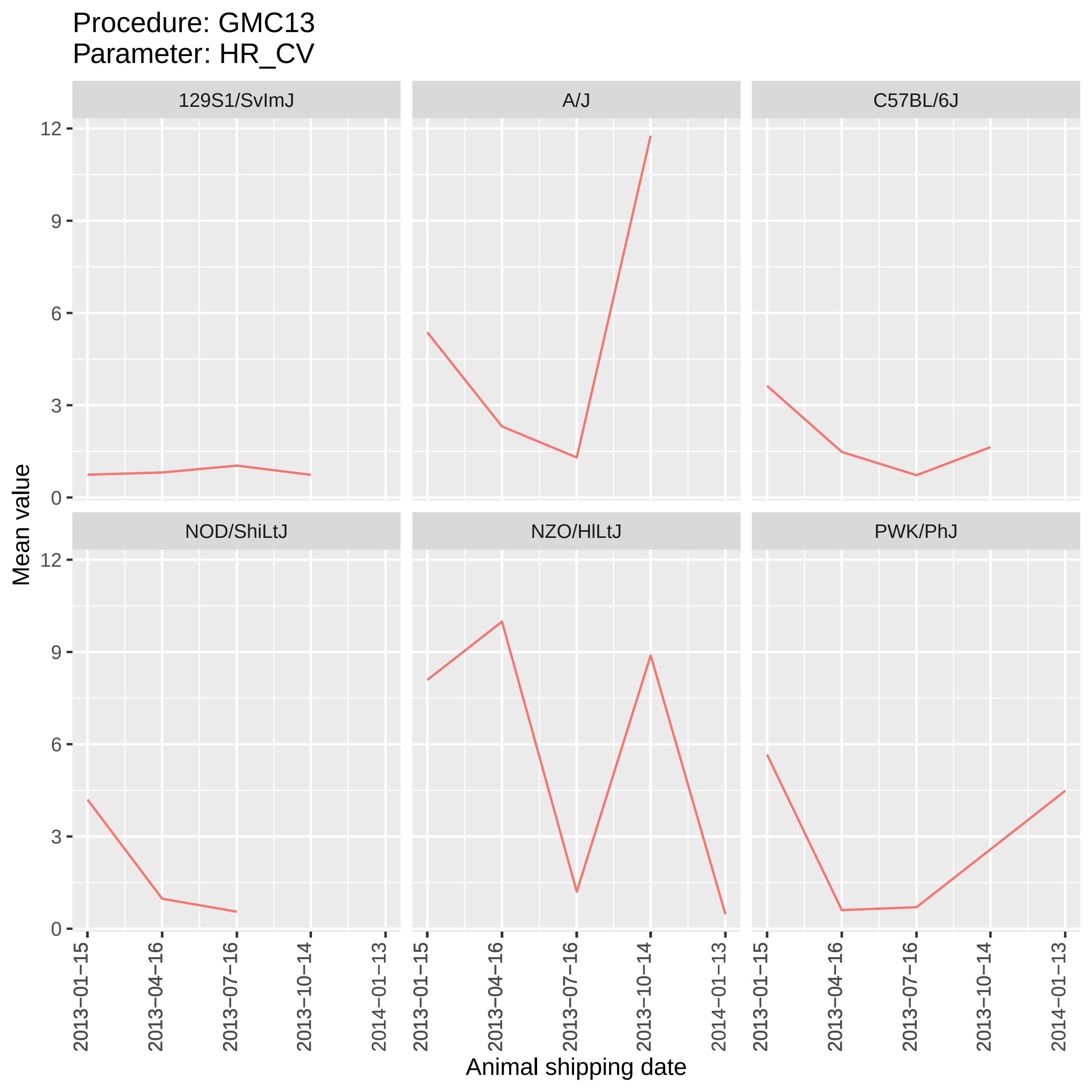
Procedure: GMC13
Parameter: HR_CV
129S1/SvImJ
A/J
C57BL/6J
NOD/ShiLtJ
NZO/HlLtJ
PWK/PhJ
Mean value
0
3
6
9
12
2013−01−15
2013−04−16
2013−07−16
2013−10−14
2014−01−13
Animal shipping date

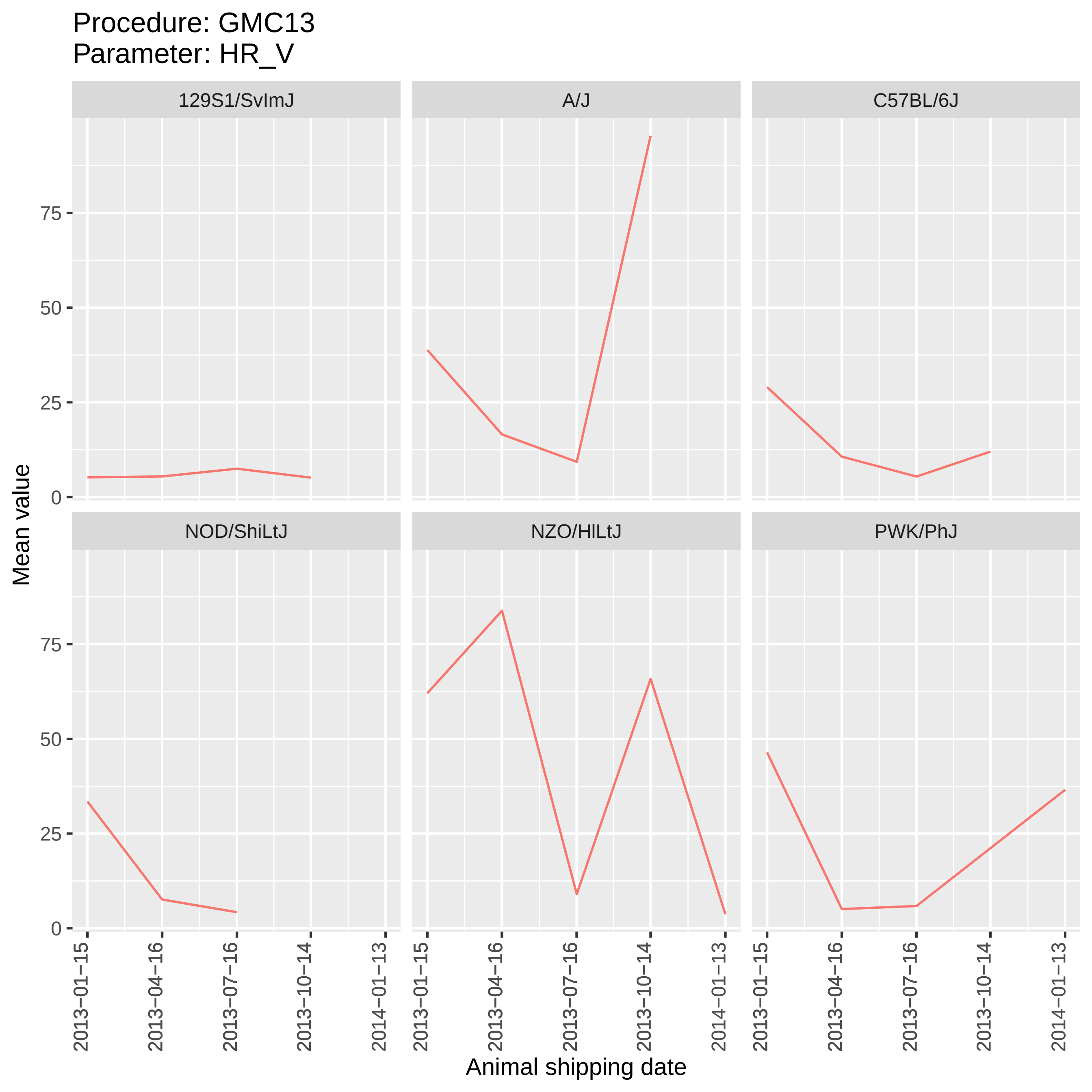

Procedure: GMC13
Parameter: HR_V
129S1/SvImJ
A/J
C57BL/6J
NOD/ShiLtJ
NZO/HlLtJ
PWK/PhJ
Mean value
0
25
50
75
2013−01−15
2013−04−16
2013−07−16
2013−10−14
2014−01−13
Animal shipping date

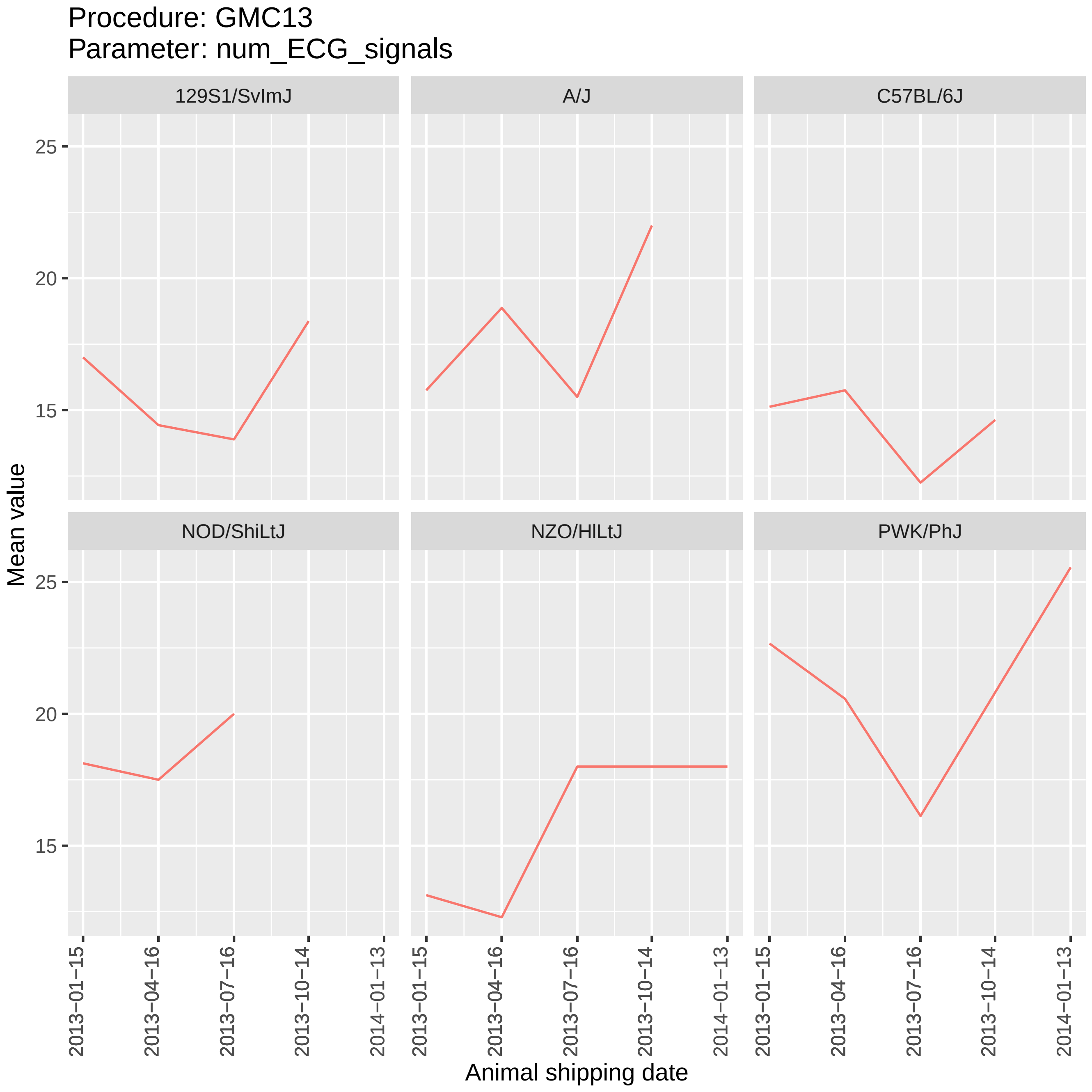

Procedure: GMC13
Parameter: num_ECG_signals
129S1/SvImJ
A/J
C57BL/6J
NOD/ShiLtJ
NZO/HlLtJ
PWK/PhJ
Mean value
25
20
15
Animal shipping date
2013−01−15
2013−04−16
2013−07−16
2013−10−14
2014−01−13

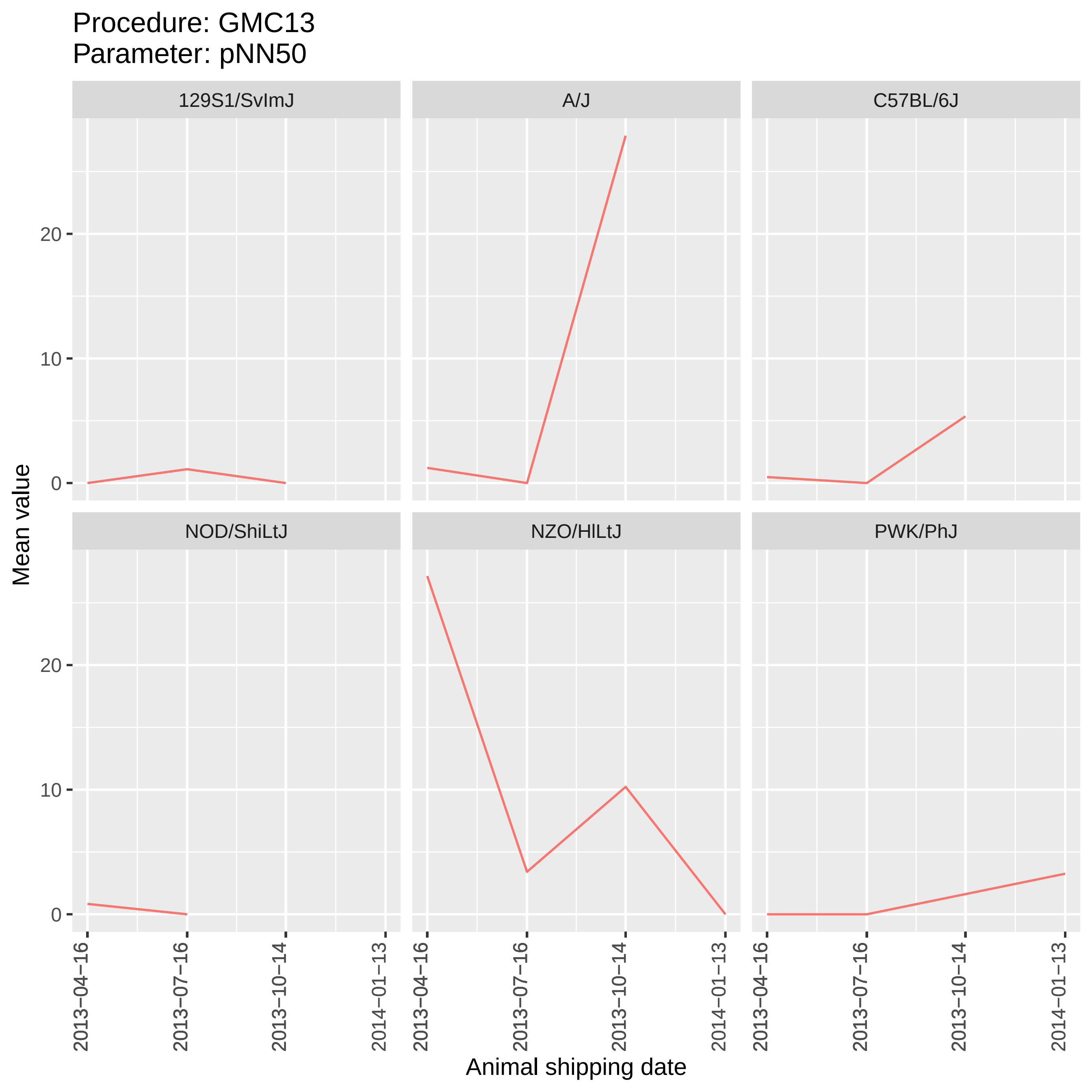

Procedure: GMC13
Parameter: pNN50
129S1/SvImJ
A/J
C57BL/6J
NOD/ShiLtJ
NZO/HlLtJ
PWK/PhJ
Mean value
0
10
20
2013−04−16
2013−07−16
2013−10−14
2014−01−13
Animal shipping date

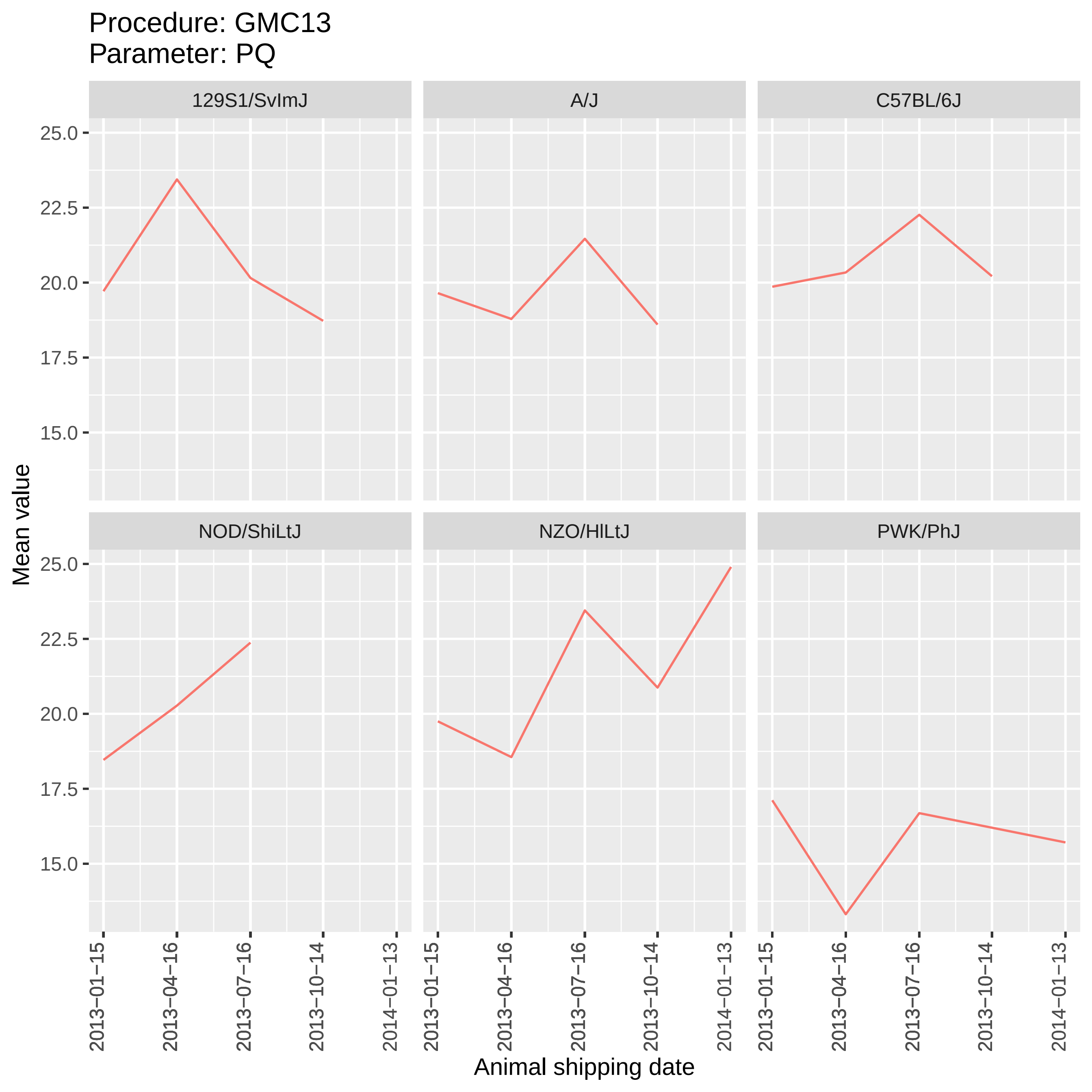
Procedure: GMC13
Parameter: PQ
129S1/SvImJ
A/J
C57BL/6J
NOD/ShiLtJ
NZO/HlLtJ
PWK/PhJ
Mean value
25.0
22.5
20.0
17.5
15.0
2013−01−15
2013−04−16
2013−07−16
2013−10−14
2014−01−13
Animal shipping date

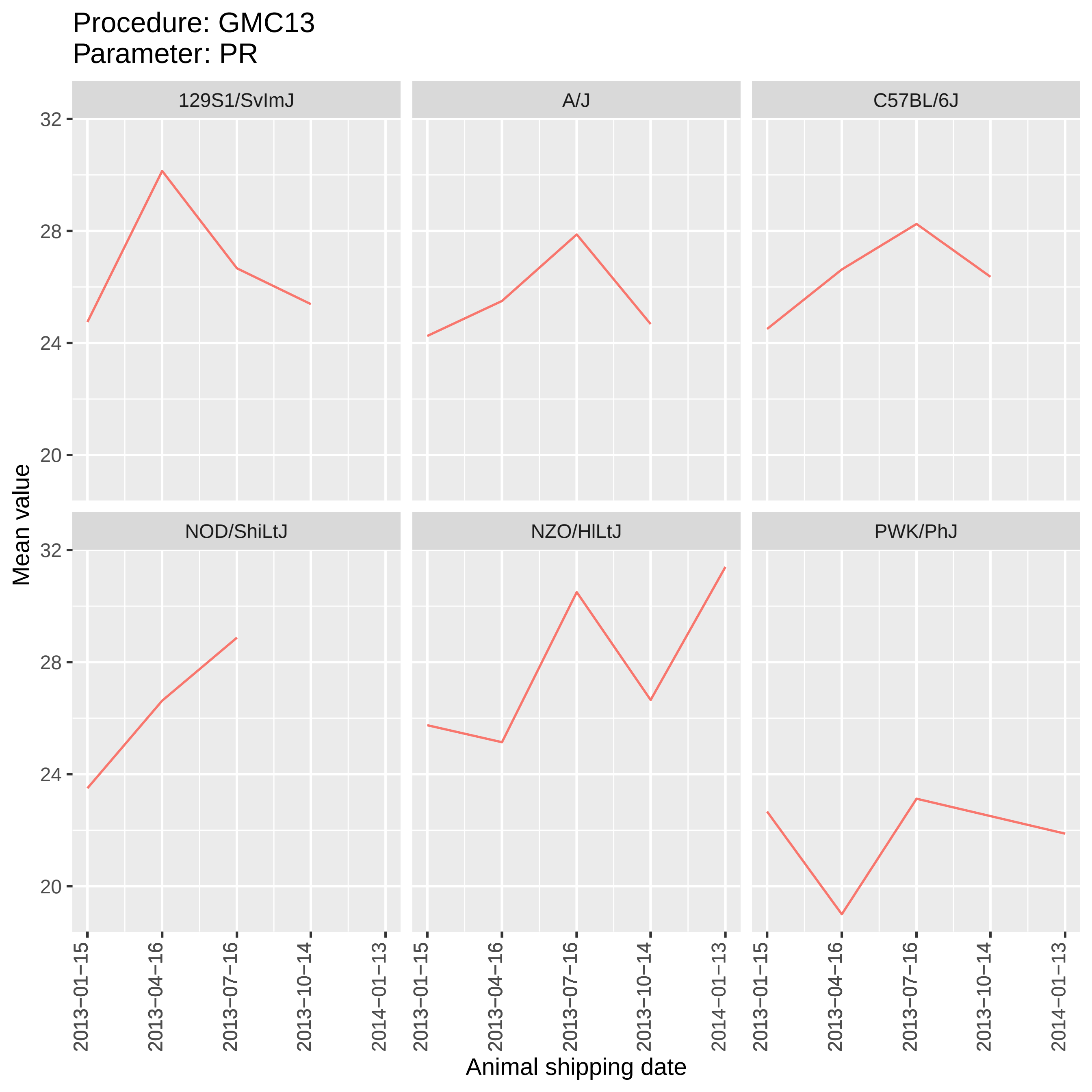

Procedure: GMC13
Parameter: PR
129S1/SvImJ
A/J
C57BL/6J
NOD/ShiLtJ
NZO/HlLtJ
PWK/PhJ
32
28
24
20
Mean value
2013−01−15
2013−04−16
2013−07−16
2013−10−14
2014−01−13
Animal shipping date

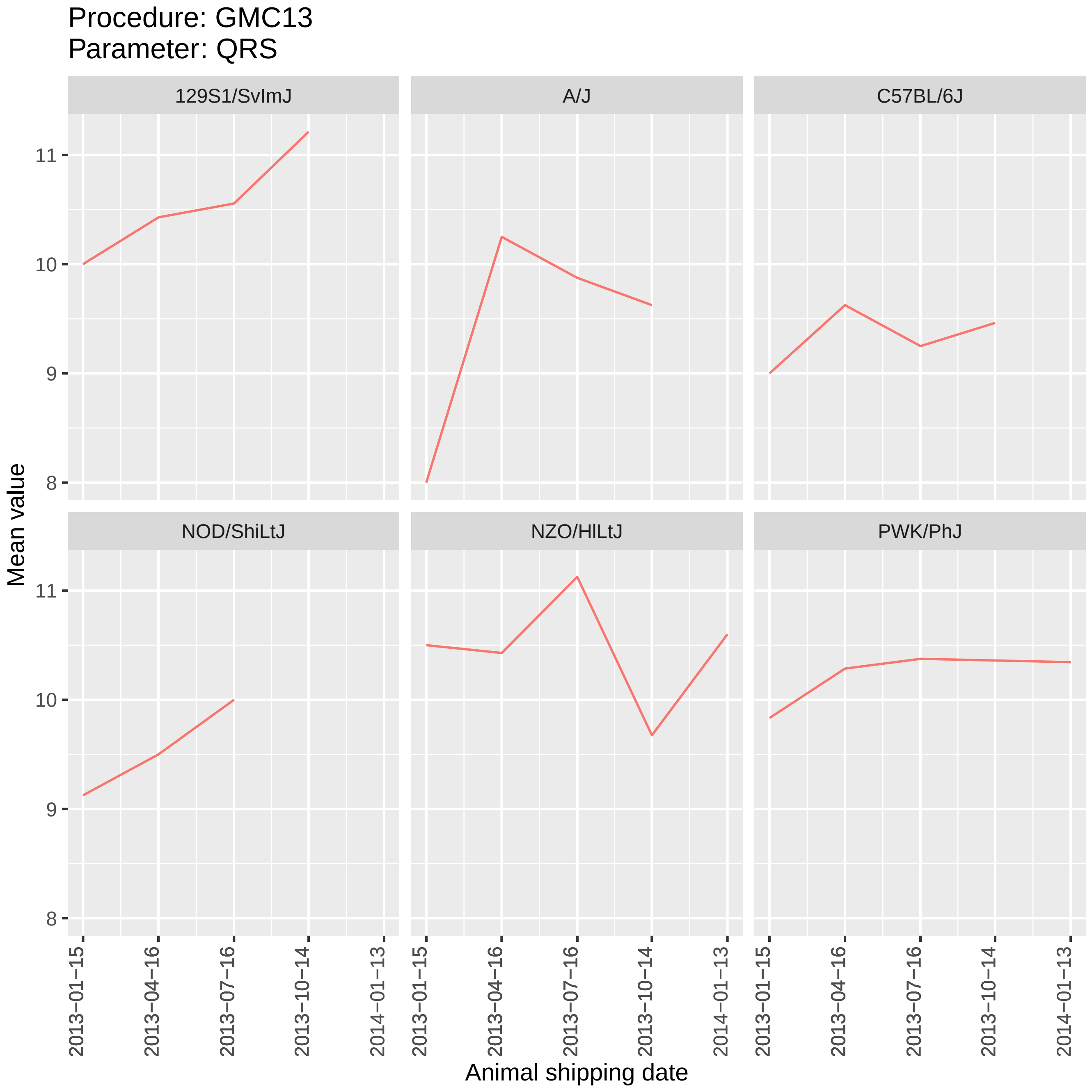
Procedure: GMC13
Parameter: QRS
129S1/SvImJ
A/J
C57BL/6J
NOD/ShiLtJ
NZO/HlLtJ
PWK/PhJ
Mean value
11
10
9
8
2013−01−15
2013−04−16
2013−07−16
2013−10−14
2014−01−13
Animal shipping date

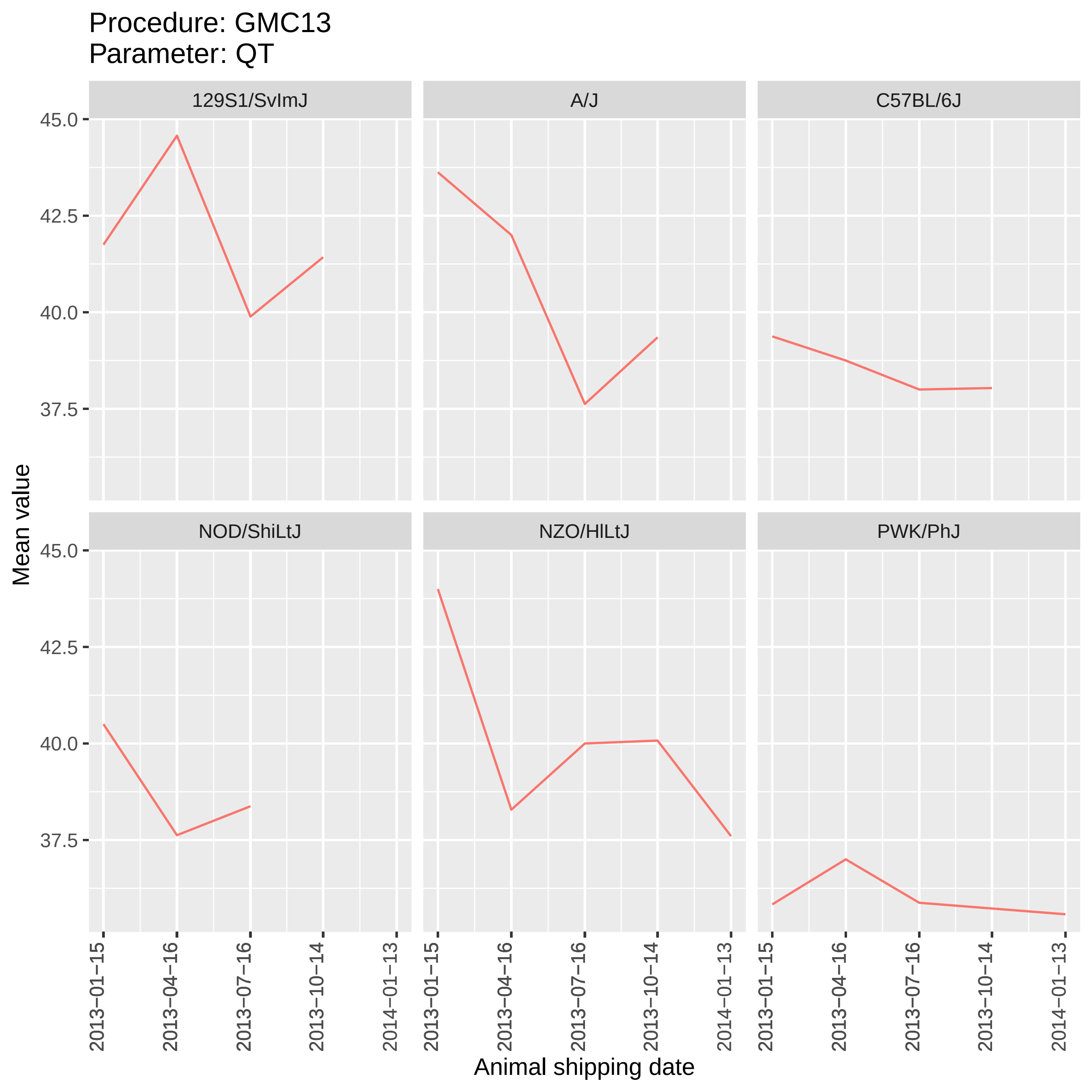
Procedure: GMC13
Parameter: QT
129S1/SvImJ
A/J
C57BL/6J
NOD/ShiLtJ
NZO/HlLtJ
PWK/PhJ
Mean value
45.0
42.5
40.0
37.5
2013−01−15
2013−04−16
2013−07−16
2013−10−14
2014−01−13
Animal shipping date

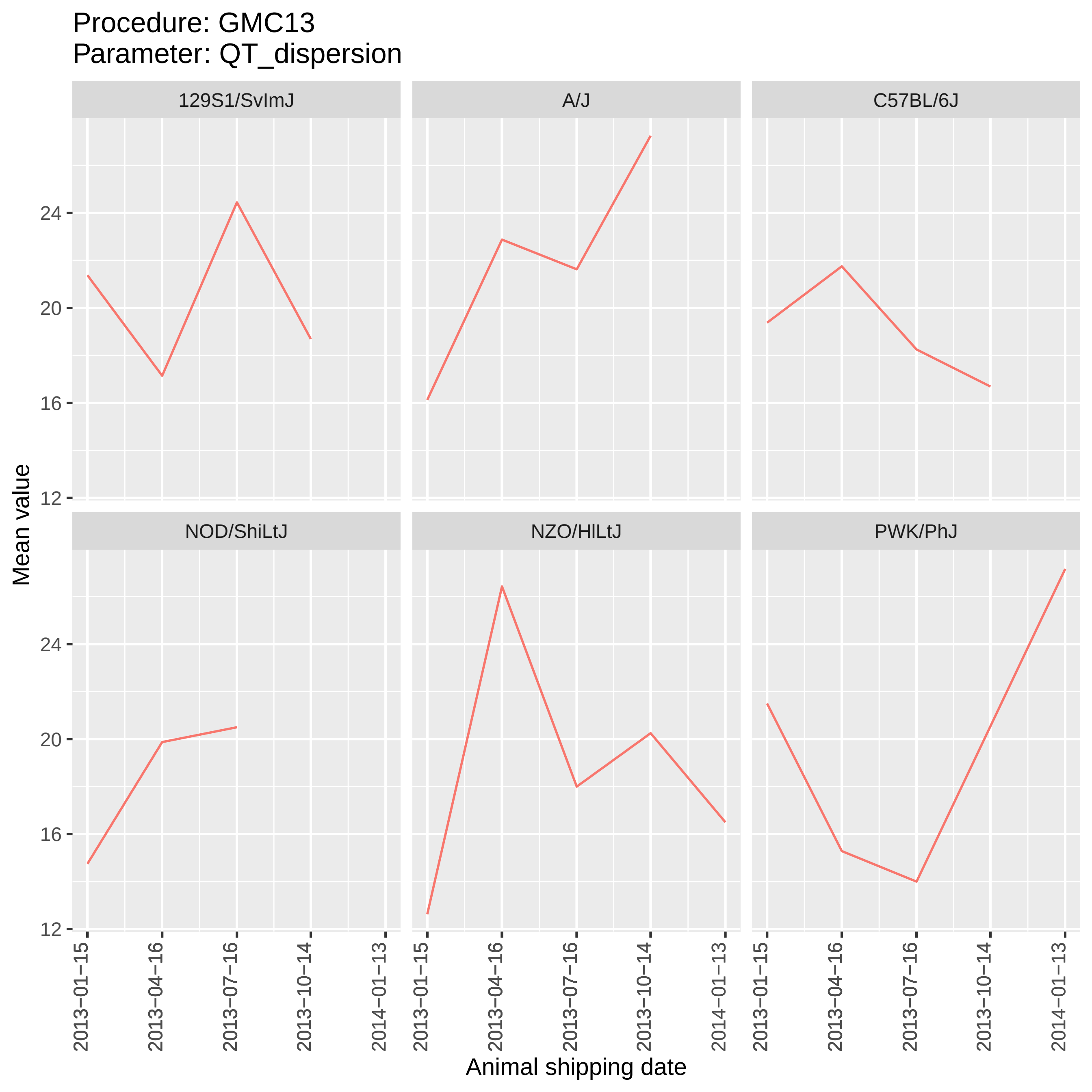
Procedure: GMC13
Parameter: QT_dispersion
129S1/SvImJ
A/J
C57BL/6J
NOD/ShiLtJ
NZO/HlLtJ
PWK/PhJ
Mean value
12
16
20
24
2013−01−15
2013−04−16
2013−07−16
2013−10−14
2014−01−13
Animal shipping date

Procedure: GMC13
Parameter: QTc

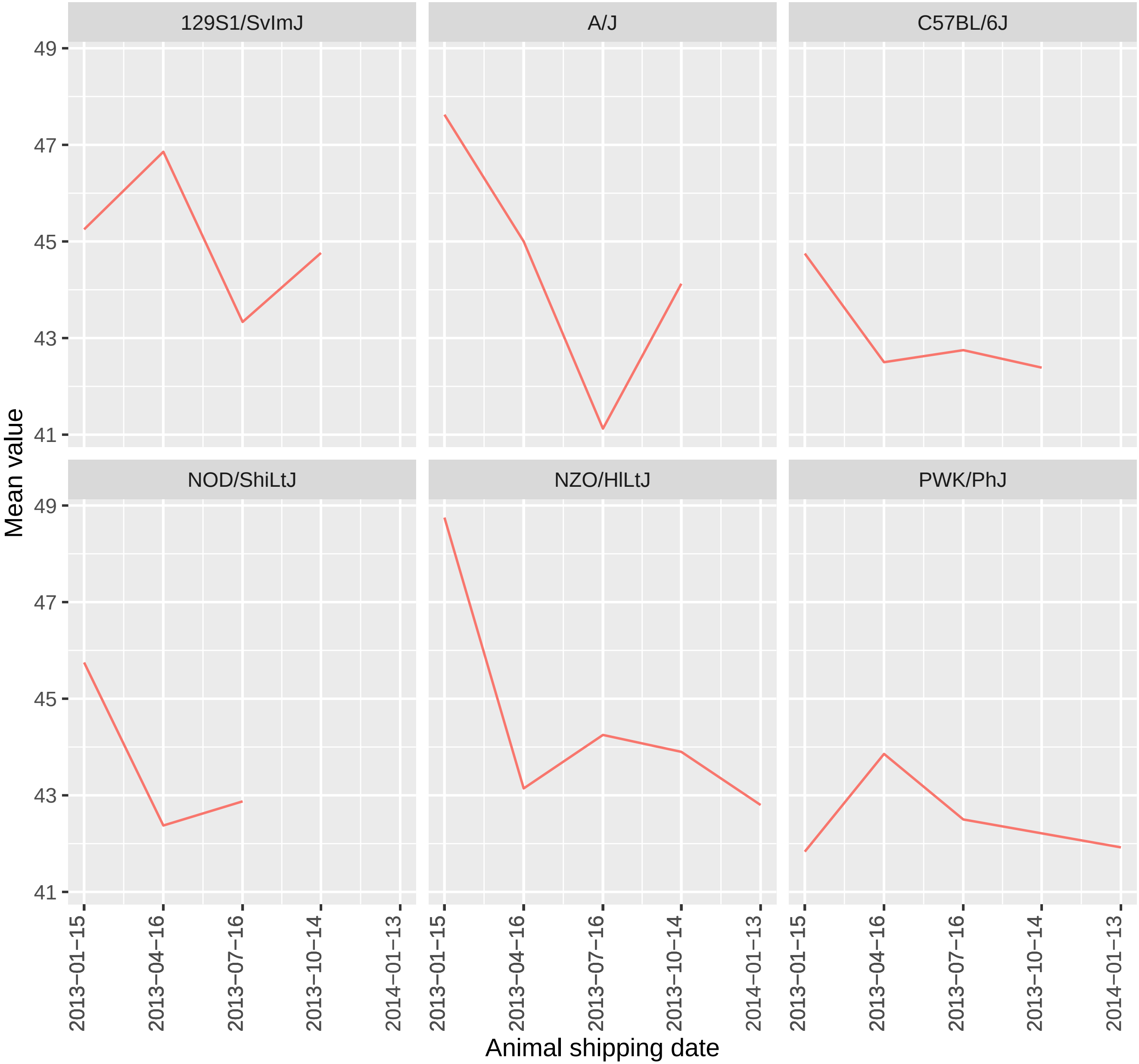

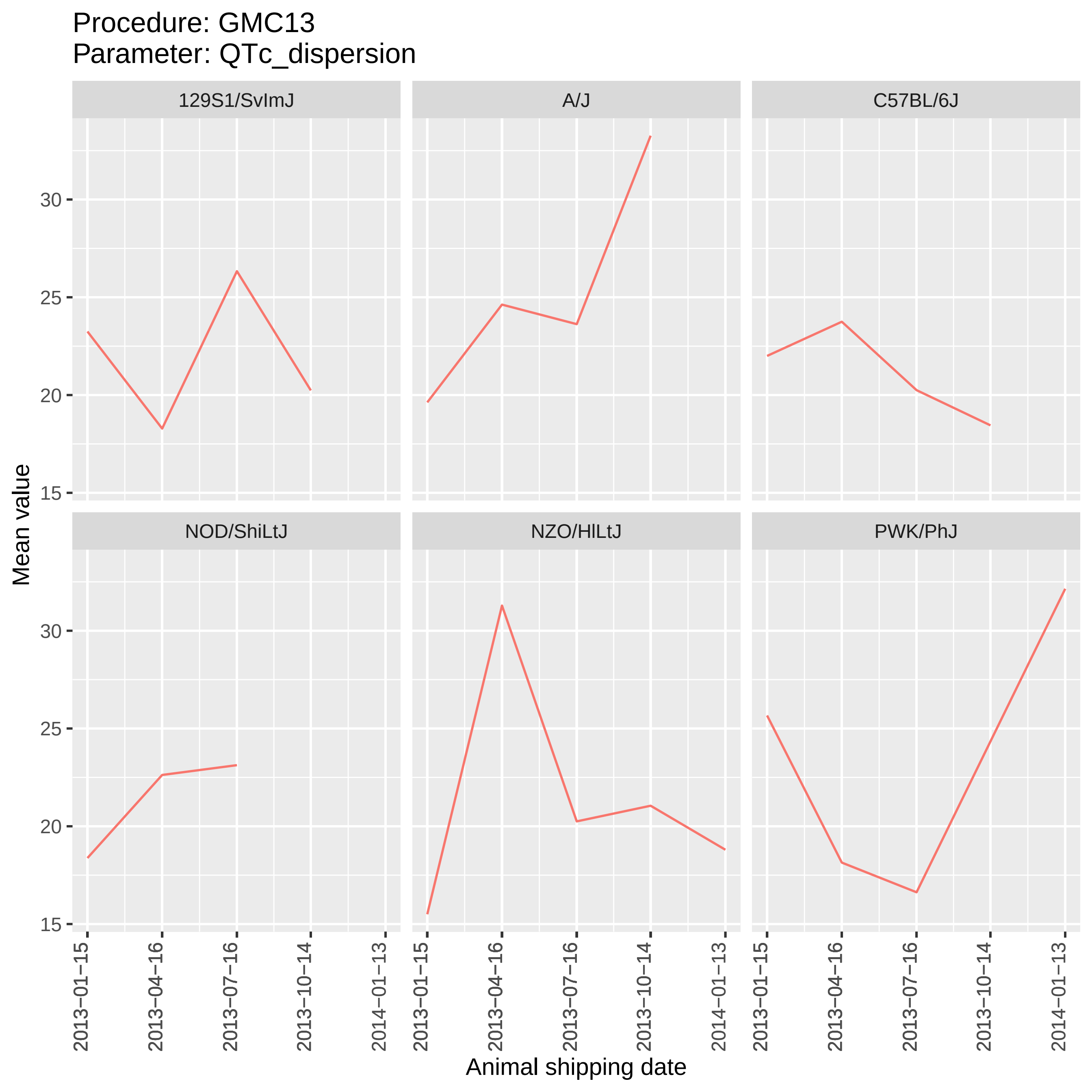

Procedure: GMC13
Parameter: QTc_dispersion
129S1/SvImJ
A/J
C57BL/6J
NOD/ShiLtJ
NZO/HlLtJ
PWK/PhJ
Mean value
15
20
25
30
2013−01−15
2013−04−16
2013−07−16
2013−10−14
2014−01−13
Animal shipping date

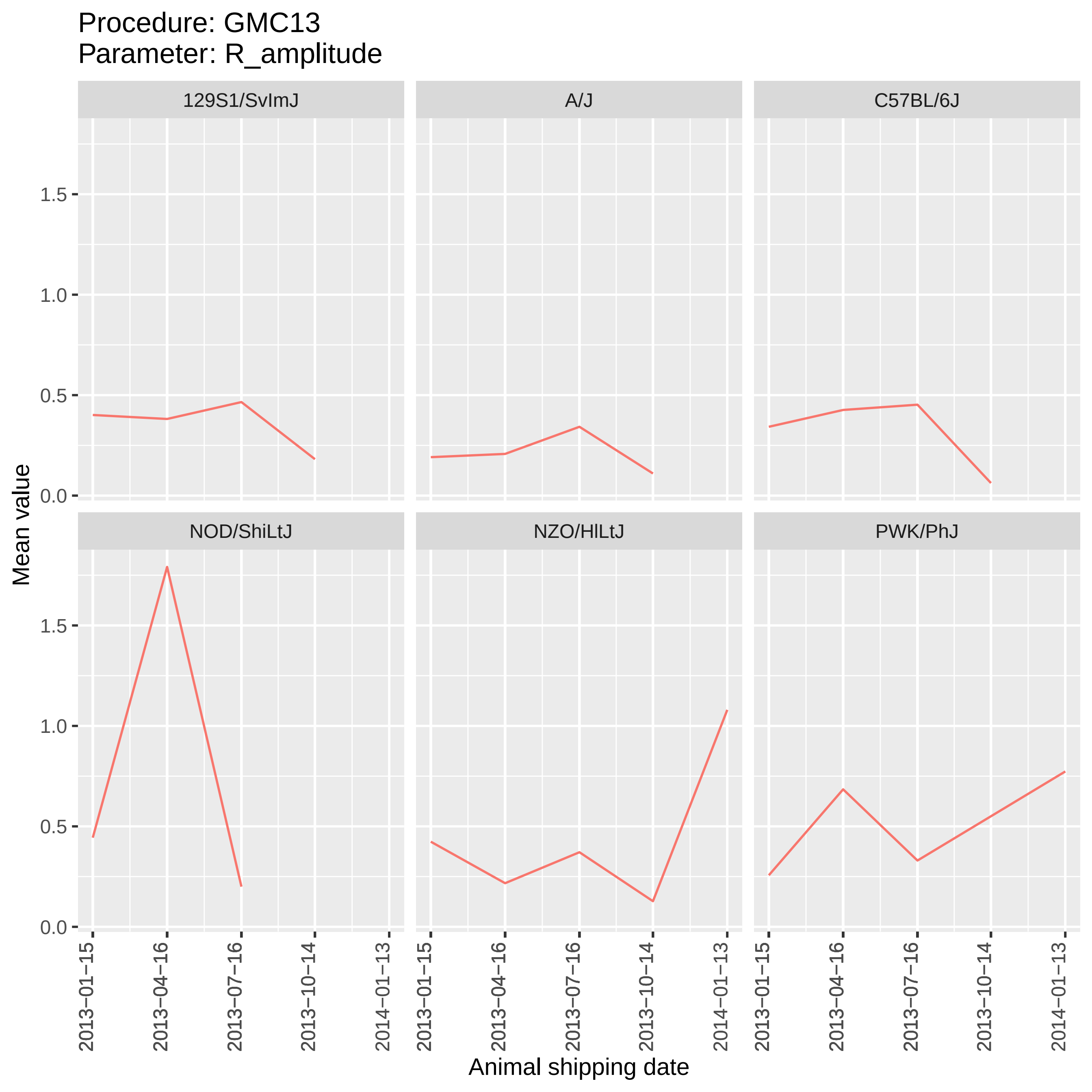
Procedure: GMC13
Parameter: R_amplitude
129S1/SvImJ
A/J
C57BL/6J
NOD/ShiLtJ
NZO/HlLtJ
PWK/PhJ
Mean value
0.0
0.5
1.0
1.5
2013−01−15
2013−04−16
2013−07−16
2013−10−14
2014−01−13
Animal shipping date

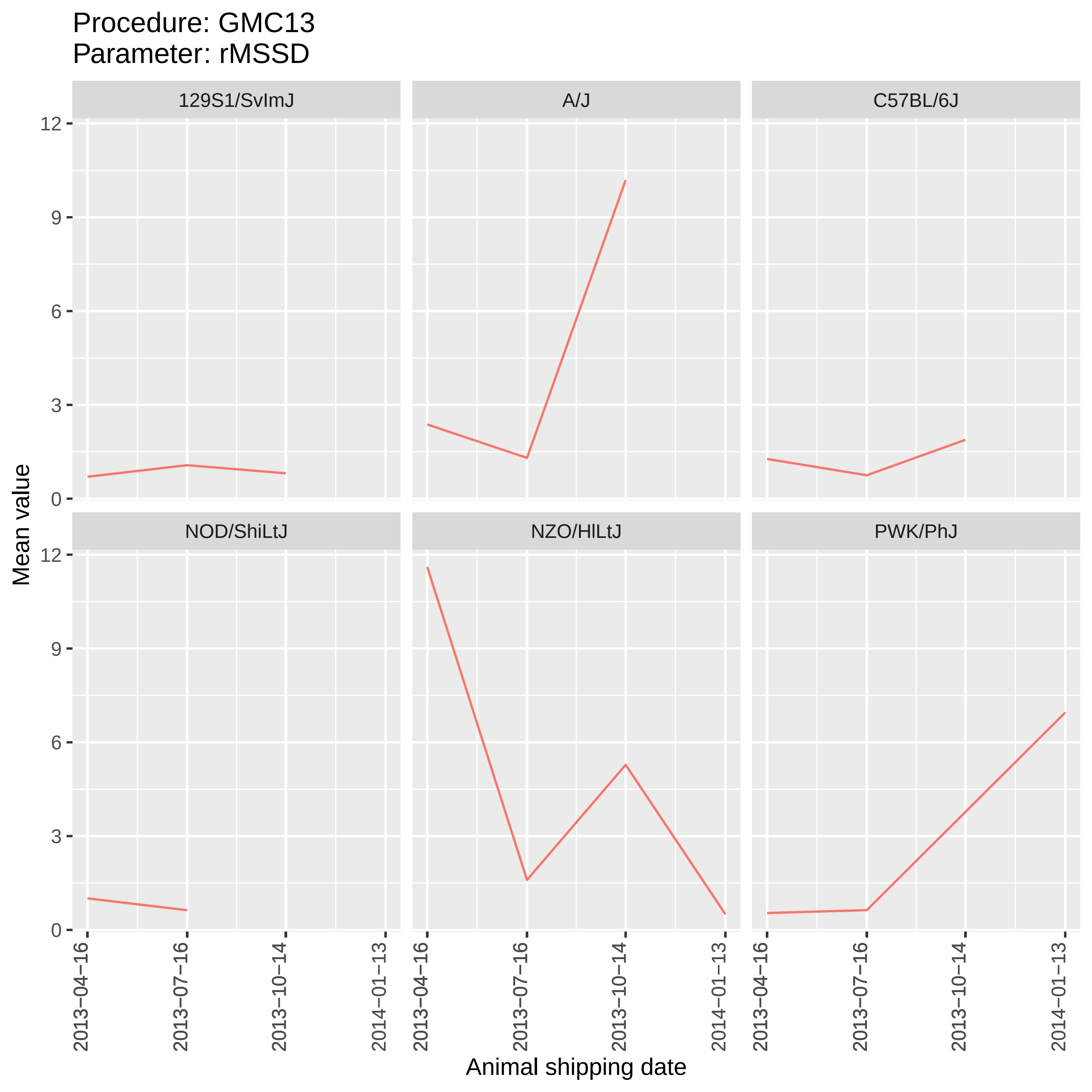

Procedure: GMC13
Parameter: rMSSD
129S1/SvImJ
A/J
C57BL/6J
NOD/ShiLtJ
NZO/HlLtJ
PWK/PhJ
Mean value
0
3
6
9
12
2013−04−16
2013−07−16
2013−10−14
2014−01−13
Animal shipping date

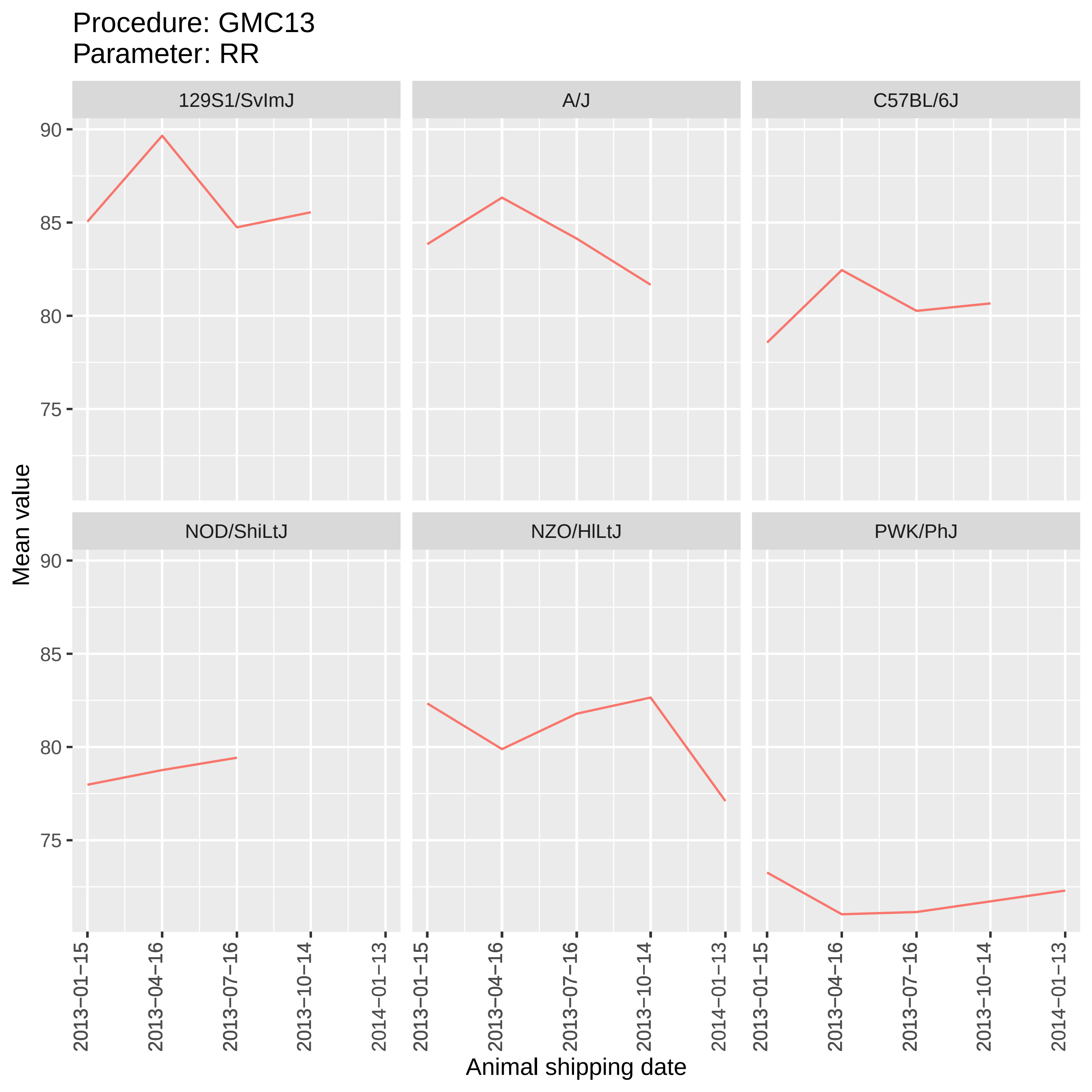
Procedure: GMC13
Parameter: RR
129S1/SvImJ
A/J
C57BL/6J
NOD/ShiLtJ
NZO/HlLtJ
PWK/PhJ
Mean value
90
85
80
75
2013−01−15
2013−04−16
2013−07−16
2013−10−14
2014−01−13
Animal shipping date

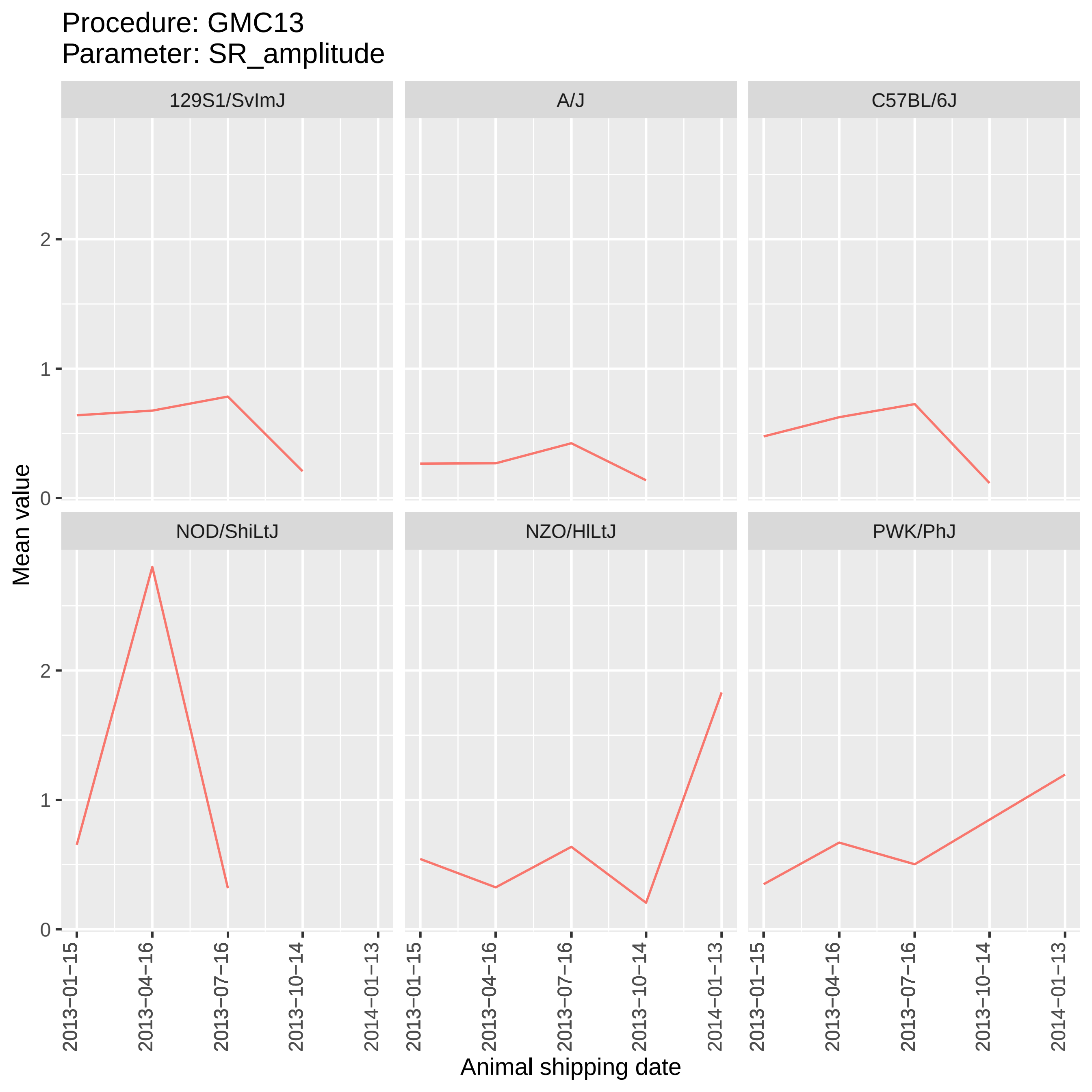

Procedure: GMC13
Parameter: SR_amplitude
129S1/SvImJ
A/J
C57BL/6J
NOD/ShiLtJ
NZO/HlLtJ
PWK/PhJ
Mean value
0
1
2
2013−01−15
2013−04−16
2013−07−16
2013−10−14
2014−01−13
Animal shipping date

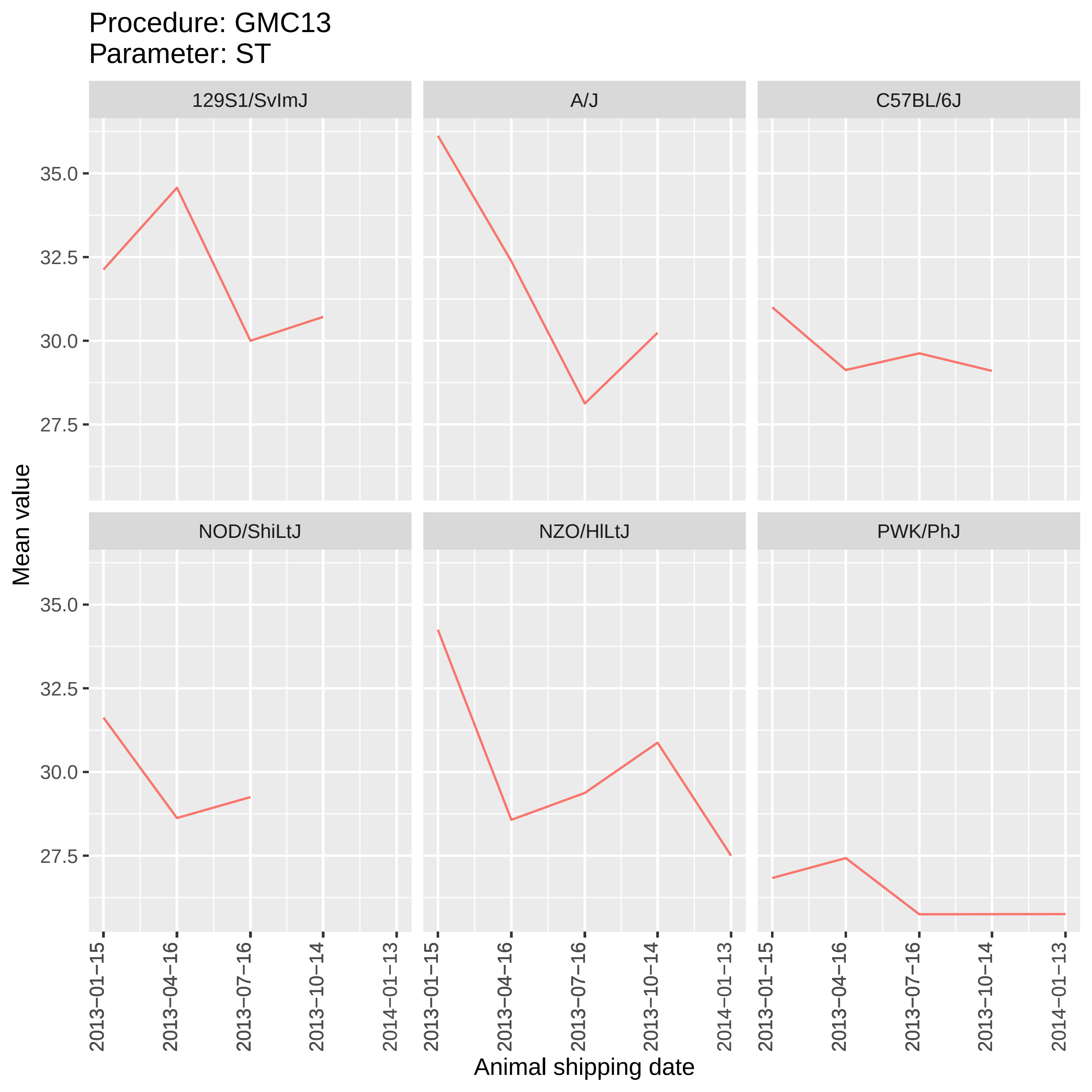

Procedure: GMC13
Parameter: ST
129S1/SvImJ
A/J
C57BL/6J
NOD/ShiLtJ
NZO/HlLtJ
PWK/PhJ
Mean value
35.0
32.5
30.0
27.5
2013−01−15
2013−04−16
2013−07−16
2013−10−14
2014−01−13
Animal shipping date

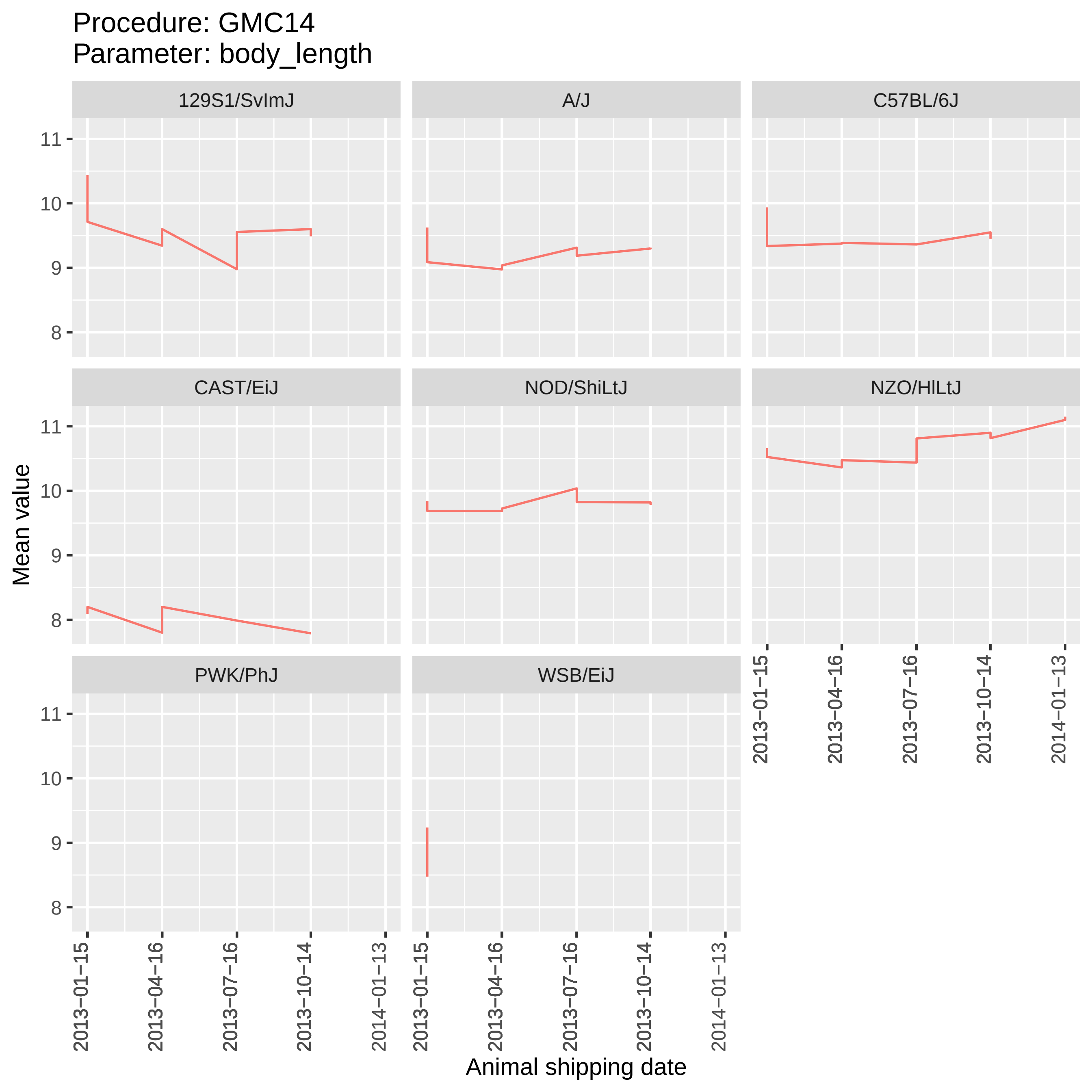

Procedure: GMC14
Parameter: body_length
129S1/SvImJ
A/J
C57BL/6J
CAST/EiJ
NOD/ShiLtJ
NZO/HlLtJ
PWK/PhJ
WSB/EiJ
Mean value
8
9
10
11
2013−01−15
2013−04−16
2013−07−16
2013−10−14
2014−01−13
Animal shipping date

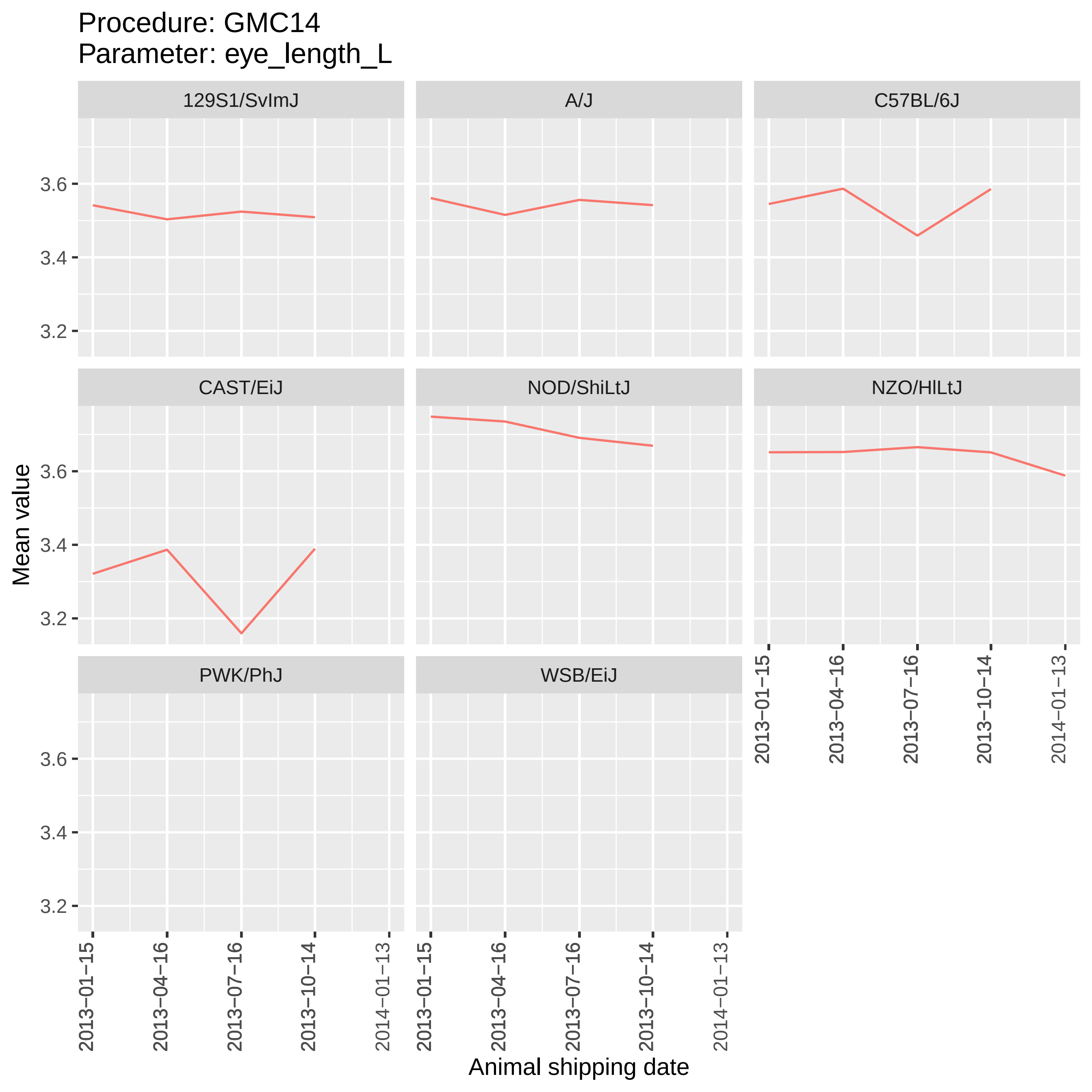

Procedure: GMC14
Parameter: eye_length_L
129S1/SvImJ
A/J
C57BL/6J
CAST/EiJ
NOD/ShiLtJ
NZO/HlLtJ
PWK/PhJ
WSB/EiJ
Mean value
3.2
3.4
3.6
2013−01−15
2013−04−16
2013−07−16
2013−10−14
2014−01−13
Animal shipping date

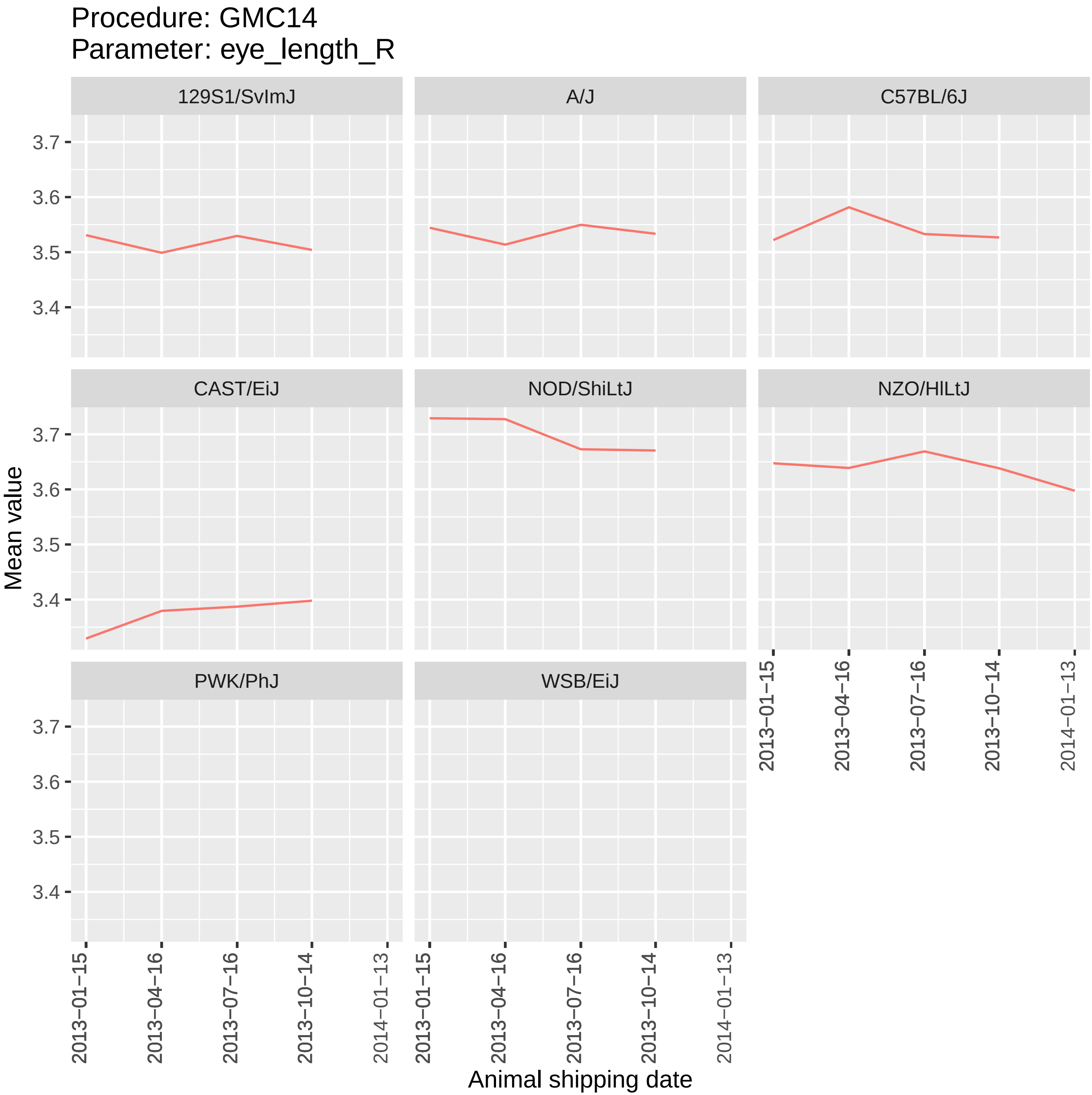
Procedure: GMC14
Parameter: eye_length_R
129S1/SvImJ
A/J
C57BL/6J
CAST/EiJ
NOD/ShiLtJ
NZO/HlLtJ
PWK/PhJ
WSB/EiJ
Mean value
3.4
3.5
3.6
3.7
2013−01−15
2013−04−16
2013−07−16
2013−10−14
2014−01−13
Animal shipping date

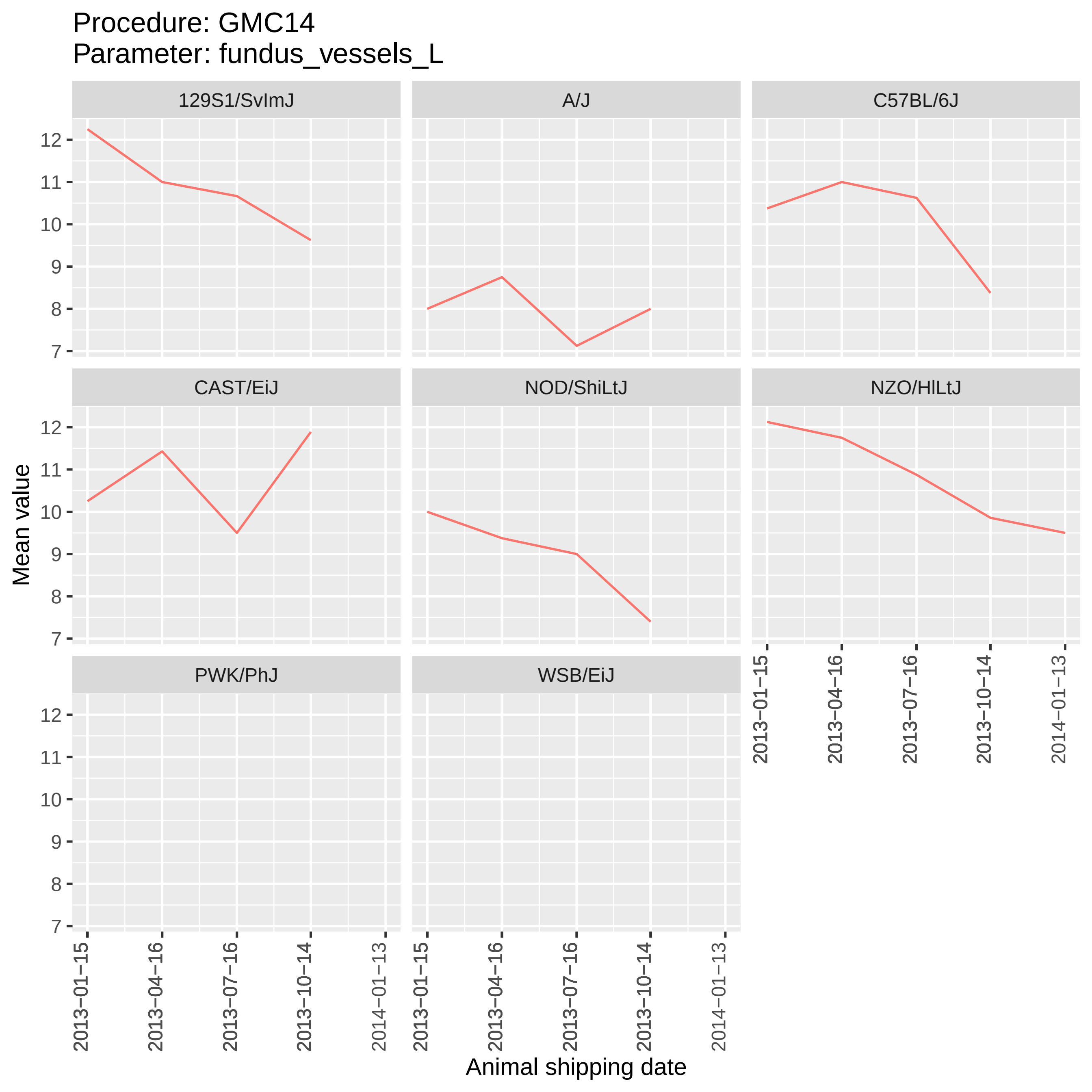

Procedure: GMC14
Parameter: fundus_vessels_L
129S1/SvImJ
A/J
C57BL/6J
CAST/EiJ
NOD/ShiLtJ
NZO/HlLtJ
PWK/PhJ
WSB/EiJ
Mean value
12
11
10
9
8
7
2013−01−15
2013−04−16
2013−07−16
2013−10−14
2014−01−13
Animal shipping date

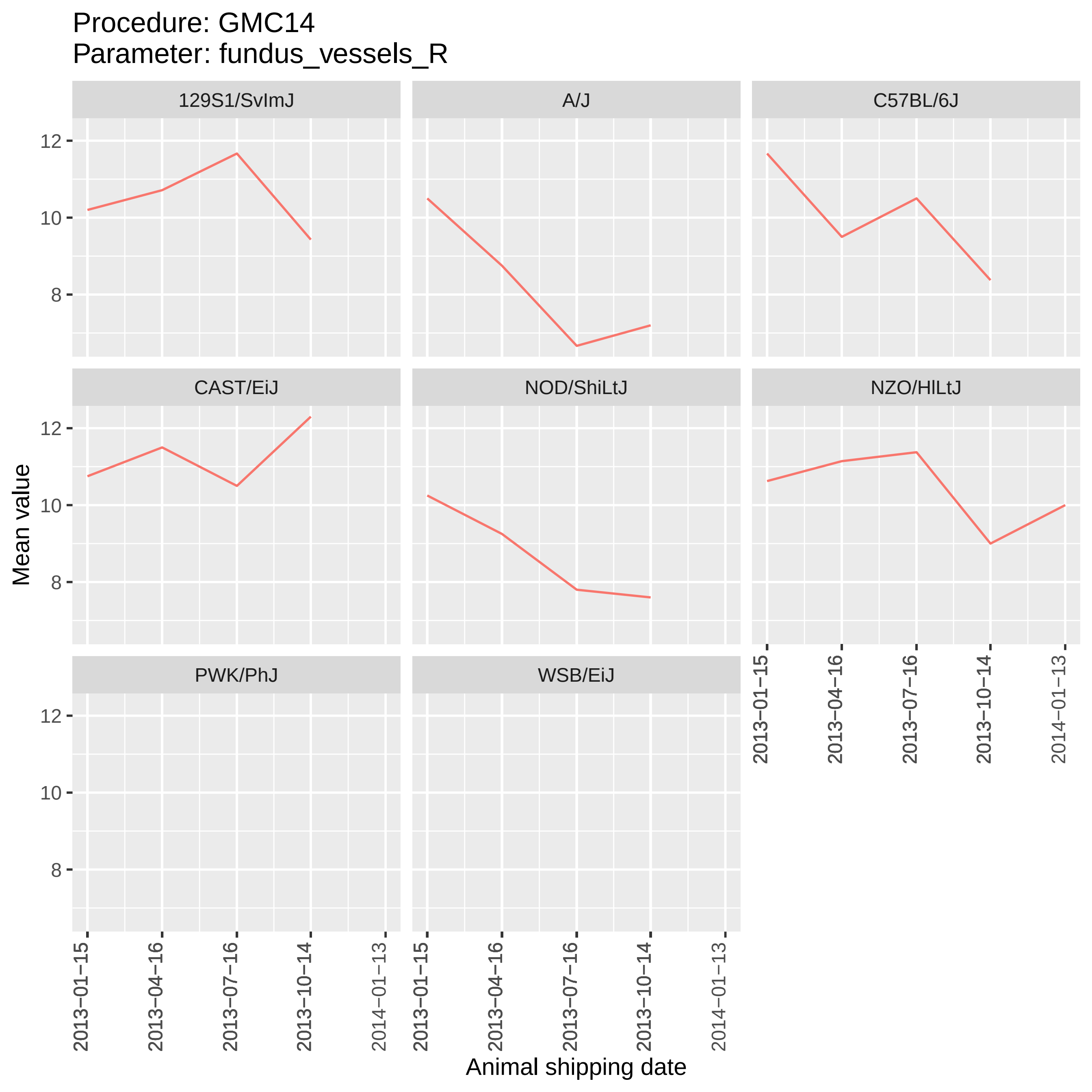

Procedure: GMC14
Parameter: fundus_vessels_R
129S1/SvImJ
A/J
C57BL/6J
CAST/EiJ
NOD/ShiLtJ
NZO/HlLtJ
PWK/PhJ
WSB/EiJ
12
10
8
Mean value
2013−01−15
2013−04−16
2013−07−16
2013−10−14
2014−01−13
Animal shipping date

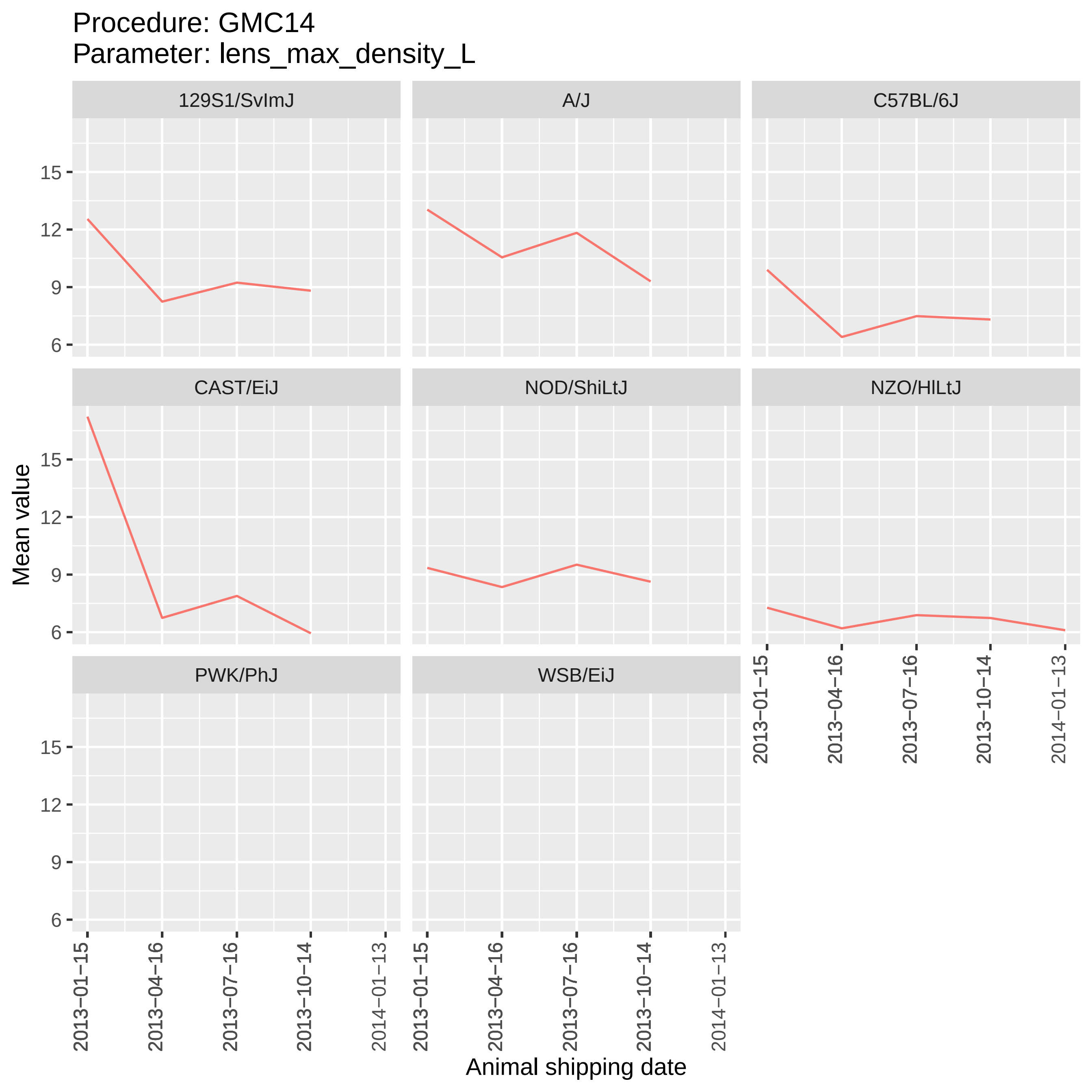
Procedure: GMC14
Parameter: lens_max_density_L
129S1/SvImJ
A/J
C57BL/6J
CAST/EiJ
NOD/ShiLtJ
NZO/HlLtJ
PWK/PhJ
WSB/EiJ
Mean value
15
12
9
6
2013−01−15
2013−04−16
2013−07−16
2013−10−14
2014−01−13
Animal shipping date

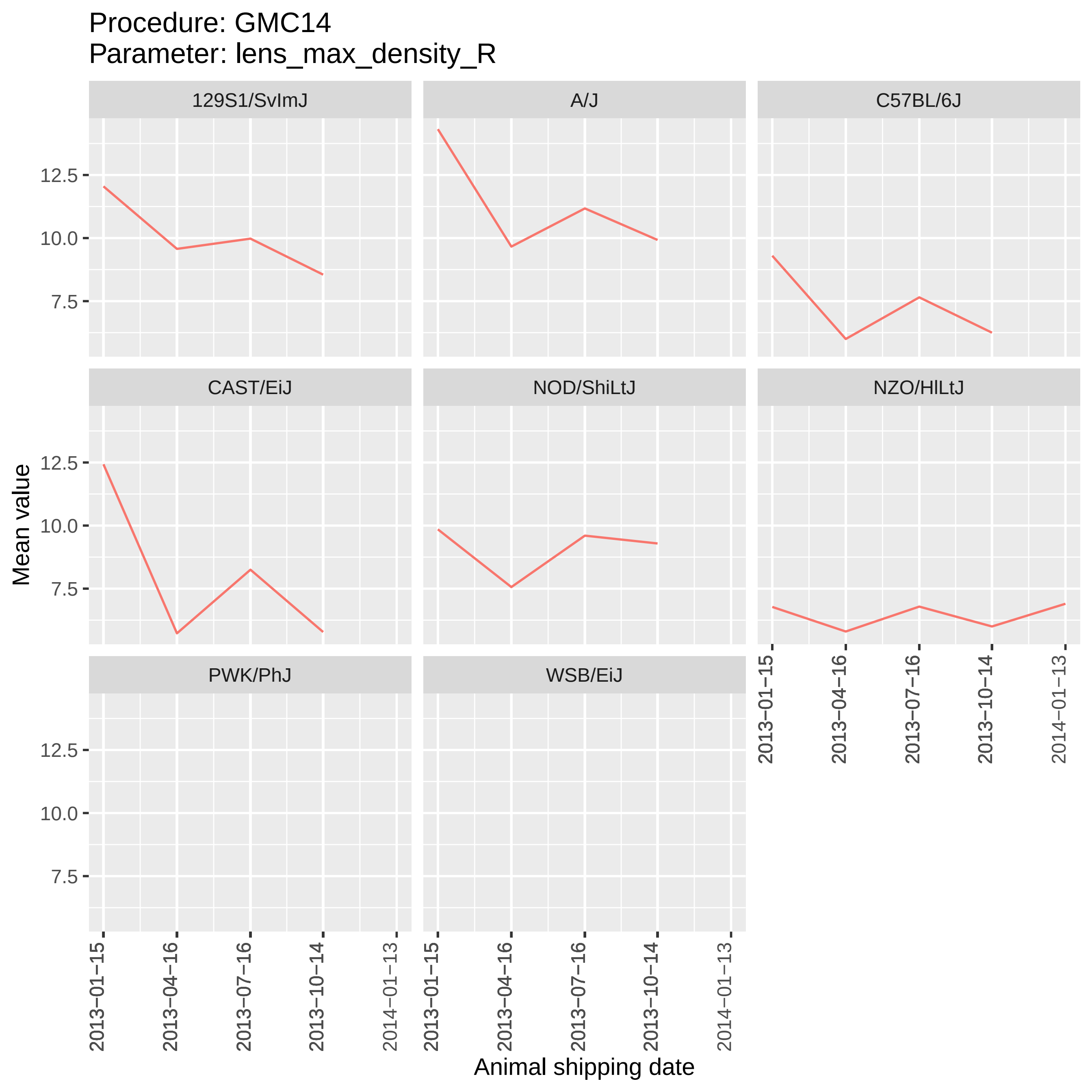
Procedure: GMC14
Parameter: lens_max_density_R
129S1/SvImJ
A/J
C57BL/6J
CAST/EiJ
NOD/ShiLtJ
NZO/HlLtJ
PWK/PhJ
WSB/EiJ
Mean value
12.5
10.0
7.5
2013−01−15
2013−04−16
2013−07−16
2013−10−14
2014−01−13
Animal shipping date

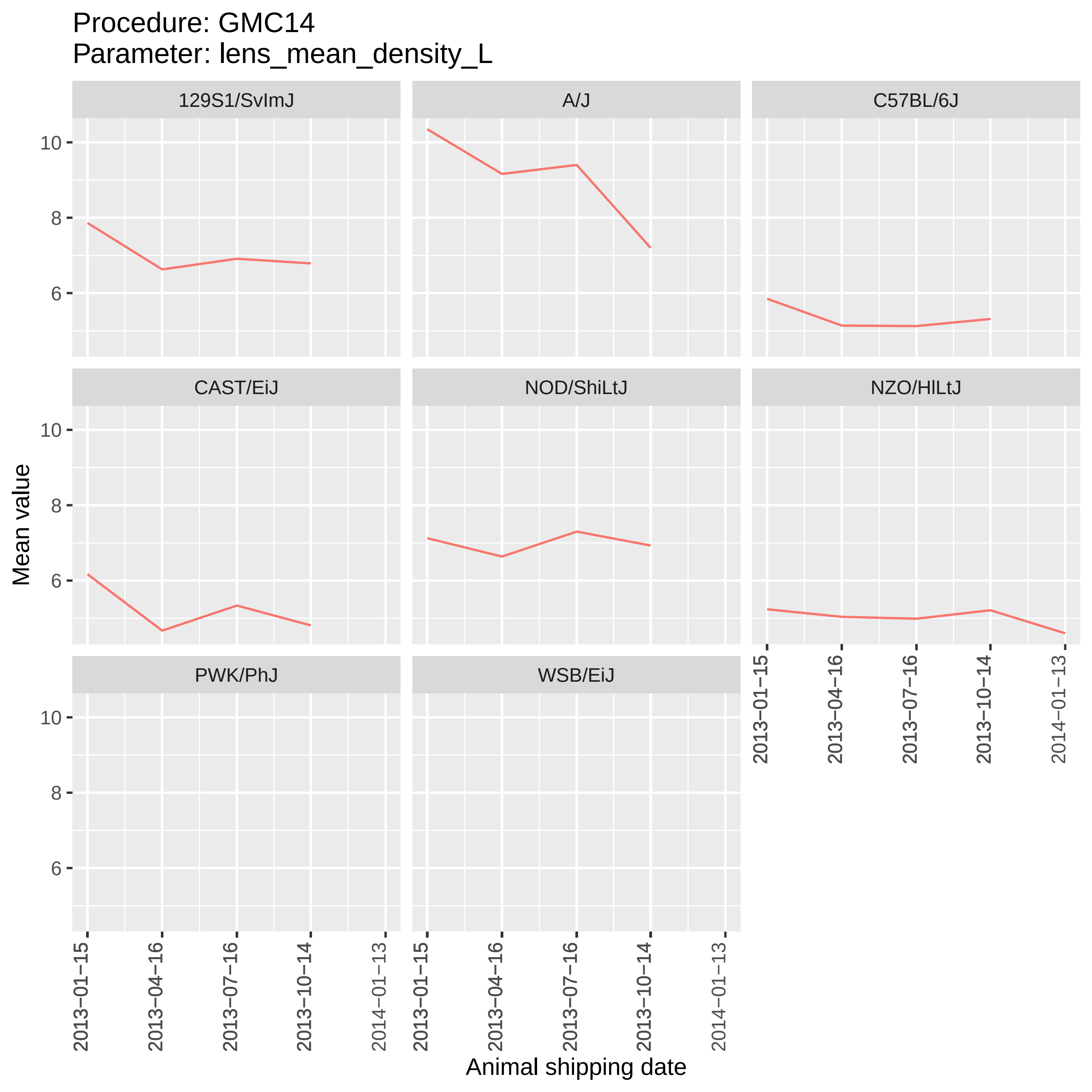

Procedure: GMC14
Parameter: lens_mean_density_L
129S1/SvImJ
A/J
C57BL/6J
CAST/EiJ
NOD/ShiLtJ
NZO/HlLtJ
PWK/PhJ
WSB/EiJ
Mean value
10
8
6
2013−01−15
2013−04−16
2013−07−16
2013−10−14
2014−01−13
Animal shipping date

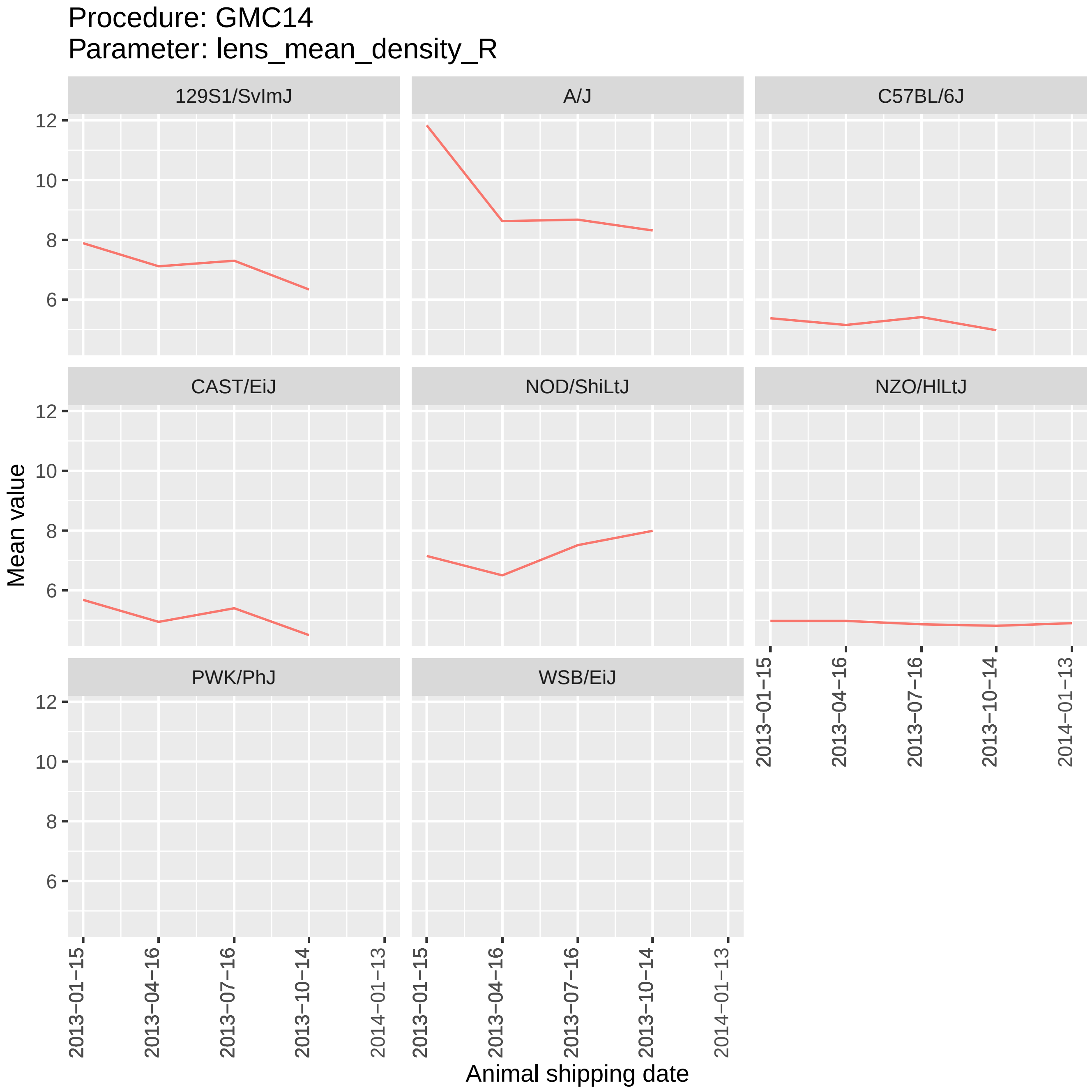

Procedure: GMC14
Parameter: lens_mean_density_R
129S1/SvImJ
A/J
C57BL/6J
CAST/EiJ
NOD/ShiLtJ
NZO/HlLtJ
PWK/PhJ
WSB/EiJ
Mean value
Animal shipping date
12
10
8
6
2013−01−15
2013−04−16
2013−07−16
2013−10−14
2014−01−13

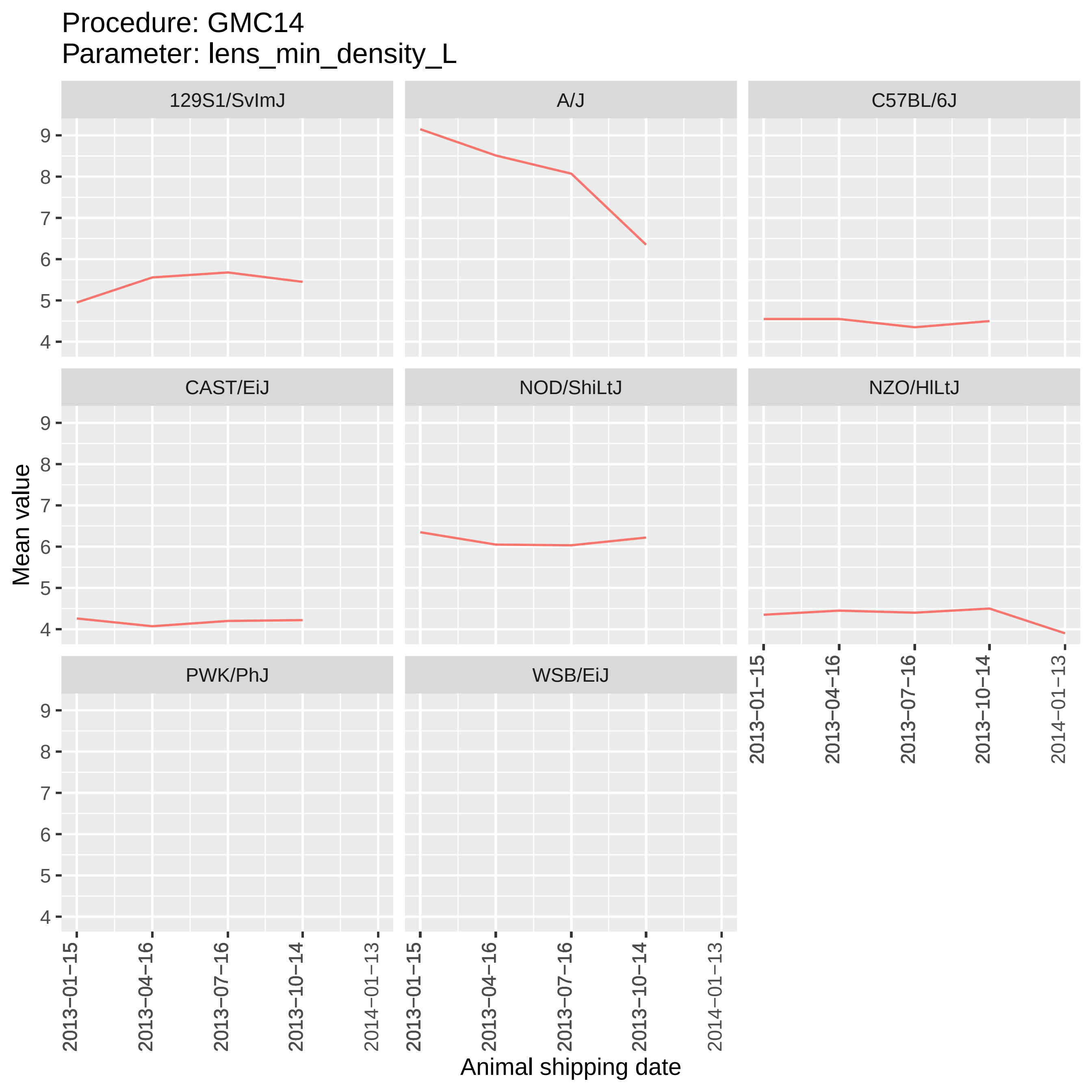

Procedure: GMC14
Parameter: lens_min_density_L
129S1/SvImJ
A/J
C57BL/6J
CAST/EiJ
NOD/ShiLtJ
NZO/HlLtJ
PWK/PhJ
WSB/EiJ
Mean value
9
8
7
6
5
4
2013−01−15
2013−04−16
2013−07−16
2013−10−14
2014−01−13
Animal shipping date

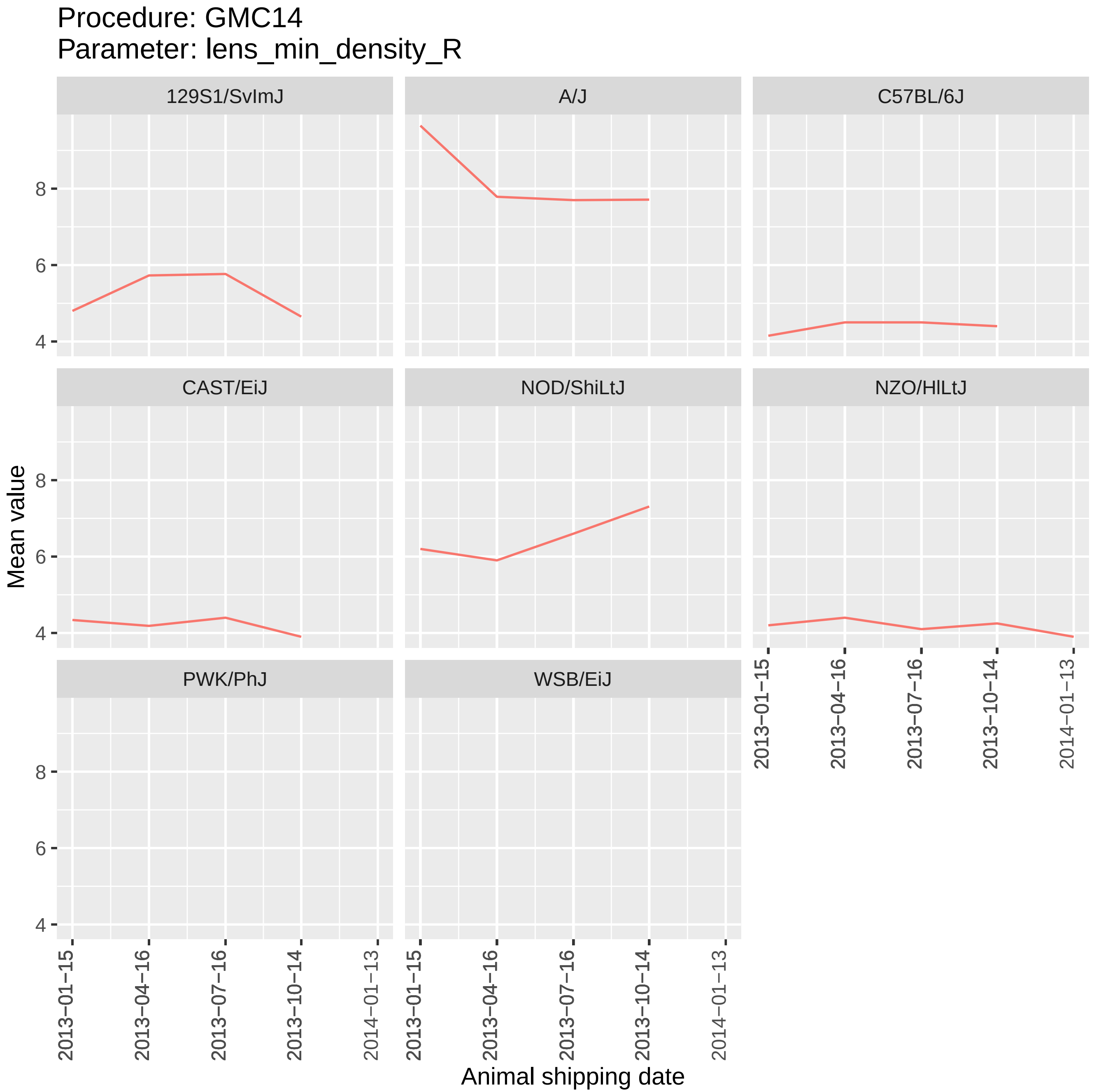

Procedure: GMC14
Parameter: lens_min_density_R
129S1/SvImJ
A/J
C57BL/6J
CAST/EiJ
NOD/ShiLtJ
NZO/HlLtJ
PWK/PhJ
WSB/EiJ
8
6
4
Mean value
2013−01−15
2013−04−16
2013−07−16
2013−10−14
2014−01−13
Animal shipping date

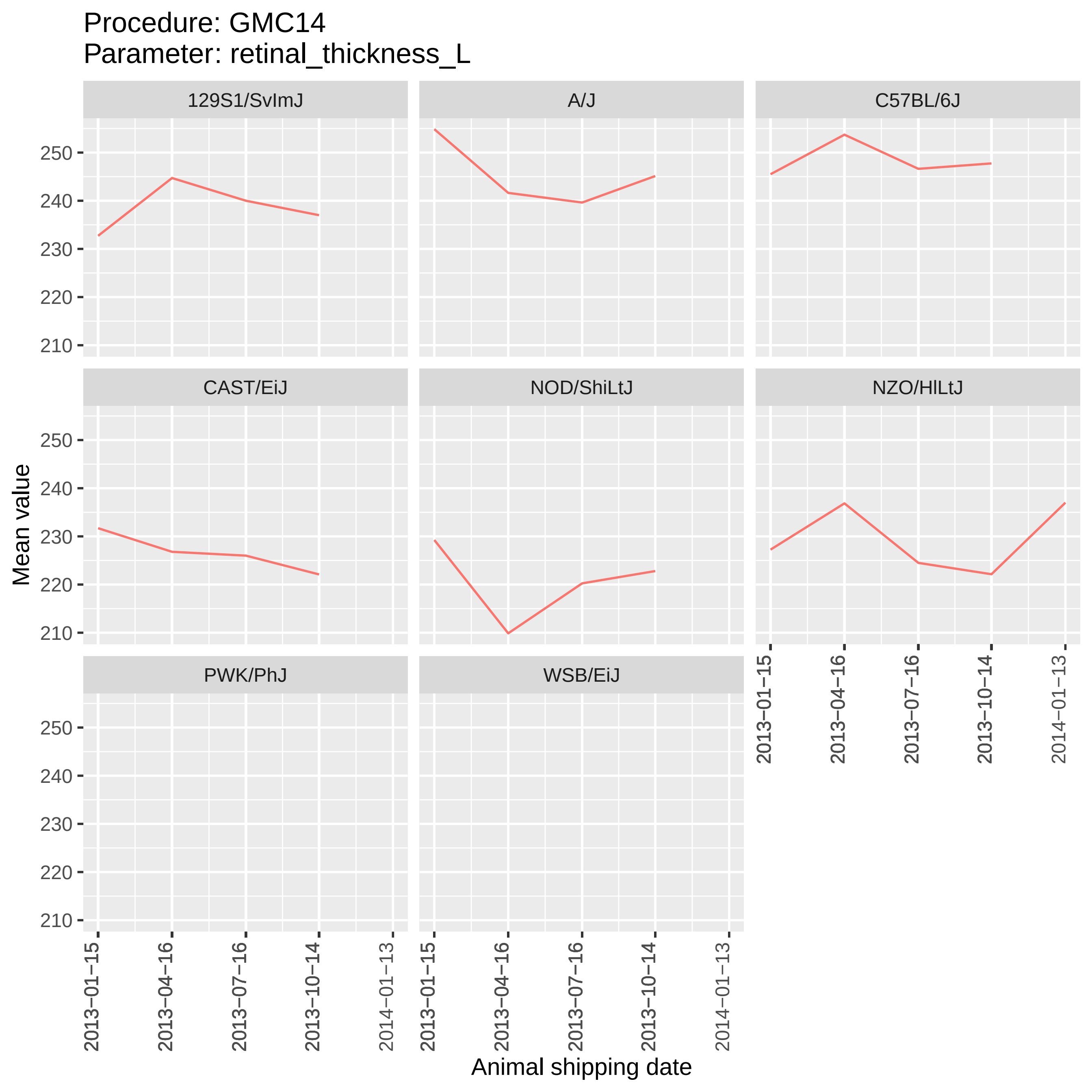

Procedure: GMC14
Parameter: retinal_thickness_L
129S1/SvImJ
A/J
C57BL/6J
CAST/EiJ
NOD/ShiLtJ
NZO/HlLtJ
PWK/PhJ
WSB/EiJ
Mean value
210
220
230
240
250
2013−01−15
2013−04−16
2013−07−16
2013−10−14
2014−01−13
Animal shipping date

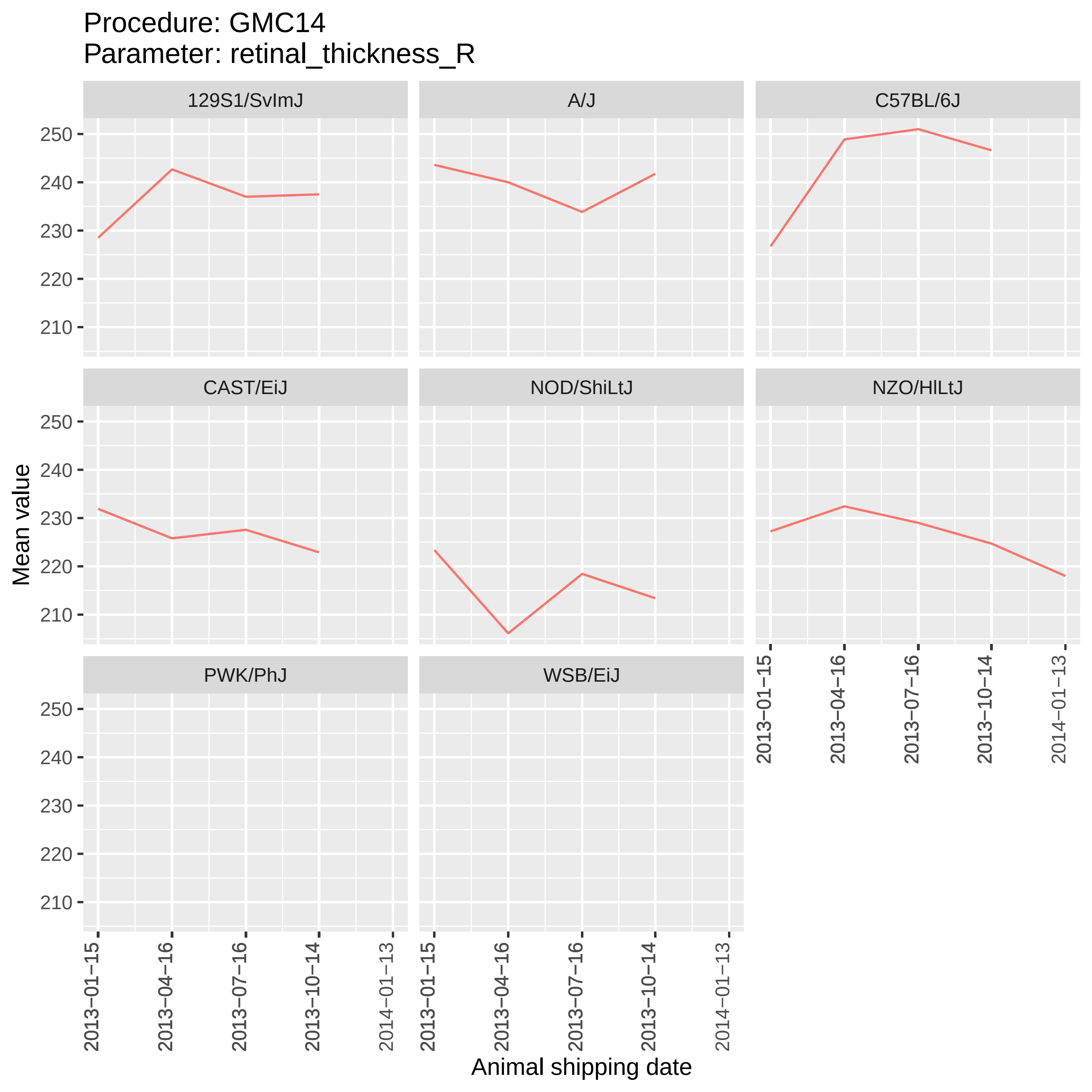

Procedure: GMC14
Parameter: retinal_thickness_R
129S1/SvImJ
A/J
C57BL/6J
CAST/EiJ
NOD/ShiLtJ
NZO/HlLtJ
PWK/PhJ
WSB/EiJ
Mean value
210
220
230
240
250
2013−01−15
2013−04−16
2013−07−16
2013−10−14
2014−01−13
Animal shipping date

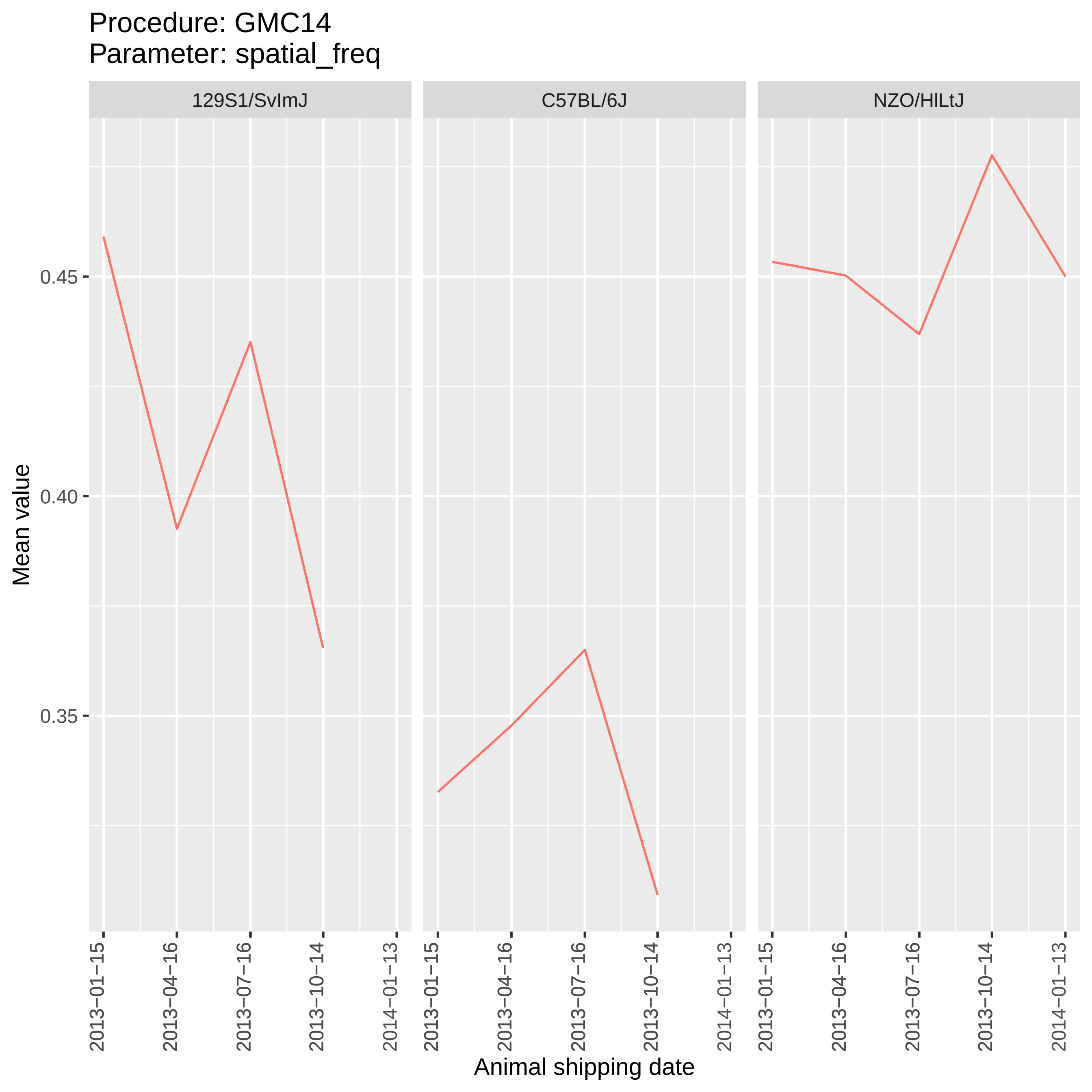
Procedure: GMC14
Parameter: spatial_freq
129S1/SvImJ
C57BL/6J
NZO/HlLtJ
Mean value
0.45
0.40
0.35
2013−01−15
2013−04−16
2013−07−16
2013−10−14
2014−01−13
Animal shipping date

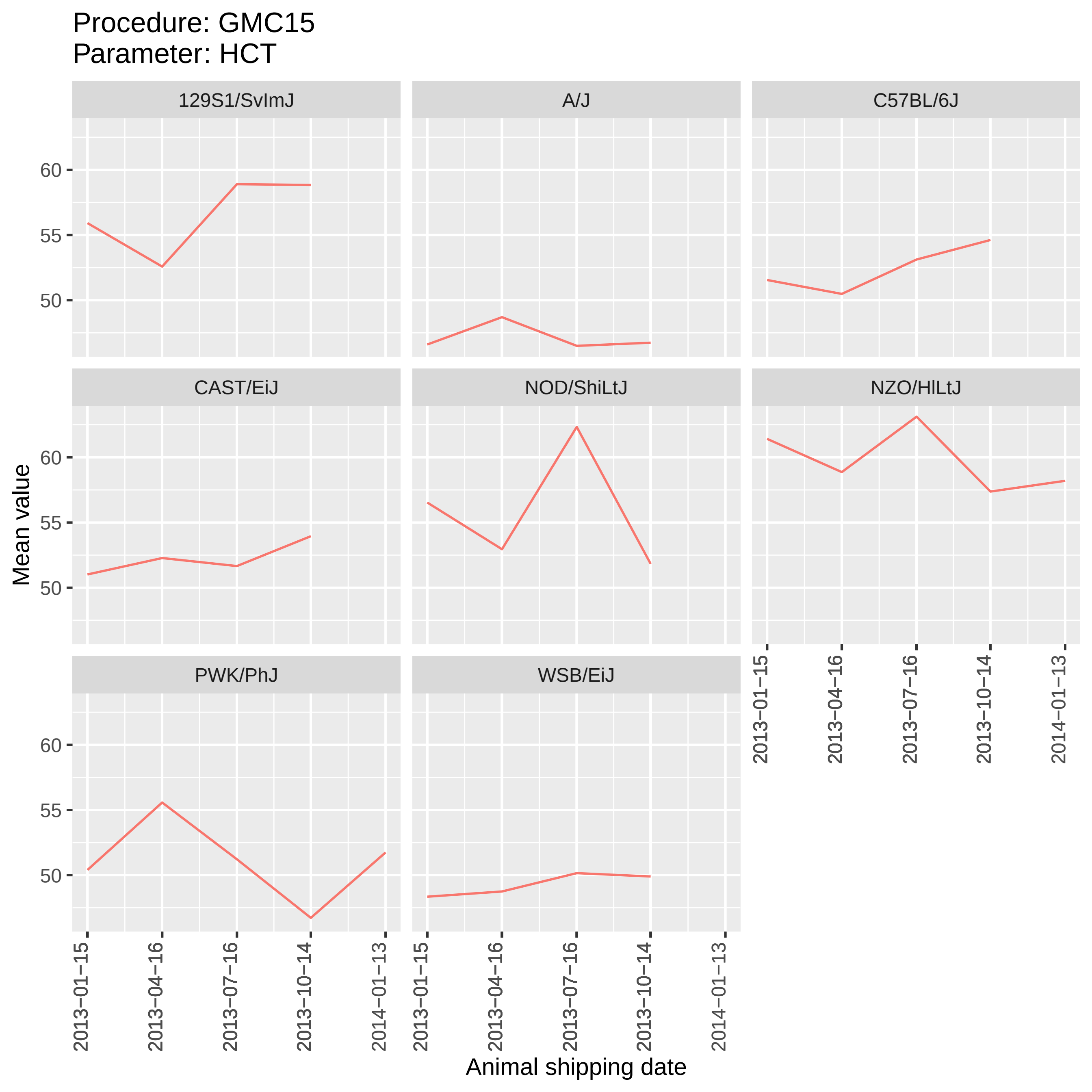
Procedure: GMC15
Parameter: HCT
129S1/SvImJ
A/J
C57BL/6J
CAST/EiJ
NOD/ShiLtJ
NZO/HlLtJ
PWK/PhJ
WSB/EiJ
Mean value
60
55
50
2013−01−15
2013−04−16
2013−07−16
2013−10−14
2014−01−13
Animal shipping date

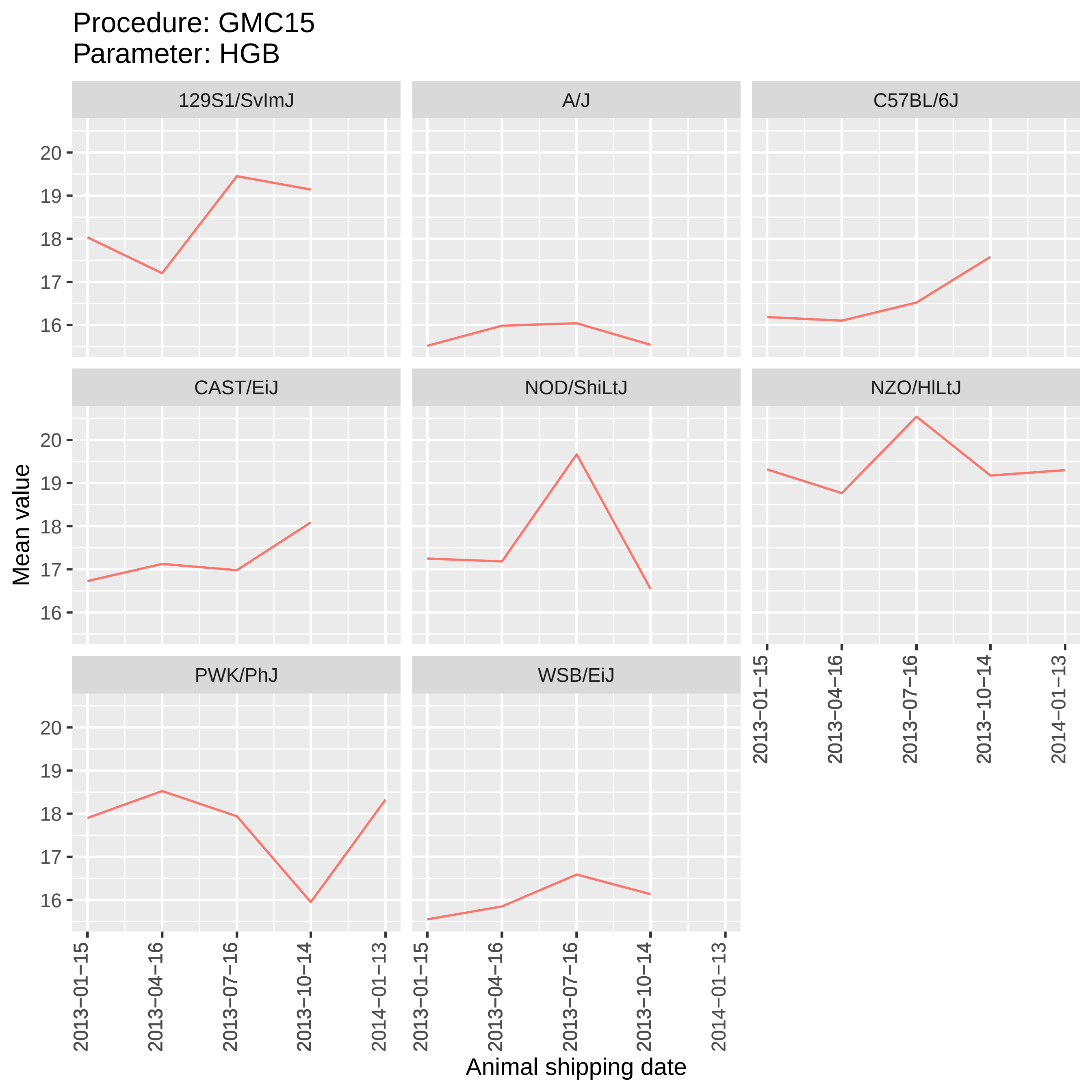

Procedure: GMC15
Parameter: HGB
129S1/SvImJ
A/J
C57BL/6J
CAST/EiJ
NOD/ShiLtJ
NZO/HlLtJ
PWK/PhJ
WSB/EiJ
Mean value
16
17
18
19
20
2013−01−15
2013−04−16
2013−07−16
2013−10−14
2014−01−13
Animal shipping date

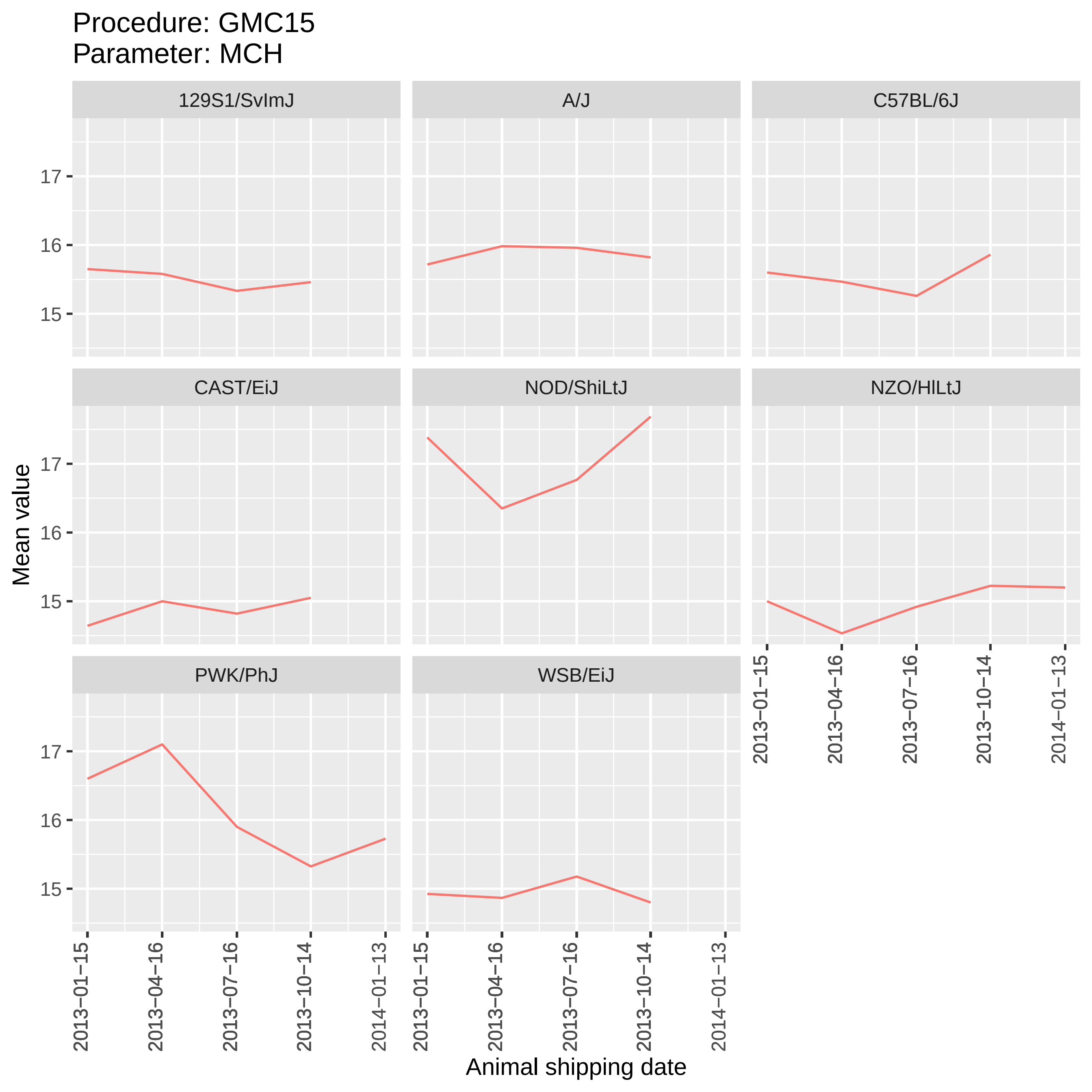

Procedure: GMC15
Parameter: MCH
129S1/SvImJ
A/J
C57BL/6J
CAST/EiJ
NOD/ShiLtJ
NZO/HlLtJ
PWK/PhJ
WSB/EiJ
Mean value
15
16
17
2013−01−15
2013−04−16
2013−07−16
2013−10−14
2014−01−13
Animal shipping date

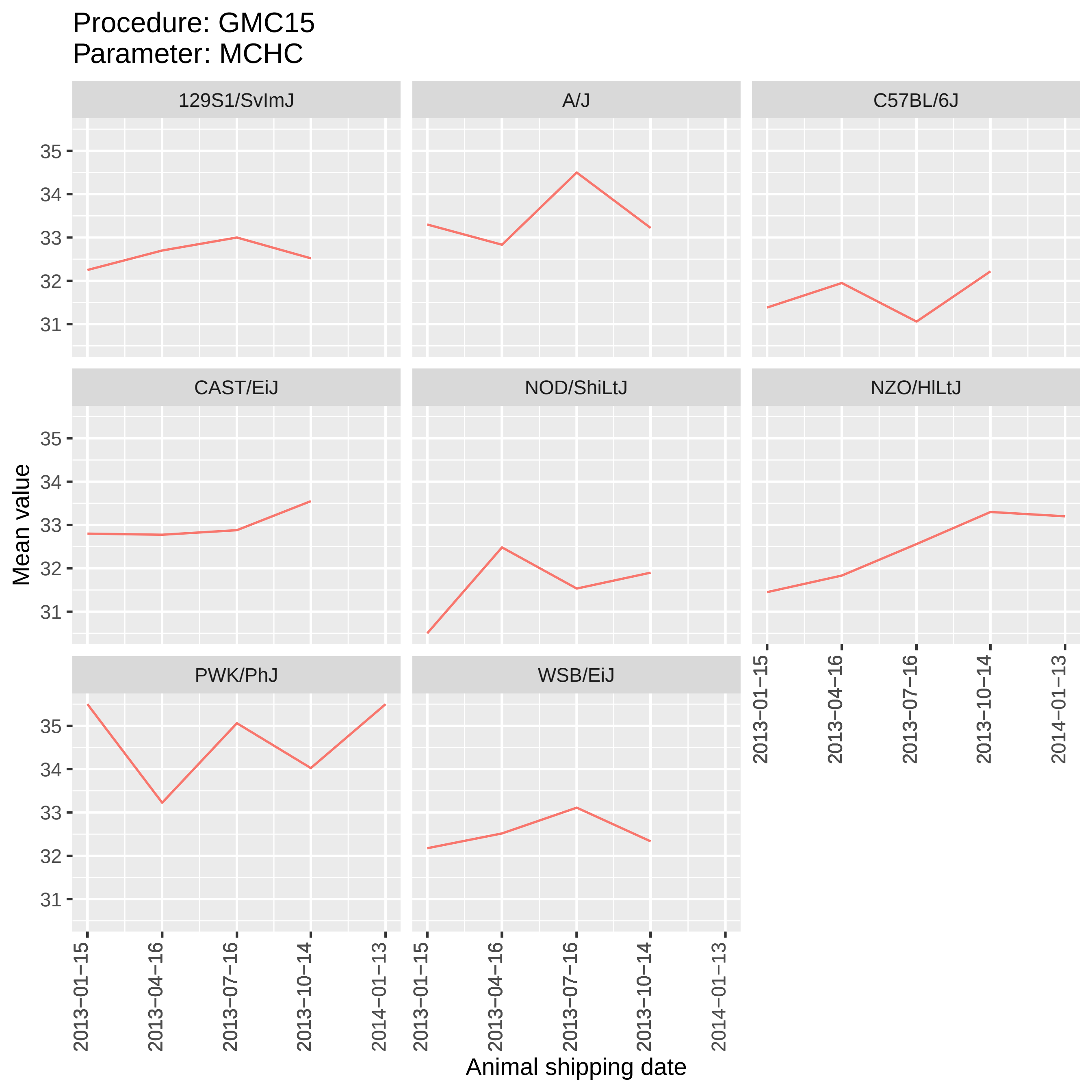

Procedure: GMC15
Parameter: MCHC
129S1/SvImJ
A/J
C57BL/6J
CAST/EiJ
NOD/ShiLtJ
NZO/HlLtJ
PWK/PhJ
WSB/EiJ
Mean value
31
32
33
34
35
2013−01−15
2013−04−16
2013−07−16
2013−10−14
2014−01−13
Animal shipping date

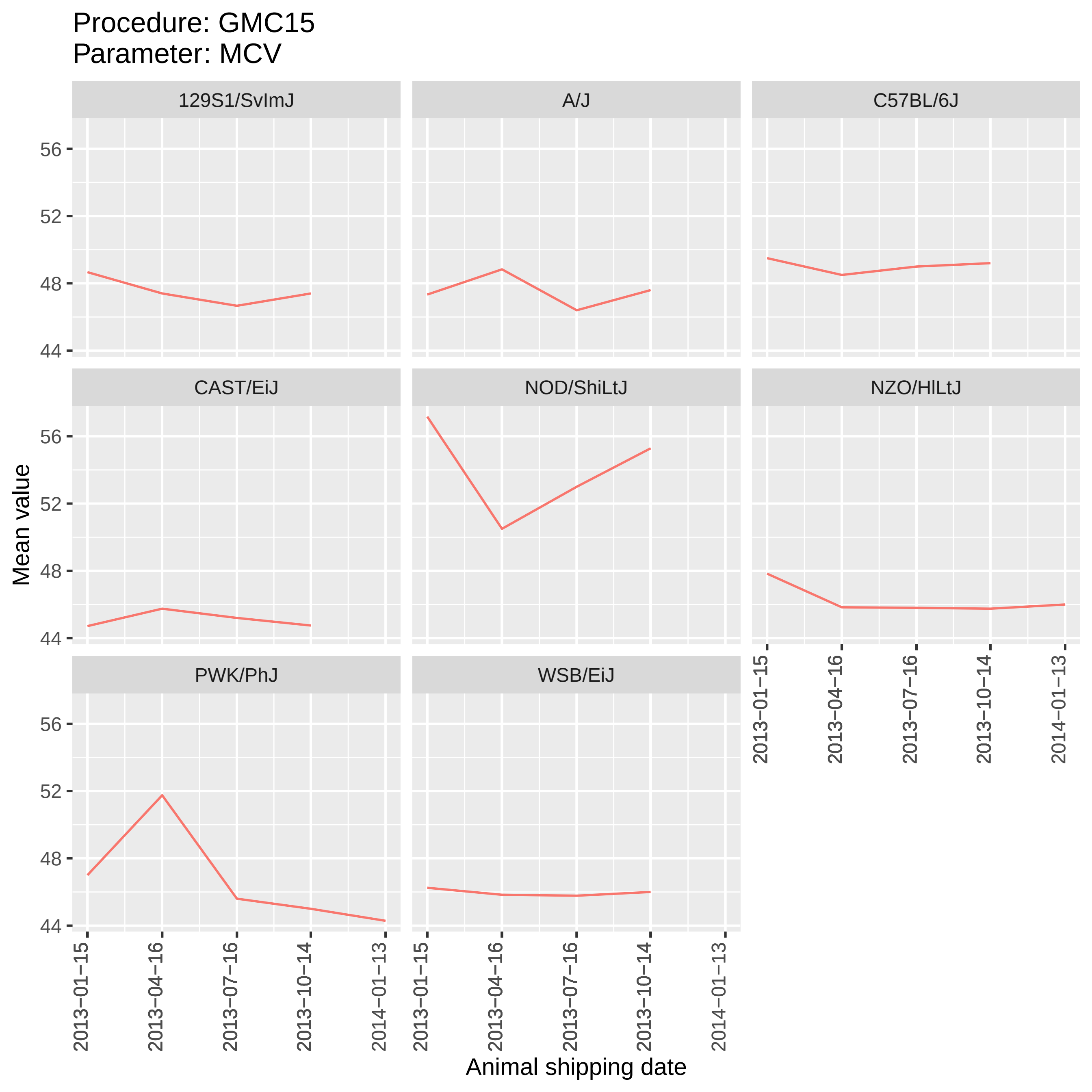

Procedure: GMC15
Parameter: MCV
129S1/SvImJ
A/J
C57BL/6J
CAST/EiJ
NOD/ShiLtJ
NZO/HlLtJ
PWK/PhJ
WSB/EiJ
Mean value
56
52
48
44
2013−01−15
2013−04−16
2013−07−16
2013−10−14
2014−01−13
Animal shipping date

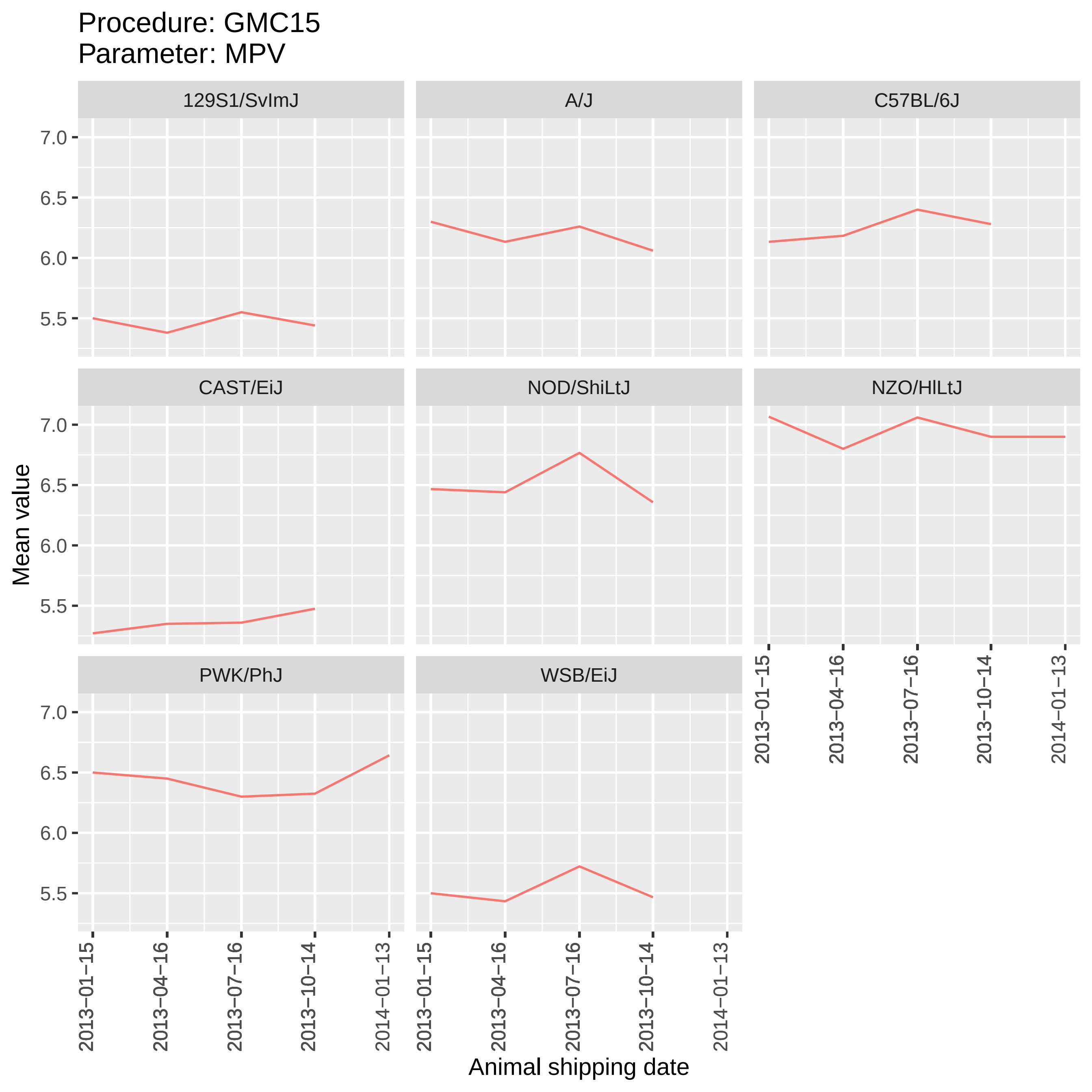

Procedure: GMC15
Parameter: MPV
129S1/SvImJ
A/J
C57BL/6J
CAST/EiJ
NOD/ShiLtJ
NZO/HlLtJ
PWK/PhJ
WSB/EiJ
7.0
6.5
6.0
5.5
Mean value
2013−01−15
2013−04−16
2013−07−16
2013−10−14
2014−01−13
Animal shipping date

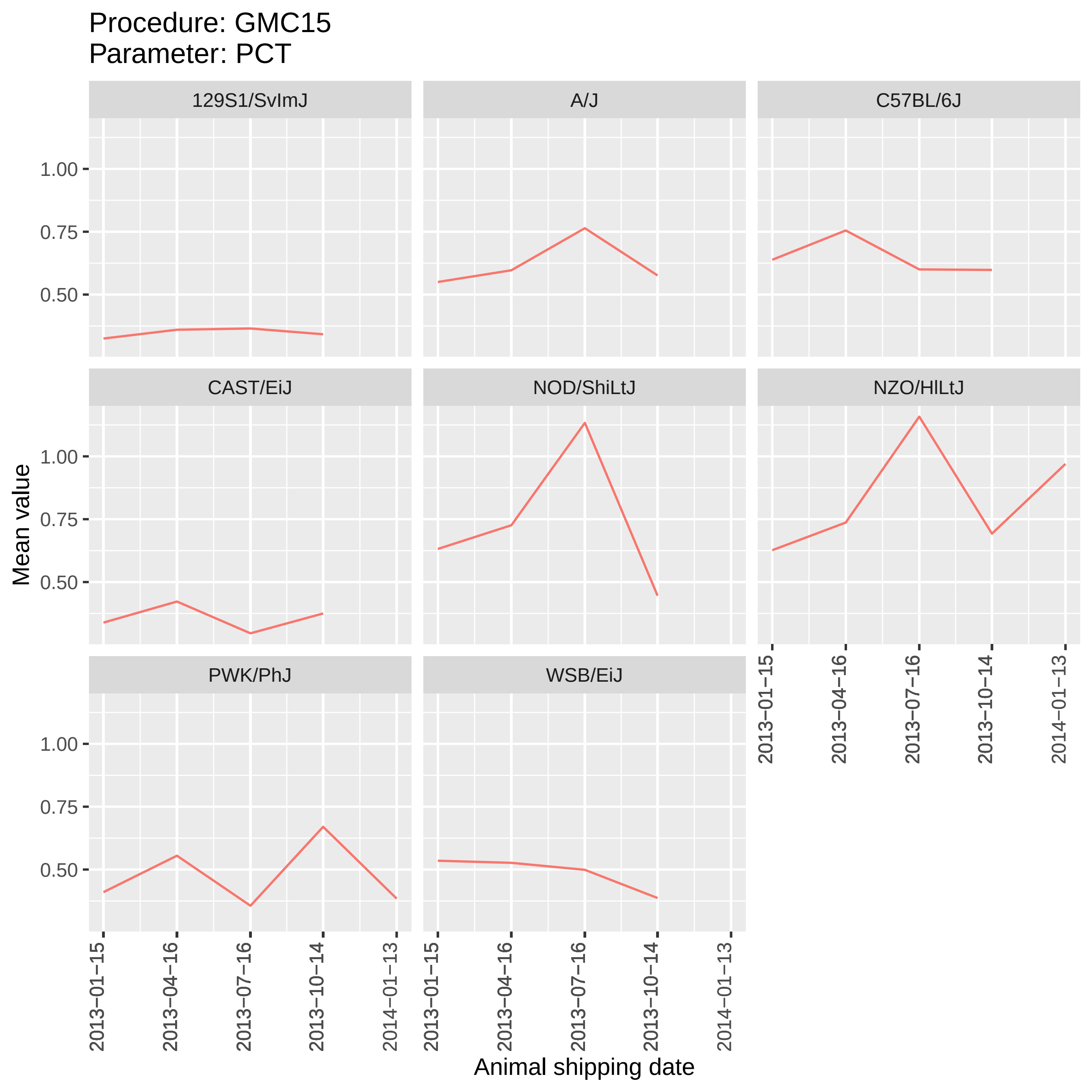
Procedure: GMC15
Parameter: PCT
129S1/SvImJ
A/J
C57BL/6J
CAST/EiJ
NOD/ShiLtJ
NZO/HlLtJ
PWK/PhJ
WSB/EiJ
Mean value
1.00
0.75
0.50
2013−01−15
2013−04−16
2013−07−16
2013−10−14
2014−01−13
Animal shipping date

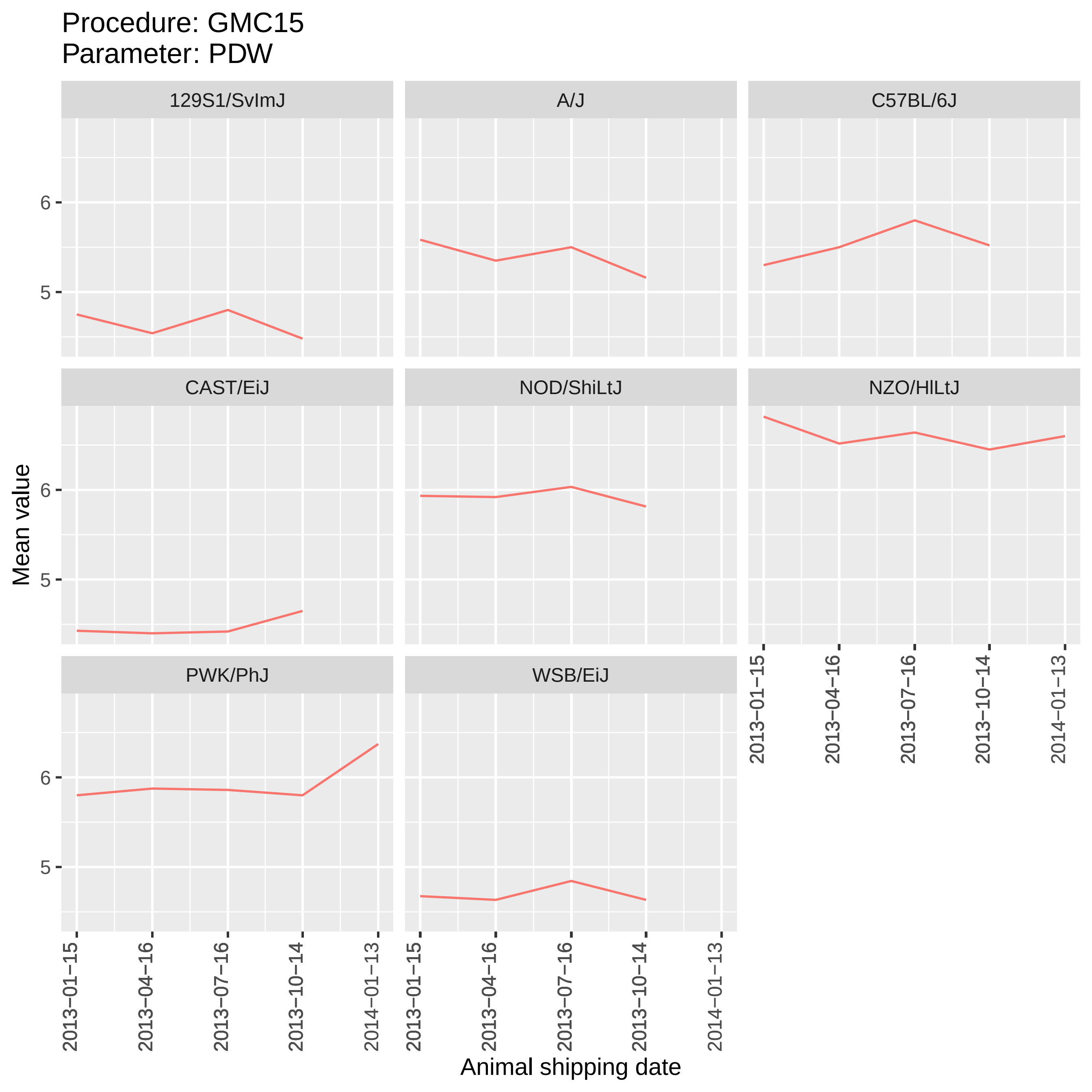
Procedure: GMC15
Parameter: PDW
129S1/SvImJ
A/J
C57BL/6J
CAST/EiJ
NOD/ShiLtJ
NZO/HlLtJ
PWK/PhJ
WSB/EiJ
Mean value
6
5
2013−01−15
2013−04−16
2013−07−16
2013−10−14
2014−01−13
Animal shipping date

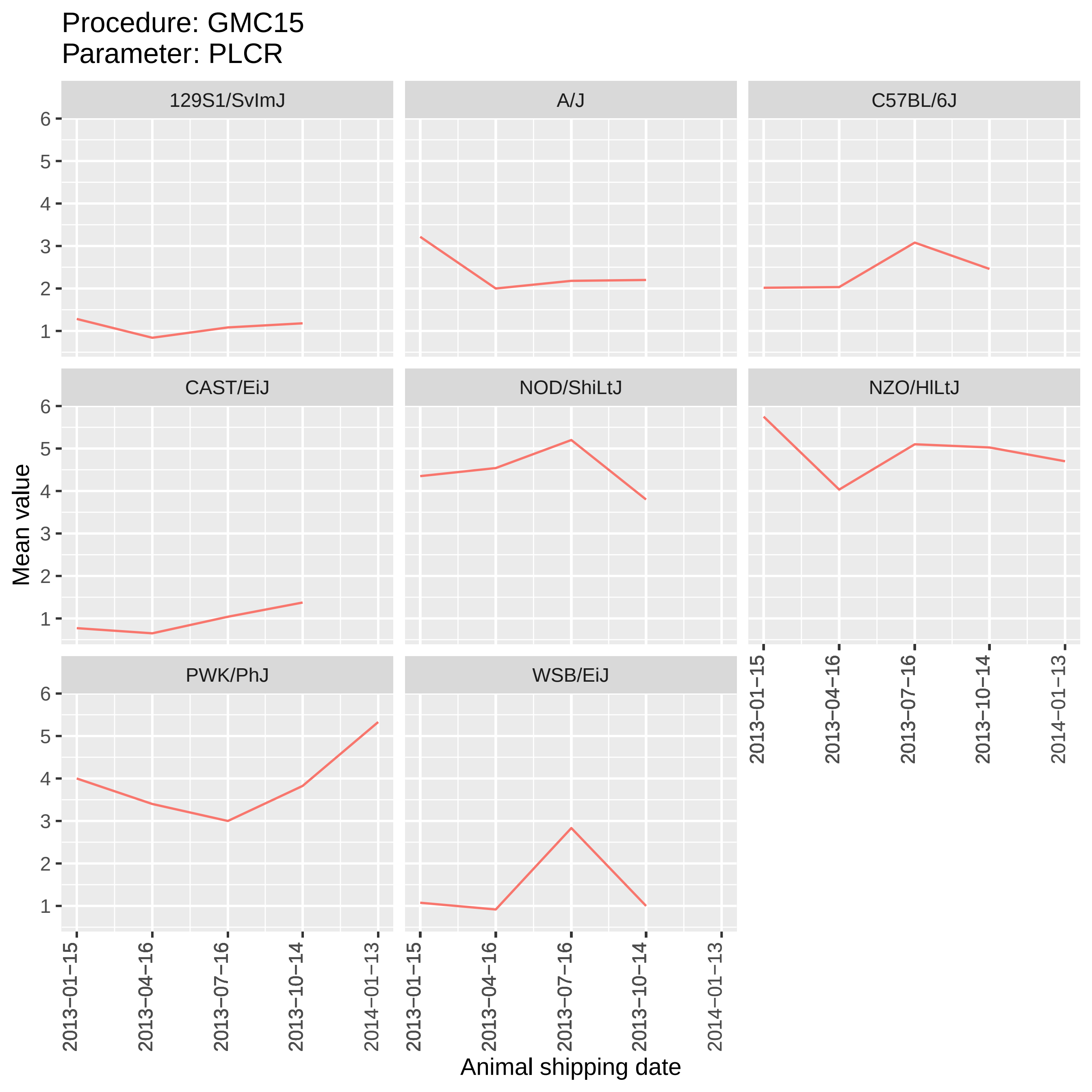

Procedure: GMC15
Parameter: PLCR
129S1/SvImJ
A/J
C57BL/6J
CAST/EiJ
NOD/ShiLtJ
NZO/HlLtJ
PWK/PhJ
WSB/EiJ
Mean value
Animal shipping date
1
2
3
4
5
6
2013−01−15
2013−04−16
2013−07−16
2013−10−14
2014−01−13

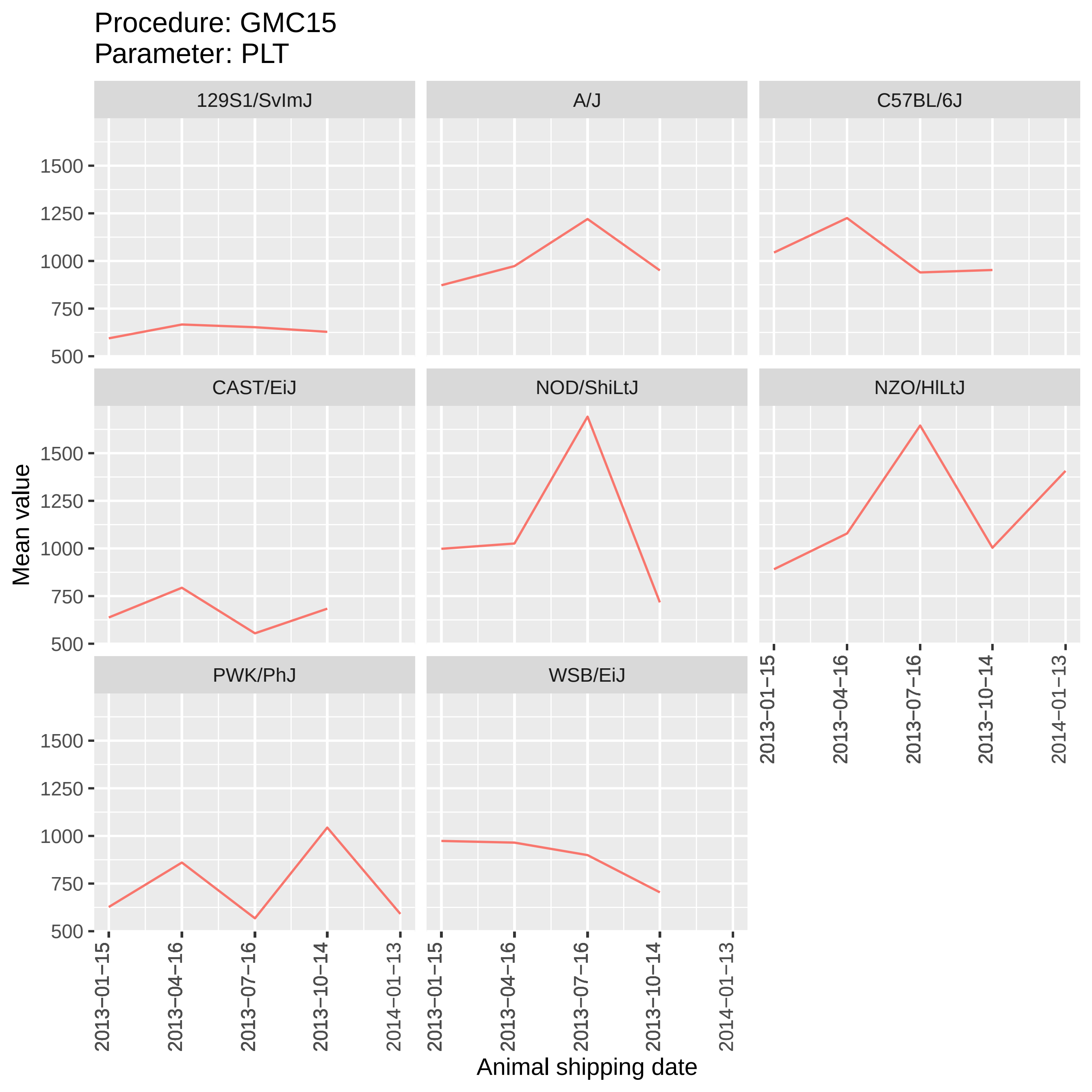
Procedure: GMC15
Parameter: PLT
129S1/SvImJ
A/J
C57BL/6J
CAST/EiJ
NOD/ShiLtJ
NZO/HlLtJ
PWK/PhJ
WSB/EiJ
Mean value
500
750
1000
1250
1500
2013−01−15
2013−04−16
2013−07−16
2013−10−14
2014−01−13
Animal shipping date

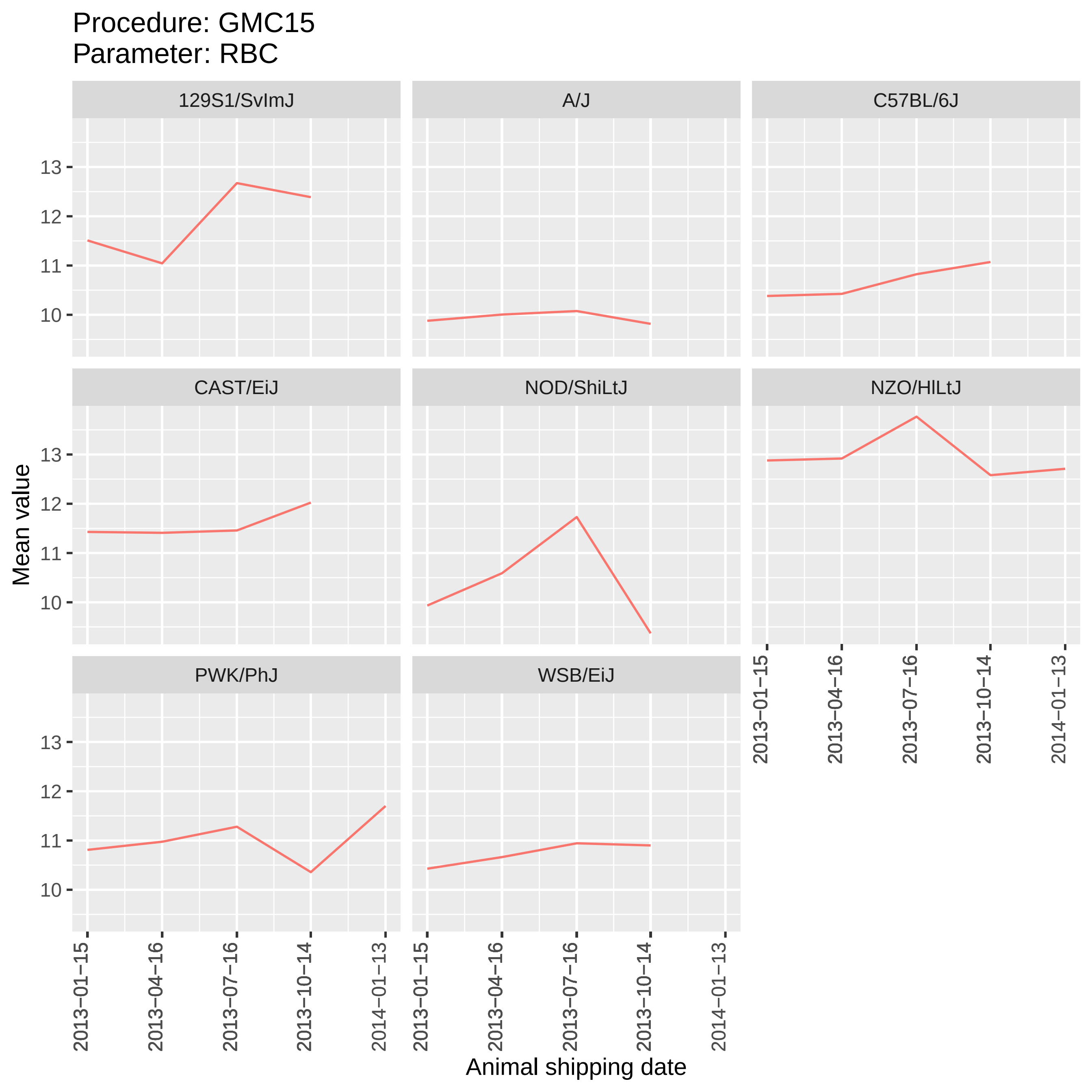
Procedure: GMC15
Parameter: RBC
129S1/SvImJ
A/J
C57BL/6J
CAST/EiJ
NOD/ShiLtJ
NZO/HlLtJ
PWK/PhJ
WSB/EiJ
Mean value
13
12
11
10
2013−01−15
2013−04−16
2013−07−16
2013−10−14
2014−01−13
Animal shipping date

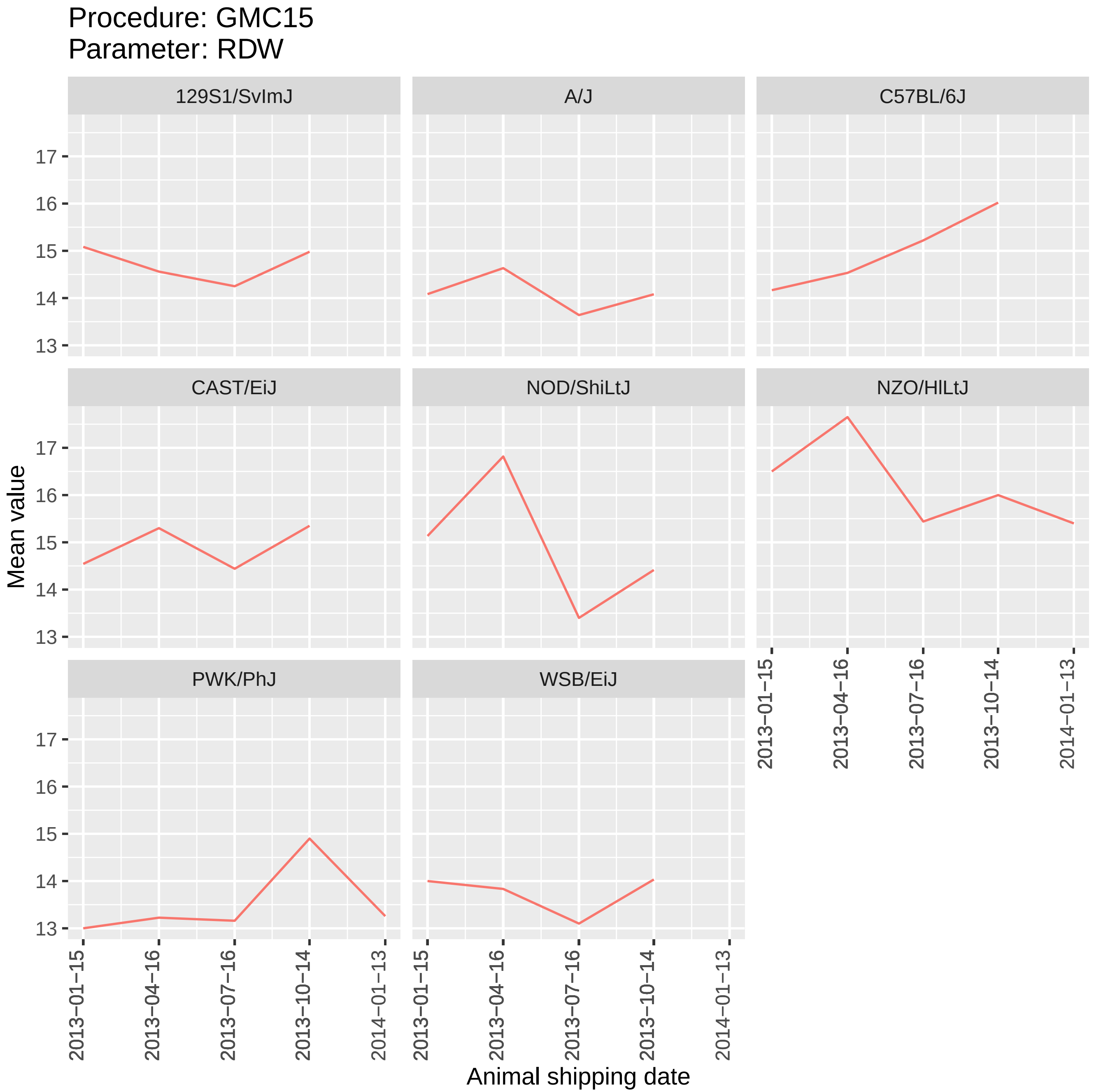
Procedure: GMC15
Parameter: RDW
129S1/SvImJ
A/J
C57BL/6J
CAST/EiJ
NOD/ShiLtJ
NZO/HlLtJ
PWK/PhJ
WSB/EiJ
Mean value
13
14
15
16
17
2013−01−15
2013−04−16
2013−07−16
2013−10−14
2014−01−13
Animal shipping date

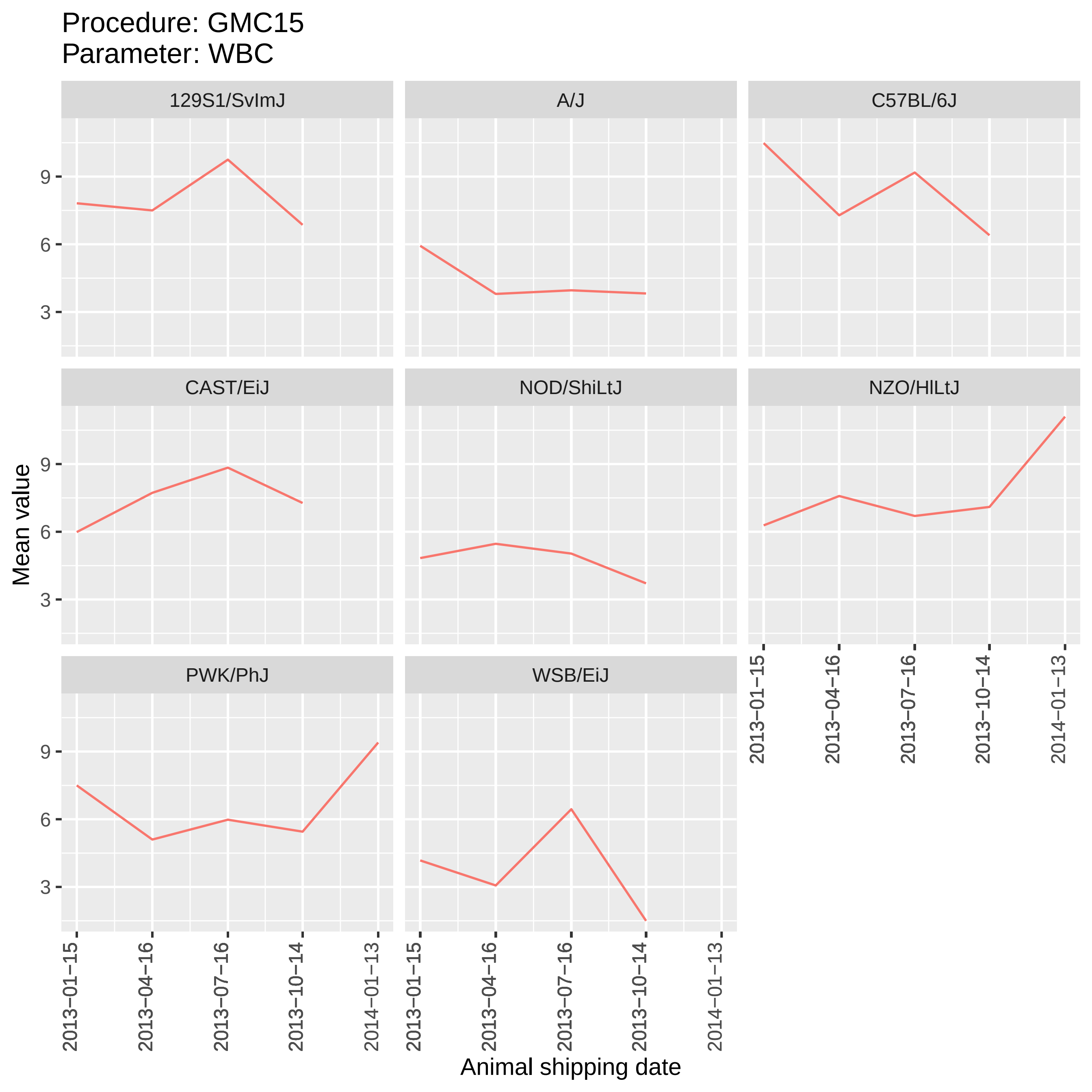

Procedure: GMC15
Parameter: WBC
129S1/SvImJ
A/J
C57BL/6J
CAST/EiJ
NOD/ShiLtJ
NZO/HlLtJ
PWK/PhJ
WSB/EiJ
Mean value
3
6
9
2013−01−15
2013−04−16
2013−07−16
2013−10−14
2014−01−13
Animal shipping date

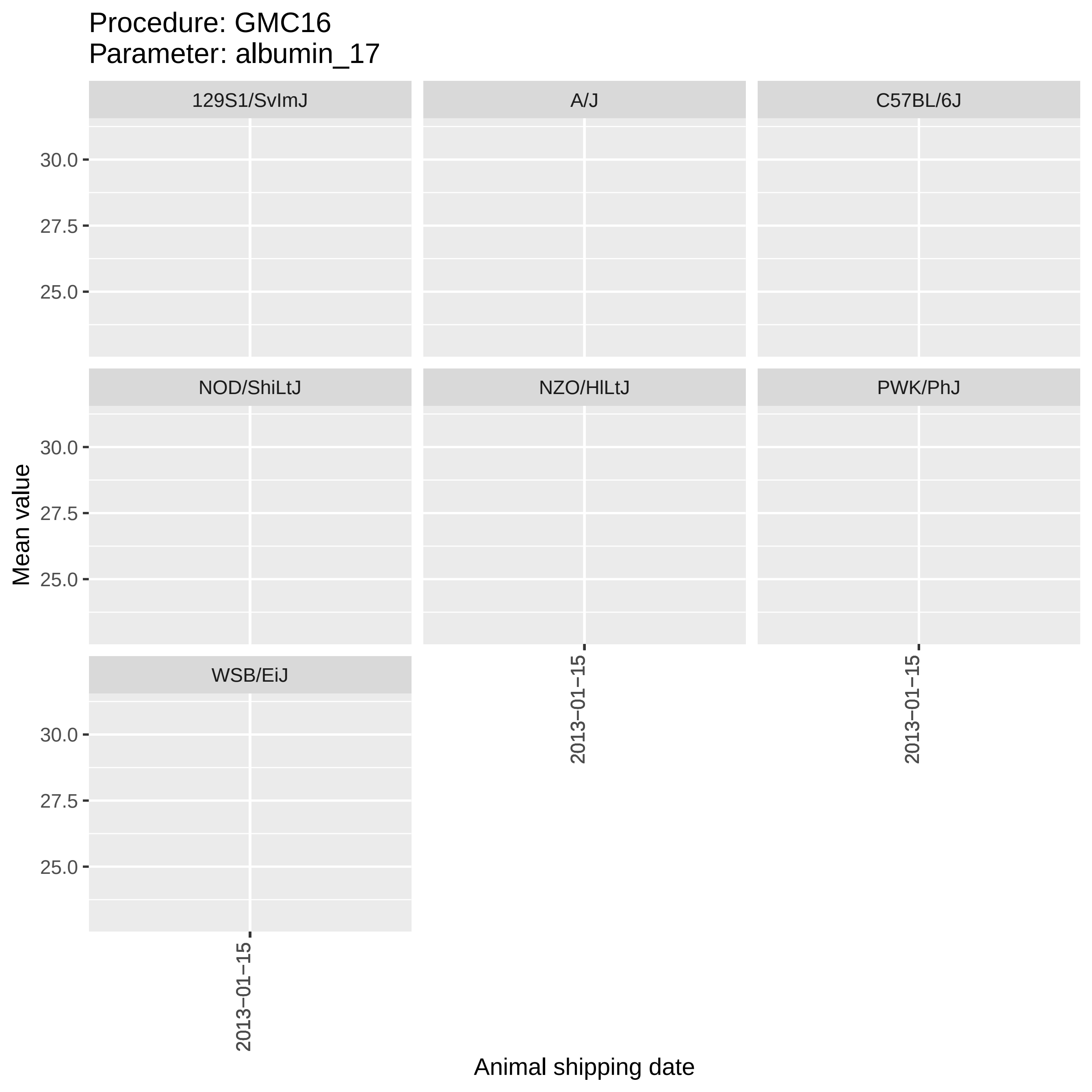

Procedure: GMC16
Parameter: albumin_17
129S1/SvImJ
A/J
C57BL/6J
NOD/ShiLtJ
NZO/HlLtJ
PWK/PhJ
WSB/EiJ
30.0
27.5
25.0
Mean value
2013−01−15
Animal shipping date

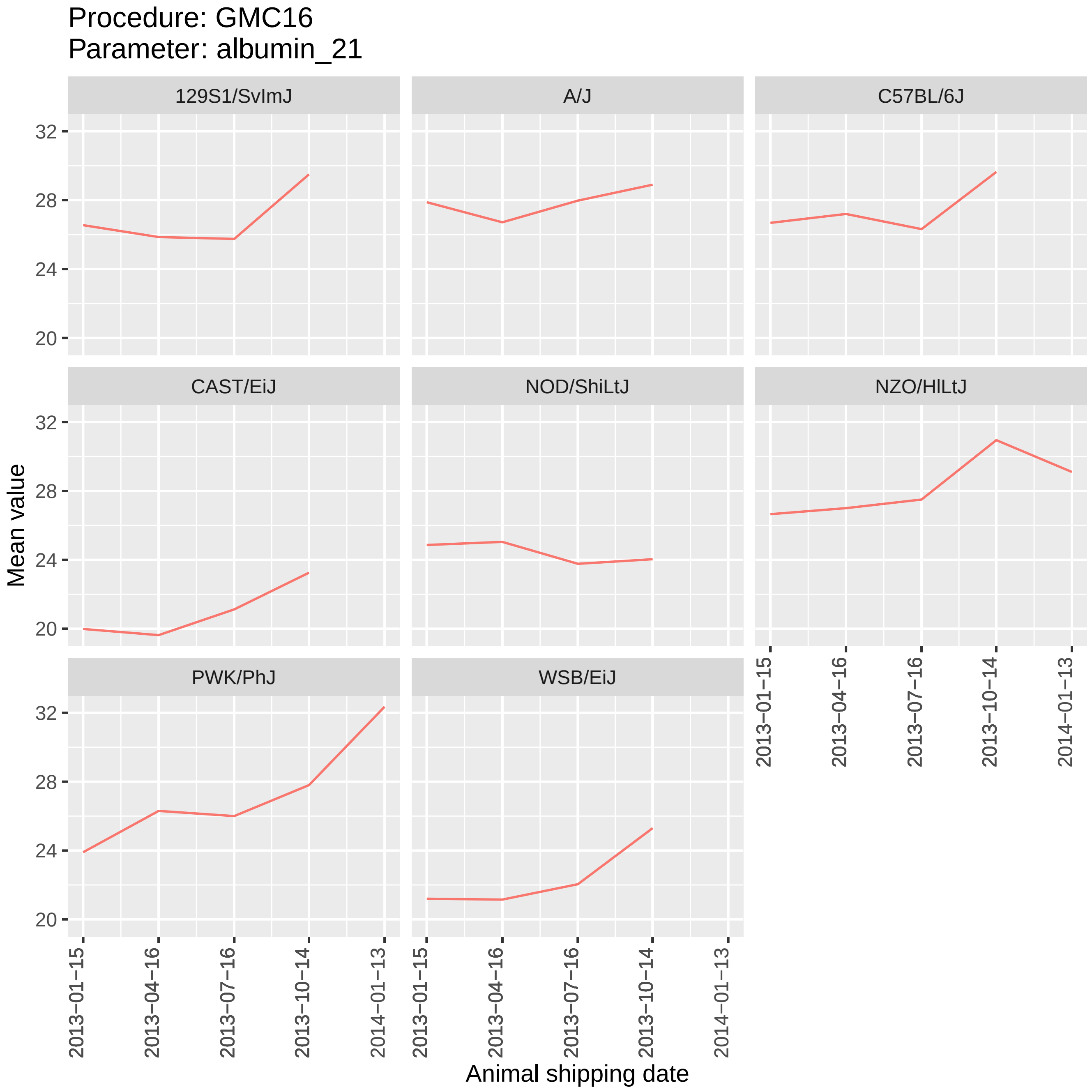
Procedure: GMC16
Parameter: albumin_21
129S1/SvImJ
A/J
C57BL/6J
CAST/EiJ
NOD/ShiLtJ
NZO/HlLtJ
PWK/PhJ
WSB/EiJ
Mean value
20
24
28
32
2013−01−15
2013−04−16
2013−07−16
2013−10−14
2014−01−13
Animal shipping date

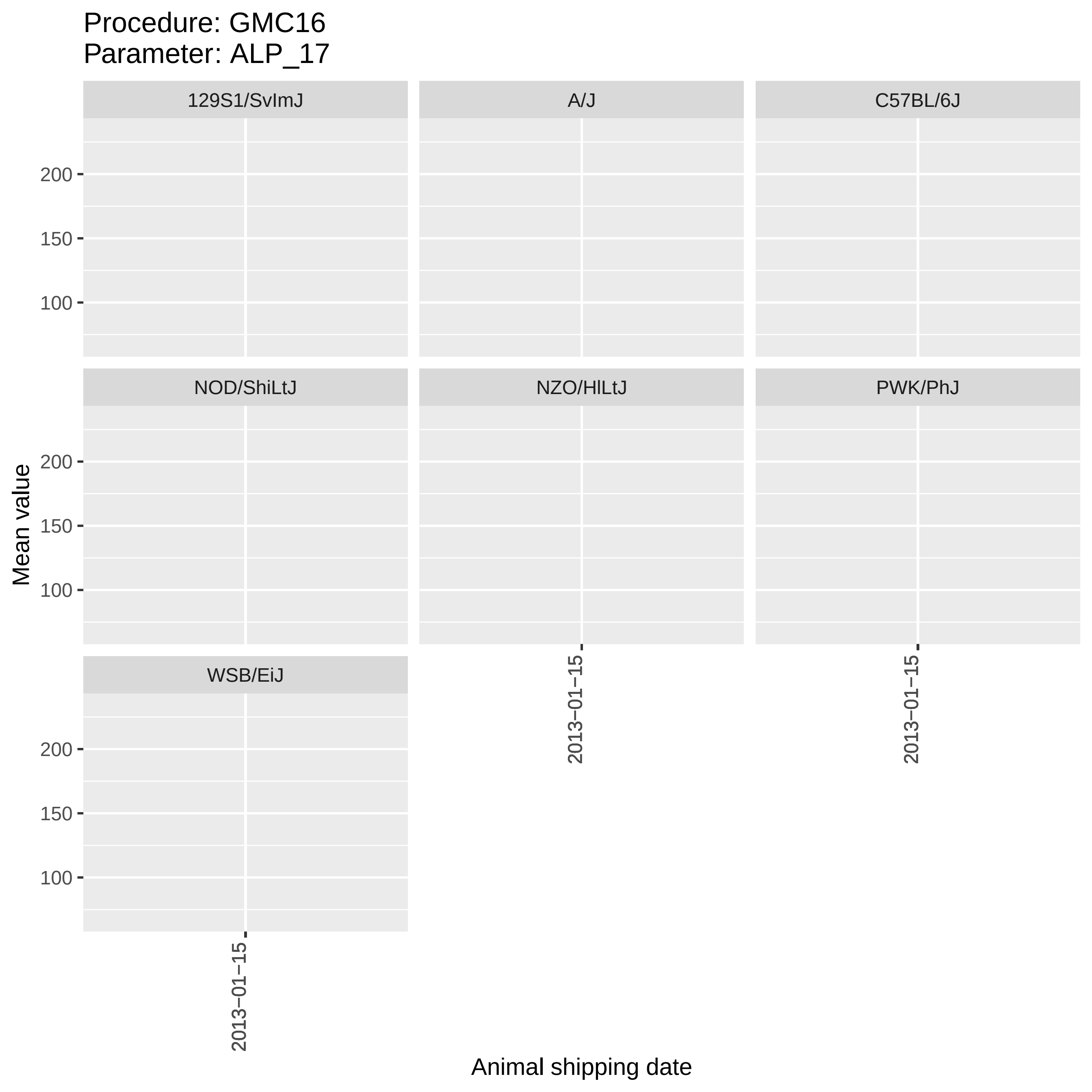

Procedure: GMC16
Parameter: ALP_17
129S1/SvImJ
A/J
C57BL/6J
NOD/ShiLtJ
NZO/HlLtJ
PWK/PhJ
WSB/EiJ
200
150
100
Mean value
2013−01−15
Animal shipping date

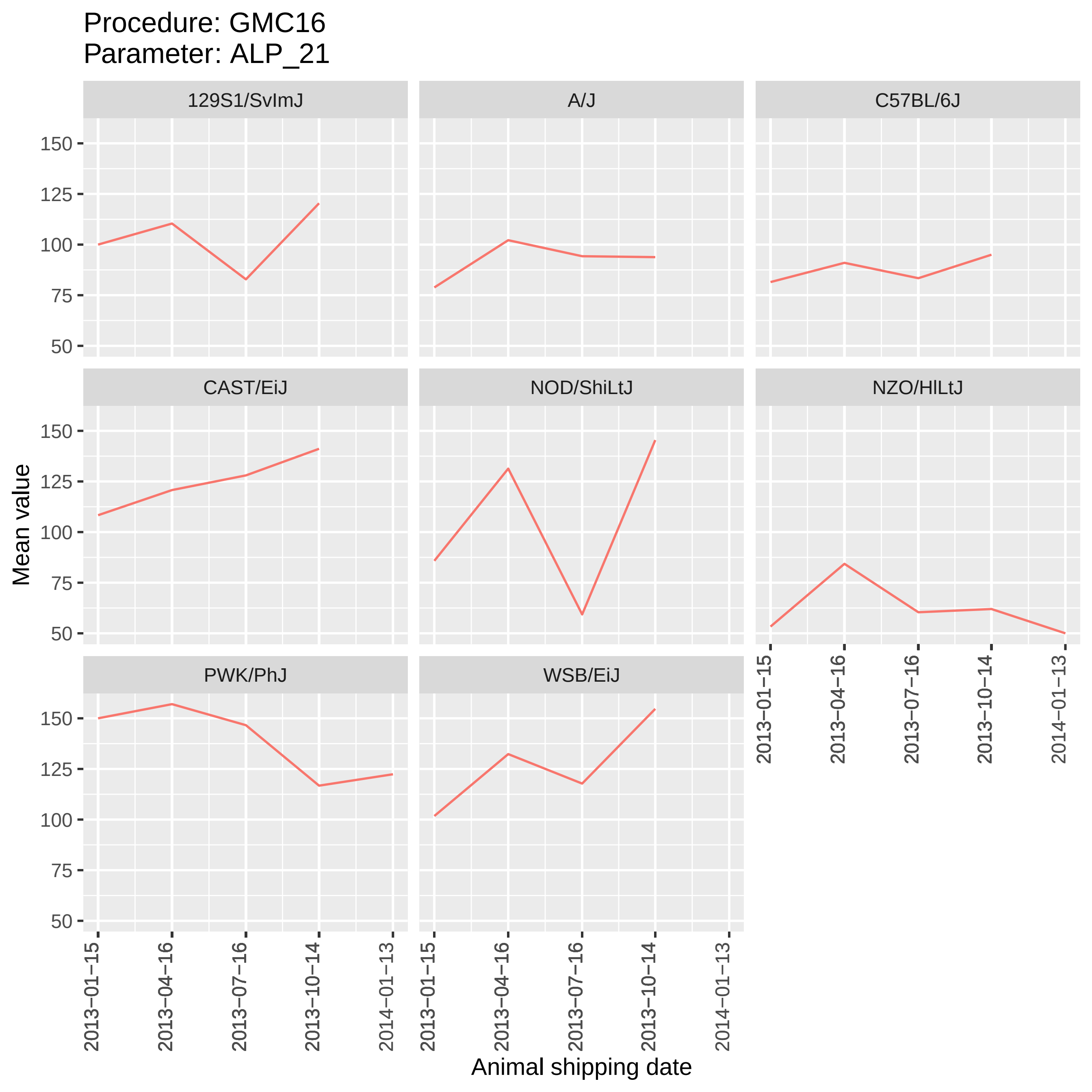

Procedure: GMC16
Parameter: ALP_21
129S1/SvImJ
A/J
C57BL/6J
CAST/EiJ
NOD/ShiLtJ
NZO/HlLtJ
PWK/PhJ
WSB/EiJ
Mean value
150
125
100
75
50
2013−01−15
2013−04−16
2013−07−16
2013−10−14
2014−01−13
Animal shipping date

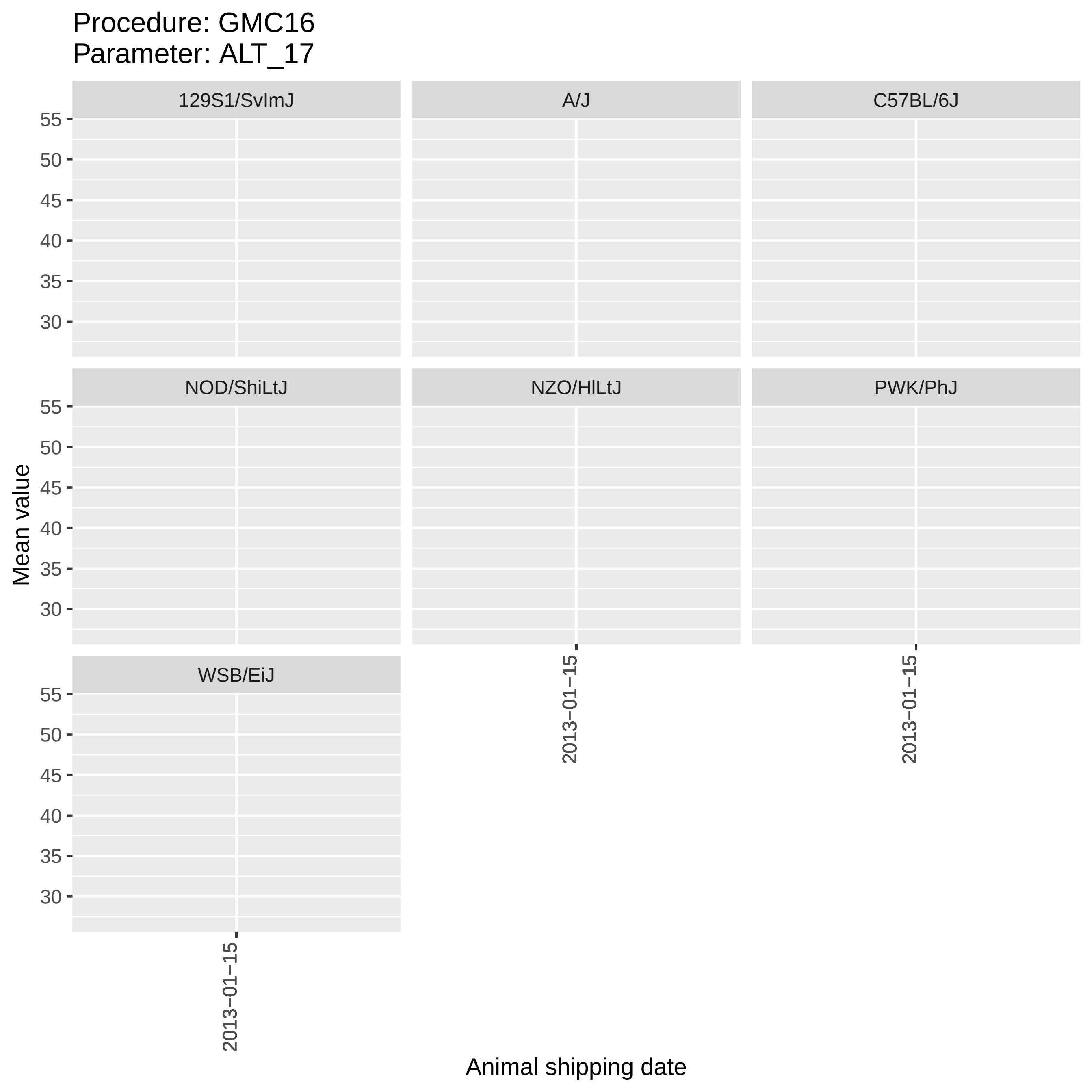

Procedure: GMC16
Parameter: ALT_17
129S1/SvImJ
A/J
C57BL/6J
NOD/ShiLtJ
NZO/HlLtJ
PWK/PhJ
WSB/EiJ
55
50
45
40
35
30
Mean value
2013−01−15
Animal shipping date

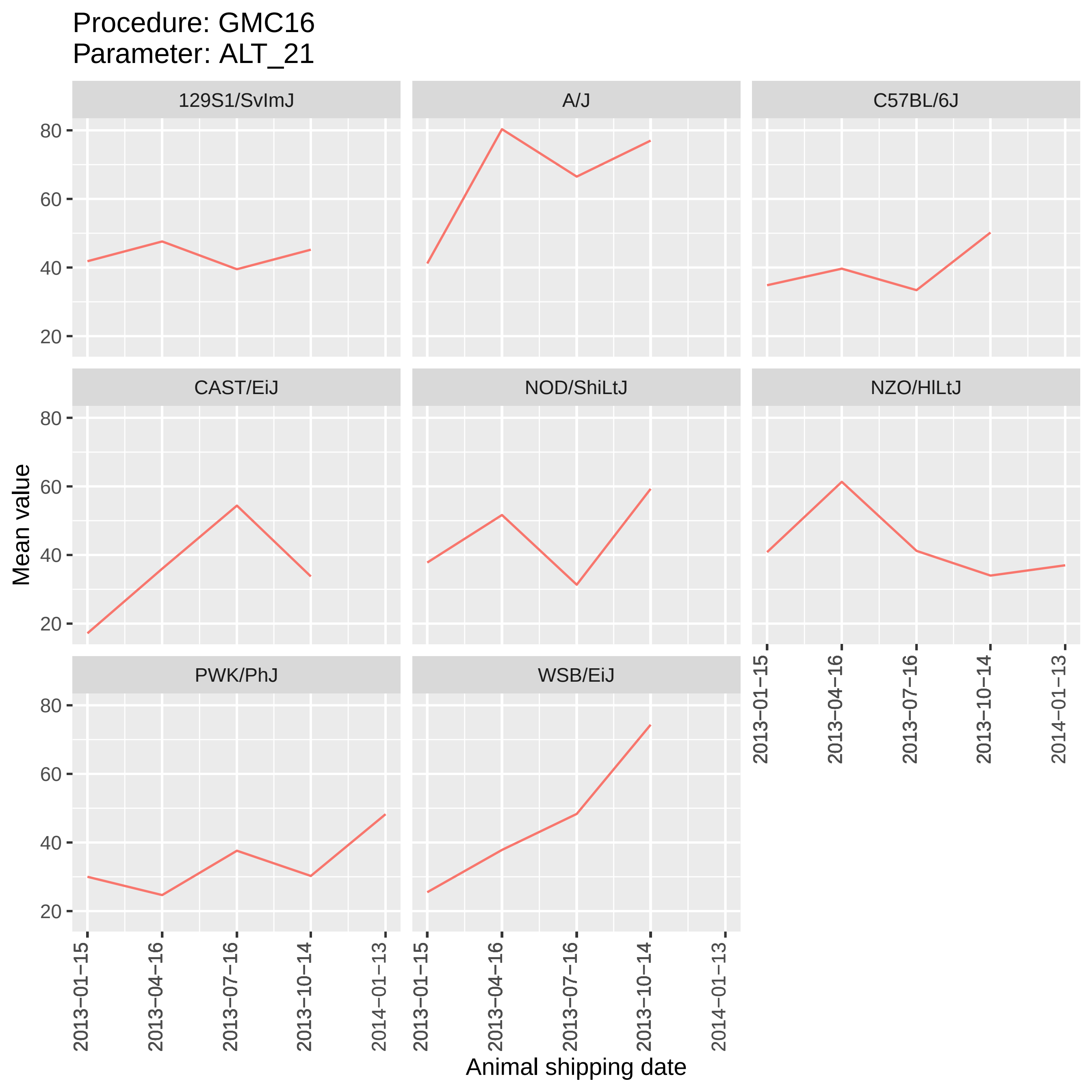

Procedure: GMC16
Parameter: ALT_21
129S1/SvImJ
A/J
C57BL/6J
CAST/EiJ
NOD/ShiLtJ
NZO/HlLtJ
PWK/PhJ
WSB/EiJ
Mean value
20
40
60
80
2013−01−15
2013−04−16
2013−07−16
2013−10−14
2014−01−13
Animal shipping date

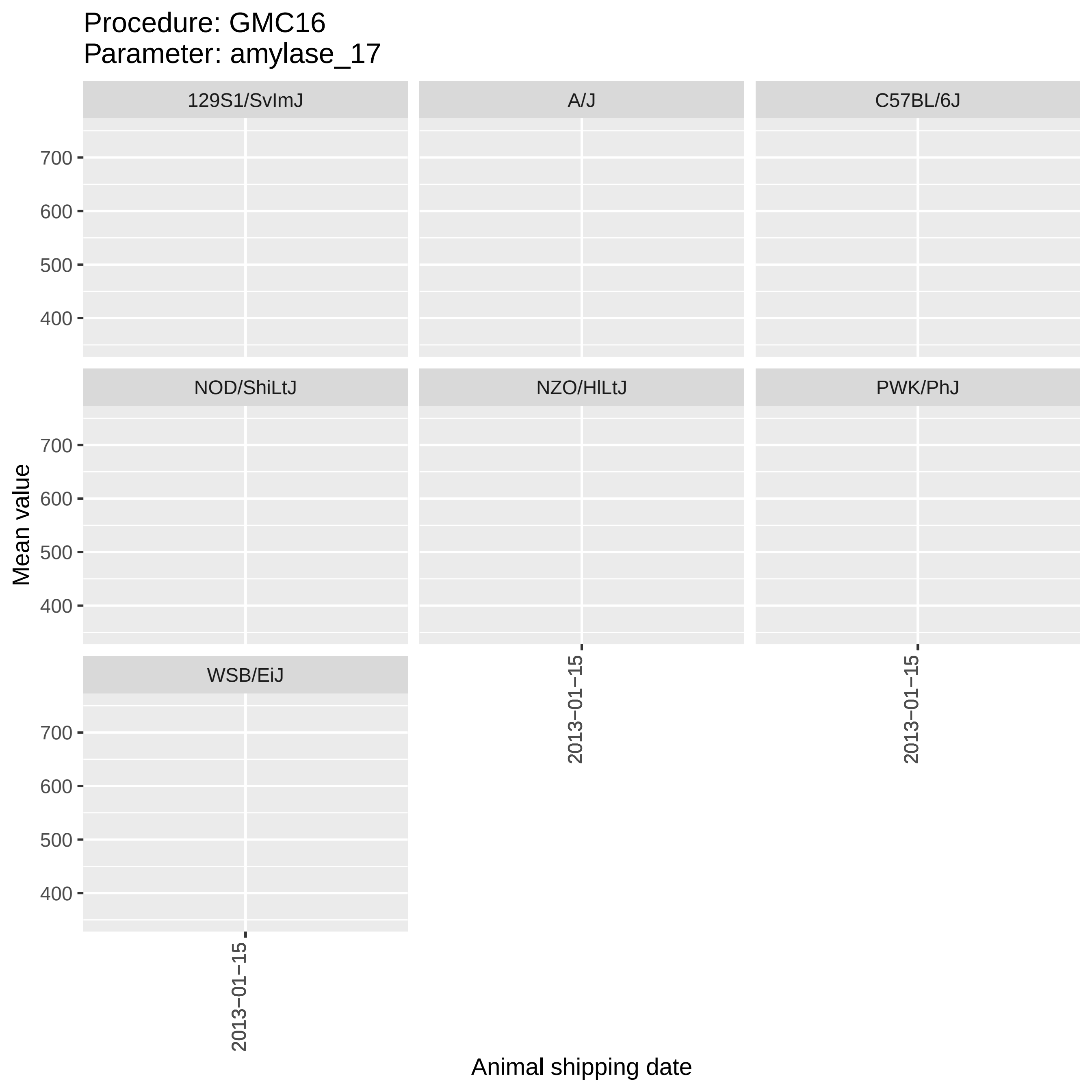
Procedure: GMC16
Parameter: amylase_17
129S1/SvImJ
A/J
C57BL/6J
NOD/ShiLtJ
NZO/HlLtJ
PWK/PhJ
WSB/EiJ
Mean value
400
500
600
700
2013−01−15
Animal shipping date

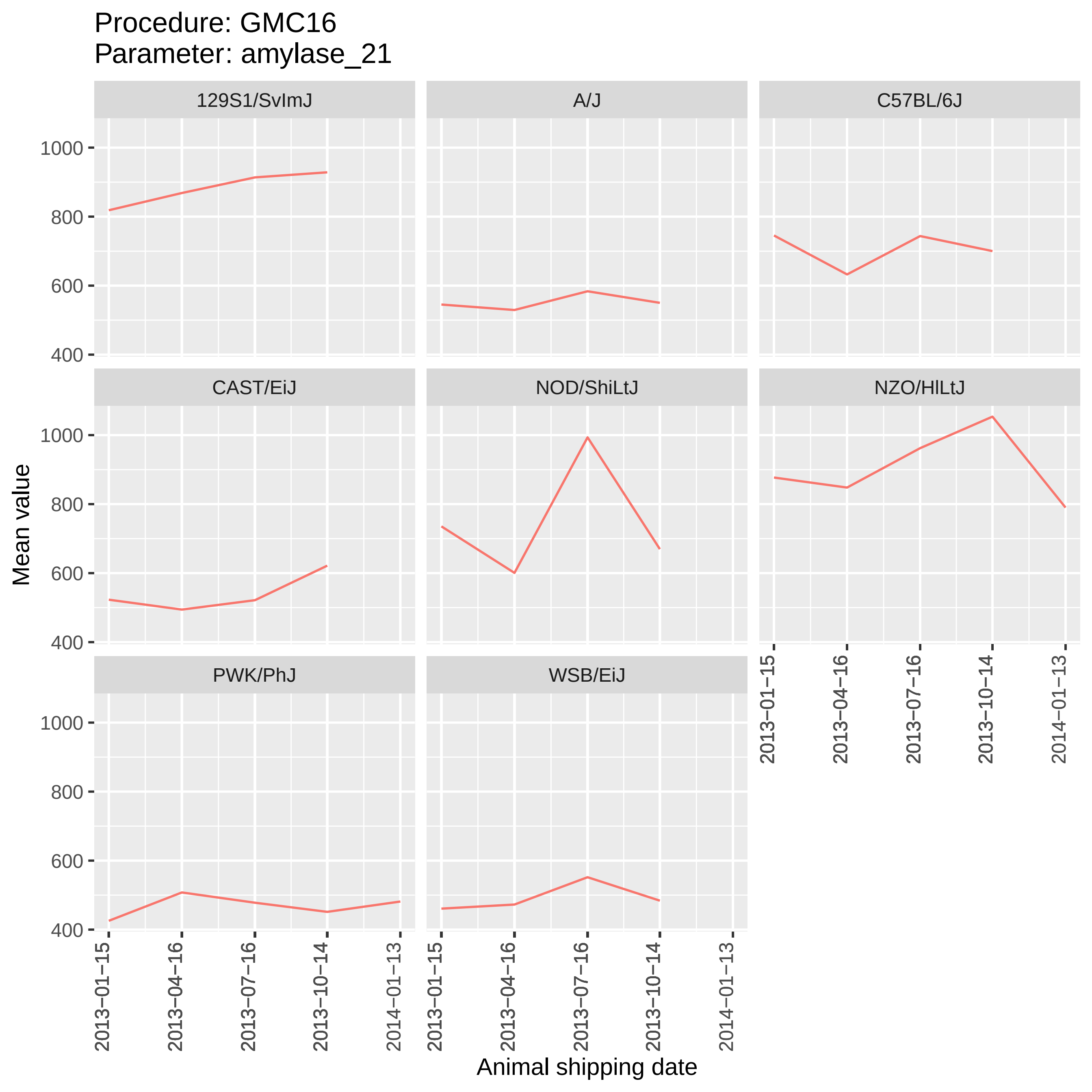

Procedure: GMC16
Parameter: amylase_21
129S1/SvImJ
A/J
C57BL/6J
CAST/EiJ
NOD/ShiLtJ
NZO/HlLtJ
PWK/PhJ
WSB/EiJ
Mean value
400
600
800
1000
2013−01−15
2013−04−16
2013−07−16
2013−10−14
2014−01−13
Animal shipping date

Procedure: GMC16
Parameter: AST_17

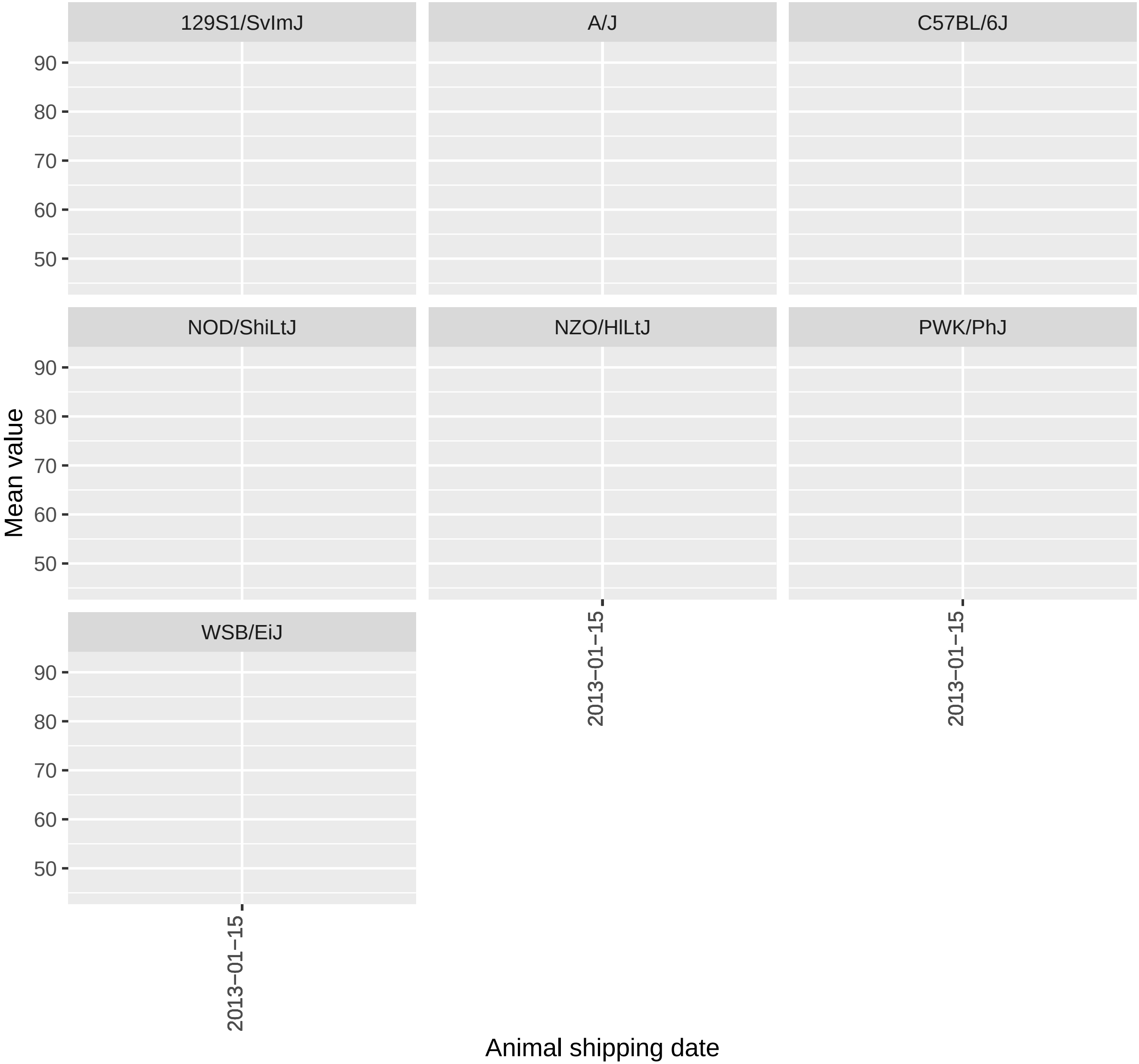

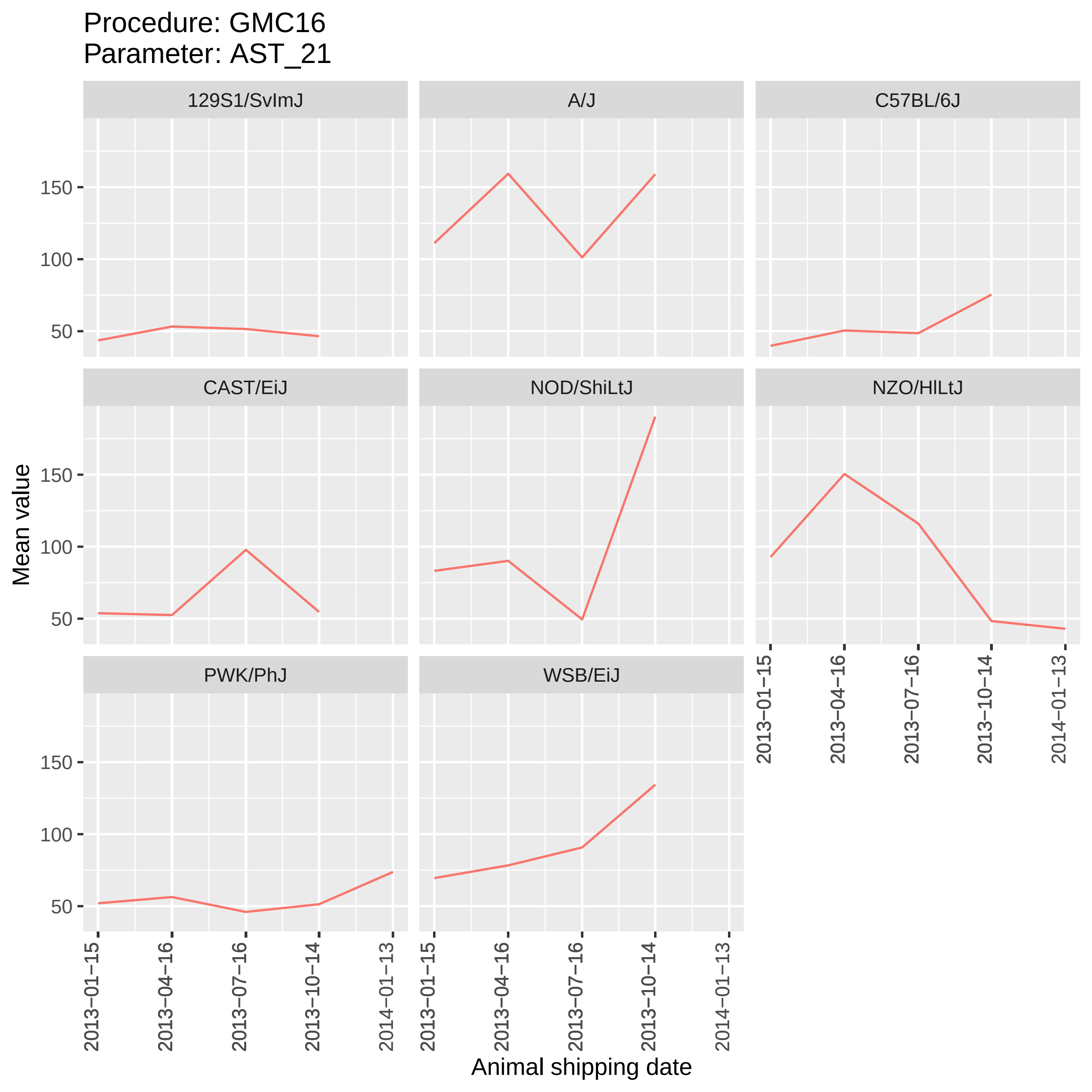

Procedure: GMC16
Parameter: AST_21
129S1/SvImJ
A/J
C57BL/6J
CAST/EiJ
NOD/ShiLtJ
NZO/HlLtJ
PWK/PhJ
WSB/EiJ
Mean value
150
100
50
2013−01−15
2013−04−16
2013−07−16
2013−10−14
2014−01−13
Animal shipping date

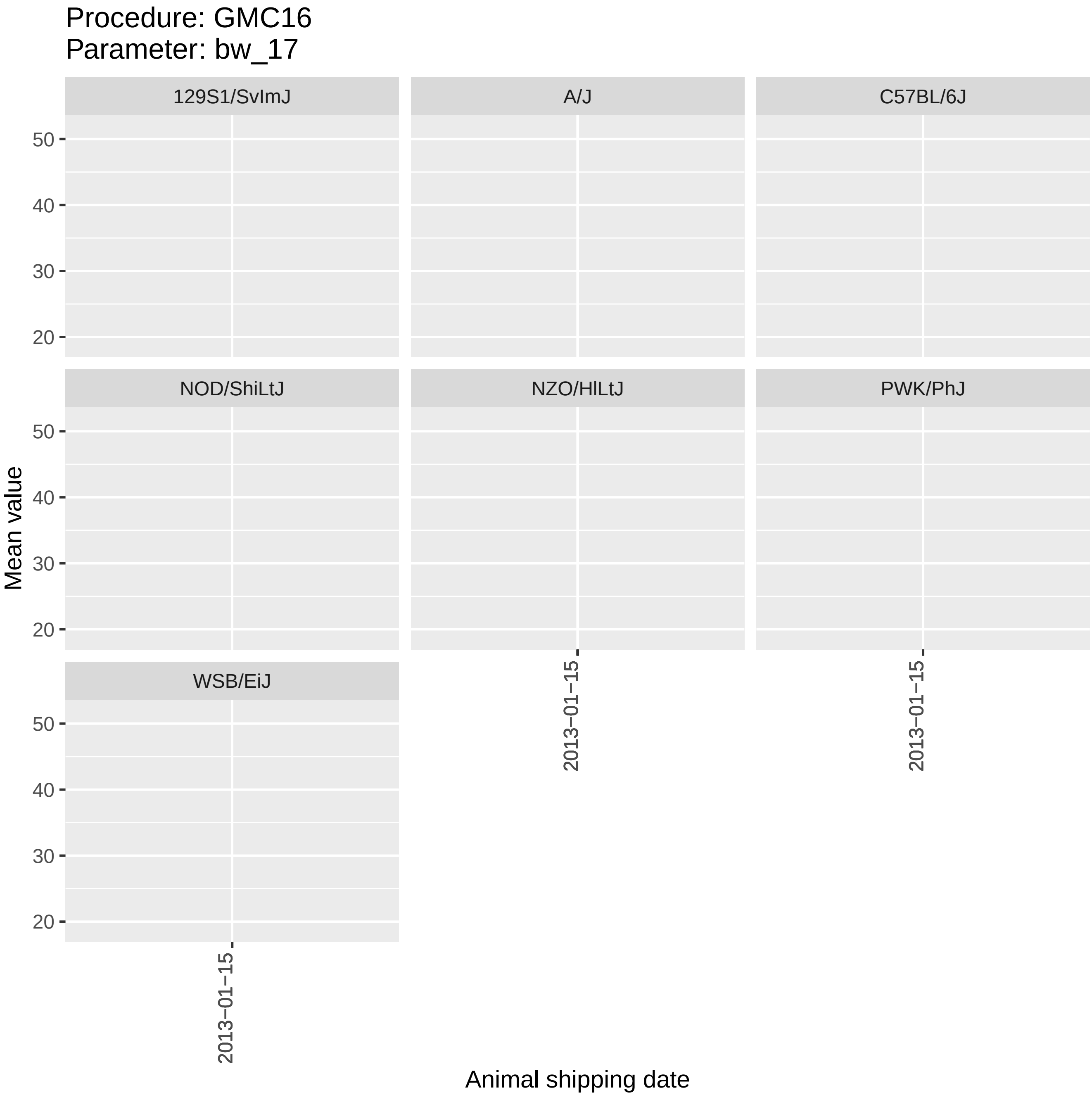
Procedure: GMC16
Parameter: bw_17
129S1/SvImJ
A/J
C57BL/6J
NOD/ShiLtJ
NZO/HlLtJ
PWK/PhJ
WSB/EiJ
50
40
30
20
Mean value
2013−01−15
Animal shipping date

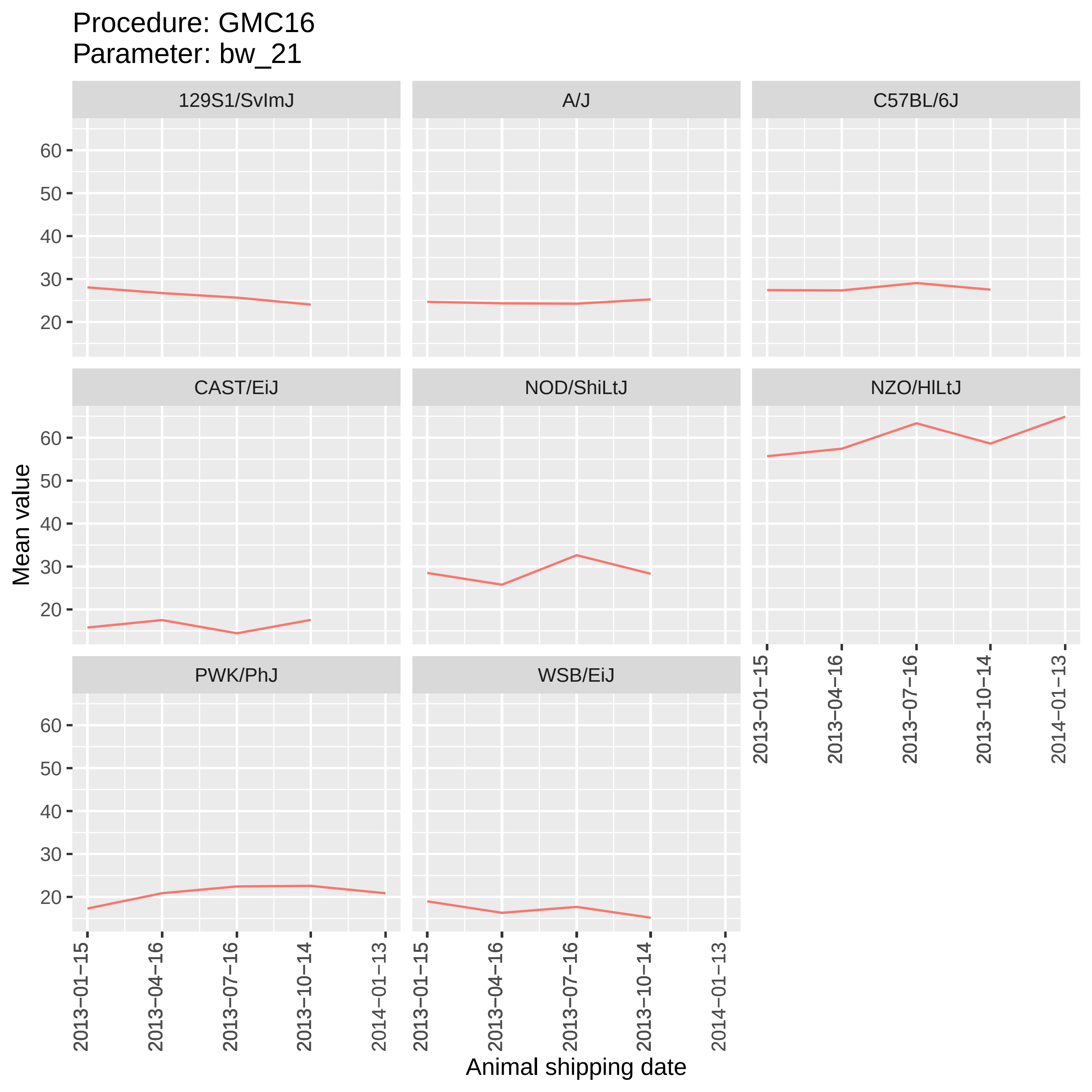

Procedure: GMC16
Parameter: bw_21
129S1/SvImJ
A/J
C57BL/6J
CAST/EiJ
NOD/ShiLtJ
NZO/HlLtJ
PWK/PhJ
WSB/EiJ
Mean value
20
30
40
50
60
2013−01−15
2013−04−16
2013−07−16
2013−10−14
2014−01−13
Animal shipping date

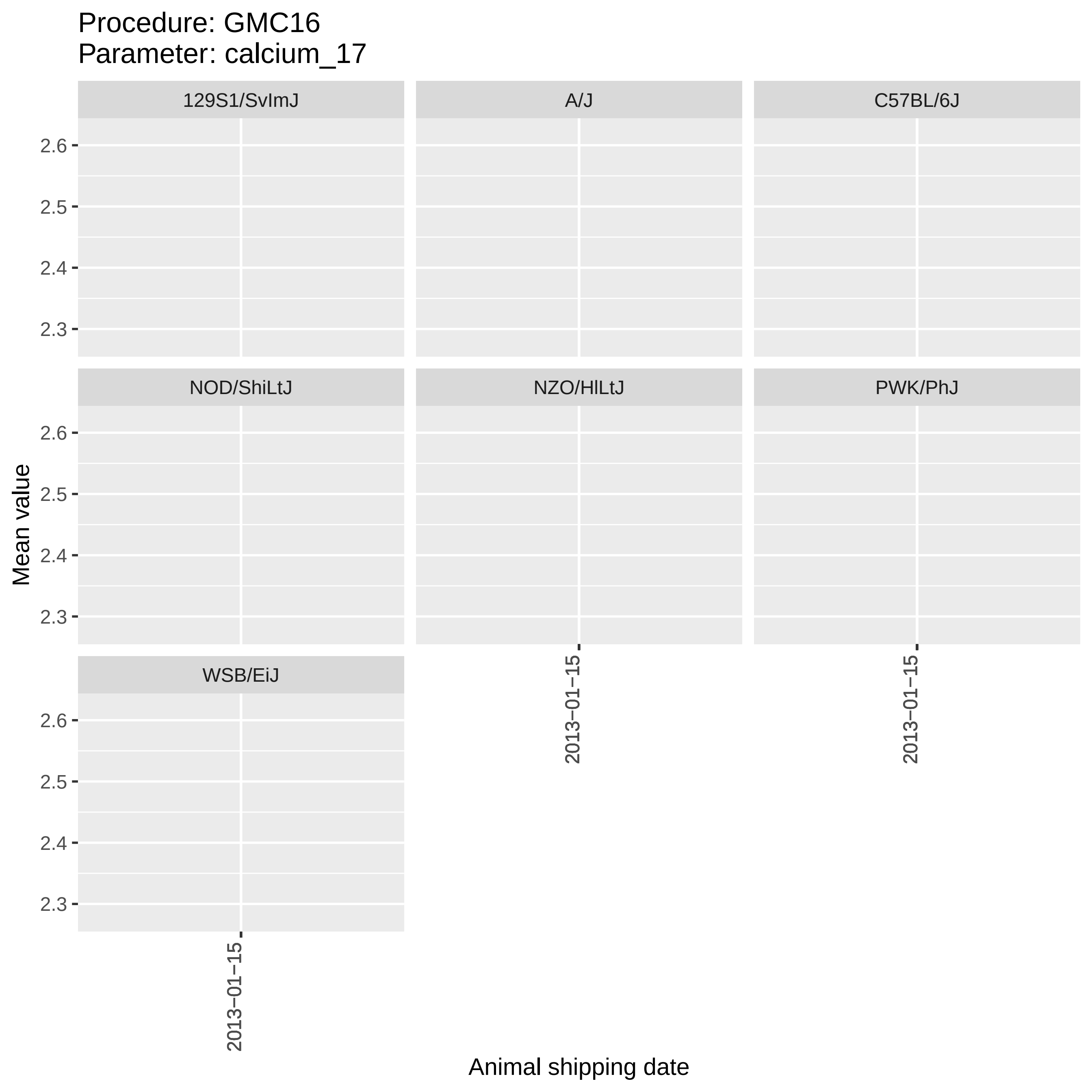
Procedure: GMC16
Parameter: calcium_17
129S1/SvImJ
A/J
C57BL/6J
NOD/ShiLtJ
NZO/HlLtJ
PWK/PhJ
WSB/EiJ
2.6
2.5
2.4
2.3
Mean value
2013−01−15
Animal shipping date

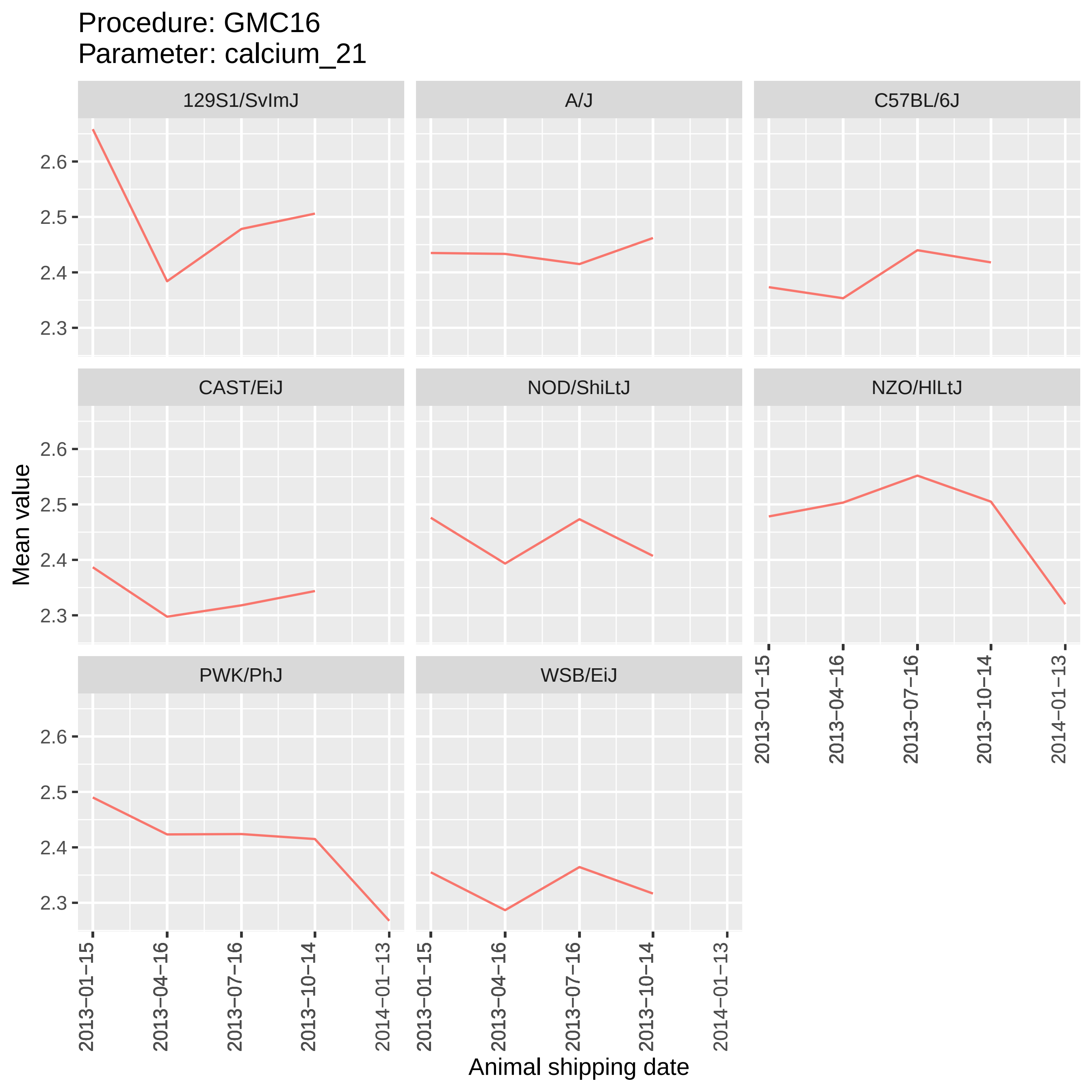

Procedure: GMC16
Parameter: calcium_21
129S1/SvImJ
A/J
C57BL/6J
CAST/EiJ
NOD/ShiLtJ
NZO/HlLtJ
PWK/PhJ
WSB/EiJ
Mean value
2.3
2.4
2.5
2.6
2013−01−15
2013−04−16
2013−07−16
2013−10−14
2014−01−13
Animal shipping date

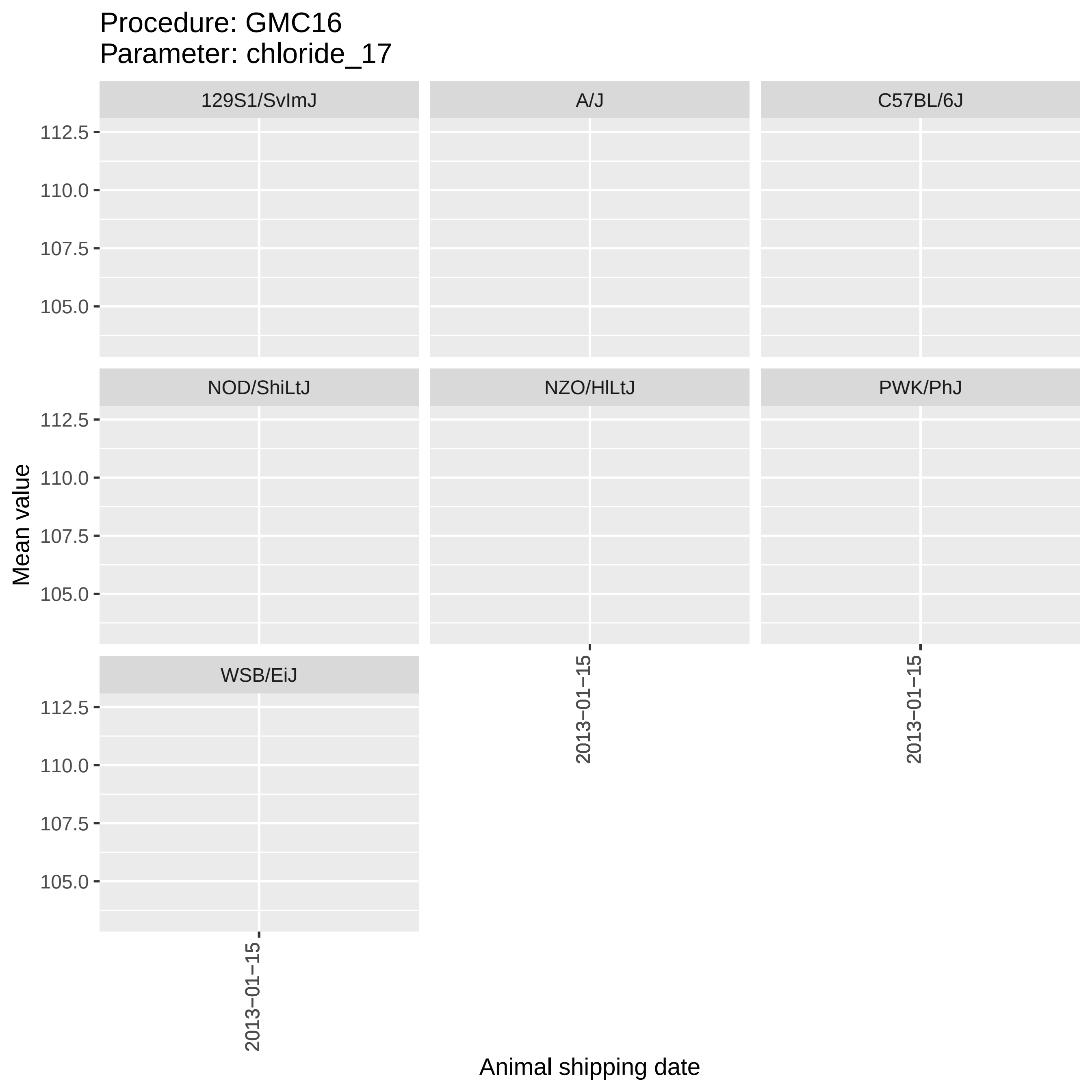

Procedure: GMC16
Parameter: chloride_17
129S1/SvImJ
A/J
C57BL/6J
NOD/ShiLtJ
NZO/HlLtJ
PWK/PhJ
WSB/EiJ
112.5
110.0
107.5
105.0
Mean value
2013−01−15
Animal shipping date

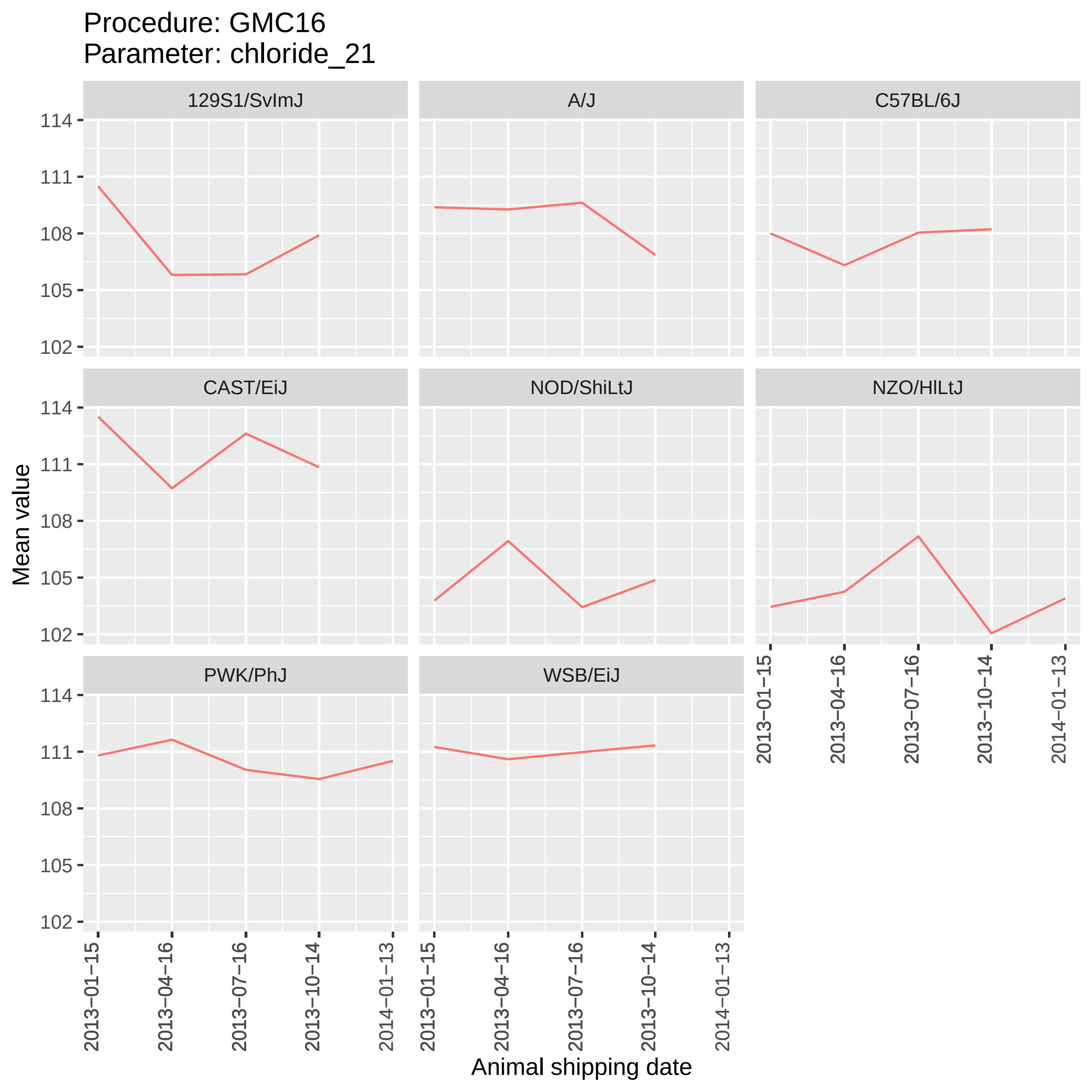
Procedure: GMC16
Parameter: chloride_21
129S1/SvImJ
A/J
C57BL/6J
CAST/EiJ
NOD/ShiLtJ
NZO/HlLtJ
PWK/PhJ
WSB/EiJ
114
111
108
105
102
2013−01−15
2013−04−16
2013−07−16
2013−10−14
2014−01−13
Mean value
Animal shipping date

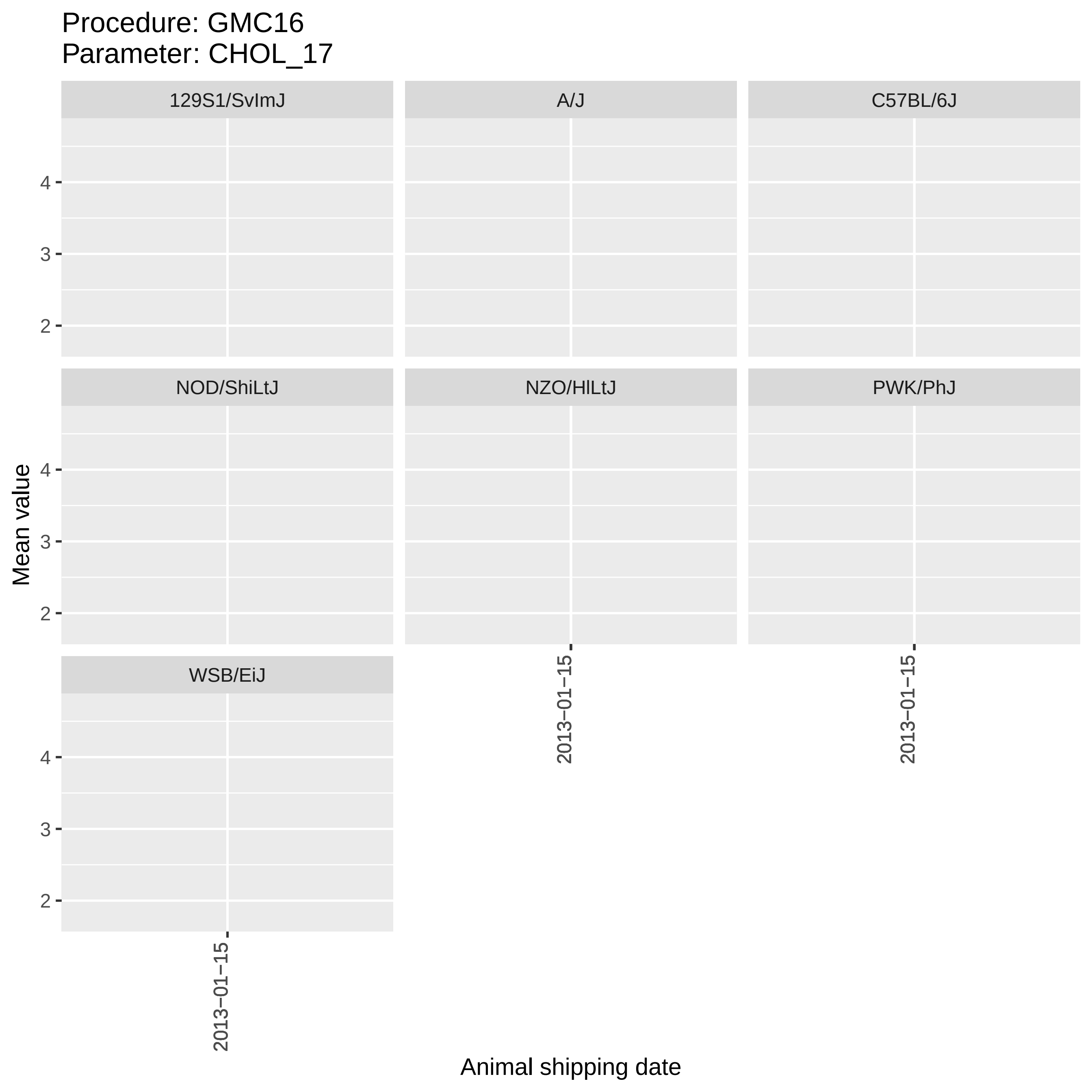

Procedure: GMC16
Parameter: CHOL_17
129S1/SvImJ
A/J
C57BL/6J
NOD/ShiLtJ
NZO/HlLtJ
PWK/PhJ
WSB/EiJ
Mean value
2
3
4
2013−01−15
Animal shipping date

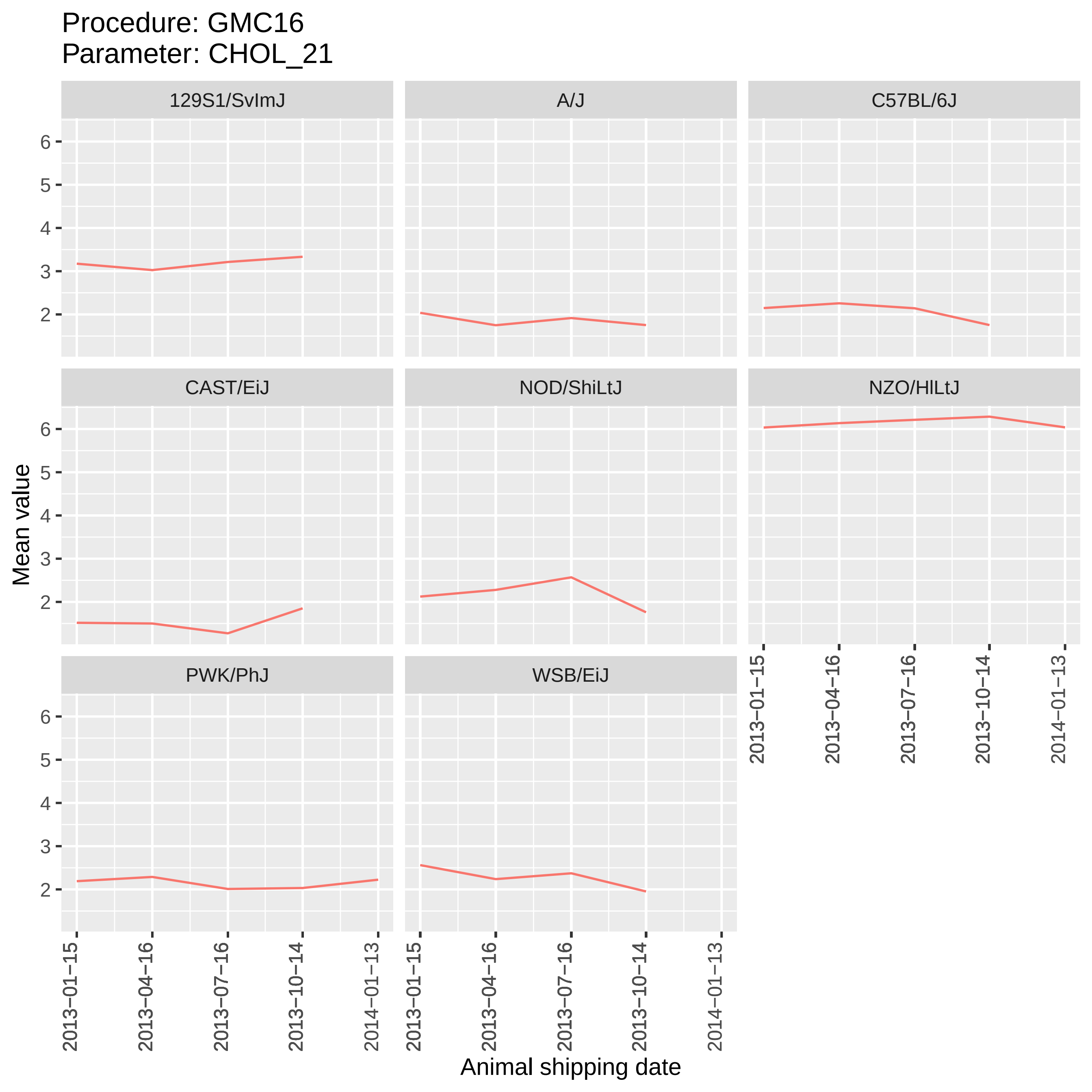

Procedure: GMC16
Parameter: CHOL_21
129S1/SvImJ
A/J
C57BL/6J
CAST/EiJ
NOD/ShiLtJ
NZO/HlLtJ
PWK/PhJ
WSB/EiJ
Mean value
Animal shipping date
2
3
4
5
6
2013−01−15
2013−04−16
2013−07−16
2013−10−14
2014−01−13

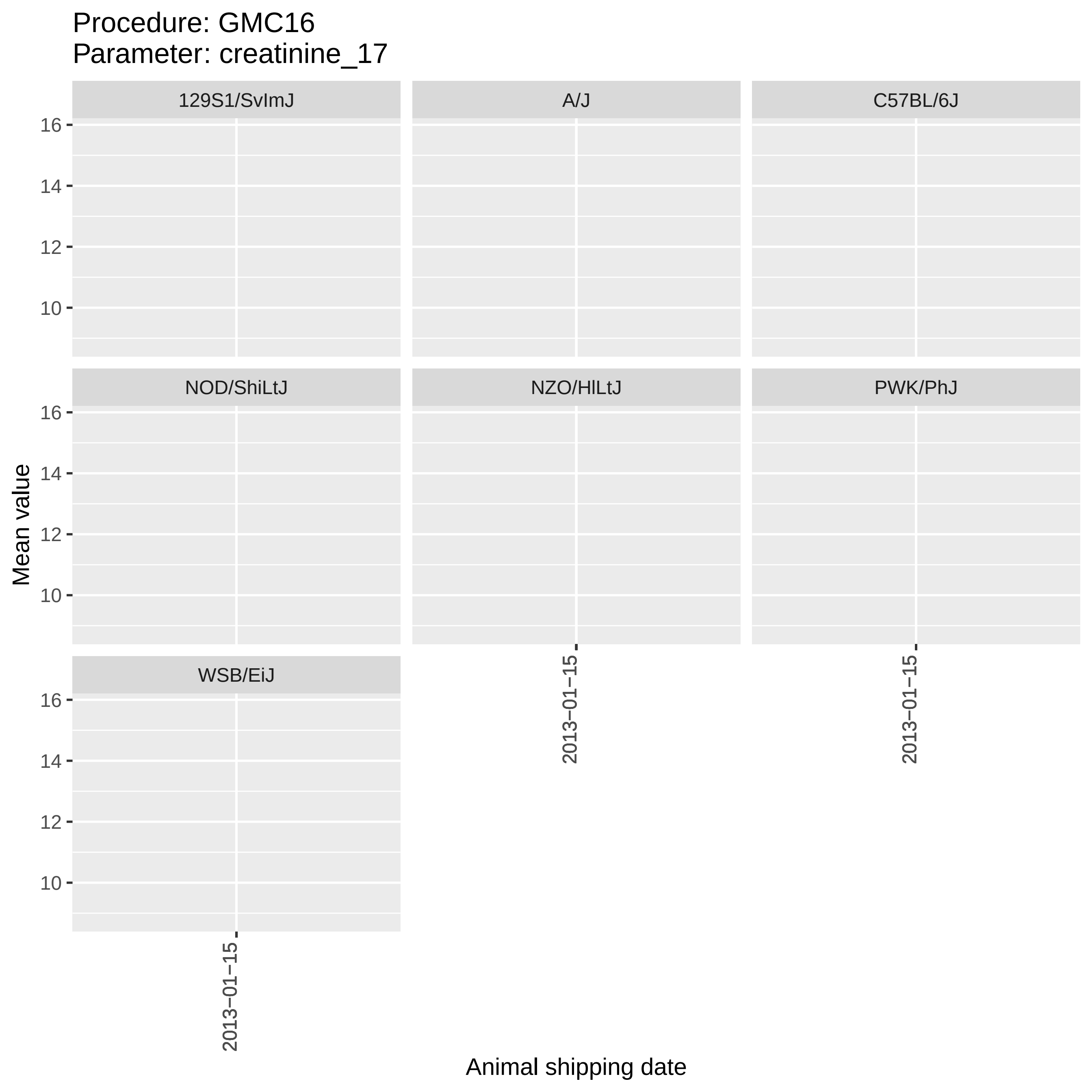

Procedure: GMC16
Parameter: creatinine_17
129S1/SvImJ
A/J
C57BL/6J
NOD/ShiLtJ
NZO/HlLtJ
PWK/PhJ
WSB/EiJ
16
14
12
10
Mean value
2013−01−15
Animal shipping date

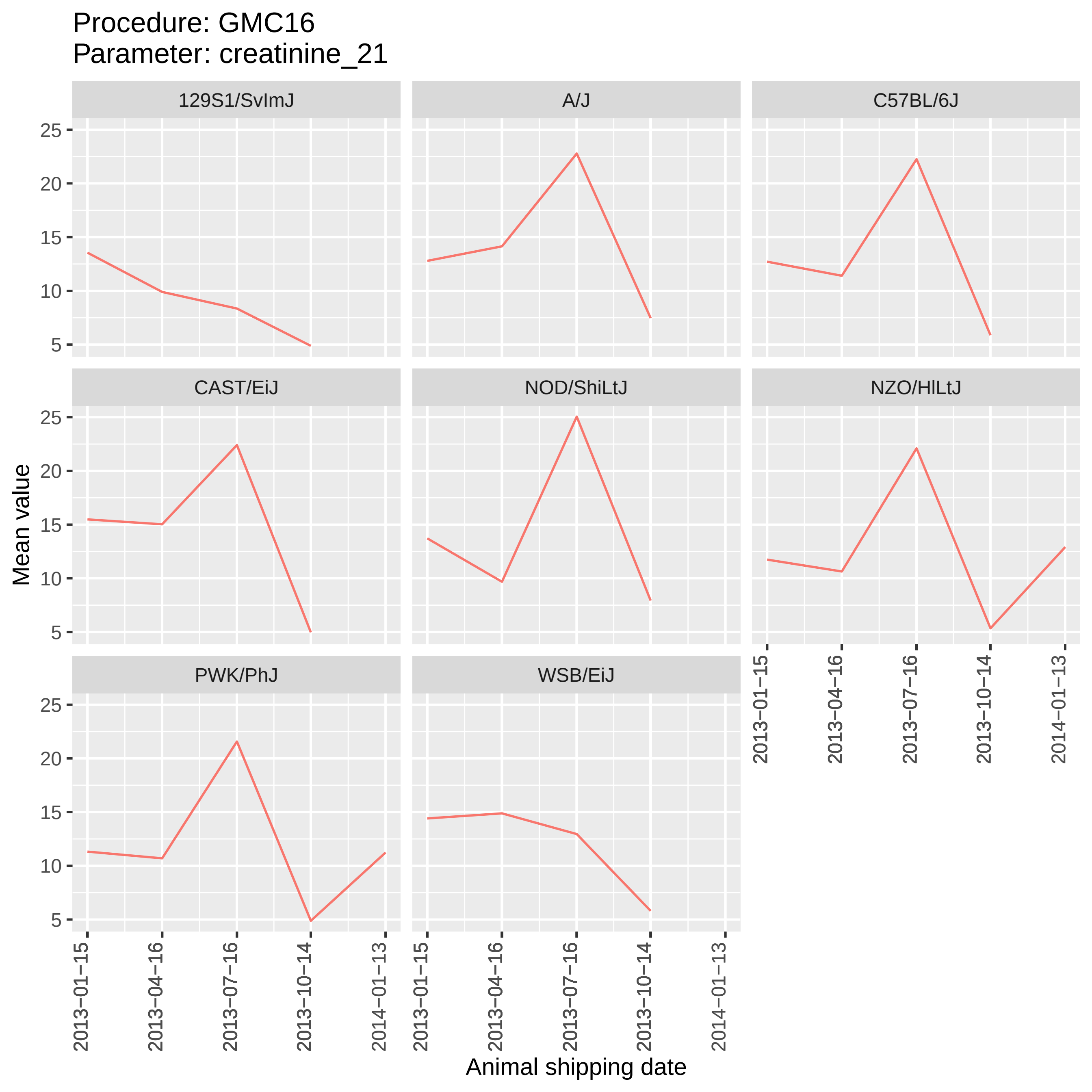
Procedure: GMC16
Parameter: creatinine_21
129S1/SvImJ
A/J
C57BL/6J
CAST/EiJ
NOD/ShiLtJ
NZO/HlLtJ
PWK/PhJ
WSB/EiJ
Mean value
25
20
15
10
5
2013−01−15
2013−04−16
2013−07−16
2013−10−14
2014−01−13
Animal shipping date

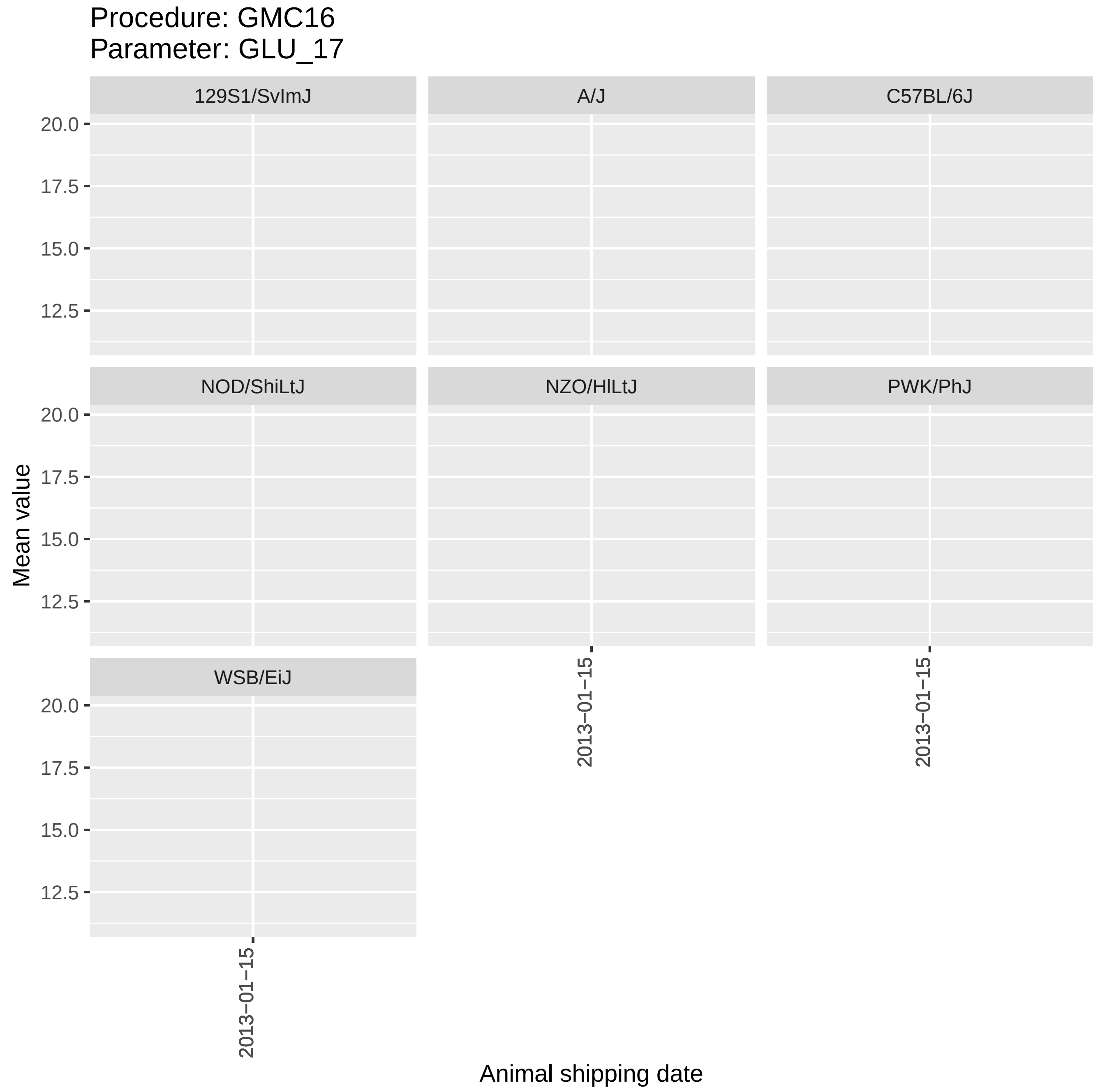

Procedure: GMC16
Parameter: GLU_17
129S1/SvImJ
A/J
C57BL/6J
NOD/ShiLtJ
NZO/HlLtJ
PWK/PhJ
WSB/EiJ
20.0
17.5
15.0
12.5
Mean value
2013−01−15
Animal shipping date

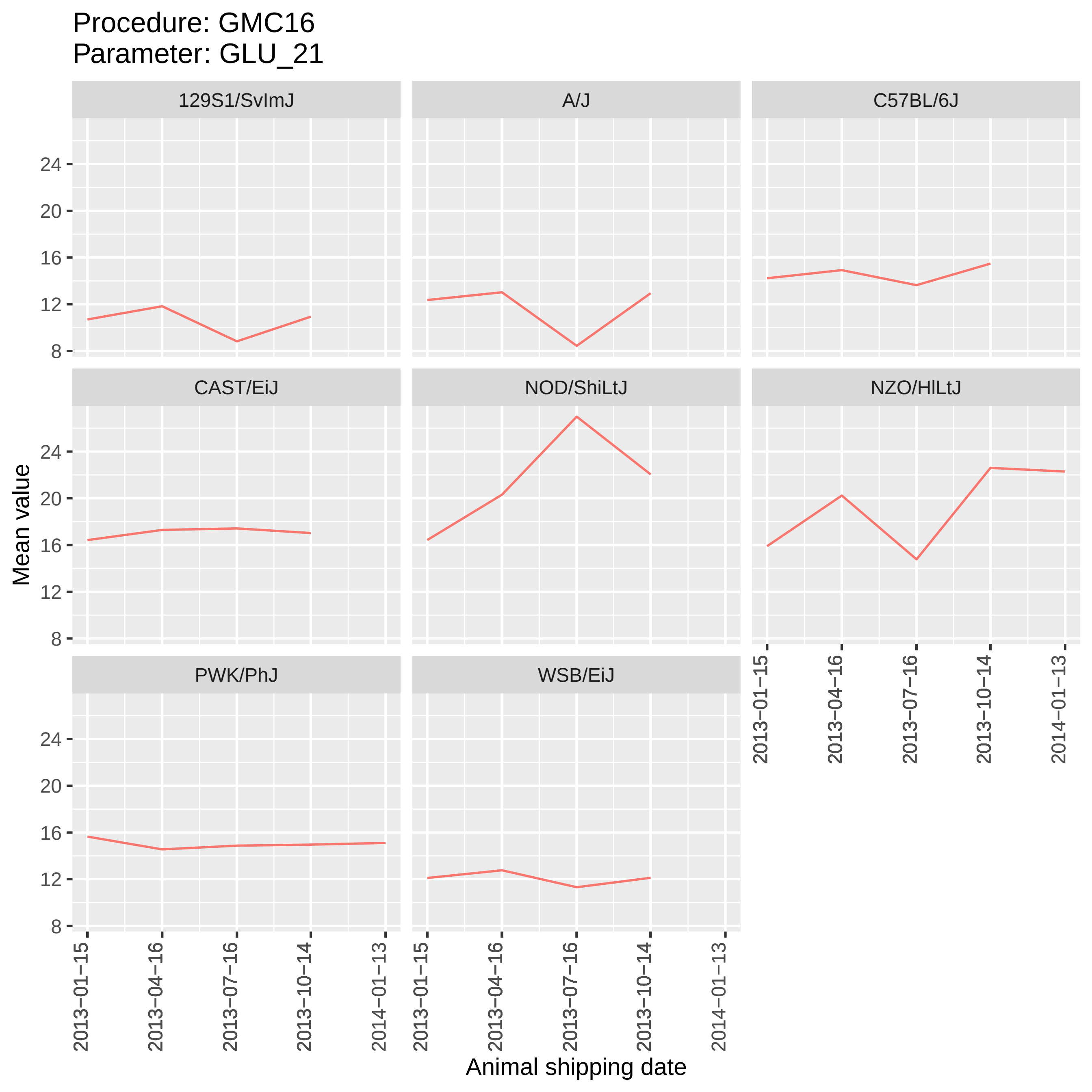

Procedure: GMC16
Parameter: GLU_21
129S1/SvImJ
A/J
C57BL/6J
CAST/EiJ
NOD/ShiLtJ
NZO/HlLtJ
PWK/PhJ
WSB/EiJ
Mean value
8
12
16
20
24
2013−01−15
2013−04−16
2013−07−16
2013−10−14
2014−01−13
Animal shipping date

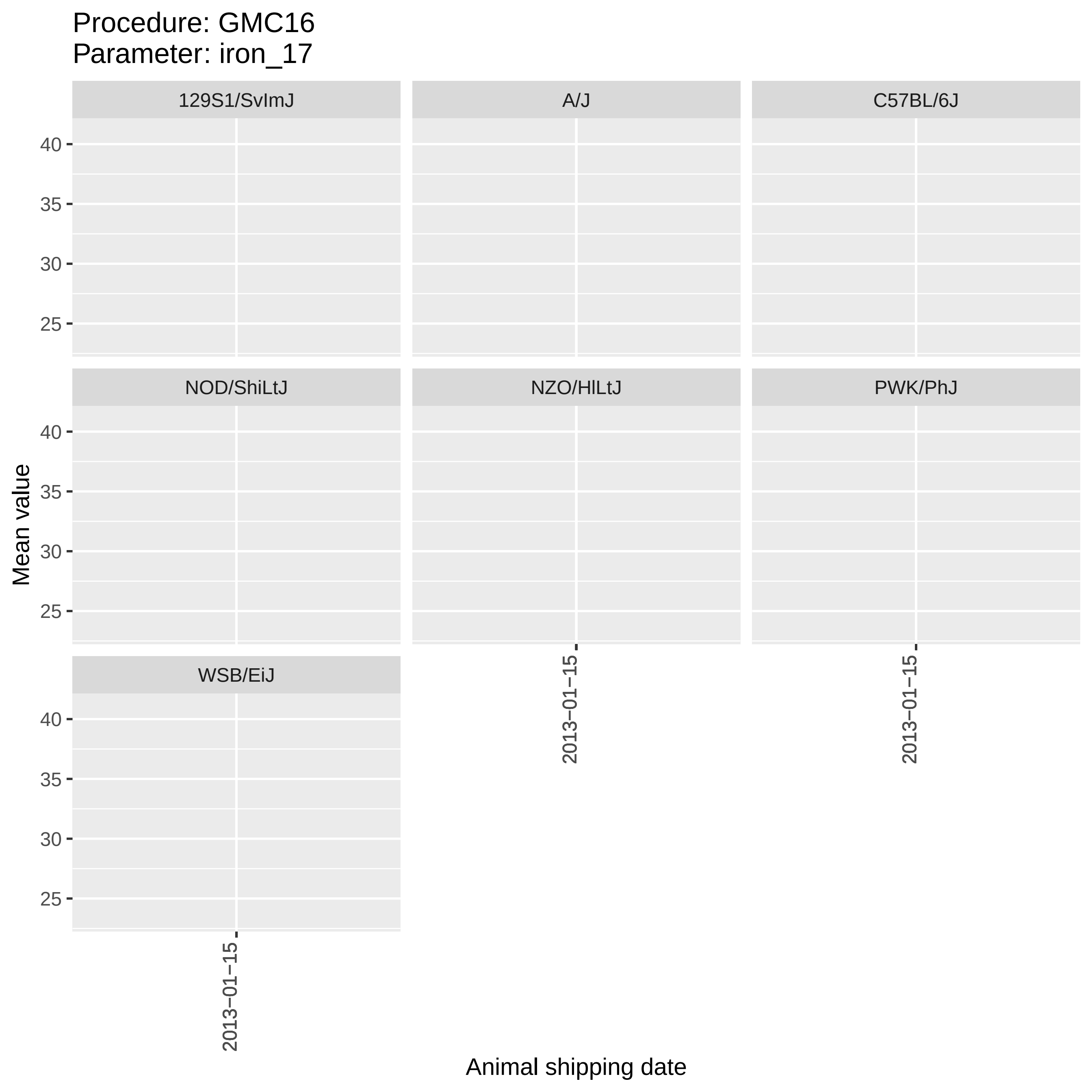

Procedure: GMC16
Parameter: iron_17
129S1/SvImJ
A/J
C57BL/6J
NOD/ShiLtJ
NZO/HlLtJ
PWK/PhJ
WSB/EiJ
40
35
30
25
Mean value
2013−01−15
Animal shipping date

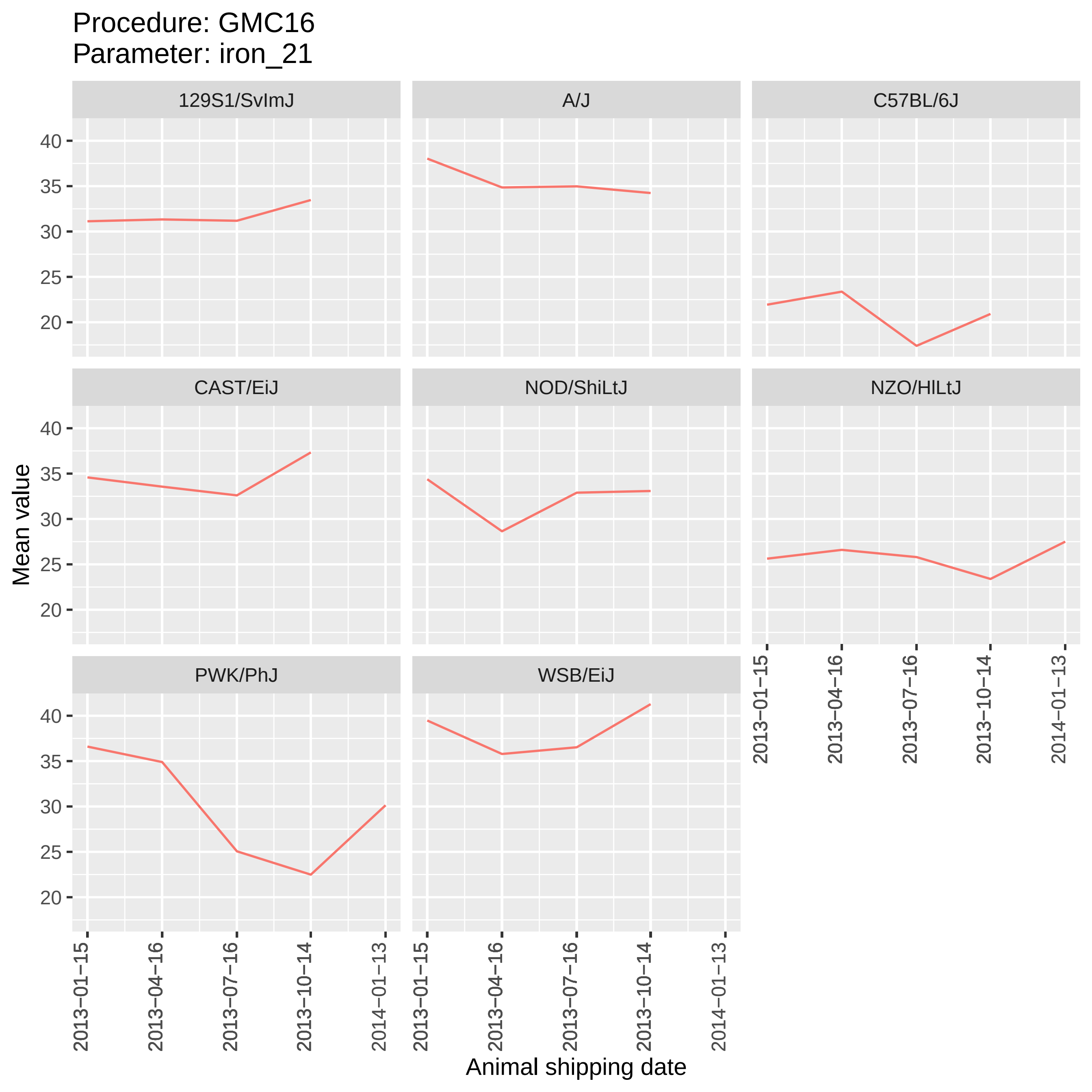

Procedure: GMC16
Parameter: iron_21
129S1/SvImJ
A/J
C57BL/6J
CAST/EiJ
NOD/ShiLtJ
NZO/HlLtJ
PWK/PhJ
WSB/EiJ
Mean value
20
25
30
35
40
2013−01−15
2013−04−16
2013−07−16
2013−10−14
2014−01−13
Animal shipping date

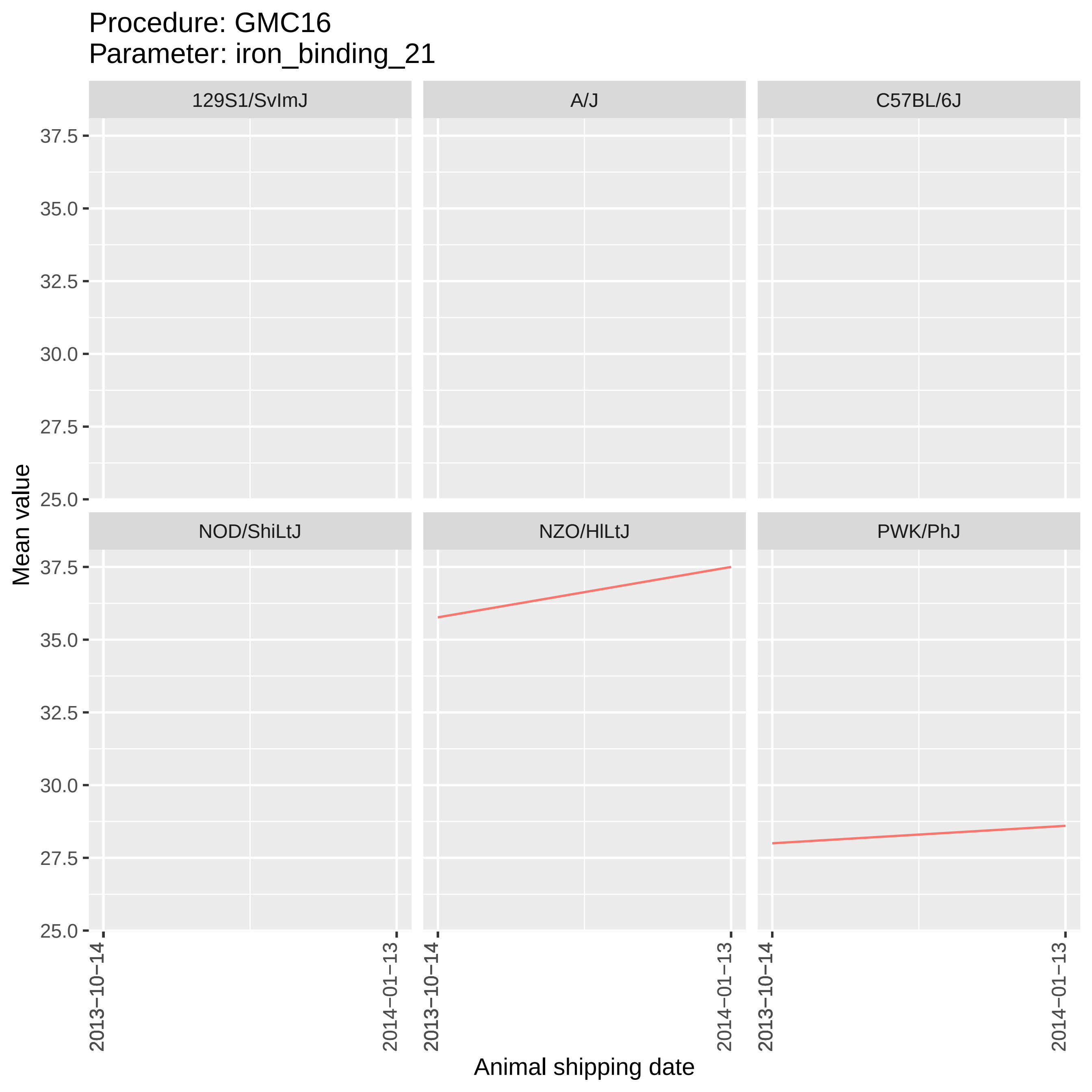

Procedure: GMC16
Parameter: iron_binding_21
129S1/SvImJ
A/J
C57BL/6J
NOD/ShiLtJ
NZO/HlLtJ
PWK/PhJ
Mean value
37.5
35.0
32.5
30.0
27.5
25.0
2013−10−14
2014−01−13
Animal shipping date

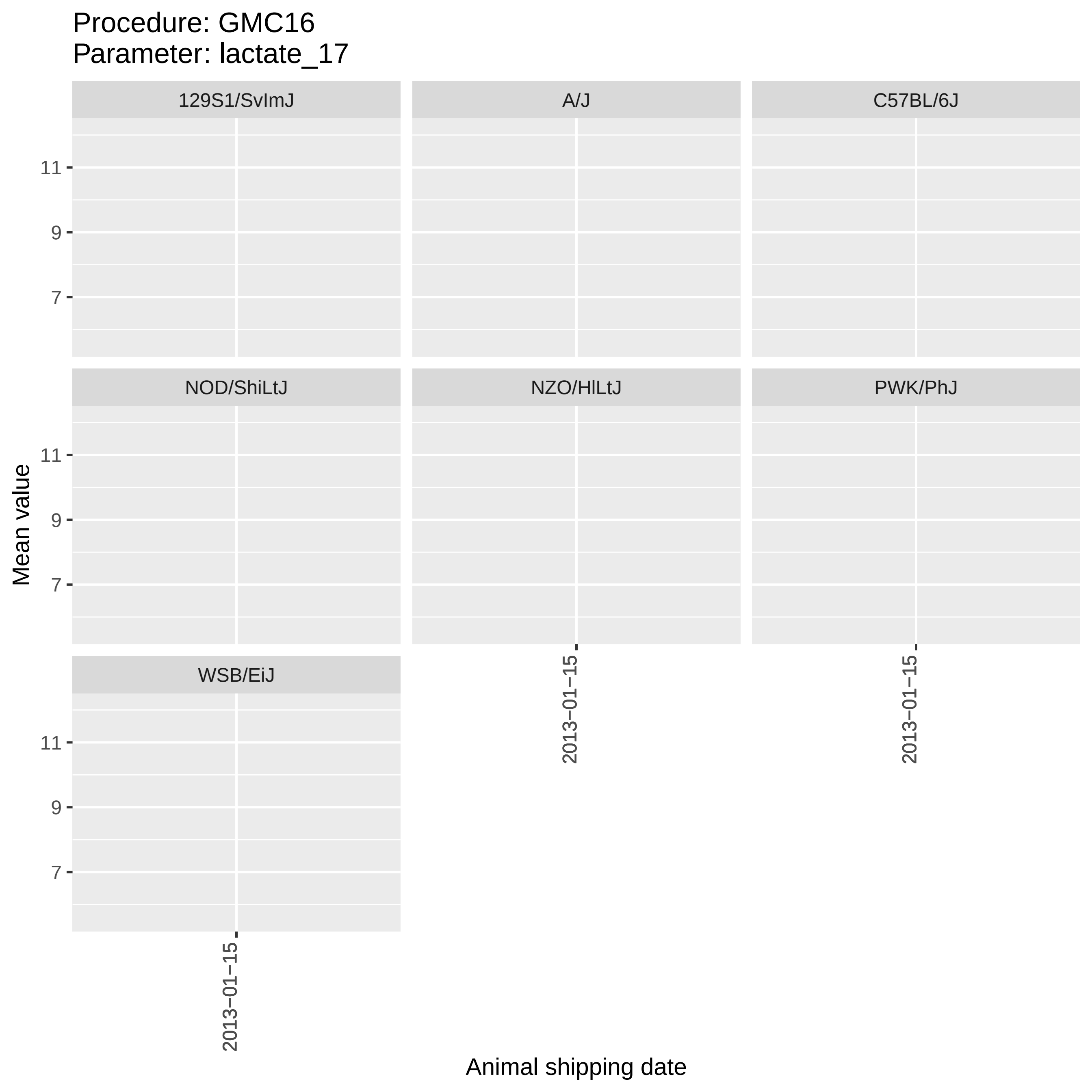
Procedure: GMC16
Parameter: lactate_17
129S1/SvImJ
A/J
C57BL/6J
NOD/ShiLtJ
NZO/HlLtJ
PWK/PhJ
WSB/EiJ
11
9
7
Mean value
2013−01−15
Animal shipping date

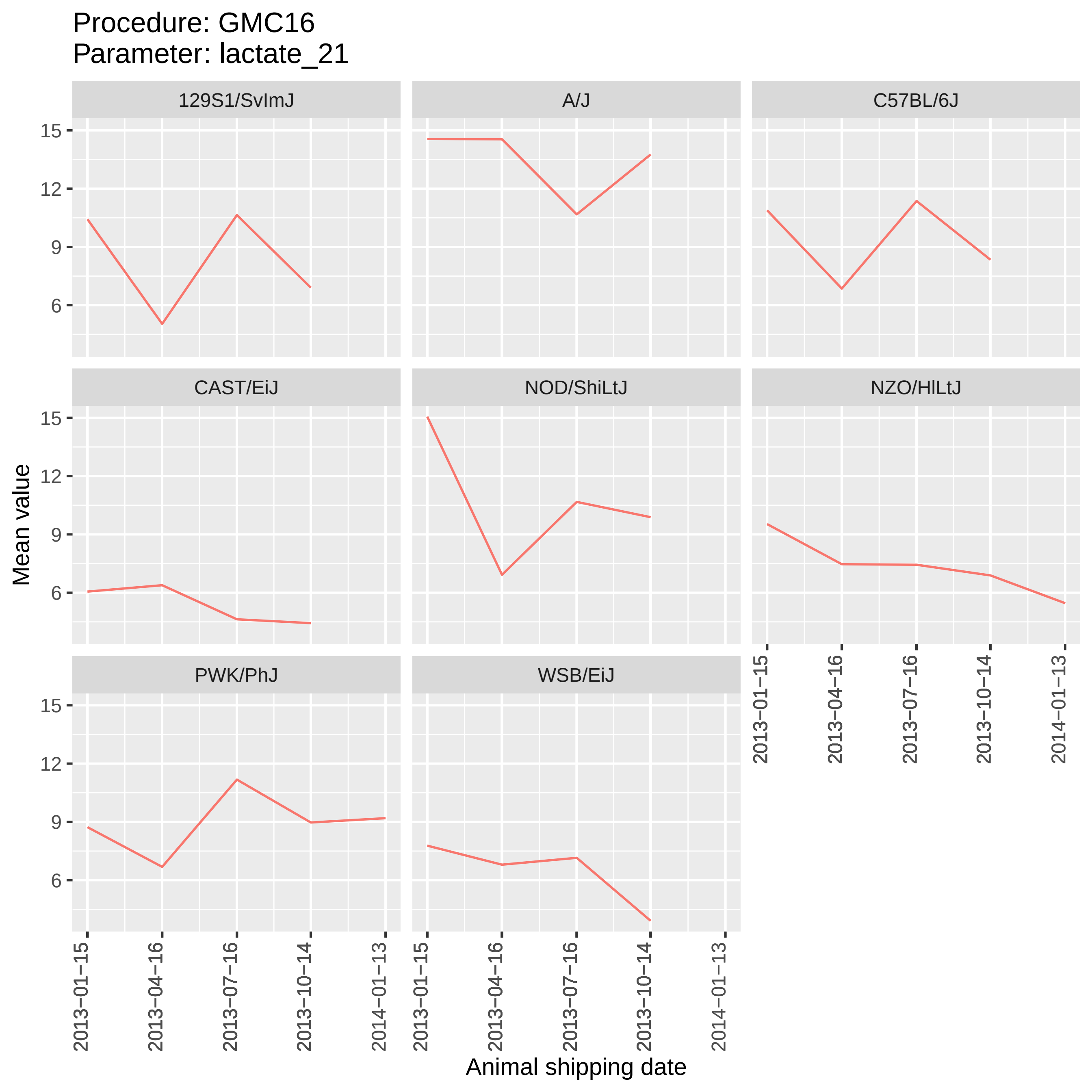

Procedure: GMC16
Parameter: lactate_21
129S1/SvImJ
A/J
C57BL/6J
CAST/EiJ
NOD/ShiLtJ
NZO/HlLtJ
PWK/PhJ
WSB/EiJ
Mean value
15
12
9
6
2013−01−15
2013−04−16
2013−07−16
2013−10−14
2014−01−13
Animal shipping date

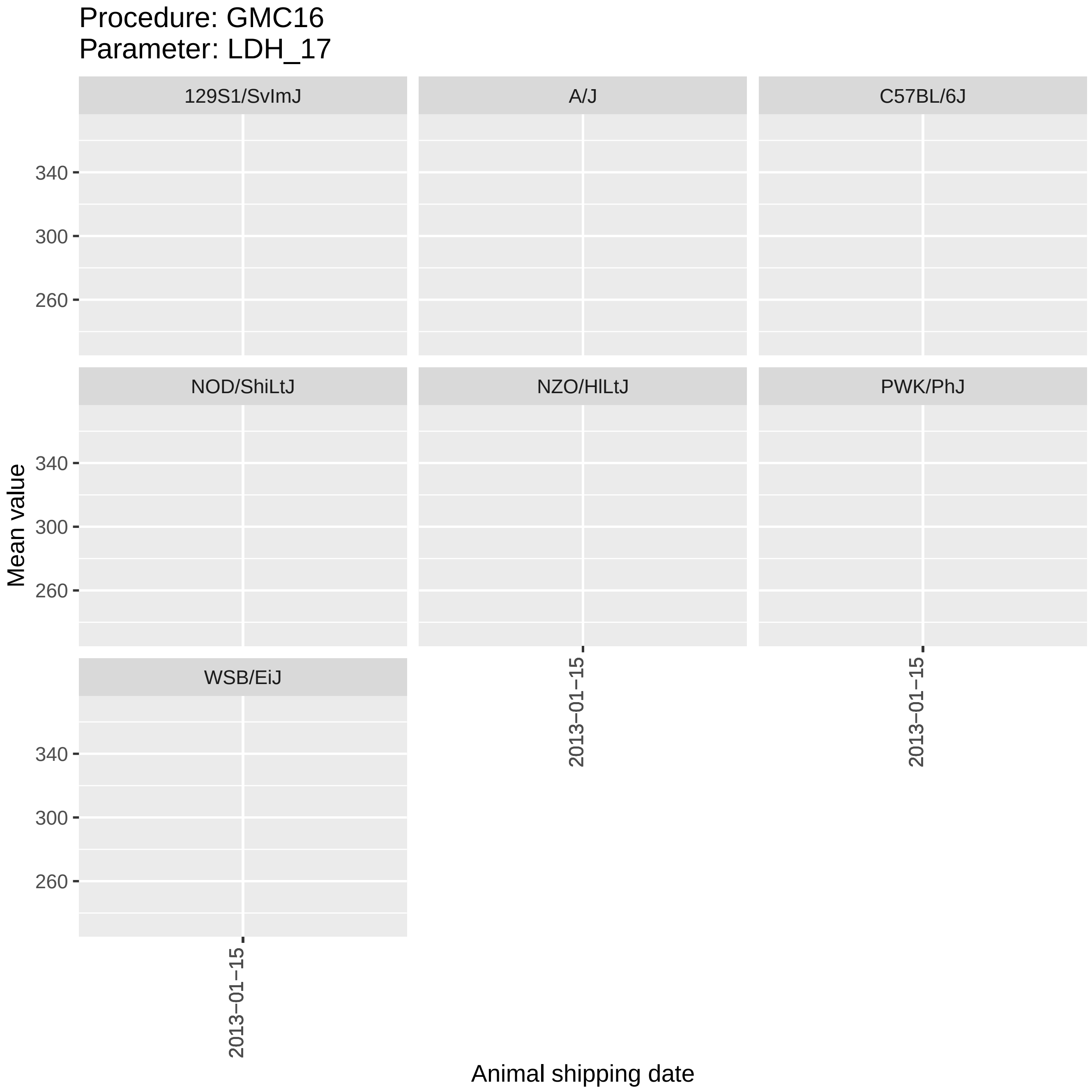
Procedure: GMC16
Parameter: LDH_17
129S1/SvImJ
A/J
C57BL/6J
NOD/ShiLtJ
NZO/HlLtJ
PWK/PhJ
WSB/EiJ
Mean value
340
300
260
2013−01−15
Animal shipping date

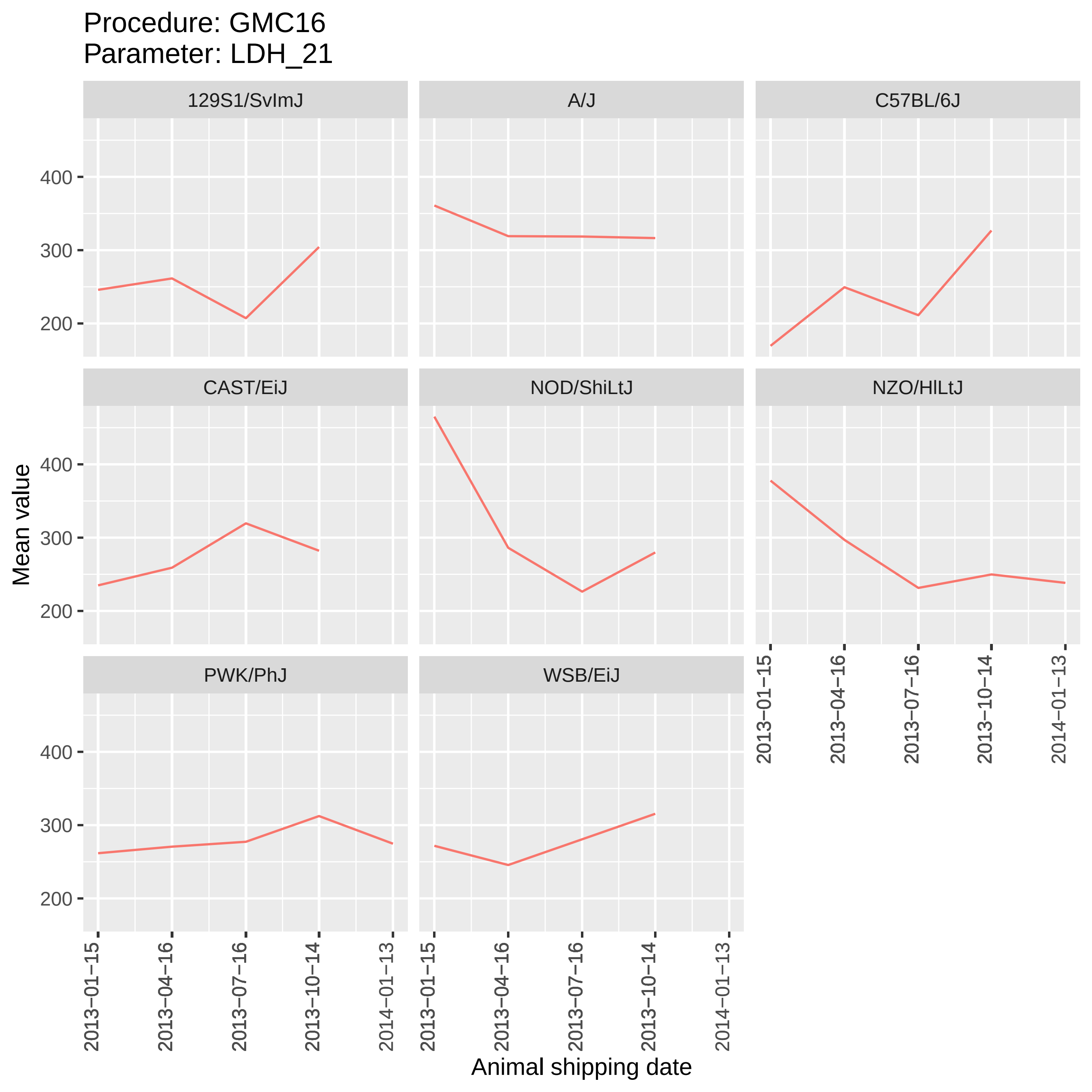

Procedure: GMC16
Parameter: LDH_21
129S1/SvImJ
A/J
C57BL/6J
CAST/EiJ
NOD/ShiLtJ
NZO/HlLtJ
PWK/PhJ
WSB/EiJ
Mean value
200
300
400
2013−01−15
2013−04−16
2013−07−16
2013−10−14
2014−01−13
Animal shipping date

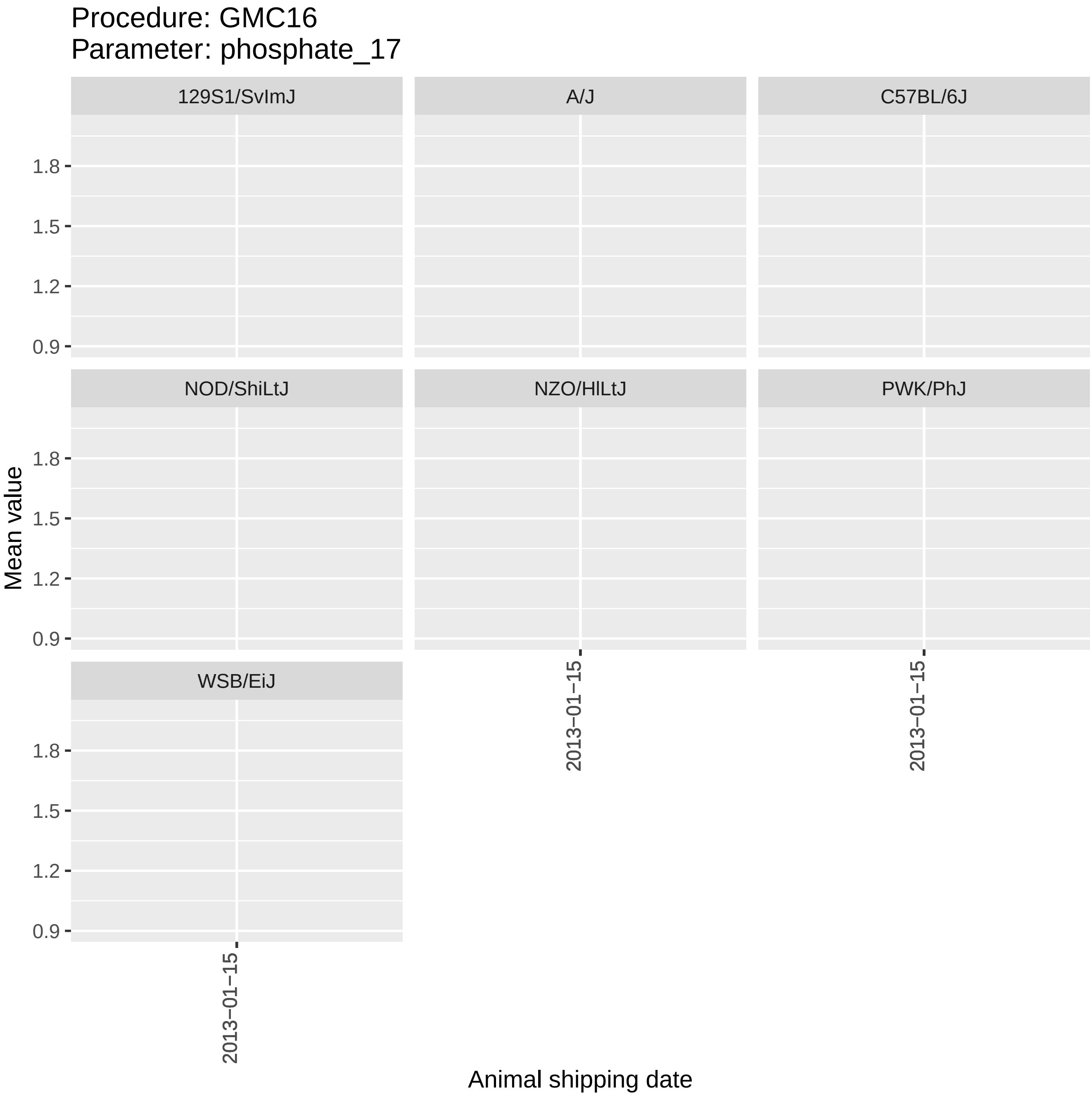
Procedure: GMC16
Parameter: phosphate_17
129S1/SvImJ
A/J
C57BL/6J
NOD/ShiLtJ
NZO/HlLtJ
PWK/PhJ
WSB/EiJ
1.8
1.5
1.2
0.9
Mean value
2013−01−15
Animal shipping date

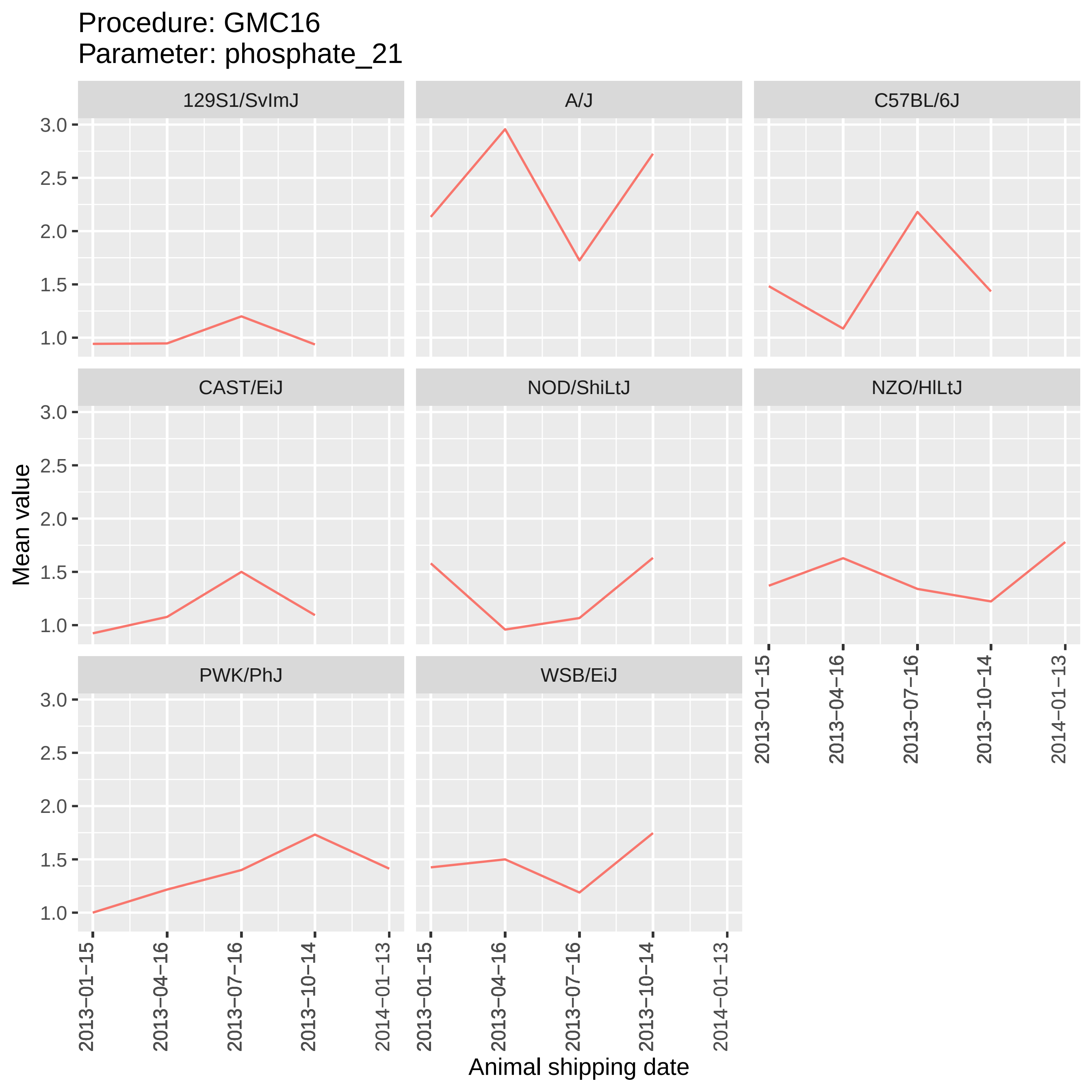

Procedure: GMC16
Parameter: phosphate_21
129S1/SvImJ
A/J
C57BL/6J
CAST/EiJ
NOD/ShiLtJ
NZO/HlLtJ
PWK/PhJ
WSB/EiJ
Mean value
1.0
1.5
2.0
2.5
3.0
2013−01−15
2013−04−16
2013−07−16
2013−10−14
2014−01−13
Animal shipping date

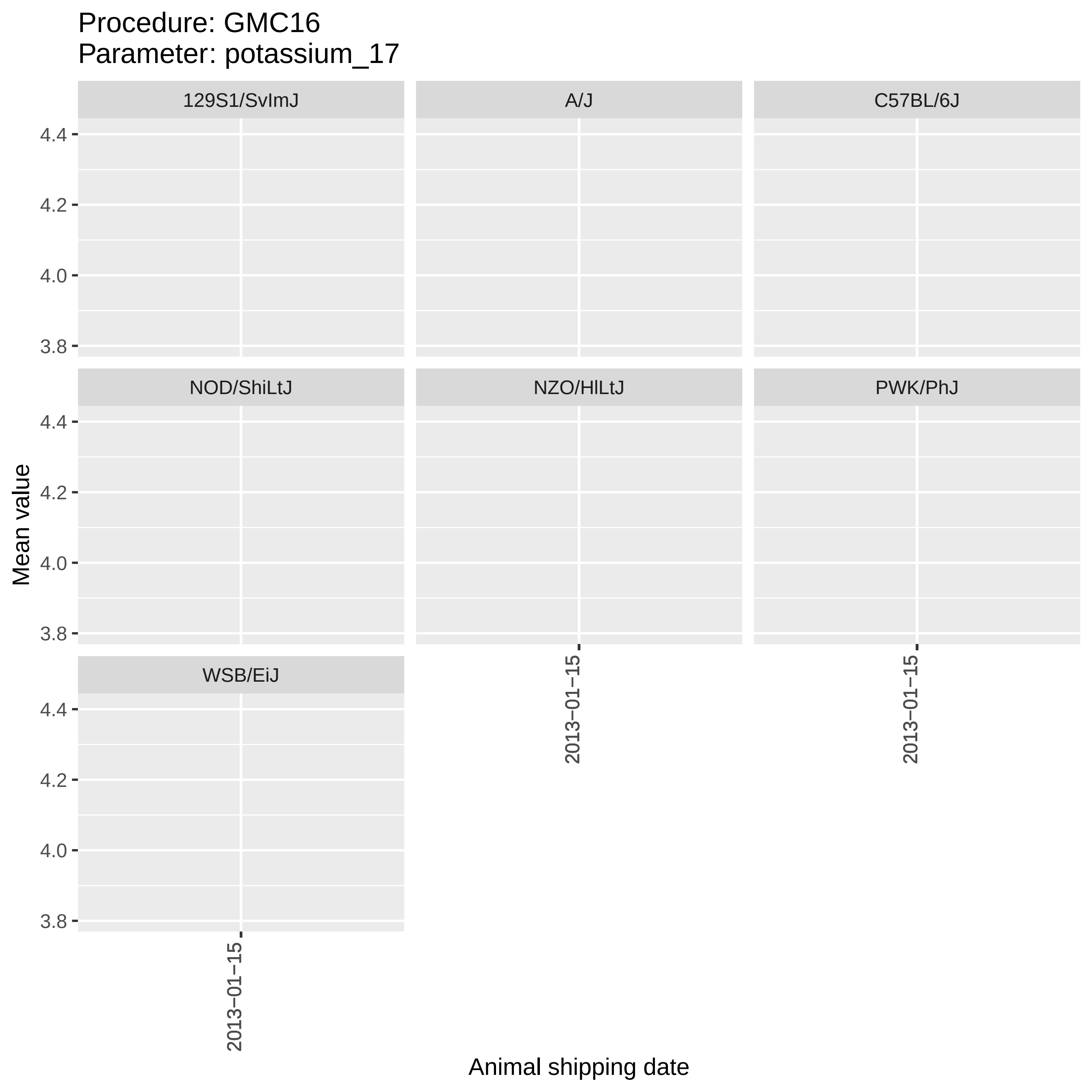

Procedure: GMC16
Parameter: potassium_17
129S1/SvImJ
A/J
C57BL/6J
NOD/ShiLtJ
NZO/HlLtJ
PWK/PhJ
WSB/EiJ
4.4
4.2
4.0
3.8
Mean value
2013−01−15
Animal shipping date

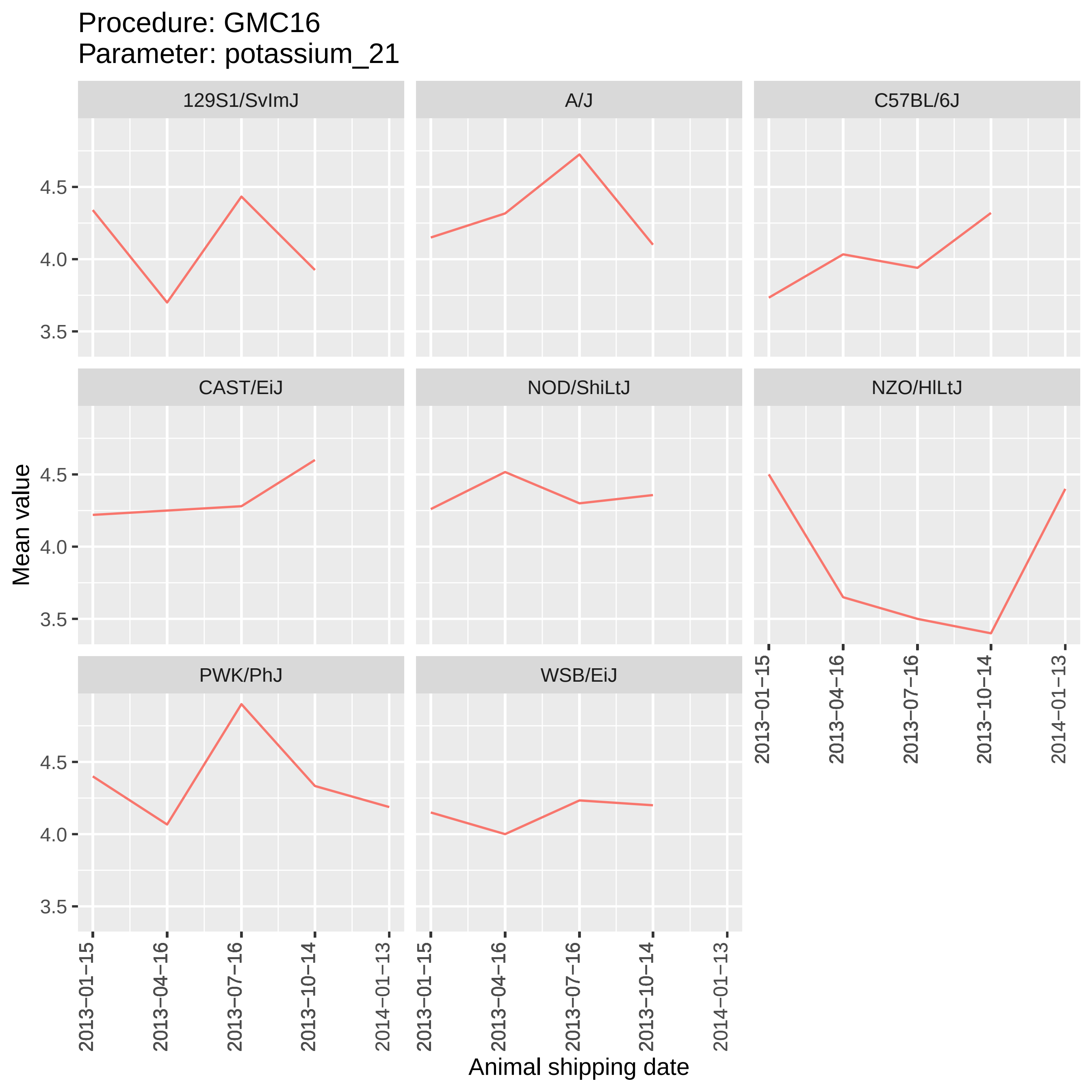

Procedure: GMC16
Parameter: potassium_21
129S1/SvImJ
A/J
C57BL/6J
CAST/EiJ
NOD/ShiLtJ
NZO/HlLtJ
PWK/PhJ
WSB/EiJ
Mean value
3.5
4.0
4.5
2013−01−15
2013−04−16
2013−07−16
2013−10−14
2014−01−13
Animal shipping date

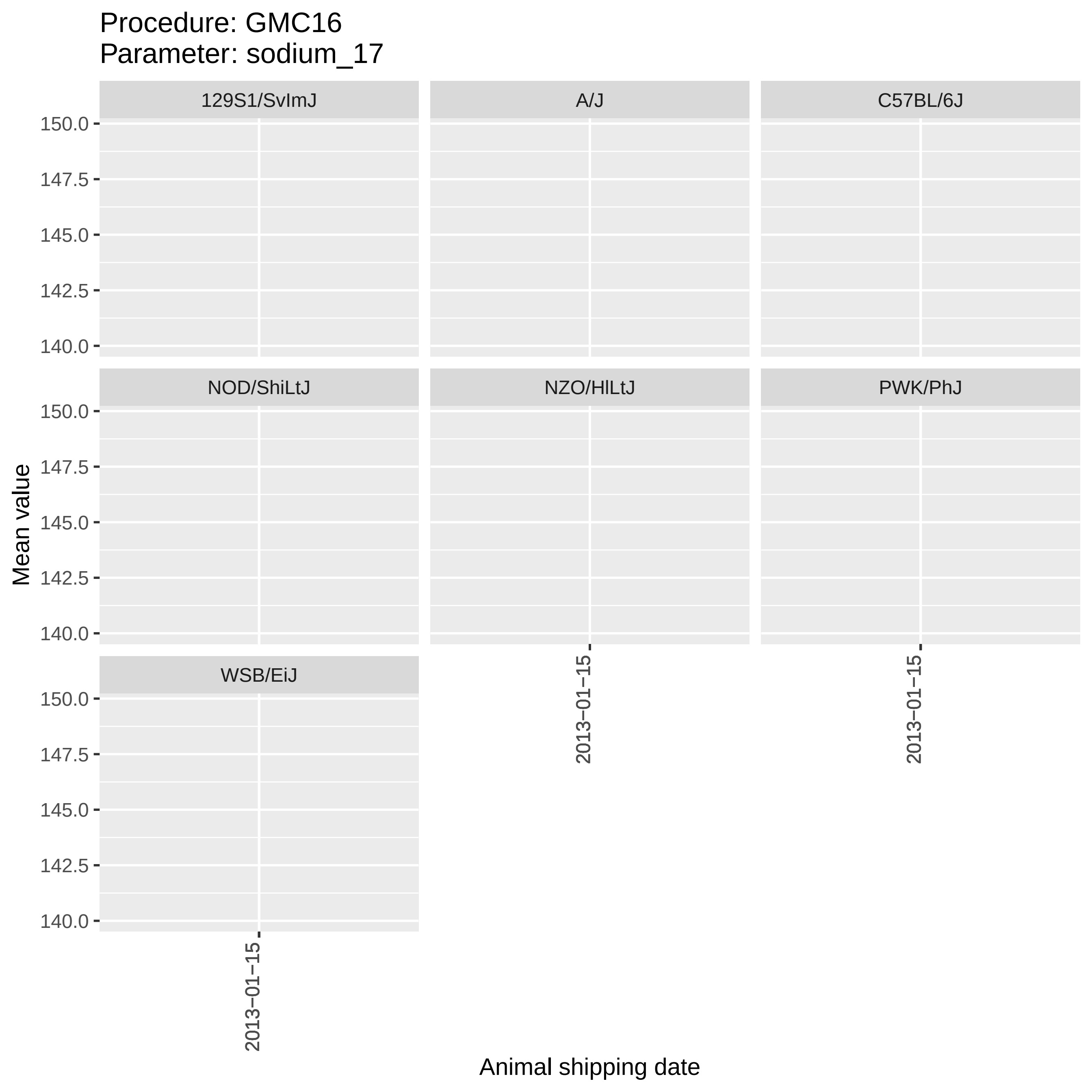

Procedure: GMC16
Parameter: sodium_17
129S1/SvImJ
A/J
C57BL/6J
NOD/ShiLtJ
NZO/HlLtJ
PWK/PhJ
WSB/EiJ
150.0
147.5
145.0
142.5
140.0
Mean value
2013−01−15
Animal shipping date

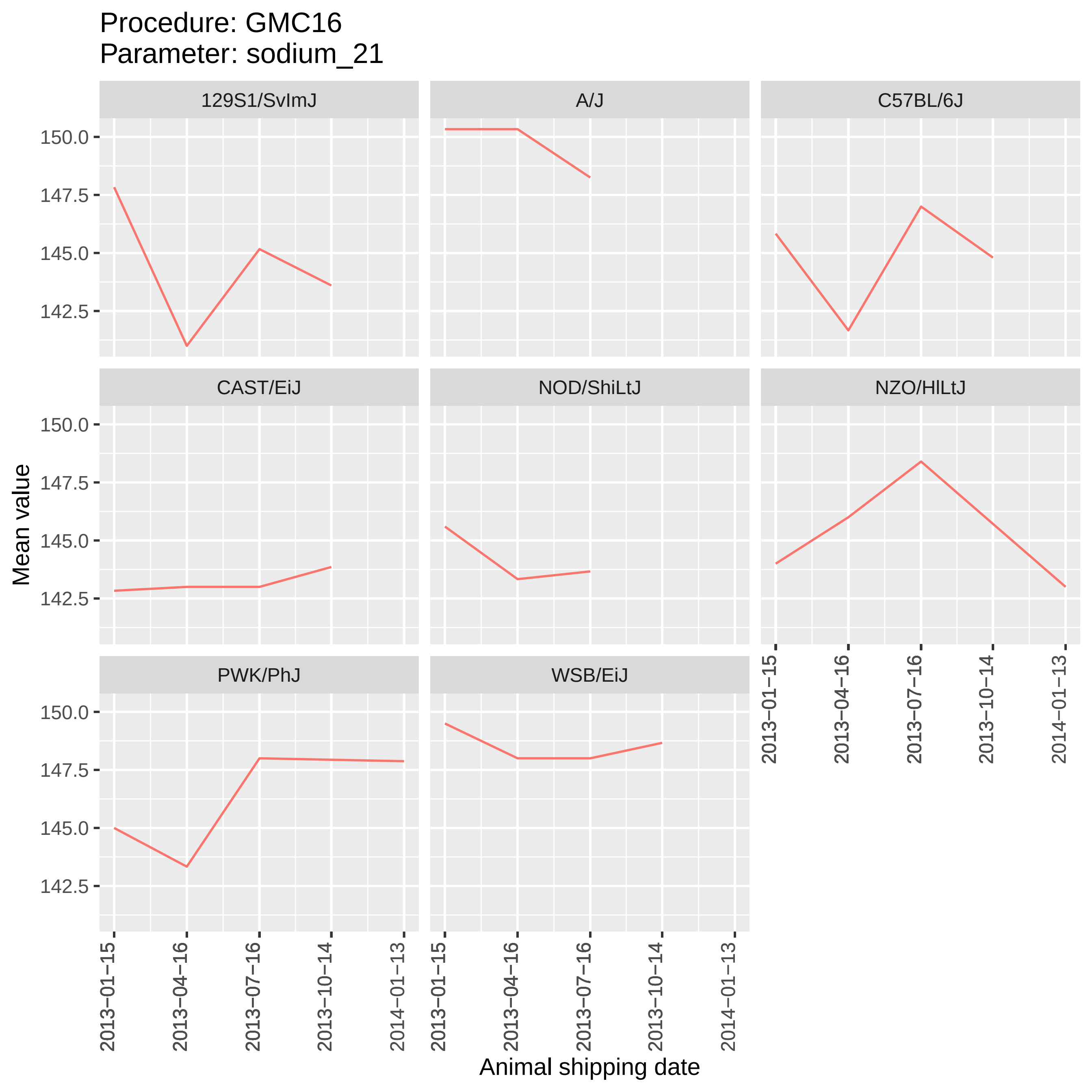

Procedure: GMC16
Parameter: sodium_21
129S1/SvImJ
A/J
C57BL/6J
CAST/EiJ
NOD/ShiLtJ
NZO/HlLtJ
PWK/PhJ
WSB/EiJ
150.0
147.5
145.0
142.5
Mean value
2013−01−15
2013−04−16
2013−07−16
2013−10−14
2014−01−13
Animal shipping date

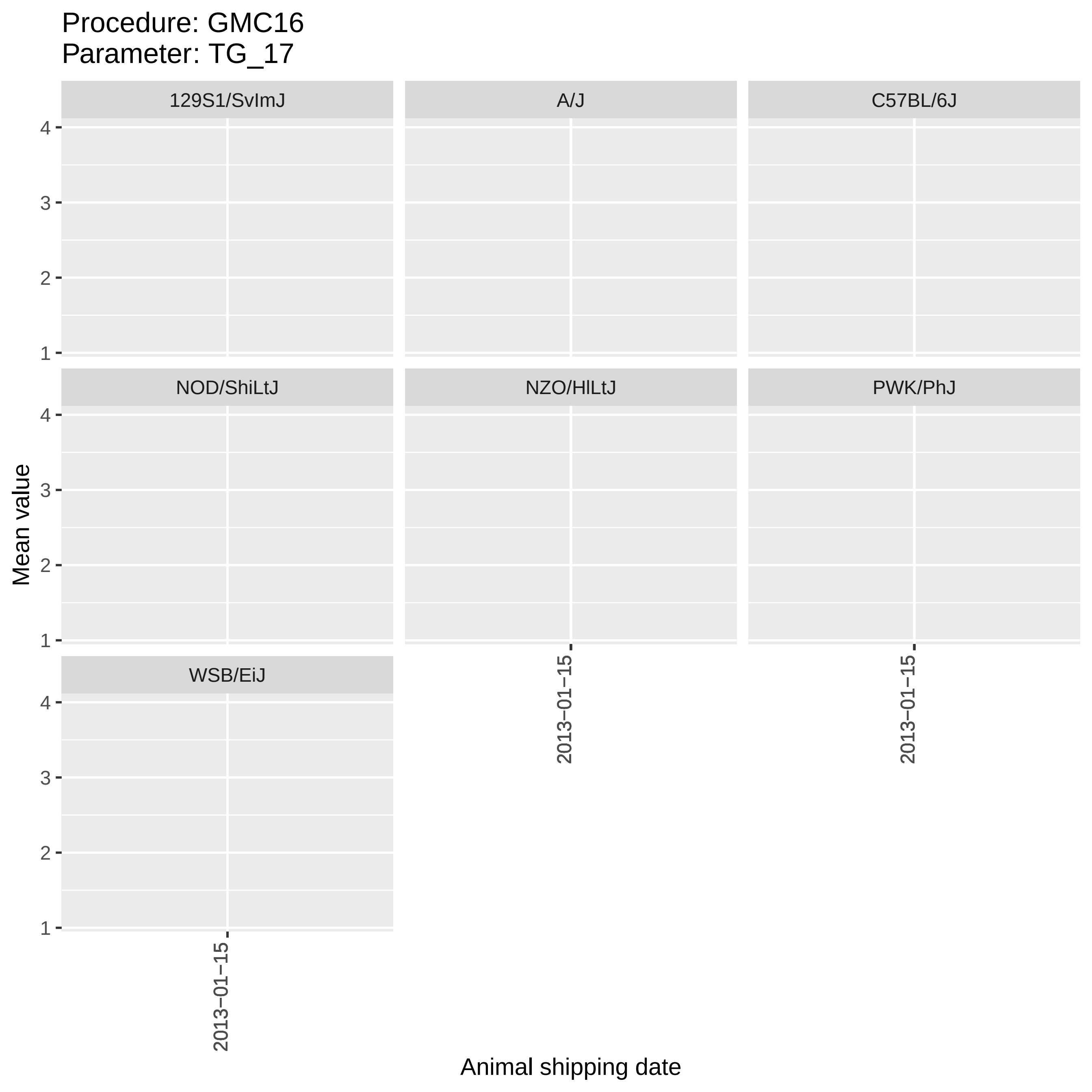
Procedure: GMC16
Parameter: TG_17
129S1/SvImJ
A/J
C57BL/6J
NOD/ShiLtJ
NZO/HlLtJ
PWK/PhJ
WSB/EiJ
Mean value
1
2
3
4
2013−01−15
Animal shipping date

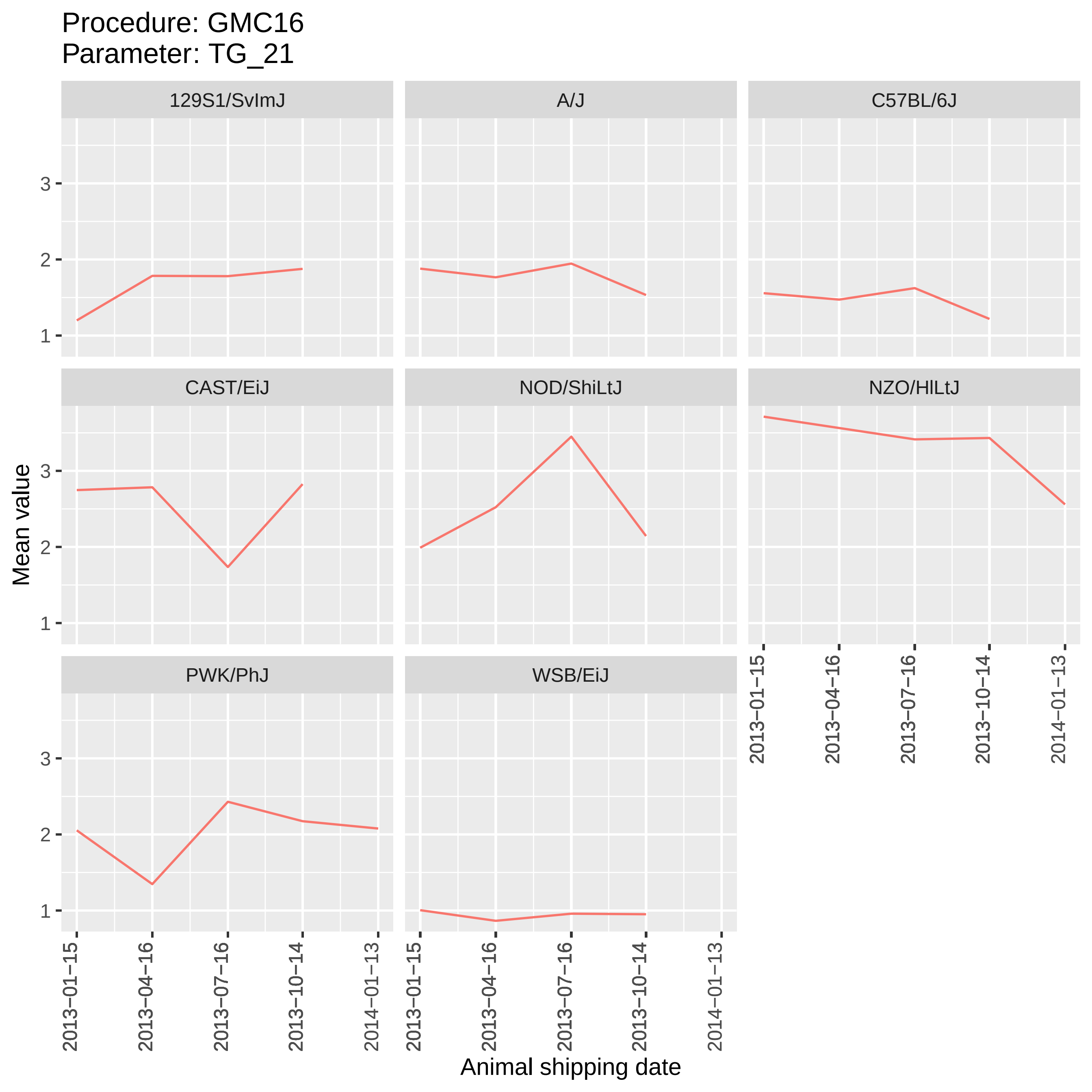

Procedure: GMC16
Parameter: TG_21
129S1/SvImJ
A/J
C57BL/6J
CAST/EiJ
NOD/ShiLtJ
NZO/HlLtJ
PWK/PhJ
WSB/EiJ
Mean value
1
2
3
2013−01−15
2013−04−16
2013−07−16
2013−10−14
2014−01−13
Animal shipping date

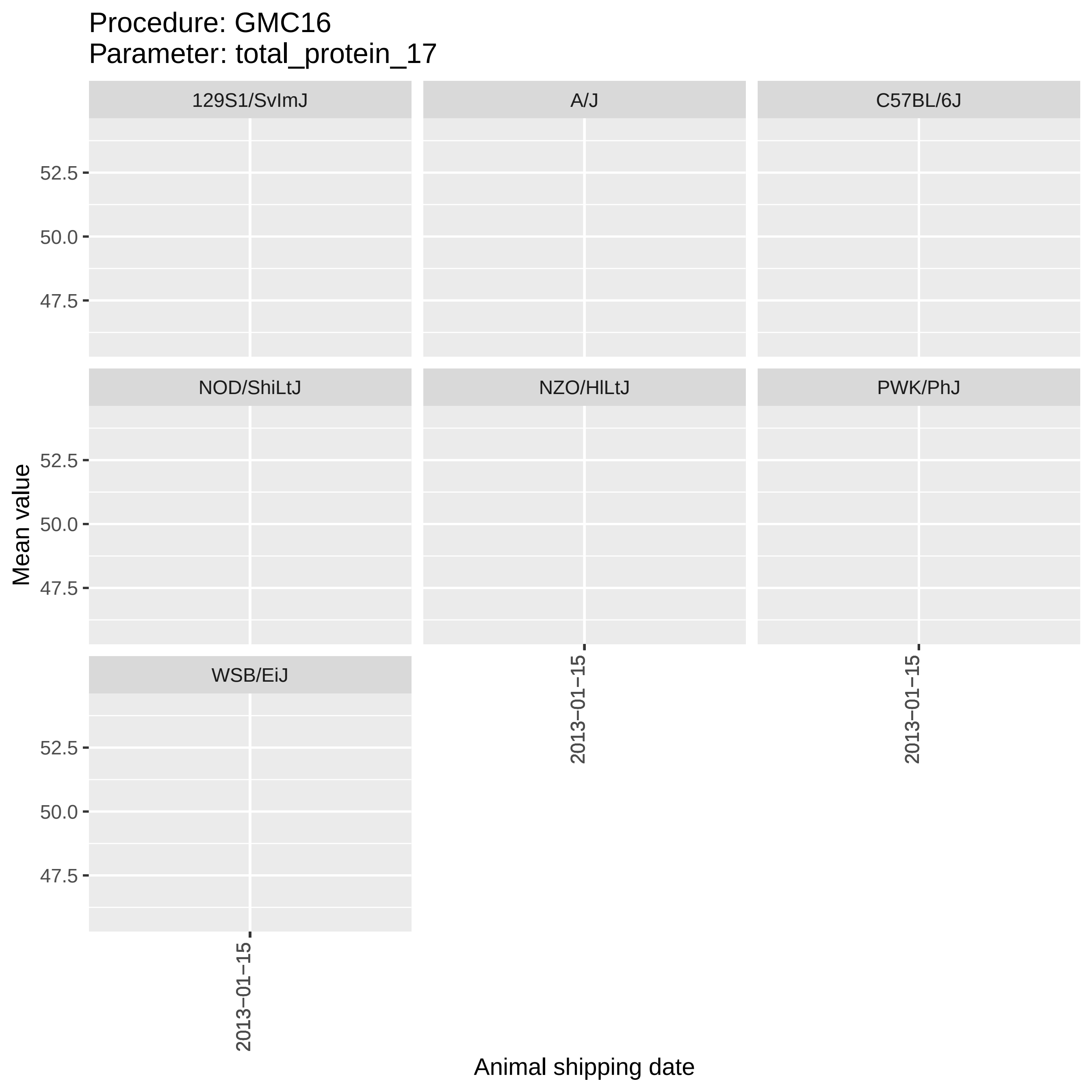
Procedure: GMC16
Parameter: total_protein_17
129S1/SvImJ
A/J
C57BL/6J
NOD/ShiLtJ
NZO/HlLtJ
PWK/PhJ
WSB/EiJ
52.5
50.0
47.5
Mean value
2013−01−15
Animal shipping date

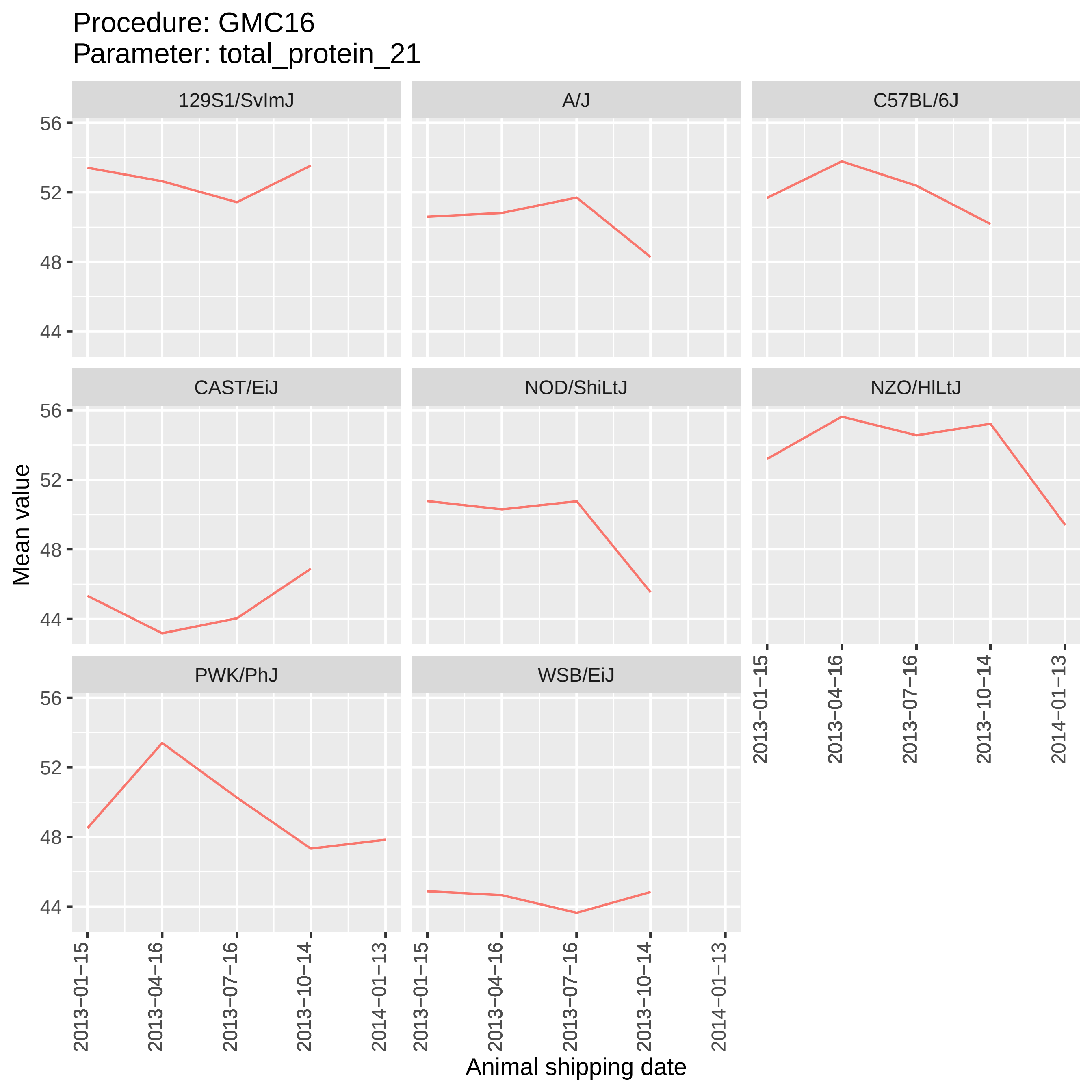

Procedure: GMC16
Parameter: total_protein_21
129S1/SvImJ
A/J
C57BL/6J
CAST/EiJ
NOD/ShiLtJ
NZO/HlLtJ
PWK/PhJ
WSB/EiJ
56
52
48
44
Mean value
2013−01−15
2013−04−16
2013−07−16
2013−10−14
2014−01−13
Animal shipping date

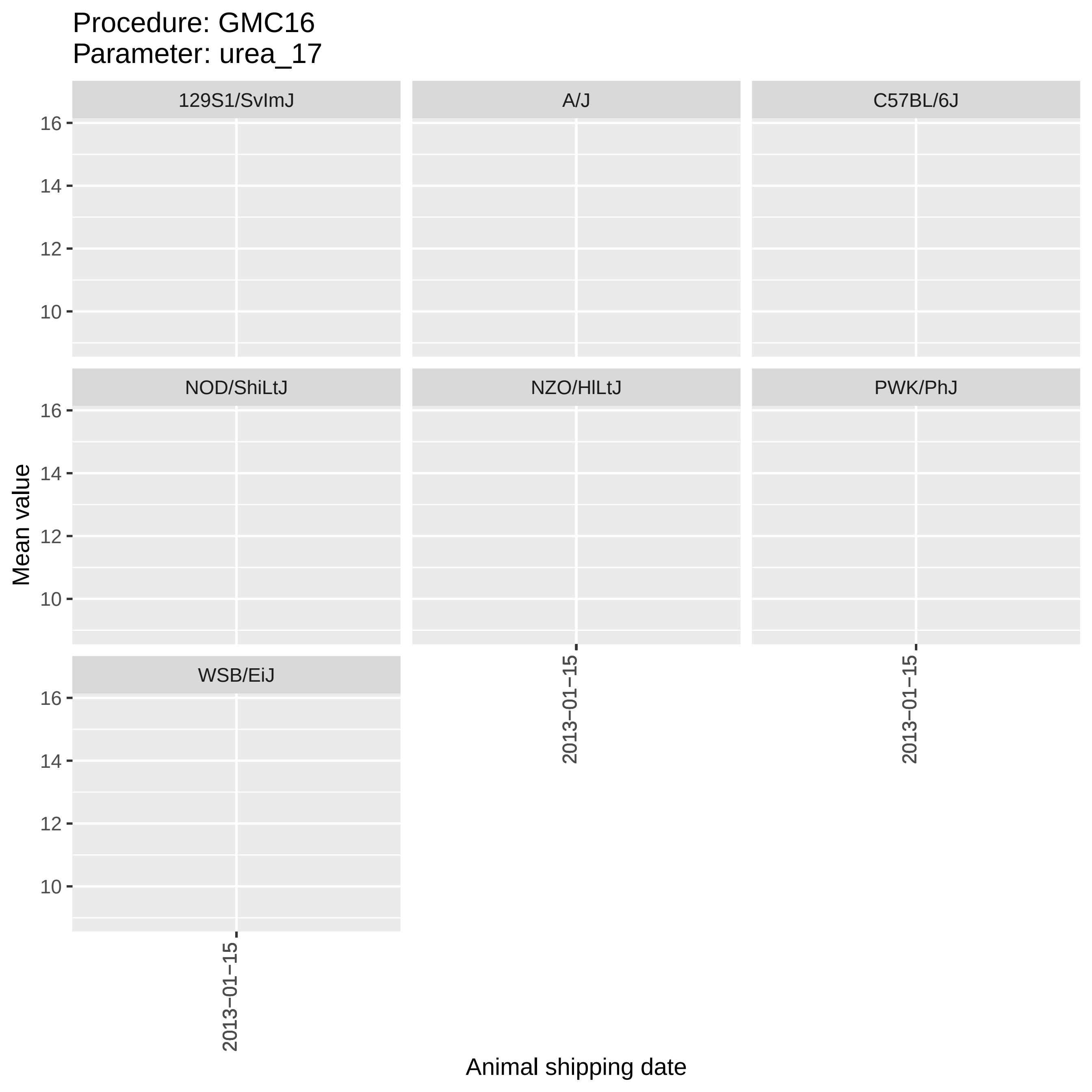

Procedure: GMC16
Parameter: urea_17
129S1/SvImJ
A/J
C57BL/6J
NOD/ShiLtJ
NZO/HlLtJ
PWK/PhJ
WSB/EiJ
16
14
12
10
Mean value
2013−01−15
Animal shipping date

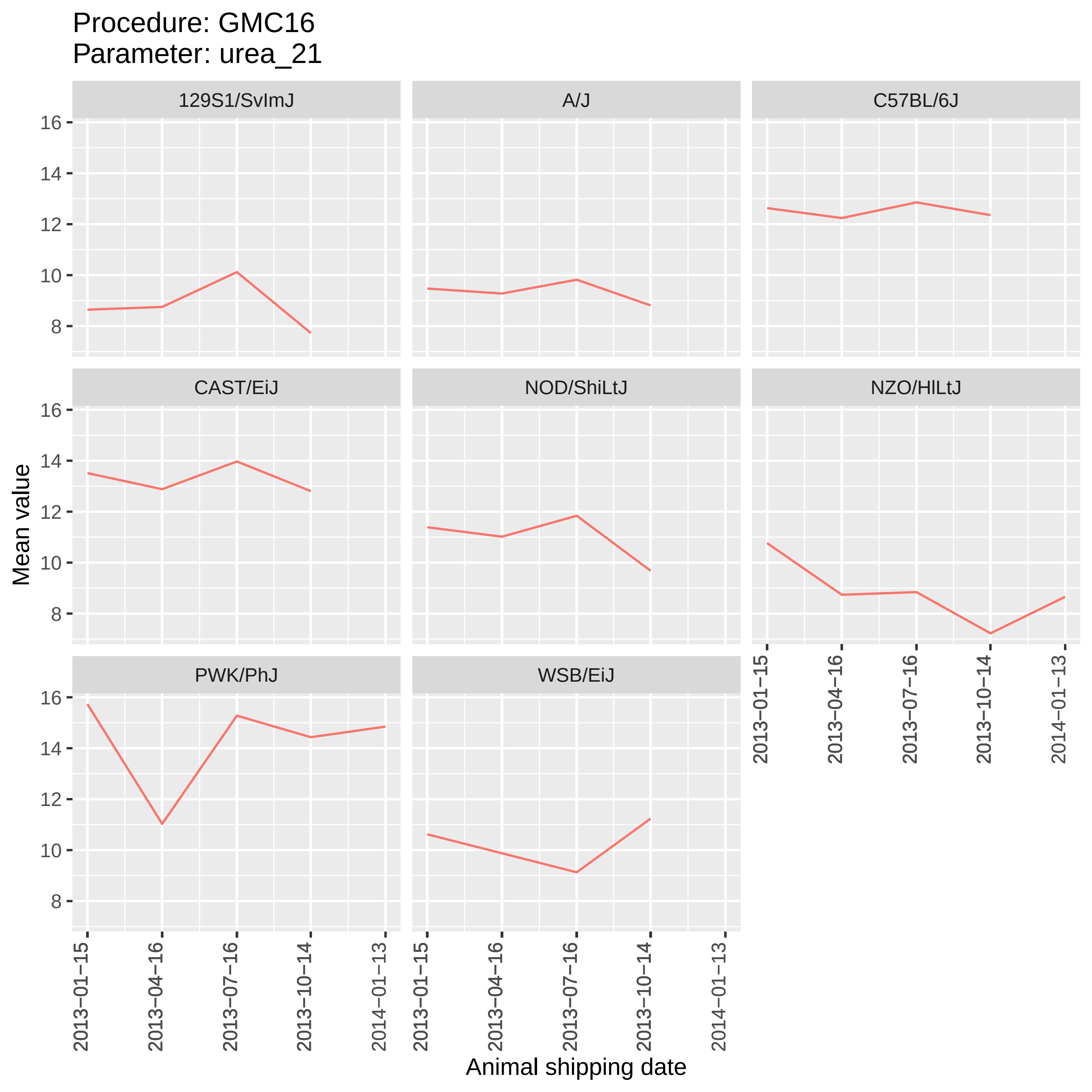

Procedure: GMC16
Parameter: urea_21
129S1/SvImJ
A/J
C57BL/6J
CAST/EiJ
NOD/ShiLtJ
NZO/HlLtJ
PWK/PhJ
WSB/EiJ
Mean value
8
10
12
14
16
2013−01−15
2013−04−16
2013−07−16
2013−10−14
2014−01−13
Animal shipping date

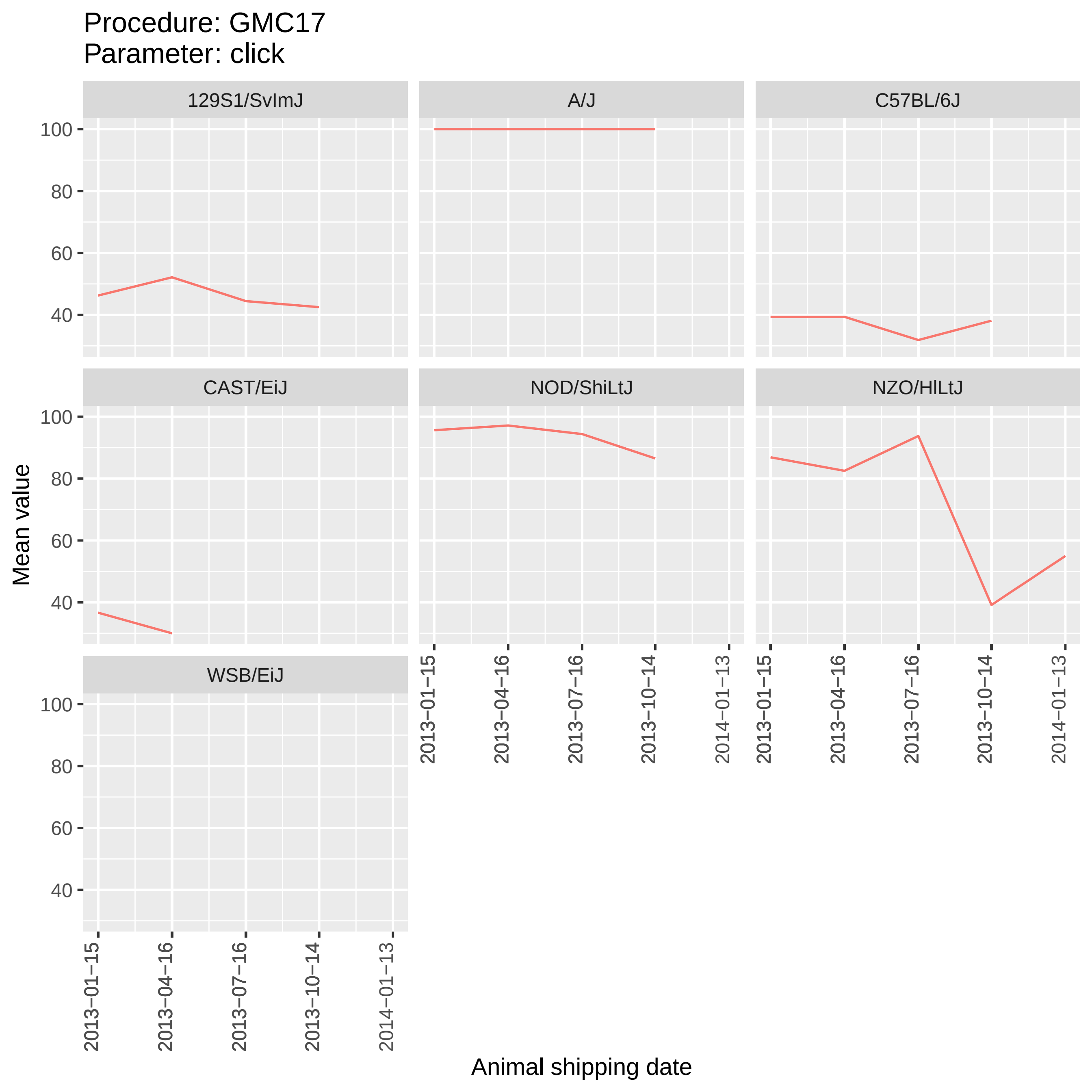

Procedure: GMC17
Parameter: click
129S1/SvImJ
A/J
C57BL/6J
CAST/EiJ
NOD/ShiLtJ
NZO/HlLtJ
WSB/EiJ
Mean value
100
80
60
40
2013−01−15
2013−04−16
2013−07−16
2013−10−14
2014−01−13
Animal shipping date

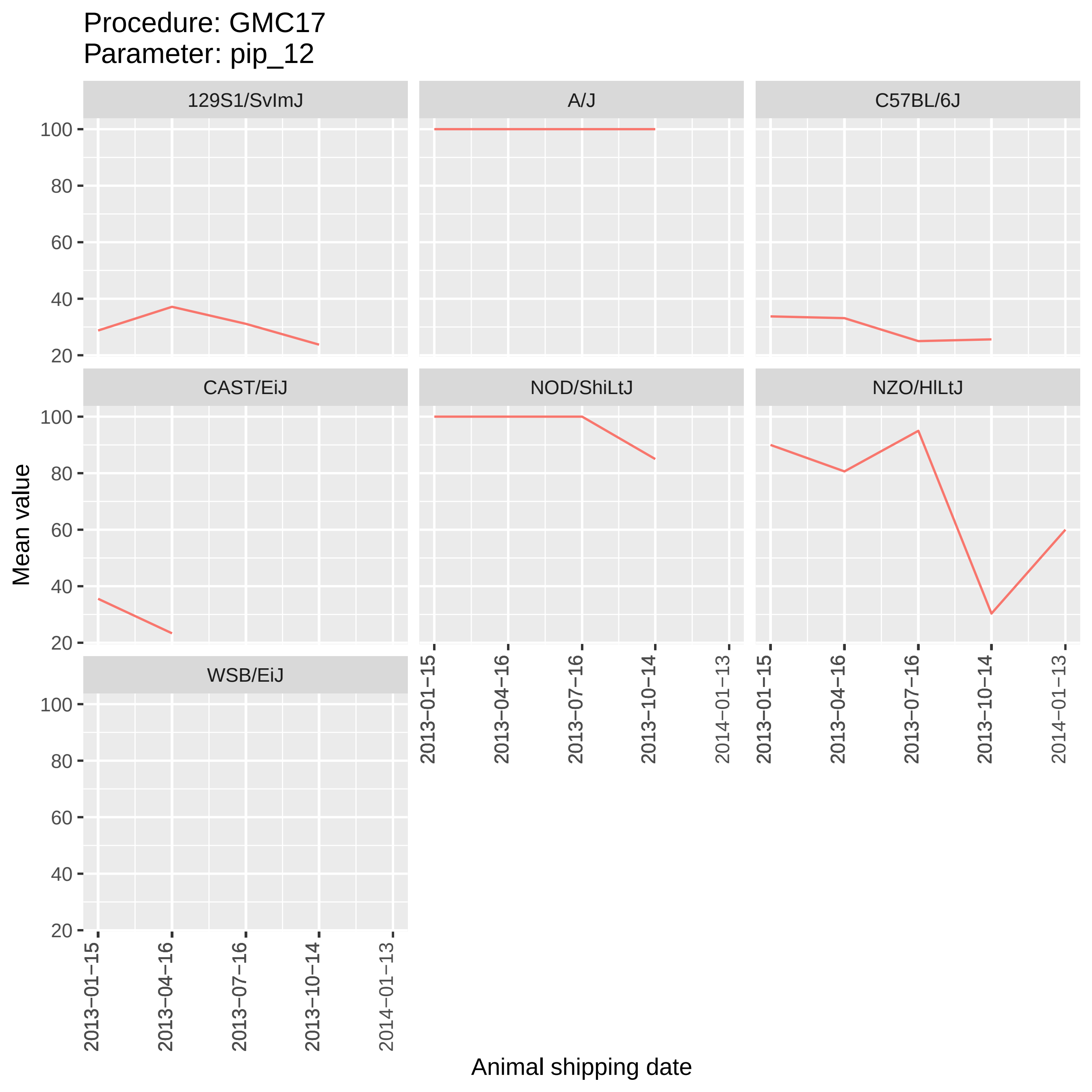

Procedure: GMC17
Parameter: pip_12
129S1/SvImJ
A/J
C57BL/6J
CAST/EiJ
NOD/ShiLtJ
NZO/HlLtJ
WSB/EiJ
Mean value
20
40
60
80
100
2013−01−15
2013−04−16
2013−07−16
2013−10−14
2014−01−13
Animal shipping date

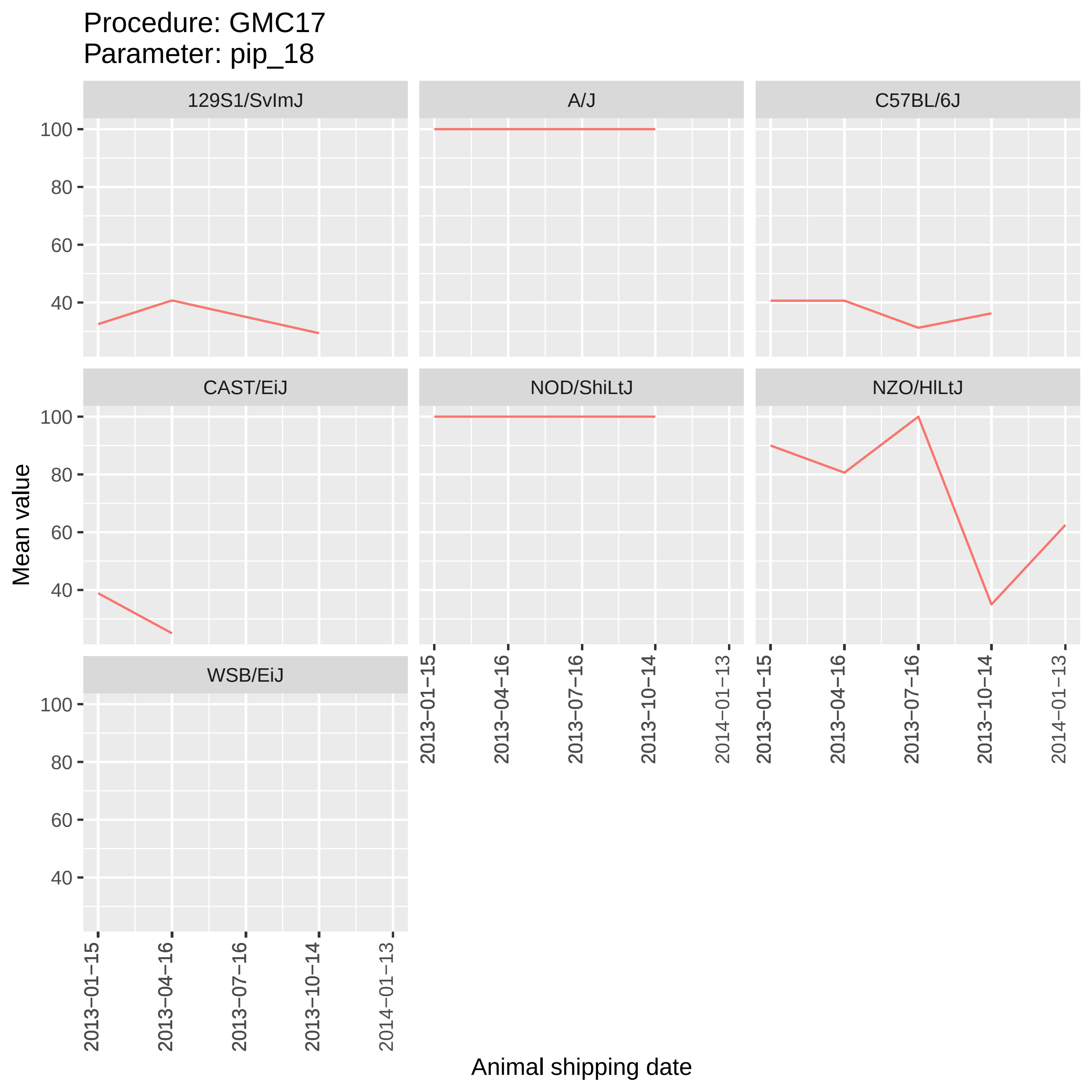

Procedure: GMC17
Parameter: pip_18
129S1/SvImJ
A/J
C57BL/6J
CAST/EiJ
NOD/ShiLtJ
NZO/HlLtJ
WSB/EiJ
Mean value
100
80
60
40
2013−01−15
2013−04−16
2013−07−16
2013−10−14
2014−01−13
Animal shipping date

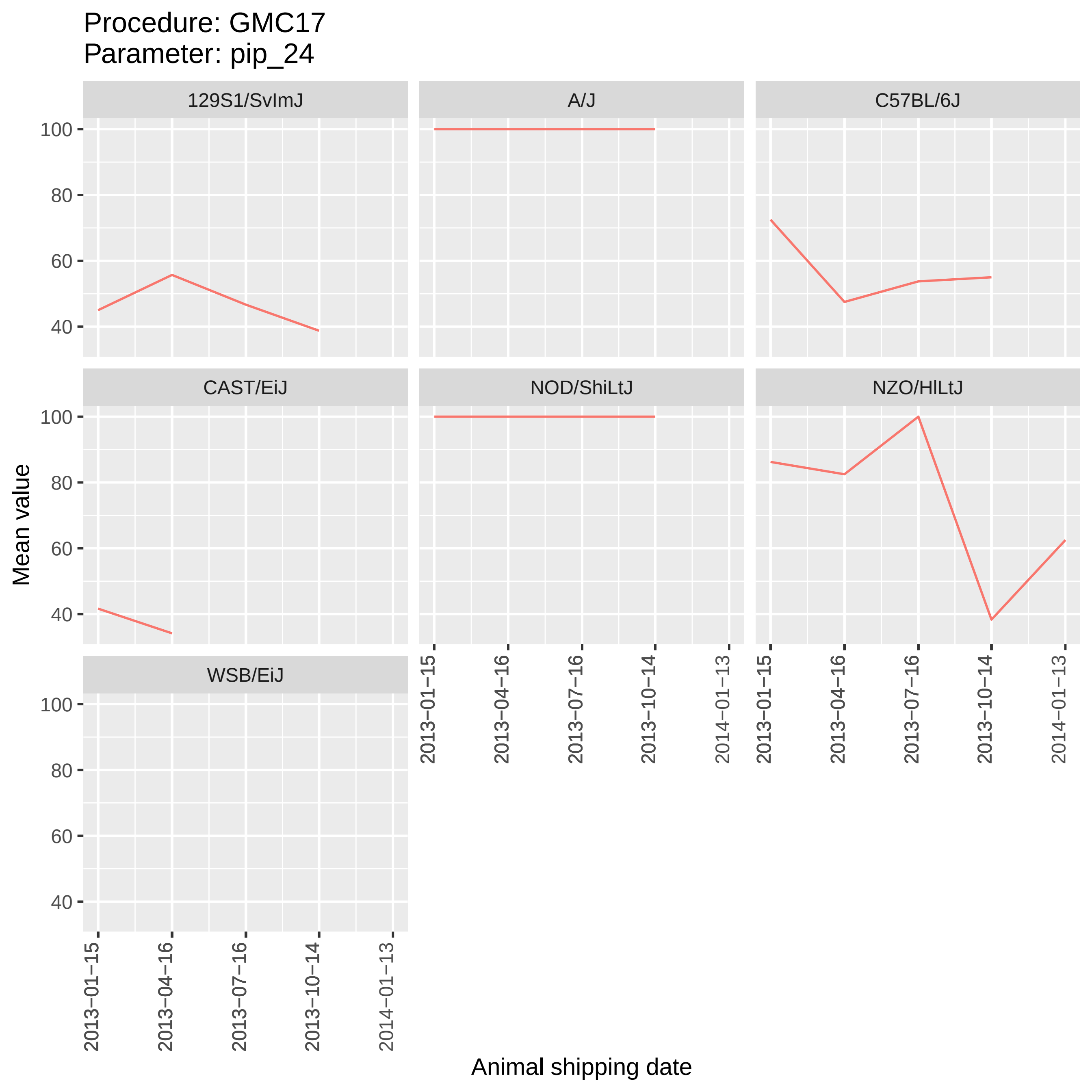

Procedure: GMC17
Parameter: pip_24
129S1/SvImJ
A/J
C57BL/6J
CAST/EiJ
NOD/ShiLtJ
NZO/HlLtJ
WSB/EiJ
Mean value
100
80
60
40
2013−01−15
2013−04−16
2013−07−16
2013−10−14
2014−01−13
Animal shipping date

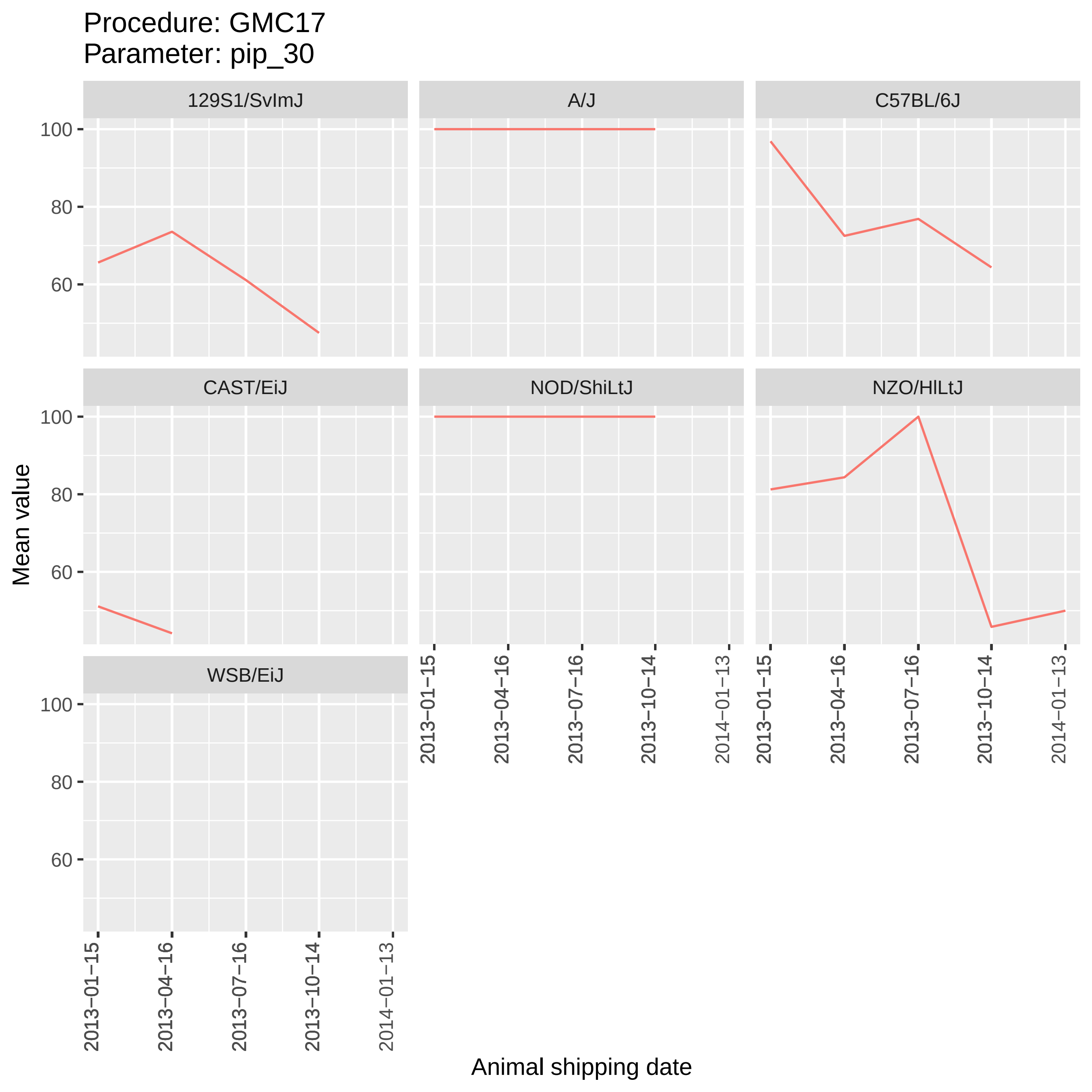

Procedure: GMC17
Parameter: pip_30
129S1/SvImJ
A/J
C57BL/6J
CAST/EiJ
NOD/ShiLtJ
NZO/HlLtJ
WSB/EiJ
100
80
60
Mean value
2013−01−15
2013−04−16
2013−07−16
2013−10−14
2014−01−13
Animal shipping date

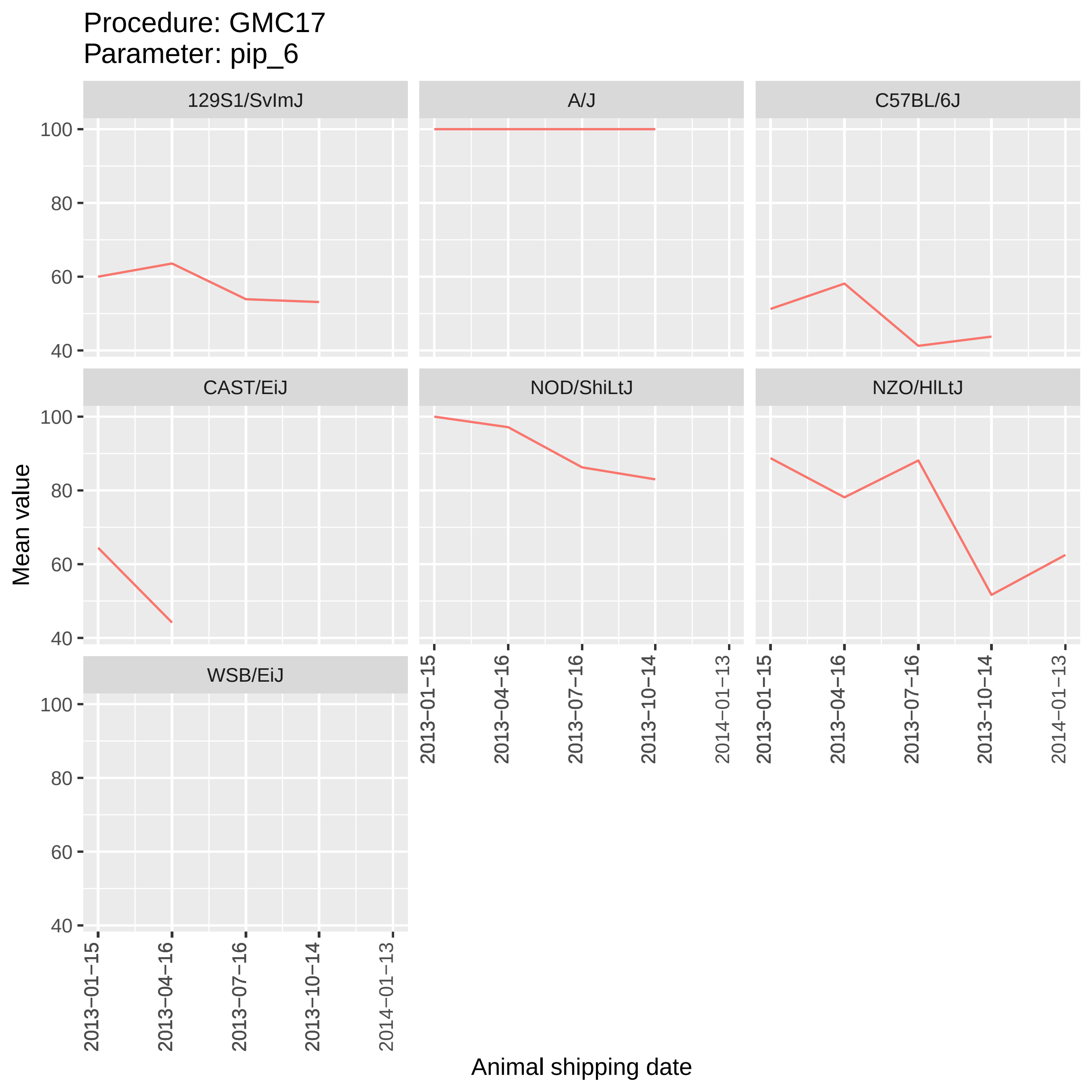

Procedure: GMC17
Parameter: pip_6
129S1/SvImJ
A/J
C57BL/6J
CAST/EiJ
NOD/ShiLtJ
NZO/HlLtJ
WSB/EiJ
Mean value
Animal shipping date
40
60
80
100
2013−01−15
2013−04−16
2013−07−16
2013−10−14
2014−01−13

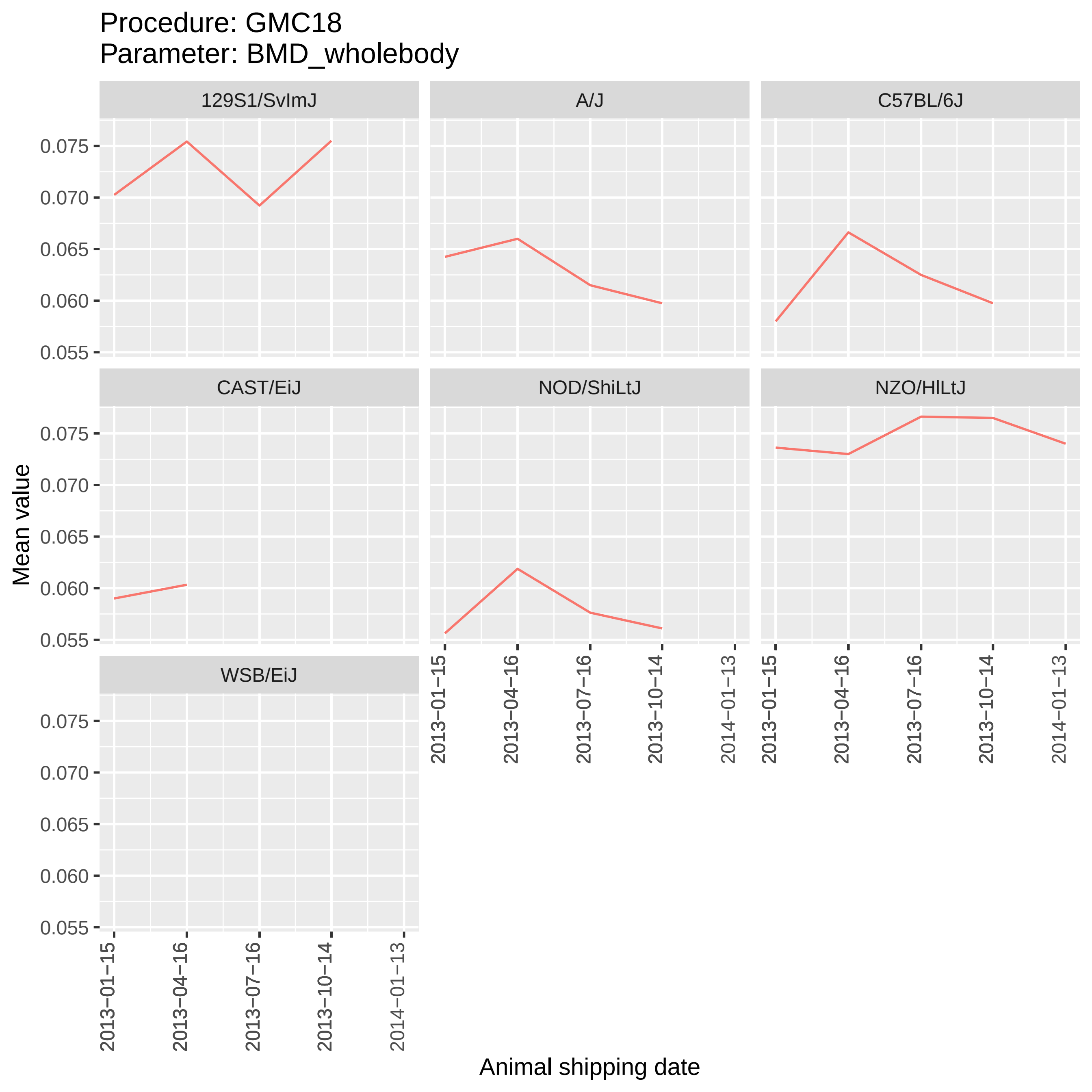
Procedure: GMC18
Parameter: BMD_wholebody
129S1/SvImJ
A/J
C57BL/6J
CAST/EiJ
NOD/ShiLtJ
NZO/HlLtJ
WSB/EiJ
Mean value
0.055
0.060
0.065
0.070
0.075
2013−01−15
2013−04−16
2013−07−16
2013−10−14
2014−01−13
Animal shipping date

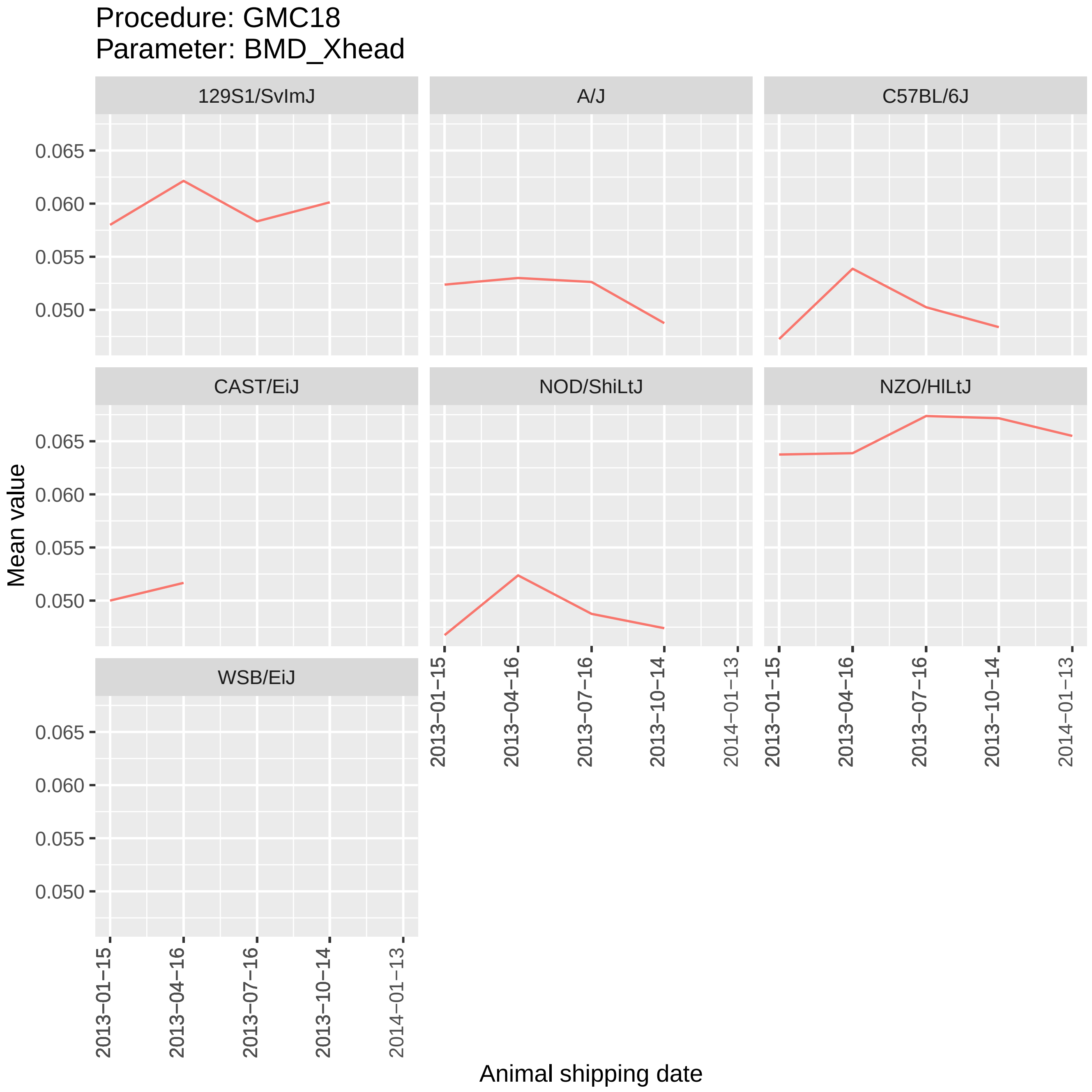
Procedure: GMC18
Parameter: BMD_Xhead
129S1/SvImJ
A/J
C57BL/6J
CAST/EiJ
NOD/ShiLtJ
NZO/HlLtJ
WSB/EiJ
Mean value
0.065
0.060
0.055
0.050
2013−01−15
2013−04−16
2013−07−16
2013−10−14
2014−01−13
Animal shipping date

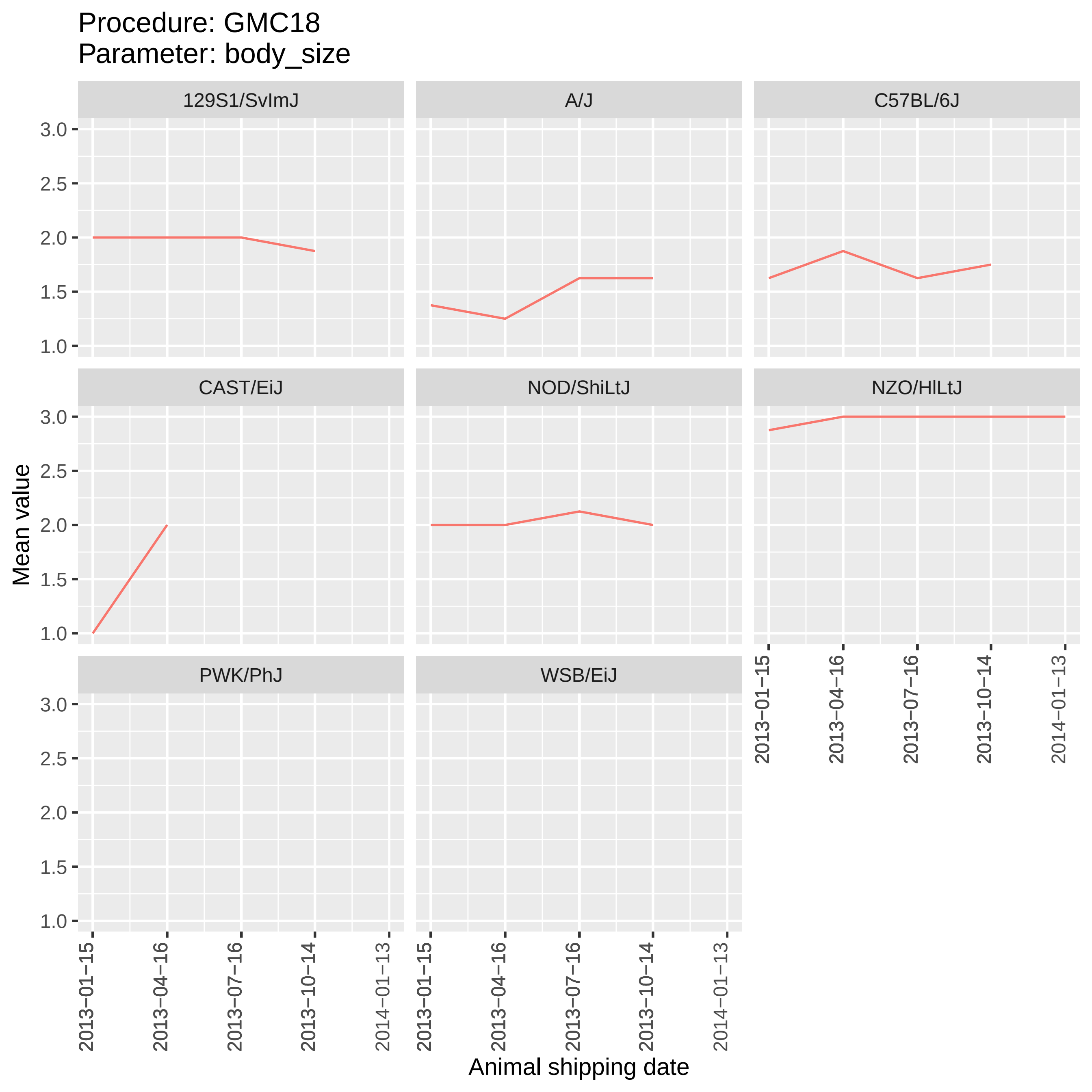
Procedure: GMC18
Parameter: body_size
129S1/SvImJ
A/J
C57BL/6J
CAST/EiJ
NOD/ShiLtJ
NZO/HlLtJ
PWK/PhJ
WSB/EiJ
Mean value
3.0
2.5
2.0
1.5
1.0
2013−01−15
2013−04−16
2013−07−16
2013−10−14
2014−01−13
Animal shipping date

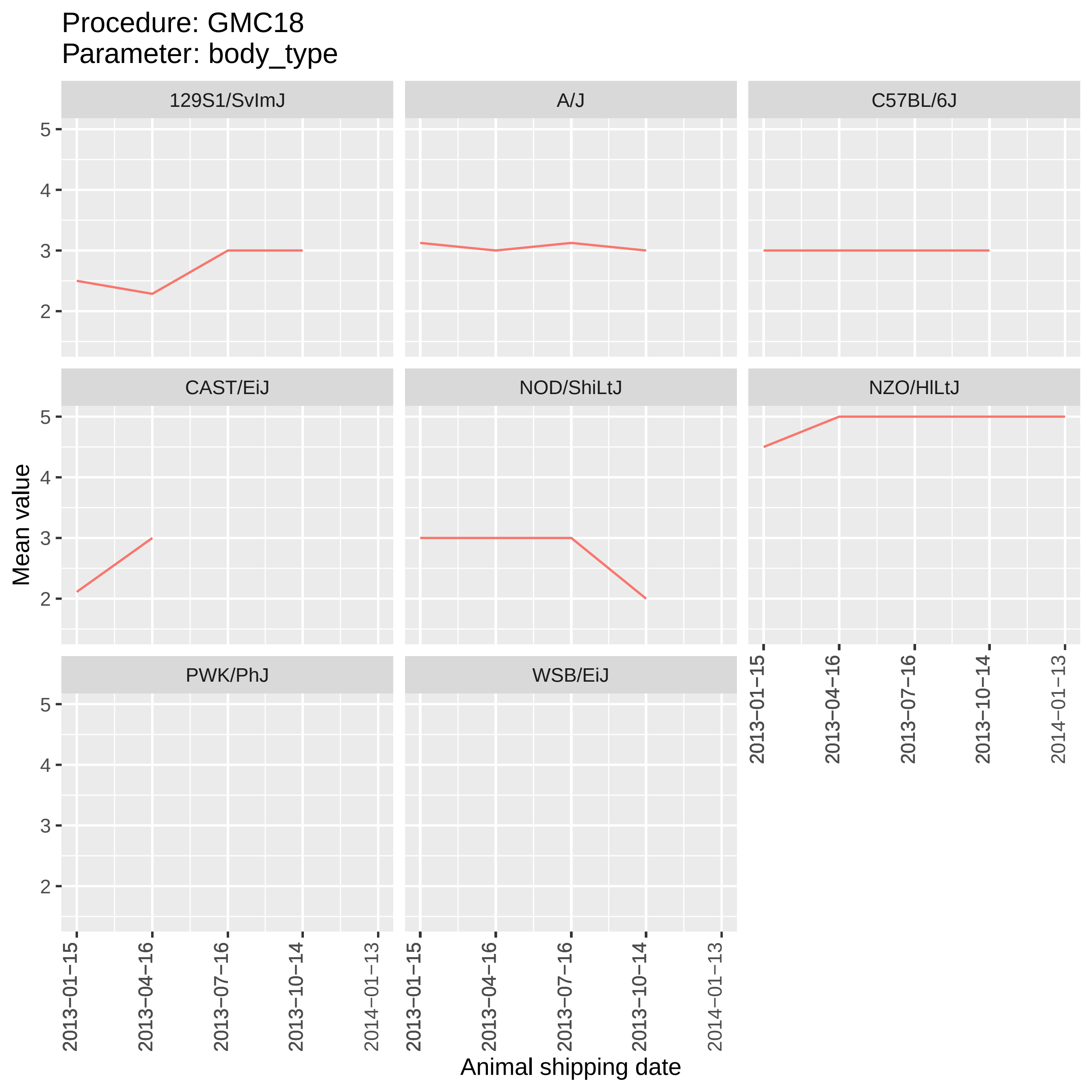

Procedure: GMC18
Parameter: body_type
129S1/SvImJ
A/J
C57BL/6J
CAST/EiJ
NOD/ShiLtJ
NZO/HlLtJ
PWK/PhJ
WSB/EiJ
Mean value
Animal shipping date
2
3
4
5
2013−01−15
2013−04−16
2013−07−16
2013−10−14
2014−01−13

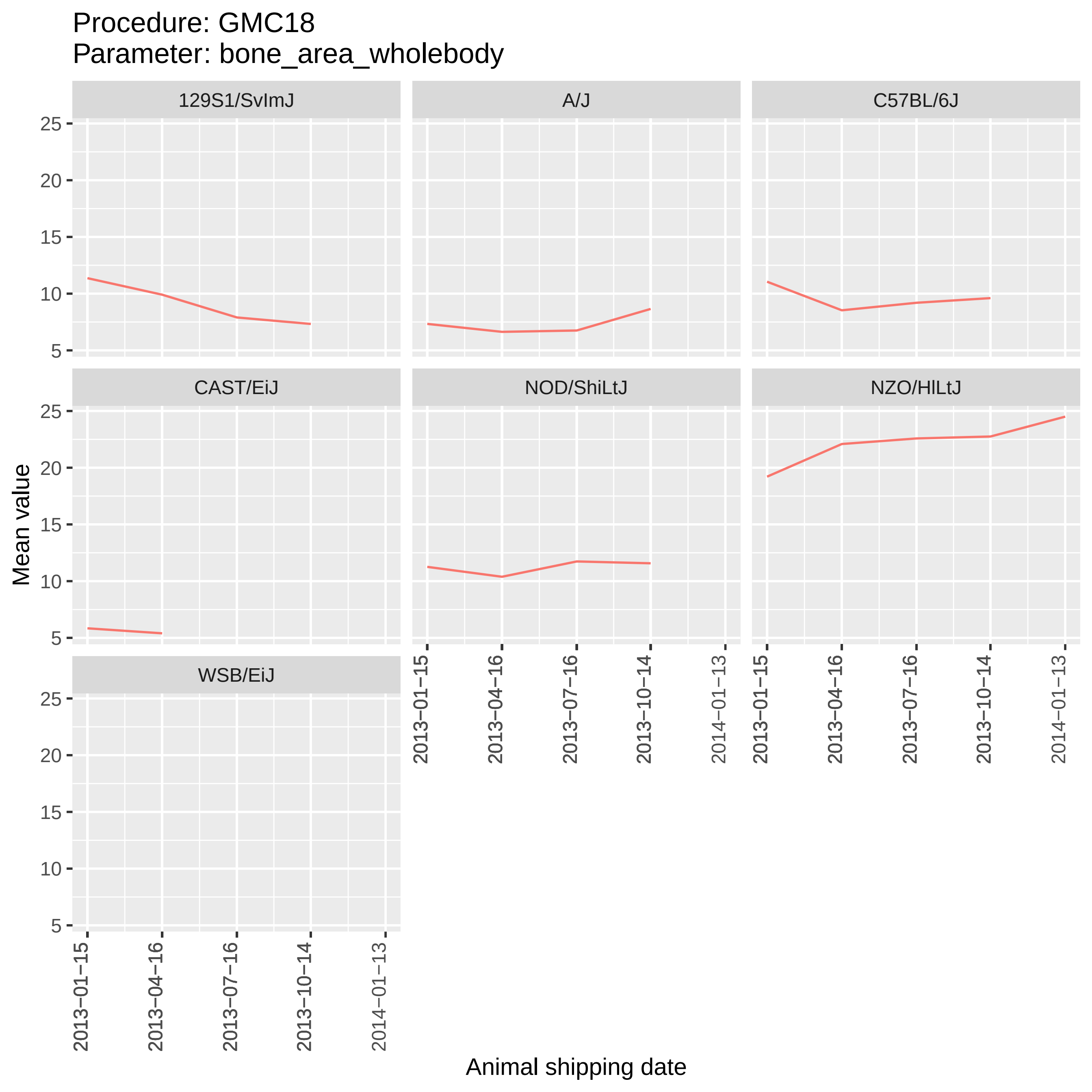
Procedure: GMC18
Parameter: bone_area_wholebody
129S1/SvImJ
A/J
C57BL/6J
CAST/EiJ
NOD/ShiLtJ
NZO/HlLtJ
WSB/EiJ
Mean value
Animal shipping date
2013−01−15
2013−04−16
2013−07−16
2013−10−14
2014−01−13
5
10
15
20
25

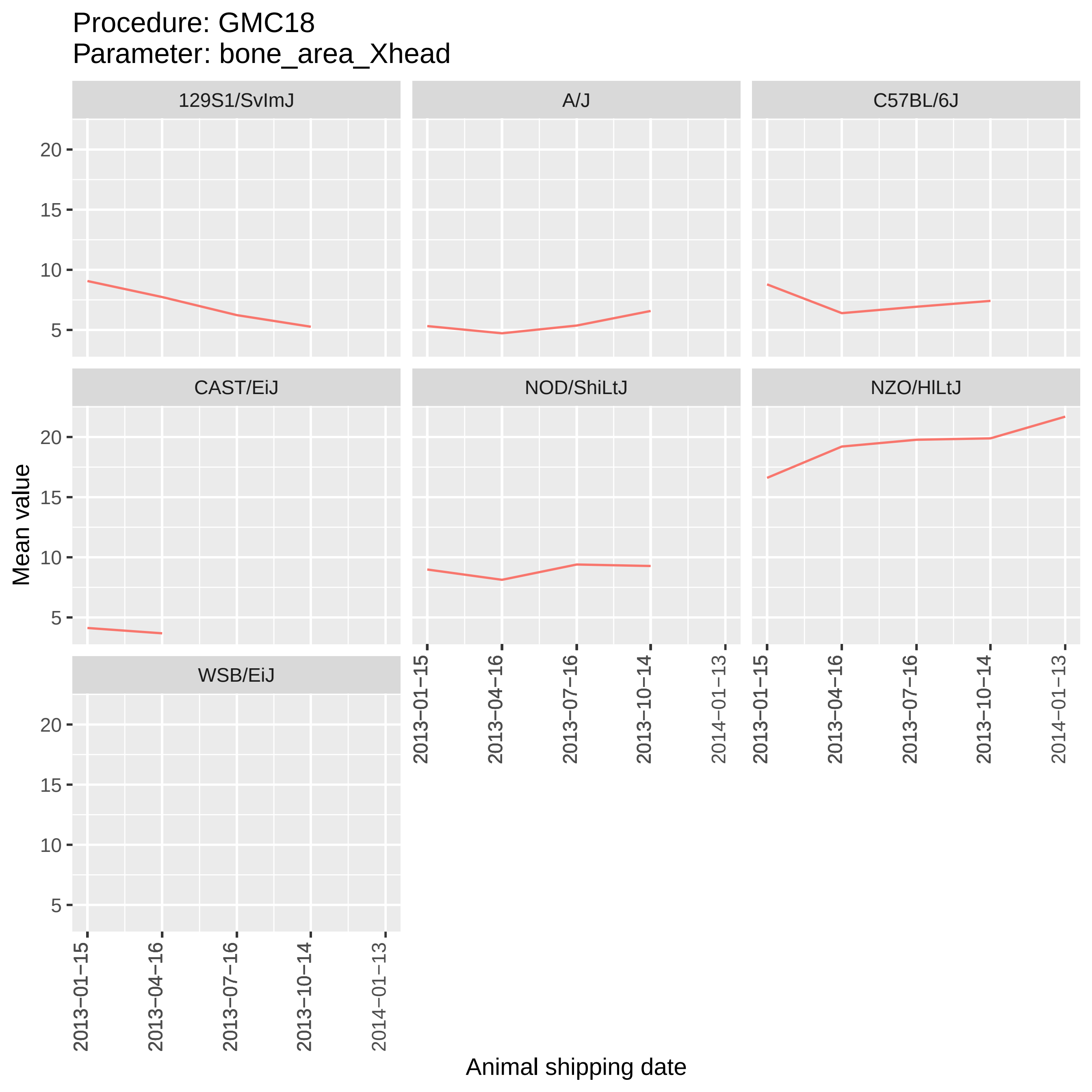

Procedure: GMC18
Parameter: bone_area_Xhead
129S1/SvImJ
A/J
C57BL/6J
CAST/EiJ
NOD/ShiLtJ
NZO/HlLtJ
WSB/EiJ
Mean value
20
15
10
5
2013−01−15
2013−04−16
2013−07−16
2013−10−14
2014−01−13
Animal shipping date

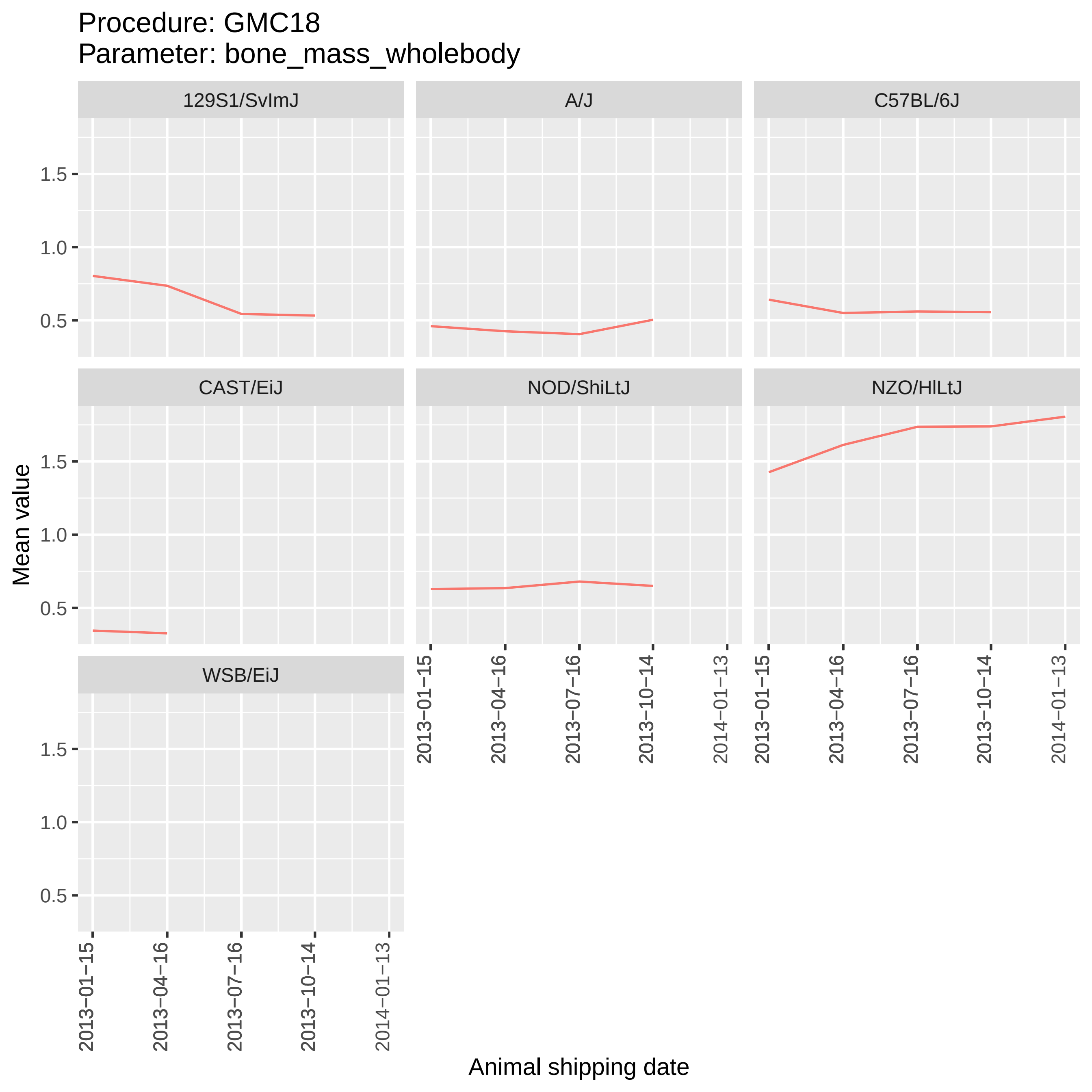

Procedure: GMC18
Parameter: bone_mass_wholebody
129S1/SvImJ
A/J
C57BL/6J
CAST/EiJ
NOD/ShiLtJ
NZO/HlLtJ
WSB/EiJ
Mean value
0.5
1.0
1.5
2013−01−15
2013−04−16
2013−07−16
2013−10−14
2014−01−13
Animal shipping date

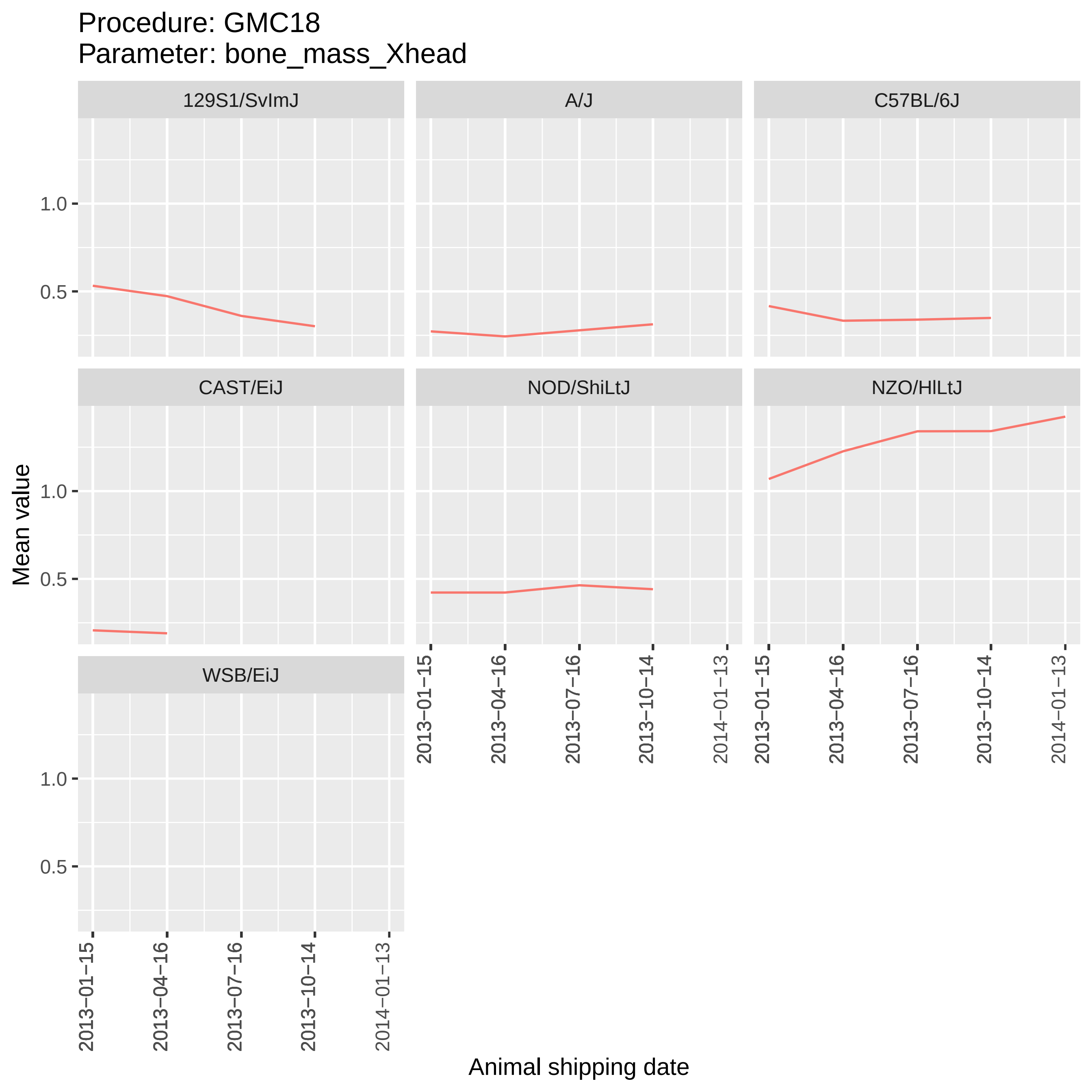

Procedure: GMC18
Parameter: bone_mass_Xhead
129S1/SvImJ
A/J
C57BL/6J
CAST/EiJ
NOD/ShiLtJ
NZO/HlLtJ
WSB/EiJ
Mean value
1.0
0.5
2013−01−15
2013−04−16
2013−07−16
2013−10−14
2014−01−13
Animal shipping date

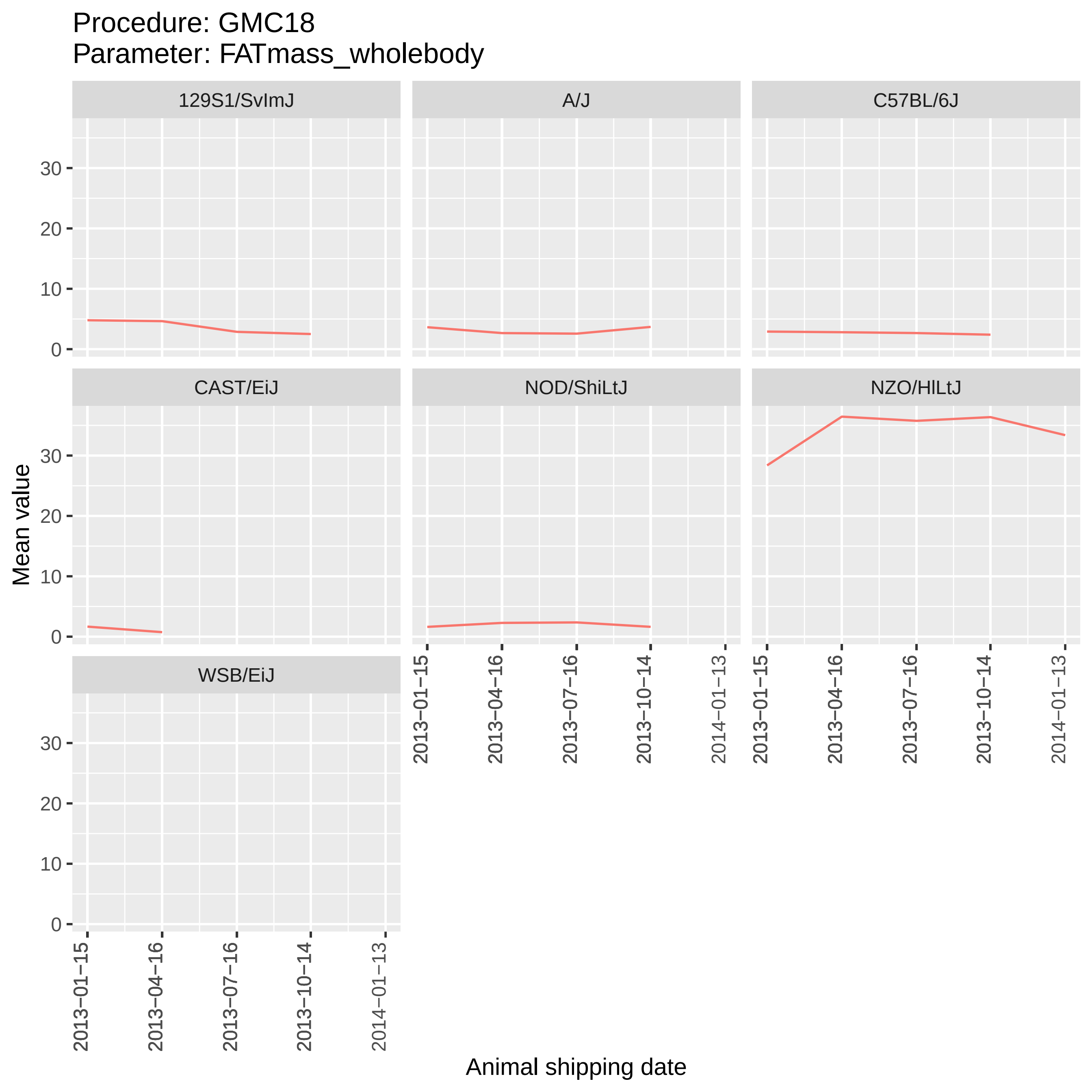

Procedure: GMC18
Parameter: FATmass_wholebody
129S1/SvImJ
A/J
C57BL/6J
CAST/EiJ
NOD/ShiLtJ
NZO/HlLtJ
WSB/EiJ
Mean value
0
10
20
30
2013−01−15
2013−04−16
2013−07−16
2013−10−14
2014−01−13
Animal shipping date

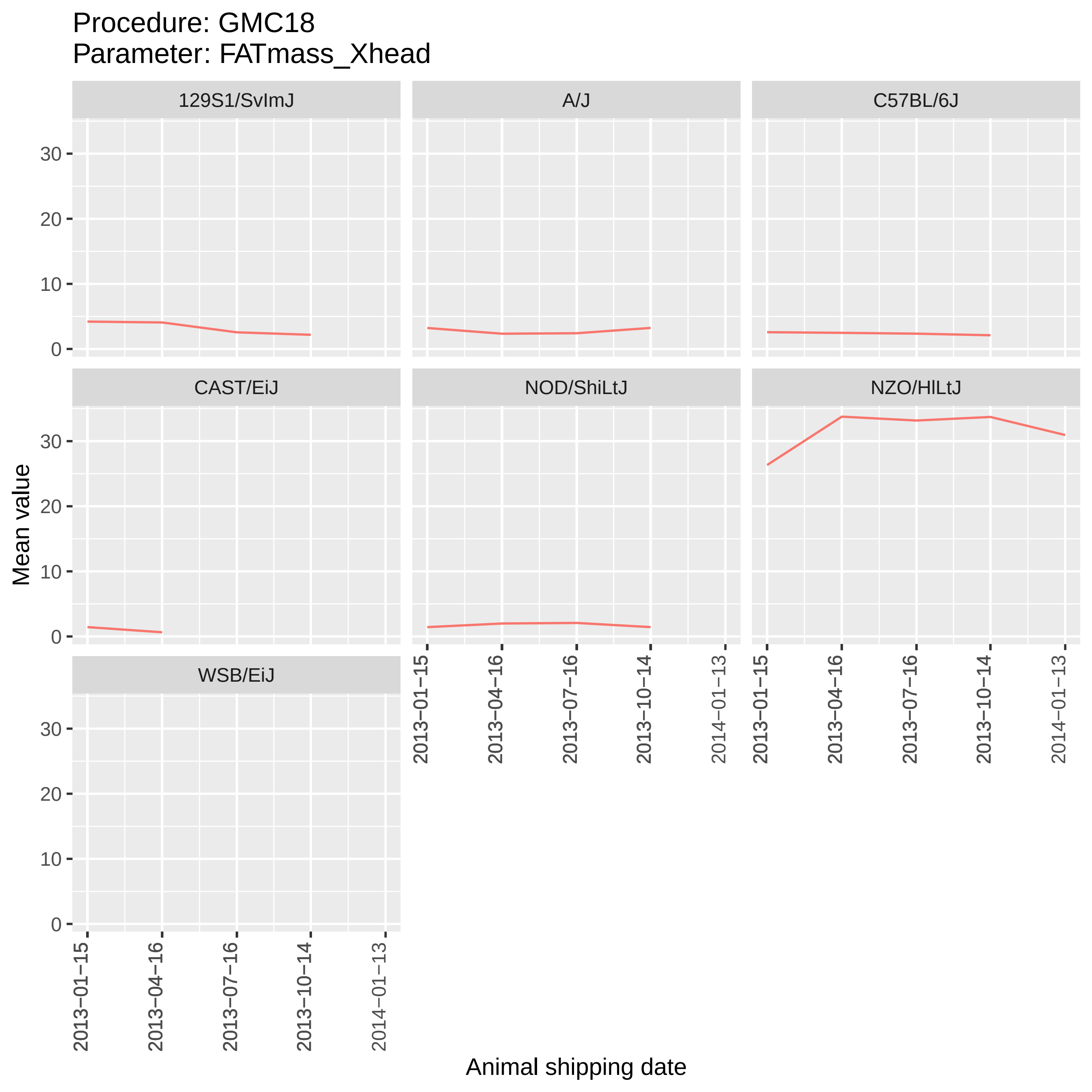
Procedure: GMC18
Parameter: FATmass_Xhead
129S1/SvImJ
A/J
C57BL/6J
CAST/EiJ
NOD/ShiLtJ
NZO/HlLtJ
WSB/EiJ
Mean value
0
10
20
30
2013−01−15
2013−04−16
2013−07−16
2013−10−14
2014−01−13
Animal shipping date

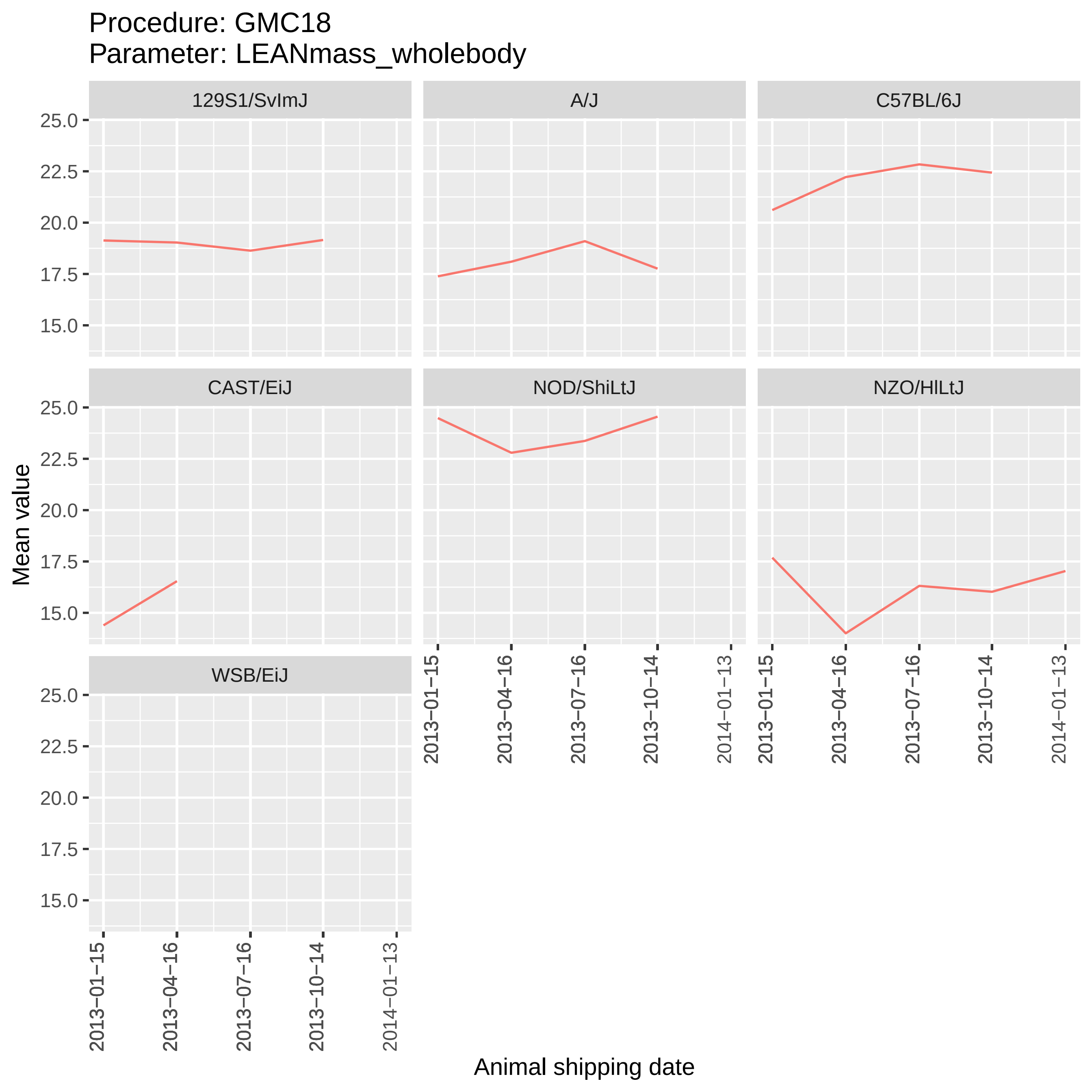
Procedure: GMC18
Parameter: LEANmass_wholebody
129S1/SvImJ
A/J
C57BL/6J
CAST/EiJ
NOD/ShiLtJ
NZO/HlLtJ
WSB/EiJ
Mean value
25.0
22.5
20.0
17.5
15.0
2013−01−15
2013−04−16
2013−07−16
2013−10−14
2014−01−13
Animal shipping date

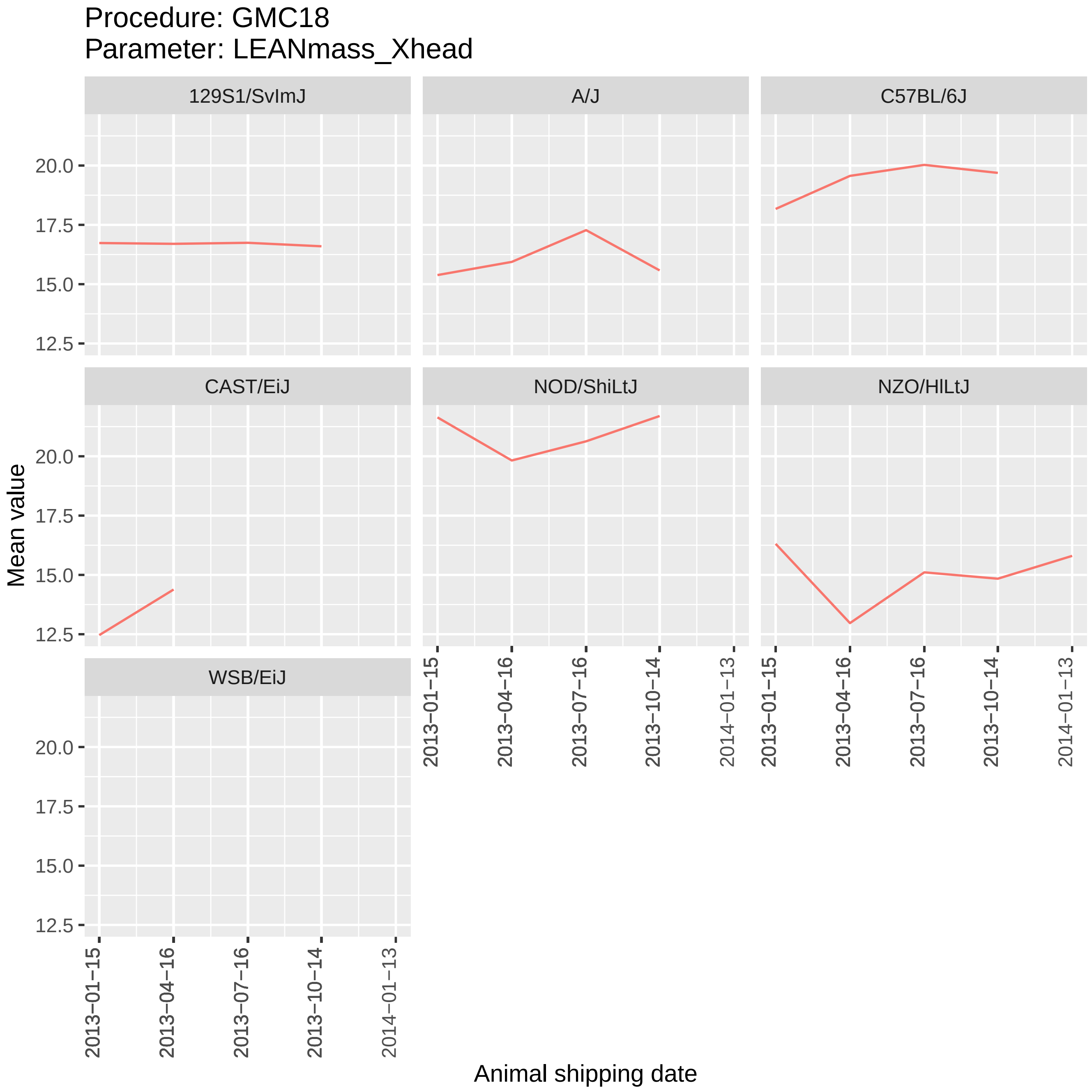

Procedure: GMC18
Parameter: LEANmass_Xhead
129S1/SvImJ
A/J
C57BL/6J
CAST/EiJ
NOD/ShiLtJ
NZO/HlLtJ
WSB/EiJ
Mean value
20.0
17.5
15.0
12.5
2013−01−15
2013−04−16
2013−07−16
2013−10−14
2014−01−13
Animal shipping date

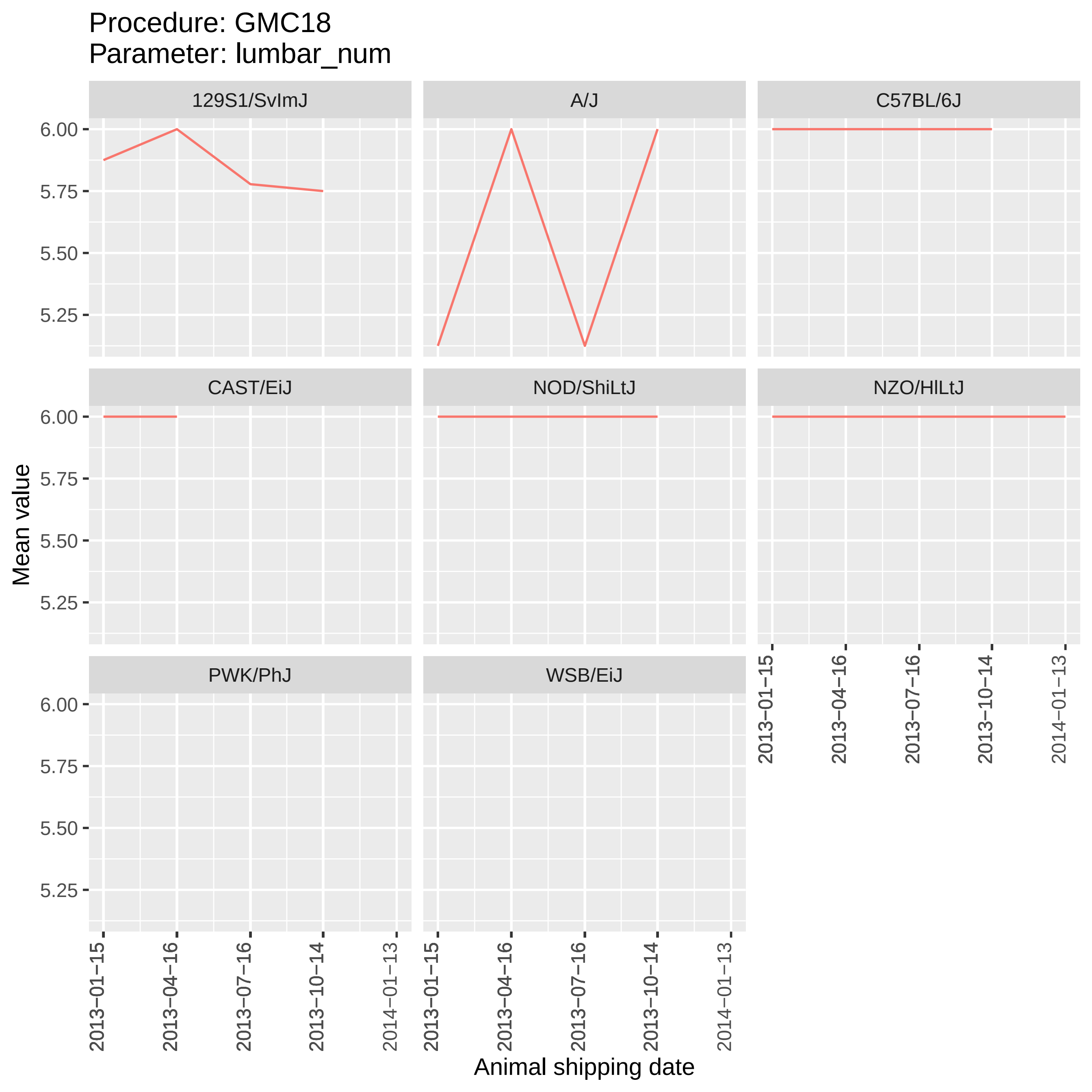

Procedure: GMC18
Parameter: lumbar_num
129S1/SvImJ
A/J
C57BL/6J
CAST/EiJ
NOD/ShiLtJ
NZO/HlLtJ
PWK/PhJ
WSB/EiJ
6.00
5.75
5.50
5.25
Mean value
2013−01−15
2013−04−16
2013−07−16
2013−10−14
2014−01−13
Animal shipping date

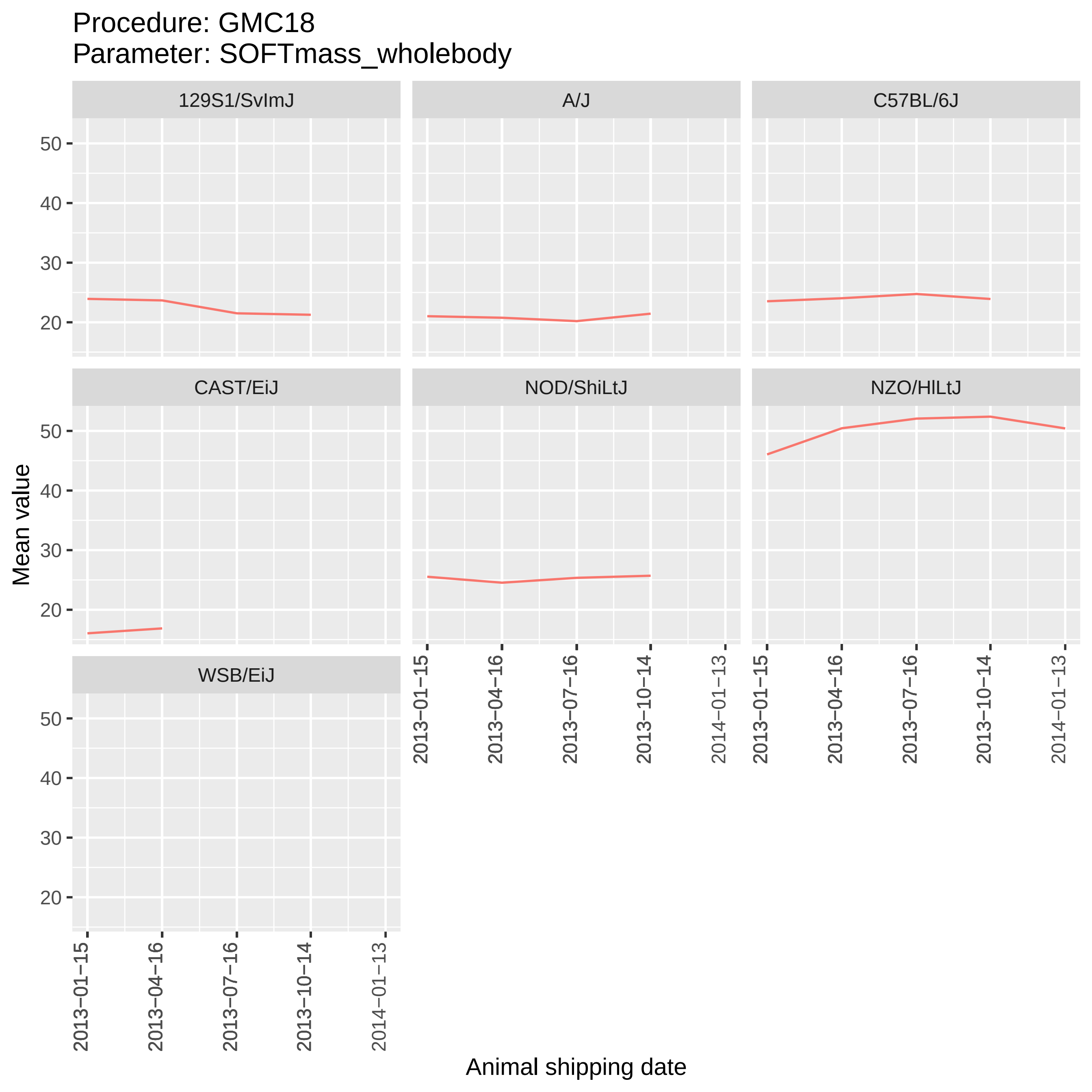

Procedure: GMC18
Parameter: SOFTmass_wholebody
129S1/SvImJ
A/J
C57BL/6J
CAST/EiJ
NOD/ShiLtJ
NZO/HlLtJ
WSB/EiJ
Mean value
50
40
30
20
2013−01−15
2013−04−16
2013−07−16
2013−10−14
2014−01−13
Animal shipping date

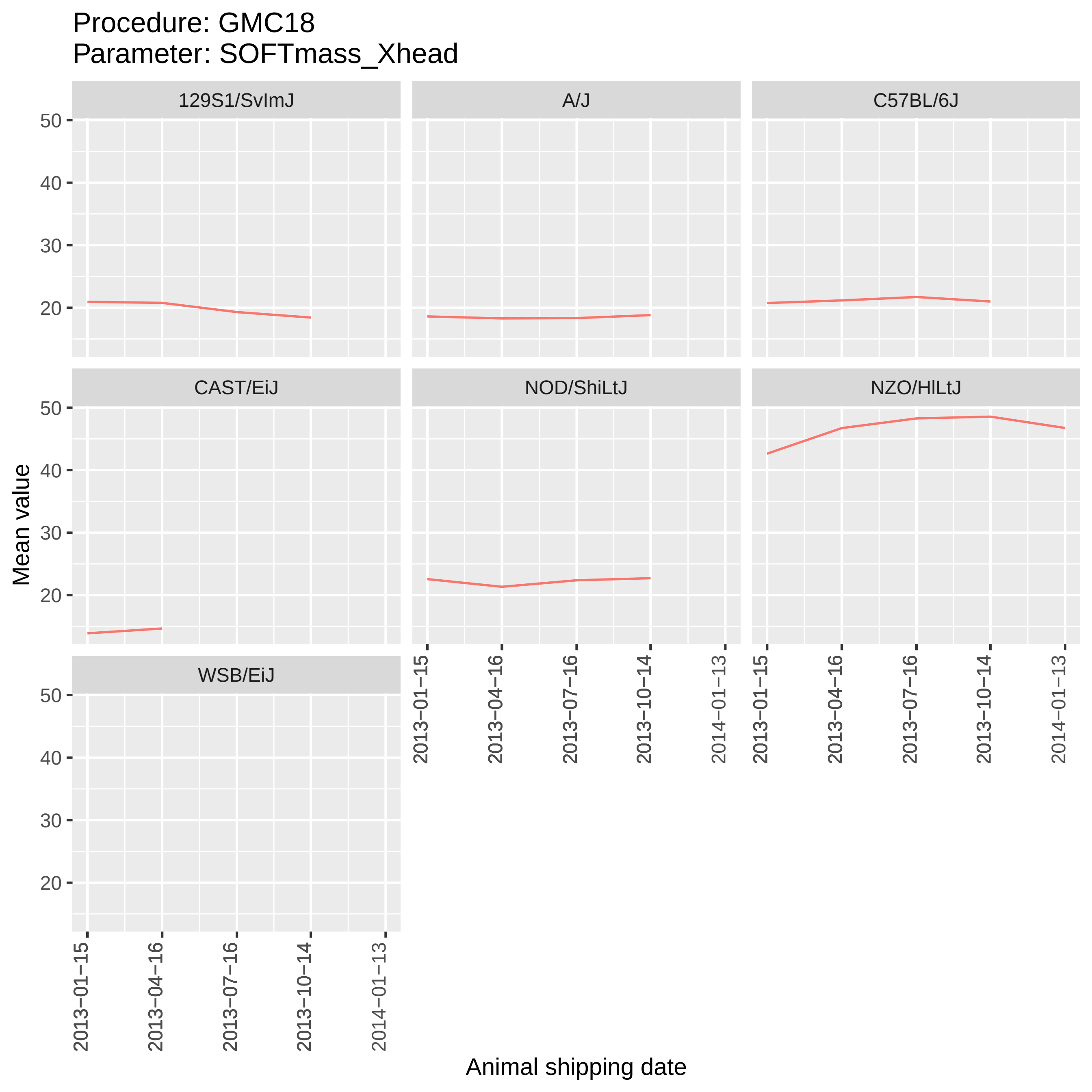

Procedure: GMC18
Parameter: SOFTmass_Xhead
129S1/SvImJ
A/J
C57BL/6J
CAST/EiJ
NOD/ShiLtJ
NZO/HlLtJ
WSB/EiJ
Mean value
50
40
30
20
2013−01−15
2013−04−16
2013−07−16
2013−10−14
2014−01−13
Animal shipping date

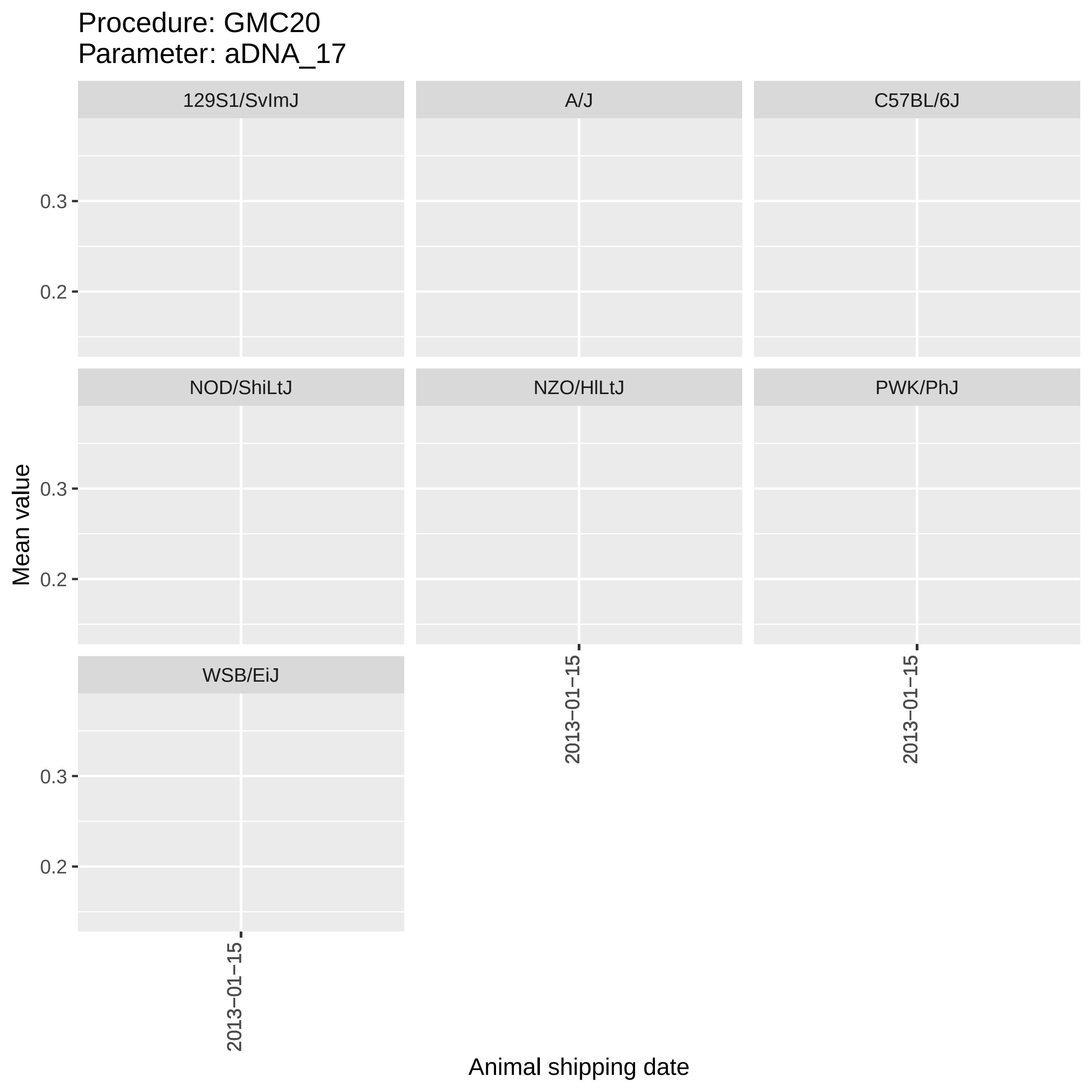
Procedure: GMC20
Parameter: aDNA_17
129S1/SvImJ
A/J
C57BL/6J
NOD/ShiLtJ
NZO/HlLtJ
PWK/PhJ
WSB/EiJ
Mean value
0.3
0.2
2013−01−15
Animal shipping date

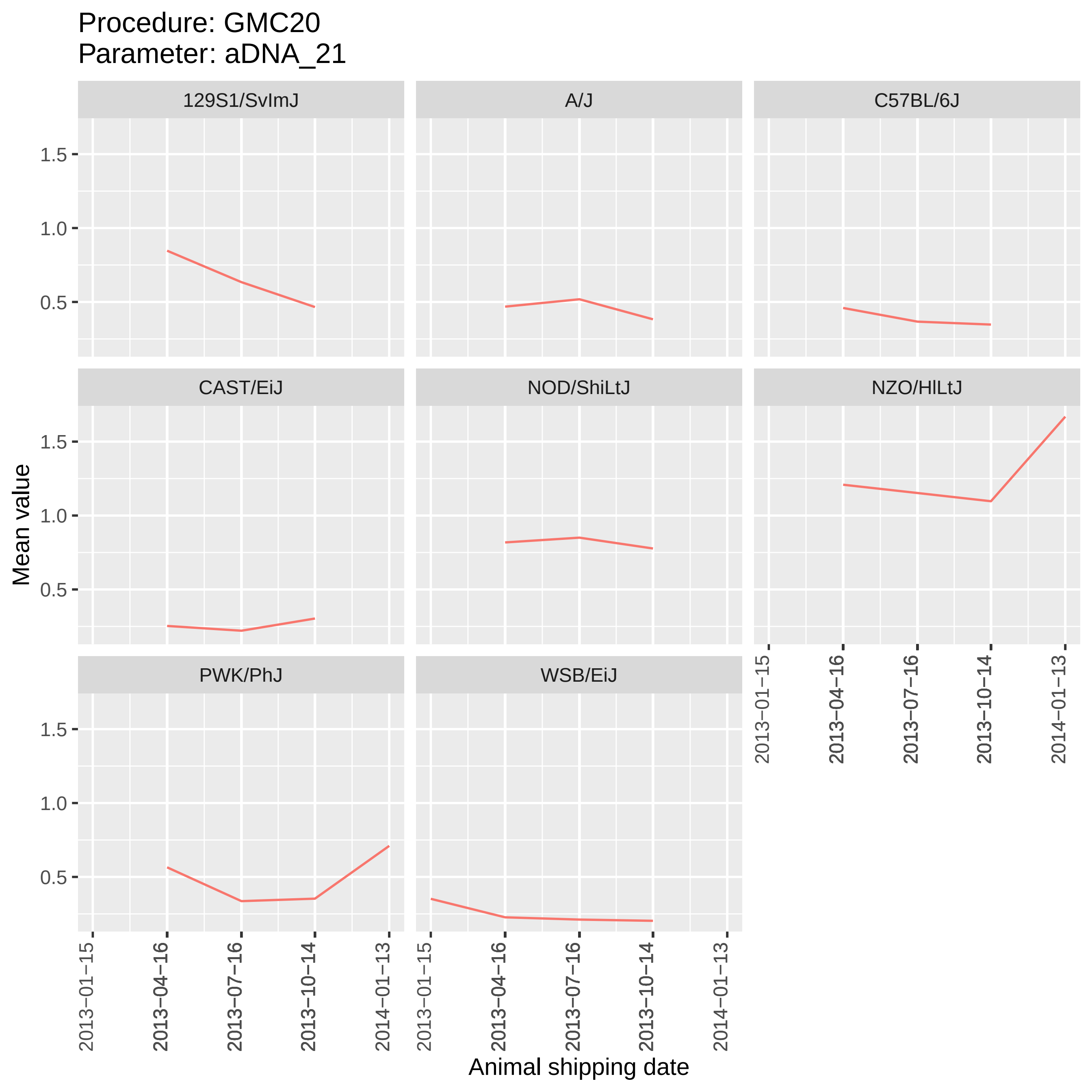

Procedure: GMC20
Parameter: aDNA_21
129S1/SvImJ
A/J
C57BL/6J
CAST/EiJ
NOD/ShiLtJ
NZO/HlLtJ
PWK/PhJ
WSB/EiJ
Mean value
0.5
1.0
1.5
2013−01−15
2013−04−16
2013−07−16
2013−10−14
2014−01−13
Animal shipping date

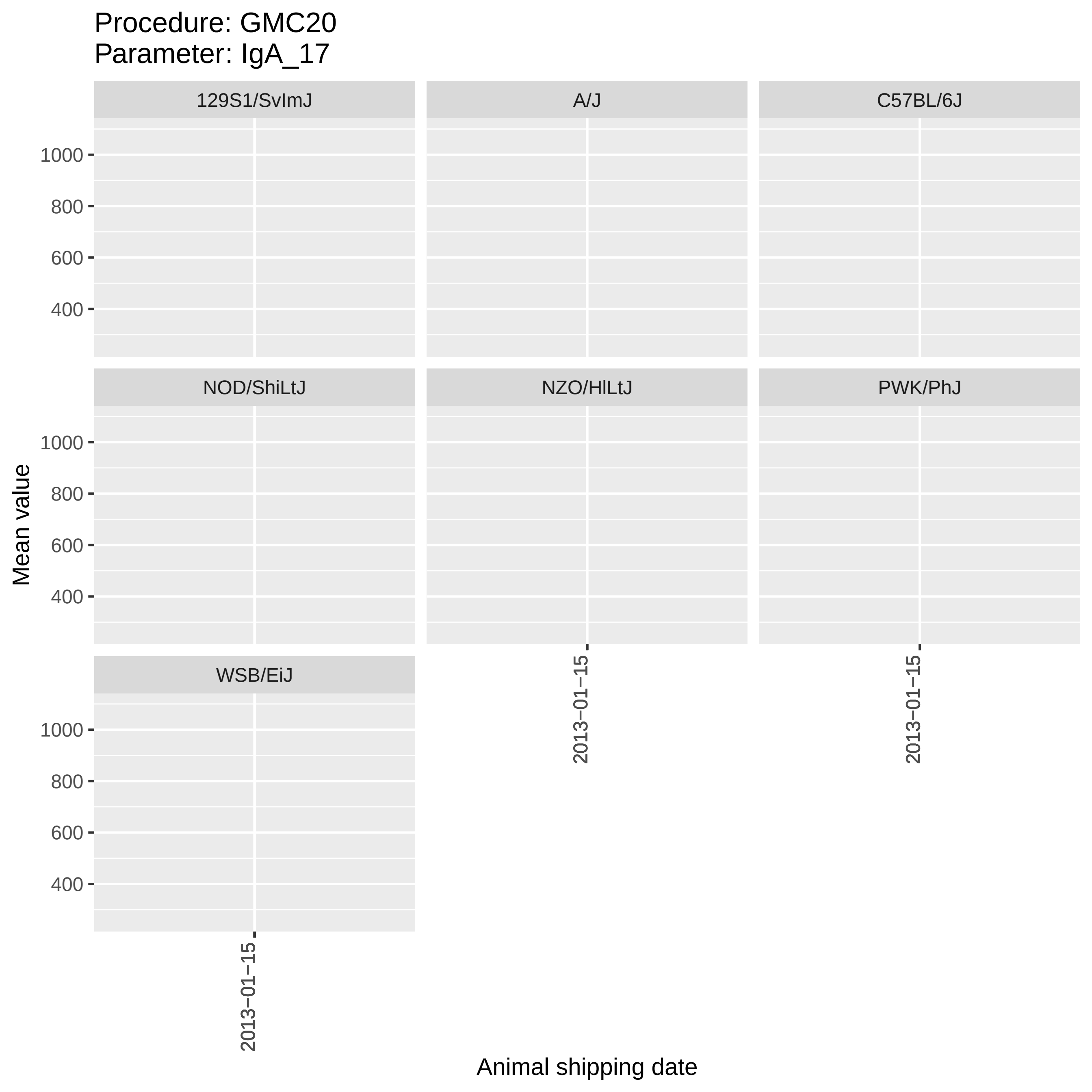
Procedure: GMC20
Parameter: IgA_17
129S1/SvImJ
A/J
C57BL/6J
NOD/ShiLtJ
NZO/HlLtJ
PWK/PhJ
WSB/EiJ
Mean value
1000
800
600
400
2013−01−15
Animal shipping date

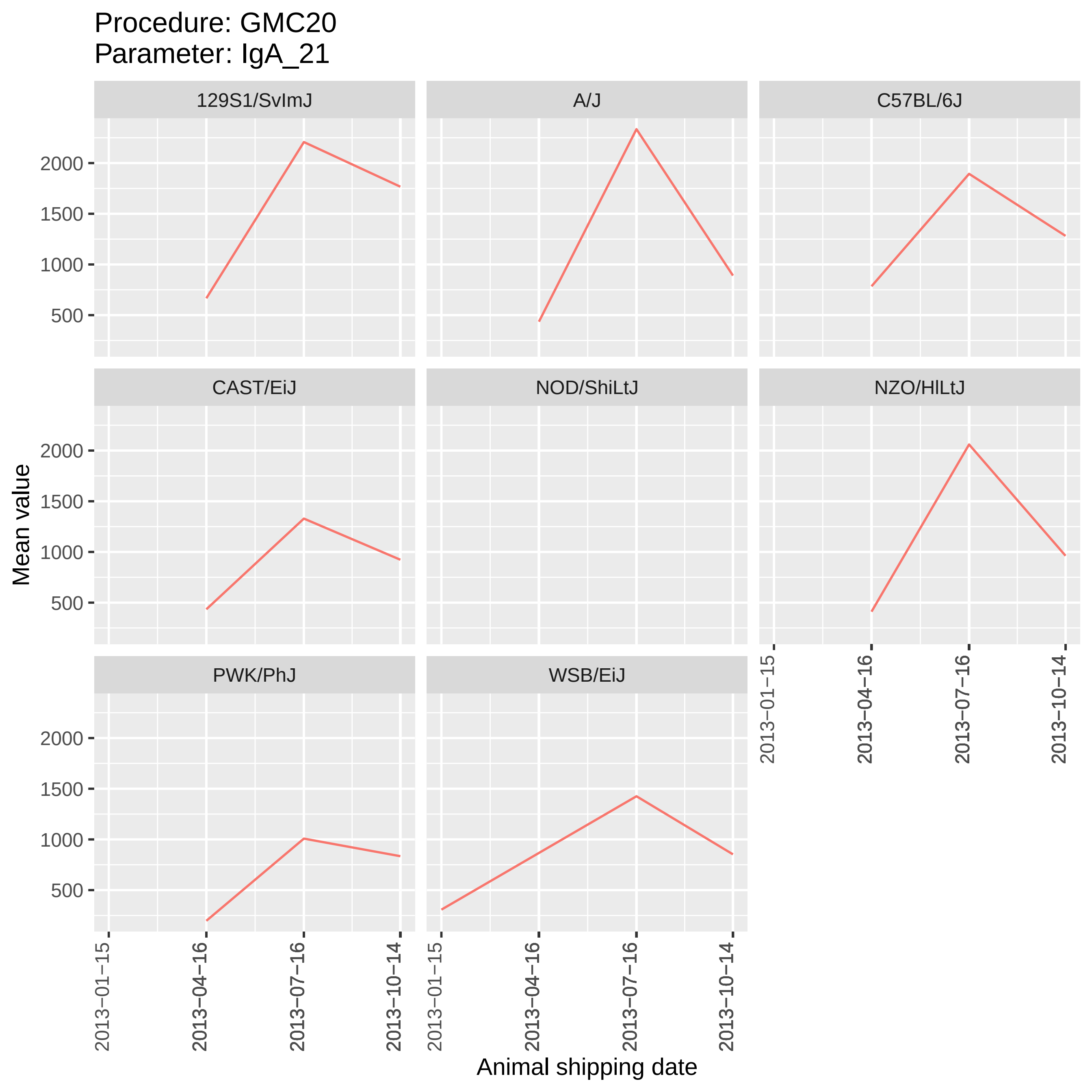
Procedure: GMC20
Parameter: IgA_21
129S1/SvImJ
A/J
C57BL/6J
CAST/EiJ
NOD/ShiLtJ
NZO/HlLtJ
PWK/PhJ
WSB/EiJ
Mean value
500
1000
1500
2000
2013−01−15
2013−04−16
2013−07−16
2013−10−14
Animal shipping date

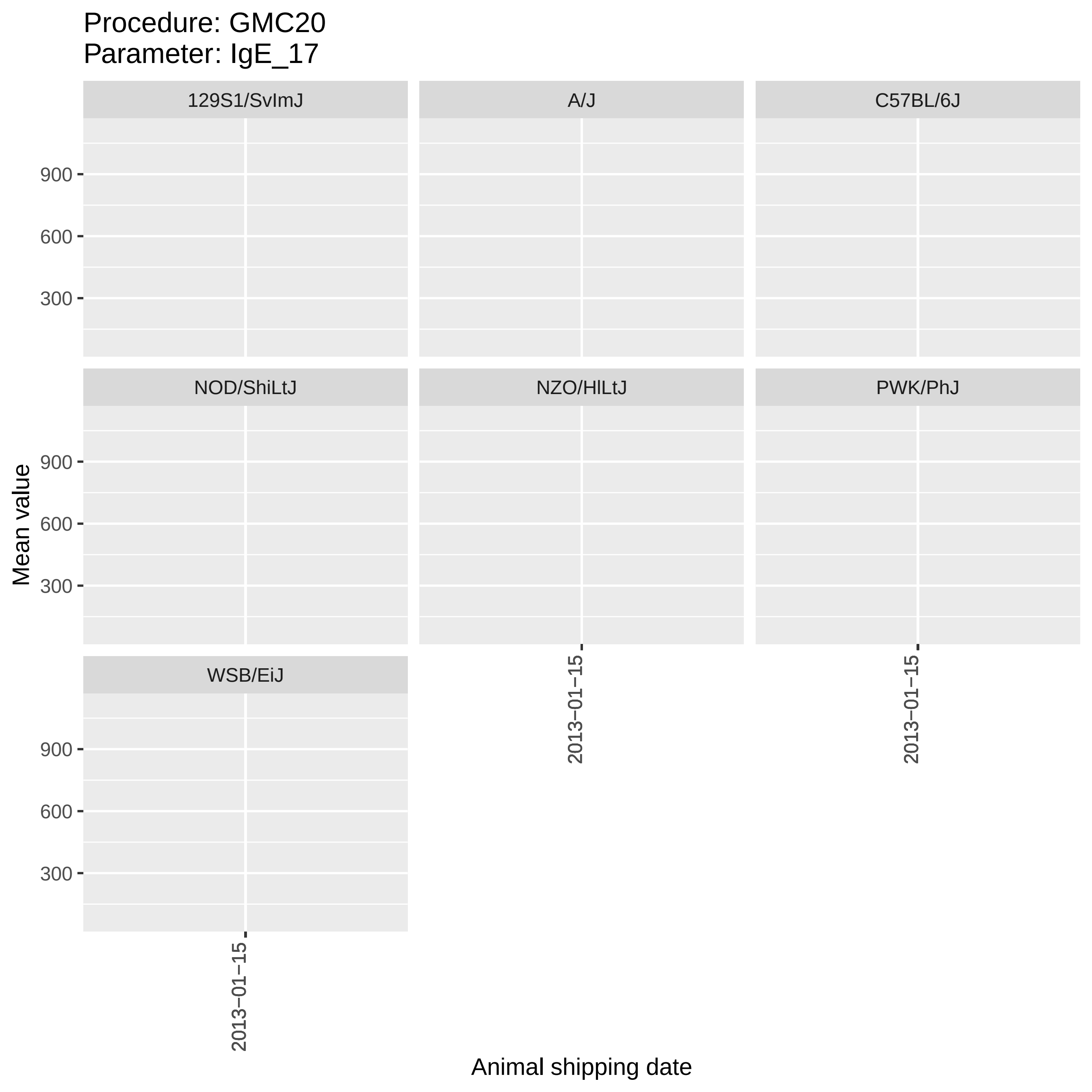

Procedure: GMC20
Parameter: IgE_17
129S1/SvImJ
A/J
C57BL/6J
NOD/ShiLtJ
NZO/HlLtJ
PWK/PhJ
WSB/EiJ
900
600
300
Mean value
2013−01−15
Animal shipping date

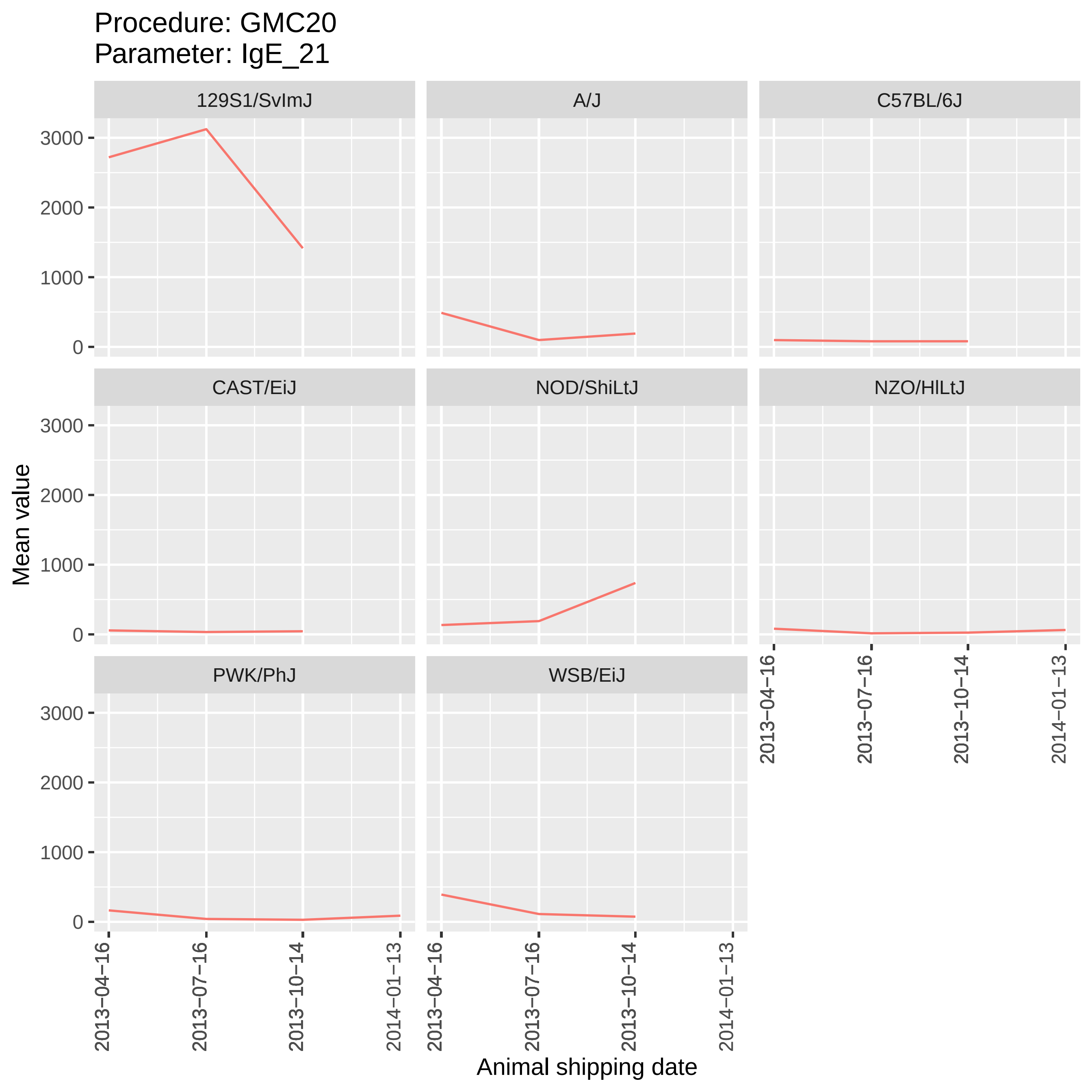

Procedure: GMC20
Parameter: IgE_21
129S1/SvImJ
A/J
C57BL/6J
CAST/EiJ
NOD/ShiLtJ
NZO/HlLtJ
PWK/PhJ
WSB/EiJ
3000
2000
1000
0
Mean value
2013−04−16
2013−07−16
2013−10−14
2014−01−13
Animal shipping date

Procedure: GMC20
Parameter: IgG1_21

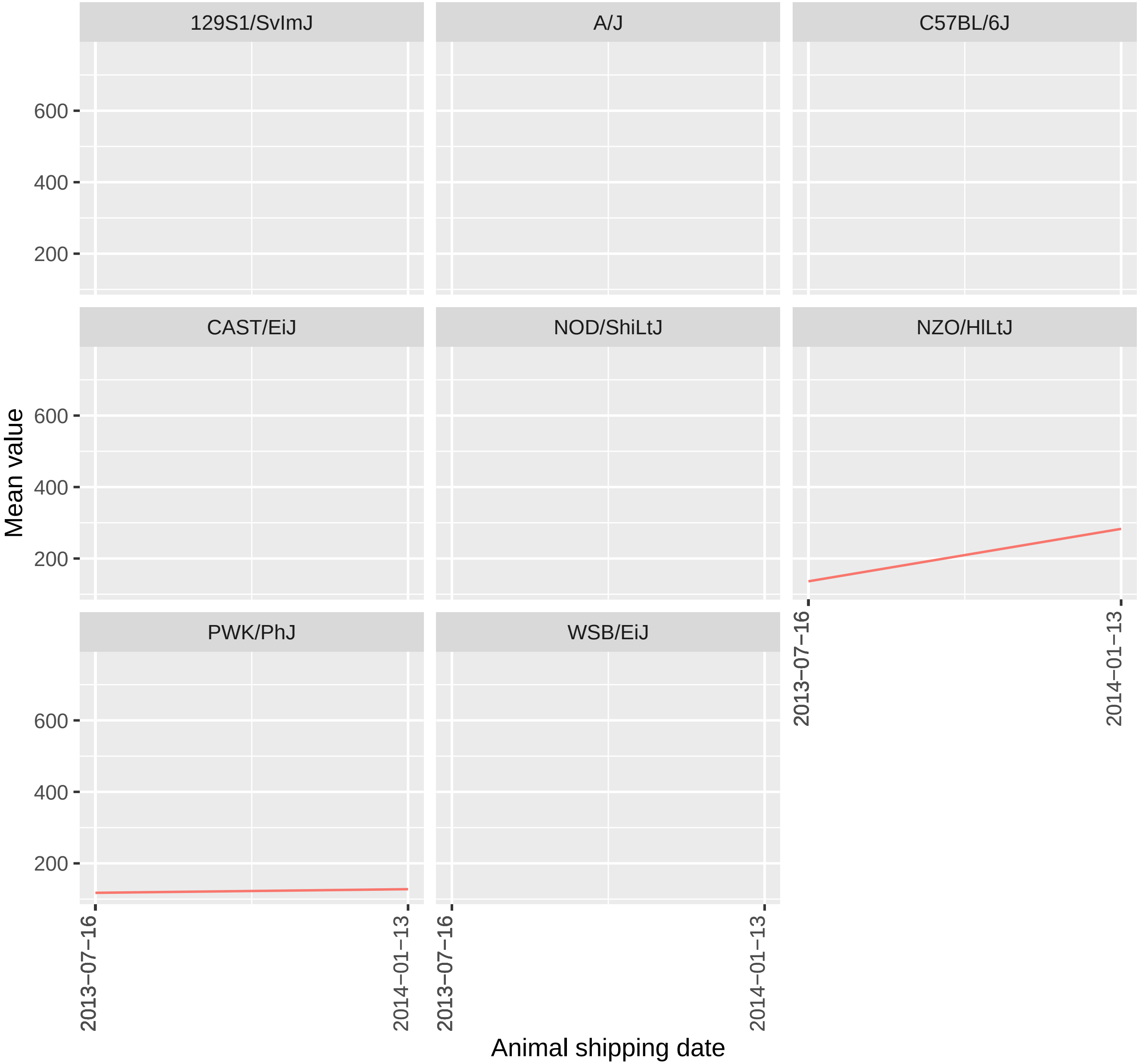

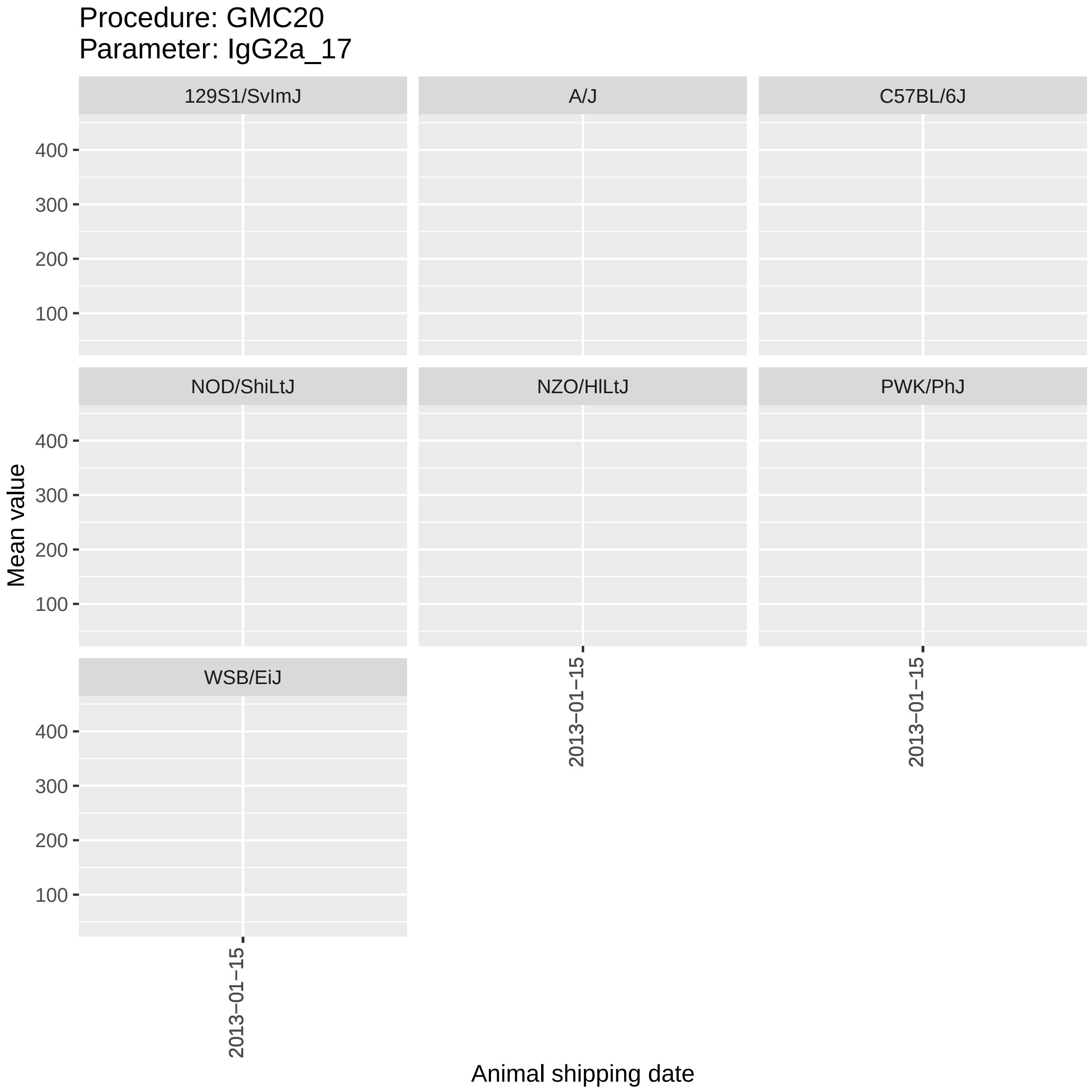

Procedure: GMC20
Parameter: IgG2a_17
129S1/SvImJ
A/J
C57BL/6J
NOD/ShiLtJ
NZO/HlLtJ
PWK/PhJ
WSB/EiJ
Mean value
100
200
300
400
2013−01−15
Animal shipping date

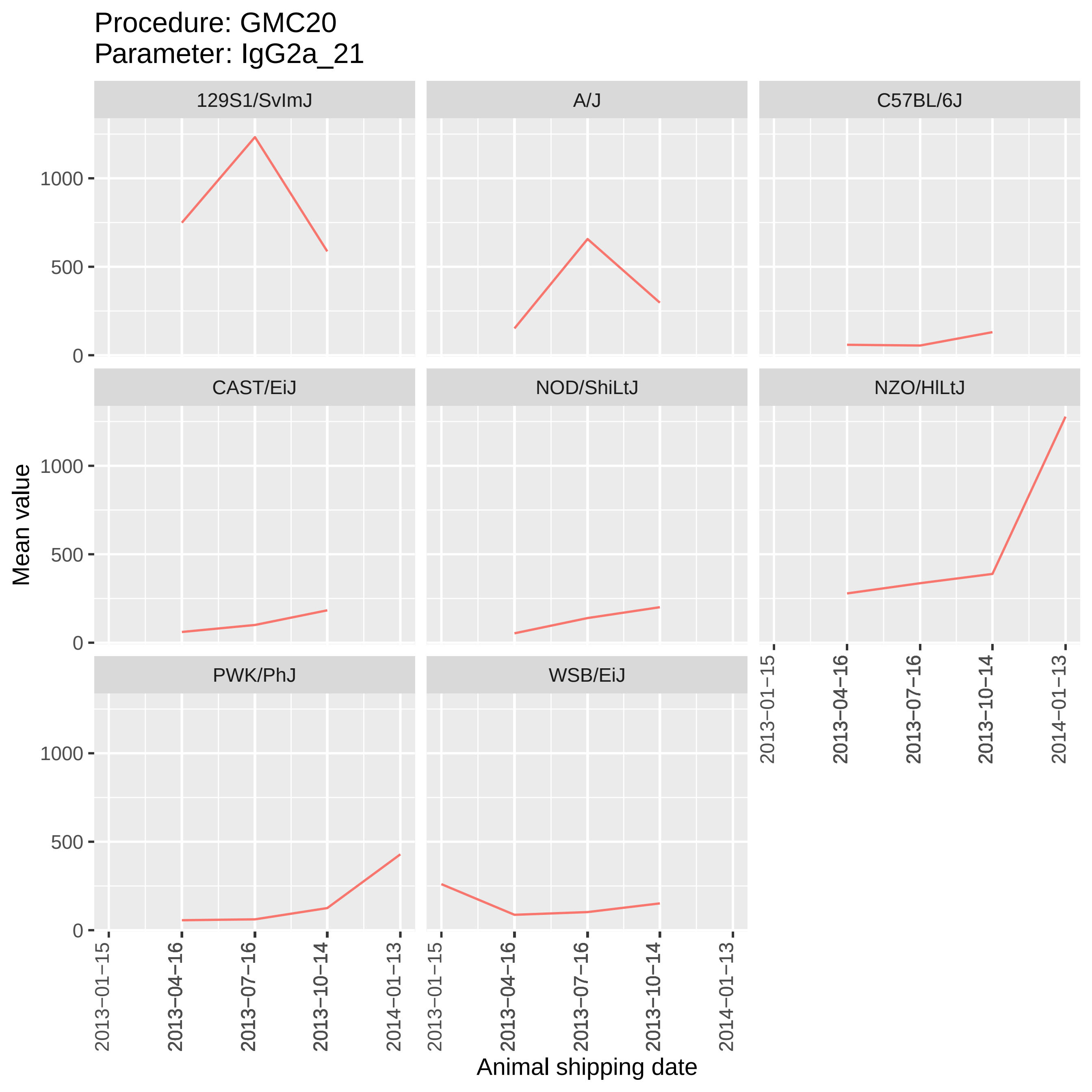

Procedure: GMC20
Parameter: IgG2a_21
129S1/SvImJ
A/J
C57BL/6J
CAST/EiJ
NOD/ShiLtJ
NZO/HlLtJ
PWK/PhJ
WSB/EiJ
Mean value
0
500
1000
2013−01−15
2013−04−16
2013−07−16
2013−10−14
2014−01−13
Animal shipping date

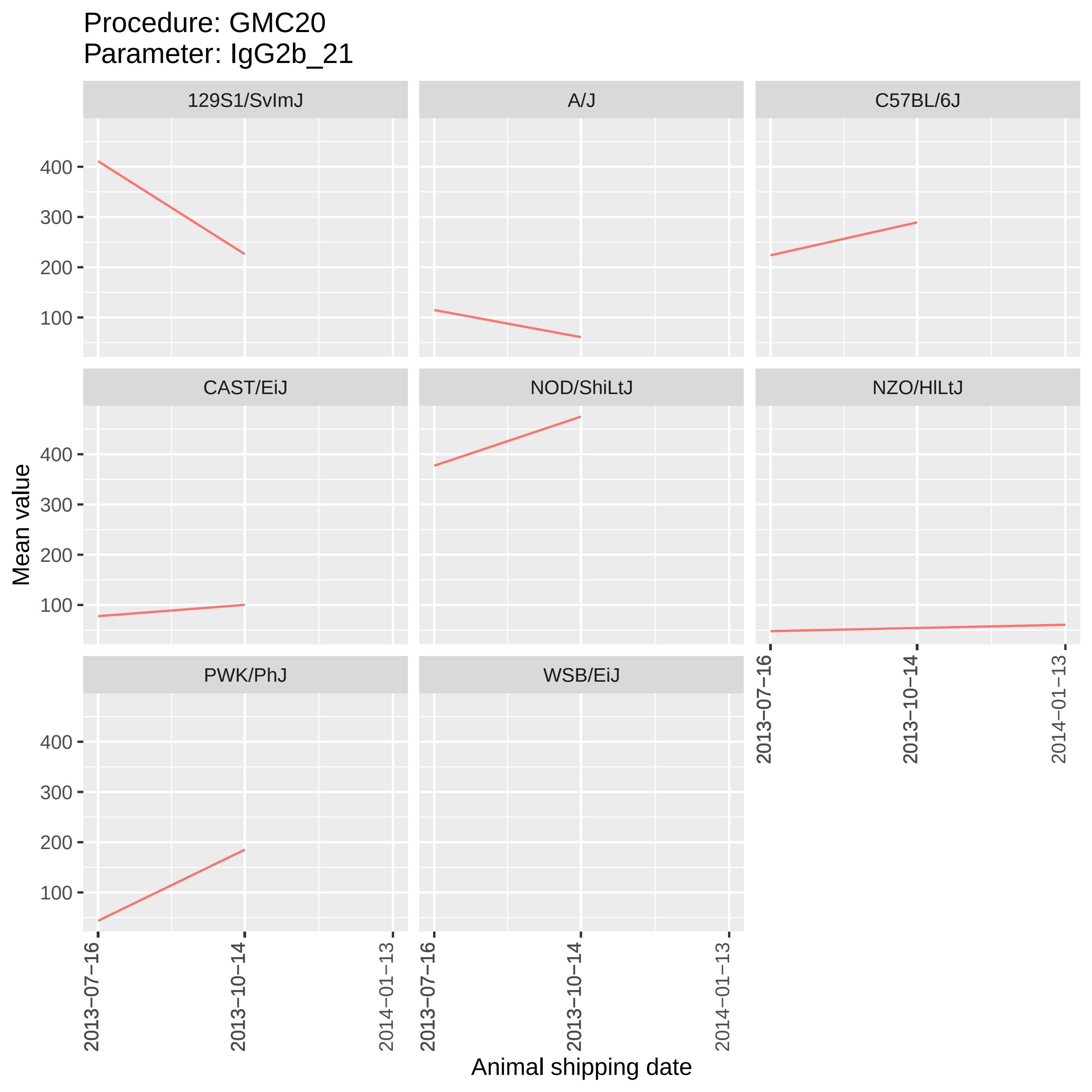

Procedure: GMC20
Parameter: IgG2b_21
129S1/SvImJ
A/J
C57BL/6J
CAST/EiJ
NOD/ShiLtJ
NZO/HlLtJ
PWK/PhJ
WSB/EiJ
Mean value
100
200
300
400
2013−07−16
2013−10−14
2014−01−13
Animal shipping date

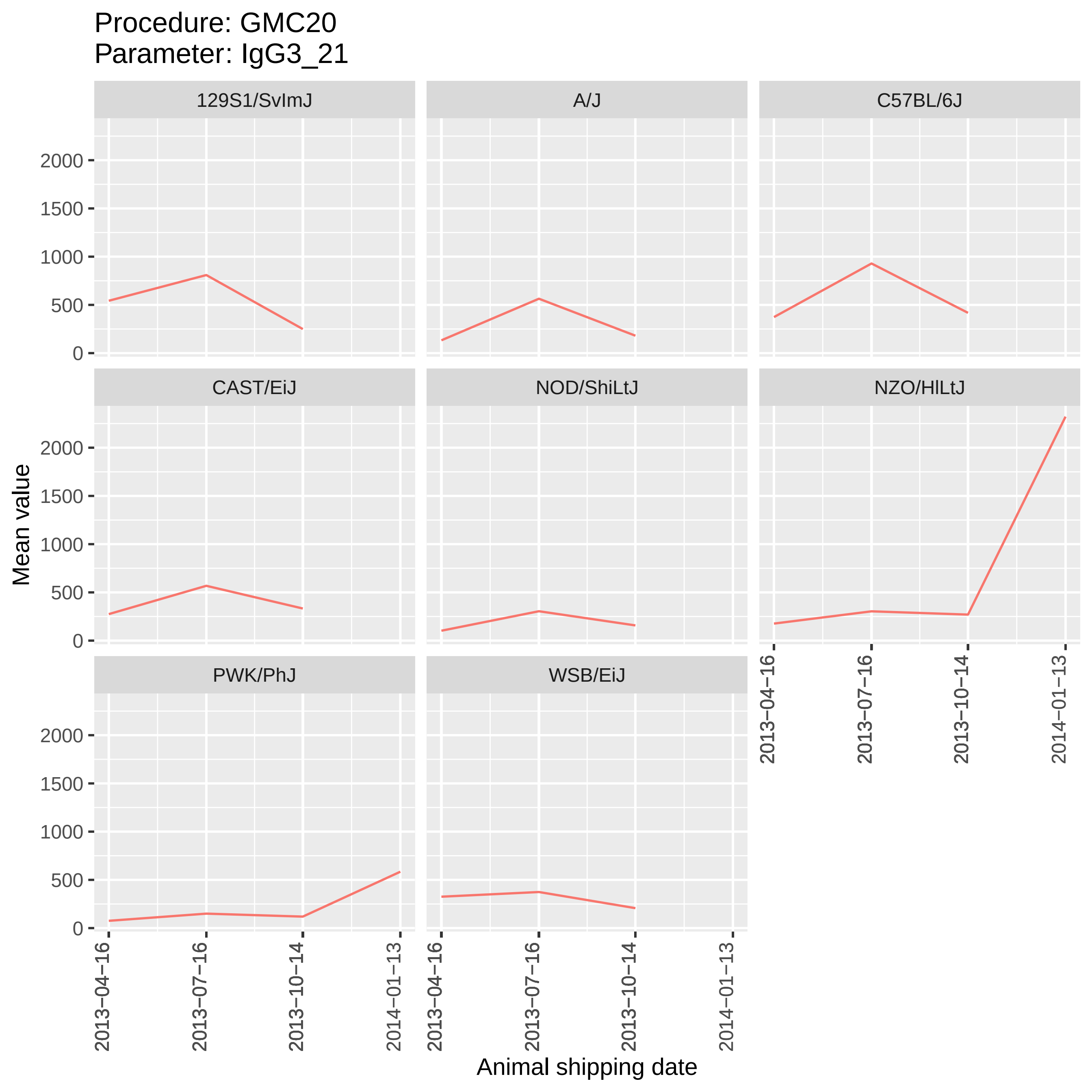

Procedure: GMC20
Parameter: IgG3_21
129S1/SvImJ
A/J
C57BL/6J
CAST/EiJ
NOD/ShiLtJ
NZO/HlLtJ
PWK/PhJ
WSB/EiJ
Mean value
0
500
1000
1500
2000
2013−04−16
2013−07−16
2013−10−14
2014−01−13
Animal shipping date

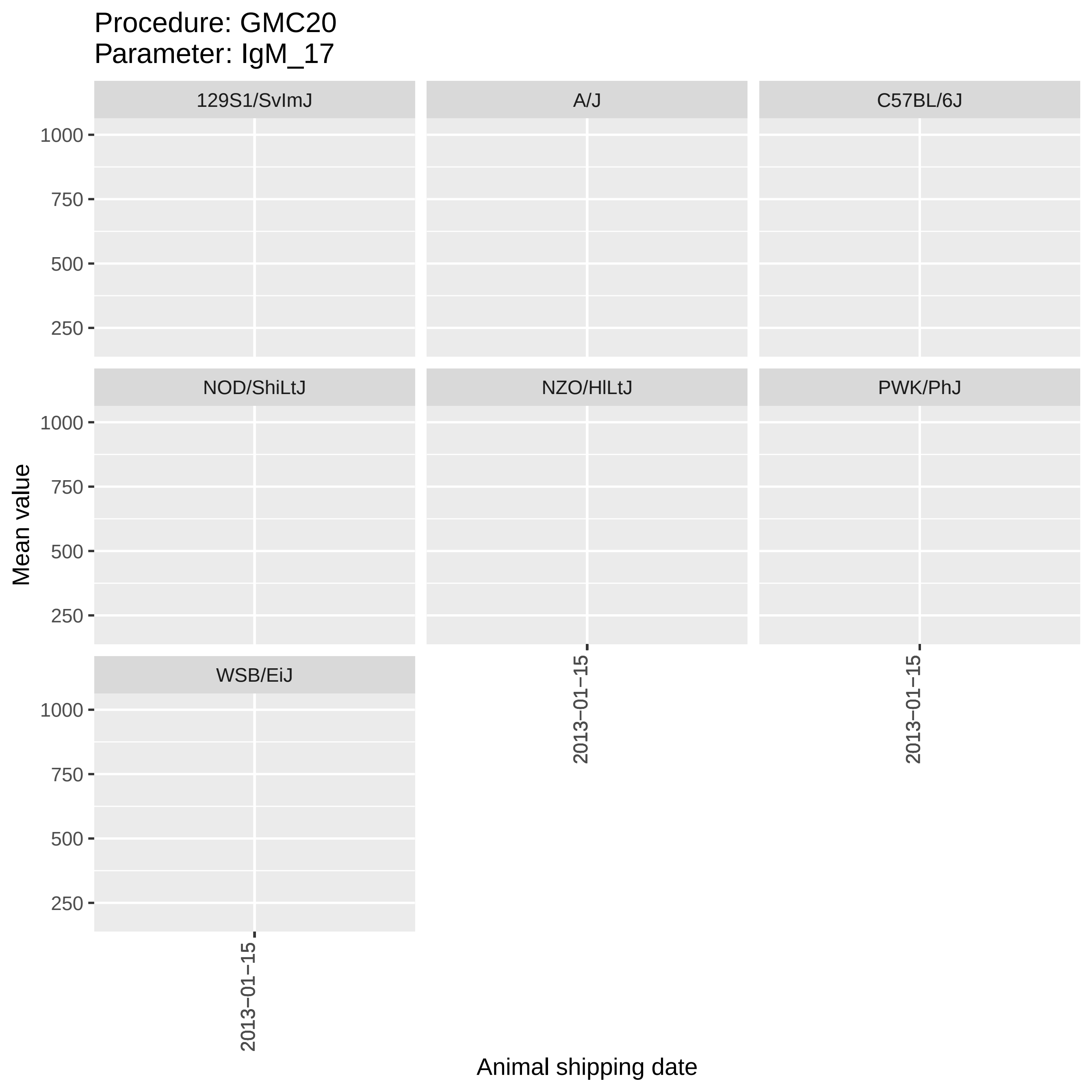
Procedure: GMC20
Parameter: IgM_17
129S1/SvImJ
A/J
C57BL/6J
NOD/ShiLtJ
NZO/HlLtJ
PWK/PhJ
WSB/EiJ
1000
750
500
250
Mean value
2013−01−15
Animal shipping date

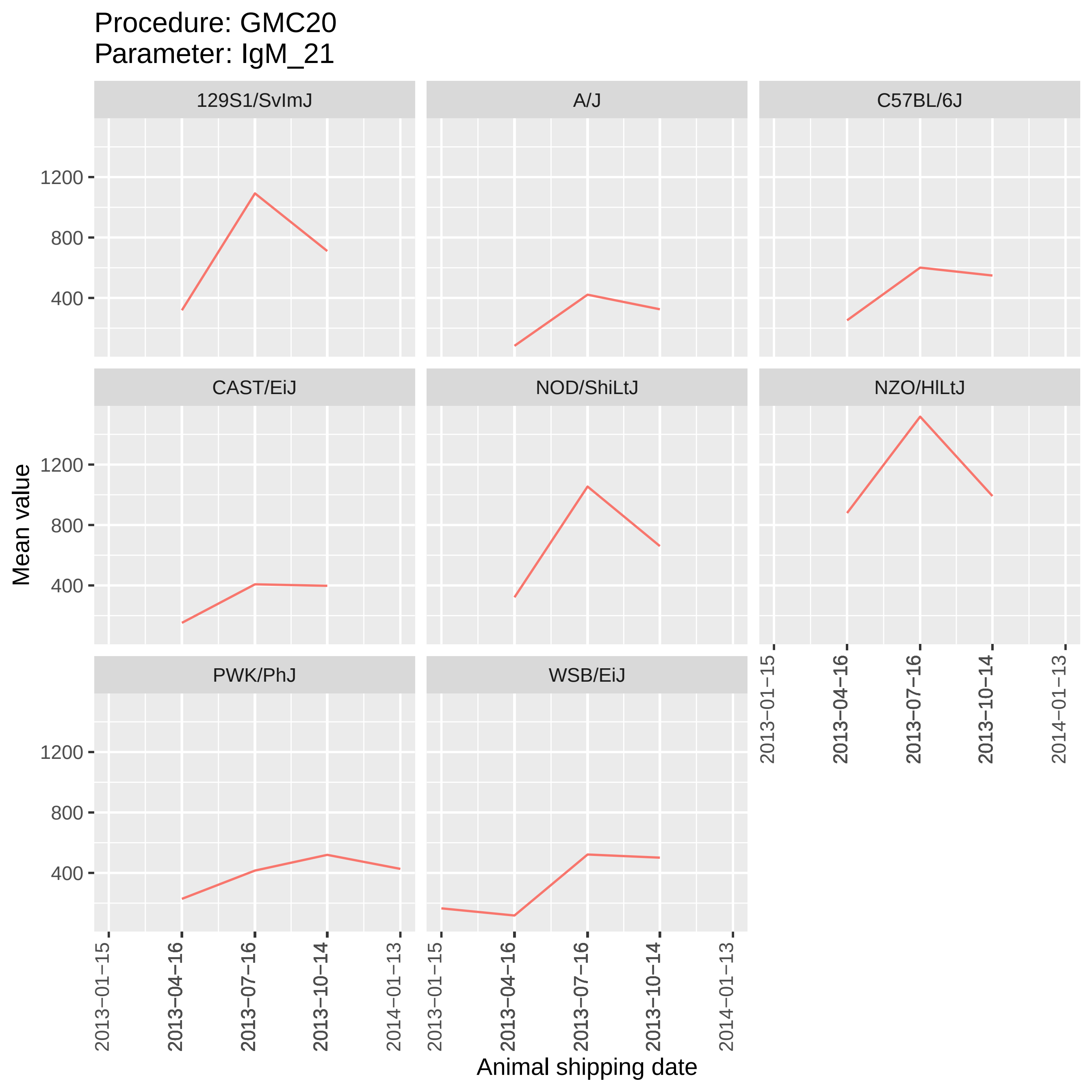
Procedure: GMC20
Parameter: IgM_21
129S1/SvImJ
A/J
C57BL/6J
CAST/EiJ
NOD/ShiLtJ
NZO/HlLtJ
PWK/PhJ
WSB/EiJ
Mean value
400
800
1200
2013−01−15
2013−04−16
2013−07−16
2013−10−14
2014−01−13
Animal shipping date

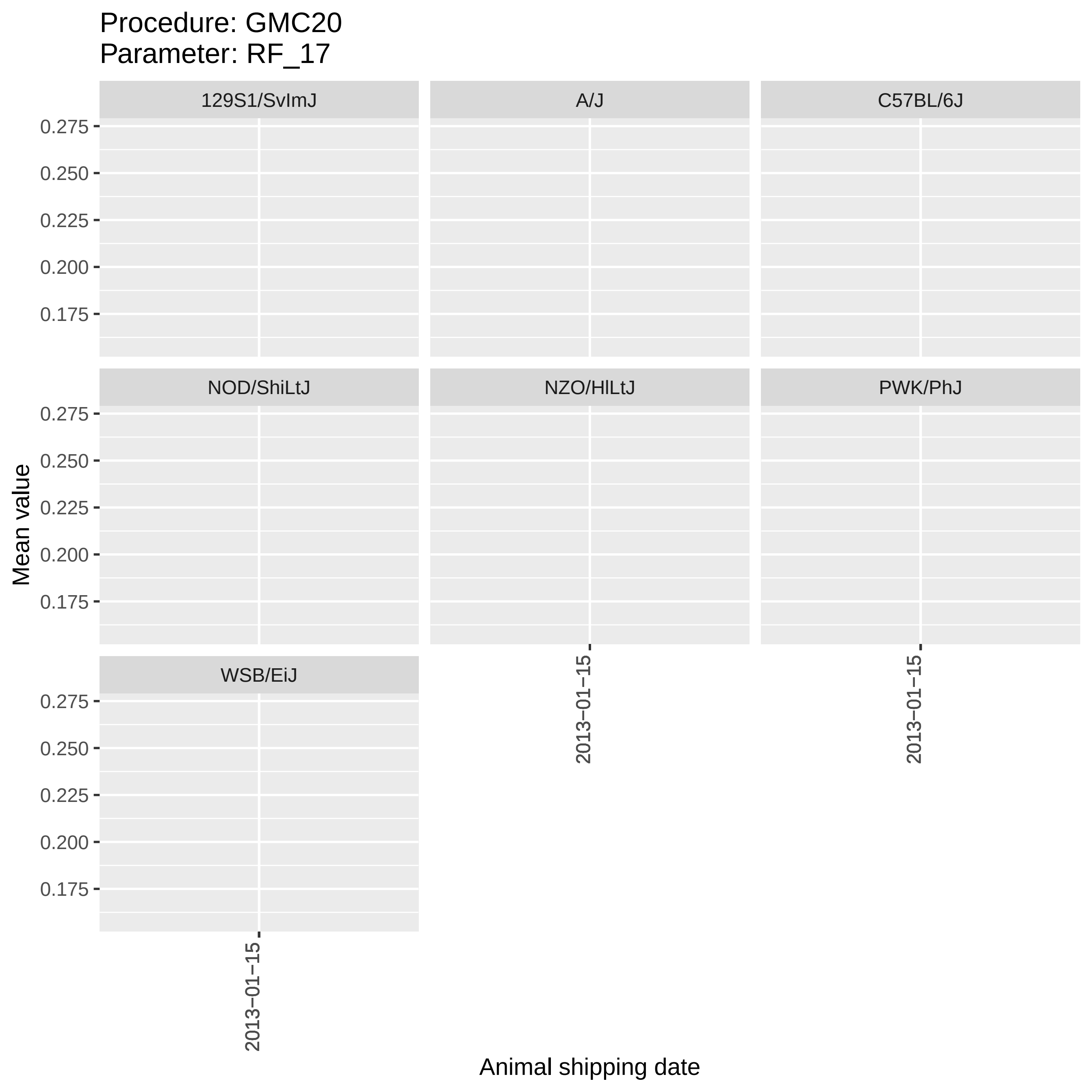
Procedure: GMC20
Parameter: RF_17
129S1/SvImJ
A/J
C57BL/6J
NOD/ShiLtJ
NZO/HlLtJ
PWK/PhJ
WSB/EiJ
Mean value
0.275
0.250
0.225
0.200
0.175
2013−01−15
Animal shipping date

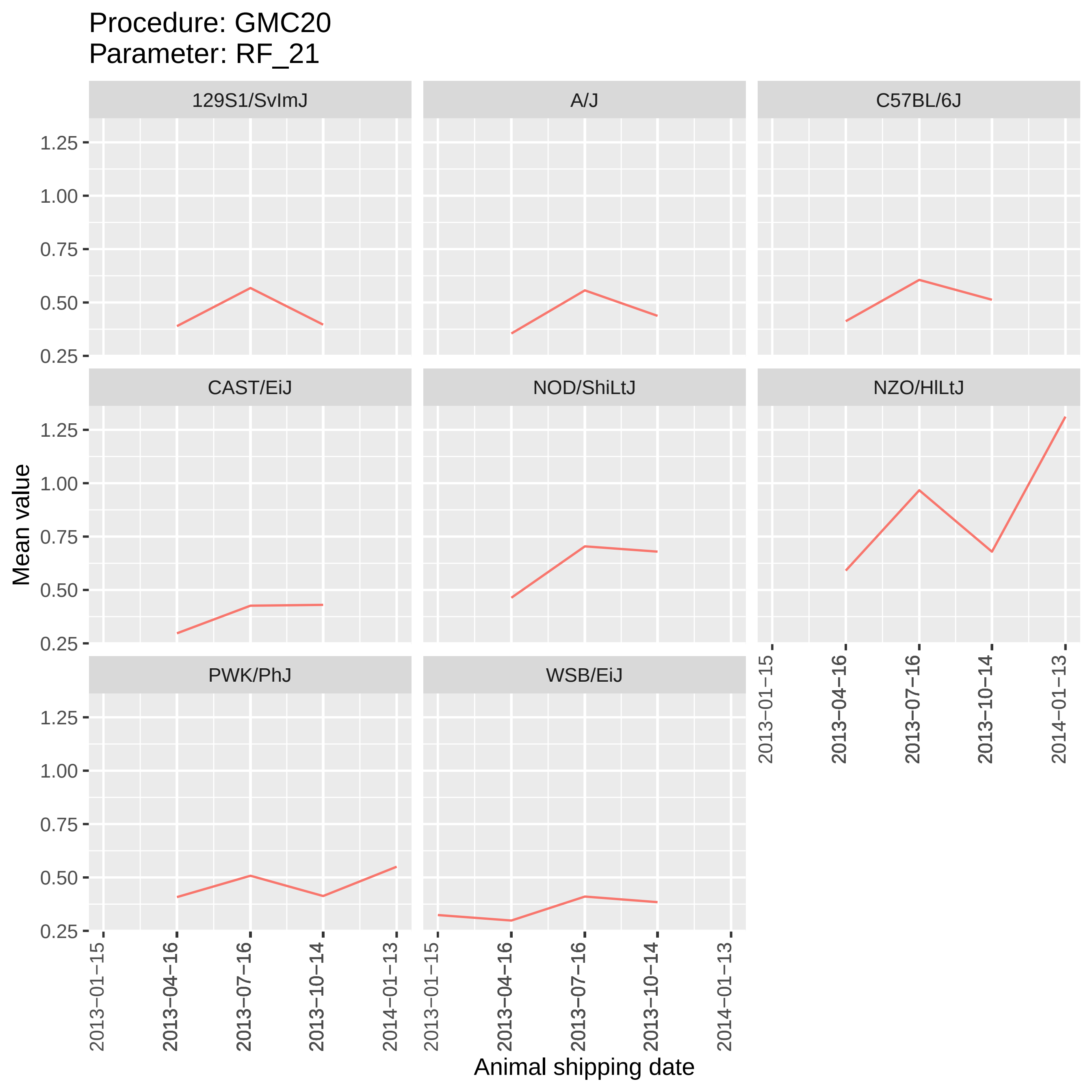

Procedure: GMC20
Parameter: RF_21
129S1/SvImJ
A/J
C57BL/6J
CAST/EiJ
NOD/ShiLtJ
NZO/HlLtJ
PWK/PhJ
WSB/EiJ
Mean value
0.25
0.50
0.75
1.00
1.25
2013−01−15
2013−04−16
2013−07−16
2013−10−14
2014−01−13
Animal shipping date

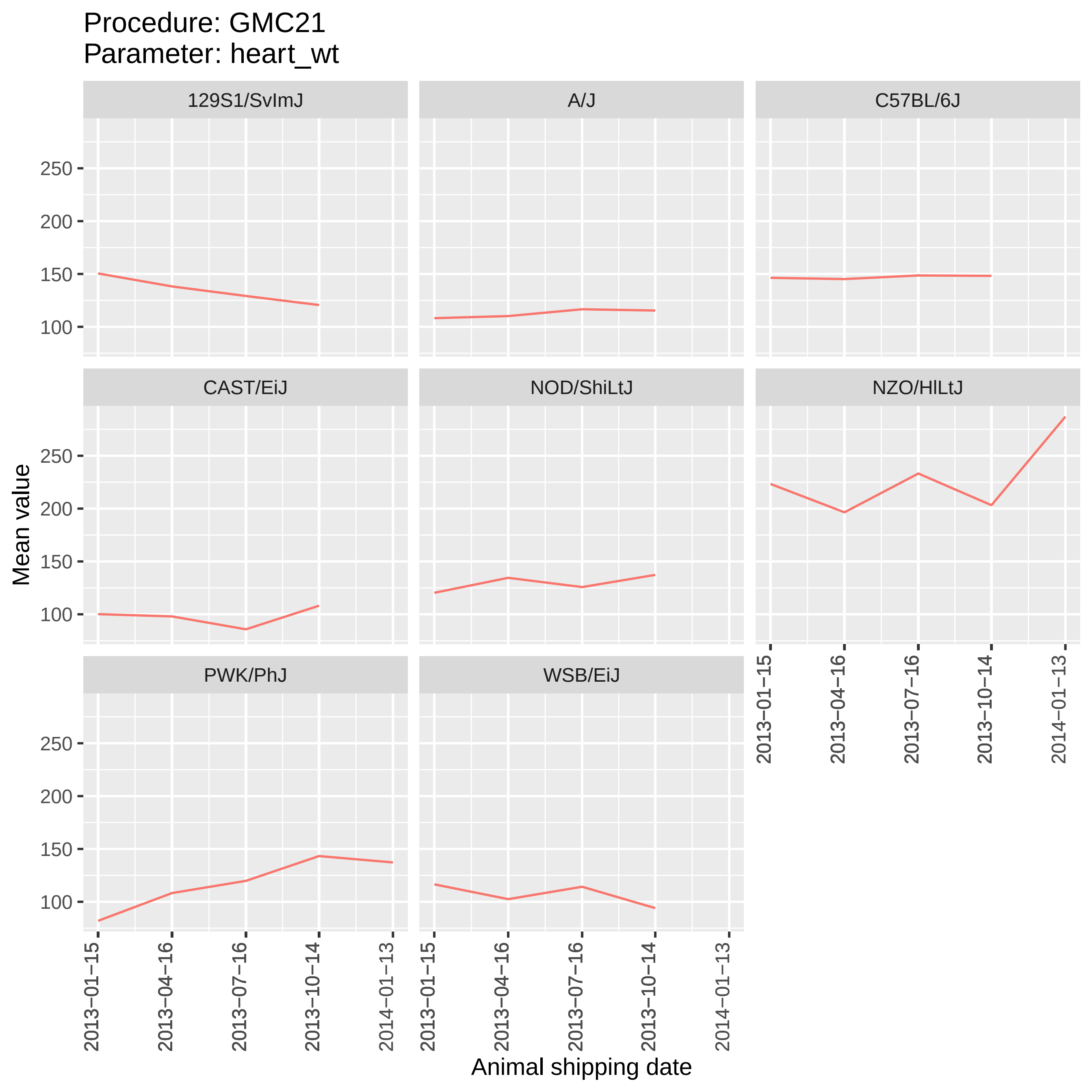

Procedure: GMC21
Parameter: heart_wt
129S1/SvImJ
A/J
C57BL/6J
CAST/EiJ
NOD/ShiLtJ
NZO/HlLtJ
PWK/PhJ
WSB/EiJ
Mean value
100
150
200
250
2013−01−15
2013−04−16
2013−07−16
2013−10−14
2014−01−13
Animal shipping date

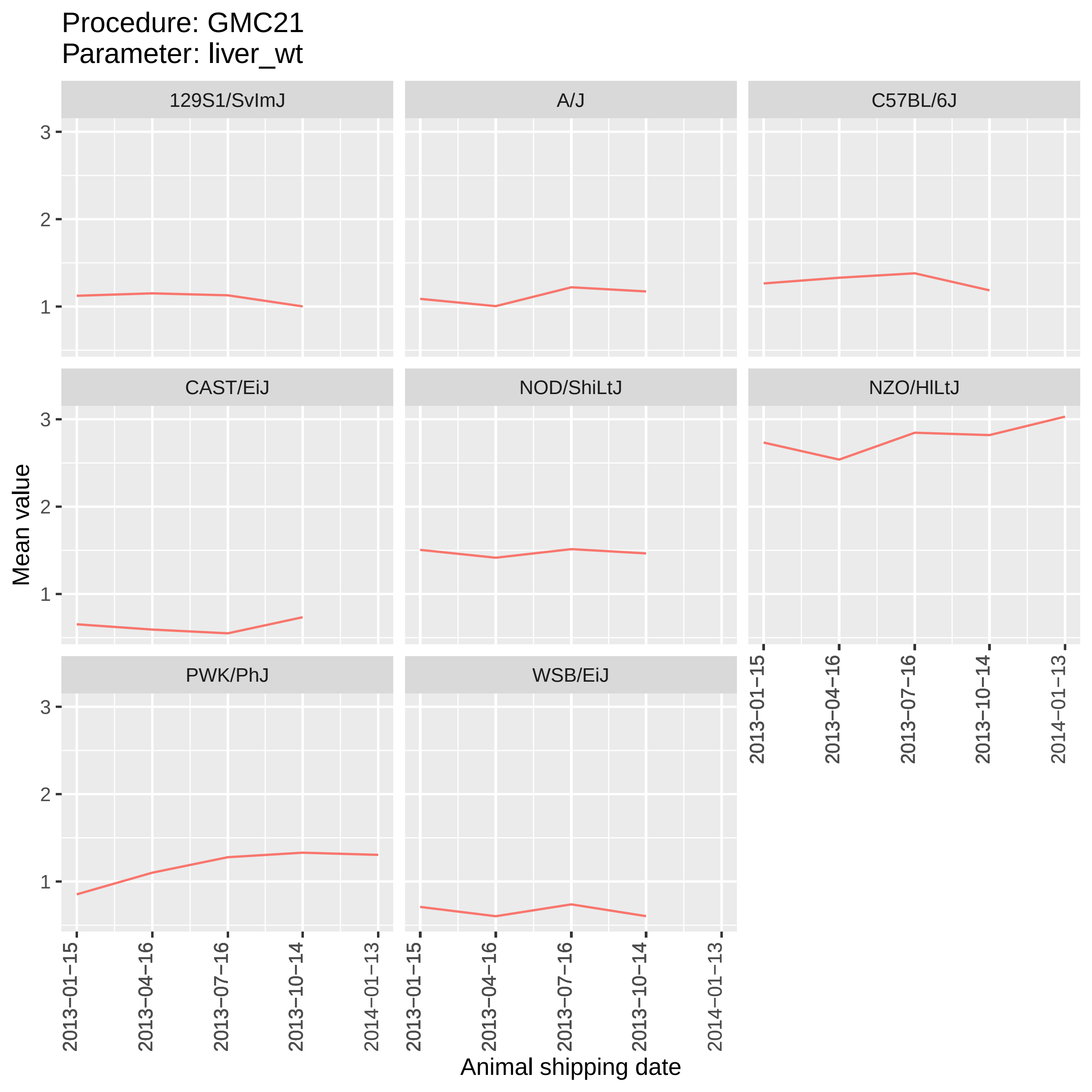

Procedure: GMC21
Parameter: liver_wt
129S1/SvImJ
A/J
C57BL/6J
CAST/EiJ
NOD/ShiLtJ
NZO/HlLtJ
PWK/PhJ
WSB/EiJ
Mean value
1
2
3
2013−01−15
2013−04−16
2013−07−16
2013−10−14
2014−01−13
Animal shipping date

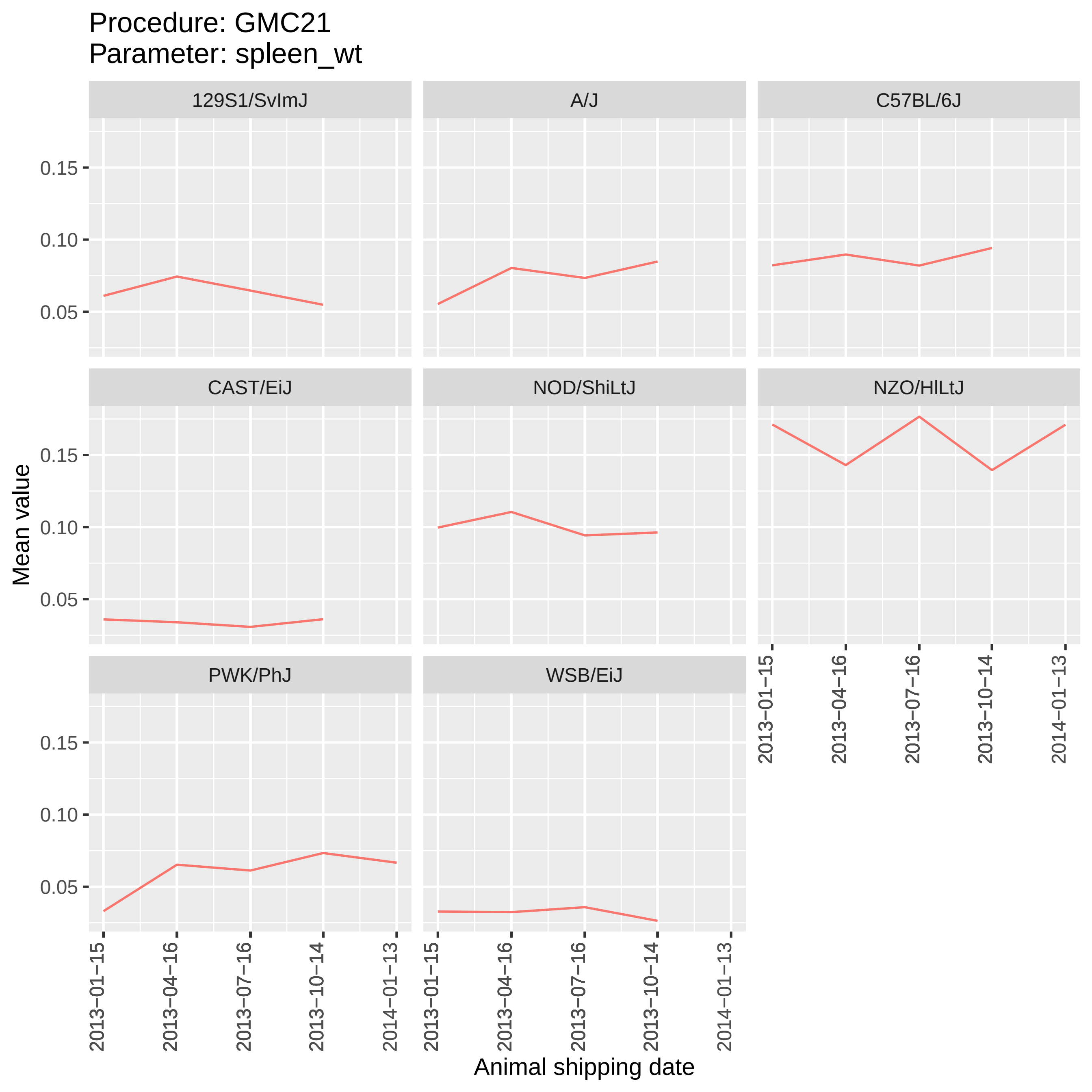

Procedure: GMC21
Parameter: spleen_wt
129S1/SvImJ
A/J
C57BL/6J
CAST/EiJ
NOD/ShiLtJ
NZO/HlLtJ
PWK/PhJ
WSB/EiJ
Mean value
0.05
0.10
0.15
2013−01−15
2013−04−16
2013−07−16
2013−10−14
2014−01−13
Animal shipping date

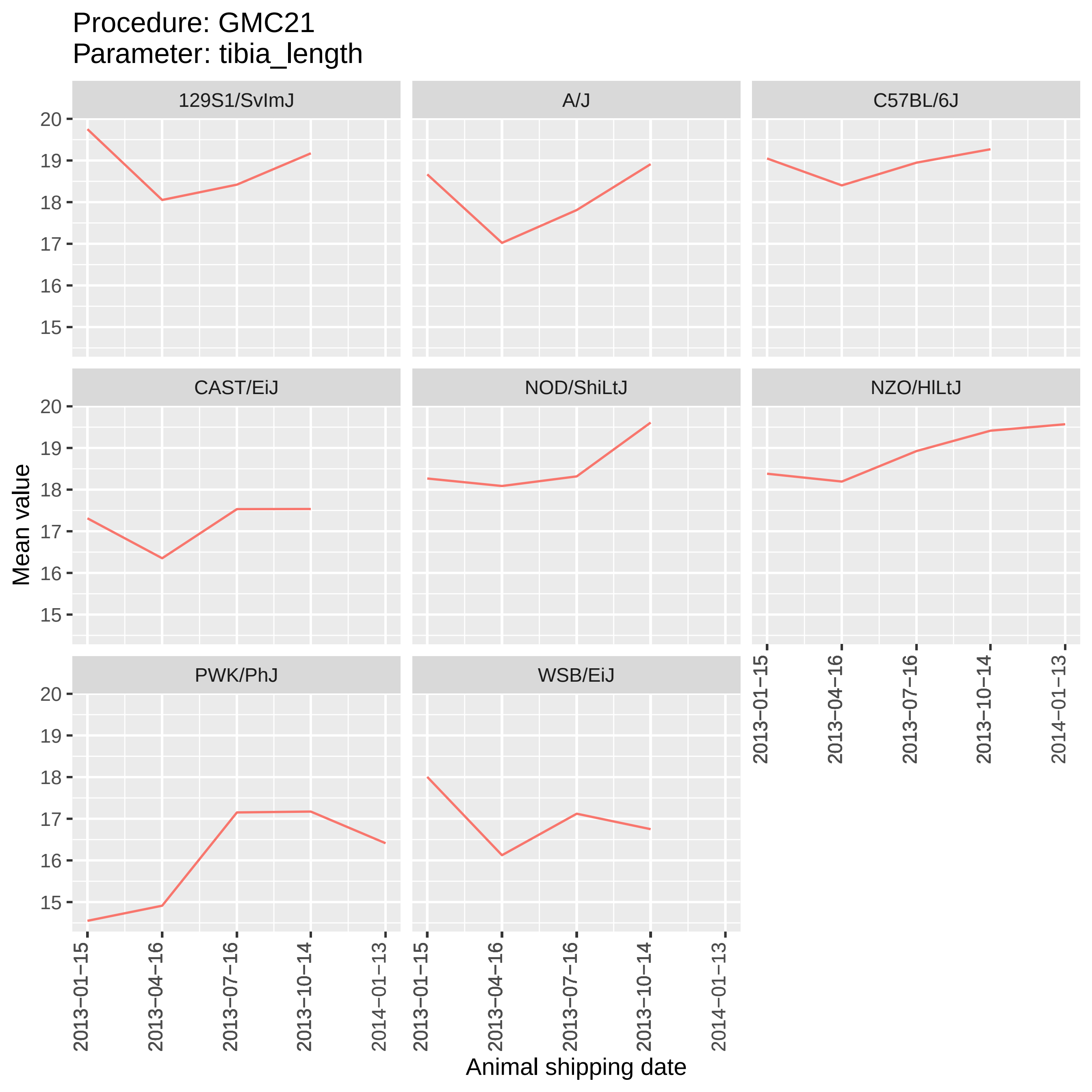
Procedure: GMC21
Parameter: tibia_length
129S1/SvImJ
A/J
C57BL/6J
CAST/EiJ
NOD/ShiLtJ
NZO/HlLtJ
PWK/PhJ
WSB/EiJ
Mean value
15
16
17
18
19
20
2013−01−15
2013−04−16
2013−07−16
2013−10−14
2014−01−13
Animal shipping date
