# Supplementary material for: A comprehensive and comparative phenotypic analysis of the collaborative founder strains identifies new and known phenotypes
Source: Mamm Genome. 2020 Feb 14;31(1):30–48. doi: 10.1007/s00335-020-09827-3 (PMC7060152; doi:10.1007/s00335-020-09827-3)
Supplement: Supplementary file 6 — Supplementary file6 (PDF 120 kb) [file 335_2020_9827_MOESM6_ESM.pdf]

Table S1

measnum	projsym	varname	descrip	units	aspect	mean_sq	sum_sq	precise_pvalue_prf	adj.p.value.BH
55001	GMC01	bw	body weight	g	strain	2623,242	18362,6939	9,87E-166	7,66E-164
55011	GMC01	distance_1	distance traveled, successive 5 min intervals	cm	strain	373535763,5	2614750345	4,34E-99	1,09E-97
55012	GMC01	distance_2	distance traveled, successive 5 min intervals	cm	strain	305606607,4	2139246252	2,55E-92	5,88E-91
55013	GMC01	distance_3	distance traveled, successive 5 min intervals	cm	strain	243582663,4	1705078644	1,54E-92	3,65E-91
55014	GMC01	distance_4	distance traveled, successive 5 min intervals	cm	strain	237943416,9	1665603918	7,30E-100	1,89E-98
55015	GMC01	distance_total	distance traveled total, 20 min test	cm	strain	4567184115	31970288804	6,44E-109	2,04E-107
55021	GMC01	rears_1	number of rears, successive 5 min intervals	n	strain	6519,6112	45637,2785	9,88E-54	8,36E-53
55022	GMC01	rears_2	number of rears, successive 5 min intervals	n	strain	9266,4665	64865,2654	2,79E-68	3,40E-67
55023	GMC01	rears_3	number of rears, successive 5 min intervals	n	strain	8193,6927	57355,8487	7,72E-68	9,16E-67
55024	GMC01	rears_4	number of rears, successive 5 min intervals	n	strain	7962,4287	55737,0007	1,41E-66	1,62E-65
55025	GMC01	rears_total	number of rears total, 20 min test	n	strain	118132,0042	826924,0292	2,02E-78	2,74E-77
55031	GMC01	whole_rest	resting time in whole arena, 20 min test	s	strain	169879,4337	1189156,036	1,82E-79	2,55E-78
55032	GMC01	whole_speed	average speed in whole arena, 20 min test	cm/s	strain	3840,1979	26881,3855	2,01E-111	6,61E-110
55041	GMC01	center_distance	distance traveled in center of arena, 20 min test	cm	strain	632749723,8	4429248067	1,17E-114	4,16E-113
55042	GMC01	center_rest	resting time in center of arena, 20 min test	s	strain	853,9171	5977,42	3,52E-19	1,51E-18
55043	GMC01	center_permanence	permanence time in center of arena, 20 min test	s	strain	385168,1725	2696177,207	6,24E-87	1,20E-85
55044	GMC01	center_speed	average speed in center of arena, 20 min test	cm/s	strain	5620,2725	39341,9077	1,27E-53	1,05E-52
55045	GMC01	center_latency	latency to enter in center of arena, 20 min test	s	strain	3414492,912	23901450,38	2,08E-50	1,59E-49
55046	GMC01	center_entries	number of entries in center of arena, 20 min test	n	strain	1053307,113	7373149,789	5,64E-115	2,10E-113
55051	GMC01	center_distance_1	center distance, successive 5 min intervals	%	strain	3450,2928	24152,0494	1,62E-81	2,47E-80
55052	GMC01	center_distance_2	center distance, successive 5 min intervals	%	strain	4880,1108	34160,7759	4,17E-84	6,72E-83
55053	GMC01	center_distance_3	center distance, successive 5 min intervals	%	strain	6071,3226	42499,2582	1,39E-76	1,83E-75
55054	GMC01	center_distance_4	center distance, successive 5 min intervals	%	strain	6037,8064	42264,6448	5,45E-83	8,46E-82
55055	GMC01	center_distance_total	percentage of total distance in center, 20 min test	%	strain	4870,5918	34094,1427	2,21E-108	6,74E-107
55061	GMC01	center_time_1	center time, successive 5 min intervals	%	strain	1758,0384	12306,269	1,67E-67	1,95E-66
55062	GMC01	center_time_2	center time, successive 5 min intervals	%	strain	2287,0182	16009,1277	7,98E-59	8,02E-58
55063	GMC01	center_time_3	center time, successive 5 min intervals	%	strain	3441,1799	24088,2594	1,47E-52	1,17E-51
55064	GMC01	center_time_4	center time, successive 5 min intervals	%	strain	3650,9348	25556,5438	6,73E-64	7,46E-63
55065	GMC01	center_time_total	percentage of total time spent in center, 20 min test	%	strain	2674,8994	18724,2961	6,46E-87	1,20E-85
55071	GMC01	periphery_distance	distance traveled in periphery, 20 min test	cm	strain	1854820364	12983742549	4,96E-87	1,01E-85
55072	GMC01	periphery_rest	resting time in periphery, 20 min test	s	strain	153654,5703	1075581,992	7,89E-84	1,25E-82
55073	GMC01	periphery_permanence	permanence time in periphery, 20 min test	s	strain	385175,4796	2696228,357	6,24E-87	1,20E-85
55074	GMC01	periphery_speed	average speed in periphery, 20 min test	cm/s	strain	3190,4426	22333,0985	1,21E-107	3,56E-106
55101	GMC02	bw	body weight	g	strain	2876,4417	20135,0921	1,63E-180	2,78E-178
55111	GMC02	front_paws1	forelimb grip strength	g	strain	11161,9119	78133,3832	2,15E-40	1,35E-39
55112	GMC02	front_paws2	forelimb grip strength	g	strain	10936,6201	76556,3406	1,49E-42	9,80E-42
55113	GMC02	front_paws3	forelimb grip strength	g	strain	11553,9522	80877,6657	7,59E-42	4,91E-41
55114	GMC02	front_paws_mean	forelimb grip strength, mean	g	strain	11171,3229	78199,2603	8,56E-49	6,41E-48
55115	GMC02	front_paws_adj	forelimb grip strength (mean) normalized to body we	ratio	strain	44,0589	308,4123	2,89E-58	2,81E-57
55121	GMC02	all_paws1	forelimb and hindlimb grip strength	g	strain	42574,514	298021,5978	4,17E-58	3,91E-57
55122	GMC02	all_paws2	forelimb and hindlimb grip strength	g	strain	39793,9737	278557,8156	9,63E-57	8,66E-56
55123	GMC02	all_paws3	forelimb and hindlimb grip strength	g	strain	38343,327	268403,2889	2,50E-53	2,04E-52
55124	GMC02	all_paws_mean	forelimb and hindlimb grip strength, mean	g	strain	40090,265	280631,8548	1,89E-61	1,97E-60
55125	GMC02	all_paws_adj	forelimb and hindlimb grip strength (mean) normalize	ratio	strain	133,1023	931,7162	1,97E-61	2,03E-60
55201	GMC03	bw	body weight	g	strain	2876,4417	20135,0921	1,63E-180	2,78E-178
55211	GMC03	coat_app	coat appearance (1=tidy, 2=irregular)	score	strain	0,6091	4,2639	8,24E-13	3,14E-12
55212	GMC03	whiskers	whiskers (0=present, 1=absent)	score	strain	3,9898	27,9286	7,76E-156	5,10E-154
55213	GMC03	body_pos	body position (0=inactive, 1=active, 2=excessively act	score	strain	0,6929	4,8505	3,44E-16	1,41E-15
55214	GMC03	pelvic_elev	pelvic elevation (0=less than 5, 1=5 or more, 2=no dat	score	strain	6,4353	45,0472	2,64E-65	2,97E-64
55215	GMC03	tail_elev	tail elevation (0=dragging, 1=horizontal, 2=elevated)	score	strain	3,4624	24,2368	5,99E-33	3,43E-32
55216	GMC03	transfer_arousal	transfer arousal (0=prolonged freeze, 1=brief freeze,	score	strain	4,978	34,8457	6,37E-28	3,38E-27
55221	GMC03	defecation	defecation (0=present, 1=absent)	score	strain	0,1332	0,9327	6,10E-08	1,83E-07
55222	GMC03	urinate	urination (0=present, 1=absent)	score	strain	1,8366	12,8563	4,46E-10	1,54E-09
55224	GMC03	loco_activity	total number of squares entered with all four paws in	n	strain	3462,0891	24234,6235	1,43E-53	1,18E-52
55225	GMC03	bite_evidence	evidence of biting (0=not aggressive, 1=aggressive)	score	strain	0,6804	4,7625	2,81E-16	1,16E-15
55226	GMC03	vocalization	vocalization (0=no, 1=yes)	score	strain	0,5108	3,5757	4,38E-14	1,72E-13
55233	GMC03	startle_response	startle response, click box (0=no reaction, 1=Preyer re	score	strain	2,5003	17,5019	1,30E-23	6,48E-23
55251	GMC03	touch_escape	touch escape (0=no response, 1=response to touch, 2	score	strain	0,9292	6,5041	1,29E-26	6,67E-26
55301	GMC04	bw	body weight	g	strain	3135,5788	21949,0514	1,98E-184	8,46E-182
55311	GMC04	LatFall_1	latency to fall from accelerating rotarod (8rpm/min)	s	strain	41157,2985	246943,791	2,52E-07	7,34E-07
55312	GMC04	LatFall_2	latency to fall from accelerating rotarod (8rpm/min)	s	strain	46254,6166	277527,6993	3,92E-06	1,06E-05
55313	GMC04	LatFall_3	latency to fall from accelerating rotarod (8rpm/min)	s	strain	62731,1509	376386,9057	1,88E-09	6,21E-09
55315	GMC04	LatFall_mean	latency to fall from accelerating (8rpm/min) rotarod,	s	strain	45849,2011	275095,2066	3,23E-11	1,17E-10
55401	GMC05	bw	body weight	g	strain	3061,5994	21431,1956	6,29E-169	5,37E-167
55411	GMC05	ASR_bn	acoustic startle response (ASR)	amplitude	strain	160067,8341	1120474,839	5,10E-07	1,45E-06
55412	GMC05	ASR_70	acoustic startle response (ASR)	amplitude	strain	119832,5957	838828,1701	1,34E-14	5,34E-14
55413	GMC05	ASR_80	acoustic startle response (ASR)	amplitude	strain	288729,7712	2021108,398	4,08E-23	1,98E-22
55414	GMC05	ASR_85	acoustic startle response (ASR)	amplitude	strain	632838,6597	4429870,618	2,53E-29	1,38E-28
55415	GMC05	ASR_90	acoustic startle response (ASR)	amplitude	strain	2727450,113	19092150,79	1,91E-56	1,70E-55
55416	GMC05	ASR_100	acoustic startle response (ASR)	amplitude	strain	15146862,92	106028040,4	2,36E-115	9,15E-114
55417	GMC05	ASR_110	acoustic startle response (ASR)	amplitude	strain	21684546,66	151791826,6	1,14E-116	4,88E-115
55418	GMC05	ASR_120	acoustic startle response (ASR)	amplitude	strain	22979172,59	160854208,2	3,90E-125	1,85E-123
55421	GMC05	ASR_PP_67	acoustic startle response (ASR), 110 db sound pressu	amplitude	strain	14495924,18	101471469,2	2,39E-84	3,92E-83
55422	GMC05	ASR_PP_69	acoustic startle response (ASR), 110 db sound pressu	amplitude	strain	14375570	100628990	2,40E-86	4,37E-85
55423	GMC05	ASR_PP_73	acoustic startle response (ASR), 110 db sound pressu	amplitude	strain	13466872,13	94268104,9	3,48E-81	5,03E-80
55424	GMC05	ASR_PP_81	acoustic startle response (ASR), 110 db sound pressu	amplitude	strain	7523466,296	52664264,07	8,16E-79	1,12E-77
55431	GMC05	PPI_67	percent prepulse inhibition (PPI), 110 db sound press	%	strain	11997,6596	83983,617	1,83E-22	8,63E-22
55432	GMC05	PPI_69	percent prepulse inhibition (PPI), 110 db sound press	%	strain	16837,5578	117862,9048	5,12E-38	3,14E-37
55433	GMC05	PPI_73	percent prepulse inhibition (PPI), 110 db sound press	%	strain	20897,1144	146279,8008	3,48E-31	1,96E-30
55434	GMC05	PPI_81	percent prepulse inhibition (PPI), 110 db sound press	%	strain	17519,3472	122635,4303	1,05E-41	6,75E-41
55435	GMC05	PPI_global	percentage prepulse inhibition (PPI) evoked by 110 dl	%	strain	16071,3141	112499,199	1,69E-38	1,05E-37
55441	GMC05	ASR_ISI_5	acoustic startle response (ASR)	amplitude	strain	12346120,16	86422841,15	2,05E-81	3,07E-80
55442	GMC05	ASR_ISI_25	acoustic startle response (ASR)	amplitude	strain	13602111,1	95214777,72	1,41E-91	3,16E-90
55443	GMC05	ASR_ISI_100	acoustic startle response (ASR)	amplitude	strain	17049324,56	119345271,9	3,94E-106	1,12E-104
55451	GMC05	PPI_ISI_5	prepulse inhibition (PPI)	%	strain	19527,0646	136689,4524	1,72E-43	1,16E-42
55452	GMC05	PPI_ISI_25	prepulse inhibition (PPI)	%	strain	18819,4799	131736,3595	3,24E-32	1,84E-31
55453	GMC05	PPI_ISI_100	prepulse inhibition (PPI)	%	strain	9874,2354	69119,6477	1,02E-22	4,84E-22
55501	GMC06	bw_before_fast	body weight	g	strain	2515,6699	17609,689	9,43E-122	4,24E-120
55502	GMC06	bw_after_fast	body weight	g	strain	2849,3597	11397,4389	8,42E-94	2,05E-92
55511	GMC06	GLU	glucose (plasma GLU, 16h fast)	mmol/L	strain	192,5128	770,0512	1,44E-35	8,51E-35
55512	GMC06	Glycerol	glycerol (plasma, 16h fast)	mmol/L	strain	0,0431	0,1726	8,29E-06	2,19E-05
55521	GMC06	CHOL	total cholesterol (plasma CHOL, 16h fast)	mmol/L	strain	76,8812	307,5248	2,01E-80	2,86E-79
55522	GMC06	HDL	HDL cholesterol (plasma HDL, 16h fast)	mmol/L	strain	24,394	97,5759	2,76E-81	4,06E-80

55523	GMC06	nonHDL	non-HDL cholesterol (plasma non-HDL) (CHOL minus	mmol/L	strain	16,0066	64,0263	6,00E-57	5,45E-56
55524	GMC06	NEFA	non-esterified fatty acids (plasma NEFA, 16h fast)	mmol/L	strain	0,489	1,9561	0,02133978	0,035682736
55525	GMC06	TG	triglyceride (plasma TG, 16h fast)	mmol/L	strain	5,4368	21,7472	1,08E-09	3,62E-09
55601	GMC07	bw	body weight	g	strain	3917,3538	27421,4764	1,01E-179	1,23E-177
55612	GMC07	latency_2	latency of nociceptive response to hot plate	s	strain	699,6954	4897,8678	2,54E-23	1,25E-22
55621	GMC07	response_1	type of response to hot plate, first reaction (0=no rea	designation	strain	1,0402	7,2814	0,001678501	0,003437506
55622	GMC07	response_2	type of response to hot plate, second reaction (0=no	designation	strain	6,2056	43,4391	3,84E-09	1,23E-08
55701	GMC08	TEWL_adj	transepidermal water loss, normalized	g/m²/h	strain	762,1762	5335,2336	1,08E-19	4,67E-19
55702	GMC08	TEWL_CV	coefficient of variation of transepidermal water loss s	%	strain	0,2328	1,6299	1,68E-05	4,34E-05
55801	GMC09	bw_before	body weight before testing	g	strain	4073,712	28515,9837	1,31E-181	3,72E-179
55802	GMC09	bw_after	body weight after testing	g	strain	3876,966	27138,7617	5,98E-180	8,51E-178
55811	GMC09	food	total food intake, 21 h test	g	strain	22,1204	154,843	4,57E-37	2,73E-36
55821	GMC09	water	total water consumption, 21 h test	mL	strain	59,6813	417,7693	1,73E-07	5,08E-07
55831	GMC09	VO2_mean	mean oxygen consumption, 21 h test, 15 min bins	mL/h	strain	9067,0945	63469,6616	1,58E-88	3,29E-87
55832	GMC09	VCO2_mean	mean carbon dioxide production, 21 h test, 15 min bi	mL/h	strain	7450,7249	52155,0743	5,63E-85	9,43E-84
55833	GMC09	RER_mean	mean respiratory exchange rate, 21 h test, 15 min bir	ratio	strain	0,061	0,427	1,99E-10	6,99E-10
55841	GMC09	heat_mean	mean heat production, 21 h test, 15 min bins	kJ/h	strain	3,8539	26,9772	9,71E-90	2,07E-88
55851	GMC09	breaks_X_mean	mean total beam breaks on X-axis, 21 h test, 15 min b	n	strain	7264940,08	50854580,56	3,19E-07	9,13E-07
55852	GMC09	breaks_XA_mean	mean ambulatory movement on X-axis, 21 h test, 15 r	n	strain	5464960,233	38254721,63	7,07E-07	2,00E-06
55853	GMC09	breaks_XF_mean	mean fine movement on X-axis, 21 h test, 15 min bins	n	strain	157968,1529	1105777,071	2,31E-10	8,10E-10
55854	GMC09	breaks_YA_mean	mean ambulatory movement on Y-axis, 21 h test, 15 r	n	strain	5776133,24	40432932,68	2,44E-18	1,04E-17
55855	GMC09	breaks_YF_mean	mean fine movement on Y-axis, 21 h test, 15 min bins	n	strain	243131,2614	1701918,83	5,76E-26	2,96E-25
55856	GMC09	breaks_Z_mean	mean rearing movement on Z-axis, 21 h test, 15 min b	n	strain	166454,3814	1165180,67	5,98E-24	2,99E-23
55857	GMC09	distance_mean	mean distance traveled, 21 h test, 15 min bins	cm	strain	118809854,8	831668983,8	7,90E-10	2,70E-09
55858	GMC09	speed_mean	mean speed traveled, 21 h test, 15 min bins	cm/s	strain	146,8586	1028,0103	8,01E-10	2,72E-09
55901	GMC10	bw_13wk	body weight	g	strain	3985,2748	27896,9233	7,94E-190	6,78E-187
55902	GMC10	bw_19wk	body weight	g	strain	4649,7837	32548,4859	1,91E-144	1,09E-142
55911	GMC10	fat_13wk	fat tissue mass, whole body with head (NMR)	g	strain	799,7912	5598,5383	2,03E-169	1,93E-167
55912	GMC10	fat_19wk	fat tissue mass, whole body with head (NMR)	g	strain	1040,448	7283,1362	1,19E-147	7,27E-146
55921	GMC10	lean_13wk	lean tissue mass, whole body with head (NMR)	g	strain	675,9641	4731,7485	3,02E-158	2,15E-156
55922	GMC10	lean_19wk	lean tissue mass, whole body with head (NMR)	g	strain	645,4838	4518,3865	5,40E-116	2,20E-114
56001	GMC11	bw_before_fast	body weight	g	strain	4042,8615	28300,0308	1,71E-171	1,83E-169
56002	GMC11	bw_after_fast	body weight	g	strain	3636,0816	18180,4081	1,44E-111	4,92E-110
56011	GMC11	GLU_0	intraperitoneal glucose tolerance test (20% glucose i.	mmol/L	strain	28,5697	114,2789	5,77E-08	1,74E-07
56012	GMC11	GLU_15	intraperitoneal glucose tolerance test (20% glucose i.	mmol/L	strain	484,801	1939,2042	2,33E-21	1,07E-20
56013	GMC11	GLU_30	intraperitoneal glucose tolerance test (20% glucose i.	mmol/L	strain	596,4481	2385,7923	8,79E-23	4,19E-22
56014	GMC11	GLU_60	intraperitoneal glucose tolerance test (20% glucose i.	mmol/L	strain	1139,334	4557,3361	4,93E-35	2,91E-34
56015	GMC11	GLU_120	intraperitoneal glucose tolerance test (20% glucose i.	mmol/L	strain	1382,9635	5531,8539	3,08E-48	2,27E-47
56101	GMC12	bw	body weight	g	strain	3513,6486	24595,5399	4,81E-141	2,57E-139
56111	GMC12	resp_rate	respiration rate	n/min	strain	80659,2819	564614,9733	8,02E-09	2,50E-08
56112	GMC12	heart_rate	heart rate, beats per min	n/min	strain	580765,9885	4065361,92	9,59E-43	6,40E-42
56121	GMC12	LV_mass	left ventricular mass corrected	mg	strain	2064,2161	14449,5127	1,66E-41	1,06E-40
56122	GMC12	fract_shortening	fractional shortening	%	strain	3784,817	26493,7192	4,67E-34	2,73E-33
56123	GMC12	EJ_fraction	ejection fraction	%	strain	2386,9274	16708,4915	1,02E-45	7,17E-45
56124	GMC12	stroke_vol	stroke volume, volume of blood pumped from one ve	µL	strain	2560,8566	17925,9959	4,92E-42	3,21E-41
56125	GMC12	cardiac_output	cardiac output, volume of blood pumped by the hear	mL/min	strain	550,8203	3855,742	1,64E-37	9,91E-37
56131	GMC12	IVS_diastole	interventricular septum	mm	strain	0,032	0,2238	2,43E-11	8,95E-11
56132	GMC12	IVS_systole	interventricular septum	mm	strain	0,0355	0,2483	2,35E-14	9,29E-14
56133	GMC12	LVID_diastole	left ventricular internal dimension	mm	strain	8,5324	59,727	9,11E-46	6,43E-45
56134	GMC12	LVID_systole	left ventricular internal dimension	mm	strain	7,1211	49,8476	1,34E-39	8,37E-39
56135	GMC12	LVPW_diastole	left ventricular posterior wall width	mm	strain	0,0174	0,1221	0,000576219	0,001284833
56136	GMC12	LVPW_systole	left ventricular posterior wall width	mm	strain	0,03	0,2099	2,78E-07	8,00E-07
56201	GMC13	HR	heart rate, beats per min	n/min	strain	67740,7081	338703,5407	1,19E-26	6,18E-26
56202	GMC13	HR_V	heart rate variability, mean of differences between se	n/min	strain	8573,4623	42867,3115	0,011913809	0,021196651
56203	GMC13	HR_CV	heart rate coefficient of variation (signal intensity)	%	strain	137,257	686,2852	0,008275366	0,015107384
56211	GMC13	RR	duration of RR interval	ms	strain	742,5912	3712,9561	6,17E-26	3,15E-25
56212	GMC13	PQ	duration of PQ interval	ms	strain	114,6475	573,2376	1,88E-09	6,21E-09
56213	GMC13	PR	duration of PR interval	ms	strain	120,8357	604,1784	3,92E-08	1,19E-07
56214	GMC13	QRS	duration of QRS interval	ms	strain	9,6748	48,3741	6,88E-06	1,82E-05
56215	GMC13	QT	duration of QT interval	ms	strain	128,0051	640,0257	2,55E-07	7,37E-07
56216	GMC13	ST	duration of ST interval	ms	strain	120,3356	601,6778	7,79E-07	2,20E-06
56217	GMC13	QTc	duration of QTc interval, calculated by dividing QT int	ms	strain	33,6175	168,0877	0,022988067	0,038120018
56231	GMC13	SR_amplitude	mean SR amplitude, mean amplitude of signal measu	mV	strain	2,9621	14,8104	4,22E-06	1,14E-05
56232	GMC13	R_amplitude	mean R amplitude, mean amplitude of signal measur	mV	strain	1,21	6,05	9,49E-07	2,65E-06
56242	GMC13	pNN50	proportion of number of pairs of successive beat-to-b	%	strain	623,1029	3115,5145	0,021488965	0,03571598
56301	GMC14	bw	body weight	g	strain	3683,6771	25785,7398	6,92E-136	3,48E-134
56302	GMC14	body_length	body length	mm	strain	1894,3776	13260,6434	4,51E-73	5,83E-72
56321	GMC14	eye_length_L	eye axial length	mm	strain	0,4223	2,9558	1,83E-54	1,58E-53
56322	GMC14	eye_length_R	eye axial length	mm	strain	0,3064	2,1445	2,78E-66	3,16E-65
56331	GMC14	lens_min_density_L	minimal density value of lens, Scheimpflug imaging	%	strain	50,9094	356,3659	2,32E-58	2,31E-57
56332	GMC14	lens_min_density_R	minimal density value of lens, Scheimpflug imaging	%	strain	59,0222	413,1557	4,10E-51	3,18E-50
56333	GMC14	lens_max_density_L	maximal density value of lens, Scheimpflug imaging	%	strain	65,3661	457,5629	1,40E-05	3,64E-05
56334	GMC14	lens_max_density_R	maximal density value of lens, Scheimpflug imaging	%	strain	74,6054	522,2376	2,47E-11	9,06E-11
56335	GMC14	lens_mean_density_L	mean density value of lens, Scheimpflug imaging	%	strain	54,6459	382,5215	6,42E-49	4,85E-48
56336	GMC14	lens_mean_density_R	mean density value of lens, Scheimpflug imaging	%	strain	66,9476	468,6331	1,69E-44	1,15E-43
56341	GMC14	fundus_vessels_L	number of main vessels in fundus	n	strain	35,9864	251,9046	5,58E-14	2,19E-13
56342	GMC14	fundus_vessels_R	number of main vessels in fundus	n	strain	34,543	241,801	1,61E-11	5,99E-11
56351	GMC14	retinal_thickness_L	retinal thickness, optical coherence tomography	µm	strain	2901,6374	20311,4621	3,15E-21	1,43E-20
56352	GMC14	retinal_thickness_R	retinal thickness, optical coherence tomography	µm	strain	2524,0809	17668,566	1,48E-17	6,17E-17
56401	GMC15	WBC	white blood cell count (WBC; per volume x 10<sup>3	n/µL	strain	58,3339	408,3372	1,77E-14	7,03E-14
56404	GMC15	RBC	red blood cell count (RBC; per volume x 10<sup>6</s	n/µL	strain	22,614	158,2981	1,02E-20	4,50E-20
56406	GMC15	RDW	RBC corpuscular distribution width (RDW)	%	strain	19,292	135,0439	1,19E-13	4,61E-13
56408	GMC15	MCV	mean RBC corpuscular volume (MCV)	fL	strain	185,6126	1299,2879	6,86E-31	3,83E-30
56412	GMC15	MCH	calculated mean RBC corpuscular hemoglobin conten	pg	strain	12,4723	87,3063	2,99E-27	1,58E-26
56414	GMC15	MCHC	calculated mean RBC corpuscular hemoglobin concer	g/dL	strain	21,57	150,9898	6,43E-21	2,88E-20
56416	GMC15	HGB	hemoglobin (HGB)	g/dL	strain	32,5826	228,078	1,14E-13	4,43E-13
56421	GMC15	HCT	hematocrit (HCT)	%	strain	374,1468	2619,0273	1,71E-17	7,08E-17
56431	GMC15	PLT	platelet count (PLT; units per volume x 10<sup>3</su	n/µL	strain	840065,6231	5880459,362	3,83E-13	1,48E-12
56434	GMC15	MPV	mean platelet volume (MPV)	fL	strain	7,1053	49,7372	2,37E-48	1,76E-47
56436	GMC15	PDW	platelet corpuscular distribution width (PDW)	fL	strain	12,5947	88,1627	1,54E-40	9,76E-40
56438	GMC15	PLCR	platelet large cell ratio (PLCR)	%	strain	50,7898	355,5287	2,07E-15	8,34E-15
56441	GMC15	PCT	plateletcrit (PCT)	%	strain	0,5732	4,0122	2,67E-21	1,22E-20
56501	GMC16	bw_17	body weight	g	strain	1071,6894	4286,7575	3,85E-21	1,73E-20
56502	GMC16	bw_21	body weight	g	strain	3955,5776	27689,0432	8,85E-86	1,54E-84

56510	GMC16	calcium_17	calcium (plasma Ca)	mmol/L	strain	0,0432	0,1727	5,59E-05	0,000136754
56511	GMC16	calcium_21	calcium (plasma Ca)	mmol/L	strain	0,1014	0,7097	1,63E-11	6,04E-11
56512	GMC16	chloride_17	chloride (plasma Cl)	mmol/L	strain	56,6383	226,5533	2,18E-07	6,37E-07
56513	GMC16	chloride_21	chloride (plasma Cl)	mmol/L	strain	158,9034	1112,324	6,39E-20	2,77E-19
56514	GMC16	iron_17	iron (plasma Fe)	µmol/L	strain	317,3081	1269,2324	2,85E-10	9,88E-10
56515	GMC16	iron_21	iron (plasma Fe)	µmol/L	strain	664,6469	4652,5286	1,02E-28	5,42E-28
56518	GMC16	lactate_17	lactate (plasma)	mmol/L	strain	28,943	115,7722	2,07E-07	6,07E-07
56519	GMC16	lactate_21	lactate (plasma)	mmol/L	strain	135,946	951,6223	2,56E-23	1,25E-22
56520	GMC16	phosphate_17	phosphorus (plasma phosphate)	mmol/L	strain	0,7571	3,0285	0,000130653	0,0003108
56521	GMC16	phosphate_21	phosphorus (plasma phosphate)	mmol/L	strain	3,8849	27,1942	1,22E-20	5,37E-20
56523	GMC16	potassium_21	potassium (plasma K)	mmol/L	strain	0,3826	2,6781	0,029497861	0,048537906
56524	GMC16	sodium_17	sodium (plasma Na)	mmol/L	strain	50,9811	203,9245	0,00014116	0,000333935
56525	GMC16	sodium_21	sodium (plasma Na)	mmol/L	strain	94,848	663,9358	2,17E-09	7,12E-09
56526	GMC16	ALP_17	alkaline phosphatase (plasma ALP)	IU/L	strain	5241,1	20964,4	2,56E-06	6,96E-06
56527	GMC16	ALP_21	alkaline phosphatase (plasma ALP)	IU/L	strain	11651,972	81563,804	2,55E-07	7,37E-07
56529	GMC16	ALT_21	alanine transaminase (plasma ALT)	IU/L	strain	1962,0487	13734,3406	0,021496503	0,03571598
56530	GMC16	AST_17	aspartate transaminase (plasma AST)	IU/L	strain	2711,9531	10847,8123	0,001150111	0,002407341
56531	GMC16	AST_21	aspartate transaminase (plasma AST)	IU/L	strain	19806,2053	138643,4369	3,65E-06	9,89E-06
56533	GMC16	LDH_21	lactic acid dehydrogenase (plasma LDH)	IU/L	strain	22672,9368	158710,5579	0,007087513	0,01310116
56534	GMC16	amylase_17	alpha-amylase (plasma)	IU/L	strain	99355,3564	397421,4255	1,04E-06	2,89E-06
56535	GMC16	amylase_21	alpha-amylase (plasma)	IU/L	strain	619587,8773	4337115,141	4,42E-45	3,07E-44
56537	GMC16	GLU_21	glucose (plasma GLU)	mmol/L	strain	274,2673	1919,8708	4,37E-06	1,17E-05
56538	GMC16	albumin_17	albumin (plasma Alb)	g/L	strain	25,0491	100,1965	0,000855371	0,001826218
56539	GMC16	albumin_21	albumin (plasma Alb)	g/L	strain	176,9878	1238,9143	6,21E-29	3,34E-28
56540	GMC16	creatinine_17	creatinine (plasma)	µmol/L	strain	23,7869	95,1474	0,000163361	0,00038327
56542	GMC16	total_protein_17	total protein (plasma TP)	g/L	strain	23,9566	95,8265	0,002707826	0,00535297
56543	GMC16	total_protein_21	total protein (plasma TP)	g/L	strain	273,999	1917,9931	3,07E-30	1,69E-29
56544	GMC16	urea_17	blood urea nitrogen (plasma BUN)	mmol/L	strain	11,8332	47,3327	0,000789656	0,001707257
56545	GMC16	urea_21	blood urea nitrogen (plasma BUN)	mmol/L	strain	98,0107	686,0751	1,92E-29	1,05E-28
56546	GMC16	CHOL_17	total cholesterol (plasma CHOL)	mmol/L	strain	11,7623	47,0491	1,48E-06	4,09E-06
56547	GMC16	CHOL_21	total cholesterol (plasma CHOL)	mmol/L	strain	47,7013	333,9088	5,37E-78	7,16E-77
56548	GMC16	TG_17	triglyceride (plasma TG)	mmol/L	strain	8,7387	34,9547	5,68E-06	1,52E-05
56549	GMC16	TG_21	triglyceride (plasma TG)	mmol/L	strain	13,6189	95,3321	3,59E-21	1,62E-20
56601	GMC17	bw	body weight	g	strain	4115,2615	24691,5691	8,52E-102	2,27E-100
56611	GMC17	click	auditory brainstem response (ABR) threshold, sound	dB	strain	21052,1216	126312,7299	1,92E-51	1,50E-50
56612	GMC17	pip_6	auditory brainstem response (ABR) threshold, sound	dB	strain	11665,3816	69992,2894	3,14E-37	1,89E-36
56613	GMC17	pip_12	auditory brainstem response (ABR) threshold, sound	dB	strain	29662,573	177975,4379	9,25E-51	7,12E-50
56614	GMC17	pip_18	auditory brainstem response (ABR) threshold, sound	dB	strain	27091,7735	162550,6407	6,24E-53	5,03E-52
56615	GMC17	pip_24	auditory brainstem response (ABR) threshold, sound	dB	strain	17504,7244	105028,3467	9,22E-38	5,63E-37
56616	GMC17	pip_30	auditory brainstem response (ABR) threshold, sound	dB	strain	9464,2429	56785,4575	3,03E-27	1,59E-26
56701	GMC18	bw	body weight	g	strain	4215,2567	25291,5401	3,00E-103	8,26E-102
56702	GMC18	body_length	body length	cm	strain	10,9435	43,7741	3,54E-62	3,78E-61
56711	GMC18	body_size	body size (1=small, 2=normal, 3=big)	designation	strain	7,8451	47,0703	7,28E-46	5,18E-45
56712	GMC18	body_type	body type (1=emaciated, 2=slender, 3=normal, 4=puc	designation	strain	19,697	118,1822	3,22E-56	2,84E-55
56721	GMC18	FATmass_Xhead	fat tissue mass, without head	g	strain	5263,5359	21054,1437	9,26E-72	1,16E-70
56722	GMC18	LEANmass_Xhead	lean tissue mass, without head	g	strain	215,125	860,5001	7,01E-25	3,52E-24
56723	GMC18	SOFTmass_Xhead	total soft tissue mass, without head	g	strain	4387,3819	17549,5276	6,47E-87	1,20E-85
56724	GMC18	BMD_Xhead	bone mineral density (BMD), without head	g/cm²	strain	0,0017	0,0066	1,32E-31	7,45E-31
56725	GMC18	bone_area_Xhead	bone area, without head	cm²	strain	933,7259	3734,9036	2,61E-58	2,56E-57
56726	GMC18	bone_mass_Xhead	bone mass, without head	g	strain	5,0552	20,2208	2,96E-68	3,56E-67
56731	GMC18	BMD_wholebody	bone mineral density (BMD), whole body including he	g/cm²	strain	0,0018	0,007	1,23E-25	6,24E-25
56732	GMC18	FATmass_wholebody	fat tissue mass, whole body including head	g	strain	6068,6065	24274,4261	7,08E-72	9,02E-71
56733	GMC18	LEANmass_wholebody	lean tissue mass, whole body including head	g	strain	327,706	1310,8241	5,04E-29	2,72E-28
56734	GMC18	SOFTmass_wholebody	total soft tissue mass, whole body including head	g	strain	4726,8171	18907,2684	6,51E-86	1,16E-84
56735	GMC18	bone_area_wholebody	bone area, whole body including head	cm²	strain	1054,2805	4217,1219	5,19E-58	4,81E-57
56736	GMC18	bone_mass_wholebody	bone mass, whole body including head	g	strain	7,3012	29,2048	2,39E-70	2,96E-69
56741	GMC18	lumbar_num	number of lumbar vertebrae	n	strain	0,8149	4,8891	3,87E-11	1,40E-10
56801	GMC19	bw	body weight	g	strain	673,6652	4715,6562	4,78E-16	1,94E-15
56813	GMC19	forced_expiratory_vol	forced expiratory volume expired in first 100 ms of fa	mL	strain	0,1623	0,6491	2,17E-05	5,56E-05
56821	GMC19	lung_capacity	total lung capacity (TLC), volume in the lungs at maxir	mL	strain	0,154	0,7698	5,74E-06	1,53E-05
56822	GMC19	vital_capacity	vital capacity (VC), volume of air breathed out after d	mL	strain	0,1702	0,8511	2,62E-05	6,64E-05
56823	GMC19	forced_capacity	forced vital capacity (FVC), volume expired during fas	mL	strain	0,1679	0,6717	6,33E-05	0,000153919
56824	GMC19	inspiratory_capacity	inspiratory capacity (IC), volume inspired during slow	mL	strain	0,111	0,4441	0,000426433	0,000955837
56831	GMC19	chord_compliance	lung compliance (chord compliance) (H₂	mL/cm	strain	7,00E-04	0,0036	0,000222739	0,000511341
56832	GMC19	dynamic_compliance	dynamic compliance (C) of respiratory system with br	mL/cm	strain	1,00E-04	8,00E-04	1,64E-05	4,26E-05
56841	GMC19	resistance	respiratory system resistance (H₂O pres:	cm/mL/s	strain	0,2291	1,6037	0,001552103	0,003186288
56842	GMC19	peak_flow	peak expiratory flow (PEF)	mL/s	strain	38,1327	152,5307	0,000853758	0,001826218
56902	GMC20	IgM_17	immunoglobulin M (plasma IgM)	µg/mL	strain	736877,2387	2947508,955	1,09E-06	3,02E-06
56903	GMC20	IgM_21	immunoglobulin M (plasma IgM)	µg/mL	strain	938117,2765	6566820,935	1,65E-12	6,28E-12
56904	GMC20	IgA_17	immunoglobulin A (plasma IgA)	µg/mL	strain	894501,5894	3578006,358	1,30E-05	3,40E-05
56908	GMC20	IgG2a_17	immunoglobulin G2a (plasma IgG2a)	µg/mL	strain	181533,3944	726133,5777	0,002960654	0,00581241
56909	GMC20	IgG2a_21	immunoglobulin G2a (plasma IgG2a)	µg/mL	strain	657937,5998	4605563,199	6,56E-07	1,86E-06
56910	GMC20	IgG2b_17	immunoglobulin G2b (plasma IgG2b)	µg/mL	strain	17699,5568	53098,6704	0,000998942	0,002111624
56911	GMC20	IgG2b_21	immunoglobulin G2b (plasma IgG2b)	µg/mL	strain	228075,0909	912300,3635	1,94E-12	7,32E-12
56912	GMC20	IgG3_17	immunoglobulin G3 (plasma IgG3)	µg/mL	strain	80326,8812	240980,6435	0,000622119	0,001379973
56914	GMC20	IgE_17	immunoglobulin E (plasma IgE)	ng/mL	strain	1671902,522	6687610,088	0,014713648	0,025748884
56915	GMC20	IgE_21	immunoglobulin E (plasma IgE)	ng/mL	strain	9835432,493	68848027,45	7,89E-18	3,32E-17
56916	GMC20	aDNA_17	anti-DNA autoantibodies (plasma)		strain	0,072	0,2882	8,37E-19	3,57E-18
56917	GMC20	aDNA_21	anti-DNA autoantibodies (plasma)		strain	1,7255	12,0783	1,33E-33	7,76E-33
56918	GMC20	RF_17	rheumatoid factor (plasma RF)		strain	0,0155	0,062	2,92E-08	8,91E-08
56919	GMC20	RF_21	rheumatoid factor (plasma RF)		strain	0,3125	2,1877	2,69E-18	1,14E-17
57001	GMC21	bw	body weight	g	strain	3787,8533	26514,9728	2,51E-90	5,49E-89
57021	GMC21	heart_wt	heart weight	mg	strain	29931,2511	209518,7575	3,00E-47	2,17E-46
57022	GMC21	liver_wt	liver weight	g	strain	9,416	65,912	1,24E-85	2,13E-84
57023	GMC21	spleen_wt	spleen weight	g	strain	0,0366	0,256	8,58E-63	9,28E-62
