# Supplementary material for: A comprehensive and comparative phenotypic analysis of the collaborative founder strains identifies new and known phenotypes
Source: Mamm Genome. 2020 Feb 14;31(1):30–48. doi: 10.1007/s00335-020-09827-3 (PMC7060152; doi:10.1007/s00335-020-09827-3)
Supplement: Supplementary file 7 — Supplementary file7 (PDF 105 kb) [file 335_2020_9827_MOESM7_ESM.pdf]

# Table S2

| measnum | projsym | varname | descrip | units | aspect | mean_sq | sum_sq | precise_pvalue_prf | adj.p.value.BH |
|---|---|---|---|---|---|---|---|---|---|
| 55001 | GMC01 | bw | body weight | g | sex | 1430,6797 | 1430,6797 | 8,54E-54 | 7,29E-53 |
| 55011 | GMC01 | distance_1 | distance traveled, successive 5 min intervals | cm | sex | 11070264,84 | 11070264,84 | 0,017268964 | 0,029733256 |
| 55012 | GMC01 | distance_2 | distance traveled, successive 5 min intervals | cm | sex | 10187682,41 | 10187682,41 | 0,018867798 | 0,03184407 |
| 55014 | GMC01 | distance_4 | distance traveled, successive 5 min intervals | cm | sex | 9302718,068 | 9302718,068 | 0,005921193 | 0,011162691 |
| 55015 | GMC01 | distance_total | distance traveled total, 20 min test | cm | sex | 128386088,7 | 128386088,7 | 0,010241811 | 0,018375013 |
| 55032 | GMC01 | whole_speed | average speed in whole arena, 20 min test | cm/s | sex | 142,56 | 142,56 | 0,002527463 | 0,005043114 |
| 55041 | GMC01 | center_distance | distance traveled in center of arena, 20 min test | cm | sex | 59353677,98 | 59353677,98 | 1,01E-06 | 2,80E-06 |
| 55043 | GMC01 | center_permanence | permanence time in center of arena, 20 min test | s | sex | 14736,8909 | 14736,8909 | 0,017774422 | 0,030298117 |
| 55046 | GMC01 | center_entries | number of entries in center of arena, 20 min test | n | sex | 60746,8225 | 60746,8225 | 0,0001043 | 0,000250204 |
| 55051 | GMC01 | center_distance_1 | center distance, successive 5 min intervals | % | sex | 521,4614 | 521,4614 | 1,22E-05 | 3,20E-05 |
| 55052 | GMC01 | center_distance_2 | center distance, successive 5 min intervals | % | sex | 216,1855 | 216,1855 | 0,013523007 | 0,023811645 |
| 55054 | GMC01 | center_distance_4 | center distance, successive 5 min intervals | % | sex | 464,2466 | 464,2466 | 0,001374733 | 0,002849569 |
| 55055 | GMC01 | center_distance_total | percentage of total distance in center, 20 min test | % | sex | 278,4916 | 278,4916 | 0,000296069 | 0,000670672 |
| 55061 | GMC01 | center_time_1 | center time, successive 5 min intervals | % | sex | 337,8764 | 337,8764 | 2,92E-05 | 7,38E-05 |
| 55065 | GMC01 | center_time_total | percentage of total time spent in center, 20 min test | % | sex | 102,2582 | 102,2582 | 0,017840914 | 0,030350877 |
| 55073 | GMC01 | periphery_permanence | permanence time in periphery, 20 min test | s | sex | 14738,2754 | 14738,2754 | 0,01776947 | 0,030298117 |
| 55074 | GMC01 | periphery_speed | average speed in periphery, 20 min test | cm/s | sex | 86,4927 | 86,4927 | 0,012793689 | 0,022714782 |
| 55101 | GMC02 | bw | body weight | g | sex | 1359,0134 | 1359,0134 | 4,01E-58 | 3,80E-57 |
| 55111 | GMC02 | front_paws1 | forelimb grip strength | g | sex | 8606,9004 | 8606,9004 | 1,95E-08 | 6,00E-08 |
| 55112 | GMC02 | front_paws2 | forelimb grip strength | g | sex | 9693,9945 | 9693,9945 | 5,76E-10 | 1,98E-09 |
| 55113 | GMC02 | front_paws3 | forelimb grip strength | g | sex | 11139,1204 | 11139,1204 | 1,83E-10 | 6,47E-10 |
| 55114 | GMC02 | front_paws_mean | forelimb grip strength, mean | g | sex | 9825,4147 | 9825,4147 | 1,47E-11 | 5,51E-11 |
| 55115 | GMC02 | front_paws_adj | forelimb grip strength (mean) normalized to body w | ratio | sex | 18,2353 | 18,2353 | 7,40E-08 | 2,21E-07 |
| 55121 | GMC02 | all_paws1 | forelimb and hindlimb grip strength | g | sex | 25603,4017 | 25603,4017 | 1,52E-10 | 5,42E-10 |
| 55122 | GMC02 | all_paws2 | forelimb and hindlimb grip strength | g | sex | 25707,664 | 25707,664 | 7,72E-11 | 2,77E-10 |
| 55123 | GMC02 | all_paws3 | forelimb and hindlimb grip strength | g | sex | 23154,732 | 23154,732 | 1,60E-09 | 5,31E-09 |
| 55124 | GMC02 | all_paws_mean | forelimb and hindlimb grip strength, mean | g | sex | 24815,0517 | 24815,0517 | 1,38E-11 | 5,20E-11 |
| 55125 | GMC02 | all_paws_adj | forelimb and hindlimb grip strength (mean) normaliz | ratio | sex | 80,93 | 80,93 | 2,04E-11 | 7,54E-11 |
| 55201 | GMC03 | bw | body weight | g | sex | 1359,0134 | 1359,0134 | 4,01E-58 | 3,80E-57 |
| 55211 | GMC03 | coat_app | coat appearance (1=tidy, 2=irregular) | score | sex | 0,743 | 0,743 | 0,000210401 | 0,000485628 |
| 55214 | GMC03 | pelvic_elev | pelvic elevation (0=less than 5, 1=5 or more, 2=no d | score | sex | 0,3968 | 0,3968 | 0,019981305 | 0,033590618 |
| 55222 | GMC03 | urinate | urination (0=present, 1=absent) | score | sex | 1,1478 | 1,1478 | 0,017665989 | 0,030233978 |
| 55301 | GMC04 | bw | body weight | g | sex | 1459,5067 | 1459,5067 | 3,50E-60 | 3,56E-59 |
| 55313 | GMC04 | LatFall_3 | latency to fall from accelerating rotarod (8rpm/min) | s | sex | 48850,8333 | 48850,8333 | 0,006639853 | 0,012380862 |
| 55401 | GMC05 | bw | body weight | g | sex | 1308,5151 | 1308,5151 | 2,36E-47 | 1,72E-46 |
| 55413 | GMC05 | ASR_80 | acoustic startle response (ASR) | amplitude | sex | 167753,5938 | 167753,5938 | 0,000414945 | 0,000932534 |
| 55414 | GMC05 | ASR_85 | acoustic startle response (ASR) | amplitude | sex | 285788,1175 | 285788,1175 | 0,000338308 | 0,000764326 |
| 55415 | GMC05 | ASR_90 | acoustic startle response (ASR) | amplitude | sex | 681730,8564 | 681730,8564 | 3,05E-05 | 7,65E-05 |
| 55416 | GMC05 | ASR_100 | acoustic startle response (ASR) | amplitude | sex | 2449395,913 | 2449395,913 | 7,92E-11 | 2,83E-10 |
| 55417 | GMC05 | ASR_110 | acoustic startle response (ASR) | amplitude | sex | 2831425,016 | 2831425,016 | 6,55E-09 | 2,06E-08 |
| 55418 | GMC05 | ASR_120 | acoustic startle response (ASR) | amplitude | sex | 2780450,346 | 2780450,346 | 4,36E-10 | 1,51E-09 |
| 55421 | GMC05 | ASR_PP_67 | acoustic startle response (ASR), 110 db sound pressu | amplitude | sex | 1203366,608 | 1203366,608 | 0,000777041 | 0,001688532 |
| 55422 | GMC05 | ASR_PP_69 | acoustic startle response (ASR), 110 db sound pressu | amplitude | sex | 942198,5579 | 942198,5579 | 0,002217497 | 0,004476932 |
| 55424 | GMC05 | ASR_PP_81 | acoustic startle response (ASR), 110 db sound pressu | amplitude | sex | 395080,3094 | 395080,3094 | 0,011681732 | 0,020827138 |
| 55441 | GMC05 | ASR_ISI_5 | acoustic startle response (ASR) | amplitude | sex | 780801,0222 | 780801,0222 | 0,003700114 | 0,007149088 |
| 55442 | GMC05 | ASR_ISI_25 | acoustic startle response (ASR) | amplitude | sex | 590501,18 | 590501,18 | 0,006850738 | 0,012690955 |
| 55443 | GMC05 | ASR_ISI_100 | acoustic startle response (ASR) | amplitude | sex | 1947555,003 | 1947555,003 | 4,51E-07 | 1,29E-06 |
| 55501 | GMC06 | bw_before_fast | body weight | g | sex | 1504,4385 | 1504,4385 | 6,64E-45 | 4,57E-44 |
| 55502 | GMC06 | bw_after_fast | body weight | g | sex | 1108,1075 | 1108,1075 | 1,64E-33 | 9,45E-33 |
| 55521 | GMC06 | CHOL | total cholesterol (plasma CHOL, 16h fast) | mmol/L | sex | 21,101 | 21,101 | 6,02E-20 | 2,62E-19 |
| 55522 | GMC06 | HDL | HDL cholesterol (plasma HDL, 16h fast) | mmol/L | sex | 7,9561 | 7,9561 | 5,94E-23 | 2,87E-22 |
| 55523 | GMC06 | nonHDL | non-HDL cholesterol (plasma non-HDL) (CHOL minus | mmol/L | sex | 3,1421 | 3,1421 | 1,65E-08 | 5,09E-08 |
| 55525 | GMC06 | TG | triglyceride (plasma TG, 16h fast) | mmol/L | sex | 15,1643 | 15,1643 | 4,38E-09 | 1,40E-08 |
| 55601 | GMC07 | bw | body weight | g | sex | 1713,9662 | 1713,9662 | 3,68E-55 | 3,20E-54 |
| 55701 | GMC08 | TEWL_adj | transepidermal water loss, normalized | g/m<sup>2</sup>/h | sex | 1517,132 | 1517,132 | 5,40E-09 | 1,71E-08 |
| 55702 | GMC08 | TEWL_CV | coefficient of variation of transepidermal water loss | % | sex | 0,2441 | 0,2441 | 0,020874489 | 0,035023209 |
| 55801 | GMC09 | bw_before | body weight before testing | g | sex | 1801,8796 | 1801,8796 | 9,07E-58 | 8,33E-57 |
| 55802 | GMC09 | bw_after | body weight after testing | g | sex | 1570,5699 | 1570,5699 | 1,15E-53 | 9,63E-53 |
| 55811 | GMC09 | food | total food intake, 21 h test | g | sex | 4,6511 | 4,6511 | 0,004475887 | 0,008551248 |
| 55831 | GMC09 | VO2_mean | mean oxygen consumption, 21 h test, 15 min bins | mL/h | sex | 2176,1962 | 2176,1962 | 3,31E-09 | 1,07E-08 |
| 55832 | GMC09 | VCO2_mean | mean carbon dioxide production, 21 h test, 15 min b | mL/h | sex | 1936,0486 | 1936,0486 | 3,34E-09 | 1,07E-08 |
| 55841 | GMC09 | heat_mean | mean heat production, 21 h test, 15 min bins | kJ/h | sex | 0,9622 | 0,9622 | 1,05E-09 | 3,54E-09 |
| 55854 | GMC09 | breaks_YA_mean | mean ambulatory movement on Y-axis, 21 h test, 15 | n | sex | 3653637,389 | 3653637,389 | 0,001210807 | 0,002528189 |
| 55855 | GMC09 | breaks_YF_mean | mean fine movement on Y-axis, 21 h test, 15 min bir | n | sex | 186226,535 | 186226,535 | 1,69E-05 | 4,36E-05 |
| 55856 | GMC09 | breaks_Z_mean | mean rearing movement on Z-axis, 21 h test, 15 min | n | sex | 51728,9061 | 51728,9061 | 0,008198655 | 0,015025003 |
| 55857 | GMC09 | distance_mean | mean distance traveled, 21 h test, 15 min bins | cm | sex | 63113483,86 | 63113483,86 | 0,030410652 | 0,049847787 |
| 55901 | GMC10 | bw_13wk | body weight | g | sex | 1703,4363 | 1703,4363 | 1,67E-61 | 1,76E-60 |
| 55902 | GMC10 | bw_19wk | body weight | g | sex | 1482,2183 | 1482,2183 | 1,65E-30 | 9,13E-30 |
| 55911 | GMC10 | fat_13wk | fat tissue mass, whole body with head (NMR) | g | sex | 7,7576 | 7,7576 | 0,004606553 | 0,008781241 |
| 55921 | GMC10 | lean_13wk | lean tissue mass, whole body with head (NMR) | g | sex | 530,802 | 530,802 | 8,13E-64 | 8,90E-63 |
| 55922 | GMC10 | lean_19wk | lean tissue mass, whole body with head (NMR) | g | sex | 580,5071 | 580,5071 | 1,25E-42 | 8,31E-42 |
| 56001 | GMC11 | bw_before_fast | body weight | g | sex | 1493,6175 | 1493,6175 | 4,48E-52 | 3,54E-51 |
| 56002 | GMC11 | bw_after_fast | body weight | g | sex | 606,4132 | 606,4132 | 5,05E-22 | 2,34E-21 |
| 56012 | GMC11 | GLU_15 | intraperitoneal glucose tolerance test (20% glucose i | mmol/L | sex | 123,8224 | 123,8224 | 0,00270503 | 0,00535297 |
| 56013 | GMC11 | GLU_30 | intraperitoneal glucose tolerance test (20% glucose i | mmol/L | sex | 244,5688 | 244,5688 | 8,15E-05 | 0,000197229 |
| 56014 | GMC11 | GLU_60 | intraperitoneal glucose tolerance test (20% glucose i | mmol/L | sex | 147,4402 | 147,4402 | 0,002003305 | 0,004063711 |
| 56015 | GMC11 | GLU_120 | intraperitoneal glucose tolerance test (20% glucose i | mmol/L | sex | 98,762 | 98,762 | 0,002612468 | 0,005200577 |
| 56101 | GMC12 | bw | body weight | g | sex | 1361,7984 | 1361,7984 | 8,38E-45 | 5,72E-44 |
| 56121 | GMC12 | LV_mass | left ventricular mass corrected | mg | sex | 537,068 | 537,068 | 0,000653818 | 0,00144653 |
| 56124 | GMC12 | stroke_vol | stroke volume, volume of blood pumped from one v | µL | sex | 1105,6296 | 1105,6296 | 1,11E-05 | 2,91E-05 |
| 56125 | GMC12 | cardiac_output | cardiac output, volume of blood pumped by the hea | mL/min | sex | 601,6338 | 601,6338 | 2,69E-11 | 9,83E-11 |
| 56133 | GMC12 | LVID_diastole | left ventricular internal dimension | mm | sex | 2,4599 | 2,4599 | 0,000128014 | 0,000306229 |
| 56301 | GMC14 | bw | body weight | g | sex | 1829,824 | 1829,824 | 6,39E-46 | 4,58E-45 |
| 56302 | GMC14 | body_length | body length | mm | sex | 985,723 | 985,723 | 2,89E-15 | 1,16E-14 |
| 56321 | GMC14 | eye_length_L | eye axial length | mm | sex | 0,1682 | 0,1682 | 2,66E-08 | 8,13E-08 |
| 56322 | GMC14 | eye_length_R | eye axial length | mm | sex | 0,0413 | 0,0413 | 6,66E-05 | 0,000161657 |
| 56401 | GMC15 | WBC | white blood cell count (WBC; per volume x 10<sup> | n/µL | sex | 44,817 | 44,817 | 0,001079811 | 0,002265746 |
| 56404 | GMC15 | RBC | red blood cell count (RBC; per volume x 10<sup>6</ | n/µL | sex | 10,7854 | 10,7854 | 0,001441421 | 0,002980566 |
| 56408 | GMC15 | MCV | mean RBC corpuscular volume (MCV) | fL | sex | 44,3169 | 44,3169 | 0,00313221 | 0,006122571 |
| 56412 | GMC15 | MCH | calculated mean RBC corpuscular hemoglobin conte | pg | sex | 7,1457 | 7,1457 | 3,57E-05 | 8,91E-05 |
| 56431 | GMC15 | PLT | platelet count (PLT; units per volume x 10<sup>3</s | n/µL | sex | 913472,7423 | 913472,7423 | 0,000246321 | 0,000563963 |
| 56441 | GMC15 | PCT | plateletcrit (PCT) | % | sex | 0,3509 | 0,3509 | 0,000253973 | 0,000579928 |

| | | | | | | | | | |
|---|---|---|---|---|---|---|---|---|---|
| 56501 | GMC16 | bw_17 | body weight | g | sex | 248,004 | 248,004 | 1,57E-07 | 4,65E-07 |
| 56502 | GMC16 | bw_21 | body weight | g | sex | 874,9392 | 874,9392 | 5,92E-13 | 2,27E-12 |
| 56513 | GMC16 | chloride_21 | chloride (plasma Cl) | mmol/L | sex | 71,5161 | 71,5161 | 0,002414171 | 0,004839676 |
| 56515 | GMC16 | iron_21 | iron (plasma Fe) | µmol/L | sex | 178,0431 | 178,0431 | 0,002809961 | 0,005529278 |
| 56519 | GMC16 | lactate_21 | lactate (plasma) | mmol/L | sex | 61,5057 | 61,5057 | 0,000802448 | 0,001726174 |
| 56522 | GMC16 | potassium_17 | potassium (plasma K) | mmol/L | sex | 1,1463 | 1,1463 | 0,0179027 | 0,030395439 |
| 56523 | GMC16 | potassium_21 | potassium (plasma K) | mmol/L | sex | 1,6669 | 1,6669 | 0,001868814 | 0,003808991 |
| 56524 | GMC16 | sodium_17 | sodium (plasma Na) | mmol/L | sex | 125,2895 | 125,2895 | 9,79E-05 | 0,0002362 |
| 56525 | GMC16 | sodium_21 | sodium (plasma Na) | mmol/L | sex | 177,4938 | 177,4938 | 5,36E-05 | 0,000131422 |
| 56526 | GMC16 | ALP_17 | alkaline phosphatase (plasma ALP) | IU/L | sex | 29484,9 | 29484,9 | 1,29E-09 | 4,32E-09 |
| 56527 | GMC16 | ALP_21 | alkaline phosphatase (plasma ALP) | IU/L | sex | 19323,9573 | 19323,9573 | 0,000798805 | 0,001722675 |
| 56531 | GMC16 | AST_21 | aspartate transaminase (plasma AST) | IU/L | sex | 49809,7037 | 49809,7037 | 0,000147037 | 0,000346877 |
| 56534 | GMC16 | amylase_17 | alpha-amylase (plasma) | IU/L | sex | 44822,3555 | 44822,3555 | 0,015739867 | 0,027488439 |
| 56535 | GMC16 | amylase_21 | alpha-amylase (plasma) | IU/L | sex | 284131,3036 | 284131,3036 | 7,20E-08 | 2,16E-07 |
| 56542 | GMC16 | total_protein_17 | total protein (plasma TP) | g/L | sex | 44,8855 | 44,8855 | 0,003981705 | 0,007641294 |
| 56543 | GMC16 | total_protein_21 | total protein (plasma TP) | g/L | sex | 52,9002 | 52,9002 | 0,008278988 | 0,015107384 |
| 56545 | GMC16 | urea_21 | blood urea nitrogen (plasma BUN) | mmol/L | sex | 31,7373 | 31,7373 | 0,000877812 | 0,001869454 |
| 56547 | GMC16 | CHOL_21 | total cholesterol (plasma CHOL) | mmol/L | sex | 10,7866 | 10,7866 | 5,35E-11 | 1,93E-10 |
| 56548 | GMC16 | TG_17 | triglyceride (plasma TG) | mmol/L | sex | 5,1429 | 5,1429 | 0,011674451 | 0,020827138 |
| 56549 | GMC16 | TG_21 | triglyceride (plasma TG) | mmol/L | sex | 23,5633 | 23,5633 | 3,12E-09 | 1,01E-08 |
| 56601 | GMC17 | bw | body weight | g | sex | 1232,1548 | 1232,1548 | 1,35E-23 | 6,65E-23 |
| 56701 | GMC18 | bw | body weight | g | sex | 1389,5884 | 1389,5884 | 1,25E-25 | 6,34E-25 |
| 56702 | GMC18 | body_length | body length | cm | sex | 3,1327 | 3,1327 | 4,09E-13 | 1,57E-12 |
| 56711 | GMC18 | body_size | body size (1=small, 2=normal, 3=big) | designation | sex | 3,7048 | 3,7048 | 7,85E-09 | 2,45E-08 |
| 56722 | GMC18 | LEANmass_Xhead | lean tissue mass, without head | g | sex | 650,5533 | 650,5533 | 7,46E-23 | 3,58E-22 |
| 56723 | GMC18 | SOFTmass_Xhead | total soft tissue mass, without head | g | sex | 1018,1182 | 1018,1182 | 9,96E-21 | 4,43E-20 |
| 56725 | GMC18 | bone_area_Xhead | bone area, without head | cm<sup>2</sup> | sex | 84,3412 | 84,3412 | 5,27E-05 | 0,000129598 |
| 56726 | GMC18 | bone_mass_Xhead | bone mass, without head | g | sex | 0,3556 | 0,3556 | 2,20E-05 | 5,63E-05 |
| 56733 | GMC18 | LEANmass_wholebody | lean tissue mass, whole body including head | g | sex | 761,739 | 761,739 | 3,18E-22 | 1,48E-21 |
| 56734 | GMC18 | SOFTmass_wholebody | total soft tissue mass, whole body including head | g | sex | 1225,5008 | 1225,5008 | 6,85E-22 | 3,16E-21 |
| 56735 | GMC18 | bone_area_wholebody | bone area, whole body including head | cm<sup>2</sup> | sex | 94,5675 | 94,5675 | 6,09E-05 | 0,000148543 |
| 56736 | GMC18 | bone_mass_wholebody | bone mass, whole body including head | g | sex | 0,442 | 0,442 | 4,31E-05 | 0,000106774 |
| 56912 | GMC20 | IgG3_17 | immunoglobulin G3 (plasma IgG3) | µg/mL | sex | 76063,6506 | 76063,6506 | 0,010219138 | 0,018372934 |
| 56914 | GMC20 | IgE_17 | immunoglobulin E (plasma IgE) | ng/mL | sex | 3582142,201 | 3582142,201 | 0,008601515 | 0,015629136 |
| 56915 | GMC20 | IgE_21 | immunoglobulin E (plasma IgE) | ng/mL | sex | 4648481,62 | 4648481,62 | 0,001944739 | 0,003954304 |
| 56916 | GMC20 | aDNA_17 | anti-DNA autoantibodies (plasma) | | sex | 0,0099 | 0,0099 | 0,000149156 | 0,000350908 |
| 56918 | GMC20 | RF_17 | rheumatoid factor (plasma RF) | | sex | 0,0065 | 0,0065 | 0,006144586 | 0,011532916 |
| 57001 | GMC21 | bw | body weight | g | sex | 980,8556 | 980,8556 | 9,93E-16 | 4,02E-15 |
| 57021 | GMC21 | heart_wt | heart weight | mg | sex | 37727,1327 | 37727,1327 | 1,10E-17 | 4,61E-17 |
| 57022 | GMC21 | liver_wt | liver weight | g | sex | 4,5752 | 4,5752 | 2,22E-22 | 1,04E-21 |
