# Supplementary material for: A comprehensive and comparative phenotypic analysis of the collaborative founder strains identifies new and known phenotypes
Source: Mamm Genome. 2020 Feb 14;31(1):30–48. doi: 10.1007/s00335-020-09827-3 (PMC7060152; doi:10.1007/s00335-020-09827-3)
Supplement: Supplementary file 8 — Supplementary file8 (PDF 107 kb) [file 335_2020_9827_MOESM8_ESM.pdf]

Table S3

measnum	projsym	varname	descrip	units	aspect	mean_sq	sum_sq	precise_pvalue_prf	adj.p.value.BH
55001	GMC01	bw	body weight	g	sex:strain	25,2635	176,8444	1,44E-07	4,27E-07
55014	GMC01	distance_4	distance traveled, successive 5 min intervals	cm	sex:strain	3002539,351	21017775,45	0,017390408	0,02988211
55023	GMC01	rears_3	number of rears, successive 5 min intervals	n	sex:strain	282,9085	1980,3596	0,002337388	0,004707852
55031	GMC01	whole_rest	resting time in whole arena, 20 min test	s	sex:strain	3395,7297	23770,1077	0,016406599	0,028420357
55041	GMC01	center_distance	distance traveled in center of arena, 20 min test	cm	sex:strain	14779705,03	103457935,2	8,78E-07	2,46E-06
55043	GMC01	center_permanence	permanence time in center of arena, 20 min test	s	sex:strain	9684,0471	67788,3298	0,000706551	0,001539271
55046	GMC01	center_entries	number of entries in center of arena, 20 min test	n	sex:strain	16412,1591	114885,1137	0,000211458	0,000486751
55051	GMC01	center_distance_1	center distance, successive 5 min intervals	%	sex:strain	110,8612	776,0284	0,00019447	0,000450074
55055	GMC01	center_distance_total	percentage of total distance in center, 20 min test	%	sex:strain	72,4681	507,2764	0,001314222	0,002730768
55061	GMC01	center_time_1	center time, successive 5 min intervals	%	sex:strain	93,8369	656,8586	2,31E-05	5,89E-05
55063	GMC01	center_time_3	center time, successive 5 min intervals	%	sex:strain	126,2559	883,7913	0,025764172	0,042558226
55065	GMC01	center_time_total	percentage of total time spent in center, 20 min test	%	sex:strain	67,2999	471,0995	0,00070396	0,001539164
55072	GMC01	periphery_rest	resting time in periphery, 20 min test	s	sex:strain	3308,6603	23160,6219	0,004955355	0,009425107
55073	GMC01	periphery_permanence	permanence time in periphery, 20 min test	s	sex:strain	9686,6774	67806,7419	0,000704699	0,001539164
55101	GMC02	bw	body weight	g	sex:strain	26,0461	182,3226	2,27E-09	7,39E-09
55122	GMC02	all_paws2	forelimb and hindlimb grip strength	g	sex:strain	1582,0717	11074,5021	0,007261731	0,013365342
55123	GMC02	all_paws3	forelimb and hindlimb grip strength	g	sex:strain	2121,8453	14852,9173	0,001044476	0,002202425
55124	GMC02	all_paws_mean	forelimb and hindlimb grip strength, mean	g	sex:strain	1558,5107	10909,5748	0,003303608	0,006412003
55125	GMC02	all_paws_adj	forelimb and hindlimb grip strength (mean) normalized to body we	ratio	sex:strain	5,3789	37,652	0,002408567	0,004839676
55201	GMC03	bw	body weight	g	sex:strain	26,0461	182,3226	2,27E-09	7,39E-09
55211	GMC03	coat_app	coat appearance (1=tidy, 2=irregular)	score	sex:strain	0,2243	1,5703	0,000175247	0,000407795
55212	GMC03	whiskers	whiskers (0=present, 1=absent)	score	sex:strain	0,0158	0,1105	0,024489057	0,040530338
55216	GMC03	transfer_arousal	transfer arousal (0=prolonged freeze, 1=brief freeze, 2=immediate	score	sex:strain	0,4605	3,2238	0,015986954	0,027749713
55301	GMC04	bw	body weight	g	sex:strain	28,9159	202,4113	2,81E-10	9,78E-10
55401	GMC05	bw	body weight	g	sex:strain	33,5073	234,5511	4,69E-09	1,49E-08
55413	GMC05	ASR_80	acoustic startle response (ASR)	amplitude	sex:strain	30365,7064	212559,9449	0,026263368	0,043299067
55414	GMC05	ASR_85	acoustic startle response (ASR)	amplitude	sex:strain	74759,4482	523316,1374	0,001502313	0,003091506
55415	GMC05	ASR_90	acoustic startle response (ASR)	amplitude	sex:strain	188706,8585	1320948,009	2,60E-05	6,60E-05
55416	GMC05	ASR_100	acoustic startle response (ASR)	amplitude	sex:strain	414162,4252	2899136,976	1,51E-08	4,68E-08
55417	GMC05	ASR_110	acoustic startle response (ASR)	amplitude	sex:strain	472671,7829	3308702,48	1,66E-06	4,55E-06
55418	GMC05	ASR_120	acoustic startle response (ASR)	amplitude	sex:strain	392071,3254	2744499,278	2,01E-06	5,49E-06
55421	GMC05	ASR_PP_67	acoustic startle response (ASR), 110 db sound pressure level	amplitude	sex:strain	321518,297	2250628,079	0,003801628	0,007328646
55431	GMC05	PPI_67	percent prepulse inhibition (PPI), 110 db sound pressure level	%	sex:strain	2732,7878	19129,5145	4,13E-05	0,000102463
55433	GMC05	PPI_73	percent prepulse inhibition (PPI), 110 db sound pressure level	%	sex:strain	1694,9496	11864,6475	0,015797964	0,02750721
55435	GMC05	PPI_global	percentage prepulse inhibition (PPI) evoked by 110 dB sound press	%	sex:strain	1154,8712	8084,0982	0,005747424	0,010883149
55443	GMC05	ASR_ISI_100	acoustic startle response (ASR)	amplitude	sex:strain	200063,1353	1400441,947	0,008872507	0,016019282
55451	GMC05	PPI_ISI_5	prepulse inhibition (PPI)	%	sex:strain	2387,9283	16715,4984	1,80E-06	4,93E-06
55452	GMC05	PPI_ISI_25	prepulse inhibition (PPI)	%	sex:strain	1814,0029	12698,0205	0,003132978	0,006122571
55453	GMC05	PPI_ISI_100	prepulse inhibition (PPI)	%	sex:strain	1144,2038	8009,4265	0,016728577	0,028861019
55501	GMC06	bw_before_fast	body weight	g	sex:strain	21,0586	147,4099	3,05E-05	7,65E-05
55502	GMC06	bw_after_fast	body weight	g	sex:strain	14,9199	59,6797	0,012835008	0,022740866
55521	GMC06	CHOL	total cholesterol (plasma CHOL, 16h fast)	mmol/L	sex:strain	1,7679	7,0716	8,57E-07	2,41E-06
55522	GMC06	HDL	HDL cholesterol (plasma HDL, 16h fast)	mmol/L	sex:strain	0,2858	1,1434	0,00094825	0,002009442
55523	GMC06	nonHDL	non-HDL cholesterol (plasma non-HDL) (CHOL minus HDL, 16h fast)	mmol/L	sex:strain	1,0706	4,2825	1,40E-08	4,35E-08
55601	GMC07	bw	body weight	g	sex:strain	36,3133	254,1931	1,53E-09	5,11E-09
55701	GMC08	TEWL_adj	transepidermal water loss, normalized	g/m²/h	sex:strain	131,1445	918,0114	0,00323634	0,00629575
55801	GMC09	bw_before	body weight before testing	g	sex:strain	34,613	242,2913	2,19E-09	7,15E-09
55802	GMC09	bw_after	body weight after testing	g	sex:strain	29,9361	209,553	3,14E-08	9,54E-08
55831	GMC09	VO2_mean	mean oxygen consumption, 21 h test, 15 min bins	mL/h	sex:strain	220,2746	1541,9221	0,00059959	0,001333463
55832	GMC09	VCO2_mean	mean carbon dioxide production, 21 h test, 15 min bins	mL/h	sex:strain	152,4535	1067,1748	0,005357116	0,010166615
55841	GMC09	heat_mean	mean heat production, 21 h test, 15 min bins	kJ/h	sex:strain	0,0898	0,6284	0,000695885	0,001527726
55851	GMC09	breaks_X_mean	mean total beam breaks on X-axis, 21 h test, 15 min bins	n	sex:strain	2628109,383	18396765,68	0,021392928	0,035682736
55852	GMC09	breaks_XA_mean	mean ambulatory movement on X-axis, 21 h test, 15 min bins	n	sex:strain	2231646,865	15621528,05	0,013519783	0,023811645
55854	GMC09	breaks_YA_mean	mean ambulatory movement on Y-axis, 21 h test, 15 min bins	n	sex:strain	1101131,32	7707919,242	0,002680141	0,005322884
55901	GMC10	bw_13wk	body weight	g	sex:strain	33,1256	231,8794	1,58E-10	5,61E-10
55902	GMC10	bw_19wk	body weight	g	sex:strain	29,6232	207,3621	0,001301889	0,00271174
55912	GMC10	fat_19wk	fat tissue mass, whole body with head (NMR)	g	sex:strain	31,5024	220,5171	5,62E-20	2,46E-19
55921	GMC10	lean_13wk	lean tissue mass, whole body with head (NMR)	g	sex:strain	14,9948	104,9634	2,24E-16	9,26E-16
55922	GMC10	lean_19wk	lean tissue mass, whole body with head (NMR)	g	sex:strain	17,9821	125,875	9,93E-10	3,36E-09
56001	GMC11	bw_before_fast	body weight	g	sex:strain	30,8355	215,8485	5,89E-09	1,86E-08
56011	GMC11	GLU_0	intraperitoneal glucose tolerance test (20% glucose i.p., 16h fast)	mmol/L	sex:strain	14,0567	56,2269	0,000372805	0,00084004
56014	GMC11	GLU_60	intraperitoneal glucose tolerance test (20% glucose i.p., 16h fast)	mmol/L	sex:strain	54,9702	219,8809	0,006798643	0,012621829
56015	GMC11	GLU_120	intraperitoneal glucose tolerance test (20% glucose i.p., 16h fast)	mmol/L	sex:strain	66,3653	265,4612	0,00010302	0,000247828
56101	GMC12	bw	body weight	g	sex:strain	22,1666	155,1665	5,46E-06	1,46E-05
56112	GMC12	heart_rate	heart rate, beats per min	n/min	sex:strain	86871,1526	608098,068	8,76E-08	2,61E-07
56125	GMC12	cardiac_output	cardiac output, volume of blood pumped by the heart per min	mL/min	sex:strain	41,0063	287,0443	0,001814657	0,003707457
56132	GMC12	IVS_systole	interventricular septum	mm	sex:strain	0,0061	0,0429	0,03013881	0,049497199
56202	GMC13	HR_V	heart rate variability, mean of differences between sequential beat	n/min	sex:strain	8316,4281	41582,1403	0,014158338	0,024827969
56203	GMC13	HR_CV	heart rate coefficient of variation (signal intensity)	%	sex:strain	121,6241	608,1204	0,016627183	0,028744159
56242	GMC13	pNN50	proportion of number of pairs of successive beat-to-beat intervals t	%	sex:strain	764,9726	3824,8632	0,006768972	0,012594122
56301	GMC14	bw	body weight	g	sex:strain	22,8029	159,62	0,000135034	0,00032033
56401	GMC15	WBC	white blood cell count (WBC; per volume x 10³)	n/µL	sex:strain	10,042	70,294	0,019004003	0,032010687
56404	GMC15	RBC	red blood cell count (RBC; per volume x 10⁶)	n/µL	sex:strain	3,8876	27,2135	0,000781454	0,001693811
56408	GMC15	MCV	mean RBC corpuscular volume (MCV)	fL	sex:strain	13,9062	97,3436	0,008414959	0,015322762
56412	GMC15	MCH	calculated mean RBC corpuscular hemoglobin content (MCH)	pg	sex:strain	0,9863	6,9042	0,018420551	0,031150793
56414	GMC15	MCHC	calculated mean RBC corpuscular hemoglobin concentration (MCH(	g/dL	sex:strain	3,0437	21,3057	0,003819661	0,007346825
56416	GMC15	HGB	hemoglobin (HGB)	g/dL	sex:strain	8,1712	57,1983	0,002114451	0,004279007
56421	GMC15	HCT	hematocrit (HCT)	%	sex:strain	57,9368	405,5573	0,009411014	0,016955709
56431	GMC15	PLT	platelet count (PLT; units per volume x 10³)	n/µL	sex:strain	201745,3754	1412217,628	0,004233812	0,008106895
56434	GMC15	MPV	mean platelet volume (MPV)	fL	sex:strain	0,2556	1,789	0,008724839	0,015786044
56436	GMC15	PDW	platelet corpuscular distribution width (PDW)	fL	sex:strain	0,8426	5,8982	0,000670895	0,001476661
56441	GMC15	PCT	plateletcrit (PCT)	%	sex:strain	0,0943	0,6603	0,000834204	0,001789976
56501	GMC16	bw_17	body weight	g	sex:strain	42,8709	171,4835	0,000168015	0,000392903
56512	GMC16	chloride_17	chloride (plasma Cl)	mmol/L	sex:strain	14,3746	57,4984	0,005989822	0,011267197
56514	GMC16	iron_17	iron (plasma Fe)	µmol/L	sex:strain	79,7067	318,8267	0,000168387	0,000392903
56515	GMC16	iron_21	iron (plasma Fe)	µmol/L	sex:strain	77,9735	545,8142	0,000429893	0,00096107
56524	GMC16	sodium_17	sodium (plasma Na)	mmol/L	sex:strain	22,3566	89,4264	0,015815035	0,02750721
56526	GMC16	ALP_17	alkaline phosphatase (plasma ALP)	IU/L	sex:strain	3139,775	12559,1	0,000176936	0,000410608
56527	GMC16	ALP_21	alkaline phosphatase (plasma ALP)	IU/L	sex:strain	4915,5568	34408,8973	0,005876427	0,011102806
56529	GMC16	ALT_21	alanine transaminase (plasma ALT)	IU/L	sex:strain	2681,4693	18770,2849	0,002490081	0,004980163
56531	GMC16	AST_21	aspartate transaminase (plasma AST)	IU/L	sex:strain	12114,3606	84800,5243	0,001052426	0,002213725
56532	GMC16	LDH_17	lactic acid dehydrogenase (plasma LDH)	IU/L	sex:strain	35170,7459	140682,9835	0,018264428	0,030948058
56535	GMC16	amylase_21	alpha-amylase (plasma)	IU/L	sex:strain	47270,5056	330893,5393	1,77E-05	4,56E-05
56536	GMC16	GLU_17	glucose (plasma GLU)	mmol/L	sex:strain	307,3325	1229,3301	0,000662431	0,001461798
56537	GMC16	GLU_21	glucose (plasma GLU)	mmol/L	sex:strain	238,142	1666,9937	2,97E-05	7,47E-05
56538	GMC16	albumin_17	albumin (plasma Alb)	g/L	sex:strain	19,7406	78,9625	0,003486771	0,006752161
56539	GMC16	albumin_21	albumin (plasma Alb)	g/L	sex:strain	16,6679	116,6755	0,002761983	0,005447421
56540	GMC16	creatinine_17	creatinine (plasma)	µmol/L	sex:strain	28,9335	115,7339	3,68E-05	9,17E-05
56542	GMC16	total_protein_17	total protein (plasma TP)	g/L	sex:strain	16,3695	65,4781	0,017428519	0,02988746
56543	GMC16	total_protein_21	total protein (plasma TP)	g/L	sex:strain	27,6063	193,2441	0,000912908	0,001939362
56547	GMC16	CHOL_21	total cholesterol (plasma CHOL)	mmol/L	sex:strain	0,9102	6,3713	0,000291821	0,000662807

56548	GMC16	TG_17	triglyceride (plasma TG)	mmol/L	sex:strain	4,1278	16,5113	0,001456264	0,003003983
56549	GMC16	TG_21	triglyceride (plasma TG)	mmol/L	sex:strain	2,5035	17,5244	0,000290372	0,000661273
56601	GMC17	bw	body weight	g	sex:strain	27,9741	167,8444	0,00635308	0,011898092
56701	GMC18	bw	body weight	g	sex:strain	27,3804	164,2824	0,007605415	0,013967794
56711	GMC18	body_size	body size (1=small, 2=normal, 3=big)	designation	sex:strain	0,531	3,1861	4,97E-05	0,000122693
56722	GMC18	LEANmass_Xhead	lean tissue mass, without head	g	sex:strain	15,5338	62,1351	0,013708759	0,024089054
56723	GMC18	SOFTmass_Xhead	total soft tissue mass, without head	g	sex:strain	31,9125	127,65	0,006587537	0,01231019
56733	GMC18	LEANmass_wholebody	lean tissue mass, whole body including head	g	sex:strain	21,3057	85,223	0,007152761	0,013193213
56734	GMC18	SOFTmass_wholebody	total soft tissue mass, whole body including head	g	sex:strain	33,9067	135,627	0,008638868	0,015663679
56908	GMC20	IgG2a_17	immunoglobulin G2a (plasma IgG2a)	µg/mL	sex:strain	119662,9456	478651,7823	0,021354403	0,035682736
56915	GMC20	IgE_21	immunoglobulin E (plasma IgE)	ng/mL	sex:strain	3434624,504	24042371,53	2,65E-07	7,64E-07
56916	GMC20	aDNA_17	anti-DNA autoantibodies (plasma)		sex:strain	0,002	0,008	0,013172225	0,023290021
56917	GMC20	aDNA_21	anti-DNA autoantibodies (plasma)		sex:strain	0,1206	0,8442	0,003169326	0,006179461
56919	GMC20	RF_21	rheumatoid factor (plasma RF)		sex:strain	0,072	0,5038	0,000130262	0,000310737
57001	GMC21	bw	body weight	g	sex:strain	33,5388	234,7718	0,01096472	0,019630756
57022	GMC21	liver_wt	liver weight	g	sex:strain	0,1975	1,3823	9,21E-06	2,43E-05
