# Supplementary material for: A comprehensive and comparative phenotypic analysis of the collaborative founder strains identifies new and known phenotypes
Source: Mamm Genome. 2020 Feb 14;31(1):30–48. doi: 10.1007/s00335-020-09827-3 (PMC7060152; doi:10.1007/s00335-020-09827-3)
Supplement: Supplementary file 10 — Supplementary file10 (PDF 84 kb) [file 335_2020_9827_MOESM10_ESM.pdf]

Table S5

List of strains in each group

LabDerived:A/J, C57BL/6J, 129S1/SvImJ
WTDerived: PWK/PhJ, CAST/EiJ, WSB/EiJ
DiseaseDerived: NZO/HlLtJ, NOD/ShiLtJ

			Group comparision		
protocol	procedure	parameter	LabDerived Vs DiseaseDerived	WTDerived Vs DiseaseDerived	WTDerived Vs LabDerived
GMC01	Open_field_Parameter	distance_1	2,46E-07	3,21E-03	2,32E-02
GMC01	Open_field_Parameter	distance_2	2,52E-05	2,72E-02	5,56E-02
GMC01	Open_field_Parameter	distance_3	4,02E-05	8,36E-03	1,91E-01
GMC01	Open_field_Parameter	distance_4	9,97E-06	1,54E-03	2,73E-01
GMC01	Open_field_Parameter	distance_total	3,62E-06	4,77E-03	8,06E-02
GMC01	Open_field_Parameter	rears_1	8,30E-10	3,36E-01	4,19E-13
GMC01	Open_field_Parameter	rears_2	2,08E-10	5,39E-01	4,21E-13
GMC01	Open_field_Parameter	rears_3	2,37E-07	5,44E-01	4,23E-12
GMC01	Open_field_Parameter	rears_4	2,66E-06	9,93E-01	4,16E-08
GMC01	Open_field_Parameter	rears_total	7,02E-10	5,75E-01	4,22E-13
GMC01	Open_field_Parameter	whole_rest	6,09E-10	3,99E-13	3,92E-13
GMC01	Open_field_Parameter	whole_speed	2,45E-06	4,24E-02	6,46E-03
GMC01	Open_field_Parameter	center_distance	1,13E-09	1,90E-05	5,32E-02
GMC01	Open_field_Parameter	center_rest	2,06E-02	8,21E-01	6,92E-04
GMC01	Open_field_Parameter	center_permanence	3,44E-11	9,72E-02	5,74E-08
GMC01	Open_field_Parameter	center_speed	2,00E-02	8,60E-01	3,47E-02
GMC01	Open_field_Parameter	center_latency	2,24E-08	2,68E-01	4,26E-13
GMC01	Open_field_Parameter	center_entries	1,31E-09	7,03E-06	1,00E-01
GMC01	Open_field_Parameter	center_distance_1	1,90E-08	9,19E-01	2,03E-09
GMC01	Open_field_Parameter	center_distance_2	1,92E-10	2,84E-01	4,21E-13
GMC01	Open_field_Parameter	center_distance_3	5,88E-09	9,09E-01	2,64E-12
GMC01	Open_field_Parameter	center_distance_4	3,91E-06	5,20E-01	1,57E-10
GMC01	Open_field_Parameter	center_distance_total	2,08E-09	6,99E-01	4,57E-13

GMC01	Open_field_Parameter	center_time_1	9,02E-11	2,52E-02	2,95E-06
GMC01	Open_field_Parameter	center_time_2	2,63E-11	7,13E-01	1,23E-11
GMC01	Open_field_Parameter	center_time_3	1,14E-09	3,08E-02	1,94E-05
GMC01	Open_field_Parameter	center_time_4	1,13E-06	3,08E-01	6,67E-05
GMC01	Open_field_Parameter	center_time_total	3,43E-11	9,76E-02	5,66E-08
GMC01	Open_field_Parameter	periphery_distance	4,01E-04	6,77E-02	1,28E-01
GMC01	Open_field_Parameter	periphery_rest	1,21E-10	3,92E-13	3,92E-13
GMC01	Open_field_Parameter	periphery_permanence	3,44E-11	9,72E-02	5,74E-08
GMC01	Open_field_Parameter	periphery_speed	1,11E-06	1,62E-02	1,21E-02
GMC02	Grip_strength_Parameter	front_paws1	7,24E-01	3,96E-13	3,93E-13
GMC02	Grip_strength_Parameter	front_paws2	8,20E-01	4,04E-13	3,92E-13
GMC02	Grip_strength_Parameter	front_paws3	9,08E-01	4,15E-13	3,92E-13
GMC02	Grip_strength_Parameter	front_paws_mean	9,96E-01	3,93E-13	3,92E-13
GMC02	Grip_strength_Parameter	front_paws_adj	4,30E-13	3,93E-13	6,35E-04
GMC02	Grip_strength_Parameter	all_paws1	2,88E-01	3,21E-13	3,21E-13
GMC02	Grip_strength_Parameter	all_paws2	1,36E-01	3,21E-13	3,21E-13
GMC02	Grip_strength_Parameter	all_paws3	1,72E-02	3,21E-13	3,21E-13
GMC02	Grip_strength_Parameter	all_paws_mean	7,96E-02	3,21E-13	3,21E-13
GMC02	Grip_strength_Parameter	all_paws_adj	8,22E-13	3,25E-13	2,32E-04
GMC03	SHIRPA_Parameter	coat_app	1,06E-03	1,92E-07	1,07E-01
GMC03	SHIRPA_Parameter	whiskers	4,88E-13	1,00E+00	2,50E-13
GMC03	SHIRPA_Parameter	body_pos	2,63E-01	5,69E-02	6,22E-05
GMC03	SHIRPA_Parameter	pelvic_elev	6,58E-01	5,28E-06	2,10E-09
GMC03	SHIRPA_Parameter	tail_elev	4,28E-01	7,13E-06	3,44E-04
GMC03	SHIRPA_Parameter	transfer_arousal	9,93E-01	4,64E-08	3,24E-09
GMC03	SHIRPA_Parameter	defecation	1,00E+00	4,30E-02	2,18E-02
GMC03	SHIRPA_Parameter	urinate	3,13E-01	1,51E-02	3,26E-01
GMC03	SHIRPA_Parameter	tremor	2,72E-01	2,51E-01	1,00E+00
GMC03	SHIRPA_Parameter	loco_activity	5,33E-03	4,17E-01	3,48E-06
GMC03	SHIRPA_Parameter	bite_evidence	9,19E-01	6,22E-04	1,98E-05
GMC03	SHIRPA_Parameter	vocalization	8,54E-01	6,31E-02	5,84E-03
GMC03	SHIRPA_Parameter	palebral_closure	NA	NA	NA
GMC03	SHIRPA_Parameter	lacrimation	NA	NA	NA
GMC03	SHIRPA_Parameter	startle_response	4,07E-03	2,97E-03	7,77E-12
GMC03	SHIRPA_Parameter	limb_grasping	NA	NA	NA
GMC03	SHIRPA_Parameter	trunk_curl	NA	NA	NA
GMC03	SHIRPA_Parameter	pos_pass	NA	NA	NA
GMC03	SHIRPA_Parameter	gait	1,00E+00	6,00E-01	5,36E-01

GMC03	SHIRPA_Parameter	touch_escape	1,00E+00	7,17E-05	9,88E-06
GMC03	SHIRPA_Parameter	pinna_reflex	NA	NA	NA
GMC03	SHIRPA_Parameter	cornea_reflex	NA	NA	NA
GMC03	SHIRPA_Parameter	righting_reflex	NA	NA	NA
GMC03	SHIRPA_Parameter	num_mice_per_cage	1,54E-01	1,28E-04	3,40E-10
GMC03	SHIRPA_Parameter	deafness	4,76E-01	2,33E-04	2,51E-07
GMC04	Rotarod_Parameter	LatFall_1	9,68E-01	2,47E-01	1,15E-01
GMC04	Rotarod_Parameter	LatFall_2	9,09E-02	3,25E-01	6,46E-04
GMC04	Rotarod_Parameter	LatFall_3	5,23E-02	8,09E-01	5,95E-03
GMC04	Rotarod_Parameter	LatFall_mean	1,18E-01	2,98E-01	8,22E-04
GMC05	Acoustic_Startle_Parameter	ASR_bn	3,54E-01	1,25E-01	6,61E-04
GMC05	Acoustic_Startle_Parameter	ASR_70	1,62E-02	9,90E-01	8,67E-03
GMC05	Acoustic_Startle_Parameter	ASR_80	5,92E-01	9,04E-01	8,05E-01
GMC05	Acoustic_Startle_Parameter	ASR_85	2,60E-01	9,79E-02	8,58E-01
GMC05	Acoustic_Startle_Parameter	ASR_90	1,07E-03	8,43E-08	5,92E-02
GMC05	Acoustic_Startle_Parameter	ASR_100	9,56E-07	5,26E-13	1,69E-02
GMC05	Acoustic_Startle_Parameter	ASR_110	1,38E-05	3,07E-13	7,62E-04
GMC05	Acoustic_Startle_Parameter	ASR_120	7,14E-06	1,45E-13	4,99E-04
GMC05	Acoustic_Startle_Parameter	ASR_PP_67	3,49E-13	2,72E-13	4,59E-01
GMC05	Acoustic_Startle_Parameter	ASR_PP_69	2,75E-13	2,72E-13	6,97E-01
GMC05	Acoustic_Startle_Parameter	ASR_PP_73	2,72E-13	2,71E-13	9,55E-01
GMC05	Acoustic_Startle_Parameter	ASR_PP_81	2,75E-13	2,73E-13	9,82E-01
GMC05	Acoustic_Startle_Parameter	PPI_67	1,58E-05	1,79E-03	3,05E-01
GMC05	Acoustic_Startle_Parameter	PPI_69	1,37E-03	3,71E-03	8,96E-01
GMC05	Acoustic_Startle_Parameter	PPI_73	6,77E-02	1,06E-01	9,53E-01
GMC05	Acoustic_Startle_Parameter	PPI_81	2,14E-01	5,26E-01	7,50E-01
GMC05	Acoustic_Startle_Parameter	PPI_global	4,04E-03	2,44E-02	7,20E-01
GMC05	Acoustic_Startle_Parameter	ASR_ISI_5	1,29E-13	1,93E-13	5,95E-01
GMC05	Acoustic_Startle_Parameter	ASR_ISI_25	1,29E-13	1,29E-13	9,61E-01
GMC05	Acoustic_Startle_Parameter	ASR_ISI_100	1,65E-11	1,29E-13	1,39E-01
GMC05	Acoustic_Startle_Parameter	PPI_ISI_5	1,34E-05	8,69E-01	4,25E-08
GMC05	Acoustic_Startle_Parameter	PPI_ISI_25	5,35E-05	5,16E-02	5,38E-02
GMC05	Acoustic_Startle_Parameter	PPI_ISI_100	1,10E-02	7,66E-03	9,97E-01
GMC06	Blood_After_Fasting_Parameter	GLU	7,81E-07	NA	NA
GMC06	Blood_After_Fasting_Parameter	Glycerol	2,83E-02	NA	NA
GMC06	Blood_After_Fasting_Parameter	CHOL	5,37E-09	NA	NA
GMC06	Blood_After_Fasting_Parameter	HDL	5,76E-06	NA	NA
GMC06	Blood_After_Fasting_Parameter	nonHDL	9,43E-12	NA	NA

GMC06	Blood_After_Fasting_Parameter	NEFA	1,99E-02	NA	NA
GMC06	Blood_After_Fasting_Parameter	TG	4,99E-06	NA	NA
GMC07	Hot_plate_Parameter	latency_1	9,70E-01	9,69E-01	1,00E+00
GMC07	Hot_plate_Parameter	latency_2	1,21E-03	1,18E-06	2,14E-01
GMC07	Hot_plate_Parameter	response_1	2,97E-01	5,16E-01	1,10E-02
GMC07	Hot_plate_Parameter	response_2	1,61E-03	1,60E-04	8,41E-01
GMC08	Transepidermal_water_loss_Parameter	TEWL	5,55E-01	4,67E-03	1,36E-05
GMC08	Transepidermal_water_loss_Parameter	TEWL_adj	4,63E-01	2,81E-09	2,63E-13
GMC08	Transepidermal_water_loss_Parameter	TEWL_daily_mean	8,66E-01	9,99E-01	8,08E-01
GMC08	Transepidermal_water_loss_Parameter	TEWL_ambient_temp	9,37E-01	5,28E-01	7,04E-01
GMC08	Transepidermal_water_loss_Parameter	TEWL_rel_humidity	9,64E-01	8,32E-01	9,36E-01
GMC08	Transepidermal_water_loss_Parameter	TEWL_CV	5,62E-01	4,47E-03	1,35E-05
GMC08	Transepidermal_water_loss_Parameter	SSWL	3,72E-01	9,38E-01	1,41E-01
GMC09	Calorimetry_Parameter	food	3,48E-12	2,11E-07	5,55E-02
GMC09	Calorimetry_Parameter	water	2,05E-04	1,31E-05	8,08E-01
GMC09	Calorimetry_Parameter	VO2_mean	2,30E-13	2,30E-13	1,97E-01
GMC09	Calorimetry_Parameter	VCO2_mean	2,30E-13	2,30E-13	1,16E-01
GMC09	Calorimetry_Parameter	RER_mean	3,76E-01	9,56E-01	4,58E-01
GMC09	Calorimetry_Parameter	heat_mean	2,30E-13	2,30E-13	1,61E-01
GMC09	Calorimetry_Parameter	breaks_X_mean	1,47E-02	8,14E-01	3,53E-02
GMC09	Calorimetry_Parameter	breaks_XA_mean	1,34E-02	6,33E-01	7,33E-02
GMC09	Calorimetry_Parameter	breaks_XF_mean	5,39E-02	2,48E-01	4,93E-05
GMC09	Calorimetry_Parameter	breaks_YA_mean	1,68E-03	5,90E-01	2,99E-06
GMC09	Calorimetry_Parameter	breaks_YF_mean	1,82E-03	8,77E-03	1,97E-11
GMC09	Calorimetry_Parameter	breaks_Z_mean	1,03E-03	3,89E-03	1,35E-12
GMC09	Calorimetry_Parameter	distance_mean	1,34E-01	2,76E-03	4,24E-08
GMC09	Calorimetry_Parameter	speed_mean	1,31E-01	2,95E-03	4,43E-08
GMC10	Minispec_NMR_13_Parameter	fat_13wk	1,77E-13	1,73E-13	3,01E-06
GMC10	Minispec_NMR_13_Parameter	lean_13wk	1,73E-13	1,73E-13	1,88E-13
GMC10	Minispec_NMR_19_Parameter	fat_19wk	1,15E-13	8,84E-14	2,48E-03
GMC10	Minispec_NMR_19_Parameter	lean_19wk	1,16E-13	8,84E-14	1,16E-13
GMC11	IGTT_Parameter	GLU_0	8,02E-03	4,06E-02	1,66E-04
GMC11	IGTT_Parameter	GLU_15	9,99E-01	1,27E-01	1,21E-01
GMC11	IGTT_Parameter	GLU_30	1,44E-01	4,81E-01	9,86E-01
GMC11	IGTT_Parameter	GLU_60	9,05E-08	3,20E-03	9,73E-01
GMC11	IGTT_Parameter	GLU_120	2,82E-12	1,88E-02	3,64E-01
GMC11	IGTT_Parameter	GLU_vol_inj	0,00E+00	7,69E-13	1,00E-01
GMC11	IGTT_Parameter	AUC_by_HAMED	1,31E-05	9,00E-14	9,00E-14

GMC12	Echocardiography_Parameter	resp_rate	9,78E-02	2,33E-02	8,81E-01
GMC12	Echocardiography_Parameter	heart_rate	4,67E-01	2,53E-02	2,68E-01
GMC12	Echocardiography_Parameter	LV_mass	3,82E-08	3,74E-05	2,09E-01
GMC12	Echocardiography_Parameter	fract_shortening	4,63E-01	2,49E-02	1,02E-04
GMC12	Echocardiography_Parameter	EJ_fraction	8,66E-01	1,13E-04	1,31E-06
GMC12	Echocardiography_Parameter	stroke_vol	1,83E-10	1,68E-07	2,90E-01
GMC12	Echocardiography_Parameter	cardiac_output	1,93E-14	1,84E-14	6,69E-01
GMC12	Echocardiography_Parameter	IVS_diastole	4,11E-02	1,44E-08	3,26E-04
GMC12	Echocardiography_Parameter	IVS_systole	1,10E-03	9,75E-08	6,91E-02
GMC12	Echocardiography_Parameter	LVID_diastole	6,52E-07	6,14E-03	2,89E-02
GMC12	Echocardiography_Parameter	LVID_systole	6,54E-02	2,87E-01	1,16E-04
GMC12	Echocardiography_Parameter	LVPW_diastole	1,84E-01	2,42E-04	4,30E-02
GMC12	Echocardiography_Parameter	LVPW_systole	1,35E-02	1,00E-05	1,06E-01
GMC13	Electrocardiogram_Parameter	num_ECG_signals	8,25E-01	2,82E-03	1,46E-04
GMC13	Electrocardiogram_Parameter	HR	1,12E-04	1,21E-13	0,00E+00
GMC13	Electrocardiogram_Parameter	HR_V	3,03E-01	6,53E-01	9,65E-01
GMC13	Electrocardiogram_Parameter	HR_CV	3,18E-01	5,91E-01	9,92E-01
GMC13	Electrocardiogram_Parameter	RR	1,94E-04	5,12E-12	0,00E+00
GMC13	Electrocardiogram_Parameter	PQ	7,49E-01	5,68E-10	4,24E-10
GMC13	Electrocardiogram_Parameter	PR	5,54E-01	5,74E-09	2,07E-08
GMC13	Electrocardiogram_Parameter	QRS	3,43E-01	8,59E-01	1,97E-01
GMC13	Electrocardiogram_Parameter	QT	7,38E-01	1,80E-04	2,44E-06
GMC13	Electrocardiogram_Parameter	ST	5,15E-01	5,16E-05	1,18E-07
GMC13	Electrocardiogram_Parameter	QTc	8,81E-01	5,15E-02	7,71E-02
GMC13	Electrocardiogram_Parameter	QT_dispersion	3,76E-01	8,26E-01	8,92E-01
GMC13	Electrocardiogram_Parameter	QTc_dispersion	7,21E-01	6,91E-01	9,63E-01
GMC13	Electrocardiogram_Parameter	SR_amplitude	1,46E-03	6,44E-01	1,31E-01
GMC13	Electrocardiogram_Parameter	R_amplitude	8,73E-04	9,59E-01	1,88E-02
GMC13	Electrocardiogram_Parameter	rMSSD	6,24E-01	9,42E-01	9,01E-01
GMC13	Electrocardiogram_Parameter	pNN50	5,69E-01	3,21E-01	7,42E-01
GMC14	Eye_size_Parameter	body_length	1,75E-14	0,00E+00	0,00E+00
GMC14	Eye_size_Parameter	eye_length_L	0,00E+00	0,00E+00	0,00E+00
GMC14	Eye_size_Parameter	eye_length_R	4,12E-14	4,12E-14	4,14E-14
GMC14	Eye_size_Parameter	lens_min_density_L	4,20E-03	3,28E-04	4,15E-11
GMC14	Eye_size_Parameter	lens_min_density_R	1,19E-01	2,52E-04	1,96E-08
GMC14	Eye_size_Parameter	lens_max_density_L	8,92E-03	2,54E-02	9,95E-01
GMC14	Eye_size_Parameter	lens_max_density_R	9,72E-04	4,86E-01	9,75E-02
GMC14	Eye_size_Parameter	lens_mean_density_L	7,12E-06	5,45E-02	1,71E-10

GMC14	Eye_size_Parameter	lens_mean_density_R	3,56E-04	1,68E-02	1,91E-09
GMC14	Eye_size_Parameter	fundus_vessels_L	5,85E-01	8,41E-03	2,81E-04
GMC14	Eye_size_Parameter	fundus_vessels_R	9,98E-01	1,36E-05	4,89E-06
GMC14	Eye_size_Parameter	retinal_thickness_L	8,96E-14	6,24E-01	2,42E-13
GMC14	Eye_size_Parameter	retinal_thickness_R	0,00E+00	7,71E-02	2,61E-07
GMC14	Eye_size_Parameter	spatial_freq	1,09E-08	NA	NA
GMC15	Hematology_Week_21_Parameter	WBC	8,58E-02	6,72E-01	3,25E-01
GMC15	Hematology_Week_21_Parameter	RBC	1,61E-02	3,06E-01	3,03E-01
GMC15	Hematology_Week_21_Parameter	RDW	2,36E-05	6,89E-10	4,95E-02
GMC15	Hematology_Week_21_Parameter	MCV	9,27E-04	4,88E-12	5,68E-05
GMC15	Hematology_Week_21_Parameter	MCH	1,04E-01	1,68E-04	5,56E-02
GMC15	Hematology_Week_21_Parameter	MCHC	2,26E-02	6,19E-09	1,99E-04
GMC15	Hematology_Week_21_Parameter	HGB	3,44E-04	1,34E-03	9,04E-01
GMC15	Hematology_Week_21_Parameter	HCT	2,17E-06	2,20E-08	5,57E-01
GMC15	Hematology_Week_21_Parameter	PLT	4,00E-03	3,82E-07	3,34E-02
GMC15	Hematology_Week_21_Parameter	MPV	1,91E-12	7,11E-15	6,48E-02
GMC15	Hematology_Week_21_Parameter	PDW	1,48E-12	8,55E-15	4,70E-01
GMC15	Hematology_Week_21_Parameter	PLCR	6,66E-10	1,53E-08	7,72E-01
GMC15	Hematology_Week_21_Parameter	PCT	2,47E-06	1,76E-12	8,67E-03
GMC16	Clinical_chemistry_week_17_Parameter	calcium_17	4,53E-01	2,59E-04	1,66E-03
GMC16	Clinical_chemistry_week_17_Parameter	chloride_17	2,75E-05	1,05E-01	4,27E-01
GMC16	Clinical_chemistry_week_17_Parameter	iron_17	4,95E-01	1,32E-02	5,83E-02
GMC16	Clinical_chemistry_week_17_Parameter	lactate_17	7,34E-01	4,72E-03	7,44E-04
GMC16	Clinical_chemistry_week_17_Parameter	phosphate_17	2,45E-02	5,77E-01	7,39E-01
GMC16	Clinical_chemistry_week_17_Parameter	potassium_17	1,86E-02	9,02E-02	9,37E-01
GMC16	Clinical_chemistry_week_17_Parameter	sodium_17	2,29E-01	8,85E-01	7,97E-01
GMC16	Clinical_chemistry_week_17_Parameter	ALP_17	6,67E-02	6,77E-02	6,56E-01
GMC16	Clinical_chemistry_week_17_Parameter	ALT_17	9,35E-01	5,04E-01	3,34E-01
GMC16	Clinical_chemistry_week_17_Parameter	AST_17	4,17E-01	9,97E-01	7,07E-01
GMC16	Clinical_chemistry_week_17_Parameter	LDH_17	1,85E-01	9,33E-01	2,80E-01
GMC16	Clinical_chemistry_week_17_Parameter	amylase_17	7,70E-03	1,90E-03	2,01E-01
GMC16	Clinical_chemistry_week_17_Parameter	GLU_17	5,89E-01	6,35E-01	9,51E-01
GMC16	Clinical_chemistry_week_17_Parameter	creatinine_17	4,98E-02	5,26E-01	2,22E-02
GMC16	Clinical_chemistry_week_17_Parameter	albumin_17	1,51E-02	1,20E-02	2,61E-05
GMC16	Clinical_chemistry_week_17_Parameter	total_protein_17	8,23E-01	2,05E-03	3,77E-03
GMC16	Clinical_chemistry_week_17_Parameter	urea_17	5,56E-02	7,79E-01	7,16E-02
GMC16	Clinical_chemistry_week_17_Parameter	CHOL_17	7,43E-03	2,58E-01	9,03E-01
GMC16	Clinical_chemistry_week_17_Parameter	TG_17	3,80E-05	2,98E-03	9,22E-01

GMC16	Clinical_chemistry_week_21_Parameter	calcium_21	6,47E-01	2,56E-08	9,78E-08
GMC16	Clinical_chemistry_week_21_Parameter	chloride_21	3,79E-08	0,00E+00	1,32E-08
GMC16	Clinical_chemistry_week_21_Parameter	iron_21	9,32E-01	9,66E-04	7,54E-04
GMC16	Clinical_chemistry_week_21_Parameter	iron_binding_21	5,31E-01	5,02E-01	9,94E-01
GMC16	Clinical_chemistry_week_21_Parameter	lactate_21	5,55E-02	1,26E-03	1,23E-09
GMC16	Clinical_chemistry_week_21_Parameter	phosphate_21	5,37E-02	7,76E-01	2,51E-03
GMC16	Clinical_chemistry_week_21_Parameter	potassium_21	6,33E-01	1,00E+00	5,49E-01
GMC16	Clinical_chemistry_week_21_Parameter	sodium_21	5,36E-01	4,76E-01	9,93E-01
GMC16	Clinical_chemistry_week_21_Parameter	ALP_21	8,54E-01	6,40E-05	8,57E-05
GMC16	Clinical_chemistry_week_21_Parameter	ALT_21	8,95E-01	4,20E-01	1,37E-01
GMC16	Clinical_chemistry_week_21_Parameter	AST_21	1,32E-01	7,62E-02	9,34E-01
GMC16	Clinical_chemistry_week_21_Parameter	LDH_21	6,46E-02	1,08E-01	9,74E-01
GMC16	Clinical_chemistry_week_21_Parameter	amylase_21	1,34E-03	9,66E-15	1,08E-12
GMC16	Clinical_chemistry_week_21_Parameter	GLU_21	5,77E-06	2,81E-03	1,78E-01
GMC16	Clinical_chemistry_week_21_Parameter	albumin_21	2,22E-01	9,86E-04	2,57E-08
GMC16	Clinical_chemistry_week_21_Parameter	creatinine_21	9,12E-01	9,48E-01	6,98E-01
GMC16	Clinical_chemistry_week_21_Parameter	total_protein_21	9,85E-01	4,04E-14	0,00E+00
GMC16	Clinical_chemistry_week_21_Parameter	urea_21	7,16E-01	2,22E-07	5,64E-07
GMC16	Clinical_chemistry_week_21_Parameter	CHOL_21	1,14E-11	1,54E-14	1,70E-01
GMC16	Clinical_chemistry_week_21_Parameter	TG_21	1,49E-09	5,35E-07	3,87E-01
GMC17	ABR_Parameter	click	9,67E-08	2,85E-13	3,00E-05
GMC17	ABR_Parameter	pip_6	7,07E-05	3,78E-07	1,38E-02
GMC17	ABR_Parameter	pip_12	8,99E-10	2,34E-11	4,88E-03
GMC17	ABR_Parameter	pip_18	9,84E-11	3,04E-13	9,21E-04
GMC17	ABR_Parameter	pip_24	9,66E-07	1,37E-13	4,59E-06
GMC17	ABR_Parameter	pip_30	1,57E-02	7,07E-12	3,48E-08
GMC17	ABR_Parameter	Extens_stimulus_lvls	NA	NA	NA
GMC18	Dual_Energy_Xray_Absorptiometry_Parameter	body_length	2,08E-14	2,25E-14	3,91E-10
GMC18	Dual_Energy_Xray_Absorptiometry_Parameter	body_size	0,00E+00	0,00E+00	2,17E-04
GMC18	Dual_Energy_Xray_Absorptiometry_Parameter	body_type	1,20E-07	1,91E-13	5,36E-05
GMC18	Dual_Energy_Xray_Absorptiometry_Parameter	FATmass_Xhead	7,69E-14	1,40E-03	8,94E-01
GMC18	Dual_Energy_Xray_Absorptiometry_Parameter	FATmass_wholebody	7,99E-14	1,34E-03	8,84E-01
GMC18	Dual_Energy_Xray_Absorptiometry_Parameter	LEANmass_Xhead	5,13E-01	3,30E-02	9,46E-02
GMC18	Dual_Energy_Xray_Absorptiometry_Parameter	LEANmass_wholebody	8,45E-01	9,50E-02	1,46E-01
GMC18	Dual_Energy_Xray_Absorptiometry_Parameter	SOFTmass_Xhead	4,57E-14	2,67E-08	2,28E-01
GMC18	Dual_Energy_Xray_Absorptiometry_Parameter	SOFTmass_wholebody	4,47E-14	1,49E-08	2,02E-01
GMC18	Dual_Energy_Xray_Absorptiometry_Parameter	BMD_Xhead	4,03E-02	6,53E-02	4,38E-01
GMC18	Dual_Energy_Xray_Absorptiometry_Parameter	BMD_wholebody	9,73E-01	1,99E-01	2,24E-01

GMC18	Dual_Energy_Xray_Absorptiometry_Parameter	bone_area_Xhead	3,01E-14	3,31E-09	2,39E-01
GMC18	Dual_Energy_Xray_Absorptiometry_Parameter	bone_area_wholebody	2,83E-14	1,62E-09	2,06E-01
GMC18	Dual_Energy_Xray_Absorptiometry_Parameter	bone_mass_Xhead	4,71E-14	2,13E-07	4,02E-01
GMC18	Dual_Energy_Xray_Absorptiometry_Parameter	bone_mass_wholebody	4,87E-14	1,11E-07	2,84E-01
GMC18	Dual_Energy_Xray_Absorptiometry_Parameter	lumbar_num	1,56E-04	1,00E+00	8,09E-03
GMC19	Lung_function_Parameter	residual_vol	2,16E-01	1,61E-01	8,55E-01
GMC19	Lung_function_Parameter	expiratory_vol	6,75E-01	6,20E-02	1,24E-01
GMC19	Lung_function_Parameter	forced_expiratory_vol	9,86E-01	4,35E-03	5,56E-04
GMC19	Lung_function_Parameter	tidal_vol	4,31E-01	2,64E-01	8,82E-01
GMC19	Lung_function_Parameter	lung_capacity	7,84E-01	1,80E-03	1,45E-03
GMC19	Lung_function_Parameter	vital_capacity	9,43E-01	2,30E-03	6,52E-04
GMC19	Lung_function_Parameter	forced_capacity	9,64E-01	3,37E-03	1,06E-03
GMC19	Lung_function_Parameter	inspiratory_capacity	9,98E-01	1,67E-02	4,82E-03
GMC19	Lung_function_Parameter	residual_capacity	5,60E-01	8,70E-01	2,88E-01
GMC19	Lung_function_Parameter	chord_compliance	5,57E-01	2,89E-02	5,19E-04
GMC19	Lung_function_Parameter	dynamic_compliance	5,87E-01	2,06E-07	5,25E-07
GMC19	Lung_function_Parameter	resistance	7,66E-01	4,99E-04	2,71E-05
GMC19	Lung_function_Parameter	peak_flow	2,84E-01	9,32E-01	5,06E-01
GMC20	All_immunoglobulins_week_17_21_Parameter	IgM_17	4,05E-05	1,13E-04	2,72E-01
GMC20	All_immunoglobulins_week_17_21_Parameter	IgM_21	1,44E-06	4,43E-08	5,34E-01
GMC20	All_immunoglobulins_week_17_21_Parameter	IgA_17	2,52E-01	1,91E-01	6,67E-01
GMC20	All_immunoglobulins_week_17_21_Parameter	IgA_21	7,21E-01	3,23E-01	5,97E-01
GMC20	All_immunoglobulins_week_17_21_Parameter	IgG1_17	4,46E-01	6,89E-01	9,12E-01
GMC20	All_immunoglobulins_week_17_21_Parameter	IgG1_21	9,61E-01	5,18E-01	1,49E-01
GMC20	All_immunoglobulins_week_17_21_Parameter	IgG2a_17	5,34E-01	6,39E-01	2,65E-01
GMC20	All_immunoglobulins_week_17_21_Parameter	IgG2a_21	3,70E-02	4,14E-01	3,39E-05
GMC20	All_immunoglobulins_week_17_21_Parameter	IgG2b_17	1,71E-02	NA	NA
GMC20	All_immunoglobulins_week_17_21_Parameter	IgG2b_21	4,65E-02	2,71E-05	5,59E-03
GMC20	All_immunoglobulins_week_17_21_Parameter	IgG3_17	2,70E-01	2,53E-02	1,54E-01
GMC20	All_immunoglobulins_week_17_21_Parameter	IgG3_21	2,35E-01	8,56E-01	3,60E-01
GMC20	All_immunoglobulins_week_17_21_Parameter	IgE_17	9,54E-01	7,05E-01	5,79E-01
GMC20	All_immunoglobulins_week_17_21_Parameter	IgE_21	2,60E-02	8,42E-01	1,06E-03
GMC20	All_immunoglobulins_week_17_21_Parameter	aDNA_17	1,10E-12	3,16E-09	8,68E-01
GMC20	All_immunoglobulins_week_17_21_Parameter	aDNA_21	2,11E-15	0,00E+00	6,63E-03
GMC20	All_immunoglobulins_week_17_21_Parameter	RF_17	4,38E-09	1,64E-05	9,30E-01
GMC20	All_immunoglobulins_week_17_21_Parameter	RF_21	8,69E-12	3,01E-14	3,39E-01
GMC21	Organ_weights_at_section_Parameter	tibia_length	9,63E-01	6,10E-14	6,07E-14
GMC21	Organ_weights_at_section_Parameter	heart_wt	1,64E-08	6,15E-14	2,81E-03

GMC21	Organ_weights_at_section_Parameter	liver_wt	5,61E-14	3,41E-14	1,61E-04
GMC21	Organ_weights_at_section_Parameter	spleen_wt	0,00E+00	0,00E+00	3,06E-11
