# Supplementary material for: A comprehensive and comparative phenotypic analysis of the collaborative founder strains identifies new and known phenotypes
Source: Mamm Genome. 2020 Feb 14;31(1):30–48. doi: 10.1007/s00335-020-09827-3 (PMC7060152; doi:10.1007/s00335-020-09827-3)
Supplement: Supplementary file 11 — Supplementary file11 (PDF 78 kb) [file 335_2020_9827_MOESM11_ESM.pdf]

Table S6

Anova model: respose~shipment date				
Protocol	Procedure	Parameter	Pvalue	Note
GMC01	Open_field_Parameter	distance_1	6,67E-01	NA
GMC01	Open_field_Parameter	distance_2	2,70E-01	NA
GMC01	Open_field_Parameter	distance_3	3,06E-01	NA
GMC01	Open_field_Parameter	distance_4	5,53E-01	NA
GMC01	Open_field_Parameter	distance_total	4,37E-01	NA
GMC01	Open_field_Parameter	rears_1	2,01E-04	NA
GMC01	Open_field_Parameter	rears_2	7,16E-05	NA
GMC01	Open_field_Parameter	rears_3	1,23E-02	NA
GMC01	Open_field_Parameter	rears_4	1,38E-02	NA
GMC01	Open_field_Parameter	rears_total	4,48E-04	NA
GMC01	Open_field_Parameter	whole_rest	3,26E-03	NA
GMC01	Open_field_Parameter	whole_speed	4,43E-01	NA
GMC01	Open_field_Parameter	center_distance	4,04E-01	NA
GMC01	Open_field_Parameter	center_rest	5,54E-06	NA
GMC01	Open_field_Parameter	center_permanence	3,93E-01	NA
GMC01	Open_field_Parameter	center_speed	4,57E-02	NA
GMC01	Open_field_Parameter	center_latency	1,26E-01	NA
GMC01	Open_field_Parameter	center_entries	4,36E-01	NA
GMC01	Open_field_Parameter	center_distance_1	6,17E-01	NA
GMC01	Open_field_Parameter	center_distance_2	2,76E-01	NA
GMC01	Open_field_Parameter	center_distance_3	5,84E-01	NA
GMC01	Open_field_Parameter	center_distance_4	9,44E-01	NA
GMC01	Open_field_Parameter	center_distance_total	6,36E-01	NA
GMC01	Open_field_Parameter	center_time_1	5,58E-01	NA
GMC01	Open_field_Parameter	center_time_2	5,97E-02	NA
GMC01	Open_field_Parameter	center_time_3	4,00E-01	NA
GMC01	Open_field_Parameter	center_time_4	7,95E-01	NA
GMC01	Open_field_Parameter	center_time_total	3,93E-01	NA
GMC01	Open_field_Parameter	periphery_distance	2,74E-01	NA
GMC01	Open_field_Parameter	periphery_rest	4,83E-03	NA
GMC01	Open_field_Parameter	periphery_permanence	3,93E-01	NA
GMC01	Open_field_Parameter	periphery_speed	4,33E-01	NA
GMC02	Grip_strength_Parameter	front_paws1	3,48E-03	NA
GMC02	Grip_strength_Parameter	front_paws2	3,04E-02	NA
GMC02	Grip_strength_Parameter	front_paws3	1,97E-03	NA
GMC02	Grip_strength_Parameter	front_paws_mean	3,95E-03	NA
GMC02	Grip_strength_Parameter	front_paws_adj	1,59E-02	NA
GMC02	Grip_strength_Parameter	all_paws1	3,90E-02	NA
GMC02	Grip_strength_Parameter	all_paws2	9,20E-02	NA
GMC02	Grip_strength_Parameter	all_paws3	5,37E-02	NA
GMC02	Grip_strength_Parameter	all_paws_mean	5,69E-02	NA
GMC02	Grip_strength_Parameter	all_paws_adj	2,64E-01	NA
GMC03	SHIRPA_Parameter	coat_app	1,29E-03	NA
GMC03	SHIRPA_Parameter	whiskers	7,57E-01	NA
GMC03	SHIRPA_Parameter	body_pos	3,53E-03	NA
GMC03	SHIRPA_Parameter	pelvic_elev	1,56E-01	NA
GMC03	SHIRPA_Parameter	tail_elev	2,82E-01	NA
GMC03	SHIRPA_Parameter	transfer_arousal	6,42E-02	NA
GMC03	SHIRPA_Parameter	defecation	1,60E-01	NA
GMC03	SHIRPA_Parameter	urinate	2,05E-02	NA
GMC03	SHIRPA_Parameter	tremor	4,77E-01	NA
GMC03	SHIRPA_Parameter	loco_activity	2,56E-01	NA
GMC03	SHIRPA_Parameter	bite_evidence	4,38E-04	NA
GMC03	SHIRPA_Parameter	vocalization	4,30E-02	NA
GMC03	SHIRPA_Parameter	palebral_closure	NA	No variation in response

GMC03	SHIRPA_Parameter	lacrimation	NA	No variation in response
GMC03	SHIRPA_Parameter	startle_response	1,21E-02	NA
GMC03	SHIRPA_Parameter	limb_grasping	NA	No variation in response
GMC03	SHIRPA_Parameter	trunk_curl	NA	No variation in response
GMC03	SHIRPA_Parameter	pos_pass	NA	No variation in response
GMC03	SHIRPA_Parameter	gait	5,51E-01	NA
GMC03	SHIRPA_Parameter	touch_escape	6,82E-05	NA
GMC03	SHIRPA_Parameter	pinna_reflex	NA	No variation in response
GMC03	SHIRPA_Parameter	cornea_reflex	NA	No variation in response
GMC03	SHIRPA_Parameter	righting_reflex	NA	No variation in response
GMC03	SHIRPA_Parameter	num_mice_per_cage	1,18E-04	NA
GMC03	SHIRPA_Parameter	deafness	4,18E-01	NA
GMC04	Rotarod_Parameter	LatFall_1	1,84E-02	NA
GMC04	Rotarod_Parameter	LatFall_2	7,96E-02	NA
GMC04	Rotarod_Parameter	LatFall_3	2,74E-02	NA
GMC04	Rotarod_Parameter	LatFall_mean	8,18E-03	NA
GMC05	Acoustic_Startle_Parameter	ASR_bn	7,56E-04	NA
GMC05	Acoustic_Startle_Parameter	ASR_70	8,93E-02	NA
GMC05	Acoustic_Startle_Parameter	ASR_80	2,97E-01	NA
GMC05	Acoustic_Startle_Parameter	ASR_85	2,44E-01	NA
GMC05	Acoustic_Startle_Parameter	ASR_90	6,78E-01	NA
GMC05	Acoustic_Startle_Parameter	ASR_100	9,78E-01	NA
GMC05	Acoustic_Startle_Parameter	ASR_110	8,38E-01	NA
GMC05	Acoustic_Startle_Parameter	ASR_120	9,71E-01	NA
GMC05	Acoustic_Startle_Parameter	ASR_PP_67	7,28E-01	NA
GMC05	Acoustic_Startle_Parameter	ASR_PP_69	7,04E-01	NA
GMC05	Acoustic_Startle_Parameter	ASR_PP_73	7,95E-01	NA
GMC05	Acoustic_Startle_Parameter	ASR_PP_81	8,66E-01	NA
GMC05	Acoustic_Startle_Parameter	PPI_67	1,06E-02	NA
GMC05	Acoustic_Startle_Parameter	PPI_69	1,56E-02	NA
GMC05	Acoustic_Startle_Parameter	PPI_73	6,18E-02	NA
GMC05	Acoustic_Startle_Parameter	PPI_81	1,44E-01	NA
GMC05	Acoustic_Startle_Parameter	PPI_global	2,46E-02	NA
GMC05	Acoustic_Startle_Parameter	ASR_ISI_5	6,29E-01	NA
GMC05	Acoustic_Startle_Parameter	ASR_ISI_25	5,27E-01	NA
GMC05	Acoustic_Startle_Parameter	ASR_ISI_100	9,04E-01	NA
GMC05	Acoustic_Startle_Parameter	PPI_ISI_5	2,39E-01	NA
GMC05	Acoustic_Startle_Parameter	PPI_ISI_25	2,59E-04	NA
GMC05	Acoustic_Startle_Parameter	PPI_ISI_100	1,11E-02	NA
GMC06	Blood_After_Fasting_Parameter	GLU	2,61E-01	NA
GMC06	Blood_After_Fasting_Parameter	Glycerol	2,84E-25	NA
GMC06	Blood_After_Fasting_Parameter	CHOL	3,92E-01	NA
GMC06	Blood_After_Fasting_Parameter	HDL	5,37E-01	NA
GMC06	Blood_After_Fasting_Parameter	nonHDL	1,02E-02	NA
GMC06	Blood_After_Fasting_Parameter	NEFA	2,55E-30	NA
GMC06	Blood_After_Fasting_Parameter	TG	9,50E-05	NA
GMC07	Hot_plate_Parameter	latency_1	2,58E-05	NA
GMC07	Hot_plate_Parameter	latency_2	1,05E-03	NA
GMC07	Hot_plate_Parameter	response_1	1,17E-22	NA
GMC07	Hot_plate_Parameter	response_2	4,33E-06	NA
GMC08	Transepidermal_water_loss_Parameter	TEWL	3,36E-69	NA
GMC08	Transepidermal_water_loss_Parameter	TEWL_adj	1,00E+00	NA
GMC08	Transepidermal_water_loss_Parameter	TEWL_daily_mean	0,00E+00	NA
GMC08	Transepidermal_water_loss_Parameter	TEWL_ambient_temp	3,84E-256	NA
GMC08	Transepidermal_water_loss_Parameter	TEWL_rel_humidity	1,36E-254	NA
GMC08	Transepidermal_water_loss_Parameter	TEWL_CV	9,79E-02	NA
GMC08	Transepidermal_water_loss_Parameter	SSWL	1,24E-17	NA
GMC09	Calorimetry_Parameter	food	1,64E-01	NA
GMC09	Calorimetry_Parameter	water	5,12E-03	NA
GMC09	Calorimetry_Parameter	VO2_mean	6,08E-01	NA
GMC09	Calorimetry_Parameter	VCO2_mean	4,38E-01	NA
GMC09	Calorimetry_Parameter	RER_mean	4,33E-04	NA

GMC09	Calorimetry_Parameter	heat_mean	6,79E-01	NA
GMC09	Calorimetry_Parameter	breaks_X_mean	2,55E-01	NA
GMC09	Calorimetry_Parameter	breaks_XA_mean	2,11E-01	NA
GMC09	Calorimetry_Parameter	breaks_XF_mean	4,09E-01	NA
GMC09	Calorimetry_Parameter	breaks_YA_mean	1,44E-01	NA
GMC09	Calorimetry_Parameter	breaks_YF_mean	5,77E-02	NA
GMC09	Calorimetry_Parameter	breaks_Z_mean	2,91E-04	NA
GMC09	Calorimetry_Parameter	distance_mean	1,64E-04	NA
GMC09	Calorimetry_Parameter	speed_mean	1,60E-04	NA
GMC10	Minispec_NMR_13_Parameter	fat_13wk	1,15E-01	NA
GMC10	Minispec_NMR_13_Parameter	lean_13wk	2,13E-01	NA
GMC10	Minispec_NMR_19_Parameter	fat_19wk	5,10E-01	NA
GMC10	Minispec_NMR_19_Parameter	lean_19wk	2,10E-01	NA
GMC11	IGTT_Parameter	GLU_0	7,41E-02	NA
GMC11	IGTT_Parameter	GLU_15	5,82E-02	NA
GMC11	IGTT_Parameter	GLU_30	2,91E-01	NA
GMC11	IGTT_Parameter	GLU_60	6,88E-01	NA
GMC11	IGTT_Parameter	GLU_120	9,40E-01	NA
GMC11	IGTT_Parameter	GLU_vol_inj	7,90E-01	NA
GMC11	IGTT_Parameter	AUC_by_HAMED	1,68E-03	NA
GMC12	Echocardiography_Parameter	resp_rate	6,33E-13	NA
GMC12	Echocardiography_Parameter	heart_rate	4,08E-06	NA
GMC12	Echocardiography_Parameter	LV_mass	1,63E-09	NA
GMC12	Echocardiography_Parameter	fract_shortening	4,73E-11	NA
GMC12	Echocardiography_Parameter	EJ_fraction	8,80E-18	NA
GMC12	Echocardiography_Parameter	stroke_vol	1,08E-07	NA
GMC12	Echocardiography_Parameter	cardiac_output	4,78E-01	NA
GMC12	Echocardiography_Parameter	IVS_diastole	6,36E-10	NA
GMC12	Echocardiography_Parameter	IVS_systole	8,55E-11	NA
GMC12	Echocardiography_Parameter	LVID_diastole	7,49E-12	NA
GMC12	Echocardiography_Parameter	LVID_systole	4,32E-16	NA
GMC12	Echocardiography_Parameter	LVPW_diastole	1,79E-30	NA
GMC12	Echocardiography_Parameter	LVPW_systole	3,48E-22	NA
GMC13	Electrocardiogram_Parameter	num_ECG_signals	6,75E-04	NA
GMC13	Electrocardiogram_Parameter	HR	3,92E-04	NA
GMC13	Electrocardiogram_Parameter	HR_V	4,56E-02	NA
GMC13	Electrocardiogram_Parameter	HR_CV	3,37E-02	NA
GMC13	Electrocardiogram_Parameter	RR	5,33E-04	NA
GMC13	Electrocardiogram_Parameter	PQ	2,59E-03	NA
GMC13	Electrocardiogram_Parameter	PR	1,58E-04	NA
GMC13	Electrocardiogram_Parameter	QRS	1,43E-02	NA
GMC13	Electrocardiogram_Parameter	QT	1,12E-03	NA
GMC13	Electrocardiogram_Parameter	ST	1,13E-05	NA
GMC13	Electrocardiogram_Parameter	QTc	4,38E-04	NA
GMC13	Electrocardiogram_Parameter	QT_dispersion	3,42E-02	NA
GMC13	Electrocardiogram_Parameter	QTc_dispersion	4,90E-02	NA
GMC13	Electrocardiogram_Parameter	SR_amplitude	1,04E-06	NA
GMC13	Electrocardiogram_Parameter	R_amplitude	6,68E-08	NA
GMC13	Electrocardiogram_Parameter	rMSSD	5,57E-02	NA
GMC13	Electrocardiogram_Parameter	pNN50	6,35E-02	NA
GMC14	Eye_size_Parameter	body_length	3,69E-02	NA
GMC14	Eye_size_Parameter	eye_length_L	3,64E-01	NA
GMC14	Eye_size_Parameter	eye_length_R	5,12E-01	NA
GMC14	Eye_size_Parameter	lens_min_density_L	5,02E-01	NA
GMC14	Eye_size_Parameter	lens_min_density_R	4,82E-01	NA
GMC14	Eye_size_Parameter	lens_max_density_L	1,48E-07	NA
GMC14	Eye_size_Parameter	lens_max_density_R	4,70E-07	NA
GMC14	Eye_size_Parameter	lens_mean_density_L	7,56E-02	NA
GMC14	Eye_size_Parameter	lens_mean_density_R	4,61E-01	NA
GMC14	Eye_size_Parameter	fundus_vessels_L	3,46E-05	NA
GMC14	Eye_size_Parameter	fundus_vessels_R	2,03E-03	NA
GMC14	Eye_size_Parameter	retinal_thickness_L	8,52E-01	NA

GMC14	Eye_size_Parameter	retinal_thickness_R	3,49E-01	NA
GMC14	Eye_size_Parameter	spatial_freq	3,88E-01	NA
GMC15	Hematology_Week_21_Parameter	WBC	1,44E-04	NA
GMC15	Hematology_Week_21_Parameter	RBC	1,48E-01	NA
GMC15	Hematology_Week_21_Parameter	RDW	3,92E-04	NA
GMC15	Hematology_Week_21_Parameter	MCV	7,31E-03	NA
GMC15	Hematology_Week_21_Parameter	MCH	6,08E-01	NA
GMC15	Hematology_Week_21_Parameter	MCHC	4,99E-09	NA
GMC15	Hematology_Week_21_Parameter	HGB	1,31E-01	NA
GMC15	Hematology_Week_21_Parameter	HCT	8,03E-01	NA
GMC15	Hematology_Week_21_Parameter	PLT	2,55E-02	NA
GMC15	Hematology_Week_21_Parameter	MPV	7,94E-02	NA
GMC15	Hematology_Week_21_Parameter	PDW	1,80E-02	NA
GMC15	Hematology_Week_21_Parameter	PLCR	2,31E-02	NA
GMC15	Hematology_Week_21_Parameter	PCT	6,17E-02	NA
GMC16	Clinical_chemistry_week_17_Parameter	calcium_17	NA	Only data on 15/01/2013
GMC16	Clinical_chemistry_week_17_Parameter	chloride_17	NA	Only data on 15/01/2013
GMC16	Clinical_chemistry_week_17_Parameter	iron_17	NA	Only data on 15/01/2013
GMC16	Clinical_chemistry_week_17_Parameter	lactate_17	NA	Only data on 15/01/2013
GMC16	Clinical_chemistry_week_17_Parameter	phosphate_17	NA	Only data on 15/01/2013
GMC16	Clinical_chemistry_week_17_Parameter	potassium_17	NA	Only data on 15/01/2013
GMC16	Clinical_chemistry_week_17_Parameter	sodium_17	NA	Only data on 15/01/2013
GMC16	Clinical_chemistry_week_17_Parameter	ALP_17	NA	Only data on 15/01/2013
GMC16	Clinical_chemistry_week_17_Parameter	ALT_17	NA	Only data on 15/01/2013
GMC16	Clinical_chemistry_week_17_Parameter	AST_17	NA	Only data on 15/01/2013
GMC16	Clinical_chemistry_week_17_Parameter	LDH_17	NA	Only data on 15/01/2013
GMC16	Clinical_chemistry_week_17_Parameter	amylase_17	NA	Only data on 15/01/2013
GMC16	Clinical_chemistry_week_17_Parameter	GLU_17	NA	Only data on 15/01/2013
GMC16	Clinical_chemistry_week_17_Parameter	creatinine_17	NA	Only data on 15/01/2013
GMC16	Clinical_chemistry_week_17_Parameter	albumin_17	NA	Only data on 15/01/2013
GMC16	Clinical_chemistry_week_17_Parameter	total_protein_17	NA	Only data on 15/01/2013
GMC16	Clinical_chemistry_week_17_Parameter	urea_17	NA	Only data on 15/01/2013
GMC16	Clinical_chemistry_week_17_Parameter	CHOL_17	NA	Only data on 15/01/2013
GMC16	Clinical_chemistry_week_17_Parameter	TG_17	NA	Only data on 15/01/2013
GMC16	Clinical_chemistry_week_21_Parameter	calcium_21	6,18E-05	NA
GMC16	Clinical_chemistry_week_21_Parameter	chloride_21	3,68E-01	NA
GMC16	Clinical_chemistry_week_21_Parameter	iron_21	7,49E-01	NA
GMC16	Clinical_chemistry_week_21_Parameter	iron_binding_21	8,75E-01	NA
GMC16	Clinical_chemistry_week_21_Parameter	lactate_21	5,13E-04	NA
GMC16	Clinical_chemistry_week_21_Parameter	phosphate_21	8,75E-01	NA
GMC16	Clinical_chemistry_week_21_Parameter	potassium_21	6,04E-01	NA
GMC16	Clinical_chemistry_week_21_Parameter	sodium_21	7,79E-02	NA
GMC16	Clinical_chemistry_week_21_Parameter	ALP_21	3,12E-02	NA
GMC16	Clinical_chemistry_week_21_Parameter	ALT_21	1,55E-01	NA
GMC16	Clinical_chemistry_week_21_Parameter	AST_21	1,92E-01	NA
GMC16	Clinical_chemistry_week_21_Parameter	LDH_21	3,25E-01	NA
GMC16	Clinical_chemistry_week_21_Parameter	amylase_21	6,53E-02	NA
GMC16	Clinical_chemistry_week_21_Parameter	GLU_21	4,79E-01	NA
GMC16	Clinical_chemistry_week_21_Parameter	albumin_21	5,56E-09	NA
GMC16	Clinical_chemistry_week_21_Parameter	creatinine_21	1,32E-30	NA
GMC16	Clinical_chemistry_week_21_Parameter	total_protein_21	2,12E-01	NA
GMC16	Clinical_chemistry_week_21_Parameter	urea_21	1,53E-03	NA
GMC16	Clinical_chemistry_week_21_Parameter	CHOL_21	8,41E-01	NA
GMC16	Clinical_chemistry_week_21_Parameter	TG_21	9,92E-01	NA
GMC17	ABR_Parameter	click	5,59E-01	NA
GMC17	ABR_Parameter	pip_6	6,88E-01	NA
GMC17	ABR_Parameter	pip_12	5,27E-01	NA
GMC17	ABR_Parameter	pip_18	6,87E-01	NA
GMC17	ABR_Parameter	pip_24	4,96E-01	NA
GMC17	ABR_Parameter	pip_30	6,07E-02	NA
GMC17	ABR_Parameter	Extens_stimulus_lvls	NA	Only data on 16/04/2013
GMC18	Dual_Energy_Xray_Absorptiometry_Parameter	body_length	3,91E-03	NA

GMC18	Dual_Energy_Xray_Absorptiometry_Parameter	body_size	7,06E-03	NA
GMC18	Dual_Energy_Xray_Absorptiometry_Parameter	body_type	7,02E-03	NA
GMC18	Dual_Energy_Xray_Absorptiometry_Parameter	FATmass_Xhead	1,23E-01	NA
GMC18	Dual_Energy_Xray_Absorptiometry_Parameter	FATmass_wholebody	1,23E-01	NA
GMC18	Dual_Energy_Xray_Absorptiometry_Parameter	LEANmass_Xhead	5,27E-01	NA
GMC18	Dual_Energy_Xray_Absorptiometry_Parameter	LEANmass_wholebody	5,39E-01	NA
GMC18	Dual_Energy_Xray_Absorptiometry_Parameter	SOFTmass_Xhead	7,86E-02	NA
GMC18	Dual_Energy_Xray_Absorptiometry_Parameter	SOFTmass_wholebody	8,56E-02	NA
GMC18	Dual_Energy_Xray_Absorptiometry_Parameter	BMD_Xhead	6,15E-02	NA
GMC18	Dual_Energy_Xray_Absorptiometry_Parameter	BMD_wholebody	1,53E-01	NA
GMC18	Dual_Energy_Xray_Absorptiometry_Parameter	bone_area_Xhead	2,37E-02	NA
GMC18	Dual_Energy_Xray_Absorptiometry_Parameter	bone_area_wholebody	2,89E-02	NA
GMC18	Dual_Energy_Xray_Absorptiometry_Parameter	bone_mass_Xhead	1,87E-02	NA
GMC18	Dual_Energy_Xray_Absorptiometry_Parameter	bone_mass_wholebody	2,85E-02	NA
GMC18	Dual_Energy_Xray_Absorptiometry_Parameter	lumbar_num	5,23E-03	NA
GMC19	Lung_function_Parameter	residual_vol	1,13E-01	NA
GMC19	Lung_function_Parameter	expiratory_vol	5,31E-01	NA
GMC19	Lung_function_Parameter	forced_expiratory_vol	5,08E-01	NA
GMC19	Lung_function_Parameter	tidal_vol	1,47E-13	NA
GMC19	Lung_function_Parameter	lung_capacity	3,18E-01	NA
GMC19	Lung_function_Parameter	vital_capacity	7,16E-01	NA
GMC19	Lung_function_Parameter	forced_capacity	4,99E-01	NA
GMC19	Lung_function_Parameter	inspiratory_capacity	5,93E-01	NA
GMC19	Lung_function_Parameter	residual_capacity	1,00E-01	NA
GMC19	Lung_function_Parameter	chord_compliance	6,90E-01	NA
GMC19	Lung_function_Parameter	dynamic_compliance	6,54E-01	NA
GMC19	Lung_function_Parameter	resistance	9,55E-01	NA
GMC19	Lung_function_Parameter	peak_flow	5,26E-01	NA
GMC20	All_immunoglobulins_week_17_21_Parameter	IgM_17	NA	Only data on 15/01/2013
GMC20	All_immunoglobulins_week_17_21_Parameter	IgM_21	2,27E-05	NA
GMC20	All_immunoglobulins_week_17_21_Parameter	IgA_17	NA	Only data on 15/01/2013
GMC20	All_immunoglobulins_week_17_21_Parameter	IgA_21	1,15E-13	NA
GMC20	All_immunoglobulins_week_17_21_Parameter	IgG1_17	NA	Only data on 15/01/2013
GMC20	All_immunoglobulins_week_17_21_Parameter	IgG1_21	1,14E-02	NA
GMC20	All_immunoglobulins_week_17_21_Parameter	IgG2a_17	NA	Only data on 15/01/2013
GMC20	All_immunoglobulins_week_17_21_Parameter	IgG2a_21	1,74E-01	NA
GMC20	All_immunoglobulins_week_17_21_Parameter	IgG2b_17	NA	Only data on 15/01/2013
GMC20	All_immunoglobulins_week_17_21_Parameter	IgG2b_21	3,73E-01	NA
GMC20	All_immunoglobulins_week_17_21_Parameter	IgG3_17	NA	Only data on 15/01/2013
GMC20	All_immunoglobulins_week_17_21_Parameter	IgG3_21	3,20E-06	NA
GMC20	All_immunoglobulins_week_17_21_Parameter	IgE_17	NA	Only data on 15/01/2013
GMC20	All_immunoglobulins_week_17_21_Parameter	IgE_21	7,45E-01	NA
GMC20	All_immunoglobulins_week_17_21_Parameter	aDNA_17	NA	Only data on 15/01/2013
GMC20	All_immunoglobulins_week_17_21_Parameter	aDNA_21	5,31E-02	NA
GMC20	All_immunoglobulins_week_17_21_Parameter	RF_17	NA	Only data on 15/01/2013
GMC20	All_immunoglobulins_week_17_21_Parameter	RF_21	3,81E-05	NA
GMC21	Organ_weights_at_section_Parameter	tibia_length	2,38E-07	NA
GMC21	Organ_weights_at_section_Parameter	heart_wt	6,54E-01	NA
GMC21	Organ_weights_at_section_Parameter	liver_wt	8,87E-01	NA
GMC21	Organ_weights_at_section_Parameter	spleen_wt	9,04E-01	NA
