# Supplementary material for: A comprehensive and comparative phenotypic analysis of the collaborative founder strains identifies new and known phenotypes
Source: Mamm Genome. 2020 Feb 14;31(1):30–48. doi: 10.1007/s00335-020-09827-3 (PMC7060152; doi:10.1007/s00335-020-09827-3)
Supplement: Supplementary file 12 — Supplementary file12 (PDF 177 kb) [file 335_2020_9827_MOESM12_ESM.pdf]

Table S7

Strain	Parameter	Strain_mean	129vsStrain_t-test	129vsStrain_N
A/J	center_distance_total	0,265625	0,016459119	37
C57BL/6J	center_distance_total	24,8	4,12E-48	3
CAST/EiJ	center_distance_total	25,45135135	2,54E-52	3
NOD/ShiLtJ	center_distance_total	33,61764706	2,28E-74	2
NZO/HlLtJ	center_distance_total	7,214705882	0,000701774	20
PWK/PhJ	center_distance_total	16,06904762	5,76E-25	4
WSB/EiJ	center_distance_total	24,84242424	1,02E-48	3
A/J	rears_total	1,21875	0,168021374	107
C57BL/6J	rears_total	135,09375	1,51E-39	3
CAST/EiJ	rears_total	125,2432432	6,90E-37	3
NOD/ShiLtJ	rears_total	157,0588235	1,98E-50	3
NZO/HlLtJ	rears_total	58,58823529	6,56E-09	7
PWK/PhJ	rears_total	135,6190476	2,22E-43	3
WSB/EiJ	rears_total	81,93939394	6,50E-17	4
A/J	center_rest	0	0,855848116	5334
C57BL/6J	center_rest	11,98125	1,16E-10	6
CAST/EiJ	center_rest	5,975675676	0,000864719	18
NOD/ShiLtJ	center_rest	10,85882353	2,76E-09	6
NZO/HlLtJ	center_rest	4,317647059	0,02024611	35
PWK/PhJ	center_rest	12,72380952	5,17E-13	5
WSB/EiJ	center_rest	5,381818182	0,003617141	22
A/J	iron_21	35,25090909	0,015573352	28
C57BL/6J	iron_21	21,06409091	7,36E-12	5
CAST/EiJ	iron_21	34,93173913	0,025919805	33
NOD/ShiLtJ	iron_21	32,09809524	0,796445377	2282
NZO/HlLtJ	iron_21	25,7152381	6,24E-05	11
PWK/PhJ	iron_21	28,45666667	0,026890017	32
WSB/EiJ	iron_21	37,50636364	9,33E-05	11
A/J	chloride_21	108,7590909	0,164881969	79
C57BL/6J	chloride_21	107,6	0,970314409	108726
CAST/EiJ	chloride_21	111,7304348	1,98E-06	8
NOD/ShiLtJ	chloride_21	104,9952381	0,00332802	18
NZO/HlLtJ	chloride_21	104,2818182	0,000168343	12
PWK/PhJ	chloride_21	110,3904762	0,001318481	15
WSB/EiJ	chloride_21	110,9727273	9,95E-05	11
A/J	calcium_21	2,431818182	0,005194933	21
C57BL/6J	calcium_21	2,393181818	4,47E-05	10
CAST/EiJ	calcium_21	2,341304348	7,73E-09	6
NOD/ShiLtJ	calcium_21	2,429047619	0,004295165	20
NZO/HlLtJ	calcium_21	2,499545455	0,654733081	782
PWK/PhJ	calcium_21	2,365714286	9,20E-07	7
WSB/EiJ	calcium_21	2,335	3,48E-09	6
