# Supplementary material for: A comprehensive and comparative phenotypic analysis of the collaborative founder strains identifies new and known phenotypes
Source: Mamm Genome. 2020 Feb 14;31(1):30–48. doi: 10.1007/s00335-020-09827-3 (PMC7060152; doi:10.1007/s00335-020-09827-3)
Supplement: Supplementary file 13 — Supplementary file13 (PDF 171 kb) [file 335_2020_9827_MOESM13_ESM.pdf]

Table S8

explanatory variable	Df	Sum Sq	Mean Sq	F value	Pr(>F)
strain	7	7,708792004	1,101256001	120,174953	1,41E-60
sex	1	1,683431809	1,683431809	183,7050954	2,08E-28
bw	1	0,492773357	0,492773357	53,77406794	1,04E-11
